# Maternal and child surveillance in peri-urban communities: Perceptions of women and community health workers from Pakistan

**DOI:** 10.1371/journal.pgph.0000295

**Published:** 2022-04-26

**Authors:** Ayesha Khalid, Rehan Adamjee, Saima Sattar, Zahra Hoodbhoy

**Affiliations:** Department of Paediatrics and Child Health, Aga Khan University, Karachi, Sindh, Pakistan; University of the Witwatersrand, SOUTH AFRICA

## Abstract

Community health workers (CHWs) in maternal, newborn, and child health (MNCH) programs play an important role in demographic surveillance activities; however, there is lack of literature regarding the community and CHWs’ perceptions about these activities. The purpose of this study was to explore perceptions of married women of reproductive age (MWRA) regarding the role of CHWs involved in maternal and child surveillance and explore facilitators and barriers for CHWs involved in surveillance activities. A qualitative study was conducted in five peri-urban surveillance sites along the coastal belt of Bin Qasim Town, Karachi, Pakistan. In-depth interviews were conducted with 25 randomly selected MWRAs and 15 CHWs. A thematic analysis was performed to explore perceptions, barriers, and facilitators of the study participants about maternal and child surveillance activities. The results showed that MWRAs perceived surveillance CHWs as service providers with regards to standard counselling i.e. importance of antenatal care, nutrition, immunization, and distribution of iron and folic acid tablets to pregnant women, child growth assessment, and referral of sick children to the health facility. Trust in the CHWs was an enabler for MWRAs, whereas lack of incentives was cited as a barrier to share their health data. CHWs perceived themselves as a bridge in liaising community with the primary health care facility. They highlighted an enabling environment such as appreciation, supportive supervision, training, and utilization of digital data collection tools as facilitators for their work. Low health literacy of the communities, lack of provision of incentives by CHWs to the community, and facility-based experiences of the community were reported as barriers. Surveillance CHWs are an integral link between the health facility and MWRAs. Hence an enabling environment may lead to improved health service delivery, translating into meaningful impact for the mother and child.

## Introduction

To achieve universal health coverage, Sustainable Development Goal (SDG) 3 emphasizes the importance of quality essential services such as reproductive, maternal, and newborn care [1]. Community health workers (CHWs) are considered the cornerstone for promoting primary health care and are recognized as key players in achieving these SDGs. They play an important role in community-based care for maternal, newborn, and child health (MNCH) interventions, and creating liaison with facility-based care, thus saving the lives of mothers and children [2]. CHWs are mainly responsible to provide preventive and promotive health messages, distributing basic health supplies (iron and folic acid supplements, contraceptives, etc.), and acting as a linkage between far-flung communities and health centers [3]. A recent systematic review of the scope of work of CHWs highlights this cadre’s role as change agents in the communities they serve by connecting communities to care facilities, providing case-based management of common illnesses, and empowering communities to address the social determinants of health [4]. With a dire shortage of trained healthcare providers in Pakistan, the minimally trained CHW serves as a catalyst between the community and skilled care providers to improve outcomes [3,5].

CHWs in MNCH programs play a vital role in demographic surveillance activities at several research field sites in low-middle income countries (LMICs). One successful example of this is the ’MATLAB’ site in Bangladesh. ’MATLAB’ was initiated in 1966 which aimed to develop a registration system for married women of reproductive age (MWRAs) and under 5 children to provide MNCH interventions [6]. MATLAB program reported a decrease in maternal and under 5 years mortality from 412 to 131 per 100,000 pregnancies and 59.1 to 43.5 per 1000 live births respectively [7]. The Kilifi Health and Demographic Surveillance System (KHDSS) was established in 2000 by the KEMRI-Wellcome Trust Research Program (KWTRP) which reported a reduction in under-5 mortality from 13.1 to 7.4 deaths per 1000 person-years of observation (PYO) from 2013 to 2015, with a further decline of 5.4 deaths per 1000 PYO from 2015 to 2018 [8]. These programs not only showed improvement in health outcomes but also yielded critical insights for guidelines and best practices for community-based MNCH programs [8,9].

Although, the indicators for success of CHWs in LMICs are well established (7, 8), the perceptions of the communities towards them is relatively understudied. According to a study conducted in Kenya, CHWs were perceived by the community as facilitators for care seeking and health education in the community [10]. The communities also felt that CHWs had good communication skills and ensured confidentiality. However, participants indicated that due to the incentives offered by CHWs, communities were temporarily driven to obtain compensation rather than improve their health [10]. Participants also stated that CHWs were not always equipped with adequate health information (due to lack of formal training) and that they were unable to favorably influence care seeking behaviors [10]. Grant et al. identified that lack of trust and the possibility of breach in their health information was the primary barrier towards the acceptability of CHWs [11].

It is equally important to understand the perceptions of CHWs regarding their work. A study from Pakistan reported that CHWs felt respected and social prestige in the community as a result of their role as care providers [12]. On the other hand, lack of career advancement and a sense of being undervalued in the health care system were cited as major barriers by CHWs [10,13]. Grossmann-Kahn et al. reported that CHWs described a lack of knowledge about preventive treatments among community members, misunderstandings or failure to provide timely information to CHWs as significant barriers to their performance [14].

Although these community-based research surveillance sites provide valuable data on burden of maternal and child morbidity and mortality they require a relatively high amount of data collection from the community as compared to standard CHW programs. The successful functioning of these sites necessitates motivated CHWs as well as receptive, tolerant communities. However, there is paucity of literature on the perception of surveillance activities by the population as well as on the CHWs involved in these activities, both of which are critical for the long-term sustainability and functioning of these sites.

The purpose of this study was (1) to explore perceptions of MWRAs regarding role of CHWs involved in maternal and child surveillance in their area and (2) explore facilitators and barriers for CHWs involved in surveillance activities.

## Materials and methods

### Study design and setting

A qualitative study was conducted in five peri-urban surveillance sites along the coastal belt of Bin Qasim Town, Karachi, Pakistan from October 2020 to January 2021. Comprising of a population of approximately 400,000, these sites are managed by the Department of Pediatrics and Child Health at Aga Khan University since 2010 [15]. Surveillance CHWs are females aged ≥ 18 years, with atleast 12 years of education, and are residents of the communities where they work. They visit the households once every quarter to enquire regarding vital events such as pregnancies, births, maternal and child deaths, and migrations, as well as to provide standard counseling messages such as the importance of antenatal care, nutrition, and childhood immunizations. This information is collected on a custom-built Android-based application installed on a tablet. The surveillance sites have been leveraged to play an integral role in several large-scale studies such as Aetiology of Neonatal Infection in South Asia (ANISA), Global Enteric Multicenter Study (GEMS), and Alliance for Maternal and Newborn Health Improvement (AMANHI) [16–18].

### Study participants

A system-generated list of MWRAs was acquired from the surveillance database prior to data collection. MWRAs aged 18–49 years and those who had been residents of the catchment area for two consecutive years were eligible for the study. Once extracted, the research team sorted the list in an ascending order to randomly select 15 women per field site. If a selected woman was unwilling to participate, the next woman on the list was approached. Eventually, we conducted 5 interviews with MWRAs per site as saturation was reached.

CHWs included in the study had at least 2 years of relevant experience from selected field sites. We interviewed 3 randomly selected CHWs per site as saturation was reached.

### Data collection

Written informed consent and all interviews with MWRAs were conducted at their homes while consents and interviews with CHWs were conducted at the primary health care facilities located at the field site. In-depth interviews (IDIs) using a semi-structured guide and each lasting 30–45 minutes were conducted in Urdu language by trained researchers with experience in qualitative research. Each interview was audio recorded. The study was approved by Aga Khan University Ethical Review Committee (ERC #2020-5451-14373).

### Data analysis

Data analyses followed the manual Thematic Analysis approach [19]. First, the interviews were transcribed verbatim into Roman Urdu to ensure the transcripts did not lose their true meaning. Transcriptions were conducted by SS and quotes used in the paper were translated into English by AK. All Urdu transcripts were initially coded by AK and RA. All codes were matched and compared by two authors and in case of disagreement, they were further discussed with the fourth author (ZH) to reach a mutual consensus. Later, the codes were merged into pre-identified themes and emerging sub-themes related to perceptions of MWRAs and CHWs about their role in surveillance activities, facilitators, and barriers. Rigor was ensured through the implementation of various strategies including debriefing with the field team to ensure interpretative accuracy, observations, and notes created throughout data collection. Due to the familiarity with research sites, the authors used a personal reflexive approach at each stage of the analysis to avoid any researcher bias which may influence the results.

Data about demographic information of MWRAs and CHWs were collected during interviews. Mean, standard deviation (± SD), frequency and percentages were calculated.

## Results

We conducted a total of 40 interviews, 25 with MWRAs and 15 with CHWs (i.e. 5 and 3 per site respectively). The mean age of MWRAs was 30 ± 6 years. Seventy-two percent of MWRAs (n = 18) had some level of education, with only 16% (n = 4) involved in gainful employment such as embroidery and stitching. The mean age of CHWs was 31± 8 years with 40% (n = 6) having graduate-level education or higher. The mean work experience of these CHWs in surveillance activities was 5.6 ± 2.5years. The demographic details of the study participants are reported in Table 1.

**Table 1:** Baseline characteristics of study participants.

Variables	Married Women of Reproductive age (N = 25)	Community Health Workers (N = 15)
**Age in years, Mean ± SD**	30 ± 6	31± 8
**Education, n (%)**		
No Education	7 (28)	-
Primary	8 (32)	-
Secondary	7 (28)	5 (33.3)
Higher Secondary	1 (4)	4 (26.6)
Graduation or Higher	2 (8)	6 (40)
**Occupation, n(%)**	*** ***	
Unemployed	21 (84)	-
Employed	4 (16)	-

The thematic analysis centered around three major a priori themes for each set of participants (i.e. MWRAs and CHWs): (i) Perceptions (ii) Facilitators (iii) Barriers. For MWRAs, facilitators were factors that motivated them to share health information with the surveillance CHWs while the barriers were reasons which prevented them from doing so. For CHWs, facilitators and barriers were factors that affected their work performance.

### MWRAs

#### Perception of MWRAs about surveillance CHWs

There were varied views regarding the work of CHWs that were captured during the interviews with MWRAs. MWRAs appreciated the CHWs by acknowledging their kind attitude, knowledge given regarding antenatal care and newborn handling, and anthropometric assessment of their child during home visits.

“*I like their care and visits to us; when I was pregnant*, *they visited me regularly*, *and asked about my health*, *how my condition was*, *how much time was left for delivery*. *In our country*, *they are for us*, *they take care of us*.*” [Bhains Colony-MWRA]*

On the other hand, few MWRAs expressed their disappointment from CHWs as they felt that there was no true benefit of the CHW visit and the only purpose of their visit was data collection. Others were annoyed for not receiving any tangible benefits such as medicine or transport facility for them or their children to visit the health facility.

“*They only visit for data entry and then never revert back for anything; we say that you enter so much of our data but give us no services*, *you just enter the data and leave; don’t we need anything*, *we go to private hospitals for medicines*, *and take our kids there too*, *obviously*, *we should get facilities for this right…” [Ibrahim Hydri -MWRA]*

#### Facilitators for MWRAs

The majority of the MWRAs stated that the CHWs demonstrated *“Acha Ikhlaq” (*good behavior*)* during routine household visits. Familiarity with CHWs as part of their community and trust in the organization they belonged to was stated as key motivators by MWRAs to share their personal and health information.

“*We feel good that the workers come on their scheduled time*, *we discuss our issues with them*. *We tell them about our problems*..*the workers belong to our own area*, *which we like…”[Rehri Goth- MWRA]*

#### Barriers for MWRAs

Few participants shared that they refused to give any information when extended family members such as mother or sister- in law were around or when they were busy with household chores. Since the main area of interest of the CHW is recording pregnancy and its outcomes, non-pregnant women felt less motivated to share their information. The lack of any tangible incentives also discouraged the MWRA from sharing information.

“*They [CHW] come to ask; one or two times I refused to share details and said I don’t have young kids; since I refused*, *they turned back” [Ali Akbar Shah- MWRA]*

### CHWs

#### Perception of surveillance CHWs about their work

CHWs expressed their role as a bridge between their community and the health care facility. They believed that they played a pivotal role in linking MWRAs to the health facility, sharing emergency numbers for transport, and prompt referral of sick children for timely management.

“*We guide them that delivery should occur in hospital*, *our center provides treatment and vaccinations services for children*, *you can visit [facility] to take services from the center” [Rehri Goth-CHW]*

CHWs described their work as a learning opportunity to expand their own knowledge about pregnancy, nutritional counseling for mother and child, and vaccination so that they could share this information with the community. They also shared that the nature of their work improved their communication skills and increased their confidence to convey health messages to the community. They expressed a feeling of fulfillment on how their work had provided them with an opportunity to serve their own community. They felt that they were well recognized, trusted, and in a privileged position to help improve the health status of their community

“*Surveillance is good work; we have a very good understanding with the community…*. *they trust us*, *we are happy that we are able to help someone [Bhains Colony -CHW]”*

#### Facilitators for CHW

CHWs expressed that training around communication skills could help them in delivering messages to the households effectively. They also expressed that appreciation of work by supervisors would motivate them to improve their work. A few CHWs shared that digitization of the surveillance work has increased their efficiency in capturing information regarding vital events related to the mother and child.

“*Previously we used to focus on hard copies; all paperwork is finished now*.. *now we enter all data in tablet*, *so it is not difficult now*.*” [Rehri Goth-CHW]*

CHWs also highlighted services like transportation services to the facility and ultrasound services available which helped establish trust between CHWs and the community.

"*If people are benefiting from center’s facilities*, *then they’ll respond better to us [CHWs] if they are satisfied then they will encourage other people that ’ we benefited from the center’s services if you visit there you will also benefit from its services*.*” [Ibrahim Hydri-CHW2]*

#### Barriers for CHWs

Many CHWs expressed that MWRAs were unable to share accurate information regarding their age, last menstrual period date, or date of delivery. Also, some MWRAs tend to hide information regarding early pregnancy due to myths in the community, which affects their work.

Some CHWs reported safety concerns that acted as barriers to their work. They stated that in certain surveillance areas, they experienced name-calling which made them uncomfortable during fieldwork. They also reported fear of snatching of their personal belongings or tablets in these areas.

Most CHWs expressed frustration due to limited resources especially in terms of vehicle availability. They felt that they were held accountable for delays in reaching daily targets when a lack of resources hindered them from obtaining their goals. They also expressed lack of incentives such as vouchers and/or medicines which may be offered by other projects which lead to refusal of certain community members to offer information.

“*So they say*, *there is no benefit for us*. *If you give us benefit*, *then we will give our data” [Ibrahim Hydri Extension-CHW]*

Many CHWs believed that the behavior of facility-based staff along with long waiting times at the health facility had negative repercussions on surveillance activities.

“*When we go on our door to door visits they refuse to provide data because when they go to the center*, *they (staff) don’t talk to them nicely and they have to wait too much [Rehri Goth -CHW]”*

## Discussion

The current study reports the perceptions of MWRAs and the CHWs regarding MNCH surveillance activities in peri-urban marginalized communities in a low-resource setting. Both MWRAs and CHWs themselves reported this cadre as the main source for maternal and child health information and linking them to health facility services.

The key perceptions of MWRAs regarding CHWs were as basic care providers for themselves and their young children at the household level. Our findings were supported by other studies where MWRAs showed gratitude towards CHWs attitude and routine activities in contributing towards primary health care. This was also confirmed when MWRA’s acknowledged that during CHWs routine visits, they provided services in the form of dispensing of iron and folic acid tablets to pregnant women, monitoring newborn growth, and standard counselling for pregnant women [20,21]. Apart from this, surveillance CHWs connect community women to health care facilities for their antenatal and postnatal visits, however, MWRAs did not emphasize this aspect of CHWs work during the interviews. Studies conducted in LMICs have reported that CHWs encourage the community to avail services which in turn improved health outcomes such as skilled deliveries, improved immunization, and timely referral of sick children. This approach has led to improving lives and reduction in child mortality at certain study sites [22,23].

In the current study, MWRAs felt that routine surveillance visits enabled them to trust and build relations with CHWs for communication of health issues. These findings were similar to a study conducted in South Africa where CHWs were able to rebuild the broken trust of the community by developing personal relationships and spending time with the community [24]. In addition, the present study also demonstrated the MWRAs trust in surveillance CHWs due to their association with a well-established organization.

It was also commonly observed that MWRAs thought the provision of incentives (such as medicines, transportation to health facilities) should be part of the CHWs regular household visits as they should be compensated for providing data. However, such demands are beyond the scope of surveillance activities which include capturing pregnancies, births, and deaths in the community. A similar perception was also reported by Glenton et al where the community showed dissatisfaction from CHWs and considered their work insufficient when they provided only promotional health services [21].

At the community level, CHWs perceived themselves as important pillars of primary health care at community level as they act as a bridge between individuals and the health facility. Their work provided them an opportunity to help underprivileged communities, where access to basic health care services is limited. Mlotshwa et al. reported that being from the same community strongly enhances CHWs sense of belonging [25].

At a personal level, CHWs believed that routine surveillance provided a platform for improving their own knowledge and skills which was a key motivating factor for their work.

CHWs reported supportive supervision, respect, and appreciation from the community, knowledge and communication skills sessions, and the digitalization of data collection as facilitators for their current work. These facilitators have been reported by other studies where the work of CHWs, may be improved through defined catchment areas, clear job descriptions, support and ownership of the community, regular training, supervision, and monitoring system [20,26].

The barriers reported by CHWs in this study were similar to the findings of other authors including the community’s low health literacy, disrespectful attitude, safety and security concerns, lack of provision of tangible benefits by the CHWs to the community, and limited resources that influence routine surveillance activities [14]. The quality and number of services provided at health care facilities also played a pivotal role, as the experiences of MWRAs at the facility shaped their relationship with the CHWs during the home visit [14].

Similar work on CHWs performance in LMICs has suggested that ongoing trainings to improve knowledge and skills, supportive supervision, strong coordination between healthcare facilities and CHWs, and community in terms of stakeholder engagement and managing realistic community expectations could lead to improved CHW performance [27,28]. The current study has further strengthened these insights that a secure and conducive work environment, training opportunities for CHWs and fostering trust between MWRAs and CHWs would empower them to ensure that the overarching goals of MNCH surveillance activities in LMICs are met.

Due to the nature of the interactions with the community through regular home visits, it is important to understand the perspectives of MWRAs and CHWs involved in surveillance activities. The strength of this study was to provide detailed insights of both MWRAs and CHWs about these activities in low-resource settings. However, this study was conducted in peri-urban communities which are research sites for the Aga Khan University therefore the results of the study may not be generalizable to other parts of Pakistan or other countries. Another limitation was that the perception of non-married women was not included as part of the study.

## Conclusion

Surveillance initiatives in Asia and Africa have strongly demonstrated that MNCH surveillance can help improve mother and child health indicators. However, to do so, it is essential to understand the perception of stakeholders (i.e. MWRAs and CHWs) involved in household surveillance in order to create an enabling environment for preventive home-based counseling and high-quality accurate data that can eventually translate into meaningful and impactful results in terms of lives saved.

## Supporting information

S1 Dataset This contains data from all in-depth interviews. (PDF)
