# Supplementary material for: Maternal and child surveillance in peri-urban communities: Perceptions of women and community health workers from Pakistan
Source: PLOS Glob Public Health. 2022 Apr 26;2(4):e0000295. doi: 10.1371/journal.pgph.0000295 (PMC10021568; doi:10.1371/journal.pgph.0000295)
Supplement: S1 Dataset — (PDF) [file pgph.0000295.s001.pdf]

AG-IDI-01

RA: jee NAME aap ki umar kitni hai

MT: meri 45 saal

RA: 45 saal hai aur aap ki taleem

Mt: 5 class tak

RA: 5 class tak aur koi kaam karti hain ghar ka

MT: ghar ka hi bas

RA: bas ghar ka hi kaam karti hain kitne bache hain aap k

MT: mere 4

RA: 4 bache hain sab se bara wala

MT: sab se bara wala 21

RA: 21 saal ka hai aur chote

MT: 13 saal ka

RA a: 13 saal ka hai aur baaki 2 aur chote hain

MT: nahi nahi us se bhi choti beti hai 3 saal choti hai us se chota beta 3 saal chota hai aur us se chota beta 3 saal 3 3 saal ka fark

RA: acha thek hai acha mujhe yeh bataaiyega joh hamari health worker hain joh aap k ghar aati hain toh woh baqaidgi se ghar arahi hain aap k yahan

MT:maloomaat karne aati hain

RA: maloomaat karne aati hain toh kitne kitne arse baad aati hain

MT: aksar aati rehti hain

RA: aksar aati hain toh kitna arsa matlab ek maheeney mein do maheeney mein ya haftey mein

MT: 15 din baad aati hain

RA: 15 din mein aati hain acha toh who aap se kia sawal poochti hain

MT: yehi poochti hain aap MT hain aap k ghar mein chote bache toh nahi hai mein ne kaha nahi chota bacha nahi hai toh chalo thek hai phir chali jaati hain

RA: pooch k chali jaati hain

MT: jee

RA: k chota bacha hai k nahi hai acha toh aap k sehat k hawale se poochti hain

Mt: nahi

RA: yeh toh bachon k hawale se bata diya na k aap k ghar mein kitne bache hain toh bachon ki sehat k hawale se kuch poochti hain aap se

Mt: nahi

RA: aap k hawale se

Mt: nahi sirf bachon ki maloomaat leine aayin k bache kitne hai

RA: acha hamal mein jab aap thi tab aate thy

Mt: nahi mein jab kahin aur rehti thi yahan pe 3 4 saal hogaye hain

RA: 3 4 saal hogaye hain acha kabhi aap se hamal k baare mein poocha ho k abhi hamal se toh nahi hain

Mt: nahi

RA: aisa bhi kuch nahi poocha aap se ya poocha ho

Mt poocha ho shayad yaad na ho hosakta hai

RA: acha aur yeh k aap k kitne bache hain aur koi maloomaat li thi

Mtt: yehi k bache kitne hain kitne bare hain

RA: acha toh aap ne kaha tha k aati hain poochti hain phir chali jaati hain matlab koi maloomaat nahi deity aap ko

Mt: bachon k liye aati polio wali hoti hain

RA acha toh batati toh hongi k mein kahan se aayi hun aga khan se hamara center bhi hai

Mt batati hain

RA: batati hain acha aap keh rahi hain mujhe 3 saal hogaye hain yahan par un 3 saalon mein tab bhi in ka aana jaana hua tha na

Mt: haan aati rehti hain aksar woh aati hain maloomaat karne aap MT hai aap k kitne bache hain yeh maloom kar k chali jaati hain

RA: jahan aap pehle rehti thi aap ne kaha k idhar mein hamal mein nahi thi toh us waqt jahan aap rehti thi kahan rehti thi

Mt: 5 number pe

RA: 5 number pe rehti thi

Mt: wahan aisa koi nahi aaya

RA: wahan aisa koi nahi aaya maloomaat karne k liye

Mtt: koi nahi aaya

RA: aap ko center ka pata tha k hamara aga khan ka bhi idara hai

Mt: nahi jab yeh aayin thi tab hamein maloom para hai

RA a: haan in k aane k baad aap ko maloom para thek hai jab hamal mein thi us waqt thi is waqt jab aayin poochne aayin k hamal mein nahi hai aap

Mt: jee

RA: acha toh aur koi aap ki sehat k hawale se koi maloomaat li aap ki tabiat k hawale se

Mt: nahi aise toh koi baat nahi

RA: aisa kuch bhi nahi poocha aap se

Mtnahi

RA: aur kabhi aisa kaha ho kabhi agar bache ki tabiat kharab hojaaye toh le k ajaayein is tarhan se kuch

Mt: nahi nahi koi chota bacha hi nahi hai

RA: acha isi wajha se

Mt: bare bache hain na

RA: bare bache hain toh woh pehle bhi poochne nahi aati thi

Mt: muje yahan aaye huye 3 4 saal toh huye hain

RA: 3 4 saal hogaye hain

Mt: bache bare thy chote nahi thy

RA: chote nahi thy

Mtt: aga khan mein chote bachon ka elaaj hota hai na toh is liye

RA: acha is liye

Mt: haan

RA: sahi toh kabhi dikhaane k liye aap wahan nahi gayin

MT: nahi

RA: chote thy tab bhi nahi

MT: nahi kabhi nahi gaye

RA gaye hi nahi toh phir jaate kahan thy aap

MT: jab hum 5 number pe thy na toh 5 number pe bachon ko sath le gaye ya private hispatal

RA: toh koi aap ki joh aas paas mein rehti hain unhon ne nahi bataya aap ko k hamara bhi ek aga khan ka idaara hai

Mt: nahi yahan se kisi ne nahi bataya aage meri bhabi rehti hain toh apni bachi ko wahin le jaati hain toh unhon ne bataya tha k yahan pe aga khan hai bachon ka toh bachi ko wahin dikhaati hun

RA: yeh kab ki baat hai

Mtt: kafi time hogaya jab main dosray ghar rehti thi

RA: acha: jab aap wahan thi tab ki baat hai na

Mtt: haan

RA: unhon ne tab bataya tha

Mt: nahi yeh yahan a k pata chala

RA: acha toh jab worker aap se poochne aati hain aap k bachon k baare mein is k baare mein toh woh koi idaare k hawale se baat karti hain

Mt: nahi

RA: k hamara idara hai yahan idhar la k dikhaayein is tarhan se

Mtt: nahi sirf bataya k mein aga khan se aayi hun

RA: acha woh pooch k chali jaati hain toh aksar woh jab aati hain toh kis kisam k sawal karti hain aap se

Mt: bas aap idhar rehte ho na aap ka naam MT hai mein ne kaha hun aap k kitne bache hain mein ne kaha 4 bache bache hain bare hain na abhi bas yehi pooch k chali jaate hain

RA acha pooch k chale jaate hain acha jab woh pooch rahi hoti hain toh kabhi aap ne unse sawal kiya hai k sab kuch kyun pooch rahi ho

Mt: nahi

RA wajha kyun nahi poocha

Mt: boli mein aga khan se aayi hun aise maloom karne aayi hun bachon ka elaaj hota hai na

RA: hmm

Mt: bhabi ko bataya bachon k elaaj karwana ho toh wahan ajana

RA acha toh lekin ek hota hai na hamare bachon k hawale se poochne araha hai yeh toh hum k bhae kyun pooch rahi ho aap ne nahi poocha acha is k elawa koi aur k same wohi larkiyan aati hain ya alag alag larkiyan aati hain

MT: nahi alag alag bhi aati hain

RA: acha toh who sirf aap ko batate hain k aga khan se aaye hain toh aap maan leite hain

MT: jee

RA: matlab aap logon ne kabhi khud se kuch socha k hum zara deakhein k waqai mein larkiyan arahi hain

Mt: nahi aisa kabhi nahi socha

RA aise socha nahi kabhi bharosa hai aap ko un par

Mt: nahi bas aati hain pooch k chali jaati hain kabhi maloomaat bhi nahi di na kabhi is baare mein mein bataya ja k maloom karein ya deakhein

RA: hmm kuch nahi socha nahi is baare mein toh kia wajha hai k jab woh aati hain toh aap unhein bata deite hain toh bhi woh poochti hain

Mtt: jaise aap aayin hain toh bata deity hun (laugh)

RA kia kia wajha hai hum aaye hain hum bhi bata rahe hain yeh joh CHWs aati hain unhein bata deite ho

Mt: bas kuch nahi hai bata diya

RA: matlab koi aaya hai aap se poochne k liye toh woh bata deite ho acha bachon k hawale se jab aap akhari dafa hamal se thi aap ka bacha toh mashaallah 13 saal ka hai baaki poorani baat hogayi us k baad kabhi hamal se huyin

Mt: nahi

RA: us k baad se nahi huyi toh us waqt aap yahin raha karti thi

Mt: nahi nahi 5 number pe

RA: ali akber shah mein hi thin

Mt: ali akber

RA: matlab yahan isi elaake ki rehaishi thi

Mt: nahi nahi korangi 5 number

RA korangi 5 number mein thy acha acha toh kabhi aap k hamal mein CHW ne koi kirdaar ada kiya tha

Mt: nahi

RA kabhi woh aayi ho bataya ho acha bhae aap hamal se ho yeh kar lo woh kar lo is tarhan ki cheez

Mt: nahi

RA: kuch nahi kabhi aap ne khud se kaha ho k har maheeney jaati hai abhi mein hamal se hun aas paas kahin se suna ho

Mtt: nahi aisa kuch nahi suna

RA aap log aapas mein kabhi health worker k baare mein baat karte hain

Mtt: nahi

RA jaise abhi aap ne kaha k ek rishtedaar hai

Mt: jee unhon ne bataya tha

RA toh yeh baat kaise chirri thi k kia hua tha k matlab worker ka zikar aaya

MT: meri bhabi batati rehti hain k munni ko na wahin se elaj karwati hun munni k le aati hun toh who aksar wahin jaati hai na toh mere paas aayin kaha baaji pani de dou paidal aayi hun aga khan se munni ki dawai le kar aayi hun

RA: hmm

Mt: is wajha se pata chala

RA aise pata chala tha lekin aap khud se joh hai k CHW joh ghar pe arahi hoti hai koi sawal nahi karti na woh batati hain acha toh un ka rawaaiya kaisa lagta hai

Mt: acha lagta hai hamare sath sahi hota hai

RA: sahi hota hai kia cheez achi lagti hai

Mt: bas baat poochti hain hum jawab de deite hain phir chale jaate hain jab woh aati hain mein peeche lag jaati hun masti karti hun phir joh polio wali hain na masti karti hain is tarhan nahi karti

RA aise nahi karti yeh wali joh aati hain agar aap jawab de deite ho aage se woh jawab de deity hain aur kia cheez hain joh in pe bharosa lagta hai aur aap bata deite ho aga khan ki wajha hai k bhae aga khan ka naam leiti ahin kia yeh wajha hai jis ki wajha se bharosa karte hain

Mt: aga khan ka toh naam hai

RA aga khan ka naam hai is wajha se woh kehti hain k hum aga khan se aaye hain aap sunte hain aur unhein de deite hain kia kisi idaare k naam se bharosa hota hai un workers k upar health workers k upar aap logon ko pata hai acha yeh idaare hain joh mashoor hain toh agar un ka naam aap k saamne liya ja raha hai toh aap workers k upar joh bharosa kareinge jaise mein agar kahon k mein bohat parhi likhi hun thek hai lekin mujhe idaaron k baare mein nahi pata ek baat hai toh kia mein parhe likhe hone se faida uthaongi ya mein kahongi k nahi mujhe is idaare ka pata nahi hai joh bhi araha hai bata dou nahi batao joh woh sunte hain kisi idaare ka naam sunte hain k haan yeh mashoor hai kia us ki wajha se aap k ghar pe darwaaze pe agar health worker aati hai toh us ko aap log bharosa karte ho

Mt: jee karte hain bharosa karte hain andar bula liya

RA: hmm

RA: acha aga khan pe kyun bharosa hai

Mt (laugh)

RA: agar mein kahon mein agar aga khan ka naam na leiti joh hamari workers aati hain woh aga khan ka naam na lein aur kahin aur se aayein toh kia unhein bata deingi maloomaat bache k hawale se ya sehat k hawale se poochti hain

Mt: (laugh) nahi

RA: nahi bataayeinge kyun

Mt: (laugh) koi aga khan ka naam suna mutmaain hain k yeh aga khan ka hi hai

RA: aga khan ka naam suna hua hai

Mt haan

RA: kabhi aga khan jana hua hai hispatal se elaaj karwaana hua ho kabhi

Mt: kabhi nahi hua

RA: kabhi nahi hua kabhi kisi aur se woh kiya ho k bhae matlab kaise suna aga khan ka naam

Mt: mera taaya hai na woh 5 number pe kaam karta hai toh un k na waise elaaj hota hai sab bac

on ka bhi taaya ka bhi yahin se hi kyun k sab bachon ka company ki taraf se hi hai toh jab suna hai na achi hai aga khan k us se bache ka deite hain na taaya ka beta wahan say hota hai… naam suna hai aur deikha hai kafi bara haspatal hai..

RA: acha matlab k joh mujhe aap ki baat samjh arahi hai k ek toh yeh k worker aati hai agar who kisi bari idara jis ka naam hai us se arahi hai toh un pe bharosa karte hain aur aap log asaani se jawab de deite hain woh pooch rahi hoti hain de deite hain aur us idara mein aap log bhi is tarhan se bharosa karte hain

k kyun k aap ki family ka elaaj karaya gaya toh wahan acha response raha is wajha se acha mujhe yeh bataogi k chote bache joh nauzaida bache hote hain jab aap ka bacha hua us mein kabhi joh worker thi us mein koi kirdaar ada kiya ho kisi sehat k hawale se

Mt: nahi aise toh koi nahi hua

RA kabhi aap k ghar aayi ho bache ko naapa ho wazan kiya ho

Mt t: nahi wahan toh aisa nhai hua kuch bhi koi aata nahi tha wahan

RA: wahan nahi aata tha acha aur yahan par kabhi kisi aas paas paros ki aurat se kabhi suna ho haan worker a k ghar pe mere bache ko

Mt: nahi nahi

RA: naap k chali jaati hai aisa kuch nahi hua

Mt nahi aisa kuch nahi hua meri logon se ziada mulaaqaat nahi hoti bata rahi hun na k meri bhabi hai woh jaati hain

RA: hmm

Mttoh raste mein ghar parta hai toh who ajaati hain yahan par

RA: haan toh unhon ne nahi bataya k woh ghar pe a k munni ko deakh k chali jaati hain aisa kuch nahi hua

Mt: nahi aisa kuch nahi hua

RA: acha mujhe yeh bataayein k jab yeh aati hain aap k khayal se in ko sirf aap se poochna chaiye ya aap ko kuch batana bhi chaiye

Mtnahi woh pooch k chali jaati hai

RA: pooch k chali jaati hai koi maloomaat deni chaiye usay aap ki sehat k hawale se ya kisi cheez se cheez ka us se aur eham hosakta hai aur acha hosakta hai

Mthaan kyun k yeh toh achi baat hai un logon k liye jaise pehle k liye hamare sehat k hawale se kuch bataaye toh faida hai na hamare liye

RA: haan aap ki sehat k hawale se kyun k aaap parhe likhe nahi ho aap ko aisa lagta hai

Mt: jee

RA: toh agar kuch maloomaat pata chal jata hai toh achi baat hai

Mt: jee achi baat hai

RA: aur woh maloomaat kis k hawale se honi chaiye

Mt: is mamle mein toh ziada help honi chaiye haddiyaan kamzor jism dard karne lagta hai

RA: hmm

Mt: pareshan rehti hun na takleefon se

RA: hmm

Mt: dusra kaam wagera bhi nahi hota thak jaati hun foran

RA: hmm hmm

Mt: agar pata chal jaaye toh aage se kisi ko pehle se maloom hoga toh apni ittataat pehle karta

RA: hmm hmm

Mt: waqt se pehle band kar lein maloomaat pehle farhaam hote thy aurat ko toh sahi rahegi sehat

RA: kitni achi baatein kar rahi hain aap aur aap keh rahi hain mujhe nahi pata bohat achi baat aap ne ki k jaise sirf hamal se nahi agar jaise aurtein mann barti hai toh haddiyon mein dard horaha hai kamzori feel horahi hai us k hawale se bhi aap ko lagta hai k worker ko thori bohat maloomaat deini chaiye

Mt: jee

RA: aur koi cheez joh aurton k kayi masaail hote hain kuch is tarhan ki koi cheez aap k sath huyi ho us k hawale se aap chah rahi ho k haan pehle se baat pata hoti toh acha hota koi aisi cheez hai

Mt: logon k sath toh kitne kitne masaail hote hain mujhe baar baar mensis arahe hain ek hafte se kal raat tak phir mensis phir mensis ab yeh horaha hai mere sath

RA: acha

Mt: ek do maheeney se ziada horaha hai

RA: hmm hmm

Mt: toh mein ne kaha dawaai le k aaon jaana hi nahi horaha khud jana nahi horaha

RAa: acha haan toh matlab k joh worker arahi hain is hawale se joh irregular periods hote hain un k hawale se aap un ko batao kuch aap ko bata dein

Mt: haan

RA: ya koi dawaai aisi ho joh di jasake acha sahi hai acha aur koi cheez yeh bhi baat achi hai aur bachiyon k hawale se ya hamala aurton k hawale se koi cheez aap batana chahein un ko maloomaat deini chaiye

Mt: joh bhi pregnant hogi yeh saari maloomaat deingi toh achi baat hai

RA: un ko toh koi pata nahi hota larkiyon ko bhi takleefein bohat hoti hain dard bohat hota hai larkiyon ko mensis hote hain bachiyon ko itni takleef hoti hai woh hil jul bhi nahi kar sakti

Mt: mensis k doraan

RA: hmm hmm thek hai ek toh aap ne kaha jaise umar jin aurton ki barti jaati hai un ko kamzoriyaan hoti hain haddiyon mein dard hota hai un k hawale se maloomaat di jaaye regular joh periods hote hain larkiyon k bhi ya aurton k aur woh bachiyon ko aaj kal takleef bohat ziada hone lagti hai periods mein un k hawale se bhi thori bohat maloomaat di jaaye aur joh pehli dafa pregnant aurtein bachiyaan banti hain toh n ko ziada maloomaat deini chaiye aur us maloomaat mein jaise kia bataayein un ko koi aap ka mashwara hai k kis cheez pe ziada zor deina chaiye

RA: acha yeh mujhe bataayein kabhi aap ne aisa mehsoos kiya hai k aap batana chah rahe ho worker ko lekin rukawat hai nahi bata pa rahe kabhi aisa kuch hua

Mt: nahi aisa toh nahi hua

RA: aisa nahi hua koi rukawat mehsoos nahi hoti ghar walon ne kabhi manah kiya ho k yeh worker ko interview nahi deina

Mt: nahi nahi bache bolte hain ap apna batain

RA: acha acha bache aap ko bolte hain bache abhi school jaate hain

Mtt: jee do jaate hain dono ne parh liya

RA acha aap k kabhi shohar ne manah nahi kiya kabhi saas ne mahan kiya k kyun ajaati hain

Mt: nahi yahan pet oh kheir nahi actually saas hai nahi yahan shohar hai shohar manah karta hai (laugh)

RA: acha shohar manah karta hai

Mt: shohar manah karta hai aise aane nahi dou kisi ko

RA: acha toh kia kehte hain yeh joh worker kia kia raaye hoti hai aap manah hojaati ho jab manah kar deine se (laugh)

Mt: ek aat dafa toh manah kar diya haan woh aati hain mein bolti hun haan sahi hai hamare bache nahi hain gahr pe un ko manah kar diya toh chali jaati hain

RA:acha toh shohar manah karta hai nahi batana toh yeh keh deity hain acha acha sahi hai acha aur is k elawa koi aur cheez joh aap batana chahoge k hum is kaam ko aur behtar bana sakein

Mt: nahi aur kuch nahi

RA: aur kuch nahi bas yehi hai aur koi health worker k hawale se koi achaai koi achi baat joh aap ko achi lagti ho un k baare mein batana chaho

Mt: nahi bs

RA kuch acha lagta hai un k baare mein joh aati hain acha bs maloomaat karne aati hain aur kuch bura lagta

Mt: nahi nahi bura nahi lagta

RA: acha shukriya bohat bohat aap ka …….

AG-IDI-

SS: jee NAME mujhe yeh bataaiyega aap ki umar kitni hai

Zt: meri 30 saal umar

SS: 30 saal hai aur bache kitne hain aap k

Zt: hamara mashaallah 7 larka hai 4 larkiyaan hain

SS maahwaari aap ko kab aayein thi

Zt t: shadi thi samjho 20 22 saal ki thi

SSa: 20 22 saal ki thi

Zt chota bacha ek saal ka tha

SS acha toh aap 7 larke bata rahe hain who 7 larkon ki umar kitni hai

ZT: bara ya chota wala

SS sab se bara wala

Ztt: sab se bara wala us ki umar 22 saal hai

SS thek hai acha aur aap ne koi taleem li hai

Zt: 5 class parha hai

SS: 5 class parha hai acha aur mujhe yeh bataayein ab joh mein aap se poochne jaongi woh yehi hai joh mein ne aap ko bataya tha k joh aap k paas health worker aati hain toh us hawale se mein aap se baat karongi toh mujhe yeh bataayein k joh CHW hain health worker hain jab woh aati hain aap k paas toh aap se kia poochti hain

Zt: woh poochte hain aap k bache ko teeka laga hai hifazati joh bacha paida hota hai usi ko deakh k jaati hain jab bimaar hota hai toh is ko dawaai deity hai mein udhar jaati hun na dawaai deity thi

SS: dawaai woh yahan a k deity hain

Zt: nahi udhar bacha le jaati hun phir dawaai deity hai

SS: acha toh kia woh baqaaidgi se aati hain aap k ghar

Zt: bacha paida hota hai usi ka 3 4 din 10 15 din poochne aati thi

SS: acha 10 15 din poochne aati thi toh aise kitne maheeney mein aati thi ya 2 maheeney mein

Zt: 3 4 din arahi thi phir bimaar hota poochne aati thi

SS: poochne aati thi acha toh kia kia aap se poochti thi

Zt: poochti thi aap ka bacha sahi hai kia bimaar hai hamara bacha sahi hota hai toh mein bolti hun sahi hai jab bimaar hota hai toh mein udhar le k jaati hun phir aga khan mein dawaai achi deite hain abhi joh bacha hamara 6 saal ka hai hum nahi jaate udhar

SS: acha aap nahi jaate 6 saal ka bacha hai toh waise aap jaate thy

Zt: haan abhi bhi 2 maheena 1 maheena poochne aayin aap ka bacha kaisa hai sahi hai nahi sahi hota hai toh hum bolti hun sahi hai

SS: sahi toh kia rozana aap se poocti hain ya phir 10 15 din baad aa k

Ztt: nahi aise aati hain roz roz nahi sehat poochti hain hamara bacha sahi hota hai toh hum hispatal nahi jaati hun

SS: acha toh aap in k kaam se mutmaain hain acha mehsoos karti hain jab aap se poochti hain bache k hawale se

Zt: hum bolte hain hamara bacha sahi hai dawaai bhi leine jaati hun

Zt: haan toh sahi hai na gareeb aadmi hai unki madad karta hai dawaai deita hai sahi sab se achi baat hai hum bhi in se dawaai leine jaate hain

SS: acha toh ghar pe a k dawaai deity hain

Zt: nahi udhar deity hain

SS: udhar deity hain joh ghar par aati hain woh deity hain

Ztbacha paida hota hai toh usi ko deakhne aati hai aur poochti hain aap ka bacha kaisa hai

SS: acha toh jab sirf bacha paida hota hai tab aati hain is k elawa nahi

Ztnahi toh dawaai poochne bhi aati hai k aap ka bacha kaisa hai

SS: aap k hawale se koi sawal karti hai

Zt: nahi aise pooch k chali jaati hain

SS: jab aati hain woh kia sawal karti hain kaise apne bache k baare mein batati ho

Zt: bacha dikhao kaisa hai hifazati teeka is ko laga hai k nahi aise poochti hain

SS: aur aap k hamal se

Zt: haan hamal ka bhi poochti hai k aap ka hamal hai k nahi hai jis ka hota hai usi ka woh usi ko bolti hai

SSacha aur kia poochti hai bache k baare mein hamal k baare mein aur aap se sehat k hawale se sawal karti hai

Zthaan hifazati teeka us ka bhi poochti hai

SS: bache k hawale se teeka aur aap k liye baaki bachon k hawale se

Zt: haan hamare bache ko poochte hain kitna teeka laga hai

SS: acha aap k hawale se kia poochti hai matlab aap ki sehat se kia poochti hai

Zt: poochne aati hai aap ka kitna bacha hai woh usi mein le k jaati hai abhi bhi ek maheena hua pooch k gayi aap ka kitna bacha hai mein ne bataya hamare 2 bache ka intekaal hogaya 11 bacha hai

SS: thek hai acha aakhari dafa jab hamal se huyin thi aap toh CHW ne kuch kiya tha

Ztaga khan walon ne

SSa: hmm

Zt: nahi jinnah hispatal mein hua

SS: nahi nahi joh worker aati hain

Zthaan woh pooch k likh k gayi thi

SS: likh k gayi thi

Zt: haan

SSa: is k elawa aur kuch kiya kuch bataya ho aap ko

zt: nahi bacha deakhne aayin thi

SS: bacha deakhne aayin thi aur jab bacha hua tab kuch kiya us ne aap k liye

Ztt: deakhne aayin 3 4 din

SS: acha phir kia deakha

Zt: bache ko deakha kaisa hai is ki sehat kaisi hai sahi hai wazan kar k jaate

SS: acha sehat kaise deakhi kaise pata chala

Zt: mein ne bola sahi hai

SS: sahi hai

Zt: haan

SS kia bataya us ne aap ko

Zt: deakha ni bacha chota nikaal k deakha bukhaar us ka check kar k phir us ne bola sahi hai

SS: bukhaar check kiya aur wazan kiya aur kuch kiya us ne

Zt: nahi

SSaur kuch nahi kiya aap ne us se poocha k yeh sab kuch kyun kar rahi ho

Zt: us ne bola mein aga khan se aayi hun

SS acha aga khan se aayin hain us ne aap ko bataya toh aap ne us ko apna bacha de diya

Zt: nahi dikhaaya usi ko dikhaya saara bacha hamara goth mein hai bolti hain aati hain deakhti hain mein aga khan se aayi hun

SS: hmm hmm saare goth mein jaati hain deakhti hain acha bache ka wazan karti hai bache ka bukhaar check karti hai aur kuch karti hai is k elawa aap ko bataati hai bache k baare mein sehat k baare mein

Zt: haan bolte hain sahi hai jab bimaar hota hai toh usi ko le k aga khan jaati hun
SS: acha center k baare mein kabhi kuch bataya aap ko
Zt: hum jaati hun bacha bimaar hota hai hum le k jaati hun
SS: khud jaati hain
Zt: nahi jaati hun le k aga khan
SS acha acha
Zt: joh chota bacha hai usi ko le k jaate hain
SShealth worker gaari le k aati hai
Zt: jee
SS aap bataati hain kisi ko kis ko bataati hain
Zeenat: woh goth mein aati hai toh hum bolte hain hamara bacha bimaar hai toh le k
SS acha worker ko bataati hain
Zt: haan
SS: joh yeh ghar pe aati hain ek maheeney k baad us ko bataya
Zt: hum le k bacha paidal bhi jaati hun
SS: acha paidal chali jaati hain toh aap ko kaise pata chala k yahan aga khan ka center hai
Zt: woh aati hain bolti hain hum aga khan k hain
SS: haan woh aga khan ki hain
Zt: woh boli hum aga khan ki taraf se aayi hunt oh hum ne bola doctoraani aayeinge hamare se sawal poocheinge hum usi ko bataongi
SShmm toh yeh center hai jahan pe bache ka aap elaaj karaati hain toh woh aap ko yeh worker ne bataya
Ztt: nahi hispatal khuli thi
SS hispatal khuli thi toh kaise pata chala aap ko
Zt: aga khan aise elaaj karta hai dawaai achi deita hai
SS aap k ghar par worker ne yeh baat bataai thi ya aap ko khud se pata chala tha
Zt: hamari goth wali jaati thi
ss: goth mein jaati thi toh jab worker aati hai toh woh aap ko bataati hai k yeh center hai
Zt: bataya 3 4 hispatal badly ki phir bhi hum jaate hain hamari majboori hoti hai hum jaati hun dawaai achi deita hai bukhaar ki bhi achi dawaai deite hain

SS: acha jab bache ko check karne aati hain ya aap ko joh hai

Zt: poochne aati hai

SS poochne aati hain toh sath mein koi maloomaat bhi deity hain aap ko

Zt: sirf 2 aurat hoti hai ya 3 aati hai pooch k phir chali jaati hain

SS: matlab sirf apna hamal se ho matlab aap ko koi masla masaail toh nahi agar hamal se huye ho toh kabhi kuch bataya ho koi achi baat koi sehat k hawale se koi baat

Zt: haan bolte hain kamzor hote hain hamare aga khan chalo koi cheez deite hain khaane k liye

SS: khaane ki cheez deity hai wahan pe yeh bataati hain k center mein koi khaane ki cheez milti hai

Ztt: haan

SS acha aur is k elawa k ghar pe reh k khayal kaise rakh sakte hain

Zt: haan samjhaaya

SS samjhaati hai kia samjhaati hai

Ztt: agar aap ki sehat sahi nahi hai toh hispatal mein jao

SShmm haspatal jao aur

Zt: bas aise bolti hain

SS aur samjhaati kia hai kia bataati hai aap ko matlab mein yeh janna chahti hun na

Zt: aise bolte hain aap ki tabiat kaisi hai aap maheeney se poochne aate ho phir aap ka bacha paida hoga hamein phone kar k bulana

SS acha yeh bhi aap ko bataati hain aap ka bacha jab paida ho toh phone kar k bulana 7 maheeney tak aap ko deakhti bhi hain acha aur aur giza k hawale se dawaaiyon k hawale se

Zt: dawaai giza deite hain kamzor hote hain usi ko koi kehte hain hamare center pe aao hamare ek bache ko bhi nahi diya

SS nahi diya kamzor nahi thi kia (laugh) sahi thi acha yeh jab aati hain toh aap se baat kaise karti hain worker joh hai kis tarhan baat karti hai aap se

Zt: aate hain koi sindhi hoti hai koi mahajir hoti hai hum sindhi mein baat karte hain hamari larki bolti hai tumhare ko sindhi saaf nahi aati nahi baat karo

SS: hmm

Zt: par mein sindhi mein bhi baat karti hun

SS: acha aap sindhi hai na

Zt: haan

SS: woh aap se sindhi mein baat karti hai

Ztt: haan abhi toh bohat sindhi bolne lagi ek sakina hai aap jaante ho sakina who amma ki bahu hai

SS: acha acha… acha toh in pe aitebaar hai aap ko

Zt: haan sahi hai doctoraani pe aitebaar kyun na karein doctoraani hai thora bacha bimaar hota hai chale jaati hun dawaai milti hai

SS hmm

Zt: achi dawaai deita hai

SS achi tarhan se baat karte hain

Zt: doctoraani bhi achi hai joh aga khan mein hai ni woh achi hain

SS: kia acha lagta hai

Zt: baat achi karti hai bache ko deakhti hai koi aisi hai joh daantti hai yeh aisi nahi karti

SS aur joh worker aati hai woh kaise karti hai

Ztt: woh bhi sahi hai

SS bache ko jab woh uthaati hai deakhti hai aap ko sahi lagta hai

Zt: haan sahi lagta hai

SS: acha aap ko lagta hai is ki ek joh aap ko bata rahi hai sahi bata rahi hai is ko itni taleem hai

Ztt: sahi karte hain amal bhi karti hun is baat pea mal bhi karti hun

SS: kis baat pea mal karti hain

Zt: joh who bataati hain us ki baat hamare ko achi lagti hai

SS acha yeh mujhe bataayein us ki konsi baat achi lagi aap ko

Zt: woh samjhaati hai har waqt woh check karne bacha paida hota hai

SS: joh woh check karne aati hai toh yeh baat aap ko achi lagti hai

Zt: haan toh woh bataati hai kisi ko check karti hai k bukhaar hai k nahi hai

SS: acha jab yeh aati hai kabhi kuch deity bhi hai

Zt: hum gareeb hain hamein kia dein kia kareingi meri larki mera larka bimaar hai meri larki ka aaj operation hai aaj opration mein deina hai 11 taareekh kal nahi parsu hai inaak ka test karna hai corona ka mera bhai le k gaya meri behan ko mujhe dar lagta hai

SS: acha aap ko dar laga aap nahi gayin lekin woh yahan aati hain aap ko kuch deity nahi

Zt: nahi

SS: nahi deity thek hai maloomaat deity hain yeh mujhe batao k aap ko lagta hai k is kaam joh aati hai aap k ghar aap k bache ko deakhti hai aap ki sehat k hawale se poochti hain

Zt: pooch k chali jaati hain

SS: sirf pooch k chali jaati hai toh is ko hum acha kar sakte hain aur kis tarhan se aur acha karein hum aap ko aur faida pohchaana chahte hain is kaam se kis tarhan se pohchaayein

Zt: aap ya dawaai deingi ya koi cheez deingi toh aap ko sab boleinge k ache hain

SS: hmm kia dawaai dein ya kia cheez dein

Zthamari kuch madad karo

SS kis tarhan ki madad

Ztjoh bhi aap ko aaye hai hamari madad kar dou

SS: matlab aap ne kaha k koi dawaai dou koi cheez dou

Zt: koi achi dawaai de jao

SS: kis kisam ki dawaai aap ko zaroorat hai

Zt: hamare ko abhi zaroorat nahi hai mera bacha mashaallah bara hogaya hai yeh chote mein chota yeh wala hai aur joh hamara bara larka woh bimaar hai pata nahi gurdey mein pathri hai kia hai aise bimaar rehta hai aur hamara aadmi bhi shohar joh hamara pehle pehle jaata tha dusre ki kashti mein 2 3 saal isi ko baandha hua tha aur ab bolte hain hamara koi kaam nahi hai hamara ghar aap deakheinge hamaara ghar saamne hain mein aap ko dikhaaongi na khirki hai na darwaaza hai na hi koi cheez hai barsaat hoti hai khachhi jhomppari mein pakaati hun barsaat hoti hai hum 2 2 din roti nahi khaate hamare yahan na khirki na darwaaza ek karma bana hua hai

SS: hmm hmm hmm

Ztt: phir koi aata hai ni behan bhai toh isi ko le k ghar nahi jaati hun hamari behan k ghar amma k beithe joh baat hai

SSa: kyun amma who aap ka apna ghar hai sharmane ki zaroorat nahi aap hamein bhi wahan le k jaayeingi bithaayeingi hum beitheinge

Ztt: hamare ghar mein paani bhi peene ka isi mein rakha hua hai tum chalo mein aap ko dikhaaongi aadmi parha hua nahi hai larka bara hai woh bimaar rehta hai

SS: hmmm toh yeh mushkilein aap ne bataai mushkilaat hai toh hum worker k zariye koi madad kar sakte hain aap ki

Zt: aap ki meharbaani hogi

SS aap hamein bataayein kis tarhan

Zt: ziada zaroorat hai aap yaqeen karo hamare abbu ne pilot diya hai mere yeh aise hi para hua hai woh deakho saamne hum bolte hain hamare ko ghar bana k dein mein dua karongi

SS: yeh baat toh hai lekin kuch cheezein aisi hoti hai hum hispatal se aate hain na toh hum aap ki sehat k hawale se kaam asakein

Zt: hamaara sab se bara job wala hai na woh kisi ki madad nahi karta hamara bhai hai ni jab matric paas kiya toh bhi duty koi nahi mil rahi hai isi ka chota chota bacha hai joh goth mein aati hai nokri woh bhi apno apno ko deita hai gareeb ko koi kuch nahi poochta

SS hmm toh sehat k hawale se koi madad aap ko chaiye ya koi maloomaat aap ko chaiye toh baateyein hamein

Ztt: yeh larki bichaari hispatal gayi hai iska bhi operation ka hai abhi bhi dusre se paise 2000 le k hamara aadmi le k hispatal gaya hai joh baat hai woh hum baat kareinge hamare ghar mein paise bhi nahi tha phir 9 10 bajh gaye phir hamara aadmi bola hamari behan aayi usi k sath jaati hai ni who thora aage rehti hai toh woh paisa le k aayi phir hamari larki ko hispatal le k gaye

SS hmm acha toh worker k hawale se kuch kehna chahogi amma koi aisa kaam joh behtar banaayein aap ki sehat k liye kuch acha karne ki koshish karein kuch batana chahogi koi mashwara

Ztt: hum toh bolte hain hum se joh poochne aate hain sab sahi hai joh aap ko manzoor ho woh aap kar lo

SSjoh bhi lekin aap hamein bataoge toh woh hum kareinge na ab hamein toh nahi pata k aap ki kia zaroorat hai aap ko apni sehat k hawale se kia cheez pasand hai ya aap kia sunna chahti ho yeh toh aap hamein behtar bata sakti hai na

Zt: hum toh sahi bataate hain hum bolte hain aga khan wala aata hai toh hamara chota mashaallah bacha tha abhi hamara ek pehle bache se bare tak hum ne aga khan se dawaai li woh poochne bhi aayi thi

SS hmm hmm

Zt: toh hamare ko acha lagta hai

SS aga khan sahi lagta hai

Zt: haan

SS aur worker bhi sahi lagti hai

Zt: haan

SS aur koi cheez hai joh aap bolna chahogi

Ztnahi joh aap poocheingi mein bata dungi

SS nahi khudse koi cheez joh aap k dil ki koi baat ho kuch karna chahti ho har maheeney baad yeh worker aati hai toh yeh mujhe bata dein

Zt: woh aate hain poochne sab se sahi hai ni

SS hmm hmm

Zt: sab se sahi hai woh poochne aati hain

SS: chalo shukriya zeenat bohat bohat……..

AG-IDI-05

SS: aap ki umar kitni hai

Interviewe: 30 saal

SS: kitne bache hain aap k

Interviewe: 7 bache

SS: koi taleem aap ne haasil ki

Interviewe: nahi

SS: kitne saal se reh rahe hain

Interviewe: 4 saal se

SS: ghar k kaam k elawa koi aur kaam karti hain

Interviewe: nahi

SS: acha mujhe thora sa phir se bataayein jab woh aati hain toh kia poochti hai

Interviewe: polio k baare mein bolti hai

SSacha

Interviewe: polio pilaaya mein ne bola nahi pilaya us ne pila k chala jaati hai

SS: polio k hawale se

Interviewe: haan

SS: aur koi teekon k hawale se

Interviewer: nahi

SS: bachon k teeko k hawale se nahi

Interviewe: ek do dafa yahan pe teeka bhi lagaya ghar pe

SS a: acha ek toh teeko wali aati hai

Interviewe: teeka lagaya hai idhar kamre pe

SS: acha

Interviewe: saare bachon ko

SS: saare bachon ko teeka lagaya us ne

interviewee: haan

SS aur aap k hawale se kia poocha aap ki sehat k hawale se

Interviewe: nahi

SS: kuch nahi poocha

Interviewe: nahi

SS acha ek hi aati hain ya alag alag aati hain

Interviewe: ek teen chaar aati hain

SS: acha aap k hawale se joh sawal karti hai woh konsi hai kuch pata hai

Interviewe: mujhse toh baat nahi kari

SS: aap se kuch nahi poochti

Interviewe: kabhi kabhi poochti hai pregnant ho mein ne bola nahi

SS: hmm

Interviewe: bas

SS: aap se pregnancy ka hi poochti hai

Interviewe: haan

SS: acha… acha toh jab aap se sawal karti hai toh aap ko kaisa lagta hai

Interviewe: mein deakhti hun us ko

SS hmm deakhti hun kia deakhti ho

Interviewe: a kar beithti hai phir chala jaati hain

SS: hmm toh ab kaisa lagta hai kyun arahi hain kyun pooch rahi hain nahi sochti

Interviewe: nahi

SS a: kyun koi aap se a k pooch raha hai na k bhae aap hamal se pregnant toh nahi hai toh aap bata deity hain usay

Interviewe: kabhi kabhi bata deiti hun kabhi kabhi nahi deity

SS acha jab bata deity hain tab kia wajha hoti hai

Interviewe: woh bol rahi hai bacha kitna hai umar kitna hai aisa bolte hain

SS hmm

Interviewe: mein ne bola woh chote bache ko 4 saal bas

SS: hmm hmm aur jab pregnant ka nahi bataati toh kia wajha hoti hai kyun nahi bataati

Interviewe: woh nahi bataati

SS: kyun

Interviewe: hum ko sharam aata hai isi wajha se nahi bataate agar ho toh tab bataate hain agar nahi hota toh nahi bataate yeh baat hai

SS acha jab hoti ho toh bata deite ho

Interviewe: aur kabhi kabhi jab woh log aate hain yeh ghar pe bhi nahi hota bache hote hain th bache toh pregnant nahi hai toh woh kia bataayeingi yeh baat hoti hai

SS: acha aap aakhari dafa jab pregnant huyi thi jab hamal se huyi thi bacha paida hua jab hamal se huyi toh CHW ne koi kirdaar ada kiya tha

Interviewe: idhar toh nahi hua

SS: idhar nahi hua toh kahan hua tha

Interviewe: sacota

SS acha woh jagha kitni dur hai yahan se

Interviewe: machi mor ki taraf

SS: kahan machi goth ki taraf

Interviewe: nahi machi mor k peeche

SS: machi goth k peeche

Interviewe: machi mor k peeche

SS acha dur kitni ek ghanta lagta hai do ghante lagte hain

Interviewe: nahi 10 mint ka hai

SS: 10 mint yahan se kareeb hai

Interviewe: haan

SS a: toh worker ko bataya tha k aap pait se ho

Interviewe: nahi woh toh jinnah mein kiya hai

SS khud hi jinnah mein chali gayi

Interviewe: haan jinnah mein hua

SS: toh woh aayi thi us waqt aap k paas jab aap hamal se thi

Interviewe: nahi udhar bhi toh aayi thi magar mein ne nahi kiya

SS aap ne unhein nahi bataya

Interviewe: nahi

SS: kyun

Interviewe: ek dafa do dafa kiya

SS a: hmm

Interviewe: phir mein chori chupke beith gayi nahi bataya

SS: wajha kia thi is ki

Interviewe: bache jawan hogaye hai na is wajha se

SS: nahi toh worker se kia sharam thi joh worker thi us se kia sharam thi aap ko yeh sharam thi k bache aap k jawan hogaye hain aur aap pregnant hogayi ho yeh sharam thi

Interviewe: haan

SSacha acha yeh kitni pooraani baat hai

Interviewe: 4 saal horahe hain

SS: 4 saal hogaye hain acha acha is liye aap ne har worker se chupa liya

Interviewe: haan

SS: toh baad mein toh pata chalni thi na toh pata kaise chali aap ne kisi doctor ko nahi dikhaaya tha

Interviewe: jinnah mein

SS: jinnah mein bhi toh ja k bataya na toh jab worker aayi

Interviewe: joh kala peeliya hai na kar nahi sakti

SS: hmm

Interviewe: isi liye jinnah mein kiya

SS: kia baat hai yeh joh batana chah rahi

Interviewe: peeliya hai na

SSinko kala peeliya hai

Interviewe: haan kala peeliya hai

SS: toh doctor ne kia kaha

Interviewe: doctor ne kaha tumhari case yahan nahi hogi jinnah mein hogi

SS: acha yeh center gayin thi

Interviewe: nahi

SS: acha

Interviewe: asal mein hum log gareeb hai na joh agar scissor wagera hojaaye phir usi time pe phir paisa utha k aise karte karte hum jaate

SS: acha lekin inse poochein worker jab aayi hogi na us waqt

Interviewe: haan us time pe bhi koi pata nahi tha achanak pregnancy hogayi bacha jawan hogaya 31 saal k baad bacha horaha hai toh is ko khud nahi pata toh isko ultiyaan jab shuru hogayi phir baad mein pata chala unhein ek dafa bataya do dafa bataya unhon ne bola agar koi masla ho toh hum log ko batana tareekh yeh checkup k liye jana yeh poochta hai

SS a: acha jab bacha paida hua toh phir kuch CHW ne bataya

Interviewe: nahi asal mein bacha paida jinnah mein hogaya haan bacha toh jinnah mein hua

SS: jinnah mein hua tha phir worker aayi thi bache ko naapne k liye kuch karne

interviewe: nahi

SS a: kuch nahi aayi

Interviewe: nahi pata bhi nahi hai usko

SS: k aap ka bacha hogaya

Interviewe: haan

SS: aap ne bataya nahi usay

Interviewe: nahi bataya 2 maheena hogayi us ko aage chal k

SS a: acha toh ab woh bacha kitna bara hai

Interviewe: abhi 4 saal ka hai

SS acha 4 saal ka hai ab aati hai toh us bache ka bataya hua hai aap ne

Interviewe: haan abhi toh bataati hun us ko polia pila k jaati hai

SS: haan

Interviewe: aur kia pata nahi

SS: nahi woh joh har dusre din har 2 maheeney k baad aati hai woh larkiyaan aati hain aap k ghar pe joh polio nahi pilaati

Interviewer: 3 aati hain

SS: 3 aati hain joh polio nahi pilaati

Interviewe: baat shaat kar k phir chali jaati hain

SS: haan woh polio nahi pilaati na teeka lagaati hain aisi larkiyaan aati hain aap k ghar

Interviewe: haan 3 aati hain baat shaat kar k phir jaati hai

SS: toh aap ko kia lagta hai woh aati hai na polio pilaati hai na teeka lagaati hai kia lagta hai aap ko kyun arahi hai

Interviewer: alag alag aati hain na koi katra pilaane wala aur koi pregnant wegera ka poochne aati hain

SS: joh pregnancy poochne k liye aati hai na woh wali ki baat kar rahi hun mein toh us ko bataya tha k pregnant hun

Interviewe: nahi

SS haan kyun nahi bataya tha yeh poochna chah rahi hun mein k jab bataogi nahi toh kaise pata chalega wajha kia thi

Interviewe: nahi bola mein ne

SS sharam arahi thi aap ko ya aap ko bharosa nahi tha worker k upar

Interviewe: nahi nahi sab sahi

SS: kyun wajha kia thi khul k baat kar sakti ho koi masla nahi hai mujhe bata sakti hain aap

Interviewe: woh aati hai baat shaat kar k abhi toh ghar pe nahi aati

SS hmm

Interviewe: bahar se baat shaat kar k chala jaati hain

SS: aap log sahi tarhan se bataate hain k is ghar mein kitni shadi shuda rehte hain

Interviewe: us ka toh maloom bhi nahi hai

SS unko nahi maloom na aap logon ne bataya unhein

Interviewe: nahi

SS: woh jab aati hain toh aap chup jaati hain

Interviewe: deakh leiti hun chup jaati hun

SS a: acha toh yeh wajha aa paisa kyun karte hain jab woh aati hain toh

Interviewe: kabhi kabhi bolti hun kabhi nahi bolti

SS kyun kia hota hai joh kabhi kabhi bata deity ho

Interviewe: ammi ka ghar pe jaati hun

SS: toh jab aap ghar pe nahi hoti tab nahi bataati jab ghar par hoti ho tab bataati ho

Interviewe: haan woh toh aamne saamne par jaati hun

SS: acha warna chup jaati ho

Interviewe: mein chup nahi jaati ghar pe nahi hote

SS: acha acha acha

Interviewe: saari zimedaari un k upar hai na samandar pe shohar kaam pec hale jaata hai toh bacha hua toh un k liy soda laana waise bimaar hain

SS acha bache mein koi kirdaar ada kiya tha worker ne aayi hogi bache ko deakha sehat k hawale se bache k jab chota bacha hua tha

Interviewe: abhi toh idhar bacha nahi hua tha

SS: jab hua tha aakhari bacha joh aap ka hua chota joh 4saal ka hai jab woh hua tha toh CHW aayi thi aap k ghar us ka wazan naapne us ka kadh naapne

Interviewe: nahi

SS aisa kuch bhi nahi hua

Interviewe: pehle wahan par ammi k ghar pe ajaati thi bolti thi yeh dabba leina hai bachi ki poti karo woh bhi checkup karaane k liye aga khan le k jana yeh bolti thi hum log ko pehle kiya tha

SS: ammi ka ghar kidhar hai aap ka ali akber shah mein hi hai

Interviewe: nahi 18 number pe ali akber shah goth

A SS: toh yahan se kitna dur hai

Interviewe: 10 mint ka raasta

SS: 10 mint ka raasta hai udhar aati hai aap k ghar mein aati hai bache ko deakhne k liye

Interviewe: haan udhar jaati hai

SS: kia wajha hai k wahan jaati hai yahan nahi aati

Interviewe: nahi us k otoh maloom nahi hai

SS: kia maloom nahi hai

Interviewe: k yeh 4 saal ki hai

SS: haan toh woh us ko pata hai na 4 saal ki hai choti thi tab nahi pata tha

Interviewe: nahi abhi toh maloom par gaya hai us ko

SS a: haan acha yeh bataayein k jab woh aati hai toh koi maloomaat bhi deity hai aap logon ko

Interviewe: ek baaji hai who hamare kamre mein rehti thi woh toh bolti hai

SS parh kar deity hai joh hamari aati hai larki woh koi maloomaat deity hai kuch pattey ki baat bataati hai

Interviewe: sab kuch bataati hai us ko behan rehti thi parosi rehti thi us ko bol deity thi

SS: acha aap ko nahi bataati aap poochte nahi ho aap ko samjh mein ajaata hai aap ko yeh bhi samjh mein aata hoga na k woh kia bata rahi hai

Interviewe: haan

SS a: kia bata rahi hoti hai

Interviewe: woh bol rahi hai aga khan se aaye hain

SS: hmm

Interviewe: kuch baat karne k liye

SShaan haan

Interviewe: toh mein ne bola us ko ja k pooch

SShmm kis ko ja k poocho

Interviewe: yeh idhar toh behan bhi rehti thi parosi bhi rahi thi

SStoh woh kia bataati hai behno ko bhi bataati hai sunti hain

Interviewe: haan

SS: toh kia bata rahi hoti hai woh

Interviewe: us ne bola idhar school k baare mein bolti hai bohat saare log aati hain pata nahi

SSkia bolti hai woh joh aati hai worker

Interviewe: yeh bolte hain na who pregnant ho aga khan checkup k liye ajaaye bacha ho bache checkup karaane k liye ajaaye sab kuch hum log kareinge yani k yahan par 3 4 party aati hai is mein koi konsa hai school k baare mein bhi bolti hai har cheez k baare mein bolte hain

SSacha aur bachon ki sehat k hawale se kia bataati hai

Interviewe: koi bacha bimaar hai aga khan chali jana wahan par checkup hota hai bache ka wazan naapne k liye yani k sab kuch karne k liye woh bolte hain toh kabhi yahan se dur rasta hota hai aur rakshaw yahan pe nahi hota hai is ki wajha se ja nahi paati yeh masla hota hai

SS: acha toh joh center nahi jaate ho woh aa k batati hai center hai

Interviewe: haan

SScenter ka pata hai aga khan ka joh center hai us k baare mein pata hai jahan elaaj hota hai bachon ka chote bachon ka

Interviewe: abhi tak toh idhar aaye pata nahi hai

SSaap ko kabhi worker ne nahi bataya

Interviewe: nahi gaye udhar

SStoh yeh joh wazan naapne k liye bolti hai k wahan ajaana wazan hai karana

Interviewe: woh toh bacha ki baat us ka chota bacha hai isi liye us ko bolti hai jis ka bacha nahi hai na us ko nahi boli us ko bolte hain pregnant ho yeh number le lo aap log call karna nahi toh hum ajaayeinge yeh masla poochte hain jab pregnant nahi hoti toh woh kia bataayegi

SS: tab kuch bhi nahi bataati

Interviewe: yeh bataate hain agar pregnant hon hum logon ko bata dein koi bache ka masla hamein bata dein hum log aga khan wale kar leinge yeh bataate hain

SS: acha toh is kaam ko hum kaise behtar bana sakte hain joh joh worker aati hai

Interviewe: un ko hum acha hi samjhte hain woh hamein kitne pyar se samjhaata hai woh hamari majboori hojaata hai toh hum nahi ja sakte

SSkia majboori hai aap ki

Interviewe: jaise hamare paas kabhi paise nahi hote kabhi rakshaw k kiraya nahi hote kabhi koi masla hojaata hai is ki wajha se hum nahi jasakte

SS: yeh wajha hoti hai aap isi liye nahi jaati ho

Interviewe: haan

SSya koi aur wajha bhi hoti hai is k elawa

Interviewe: nahi

SS: paisa nahi hota toh nahi jaate ho

Interviewe: itna sahulat who hamein dein toh kaise manah kareinge

SSdur bhi hai

Interviewe: haan

SS: thora dur hai

Interviewe: yahan se dur hai na

SS: acha lekin jahan aap jaate ho who kareeb hai aur free mein hota hai

Interviewe: nahi

SStoh phir free mein toh hota hai

Interviewe: paidal nahi jasakti hun

SS: acha paidal jana hota hai rikshaw ka kiraya lagta hai hmm acha acha… acha mujhe yeh batao k aap ki taraf se koi cheez hon jaise ek dou cheez aap ne bataai k woh aap ko bata rahi hoti hai lekin aap khud se un ko nahi bataati ya toh aap ghar par nahi hoti ho thek hai ek aap ki zaban ka thora rukawat hai na k aap who urdu mein poochti hai toh shayad aap ko nahi samjh mein aati aap unhein nahi bata sakte toh is k elawa koi aur cheez hoti hai joh aap batana toh chahti ho lekin aap worker ko bata nahi paati koi rukawat aap ki taraf se hoti hai

Interviewe: bohat bolne ki koshish karti hun bache saamne ajaate hain sharam ki wajha se bata nahi sakta aur kabhi ghar pe shohar bache who hojaata hai toh bache k saamne kaise bataayein

SSa: kia cheez hai joh inko bohat inko mehsoos hoti hai sharam wali baat joh yeh nahi bata paati

Interviewe: yeh achanak pregnancy hogayi hai toh bacha saamne hai phir is ko yeh nahi bata sakti k pait mein dard hai ya koi masla hai toh aga khan ki taraf se elaaj kar sake

SS: hmm matlab ghar mein shohar hote hain ya bache hote hain

Interviewe: koi agar mehmaan wagera ajaata hai

SS: toh us ki wajha se nahi bata sakti

Interviewe: jab woh log ajaata hai tab nahi hote yeh masla hota hai

SSacha aap ne kaha k aap ya toh ghar pe nahi hoti ya phir yeh bache wagera aas paas hote hain toh bache bare hain toh sharam ki wajha se nahi bata paati acha toh kabhi aap ne us ko yeh nahi bola k mein akele mein tumse baat kar lun

Interviewe: nahi

SSaap k ghar k andar kitne kamre hain

Interviewe: 3

SS: 3 hain aur aap log total afraad kitne hain ghar mein

Interviewe: jawan beti hai jawan bahu hai 10

SS: 10 log hain thek hai total sab ko mila k

Interviewe: 7 bacha hum dono 7 8 9 10

SS: 10 log hain toh aas paas ziada log hote hain is wajha se aap sharmaati ho

Interviewe: haan

SSyeh baat bataate huye acha koi aap ko lagta hai k joh arahi hai worker joh sehat ki hai larkiyaan aati hain us se aap ko koi faida hota hai acha lagta ho ya kuch bura lagta ho

Interviewer: nahi acha lagti hai isliye gup shup maar k beith gayi thi

SS: hmm unko sunti ho

Interviewe: hm

SS: inko sunti ho

Interviewe: haan

SSacha aap ko bataati hai k yeh center hai toh aap us ko sunti ho k bhae yeh bata rahi hai toh mein is ki baat pe amal karongi

Interviewe: haan amal karti hun bol nahi sakti

SS: acha kis baat pea mal karti ho

Interviewe: nahi us ki baat toh samjh ajaati hai

SS: hmm

Interviewe: mujhe bolne nahi aati thora thora aati hai

SS:acha konsi aisi baat hai joh aap ko achi lagti ho unki

Interviewe: us k otoh insaan hai na

SShmm

Interviewe: musalmaan hai isliye achi lagti hai

SS: isliye achi lagti hai us ka baat karna acha lagta hai kia cheez achi lagti hai

Interviewe: us ka toh sab cheez achi lagti hai

SS: acha koi aur cheez batana chahogi joh hum behtar kar sake apna yeh larkiyaan aati hain inse aap ko faida aap ki hum zaban larki hogi faida hoga jis ko bangali aati hogi

Interviewe: hm

SSjis ko bangali aati hogi

Interviewe: haan us zaban ko bhi aati hai

SS: aati hai koi larki

Interviewe: haan

SS: us ko bata deity ho araam se

Interviewe: haan us ko toh bolti hun

SS: woh kaisi lagti hai joh bangali haui

Interviewe: sahi hai

SS: woh bataati kia hai

Interviewe: woh sunti hai us ko bolti hai

SS: kis ko kia bolti hai

Interviewe: behan hai aisa aisa bol rahi hai toh bolti hai us ko samjh mein aati hai

SS: us ko aap ki baat samjh deity hai

Interviewe: haan

SS: acha chalein thek hai shukriya bohat bohat…….

AG-IDI

AK: acha ambreen assalam o alaikum mera naam NAME hai ambreen mujhe yeh bataayein aap ki umar kitni hai

AN: meri umar 27 saal hai

AK: 27 saal hai acha aur aap ki shadi ko kitna time hua hai

AN: 10 saal hogaye

AK: 10 saal hogaye kitne bache hain aap k

AN: mere 3 bache hain

AK: 3 bache hain sab se chote bache ki kia umar hai

AN: asal mein woh 4 saal ka hai

AK: 4 saal ka hai acha ambreen kitna parhi huyi hain aap

AN: mein ne 7 class parha hai

AK:class parhi huyi hai acha ghar k kaam k elawa koi aur kaam karti hain

AN: nahi kuch bhi nahi

AK:aamdani k liye koi hunar wagera

AN: nahi nahi kuch nahi ghar k kaam bas

AK: sahi hai yahan rehte huye kitna time hogaya hai

AN: mujhe 10 saal hi hogaye hain yahan pe rehte huye hai na

AK:shuro se yahin reh rahe hain

AN: haan jab se shadi huyi hai yahi reh rahe hain

AK:acha teeno bache bhi aap k yahin huye hain

AN: yahin huye hain teeno bache

AK:acha sahi hai acha ambreen hum na kuch aap se apne health worker k baare mein unki kaarkardagi k baare mein kuch sawal karna chahte hain yeh chahte hain k hum sawalon se jaiza le sakein k aap log un k baare mein kia sochte hain thek hai acha mujhe aap yeh bataayeingi k yeh joh health worker hai yeh aap k ghar par kitne time k baad aati hain

AN: yeh toh maheeney mein do maheeney mein is tarhan chakkar lagaati hain

AK: acha

AN: ek dafa jaise aati hain bachon k baare mein pooch k jaati hain k kitne bache hain aur kia hai itne khaas baat toh nahi karti 2 4 baat kar k jaise a k chali jaayein

AK: hmm acha jab yeh aati hain toh woh kia kaam kia karti hain a k kia kaam sar anjaam deity hain

AN: kaam toh aisa koi bhi anjaam nahi deity toh jaise aati hai darwaaze pe poochti hain k aap k kitne bache hain aur kia aap hamal se toh nahi hai bas aur kisi k baare mein poochna hota hai toh bas pooch k chali jaati hain

AK: aur joh kisi k baare mein poochna hota hai kia poochna hota hai

AN: yeh yeh poochti hai jaise aap ka ghar koi kiraaye par hai udhar woh rehti thi idhar yeh bas is tarhan poocha hai

AK:is tarhan k sawal karti hain acha aap k hawale se kia poochti hain

AN: mere hawale se yeh poochti hain aa k kitne bache hain aap hamal se toh nahi hai bas

AK:hamal k baare mein poochti hain

AN: poochti hain

AK: aur bachon mein

AN: bachon k baare mein poochti hai

AK:aur bachon k baare mein kia poochti hain

AN: jaise kitne bache hain aap k

AK:hmm hmm

AN: jaise mein ne bola 3 bache hain aise pooch liya ya kuch poochne k liye aati hain bas

AK: kitne bache hain bas yeh poochne k liye aati hain bachon k elawa bachon ki sehat k hawale se koi sawal nahi karti

AN: nahi bachon ki sehat k elawa mujhse toh koi baat nahi karti

AK: kuch nahi poochti k

AN: nahi

AK:bacha 5 saal se chote bache kitne hain aisa bhi nahi poochti

AN: haan yeh poochti hain 5 saal se chote kitne bache hain aap hamal se toh nahi hai bas is tarhan ka poochti hain

AK:acha aur nahi poochti bachon k teekon k baare mein

AN: teekon k baare mein joh pehle wali aati hai na woh poochti hain

AK: yeh nahi poochti

AN: yeh toh nahi poochti

AK: kabhi aap se card nahi deakhti hain ya is tarhan ka kuch

AN: nahi card wagera bhi nahi deakha mere paas toh

AK:acha acha toh aap kaisa mehsoos karti hain in k kaam k hawale se

AN: in k kaam k woh bas pooch k chali jaati hain kia mehsoos karon mujhe dawaai wagera tablet kuch leini ho toh mein karongi aisa toh mein kuch bhi mehsoos nahi karti in ka kaam hi yehi hai aati hain poochti hain hamari kheir kheriat pooch k chali jaati hain

AK: aap ko lagta hai k unka kaam sirf itna hai k pooch k chali jaati hain

AN: toh mein aur kia keh sakti hun yeh bhi toh kahe koi bimaari k baare mein kisi cheez ka poochein toh mein bolon bhi

AK: toh aap kia chahti hain k woh aap se kia poochein

AN: mujhse kia poocheingi jab poochti hain toh aisa koi masla nahi hai Allah ka shukar hai koi bimaari koi woh nahi joh un k sawalaat hote hain pooch k chali jaati hain bas

AK: hmm toh aap ne kabhi nahi poocha yeh mujhse kyun pooch rahi ho aa k

AN: haan yeh mein ne poocha hai toh kehti hain k hum aga khan se aaye hain

AK:hmm

AN: toh woh pooch k likh k chali jaati hain

AK: toh jab woh aap ko keh deity hain k aga khan se aaye hain toh aap mutmaain hojaati hain

AN: mein ne bola aate honge acha hai itne gharon mein aayin hain

AK:hmm

AN: toh bas

AK: aapas mein kabhi jaise parosi ya aap log mil kar aapas mein koi baat

AN: koi us taraf nahi aati na

AK: yeh mere ek hi ghar aati hain udhar kisi ko pata nahi hai bas aati hain

AK: hmm

AN: jab woh ziada aati hain na pooch taaj karne k liye bache ka naam us chakkar mein ziada aati hain waise jab se mera beta hua hai tab se nahi aati itna pooch taaj nahi karti jab hum bolte hain tab pooch taaj karti hain un logon ko deakhti hain

AK:haan toh mein yeh janna chahti hun k

AN: un cheez mein toh bohat achi hain is tarhan poochne aati hain konsa hamal hai konsa maheena laga yeh cheezein jab poochne aati hain ab yeh 4 saal ka mera beta hogaya abhi tak toh mujhse aisa koi sawal nahi kara

AK:hmm hmm toh aur aap ko woh keh deity hain k joh hai mein aga khan se aayi hun is liye aap unko saari maloomaat joh woh pooch rahi hoti hain woh bata deity hain

AN: bas joh poochti hain woh bata deite hain

AK:toh aap ne kabhi unse yeh sawal nahi kiya k joh yeh maloomaat le rahi hain

AN: nahi yeh mein ne toh nahi poocha

AK: wajha is ki

AN: wajha nahi poochi

AK: nahi aap ne kabhi zaroorat nahi mehsoos ki yeh sawal karne ki

AN: mein ne bola nahi mein bata toh rahi hun hum ne socha aga khan se aati hongi yeh pehle bhi suna yahan par aati hain par mein bola isliye us mein aati hongi

AK:isliye aap ko aitebaar hai

AN: jee aitebaar hai k pooch taaj kar k chali jaati hain aur ziada kuch aur nahi karti

AK:acha jab aakhari dafa aap hamal se huyin thi jab chota bacha tha toh us mein CHW ne koi kirdaar ada kiya tha

AN: nahi aisa toh kuch bhi ada nahi kiya

AK:kuch nahi kiya us ne toh kahan gayin thi aap

AN: mein toh landhi mein gayi thi wahin hua tha mera beta

AK: hmm aap ne us ko bataya tha k aap hamal se ho

AN: hamal se hain bataya tha unko jab woh poochne aati thi toh unko bataya waise phir bete ki dafa woh aayin thi phir checkup waikup kar k gayin phir jab yeh hogaya tha na

AK:kia checkup kar k gayin

AN: pata nahi kaafi 3 4 doctorni aayin thi wazan check karat ha yeh kara tha is tarhan karte thy aur kuch bhi nahi

AK:aur kuch bhi nahi kiya tha acha aur koi maloomaat wagera di ho

AN: nahi maloomaat toh itna mujhe yaad nahi waise mein keh rahi hun sawal jawab nahi karti ek aat baat kari bahar darwaaze pe aur phir chali jaati hain hum aga khan se aaye hain yeh woh likh k chali gayin kitne bache hain abhi kuch hai toh nahi bas is tarhan

AK: acha aap ko lagta hai k unko matlab koi maloomaat bhi deini chaiye aap ko

AN: woh toh jab poocheingi maloomaat kareingi toh deini toh paregi na

AK: nahi unko kuch aap ko batana chaiye koi maloomaat

AN: haan mein is cheez ko sochti hun mein ne bola yeh toh kuch bataati bhi nahi hai jaise kisi tablet k baare mein aur bohat saari baatein hoti hain is mein toh kuch bhi nahi bolti

AK: kis cheez k upar aap chahti hain k aap ko maloomaat dein

AN: jaise k mein bimaar hun kisi hamal se hun kisi cheez ka woh hai toh pooch toh sakti hai na k aap ko kisi cheez ki zaroorat hai

AK: yeh cheez poochna chah rahi hun k kia cheez hai joh aap ko lagta hai k aap ko zaroorat ho us ki maloomaat

AN: jaise k hamara checkup kar k chali gayin bache k baare mein bata diya aap itne woh ho jaise is cheez k baare mein bas aur toh koi woh nahi

AK: jaise hamal se huye toh us mein kia maloomaat dein

AN: jaise maloomaat mein yeh hai k bache k baare mein pooch liya k deakhne checkup jaise karne arahi thi bache ki dharkan kaisi hai har cheez jaise is tarhan ka toh woh nahi hai aap logon ka bas aap log toh poochne aate hain chale jaate hain

AK:sahi hai aur aurton k aur bhi masaail hote hain un k sehat se related cheezein hoti hain aap chahti hain k kuch us k baare mein aap ki sehat k hawale se koi maloomaat di jaaye

AN: nahi aisa toh woh nahi

AK:nahi waise aap ko nahi lagta agar aap sehatmand bhi hain

AN: haan

AK:toh aap ko nahi lagta k unko koi sehat k hawale se koi maloomaat deini chaiye

AN: aisa toh koi bhi nahi

AK: aisa kuch nahi

AN: nahi

AK:acha yeh mujhe bataayein k joh bacha tha chota bacha hua aap ka tab aur CHW ne us ki sehat k hawale se koi kirdaar ada kiya ho

AN: kirdaar ada toh nahi kiya bas aayin thi wazan check kara unhon ne

AK: hmm

AN: aur bache ko check kara tha bache ka wazan deakha tha

AK:hmm

AN: bas

AK: acha us ne wazan check kiya

AN: jee

AK:aap ne unse poocha k bache ka kitna wazan hai

AN: haan us ne bataya tha mera bhi bataya tha jaise feed karna sikhaaya tha k kis tarhan karate hain bas yeh bataya unhon ne

AK: feed karna bataya

AN: jee

AK:kis tarhan se karwaate hain

AN: aur toh kuch bhi nahi hai bas

AK:aur deakh k bataya bache ka wazan

AN: haan wazan kitna hai pata nahi kitna bataya tha mera deakha tha is tarhan

AK:aap ne poocha unse mere bache ka wazan kitna hai

AN: haan woh keh rahi thi sahi hai mashaallah se sahi tha sehatmand

AK: hmm acha yeh joh center hai hamara aga khan ka toh is idaare se jorne mein koi CHW ka kirdaar ho

AN: nahi hai

AK: nahi hai aap ko kaise pata chala k yeh center hai is elaake mein

AN: yeh toh meri mumaani saamne rehti hai jab woh aati jaati thi tab pata chala k aga khan wali aati hain pooch taaj karti hain upar aati thi jaise yeh kiraaye pe rehte hain toh un k paas unhein bitha kar bhi le k gayin hain teeke k baare mein har cheez un ko ziada woh kara hai mere paas itna nahi aati waise kuch poochti nahi mere bachon ko toh teeke lage huye hain sab kuch itna woh nahi kara

AK: aap ne center se lagwaaye hain teeke

AN: nahi mere toh jahan bache huye hain wahin se lagwaaye

AK:kahan se

AN: humaira doctor nahi hai landhi mein udhar se bachon ka kar kara k kahin se karaya is tarhan karaya

AK: toh landhi yahan se kareeb hai

AN: jee dur hai

AK:dur hai toh teeke lagwaane aap laandhi jaati thi

AN: jee

AK: toh mushkil hoti thi

AN: nahi mushkil nahi jahan se asaani lagti hai na jin ka hath acha ho udhar se lagwaate hain

AK:acha toh aap ko nahi pata center pe bhi teeke lagaaye jaate hain aisa

AN: aisa mujhe pata tha lekin wohi hai na aane jaane mein in k papa manah karte hain

AK: kia wajha hai kyun manah karte hain

AN: bas kehte hain ghar mein hi raho kisi ko jaise na bulaao na kuch toh aap log ajaate hain aap logon ko sawal jawab ki jawab de diye hum logon ne warna manah karte hain woh apne sath hi le kar jaate hain apne sath hi le ate hain

AK:aap k husband joh hai who apne sath le k jaate hain

AN: jee le kar bhi aate hain

AK: toh woh husband ko pata hai is ka center ka

AN: aap k

AK: hmm

AN: shayad pata hoga unko pata hai

AK: matlab jaise worker aati hai toh woh kabhi bataati hai is hawale se k center hai aap k bache ko le kar aao teeke lagwaao

AN: haan yeh bolti hain yeh bola upar kiraayedaar ko na unse mujhe kaafi pata chala woh le gayin bhi apne sath gaari aati hai na aap logon ki

AK: hmm toh matlab worker ne toh bataya phir bhi aap gayin nahi center pe

AN: nahi hum ne kabhi is cheez mein woh nahi kara

AK: kyun kia worker ki baat pe aap ko aitebaar nahi hai

AN: aitebaar ki baat hi nahi hai shohar ko bataya toh us ne mujhe woh hi nahi liya woh bolte k haan yahan se lagwao suna hai bohat acha hai bachon k liye dawaai wagera bhi milti hai na har cheez ki yeh suna hai

AK: hmm hmm acha yeh mein poochna chah rahi hun k koi aisi rukaawat hai jaise aap apni baat karna chah rahi hain CHW se lekin aap kar nahi pa rahi hain

AN: nahi aisa toh koi bhi nahi

AK: koi rok tok ho

AN: bas joh hamara shohar hai na woh apni marzi se karte hain

AK: acha

AN: joh bhi karna hota hai woh apni marzi se joh un ko acha lagta hai woh karte hain ab yani k polio k katre nahi pilaate thy bilkul manah karte thy polio walon ko phir woh rangers police ko le kar aaye toh phir hum pilaane lag gaye woh kehte hain nahi in cheezon se kia hai

AK: acha acha matlab yeh hai k aap worker toh bata deity hai lekin k joh ghar wale hain unki marzi se karna parta hai

AN: unki marzi se karna parta hai jee

AK: thek acha waise koi is baat pe aap logon ka dhiyaan hota hai joh worker ghar arahi hai us ki zaban konsi hai kis us se taaluk rakhti hai yeh koi cheez aap ko lagti hai

AN: nahi mein ne toh aisa kuch nahi socha mein keh rahi hun na mujhse itni baat kari nahi un k baare mein aisa sochon ya bolon woh jab pooch k chali gayin bas un ko bata diya aap k kitni family hai aap kitne ho bas yeh karti hain ziadatar aur koi toh baat nahi karti

AK: haan lekin aap apne bachon ki information de rahi hain aap apne hamal k baare mein unhein bata rahi hain

AN: haan

AK: toh aap khud se nahi sochte k bhae konsi larki arahi hai kyun arahi hai

AN: ab yeh toh 2 4 kabhi konsi arahi hain kabhi konsi aati hain toh har tarhan ki aati hai na

AK: hmm hmm

AN: ab hamein pata hi nahi hota hum log toh khud bolte hain k aga khan wale itne aate hain kabhi kon araha hai kabhi koi kabhi koi aise aati hain

AK: haan phir kia kehte hain kabhi koi araha hai kabhi koi

AN: ab un ko hum kia bolein joh woh poochti hain toh woh bata deite hain bas ziada woh karte nahi hain phir mein woh nahi karti

AK: acha

AN: kyun k jab mujhe karwaana hi nahi hai idhar kyun k hum karwaate jahan se bache dikhaayeinge wahin hote hain wahin jana parta hai dawaai wagera le k

AK: aap parwa nahi karte

AN: jaise mein woh nahi leiti ziada notice k bhae kia karon

AK: hmm

AN: jab jaati toh koi baat karti toh leina bhi parta hai

AK: matlab interest hi nahi hai aap ne jana hi nahi hai

AN: jana hi nahi hai yeh baat hai

AK: toh yeh bari bari si baat aap ne ki lekin agar hum yeh chahte hain k aap aayein hamare center par aayein aur hamare center se a k services lein toh worker k zariye hum is baat ki kaise aap ko aitemaad dilaayein hum worker k zariye kia karein joh aap hamare center tak ajao

AN: ab center par toh mein nahi asakti mujhe itni position toh nahi k mein wahan a paaon

AK: matlab bachon ko le kar aaye

AN: bachon ko bhi le k nahi asakti

AK: toh aap k husband worker joh aap k husband se baat karein unko samjhaaye

AN: haan un ko samjhaaye toh woh shayad unki samjh mein ajaaye

AK: hmm

AN: toh yeh k woh samjh jaaye

AK: k worker joh hai woh husband

AN: ab yeh k bache bhi bimaar hote hain toh mujhe doctor ki dawaai leini parti hai

AK: hmm

AN: toh yeh hai

AK: jahan aap jaati hai elaaj karwaane k liye kia wahan par muft mein elaaj hota hai

AN: nahi wahan paison se hota hai

AK: aur joh hamara center hai aap ko pata hai k muft elaaj hota hai

AN: nahi mujhe itna nahi pata

AK: acha kia yeh kabhi is tarhan ki baat worker ne bataai ho k idaara joh hai

AN: nahi aisa toh kuch bhi nahi bataya k muft elaaj hai ya kuch hai is tarhan koi baat nahi kari

AK: hmm acha.. acha mujhe yeh batao k aap ko lagta hai k joh arahi hai in ki taaleem utni hai in mein itni qaabiliyat hai joh woh kaam kar rahi hain

AN: ab yeh mujhe itna toh nahi pata un logon k baare mein

AK: matlab aap pooch rahi hain kuch poochti hain

AN: joh poochti hain mein ne bataya na joh poocha us ka jawab de diya

AK: haan aap ko lagti hai unki qaabiliyat hai jis k hawale se woh kaam kar rahi hain

AN: haan toh hogi qaabiliyat

AK: jaise bache ko bache ka wazan naapa us ne bache ka kadh kiya toh kia woh aap ko qaabil lagi

AN: haan kaafi saari thi achi bari doctorniyaan lag rahi thi

AK: hmm.. aap ko qaabiliyat lagi thi

AN: jee

AK: jaise aitebaar hota hai

AN: haan woh hamare shohar bhi ghar mein thy na

AK: hmm

AN: toh unhon ne bola tha k jaise dikhao bache ka wazan bohat ache se check kiya hamein bhi bache ko bhi toh hamein acha laga

AK: acha.. toh jaise qaabiliyat hogayi aap ne kaha qaabiliyat hosakti hai aap k shohar ko un k upar aitebaar hojaaye toh hamara taaluk

AN: phir yeh k hamara taaluk rakh sakte hain aap se

AK: worker se taaluk hosaka hai

AN: jee

AK: acha aur koi cheez aisi joh aap hamein mashwara deina chahein k hum kis tarhan se worker ka kaam behtar bana sakte hain

AN: kia bataon kyun k mein is cheez mein pari bhi nahi aur na bole yeh masla hai kyun k mein har kaam apne shohar se pooch k karti hun

AK: hmm

AN: joh woh bolte hain woh karna parta hai

AK: acha toh aap un k baare mein sochti bhi hain k worker kyun arahi hain kaise arahihain

AN: kaise arahi hain mein ne bas bataya na aap ko bas yeh baat hai

AK: aur koi cheez kehna chahti ho koi aisi sehat k hawale se

AN: nahi aur koi kuch nahi kehna

AK: acha agar hum worker k zariye koi maloimaat deina chahein aap k hawale se ya in k kaam ko behtar bananey k hawale se toh kia cheez hogi joh aap ko pasand aayegi

AN: hamara toh yehi kehna hai k hamein acha lagta hai k yeh aati hain hamari pooch taaj karti hain bachon k baare mein karti hain is se ziada aur kia keh sakte hain

AK: aur koi cheez koi maloomaat kia dein agar dein toh jaise taise pooch k chali jaati hain

AN: pooch k chali jaati hain bas aur aisa toh kuch bhi nahi

AK: lekin woh kia bataaye aap ko kis hawale se bataaye k aap ko acha lagega

AN: acha kia sab kia acha lagega yeh bhi hai

AK: chalein shukriya bohat bohat aap ka……..

AG-IDI

SS. Apki umer

MR- umar 35 saal hai

SS: ap kay kitnay bachay hain

MR- bacha ek hai 5 saal ka

SS: Aur apki kiya taleem hai

MR- taleem 5 class

SS: ap koi kaam karti hai

MR- silaai karhaai ka kaam

SS: Acha mujhy yeh batain jab workers apkay ghar ati hain tou kiya kaam sanjam dayti hain

MR- kia poochti hain

SS: G..

SS apk baray main ya bachin k baray main pochti hain?

MR-haan yehi poochti hain kehti hai k hamare sath chalo bachon ko oxygen ki zaroorat hoti hai toh aga khan ka idara khula hai

SS: acha kiya kaam karti hain tou, woh ap kay pass kitnay arsay main ati hain?

-MR haftay ya maheeny main ati hain …. waise bhi aati rehti hain bache ka poochti hai

SS: ap kay ahwaly say kiya pochti hain?

MR pochti hain abhi hamal say tou nahi ho, ap kay kitnay bachy hain aisa pochti hain

SS: iss kay ilawa aur kuch pochti hain?

MR: na bas yehi pochtiii kitnay chotay bachy, pait say tou nahii aisa pochti hain aur bas…

SS: acha jab who ap say yeh sab pochti hain tou ap kaisa mehsoos karti hain

- MR-acha mehsoos karti hun apna khayal rakho bache ka khayal rakho aisa bolti hai hamara khayal rakhti hai dhoop ho ya barish ho hamare paas aati hain ….

SS: acha jab app akhri dafa hamal say thien tou koi workers koi kirdar ada kiya…

-MR- haan koi kirdaar ada nahi kiya unhon ne aisa kuch bhi nahi kiya kabhi nahi kaha k hamal mein ho toh kuch kiya ho kuch nahi kaha deakhne k liye aati hain keh k chali jaati hain BP high hota hai toh

hamein Jinnah k liye kehti hai k Jinnah le jao hum khud hi jaayein dhakke khaayein aati hai sirf poochti hai sirf tabiat ka poochti hai bas aur chali jaati hain kabhi yeh zehmat nahi huyi wazan gira horaha hai toh kuch bata dein checkup kar dein aisa kuch bhi nahi bolti

Interview intrupted by sister in law.. after that respondent was in hurry…give very short answers

SS: ap bachy ki nashonoma kay hawalay say koi kirdar ada kiya?

- MR-nahi kabhi yeh zehmat nahi karti wazan gira raha hai toh kuch bata dein checkup kar lein aise kuch nahi bolti chali jaati hain meri bachi 4 saal ki hai lekin lagti 3 saal ki hai lekin kuch nahi karti sirf poochti hai

SS: ap ka khiyal main worker nay ap ko sehat kay idaray say jornay main koi kirdar ada kiya?

- MR-kabhi idaare se nahi jora jab bhi kaam ka waqt hota hai toh kuch nahi karti, ati hain chali jati hain…

Started talking to her sister in law in sindhi…

SS: acha iss kay ilawa ap kuch bata chahain gi, acha mujhy yeh batain k kiya wjah hai k ap unhain apni malomat day dayti hain?

- MR-insaan par bharosa hota hai ab woh apne duty k time pe hi aati hain..

SS: ap un say sawal nahi karti k tum yeh sab malomat kiun lay rahi ho?

- MR-hum ne khudse nahi poocha woh apne duty k time par aati hai unko us ki thankwa (salary) milti hai woh wohi karti hai

SS: tou ap ko lagta hai k who yeh sab apni tankhawa k liye karti hain?

- MR-hamari jaib mein paise hote hain toh hum private le jaate hain jab nahi hote toh ghar par hi joh dawaai hoti hai woh de deite hain center par nahi jaate

SS: tou kiya workers nay apko bataya center

- MR- nahi na yeh bataya k center kab khula hota hai us ki kia timings hai kabhi nahi bataya 10 se 2 khulta hai woh bhi logon se maloom para jab hamein zaroorat hoti hai toh koi nahi aata hai dil mein sochte hain hum bache ka wazan check karein giza k baare mein bataaye khoraak kaisi dein hamein sehat k baare mein bataayein

SS: ap kay khayal main worker ko kin kin cheezon par malomaat dayni chahiye?

- MR-hamal se ho jawan hon BP ka chakkar hota hai kahan se checkup karaayein door se door jaati hain aap ko toh pata hai k kia hota hai kia hona chaiye yeh hona chaiye apni gaari le kar aaye aur hamein le kar jaayein hamara din hai toh hamein center le kar jaayein jab hum dur ja sakte hain toh kareeb kyun nahi ja sakte hain center jahan hai wahan ka maahaul acha nahi hai wahan akeli aurat nahi ja sakti hai maahwaari k hawale se hamein bataayein

SS: is kay ilawa koi aisi cheez jo ap kay worker k darmiyan rukawat hon

MR- aur rukhaawat joh hai woh nand hai thek hai nand joh hai rukawat hai jis ne interview joh hamein de rahi thi toh us ne rok diya tha woh worker ko maloomaat deine mein is liye darte hain k meri jo joh nand hai thori si woh hai ……..

SS: is kay ilawa ap koi aur baat kehna chaiye gi?

MR: nahi..

SS: acha apka bahut shukria

Date: 01122020

RA: Jee NAME aap ki umar kitni hai

CHW: meri umar 24 years

RA: aur aap ki taleem

CHW: meri matric

RA: matric hai acha aur matric kis mein kiya hai aap ne

CHW: mein ne arts mein kiya hai

RA: arts mein kiya hai

CHW: jee

RA: aur aap k kaam karne ka tajurba kitne saal ka

CHW: mujhe 3 years ho chuke hain yahan pe 4 years chal raha hai

RA: 4 years chal raha hai toh yeh kia pehle se hi kaam karte aayi thi ya surveillance mein

CHW: nahi nahi pehle mein dusre project mein thi CODI project mein phir us k baad mera yahan transfer hua because who khatam ho chuka tha toh mujhe surveillance 2 year huye hain yahan par

RA: surveillance mein 2 year huye

CHW: jee

RA: acha aur surveillance k kaam k hawale se aap mujhe batayein thora bohat k kaisa mehsoos kar rahi hain

CHW: yahan pe kaam toh pehle toh shuro shuro mein thora sa difficult laga tha kyun k mein ne kaam kiya nahi tha CODI k hawale se who thora different tha yeh thora different tha lekin ahista ahista thora bohat agaya karne mein samjh aaya lekin joh hai na community k hawale se thora sa joh hai mushkil pesh aati hai

RA: acha aur kia mushkil pesh aati hai aap ko

CHW: sab se pehle toh yeh kehte hain k aap log karte kia ho just bachon ka aap checkup karte ho ghar pe a k wazan bukhaar joh bhi karna hota hai who karte ho apne faide k liye hamare faide k liye nahi karte aur agar hum aap k center mein aate bhi hain toh phir hamein bohat ziada wait karna parta hai wait k baad jab number aata hai hum se sahi se baat nahi ki jaati toh phir hum aap se baat sahi se kaise karein jab hum aap k center jaate hain hamare sath behavior acha nahi hota aur phir aap kia deite ho

just ek calpol deite ho calpol k siwa kuch nahi deite toh bohat saare hamein counseling karni parti hai k bachon k liye ziada dawaiyan achi nahi hoti kyun k bache ka maida kamzor hota hai is wajha se who aur beemaar par sakte hain sust hosakte hain is liye hum dawai ziada nahi deite toh phir who kuch joh hote hain who maan jaate hain lekin kuch joh hote hain who kehte hain nahi bhae hamare ghar mein aana hi nahi hai aap

RA: acha toh aisa kyun kar rahe hain

CHW: who chahte hain k hum un ko kuch dein un k faide k liye

RA: jaise kia deina chaiye

CHW: jaise k who kehte hain bachon ka nahi aap k center mein baron ka bhi elaaj hona chaiye hum kahan jaayein who yeh sawal karte hain hum se toh hum unay bolte hain k hum aap ki request aage kareinge k aap k hawale se bhi kaam kiya jaaye kyun k abhi toh sirf bachon pe hain na toh thore time baad baron pe bhi kar leinge toh hum unhein tassali deite hain

RA: acha toh aap ne bataya tha k yeh bachon ka hi hai baron ka nahi hai aisa bataya

CHW: jee jee bataya hai k yeh bachon ka hi hai 0 day se 5 saal tak k bache hote hain yahan par in mein joh bhi beemari hoti hai us ka elaaj kiya jaata hai

RA: acha toh jaise aap ne kaha na k aap ne abhi kaha behavior bhi sahi nahi hota jab hum aate hain toh who kis wajha se keh rahe hain behavior sahi nahi hota

CHW: yeh sab nahi pata who joh bolte hain na toh hamare zehan mein rehti hai baat hum a k api ko batatey hain api deakhein is tarhan se unhon ne aisa bola hai ab kia kar sakte hain aur phir ghar pe kuch aur bolti hain yahan pe kuch aur bolti hain api k saamne kuch aur boleingi double minded hote hain yahan pe

RA: api kon

CHW: hamari NAME aapi

RA: acha NAME hai

CHW: Jee

RA: toh phir matlab kia kuch cheezein jab aap ne un ko barai toh phir toh aage un ki taraf se kia response mazeed a raha hai

CHW: who kehte hain k aap hamara bhi time zaaya kar rahe ho aur apna bhi time zaaya kar rahe hain

RA: toh mushkil aap ko yahan pe arahi hai

CHW: jee

RA: acha aur kia kia kehti hain

CHW: aur kehti hain k aap log kam uz kam panadol toh dein panadol tak nahi deite aap log aap log joh kaam karte hain hum se likh likh k jaate ho leikn is mein hamara koi faida nahi hai aap ka hi faida hai

RA: toh is hawale se joh hai aap surveillance k kaam k hawale se aap acha mehsoos kar rahi hain

CHW: jee jee kaam toh bilkul acha hai kyun k hamein bohat kuch seekhne ko milta hai yahan par lekin joh hai who counseling bohat achi karni parti hai toh phir who maan jaati hain kuch maan jaati hain lekin kuch toh bilkul bhi nahi maanti mau pe gate band kar deite hain

RA: aur wajha yehi hai kuch aap deite nahi ho

CHW: deite nahi hai

RA: acha deite nahi hai

CHW: jee wajha yehi hai

RA: agar deina hum chahein toh phir sahi hojaayeinge

CHW: hosakta hai sahi hojaaye in ko convince de di jaaye kyun k pehle yahan par hota tha na k convince di jaati thi jaise dusre project se le kar aate thy bachon ko vaccination karwaate thy toh un ko who sab cheezein yaad hai puraane patients ko toh who kehte hain pehle gaari aati thi ab kyun nahi aati ab aap log aate ho gaari kyun nahi aati hamein gaari mein jana hota hai hum itni dur pedal nahi ja sakte aur apne kiraya kharch bhi nahi kar sakte who yeh bolte hain k hum itni dur se aap k laal bangla aaye wahan par lekin hamein wahan pe kia milta hai ek panadol calpol is k elawa toh kuch nahi deite

RA: sahi toh phir aur joh mazeed aisa lagta ho k koi refuse kar raha ho joh aisa lag raha ho k who aane se bhi refuse kar rahe ho kyun k gaari nahi hai

CHW: hmm

RA: aisa hua hai kabhi

CHW: jee aisa hota hai who kehti hain hamein gaari dou toh phir hum aayeinge center aur gaari deinge toh takreeban ek gali ki 5 aurtein a hi jaati hain

RA: jab aap surveillance pe jaate ho toh us waqt response aap k sath kaisa hota hai yeh toh chalo aap ne bataya tha k gaari ka masla hai waise who sahi aap ko response de rahi hoti hain

CHW: han kuch joh hain who bohat acha response deity hain kuch joh hain who aati nahi hain centers baqaida vaccination poori poori yahin se karwaati hain kuch bohat acha response deity hain lekin kuch joh hai na who aana hi nahi chahte who kehte hain hum private karwa leite hain toh jab hum private karwa sakte hain toh aap k center kyun aayein

RA: acha sahi aur kia

CHW: yahan pe joh hai na thora mother ka bhi system hona chaiye na thora behtar hosakte hain refusal

RA: mother ka kaise

CHW: jaise k un ka ultrasounds IH mein hota that oh wahan pe kaafi saare patients aate hain toh yahan pe bhi a sakte hain jaise ultrasound hojaaye mother k BP check hojaaye aisa kuch ho na toh hamare refusal kaafi kam hosakte hain

RA: acha

CHW: kyun k un ko pata hoga na k hamara bhi toh elaaj horaha hai na bachon ka sirf nahi horaha

RA: hmm aur aap

CHW: choti moti sahulaat de dein toh who khush hojaayeinge waise yahan pe expect karte hain humse k kuch unhein dein

RA: sahi toh phir kaam aap ka aur ache se hone lagega

CHW: ache se milega aur phir aur refuse joh hain who hamare bilkul hi kam hojaayeinge joh hi-fi log hain who thora response kam deinge lekin joh nikhle darje k society hai who hamein response acha deingi

RA: aur joh hi-fi log hain who

CHW: who joh hain who bilkul response nahi deite

RA: acha who kis kehte hain

CHW: who kehte hain k hamare paas itna hai hum kar sakte hain toh yeh joh aap un logon mein bhi karein joh nahi kar sakte who seedha yeh bolte hain

RA: acha toh kyun aisa kehte hain who

CHW: who kehte hain hamari family mein khud doctor hain hamari mother bhi job karti hain hamare father yahin hai toh hamein is tarhan ki zaroorat nahi hai

RA: sahi thek acha aap apne household visit se hamal ya nauzaida bachon ki deakhbhaal mein kia kirdaar mehsoos karti hain jab aap gharon mein jaate ho hamal ya nauzaida bachon ki deakhbhaal k hawale se us mein jab aap kaam karte ho un ko aap batatey ho us mein aap kaisa mehsoos kar rahe ho

CHW: us mein joh hai na yani kuch bache joh hote hain naumaulood joh bache hote hain un ki care hai na mother thek se nahi karti agar who bacha leita hua hai na toh mother apne kaam kar rahi hai aur who zameen pe leita hua hai safai ka khayal nahi rakhte un ko bohat ziada counseling karni parti hai bacha aise hi beemar hota hai isi se jaraseem lagte hain toh hi bacha beemar hota hai us ki safai ka khayal rakhe us k paas rahein us ko lapeit k rakhein agar thand hai toh toh bacha mehfooz reh sakta hai aap ka yeh system joh hai bohat deakhne ko aata hai k aap ka bacha zameen pe beitha hua kheil raha hoga bahar kheil raha hoga chota sa bacha hai who aur who bahar hai leikn mother ko joh hai who sense nahi hai apne kaamo mein magan hai agar sorahi hai toh sorahi hai toh us ko bohat ziada bolna parta hai apne bache ki care karein aise toh bache beemar hote hain isi liye bemaar hote hain

RA: sahi kabhi aisa kuch hua hai matlab k aa matlab is tarhan se kehti hon maayein k aap kyun is tarhan se matlab k pooch rahi hain aisa kuch

CHW: han bolte hain k yeh sab aap kyun pooch rahe hain hum kyun bataein aap ko hum kyun bataaein aap ko pehle toh un ko introduction karwaana parta hai kuch log toh aise hote hain shakal deakh k hi pehchan jaate hain aga khan se aayi hona hamein nahi karwaana jao aap

RA: acha aisa kehti hain

CHW: jee bilkul aise hi kehte hain aga khan se aayi hona hamein nahi karwaana jao aur kuch log joh hote hain who samjhte hain k hum aman foundation se aaye hain toh phir who khushi khushi andar bulaayeinge phir bolte hain aap aman se aayi hain phir hum batatey hain hum aga khan se aaye hai na nahi nahi nahi nahi aga khan se toh hum kuch nahi karwaate aisa bolte hain nahi naam katwa diya hum ne hamare paas naam hota hai un ka lekin who kehti hain hum ne naam katwa diya unse bola tha kaat dein naam nahi kata hamara

RA: acha aisa kehti hain

CHW: aisa kehte hain

RA: toh yeh aman foundation wale kia karte thy joh k aap k sath aisa kar rahe hain aman wale aisa kia karte thy

CHW: aman wale joh hai ghar ghar ja k joh hai na BP check bhi karte thy aur joh bolte thy na k community k hamein yeh dard hai bukhaar hai toh hamein kuch chaiye toh fori un ko panadol de deite thy un ka itna bara bags hota tha un mein samaan hote thy mein ne khud bhi deakha hai na kaafi saare hain toh who un ki bohat ziada help karte thy

RA: toh koi faida un ki taraf se a raha tha aap mein nahi

CHW: hmm

RA: faida

CHW: han kyun k yahan k log expect karte hain na k kuch dein deine walon ko ziada yaad rakhte hain na toh aise hi hai

RA: sahi aur hamal k hawale se aap kia batatey ho maaon ko

CHW: maaon ko hamal k hawale se batatey hain aksar maayein care bhi karti hain apni lekin aksar maayein care nahi karti toh un ko samjhaate hain k aap joh hain khaane peene k hawale se apni diet ka bohat khayal rakha karein aur joh hum deite hain folic aur pharas deite hain yeh bhi khaya karein lekin joh hai na aurtein kuch pharas leine se manah kar deity hain

RA: kyun

CHW: who kehti hain k kaali goli hum nahi khaayeinge toh hum un ko batatey hain yeh khoon banati hain is k bohat ziada faide hain aap ko joh end time pe delivery k time pe blood ki zaroorat parti hai toh is se khaane se blood ki kami nahi hoti hai kuch maan jaati hain kuch kehti hain hamein ghabraat hoti hai bohat ziada (laugh) yeh khaane se folic toh who khushi khushi le leiti hain

RA: hmm sahi acha aap ko kia lagta hai k rozmara surveillance k kaam k elawa zachki aur nauzaida bache k hawale se maloomaat farhaam karna chaiye

CHW: jee bilkul karni chaiye

RA: kaise

CHW: kaise jaise hum deakhte hain na ghar mein bacha hai baby hai toh hamein chaiye k hum un ko bataaein k aap aayiyein hamare center aayein agar fever wagera kuch bhi hai is ko vaccinate nahi hai toh plz aap a k teeka lagwaaiye hamara yeh free ka kaam hota hai jab hum free ka bolte hain toh kehte hain acha thek hai koshish kareinge aayeinge lekin ab pata nahi k who aayeinge ya nahi

RA: acha aur kia aap ne bataya hai sehat k hawale se unhein

CHW: jee sehat k hawale se yehi k bache ko aap ziada se ziada 6 maheeney tak dood pilaayein apna 6 maheeney k baad halki phulki giza dein yani soojhi wagera kuch bhi dein leikin joh hai na maayein aksar 6 months k baad who giza nahi deity who apne type ki giza deity hain ya toh who biscuit deingi ya toh who paapey deingi is k elawa who kuch nahi deingi yani boil egg wagera kuch bhi nahi deingi

RA: acha aisa hai

CHW: toh hum un ko batatey hain k yeh sab cheezein faidemand nahi hain lekin agar aap khud se banaoge ghar mein toh yeh ziada safai bhi hai aur bacha bhi sehatmand rahega aksar maayein bolti hain k yeh khaate nahi hain yeh nahi khaata mein khilaati hun yeh nahi khaata

RA: acha

CHW: aisa bolti hain

RA: acha kis tarhan ki zachki aur bachon k sehat k wasaail unhein diye jaayein

CHW: hmm kia bataon

RA: wasaail matlab k koi information unhein maloomaat deina aur kia deini chaiye

CHW: bas yehi deini chaiye k who apna bhi khayal rakhein aur bachon ka bhi khayal rakhein saaf suthra mahaul rakhein ghar mein

RA: acha aap k khayal mein aap ko maaon aur nauzaida aur 5 saal se kam umar bachon ko maloomaat farhaam karne ki aap joh cheezein aap batatey ho unhein toh munasib training aap ko haasil hai

CHW: nahi hamein joh hain na ap baat karna chah rahi hun k hamare liye mukhtalif training honi chaiye jaise joh kuch hamein pata hai is k elawa hamein aur jaankaari honi chaiye ta k hum ziada ache se unhein bata sakein na aur who bhi joh hai hum bolte hain na who seekhti hai mother joh hai na kaafi gor se sunti hain toh zahir si baat hai hamein agar achi maloomaat hogi toh who acha samjheingi

RA: aur kis kisam ki training kitne arse mein di jaani chaiye

CHW: meheeney mein ek dafa toh honi chaiye

RA: aur kin kin cheezon pe honi chaiye

CHW: jaise k un ko bataney k lehaaz se k kia kia karna chaiye bachon ki care k baare mein apni care k baare mein family k hawale se kis se bachna chaiye kis se nahi bachna chaiye aise hi activities kuch ho un k liye k hum

RA: mukhtalif ki training aap ne kahi thi who mukhtalif aap k zehan mein kia chal raha hai

CHW: mukhtalif jaise k

RA: yeh toh aap ne care k hawale se bataya aap k khayal mein training kin cheez pe honi chaiye joh zaroori hai aap k liye

CHW: zaroori hai jaise

RA: maaon pe ya nauzaida bache hain ya 5 saal se kam hain kin kin par honi chaiye

CHW: nashonuma k hawale se honi chaiye

RA: acha nashonuma k hawale se han jee bataein kia honi chaiye nashonuma k hawale se

CHW: k un ki nashonuma behtar hosake jaise bache joh gharon mein kheilte hain na who kheilne k hawale se us k baad bhi kuch karein maaon ko koi activities bata sakein k who apne bachon par try karein un ko bataayein k aisa nahi aisa karna hai school toh who jaate hi hain lekin us k elawa joh hai na school kam aur bahar ziada hote hain

RA: acha

CHW: haan

RA: aur phir kia kehte hain aap

CHW: phir yeh kehte hain hamare bache beemaar hogaye yeh hogaya who hogaya aap log kuch dawai nahi deite phir hamare pe baat ajaati hai k aap log toh dawai nahi deite

RA: acha nashonuma k elawa aur kia kia hona chaiye aur kin kin cheezon pe honi chaiye training

CHW: laugh

RA: kyun k aap k khayal mein hamein bhi maaloom hona chaiye na kin kin cheezon pe aap chah rahe ho k training ho

CHW: hmmm is kaam k elawa joh hum kar rahe hain

RA: bhale us kaam k hawale se bhi kyun na ho

CHW: han hosakti hai

RA: chalein thek hai acha aap ko kia lagta hai k as a sahulatkaar aur nauzaida maaon k darmiyaan aap kia kirdaar ada karti hain jaise k aap un ko facilitate karte ho service ka bhi kehte ho kehte ho k aap aao toh us mein aap ka kia kirdaar tha

CHW: is mein yehi hai k hum jaate hain na ghar pe toh sab se pehle un ki kheiriat maaloom karte hain hum log k aap kaise hain k agar hum unse sweetly baat karte hain na toh who log bhi sweetly jawab deite hain hamein aisa nahi hai k hum baat kar rahe hain toh who rudely behave karein sab log aise nahi hai toh hum joh un ko baat batatey hain na toh kaafi log hum se khush bhi hote hain kaafi log acha response deite hain k aap aaiye beithiye phir bataiye kuch log aise bhi hai hum un ko batatey hain k aap hamare center aaiye toh is se aap ka hi faida hai hamara nahi hai aap ka hi faida hai aap aayeingi aap ko pata chalega toh yeh sab cheezein aap k liye asaan hongi toh who bohat acha response deite hain leikin kuch log joh hain who nahi deite response kuch log toh andar hi nahi bulaate who bahar se hi kehte hain

RA: jab aap kehte ho na k aap kehte hain k hamare paas aao center mein us waqt mother ka reaction kia tha aap k sath

CHW: kehte hain dur hai bohat bohat dur hai aur mere ghar mein joh hai na bachon ko kahin chor k nahi a sakti hum bolte hain na k aap apne bachon ko le aaiye yahan center mein hai jagha wahan khare hojaayeinge beith jaayeinge toh who kehte han nahi asal mein baat yeh nahi hai k mein le k nahi asakti asal mein baat yeh hai k mujhe convince di jaaye toh mein apne bache ko le k bhi ajaaon aur phir wapsi mein drop bhi kiya jaaye warna toh mujhe itni dur dhoop mein jana parta hai peidal phir wahan ja k beitho phir bache tang kareinge phir wapsi peidal jana parta hai toh is wajha se

RA: aur kabhi aisa hua hai k jaise mother center pe aayi ho toh koi behavior k hawale se bata rahi hon

CHW: han kaafi saare logon ne joh hai yeh kaha hai k hum se badtameezi ki jaati hai hum ne apni aankhon se toh nahi deakha kyun k hum toh field pe hote hain na toh kaafi saare logon ne bola hai tum log sahi se baat nahi karte hain na toh hum bolte hain k hum thy center pe nahi nahi aap log nahi thy aisa bolte hain

RA: kis hawale se baat kar rahe hain who kin k baare mein baat kar rahe hain

CHW: matlab aise hi in logon k baare mein bol rahe honge

RA: kin k baare mein

CHW: jaise OPD staff hai inhi se patient ka link hota hai aur kisi se nahi hota

RA: toh kia kehte hain behavior sahi nahi hai

CHW: han agar yeh log bhi sweetly hojaaye toh phir joh hai bs aur bhi refusal kam hosakte hain

RA: acha toh aap ko lagta hai k refuses is wajha se bhi rorahe hain behavior sahi nahi hai center mein aate hain

CHW: jee

RA: ya kuch aur wajha hai

CHW: yehi wajha hai aur medicine ki wajha hai kehte hain calpol k elawa aur kuch nahi deite bache ko sahi se check nahi karte ziada beemaar hota hai toh bolte hain 5 number le k jao toh phir aap logon ne center kyun khola hai jab hamara elaaj yahan nahi hosakta phir hum bolte hain yahan pe who sahulat nahi hai yahan pe machineries nahi hai aap 5 number jaoge wahan toh baraaa hospital hai wahan pe aap ko achi machiney mileingi wahan pe aap ki ziada deakhbhaal hosakegi is liye wahan refer karte hain yahan pe hamara center chota hai na toh machines wagera kam hai kuch bhi nahi hai is wajha se toh kuch samjh jaate hain kuch kehte hain nahi nahi nahi tum log joh ho bas apne faide k liye karte ho

RA: acha aur kia kia kehte hain

CHW: who kehte hain tum log joh hai who hamari wajha se paise kama rahe ho

RA: acha aisa kehte hain

CHW: haan agar hum tum logon ko information na dein toh tum logon ko paise mileinge tum logon ko tankhwa milti hai hamare ghar aate ho na tum logon ko tankhwa milti hai aisa bolte hain aur ek aurat joh hoti hai poori gali mein refuse karwa deity hai

RA: kaise

CHW: jaise ek aurat joh hai who gali mein beithi hai us k sath 3 4 aur khawateen beithi hai toh who joh hai who bolegi k hum un k ghar peg aye hain hamein nahi pata na k who bahar beithi huyi hai ghar who hai toh who knock kiya toh who kehte hain joh hai na yeh mera ghar hai yahan nahi jao yahan joh haina mere elawa koi nahi hai haanlake family hoti hai baho bhi hai beite bhi hain bachein hain lekin who batati nahi hai kuch bhi na toh phir acha next ghar mein gaye is mein bhi nahi jaayein yeh mera ghar hai acha bhae thek hai aunty aap ka ghar hai dusre peg aye yeh in ka ghar hai in k ghar mein koi bhi nahi hai shaadi shuda in k elawa aise pooriii gali ko refuse karwa deingi phir joh hai na chaaron gali mein beith k baatein karti raheingi k kuch karte toh hai nahi ajaate hain chale aate hain ek jaraha hota hai dusra aata hai ek ghar mein kitni dafa larkiyan jaati hain hum dus baar keh chuke hain hamare ghar nahi aao lekin yeh ajaati hain pata nahi kia karti hain kuch diya toh jata nahi hai in logon se aisa bolte hain

RA: toh sab ko refuse karwa deity hain

CHW: phir yeh refuse kara deity hain

RA: phir aap un ko manate ho

CHW: haan un ko bolte hain k aunty aisa nahi hai aap hamein galat samjh rahe hain aap hamare center pe visit karein aa k deakhein elaaj hota hai hamein nahi aana center wenter

RA: acha aisa keh deity hain

CHW: aisa kehti hain hamein nahi aana tumhare center wenter

RA: phir kia kehte hain aur bas yehi kehte hain hamein nahi aana center

CHW: haan

RA: aur kia kehte hain

CHW: bas isi hawale se kehti hain bachon ka karte ho baron ka kyun nahi karte baron ka karo hum mar rahe hain hamein kon deakhega aise bhi kehte hain

RA: aur yeh kab ki baat hai joh aap bata rahi hain refuse kar diya hai

CHW: yeh toh pichley circle ki baat hai

RA: yeh joh surveillance mein rehte huye

CHW: jee jee yeh ek poori gali ko refuse kara diya tha ek aunty ne phir us gali k 4 5 ghar thy unhon ne sahi data bataya kyun k who bahar nahi thy who andar hi thy apne ghar k

RA: yeh pichle circle ki baat hai aur ab

CHW: ab toh ab joh hai who gali hamari kal aayegi ab deakhein kal kia response milta hai

RA: acha mujhe bataein k aisi konsi cheezein hain joh aap k kaam k hawale se joh aap sunti hain joh aap ko acha bhi lagta hai aap ki hausla afzai horahi hai aur aap chah bhi rahi hain k jaise mere hausla afzai ho bhi rahi hai mera kaam aur ache se hona chaiye aisa kuch hua hai

CHW: jee aisa hua hai k jab hum roz daily aate hain na toh sab se pehle joh hai na hamari RA hai who bohat acha sa hamein samjhaati hai jaise kuch bhi hawale se joh hai who batati hai k bache ki care aise karni chaiye maa ki care aisi karni chaiye tum log aisa kiya karo toh kaafi cheezein joh hai who seekhne ko milti hai aur yeh bohat acha lagta hai medical field mein hai toh itna kuch pata hai toh kis wajha se pata hai aapi ne hamein bataya isliye hamein pata hai bohat acha lagta hai aur kabhi kabhi jab mother ko guide karte hain us hawale se conselling karte hain na toh khud ko bhi thora proud feel hota hai itna kuch pata hai tabhi hum dusru ko bata rahe hain

RA: toh aap ko acha lagta hai k mazeed behtar kar sakte hain

CHW: jee mazeed behtar kar sakte hain acha lagta hai hamara kaam bohat acha hai bohat kuch seekhne ko milta hai yahan par

RA: sahi acha aur kia aap surveillance k doraan aap ko koi rukawaton ka saamna karna para tha jaise aap ne bataya tha k refuses k hawale se bataya tha k poori line ko refuse kar diya us aurat ne is k elawa kuch batayein aur mazeed kuch kaam mein dushwaari aayi ho

CHW: nahi is k elawa toh koi dushwari nahi aayi leikin joh hai kabhi kabhi aisa hota hai k area joh hota hai who poora sannata hota hai waise poori row hai who corner tak ja rahi hai lambi si line lekin udhar ka area joh hai who sun saan hai kuch bhi nahi hai toh hamein thora sa dar lagta hai kyun k hamare paas tablets hote hain aur phir joh hain ek ghar mein joh hai dou hi workers jaati hain kyun k area ko deakhna parta hai na toh thora sa sath hota hai toh phir sahi lagta hai lekin joh hai yeh thora sa mushkil hota hai k poora area aisa hai kuch log aise beithe huye hain toh pareshani hoti hai

RA: koi pareshani aayi hai wahan joh aap bata rahi hain k kuch log beithe hain

CHW: ab tak toh nahi aayi waise aisa hota hai kaafi dafa

RA: kaafi dafa aur surveillance mein rehte huye hua hai

CHW: jee kuch areas aise hain na joh blocks hain na un mein factories ziada hai ek do blocks aise hain hamare jis mein factories factories hain beech mein ek ghar ja k bana hua hai toh aisa hai toh hamare paas gaari honi chaiye na k agar koi aisi situation ho toh hum gaari mein ja kar beith sakte hain

RA: aur kabhi aisa hua hai k aa aap ne koi cheez suni ho corona k hawale se kuch bol rahe hon raaste mein aisa kuch

CHW: han hum mask laga k jaate hain toh bolte hain k yeh corona wale hain le jaayeinge utha k aisa bolte hain toh hum bolte hain aga khan se aaye hain mask toh phir lagana chaiye na abhi pata hai na beemari pheili huyi hai mask toh bohat zaroori hogaya hai toh kuch log joh hai who hamein deakh k hansne lag jaate hain who hamari baat ka jawab nahi deite lekin who khud aapas mein hans rahe hote hain

RA: aur kabhi aisa k mother ne aap ko manah kiya ho is tareeke se k aap ne mask pehna hai aisa kuch

CHW: nahi nahi nahi nahi mothers toh aisa nahi kehti

RA: yeh aap ko raaste mein is tarhan se kehte hain

CHW: haan

RA: aur kia kia kehte hain

CHW: bas yeh sab

RA: aur koi mushkil aayi

CHW: nahi

RA: refuse k elawa aur yeh joh cheezein aap ne bataaein hain un k elawa

CHW: nahi in k elawa toh bas yehi hai

RA: community mein aisa kuch bhi nahi hua hai surveillance mein rehte huye

CHW: nahi nahi hamare sath toh aisa nahi hua jab se mein yahan aayi hun mere sath toh aisa nahi hua

RA: acha aap k khayal mein konsi cheezein community mein CHW k kirdaar ko behtar bananey mein madad kar sakti hai jaise aap ne kaha na k who log kehte hain k hamara kia faida aap log aate ho aisa kuch aap ne bola tha sahi toh hum aisa kia karein jin se aap ko lage k CHW k kaam mein assani hojaaye kia hum un ki madad kar sakte hain

CHW: un ko bas choti moti cheezein de dein yani panadol de dein unhein toh who bohat khush hojaayeinge

RA: acha aur

CHW: aur is k hawale se un ka BP check kar lein BP check kar lein ya koi calci wagera de dein joh un ko nuksaan bhi na pohchaaye aur un k liye sahi bhi ho aisi cheez

RA: toh is se aap ko lagta hai k CHW k kirdaar aur ache behtar hosake

CHW: who hum se mohabbat karne lageingi na yani acha response deingi hamein apne baare mein ziada achi information bata sakte hain kyun k ek lady hoti hai na who kabhi apne mood mein nahi hoti toh who hamein saara data batati toh hai lekin galat bata deity hain

RA: acha

CHW: han aisa hota hai kyun k who galat bata deity hain phir hum dobara jaate hain hum poochte hain kehti hain nahi mere toh bache itne hain nahi hum bolte hain aap ne bataya tha na bolti hain mein ne kab bataya aise keh deity hain

RA: toh yeh reason kia tha joh sise bata deity hain

CHW: un ka mood nahi hota (laugh)

RA: acha mood nahi hota

CHW: mood nahi hota unka bataney ka toh who galat bata deity hain jaise k abhi mere sath aisa hua tha k who joh hai na pregnant nahi thi lekin us ne bataya k han mein pregnant hun toh jab mein ne un se poocha k aap ka konsa month chal raha hai start hai toh who kehti hain han 8 maheena meinne kaha 8 maheena lag toh nahi raha toh phir who hasti rahegi haste haste bolegi mein Mazak kar rahi thi hum ne TAB mein enter kar liya aur who unka Mazak hogaya

RA: oh

CHW: aisa bhi hota hai

RA: phir us ka kia kiya joh aap ne inki information note ki thi

CHW: haan who bas LMP mein hota hai na toh who done nahi kiya hota unse confirmed karte hain na ultrasound maangte hain toh hum ultrasound bohat zaroori maangte hain unse ultrasound dikhaaye aap ne karwaya hoga aap dikhaayein toh jab dikhaati hain toh us se pata chal jaata hai aur jab nahi dikhaate who phat gaya ya ghoom hogaya mein ne toh phenk diya toh phir is se shak hojaata hai hai na

RA: hmm

CHW: toh thora back karna parta hai form ko back jaake phir unki sahi tarhan

RA: is mein bhi aap ko mushkil aayi jaise aap ne kaha k mushkil nahi hai

CHW: haan

RA: ab kaise nikli mushkil

CHW: yaad aayi na

RA: yaad aayi na aur kia kia cheezein joh aap ko lagta hai k deni chaiye aisi koi cheez jis se CHW k kaam aur behtar se hosake yeh k aap k sath behavior acha ho un ka aisi konsi cheez

CHW: un ko sahulat de dein gaari ki de dein gaari ki sahulat panadol calci BP bas \

RA: is se khush hojaayeinge

CHW: khush hojaayeinge bohat khush hojaayeinge kuch log toh kehte hain aap ne raashan nahi diya parchiyaan le k gaye thy raashan nahi diya aga khan waalon ne baanta hai hain aga khan walon ne raashan baanta hai kab baanta hamein toh nahi pata toh aisa bolte hain aur kuch log joh hai na jaise ilyaas goth ka naam bohat leite hain k wahan pe itna acha hai wahan ki workers aati hain hum ko le k jaati hain apne sath wahan pe delivery bhi hoti hai shuro shuro mein hamein nahi pata tha k RG mein delivery case hote hain toh unhon ne yeh bola tha jaise PW ki information leite hain na us ki saas barabar mein beithi huyi hoti hai toh bolti hai kyun naam likh rahe ho tum log karwaate toh ho nahi jab pareshani aati hai case kareeb aata hai toh tum log k yahan toh kuch hota nahi hai hamein doooor hospital mein le k bhaagna hota hai Jinnah jaana parta hai toh hum un ko bolte hain k hamare paas aisi sahulat nahi hai hamara center chota hai baat ki hai aage hojaayega toh who rehri goth ki misaal deite hain k meri behan wahan rehti hai us k saare dono bache wahin huye toh aisa bolte hain

RA: acha toh aap chahti hain k yeh cheezein un ko farhaam honi chaiye

CHW: unko farhaam ho jaise IH mein bhi hai na IH mein bhi ultrasound karwaate hain PW ka na toh yahan pe bhi ultrasound ka hojaaye ta k hum bleb hi k hamare yahan bhi ultrasound ka hota hai toh who aayeinge bhale apni convince se aaye lekin who aayeinge zaroor

RA: ok toh is se phir aap k kaam mein aur behtari ajaayegi

CHW: kaam mein behtari hogi khush bhi hojaayeinge aur phir joh aga khan ka thappa laga hua hai na (laugh) toh who bhi hat jaayega aga khan waale aate hain apne faide k liye aate hain hamara kuch nahi

karte toh who yeh bhi kaheinge nahi bhae aga khan mein joh hai aurton ka bhi hota hai ultrasound bhi hota hai bahar se karwaane jaate hain nazdeek se karwa leinge toh phir acha response deinge na

RA: koi aisi mother joh k bina faide k bhi aajaati hain

CHW: haan aati hain jaise k is area k log bina faide k aate hain

RA: acha

CHW: bachon ko le k aayeinge routine checkup bhi karwaate hain routine checkup karwaate hain bachon ko kuch nahi hota par who aate zaroor hain check up karwaane aur vaccination poooori yahin se karwaate hain kuch ache log bhi hain

RA: sahi acha aap ki raaye mein household ki sateh par hamal aur nauzaida bachon k hawale se konsi maloomaat deini chaiye aap ki raaye mein matlab jab aap jaarahe ho na aap hamal aur nauzaida bachon k hawale se aisi konsi mazeed maloomaat unhein deini chaiye

CHW: hamari haaye mein toh behtar conselling honi chaiye joh un k demaag maein hai saaf alfaazon mein un ko samjh ajaaye jab hum unko ache se bataaeinge toh copy toh who karti hain samjh bhi jaayeingi

RA: aur kuch joh aap ki raaye mein ho matlab aap ki raaye ho ya aap k paas koi khayalaat hon joh aap surveillance k kaam k baare mein aap batana chahti hon koi bhi raaye koi bhi khayalaat joh aap k zehan mein chal raha ho joh aap batana chah rahi hain aura b bata nahi pa rahin is tareeke se aap hamein bata sakti hain

CHW: is tareeke se toh kheir koi nahi hai balke joh khayalaat thy who mein ne aap ko bata diye

RA: aap k khayalaat ki baat horahi hai surveillance k kaam k baare mein

CHW: hamara kaam bohat acha hai sahi hai joh kuch us mein information hoti hai hum saamne wale se poochte hain toh samjh mein ajaata hai bas

RA: aur koi raaye joh aap deina chah rahi hon ya aap k dil mein ho batana chah rahi hon bata nahi pa rahi

CHW: nahi bas thek hai

RA: yehi hai

CHW: hmm

RA: aur kuch nahi batana

CHW: nahi bas joh hai na bachon ki joh maaon ko hai na DOB yaad nahi rehti date of birth joh hai who yaad nahi rakhti toh isliye thori si masla hota hai kyun k phir unse pooch pooch k pooch pooch k konse

maheeney mein hua tha chand ki tareekh bata dein aap toh phir hum andaza lagaate hain chand ki tareekh yeh thi toh aise baat hojaati hai

RA: yeh surveillance ka hi joh aap chah rahi hain is cheez mein asaani kar dein

CHW: assani kar dein thori si

RA: aur kuch

CHW: bas

RA: bas chalein shukriya ……

Date: 01122020

RA: acha NAME aap ki umar kitni hai

CHW: 27

RA: 27 years aur aap ki taleem

CHW: inter continue

RA: inter continue ho acha inter kis mein kar rahi hain

CHW: commerce

RA: commerce mein aur kaam karne ka tajurba kitne saal ka hai

CHW: 9 years

RA: 9 years aur surveillance mein

CHW: surveillance mein mujhe 5 years hogaye hain matlab start se hi 9 years hai pehle extension mein thi toh abhi NWSR mein aaye huye mujhe 5 years hogaye

RA: 5 years hogaye hain

CHW: jee

RA: acha ab mujhe bataaiyega surveillance k kaam k hawale se aap kaisa mehsoos karti hain

CHW: matlab kuch acha hi hai sab koi aese nahi hai matlab start jab hota hai kaam problems toh ati hai matlab kuch samjh ein nahi aata leikin yeh hai k ahista ahista sab hojaata hai matlab sab pata hai aur kuch nahi hota toh phir hum RA se rehana aapi aati hain un se discuss kar leite hain

RA: aur koi cheez joh aap acha mehsoos kar rahi hain surveillance k kaam k hawale se

CHW: jee sab hi acha hai kuch aisa nahi hai joh kharab lage sab acha hai

RA: acha aur kuch bataayein chalein aura b cheezein batayein surveillance mein aap ne kaam kiya hai us se pehle aap kia karte thy

CHW: us se pehle toh matlab jab matric kiya tha mein ne us k baad mein ne teaching ki thi phir us k baad direct hum yahin agaye thy phir yahin a kar bohat saari cheezein pata lagi hain matlab joh pregnant womens hain yeh who matlab bohat saari maloomaat joh nahi thi who yahan pe huyi hai toh yeh hai k logon ko guide karna acha lagta hai matlab batana logon ko matlab k hum kisi ki help kar rahe hain isi hawale se

RA: sahi jin ko batatey hain aap k yeh hum help kar rahe hain toh kahin aisa suna k aa kuch cheezein bol rahe hon khush bhi nahi hon

CHW: nahi refusals toh hote hi hain aur matlab abhi toh ziada refusals hain bs yeh hai k yehi bolte hain k jaise hum aap k center aate hain toh hamein medicine wagera nahi di jaati ya calpol wagera yehi de deite hain toh ziada tar log yeh kehte hain delivery honi chaiye yahan par ultrasound hona chaiye jaise hum log itni dur jaate hain 5 no. aur is tarhan se toh matlab aap k center aa k faida nahi hota matlab koi bhi beemari ho toh hum yahan calpol de deite hain ya pharas de deite hain toh who toh hum matlab medical store se bhi magwa sakte hain itni dur ja k kia faida hum itna kiraya laga k aate hain aur matlab convince deini chaiye jaise pehle tha toh matlab gaariyan leine jaati thi patient ko toh un ko yehi pata hai k gaariyan leite aati hai toh who log yeh samjhte hain jaise dusre project who apne patient ko leine jaate hain toh who kehte hain un ko leine gaye hain hamein le k nahi aaye toh un ko yeh hota hai who kehte hain k pehle leine aate thy hamein ab leine nahi aate toh phir yeh un ko batatey hain matlab ab nahi hai pehle hota tha yeh toh phir aa bhi jaate hain kuch log pehle log vaccine lagaane nahi aate thy ab jaise hum log counseling wagera karte hain who vaccine lagane bhi aate hain yahan pe vaccination karwaane k liye aur yeh hai refusals toh isi wajha se hote hain who kehte hain hamein yahan cheez nahi milti hai aur delivery who kehte hain k yahan rehri goth mein delivery hoti hai hum yani k hamare rishtedaar hai hum wahin jaayeinge karwaane k liye wahan pe sab kuch matlab medicine wagera di jaati hai toh ziadatar log wahin jaate hain

RA: toh wajha yeh hai k aap log kuch nahi deite ho aisa

CHW: who aisa who yeh bolte hain agar hamein pregnant koi milti hai toh hum us ko folic acid deite hain aur matlab end mein hum pharas deite hain toh un ko yeh bhi pata hai k hum un ko black wali goli aur yellow wali goli deite hain hum koi PW wagera matlab jaise koi capture karte hain toh phir who direct hamein yehi bolti hai koi faida nahi hai yahan pe dikhaane ka aap hamein kaali wali aur peeli wali goli hi deingi

RA: goliyaan na dein

CHW: goliyaan hi deingi aap hamein toh matlab ultrasound wagera kuch nahi karwaate aap log kia faida goli deine ka matlab toh phir hum un ko batatey hain is tareeke se yeh faide hain is se faida aap lein bohat se log matlab samjhte hain toh phir matlab le toh sabhi leite hain lekin joh khaate hain who matlab kuch log hi hote hain joh khaate hain jaise proper jaise un ko bol k jaate hain aap is ko khaaiyega itni dafa khaaiyega yeh khaaiyega is k faide hain bohat kam log khaate hain warna toh kuch log aise hote hain hum se le leite hain aur who na matlab k un k bache kheil rahe hote hain koi udhar pheink rahan hota hai toh hum un ko bolte hain nahi sunte refuse karte hain is tarhan se

RA: sahi toh wajha kia thi yehi k nahi de rahe hain kuch kar nahi rahe hain

CHW: kuch kar nahi rahe hain who yeh kehte hain delivery bhi honi chaiye yahan par

RA: acha

CHW: haan matlab main joh hota hai who delivery hi hoti hai baaki jaise agar hum un ko batatey hain aap folic acid khaayein yeh de k jaate hain who yeh kehte hain yeh toh medical store se manga k kha leite hain ya hamare kareebi doctors hain who bhi hamein deite hain ya aksar log daayon k paas jaate hain daayaan bhi un ko likh k de deity hain yeh cheezein

RA: sahi aur kia kehte hain

CHW: aur yehi bas

RA: bas yehi kehte hain

CHW: hmm

RA: toh matlab k jab aap jaate hain toh phir kabhi aisa k aaye ho kyun darwaaza band karna

CHW: hmm jaise joh ziada refusals hote hain jaise matlab baat hi kar rahe hote hain toh matlab bahar nikaal k foran darwaaza band ka deite hain

RA: toh yeh keh k aap

CHW: matlab hum jaise matlab jaate hain pehle salam karte hain un ko apna ntroduction karwaate hain k hum aga khan se aaye hain kuch log aise hote hain hum bas yehi bolte hain hum aga khan ki taraf se aaye hain aap se kuch information leini hai toh bas yeh sun k foran darwaaza band kuch sunte hi nahi hai matlab kuch log bohat ache bhi hote hain matlab ache se baat karte hain jab un ko batatey hain k aga khan ki taraf se aaye hain aur har cheez batatey hain jaise NIC number leite hain contact number har cheez batatey hain patient card la k deite hain kuch log bohat ache bhi hote hain aur kuch log aise bhi hote hain joh aga khan ka naam sunte hi who foran darwaaza band kar deite hain

RA: sahi toh yeh kia aap ko koi task diya gaya tha k aap ko in jaghaaon pe jaana hai yahan pe refisals ziada nazar aaye hain ya kahin aur jagha pe bhi joh aap ko kaam diya gaya ho

CHW: nahi refusals toh har area mein hai matlab aisa nahi hai k kisi mein bilkul bhi nahi hai lekin kuch blocks hain us mein bohat ziada hai aur matlab yeh kuch aise hain matlab masjid k imam wagera kuch hote hain who yeh sab cheezein nahi maante toh who yeh kehte hain k hum aga khan se elaaj nahi karwaayeinge kuch log aise hote hain hamara panel hai toh hum wahan se elaaj karwaayeinge aur kuch logon ko elaaj hi acha nahi lagta hai waise toh who is wajha se elaaj nahi karwaate k matlab kuch nahi diya jaata aur phir kuch log aise hote hain who yeh kehte hain hum center aate hain sahi baat nahi hoti hai toh phir hum is wajha se nai aate

RA: toh center mein kia kehte hain k sahi baat nahi hoti

CHW: hum log jaate hain na sahi baat nahi hoti hum se k hum log matlab kuch bata rahe hote hain toh nahi poocha jaata yeh who toh is tarhan se toh yani k hum ne mam se bhi bola tha yani k mam ne bhi baat ki thi sab se k aap log sahi se baat karein yeh who toh ab aise issue nahi aate

RA: yeh kin se baat ki thi waise

CHW: matlab wohi OPD wali larkiyon se joh OPD mein staff hai un se matlab toh matlab aisa nahi hai kyun k matlab patient aate hain hum log un k ghar pe jaate hain toh who log hamein bhi sahi nahi batatey card wagera jaise hum log deite hain yahan se center e diya jaata hai toh who bhi ghuma kar ajaate hain un ko date of birth bhi yaad nahi rehti aur kuch bhi yaad nahi hota toh phir un se ugalwaana parta hai jaise hum log bhi jaate hain toh abhi kabhi date of birth bhi galat hojaati hai toh un logon ko yaad bhi nahi hota toh un ko aise yaad dilaana parta hai k bakra eid pe hua tha ya ramzan mein hua tha yani k beech mein hua tha is tareeke se phir unhein yaad dilaana parta hai

RA: acha toh matlab yehi hai na k aa aap se isliye bhi sahi se baat nahi kar rahe hain k center pe nahi matlab k sahi se baat nahi karte

CHW: pehle a rahe thy aise issue abhi toh aise nahi hai

RA: aur yeh surveillance mein rehte huye hua hai aisa kuch

CHW: nahi abhi tak toh nahi hua kssfi time pehle ki baat hai yeh

RA: kitne time pehle ki baat hai

CHW: hmmm last year

RA: last year ki baat hai . acha tabhi yeh response aap ko sahi nahi deity kyun k yahan pe bhi masle aaye thy

CHW: nahi ziadatar log aise nahi bolte kuch log aise hote hain k log bolte hain hum yahan center gaye thy toh humse sahi tareeke se baat nahi huyi is wajha se hum aap k center nahi jaayeinge

RA: lekin aap ne poocha kis ne aisa kiya hai

CHW: jee hum ne poocha tha un ko naam wagera nahi pata hota k matlab kon hai kon nahi hai bas yeh pata hai hum center gaye thy wahan pe na unhon ne sahi baat nahi kari thi toh is wajha se toh phir hum poochte hain kis wajha se aksar jaise unhein bola jaata hai vaccination lagwaane k liye aap is din aaiyega who nahi aate time pe jaise jab time nikal jaata hai tab aate hain toh phir un ko bataya toh aisa lagta hai k matlab hamein daant rahi hain

RA: acha

CHW: is tarhan se bhi hota hai toh phir hum un ko samjhaate hain poochte hain kis wajha se aap ko bola tha kis liye toh phir hum un ko bataya nahi bhi aisa lagta hai hum bhi un se galat baat kar rahe hain toh who aisa samjhti hai

RA: sahi acha aur kia samjhti hain

CHW: matlab isi hawale se aur yehi k matlab kuch bhi yaad nahi hota yahan pe matlab agar ultrasound bhi log karwaate hain toh jaise who ultrasound karwaaya un ko doctor ne bataya aap ko itne hafte chal rahe hain aur matlab maheeney yeh chal rahe hain toh who wahin chor k ajaati hain ultrasound ki report bohat saari aurtein ziadatar ali akber shah mein pehle juma goth mein bhi hota tha lekin ab yeh hai k

itna nahi hota logon mein thori awareness agayi hai lekin yeh hai ali akber shah mein bohat kam log hain joh samjhte hain baaki ziadatar log matlab 4 month bhi hote hain un ko nahi pata hota un ko pregnancy hai toh bohat mushkil se matlab andaza laga k unse poochna parta hai k aap pregnant hai toh matlab lag raha hai hamein kehti hain tumhein kaise lag raha hai mein toh nahi hun yeh who phir hum kehte hain bata dein bohat ziada matlab who kar k karna parta hai toh unse poochna parta hai kabhi kabhi LMP ki mistake hojaati hai matlab kuch pata hi nahi hota

RA: hmm sahi acha aap apne household visit se hamal ya nauzaida bachon ki deakhbhaal mein kia kirdaar mehsoos karti hain jab aap jaate ho hamal ya nauzaida bachon ki joh aap deakhbhaal k hawale se batatey ho us mein aap ka kia kirdaar tha

CHW: matlab jaise unhein sehat k hawale se batana matlab unhein batana health k hawale se bache k maa ko yeh pooch rahi hain aap yeh hai k jaise matlab k newborn hota hai toh hum unhein ziadatar yehi batatey hain 6 month tak aap apna hi feed karwaaiyega ya phir sab se pehle baby aap ka jab paida ho toh foran BCG lagwaaiyega pehle matlab log apna feed nahi karwaate thy aur sab se pehle joh matlab peela joh dood aata hai who zaroor pilaaiyega toh pehle jab yahan pe start hua tha NWSR start hua tha pehle ANISA tha toh matlab ziadatar isi pe conselling karte thy k matlab jaldi jaise matlab sab se pehle maa apna hi dood pilaaye feeder na dein toh matlab yani us k baad se log matlab ziadatar apna hi dood pilaate hain bohat kam log aise hote hain jinhein nahi araha hota toh matlab who feeder wagera deite hain baaki log apna dood pilaate hain is k elawa jaise pehle tha hum log vaccination ka poochte thy toh log nahi lagaate thy leikin ab hum jaise card wagera deite hain ja k enter karte hain ab ki dafa bohat ziada cards mile hain vaccination k aur lagaane bhi aate hain log cener hamare pehle nahi aate thy abhi bhi khud bolti hain pehle nahi aate thy lekin ab ki dafa bohat ziada aate hain mashaAllah se

RA: joh pehle nahi aate thy joh aap ne bataya tha wajha wohi thi

CHW: nahi nahi who nahi matlab pehle log matlab yahan par tha yeh 7 block mein pehle lagte thy teeke wagera center that oh log wahan chale jaate thy 5 number chale jaate thy kuch log aise hote thy matlab yahan nahi arahe k kahin pe kisi ne manah kar diya wahan nahi jana toh un ki baton mein a k matlab nahi aate thy is tarhan se

RA: sahi aur rozmara surveillance k kaam k elaawa zachki aur nauzaida bache k hawale se maloomaat farhaam karna chaiye

CHW: jee

RA: kaise

CHW: matlab hum yehi bolte hain jaise aap ki pregnancy hai matlab jaise yeh cheezein khaayein yani k paani ziada peeyein dood peeyein aur daalein joh aap ka dil kare who khaayein jab jaise delivery aati hai jaise hone wali ho toh aap yani k kisi gaari wagera ka intezaam kar lein aur yeh hai k doctor pehle naam likhawa lein dusri aap ko 5 month aap ko jahan pe kareeb lag raha hai aap wahan ja k apna naam likhwa lein toh yeh sab cheezein hum unhein batatey hain aur is k elawa hum unhein medicine deite hain folic acid deite hain start k month mein aur phir jab yani k hum dobara jaate hain pehle yeh har month visit

hote thy abhi jaise k call pe pooch rahi hoti hain toh phir hum dobara jaate jab unhein 5 months ya 6 months chal raha hota hai pharas deite thy toh kuch log hote hain joh kha leite hain kuch log nahi khaate who log pheink deite hain ya phi raise hi hum dobara ja k pooch leite hain hum aap ko de k gaye thy aap ne khaai toh phi raise hi rakhi hoti hai un k paas

RA: acha

CHW: hmm

RA: aur kia kehte hain

CHW: aur phir who kehti hain k hum nahi kha rahe k matlab kuch faida nahi hai is ka phir hum un ko batatey hain kuch log samjh jaate hain kuch log nahi samjhte

RA: aur hamein kia unhein batana chaiye sehat se mutallik nauzaida bachon k hawale se aur kia maloomaat unhein farhaam karna chaiye

CHW: aur yeh k khaane peene k hawale se un ko matlab yeh hai k apne aap ko khush rakhein jab aap relax hongi phir yeh hai k aap ki health bhi achi hogi k tension wagera ziada na lein apne aap ko khush rakhein toh is tarhan se aur jab bacha paida ho toh matlab us ko saaf suthra rakhein apne aap ko bhi saaf suthra rakhein apne ghar ko bhi saaf suthra rakhein aur us ki vaccination bhi complete karwaayein 6 month tak apna hi feed karwaayein us k elawa 6 month k baad us ko thori thori giza dein thek hai aur yeh hai k saaf suthra rakhein us ko

RA: acha yeh joh joh cheezein aap un ko maloomaat de rahe ho thek hai nauzaida bache hain maaon ko 5 saal se kam umar k bachon ko joh aap maloomaat de rahe ho toh aap ko koi munasib training haasil hai

CHW: yeh matlab jab ANISA than a toh tab hamein antenatal or post natal k baare mein bataya gaya tha tabhi k matlab is tarhan se yeh cheezein deini chaiye aur is tarhan se feed wagera pehle hamein is cheez k baare mein nahi pata tha jab hum aaye tabhi in cheezon k baare mein pata laga hai aur phir hamein kuch nahi pata hoti toh hum mam se ja k pooch leite hain aur waise yeh hamein training di gayi thi jab hum yahan aaye thy NWSR mein ANISA mein toh tab hamein bataya gaya tha matlab k 6 month tak bache ko ma aka dood pilaayein aur yeh saari cheezein kia kia khana chaiye aur matlab naam likhwaayein sab matlab bataya gaya that oh phir hum follow karte arahe hain wohi sab bata rahe hain aur kuch log aise hote hain who matlab k bolte hain haan hamein bataya tha pehle hamein pata hai mera pehla baby hua that oh hamein bataya tha k is tarhan se karna chaiye toh hum waise hi kar rahe hain aur kuch log aise hote hain who un ko baar baar bhi batatey hain who nahi follow karte

RA: aap chahte hain aur mazeed training hamein milni chaiye

CHW: jee matlab yeh hai k hum chahte hain hamari knowledge mein izaafa ho aur matlab acha lagta hai kuch cheezein matlab hum seekhein aur mashaAllah se matlab hum aisa sochte hain k hamein matlab bohat ziada seekhne ko mila hai yahan se matlab jitney projects aaye hain matlab jaise aate hain matlab yeh k TAB wagera yeh who toh matlab bohat saari cheezein seekhne ko mili hai aur vaccination k baare

mein hamein itna nahi pata tha ab jaise hamare ghar k log hain jaise hum unhein bhi guide karte hain vaccination lagaayein ROTA vaccine lagaayein yeh who toh pehle toh log ROTA wagera nahi lagwaate thy lekin ab yeh hai k lagaate hain toh matlab hamare ghar k log hamari gali k log bhi khud lagaane jaate hain

RA: aur kin kin cheezon par training di jaani chaiye

CHW: matlab yeh waise bhi course wagera hoti hai jaise ultrasound mein yeh hai k hamein jaise poocha tha mam se ja k toh mam ne hamein bataya tha kis tareeke se weeks nikaalte hain weeks joh nikaalte hain LMP se ya weeks likhe huye hote hain LMP nikaalte hain who hamein bataya gaya tha hamein nahi pata tha jaise log hote hain who hamein batatey hain k yeh report aap parh k bata dein toh hum bolte hain hamein nahi pata aap hamare center aaye wahan pe hamari doctor hain aap matlab unse poochein matlab us mein se kuch cheezein hoti hain jaise main main hamein training deini chaiye matlab koi humse poochein toh hum unhein bata sakein

RA: sahi acha aap ko kia lagta hai k asa a sahulatkaar aur nauzaida maaon k darmiyaan aap ka kia kirdaar tha jaise k aap ne kaha na aap un ko facilitate karte ho service bhi provide kar k deite ho community mein ja kar matlab un ko cheezein batatey ho aap aayein bache ko le aayein toh yahan sahulatkaar aur maaon k darmiyaan aap ka kirdaar kia tha aap ka kaam kia tha

CHW: yehi k hum unko matlab bataya k un ko kia cheez karni chaiye kia nahi awareness un ko matlab di toh yeh hai k follow karti hain aur hum jab next time jaate hain toh yeh sab cheezein follow kar rahi hoti hain vaccination lagaai huyi hoti hai toh phir hamein acha lagta hai k hum ne unhein bataya toh unhon ne saari cheezein follow ki aur matlab kuch log lagwa bhi rahe hote hain poori vaccination complete karwaai hoti hai 6 months tak jaise hum un ko bata rahe hote hain feed karwaayein apne bache ko saaf suthra rakhein yeh who sab who waise kar rahe rote hain aur kuch log pehle se ache hote hain matlab sab cheezein unhein pata hoti hain jaise kuch blocks hain 27 block 36 block is tarhan k blocks hain us mein logon ko pehle se pata hota hai toh un ko matlab ziada who bolte hain hamein sab pata hai k hamein ja k lagwana hai toh khud hi hamare center a k lagwaate hain aur kareeb k block hain who bhi saare aate hain joh dur k hain who yehi bolte hain hum is wajha se nahi arahe kyun k koi gaari wagera nahi hai aur jab hum jaate hain hamein aise deakhte hain sab cheezein follow kar rahe hain hum ne un ko bataya hai hum ne un ko toh acha lagta hai

RA: acha jab aap un ko facilitate kar rahe hote ho un ko bata rahe hote ho aap aayein joh koi aa bhi jaati hain koi gaari ki wajha se nahi a paati

CHW: jaise hum jaise new born hota hai hamein koi PW milta hai hum un ko batatey hain k Thursday wale din BCG lagega aap wahan ja k lagwa lijiyega toh ziadatar log ab lagwa leite hain bohat saare log aa jaate hain lagwane k liye kabhi kabhi aisa bhi horaha hota hai k 20 bhi arahe hote hain maaein lagwaane k liye kabhi 18 ab toh bohat ziada a rahi hoti hain lagwane k liye api batati hain hamein aisa malika aapi k bohat ziada aati hain aur kuch log aise hote hain k nahi hum 5 number se lagwa leinge bohat kam yani k ab aisa nahi hota hai k hum bolte hain k 5 number se lagwa leinge aksar lashaari k log hote hain FM k log hote hain un ko yeh ziada dur parta hai toh phir who Ibrahim hyderi lagane chale jaate hain ya phir

yahan pe indus chale jaate hain lagaane k liye yeh hai joh kareeb k log hai na jaise 27 block 11 13 yeh who bohat ziada matlab lagwaane k liye ajaate hain

RA: sahi

CHW: bas convince ka bolte hain k hum jaise teeke lagwaane jaayeinge jaise delivery hai kisi ki toh sawa maheeney tak bahar jaise yeh hota hai na nahi nikalte toh kisi ki saas wagera hoti hai who le k a jaati hain baby ko toh who yehi kehti hain agar gaari ho toh hum le k chale gaye warna hamein problem hoti hai toh nahi lagwaate toh isi wajha se delay hojaata hai teeka toh un ka bacha jab 5 months ka hota hai 6 months ka horaha hota hai toh who lagwaane ajaati hain kyun k who kehti hain hamein masla hota hai jaane mein hamare husband bhi gaye huye hote hain aur jab who aate hain kaam mein se toh yeh hai k aap ka center band hojaata hai

RA: aur aisi konsi cheezein hain joh aap k kaam k hawale se joh aap sunti hain joh aap ko acha lagta hai aap ki hausla afzaai horahi hai aur aap chahte hain k yahan kaam aur mazeed ache se hona chaiye aisa kuch joh aap apne hawale se sunte ho acha hausla afzaai bhi aap ki horahi hai aisi koi cheez joh aap chahte ho

CHW: kuch log hain joh matlab bolte hain k hum aap k center gaye thy wahan pe matlab hum yani k hamara baby sahi hogaya hai hamara baby bhi tha hum ne wahan ja k mam se matlab un ko check karwaaya unhon ne hamein guide kiya k k is is tareeke se matlab sardiyan hain aap us ko matlab aksar log yahan pe bachon ko kapre nahi pehnaate aise hi nangey ghoom rahe hote hain toh mam un ko batati hain guide wagera karti hain wahan gaye thy toh medicine yehi calpol wagera di thi aur medicine bhi likh k di thi ya gharelo matlab bataya tha hum ne follow kiya tha hamara bacha acha hogaya hai aur yeh k hum aur ko bhi batatey hain k wahan jaayein center bohat se log aise bhi milte hain bohat ache se aur phir hamein bithatey hain bohat ache se matlab baat karte hain aksar aisa hota hai k who apne matlab kisi aur family matlab member ko bula leite hain k yeh matlab pregnant hai aap un ka bhi zaroor naam likhein toh matlab lekin kabhi aisa hota hai hamare matlab out of DSS hoti hain toh phir hum log nahi likh paate toh phir hum un ko manah kar deite hain lekin hum unhein yeh bolte hain aap center chale jaayein agar aap ko titanus k teeke lagwaane hain wahan lag jaayeinge

RA: yeh a surveillance k kaam ki doraan ki baat horahi hai us mein aap joh mehsoos kar rahi ho who kaisa mehsoos kar rahi hain aap acha mehsoos kar rahi hain aap ki hausla afzaai horahi hai surveillance k kaam k hawale se bata rahi hun

CHW: jee ho toh rahi hai

RA: k aap ko acha lag raha hai

CHW: jee haan bilkul acha lag raha hai matlab acha lagta hai jab hum kisi ko guide kar rahe hote hain ya koi bata raha hotahai hamein is se faida horaha hota hai toh acha toh lagta hai aur jab koi refuse karta hai toh (laughing) jab koi refuse kar raha hota hai toh mau pe darwaza band kar deite hain toh phir matlab acha nahi lagta toh matlab jab bolte hain toh jaise hum itna yahan k logon pe mood pe baat hai kuch log aise hote hain abhi toh hamein bohat acha bataya bohat ache se baat ki hai aur phir next time

hum gaye hain aur phir wohi log foran se darwaaza band kar deite hain un ka dil nahi kar raha hota baat karne ka phir hum subha subha jarahe hote hain phir who knock kar k neendon se uth k aate hain toh un ka mood nahi hota toh who darwaza band kar deite hain foran se

RA: toh yeh surveillance k doraan hua

CHW: jee jee toh phir yeh hai k phir acha nahi lagta

RA: yeh toh aap ki ek surveillance kaam k thora joh aap bata rahi hain yeh aap ki rukawatein hain pareshania hain yehi wohi cheez hain joh aap bata rahi hain koi aisi rukawatein bataaeyein refuses aap ne bata diye thek hai aur aisa joh surveillance k kaam k doraan aap ko laga ho un rukawaton ka saamna karna para ho community mein

CHW: nahi is k elawa toh kuch aisa nahi hai matlab sab sahi hai

RA: kuch aisa community mein aap jarahe hote ho koi aisa waqiyan bayan kaein joh hua ho community k doraan

CHW: yeh hai matlab surveillance jaise market mein kar rahe hote hain phir kahin DSS kar rahe hote hain ab jaise last round mein hum log mein aur meri partner hum log 14 block mein hum log field kar rahe thy toh hum log wahan pe kaafi saare larke khare thy toh who log drugs wagera le k khare huye thy toh un k paas gun bhi thi toh hum logon ko nahi pata tha k un k paas gun hai toh hum log field kar rahe thy toh phir ek aunty hai toh matlab un ki hum ne na information le li thi toh phir who un ko deakh rahithi toh phir unhon ne hamein bulaya ishaara kar k toh phir mein un k paas gayi toh bataya k in k paas gun hai yeh log aap logon ko deakh rahe hain aur aap logon k paas tablets wagera bhi hai toh unhon ne bola aisa karo k abi nahi aap log jao larkiyan hon yahan pe kaafi sannaata laga rehta hai aur yeh area sahi nahi hai toh aap log thori deir yahan beith jao toh phir hum log wahan thori deir beith gaye thy jab who log chale gaye hum ne kaam start kar diya

RA: matlab aap ko yeh tha k

CHW: koi nuqsaan na pohchaaye tablets na le lein humse is liye toh phir hum ne api ko bataya that oh phir next day api hamare sath gayi thi toh phir hum logon ne sath field kari thi toh phir jab tak hum ne block kiya tha tab api thi hamare sath

RA: acha aur koi jaise koi corona ki wajha se bol rahe hon

CHW: haan who yeh kehte hain k matlab joh corona wagera nahi hai hum log matlab aap log wahan se aaye ho koi information nahi deini aap hamara test karne k liye ajaoge yeh who toh phir hum unhein batatey hain is wajha se nahi hum joh pregnant womens hoti hain is wajha se hum log aaye hain toh phir kehte hain nahi hamein hospital k hawale se kuch nahi karwaana aur jaise bohat kam 2 ya 3 honge aur koi aisa nahi mila phir 2 ya 3 log matlab ghar aise nahi hai jinhon ne hamein aise bola ho abhi tak jab se corona aaya yani k june mein july mein kaam start hua tha tab se le kar ab tak toh phir matlab k who keh rahe thy hamein nahi karwana who pehle se bhi refuse thy joh family jinhon ne hamein bola kaafi saalon

se refuse hain aur jaise who log yehi hai jaise hota hai na imam wagera hote hain masjid k who nahi karwaate

RA: kabhi aisa hua hai refusals walon ne kaha ho k hamara aap ne naam kyun rakha hai aisa kuch

CHW: haan hote hain kuch log who bolte hain k hum ne toh naam likhwaya nahi tha aap k paas hamara naam kaise agaya aur phir hum log unhein batatey hain last time jab hum aaye thy aap yahan beithi huyi thi phir ghar mein jab jaate hain yaad ajaata hai hum ne in se kaise information li thi kaise nahi li thi toh phir unhein hum yaad dilate hain phir unhein yaad ajaata hai toh keh rahi thi k ab hamara naam kaat dou hamein nahi likhwana

RA: phir aap ne kia kaha

CHW: toh phir hum ne kaha k naam toh nahi kat sakta yeh hai k aap k bhalaai k liye hai k agar kabhi aap pregnant huyi ya aap k chote bache hain yahan a k elaaj karwaaye abhi toh nahi hain jab honge tab likh leina hamara naam

RA: acha aisa kehti hain

CHW: haan jab hamare bache honge jab pregnant honge tab aap k center a k bata deinge hamara naam likh leina aur kuch log aise bhi hote hain unhein pata hota hai hamare paas saari information hai toh who bolti hain k matlab hum jab naam likhwaayeinge pehle bare bache ka naam likha tha na 4 saal pehle us ki date of birth laga dou hamein (laugh) toh who kehti hain who hota hai na 5 years se bara hojaata hai toh hat jata hai toh maa kehti hai nahi hum ne likhwaya tha aap humse saari information le k gayi thi aur gift bhi diya tha chaadar di thi aur doctor aayin thi checkup karne k liye aur matlab k information hai saari do birth certificate banwa dein toh kuch cheezeon pe bol rahi hoti k nahi jab tak la kar nahi dougi na hum ne kaha aap hamare center jaayein aap matlab wahan pe unse bolein aap ko nikaal k de deinge api k paas hoti hai information saari BIA joh hain un k paas who nikaal k de sakti hain toh phir hum un ko bolte hain k aapi ya phir kabhi dobara hamara visit hota hai toh aapi se nikalwa kar de deite hain

RA: aur kuch joh kehte hon aur koi mushkil ka saamna karna para ho

CHW: nahi aur toh kuch aisa nahi

RA: acha aap k khayal mein konsi cheezein community mein CHW k kirdaar ko behtar bananey mein madad kar sakti hai jaise aap ne kaha na k jab aap jaate ho kia kehte hain hamara kia faida hai aisi baatein kar rahe hote hain toh aap bataayein aisi konsi cheezein hain community mein jis se community wale aap se khush hojaayein

CHW: matlab yeh hai separate un k liye facility ho jaise delivery wagera jaise aap ko bataya yeh saari cheezein toh is tarhan se matlab hum jaayeinge hamein aur ache se bataayeinge k matlab k log aise hote hain chupa rahe hote hain pregnancy wagera bhi jaise newborn hota hai who bhi kuch log aise hote hain bache ko chupa deite hain k ab aga kha wale aaye hain in ko batana parega toh who bolte thy nahi nahi yeh hamara bacha nahi hai kisi aur ka bacha hai kuch log aise bhi hote hain toh yeh hai unhein center mein sahulat mil rahi hoti hai aur ache se bataayeinge information deinge

RA: aur aap ka kaam bhi acha hoga

CHW: jee aur yeh hai k hum folic acid deite hain aur pharas deite hain agar hamein kuch aisi medicines hain joh hum unhein de sakhein hamein aage se bola jaaye aap yeh de sakte hain toh agar hum unhein ja k dein unhein guide karein hosakta hai kuch acha hojaaye refusals kam hojaaye aur jaise convince agar mil jaaye jaise unhein pehle le k jaate thy vaccination k liye laate thy checkup k liye laate thy toh us mein aur PW jaise hum deite hain toh us mein matlab call wagera kar k pooch leite thy yeh sab aise log hain un k paas balance bhi nahi hota

RA: sahi acha aur aap ki raaye mein household ki sateh par hamal aur nauzaida bachon k hawale se kaunsi maloomaat deini chaiye

CHW: matlab joh important maloomaat hain un ko hamein joh hamal wali aurton ko aur bachon ko deini chaiye yehi k ek toh matlab health k hawale se aur phir unki matlab joh tetenis k teeke lagte hain k polio teeke lagte hain who lagwaayein laazmi is k elawa matlab delivery apni hospital mein hi karwaayein kisi daayi wagera k paas na karwaayein aur matlab kuch log nahi maante hum un ko yehi bolte hain k aap agar daai se bhi karwati hain toh un ko bolein saari cheezein naai istamaal karein use wali istemaal na karein kyun k aksar log bilkul hi nahi maante k hum log nahi jaayeinge hospital waise toh hum log ziadatar yehi bolte hain aap koshish karein aap hospital mein hi naam likhwaayein aur is k elawa jab baby hojaaye toh us ko mother feed pe matlab rakhein feeder wagera na dein aur jaise jin ko nahi araha ya aate hi nahi hain pehle bache ko nahi pilaaya toh hum bolte hain aap koshish karein aap mentally socheingi mujhe apne bache ko feed karwaana hai toh aayega toh agar koshish karein k aap nahi karwaayein is k elawa na dein bache ko khulla na rakhein is tarhan se aur vaccination bache ki laazmi karwaayein

RA: sahi aur aap k paas koi raaye ya khayalaat hain joh aap surveillance k kaam k baare mein batana chahti hain jaise aap ne kaha na k yeh cheezein honi chaiye community mein tabhi hamare kaam mein aur behtari aayegi toh aap ki raaye janna chah rahe hain aisa kia hai joh surveillance k kaam ko aur behtar bana sakein behtari a sakti hai community mein who kia hai aap k khayal mein

CHW: yehi jaise matlab aur jaise medicines wagera hai aur jaise calpol yeh deite hain toh who log bolte hain k syrup wagera taaqat ka syrup wagera deite hain aur jaise vaccination k hawale se bohat saare bache aate hain kyun k vaccination horahi hai is k elawa bhi aur koi matlab jaise ajaaye jaise treatment wagera jaise ultrasound hai agar ultrasound yahan hone lag gaya toh log ziada aayeinge aur matlab ache se response bhi deinge kyun k log ziadatar ultrasound ka bhi bolte hain us k elawa delivery yahan pe hone lag gayi us k liye bhi matlab bohat ziada

RA: sahi aap k khayal mein surveillance k kaam k baare mein kia batana chahti hain surveillance k kaam k baare mein

CHW: matlab k surveillance ka kaam kaisa rahe surveillance ka kaam acha hai yeh hai

RA: joh cheez aap k zehan mein ho joh aap batana chah rahi hon surveillance k kaam k hawale se joh bhi joh aap ki raaye hain ya khayalaat hain aap k zehan mein

CHW: abhi tak toh sab hi acha hai surveillance k kaam mein kuch aisi matlab kharab nahi hai joh aap ko bataon jaise sab hi acha chal raha hai aur matlab field wagera pe agar koi masla bhi hota hai phir yeh hai k api ko bata deite hain who hojaata hai solve aur kuch aisa bara issue nahi hai joh mein discuss karon sab hi acha hai

RA: sab hi acha hai bas

CHW: haan TAB bhi achi hai surveillance k doraan joh TAB use kar rahe hain aur matlab mushkil nahi hai easily samjh mein ajaata hai matlab aur har cheez aur koi sab acha hai

RA: bas aur kuch batana chaheingi

CHW: nahi aur kuch nahi

RA: bas chalein thek hai shukriya …..

Date: 01122020

SS: acha aap ka naam kia hai

SRA: NAME

Saima: NAME

SRA: Jee

SS: acha NAME aap ki umar kitni hai

SRA: meri umar mashaallah ziada hogayi hai 51 (laugh)

SS: 51 mashaallah aur aap ki taleem

SRA: mein ne master kiya hai

SS Master kiya hai

SRA: jee IR mein

SS: IR mein master kiya hai

SRA: jee

SS: sahi aur aap k kaam ka tajurba kitne saalon ka

SRA: tajurba agar aap poochein mein ne toh first job 1992 se dr maulla hua karte thy jab aga khan ka structure bhi joh hai na bilkul change tha us waqt se mein ne job ki hai pehli job thi toh who mein ne ki hai JPMC mein takreeban 9 maheeney ka who project tha persistant diarrhea par toh us k baad meri govt job hogayi thi toh mein ne job chor di thi phir is baad mein ne dobara join kiya hai 2004 mein toh mein 2004 se le kar 2009 tak joh mein ne hai na CHS department mein hi kaam kiya hai community health sciences aku mein phir us k baad ek 9 maheeney tak takreeban who aa meri bhaabi pregnant thi thore masle masaail thy phir who job chor di phir mein ne 2012 mein peads department join kiya tha ANISA mein

SS: sahi toh andaazan kitne saal hogaye honge

SRA: andaazan aap laga lein poori aadhi umar guzar gayi job kar k (laughing)

SS a: 20 30 saal guzar gaye

SRA: jee jee bilkul

Saima: aur surveillance mein aap ko kitne saal huye

SRA: surveillance mein aayi hun phir asal mein mein ANISA mein thi toh refusal hote thy blood sample k dusre aur community k toh un mein bohat ziada kaam kiya hai kyun k meri counseling or motivation sab kehte hain bohat achi hai Alhamdulillah toh us pe mein ne kaafi arsay kaam kiya hai toh phir ANISA khatam hua toh phir mein aayi thi ilyaas sir k paas aayi hun mein toh us waqt se mein surveillance mein hun takreeban 6 saal toh hogaye

SS: 6 saal hogaye hain

SRA: jee

SS: acha pehle ki nisbat se ab joh aap surveillance mein kaam kar rahi hain us hawale se mujhe bataein aap kaisa mehsoos kar rahi hain

SRA: kaam toh kaam ki nauyat joh hai who change huyi hai thori si aur thori si un ko behtar bhi kiya hai lekin SRA k liye joh hai na challenges bohat barh gaye hain ab hamein akele jaana hota hai na hamare sath assistant hoti hai hamein DSS chale doodh gaye kisi tarhan se bhi chalein yeh block pe pohanch gaye wahan jab jaate hain toh dangers joh hai dogs hamara istakbaal karne ko khare hote hain ek ghar pe mwin ne abhi khola hi tha gate toh saare dog ek dam se aaye aur mein ne itna bhaari weight tha un ko mein ne kis tarhan se band kiya hai poori jaan nikal gayi mein dar gayi hun is k elawa joh logon k community mein jab jaate hain ghar pe aap ne aap ne knock kiya kis tarhan se hum se behave karte hain attitude dikhaate hain yeh sab hamein bardash karna parta hai

SS: acha toh jab aap jaate ho toh us ka attitude aap k sath kaisa hota hai aap ne kaha bardash karna par raha hai kyun

SRA: kyun k baaz log hote hain na joh matlab totally refuse hote hain ziadatar mein joh hamari team k paas refusals aate hain na yahan pe Alhamdulillah refusal ratio kam hai kyun k mein sab ko na attend karti hun toh mein wahan jab jaati hunt oh un ka pehla joh hota hai na yeh samjhe un ka body language se bhi aisa lagta hai k who mau pe darwaaza maar raha hai aur kabhi kabhi toh darwaaza kholne ki zehmat bhi apne aap ko nahi deite aur wahin se kehte hain bache se kehalwaate hain k who nahi hain phir un ko counseling karte hain baat karte hain toh phir Alhamdulillah toh who agree hojaate hain lekin yeh hai k bohat tough time hai joh us waqt guzarta hai na ek ne toh is tarhan bol diya bas samjhe taange tor dunga dobara agar idhar aaye abhi ki abhi ki baat hai toh mein ne kaha k aap se DOB

SS: aur yeh surveillance k hi doraan hua

SRA: jee jee surveillance k doraan han mein apna hi kaam kar rahi thi PW EDD approaching ka toh mein gayi hun mein ne poocha k who khatoon ka naam liya who joh thy hum ne kaha who beithi huyi thi khatoon khana kha rahi thi un k husband ne kaha aankhein aisi bahar nikaal k aur ek kamre se doosre doosre se teesre mein akeli gayi toh mujhe dar bhi laga koi bhi nahi tha mein akeli hun unhon ne kaha andar ajaayein ek k baad doosre teesre mein jab gayi hun toh who log nazar aaye toh phir unhon ne mujhe kaha k kia faida hai hum kyun bataein hamein nahi batana aap ko aur matlab koi baat ek toh hota hai na aap agree us ko karte hain joh aap ki baat sun sakta ho magar who sunne k rawadaar nahi thy who keh rahe thy k mein aap ki koi baat sunna nahi chahta aap yahan se jaayein ainda qadam nahi

rakhiyega hamare ghar mein mein ne kaha thek hai koi masla nahi hai aap nahi batana chahte na batayein lekin sirf aap DOB bata deina bacha kab paida hua hai phir hum log nahi aayeinge manah kar deinge worker ko us ne kaha nahi bataonga kia kar loge mein ne kaha kuch nahi kar sakte aap ki marzi thek hai Allah hafiz toh kabhi kabhi aisa bhi hota hai k itna tough hota hai hum un ko agree nahi kar sakte

SS: aur yeh kia aap ko kia koi task diya jaata hai k aap is particular area mein jaayein

SRA: jee jee hamara task yeh hai k wohi EDD approaching kar k hum log jitni bhi pregnant lady hoti hain un ki EDD k hisaab se kab suspect hain toh hum ja kar un ko visit karte hain phone number hote hain agar un k phone number available hain toh phone number bhi hote hain available bhi hain phir bhi who phone uthaate nahi hain kabhi kehte hain office mein hain kabhi kehte hain mein un ka bhai hun koi kehta hai devar hai jeith hai who samjhe na hone k barabar ziadatar phir us k baad hum un ki visit karte hain phir visit k zariye outcome leite hain un ka k bhae bacha hogaya hai k nahi toh batatey hain k vaccine karni hai kis tarhan bache ka khayal rakhna hai apni feed kis tarhan karwaani hai yeh 2 3 cheezein un ko hum knowledge deite hain

SS: acha jaise aap jaati hain un ko jaise aap ne kaha k who darwaaza band kar deite hain ya aisa kuch k baat sahi nahi karte hain toh wajha kia hai joh is tareeke se kar rahe hain aap k sath

SRA: who yeh kehte hain aap joh aate ho aap ki toh job hai thek hai toh aap toh poochte hain aap ki salary kitni hai yeh falah kitni hai is tarhan k question karte hain who yeh aap kit oh job hai kehte hain hamein kia faida hai toh un ko un k faide ginwaate hain ghar beithe aap ko service mil rahi hai ghar beithe aap k bache ka deakhein yahan pe han mein kehti hun mere ghar mein bache hain aur mein khud aga khan mein linon mein lag k apne bache ko dikhaati hun fees bhi pay karti hun aur aap ko na fees deini hai na line mein lagna ghar mein a k aap k bache ko itna acha checkup karein ek ek cheez koi joh bhi hai aap ko inform bhi kar rahi hain toh who kehte hain yeh sab thek hai lekin hamari delivery saasein joh hoti hain na ziadatar who yeh kehti hain k aap yeh bataein delivery ka joh masla tha meri bahu k sath yeh hua beti k sath yeh hua us waqt toh aap khare nahi thy sath toh un ko yeh chaiye k delivery ho kam se kam mein yeh kehti hun lekin k antenatal checkup toh hona hi chaiye zaroori hai hona chaiye mothers ka na kyun k antenatal checkup ho who log bhi chahte hain dusra who yeh kehte hain medicine mein kaali aur peeli goli sahi hai hum log deite hain folic acid aur pharasulphate toh yeh toh govt se bhi mil jaati hai aur in ki toh quality baaz bohat educated log bhi hote hain who keh kehte hain educated na bhi ho un mein is cheez ka sense hai k who qeemat deakhte hain who kehte hain yeh toh itni sasti hai yeh toh hum khud le leinge matlab k mehangi wali ho jahan hum antenatal checkup kara rahe hain who medicine unhon ne hamein diye prescribed kiye who hamein aap k center se milni chaiye un ka yeh kehna hai dusra yeh k hum jab jaate hain toh us ghar mein aged khawateen bhi hoti hai toh who yeh chahti hain k aap koi kehti hain mere taangon mein dard hai ghutno mein dard hai koi falah problem hai sugar ki yeh toh general OPD bhi yeh log chahte hain ho toh mein toh un ko matlab yehi counseling karti hun k deakhein hum apne ghar ko chalaatey hai na ek budget diya jaata hai hum roz biryani nahi paka sakte roz sabzi nahi paka sakte kabhi biryani pakaate hain kabhi qorma kabhi sabzi kabhi daal toh hamare paas bhi ek budget hai aga khan ka inshaallah kabhi Allah taala ne itna budget mein ijazat di toh hum aisa bhi kareinge k deliveries bhi hongi aur antenatal checkup bhi hoga aap k liye general OPD bhi

hogi lekin yeh thora thora kar k hi hoga aap corporate karein joh service aap ko de rahe hain us se aap faida uthaayein toh aage kaam hoga

SS: sahi phir un ki taraf se kia response aaya jab aap ne kaha

SRA: bohat se bohat khush hojaate hain who kehte hain aap sahi keh rahi hain han bache ka checkup kar lein aap deakh lein lekin yeh hai who un ka concept yeh bhi hota hai k agar healthy bacha hota hai who kehte hain nazar lag jaati hai bache ko

SS: acha

SRA: han un ka concept yeh hota hai k nazar lag jaati hai khaas tor pe boorhi aurtein hoti hai who kehti hain yeh toh mein aisi hi misaal deity hun mere ghar mein toh bache hain who toh mashaalllah itna healthy hai over weight hai leikin hum ne toh weight karwaya kuch nahi hua us ko phir is tarhan ki baatein kar k who karwa leite hain phir khush bhi hojaate hain baaz toh toh phir mein un ko joh rehri goth mein hamara hai us ki misaal deity hun deakhein wahan log cooperate karte thy joh service di un se faida uthate thy toh phir aage bhi kaam hua hai wahan par thek hai

Saima: toh jab who aa aap jaate thy jaise aap ne bata diya unhon ne kaha tha hamara kia faida

SRA: jee

SS: yeh kia jis jagha aap jaate ho yeh koi ek do gharon mein aisi baat hai ya aur bhi

SRA: nahi yeh ziadatar gharon mein mujhe toh itne log nahi kehte meri workers jab aati hain yahan par data deine k liye yeh sham mein who yeh batati hain yeh yeh refusal hain who yeh yeh comments batati hain phir mein jab un k paas jaati hun toh is tarhan ki baatein karte hain k hamein kia faida hai un ka yeh pehla lafz hota hai agar aap achi counseling kar lein motivation kar lein who agree hojaate hain bohat saari worker hamari mashaallah bohat ache hain abhi ek maheena chutti ki toh who bata rahi hain aapi aap nahi aayin thi jis tarhan aap baat karti thi mein ne baat kar k newborn ko agree kar liya matlab good zabardast

SS: aur joh pehle k project mein in ko koi faida milta tha ya abhi surveillance mein aap ko is tarhan se keh rahe hain k aap pehle wale project mein aisa tha k un ko faida hota tha

SRA: pehle project mein yeh tha ANISA mein hum log jab aa aa delivery hoti thi outcome aata tha toh jaise abhi hum ja k poochte hain outcome ka toh us waqt un ko gift deite thy chota sa jis mein ek bache ka suit hota tha wrapping sheet hoti thi aur lotion wagera yeh cheezein hoti thi choti toh who khush hojaate thy lekin aap ko pata hai har tarhan k log hote hain kuch log us ko bhi yeh keh deite thy yeh third quality ka aap ne diya hai yeh toh hum khud khareed sakte hain peer bazar se mangal bazar se who toh phir har tarhan k log hote hain lekin ziadatar log joh hai na khush hote thy k kuch na kuch toh aap ne diya hai na phir dusri baat yeh hai dusre project k log bohat saari cheezein de rahe hote hain ek project mein blanket hota tha lotion soap aur pata nahi kia kia cheezein de rahe thy toh phir unhon ne kaha aur ek mein joh hai who aga khan ka cup de rahe thy CHS ki taraf se project tha toh hum bhi gaye toh unhon ne kaha k aap ne kia diya mein ne kaha yeh hum ne toh diya hai yeh aga khan ka hi toh hai naam aap ka

kuch bhi nahi hai keh rahe hain aga khan hi toh de raha hai is tarhan se kar k mein ne un ko motivate kiya tha

SS: matlab is tarhan se kiya tha

SRA: jee

SS: sahi aur phir who maan bhi gaye

SRA: maan bhi gaye thy

SS: asaani se aap ko lagta hai k haan surveillance mein aap acha mehsoos kar rahi hain

SRA: surveillance mein acha hai toh lekin SRA k liye problem barh gayi k us k sath assistant nahi hai gaari bhi nahi hai us k paas pehle gaari hoti thi assistant hota that oh kaam ziada behtar tor pe ho paata tha abhi joh hai na hamein DSS ka itna ziada hamein nahi pata k matlab hamein dhoondna parta hai hamein numbering k liye thora time chaiye hota hai jitna time hum numbering dhoondne mein lagaate hain utna hamare paas assistant hon toh hum double se ziada kaam kar sakte hain yeh is liye deakha gaya hai k abhi thore dino mein aftab bhai aaye thy toh un k sath bhi mein ne listing kar k kiye visit toh mein ne 25 visit kiye thy toh matlab faida hota hai assistant hota hai kyun k un ko bhi pata hai numbering ka yeh hai kaam toh pehle bhi horaha tha abhi bhi horaha hai

SS: acha mujhe bataiyega k aap apne household visit se hamal ya nauzaida bachon ki deakhbhaal mein aap kia kirdaar mehsoos karti hain

SRA: us mein hum yeh hai k hum sab se pehle mother ko batatey hain vaccine ka BCG ka joh at birth lagana chaiye jaise bacha paida hota hai aap ne foran se BCG lagana hai kyun k Pakistan mein sab se ziada tuberculosis hai toh jitni jaldi hosake aap us ko vaccine laga lein bache ko dusra hum feed k baare mein un ko batatey hain us ko din aur raat mein kis tarhan se feed karni hai aur apna kis tarhan se khayal rakhna hai feed karne se pehle kis tarhan se us ne paani peena hai kis tarhan se apne joh bhi Allah taala ne us ko diya hai mein us ko yehi alfaaz istemaal karti hun jitna bhi Allah taala ne diya hai ek toh aap khush rahein dusra doodh peeyein joh sastey fruit hain aap who fruit lein rotiyaan khaayein ziada apna giza ka khayal rakhein aur kapron ka bhi hum batatey hain kyun k sardiyon mein khule khule kapron mein bache aise saamne hawa mein leite hote hain kis tarhan se un ki care karni hai bache ki sab se ziada vaccine k upar aur feed k upar ziada karte hain hum in ko proper tareeke se na feed dein aur upar se koi cheez na dein 6 maheeney tak

SS :sahi koi aise jinhon ne refuse kiya ho vaccine se

SRA: nahi mujhe toh koi aisa nahi mila lekin worker ko aise milte hain joh vaccine se refuse karte hain aise aur kuch toh joh bengoli family aisi hain joh kehte hain k hum ne kabhi vaccine lagaai hi nahi Ibrahim hyderi mein thi wahan pe sindhi community mein kuch log aise thy paarey mein who kehte thy hum ne kabhi bhi nahi lagaai na lagwaayeinge kyun k ek dafa kuch aisa hogaya tha bacha mar gaya tha vaccine expiry thi is ko kia pata who toh jis ne jis tarhan se un ko guide kiya un k zehan mein cheez beith gayi vaccine joh hai expired thi is wajha se who log nahi lagwaate

SS: acha

SRA: jee yeh hai Ibrahim hyderi k paare ki baat thi yahan pe aisa toh nahi hai lekin who yeh kehte hain koi faida nahi hai bache beemar hi hote hain aur kia hota hai un ko bohat samjhaate hain beemari aaj kal pehle zamaney mein jitni beemari thi us se kahin ziada abhi beemari hai toh vaccination bohat zaroori hai kabhi kabhi hum jaate hain toh mein poochti hun vaccine ka toh kehte hain is ka weight kam tha toh mein ne kaha is ko toh ziada zaroorat hai is ko toh double zaroorat hai aap samjh lein is ka toh weight kam hai is ko toh ziada beemariyan joh hai lagegi toh kehti hain acha thek hai hum lagwa leinge motivate hojaati hain

SS: sahi aur kia kia cheezein batati hain aap deakhbhaal k hawale se hamal aur nauzaida bachon k hawale se

SRA: hamal k hisaab se hum un ko diet ka hi batatey hain aur pehle toh hum chart bhi le k jaate thy un ko dikhaate thy k deakhein k kis tarhan se dastarkhuan pe log beithy huye hain aur joh pregnant hain aage ziada cheezein rakhi huyi hain who dahi bhi use kar rahi hai who sabziyaan bhi use kar rahi hain fruit bhi le rahi hain milk bhi le rahi hain waqfe waqfe se lein do do ghantey k baad lein ek sath ziada cheezein na khaayein aap lekin ziada se ziada jitni gunjaaish hai aap khaane ki koshish karein

SS : sahi thek hai acha aap ko kia lagta hai k rozmara surveillance k kaam k elawa zachki aur nauzaida bache k hawale se maloomaat farhaam karna chaiye

SRA: bilkul karna chaiye un mein awareness ki kami hai aur yeh bohat zaroori hai kyun k who aware hongi tabhi who vaccine lagwaayeingi tabhi who proper tareeke se feed karwaayeingi proper tareeke se diet deingi aur apni diet ka khayal tab hi rakh sakti hain

SS: kis tarhan se zachki aur bachon ki sehat k wasaail diye jaayein unhein

SRA: mein samjhti hun k zahir hai ek toh yeh hai ziada se ziada un ko who joh hai na counseling ki jaaye charts k zariye mukhtalif chote chote group bana k matlab un ko dikhaaya jaaye k yeh deakhein is tarhan ab kia horaha hai kis tarhan se diet le rahi hai kis tarhan se vaccine zaroori hai pregnancy mein kis tarhan se aap k liye doodh zaroori hai medicine zaroori hai aap k liye folic acid aap zaroor lein pharasulphate zaroor lein iron ki koi bhi tablet lein toh mukhtalif session k zariye bhi aap un ko bata sakte hain yeh baatein

SS: sahi aur kia kia cheezein batatey hain rozmara surveillance k jab aap jaate hain wahan par aur mazeed kia batatey hain

SRA: ziadatar joh hai hum log toh apne kaam k hawale se vaccination ka ziada batatey hain proper feeding ka ziada batatey hain aur wahan safai suthraai nahi hoti toh mein un ko yeh bhi batati hun k kis tarhan bache ko nehlaayein dhulaayein saaf suthra rakhein toh yeh bhi us ki sehat k liye bohat zaroori hai k safai nifs emaan bhi hai yeh bhi mein un ko bolti hun k yeh bohat zaroori hai

SS: thek hai aur aap k khayal mein aap ko maaon aur nauzaida or 5 saal se kam umar bachon ko maloomaat farhaam karne ki aap ko munasib training haasil hai joh joh cheezein aap ne unhein samjhaayi hain toh kia aap ko koi training di gayi thi

SRA: jee jab hum CHS mein thy mukhtalif training hum ne kiye hai na immunization ki bhi kiye or nutration ki bhi ki hai kaafi saari training hum ne kiye hain HOPE health mukhtalif NGOs HANDS mein wahan mein ne kaam kiya hai wahan pe bhi bohat saari trainings hoti rehti thi

SS: aur kin kin cheezon pe hoti thi training

SRA: nutration pe thi diet pe thi breast feeding pe thi aur is tarhan se PW k hawale se thi during pregnancy kis tarhan se un ko diet leini chaiye kitna kitna time period k baad leina chaiye kia kia leina chaiye sab kuch khana chaiye kuch log hote hain koi machli nahi khaata koi kuch nahi khaata har cheez ki ek zaroorat hai Allah taala ne hamare jism mein need rakhi hai who har cheez joh hai na aap ne leini chaiye phir who un ko samjhaate hain k har cheez jism ko zaroorat hai

SS: aur aur koi training ki aap ko zaroorat hai k honi chaiye

SRA: bilkul training toh aisi cheez hai k who toh honi hi chaiye jitna ziada training hogi utni ziada hamein knowledge hogi aage hum log brief kar sakte hain na

SS: kis qisam ki training aap chah rahe hain k honi chaiye aur kitne kitne arsay mein

SRA: mein sochti hun har 3 maheeney k baad trainings honi chaiye antenatal care pe honi chaiye immunization par honi chaiye breast feeding k hawale se honi chaiye kyun k maaon ko nahi pata kis tarhan se pehla doodh joh hai nikaal k pheink deity hain toh yeh sab cheezein honi chaiye waise jitni mujhe maloomaat hai mein worker ko batati rehti hun

SS sahi toh is se bhi aap k kaam mein aur behtari aayegi

SRA: behtari aayegi bilkul

SS: behtari aayegi

SRA: bilkul

SS: sahi toh aap k khayal mein maaon aur nauzaida ki deakhbhaal mein k aap ko kia lagta hai as a sahulatkaar aur nauzaida maaon k darmiyaan aap ka kia kirdaar tha aap ka kia kaam tha

SRA: matlab

SS: matlab k jaise aap jaate ho community mein facilitate karte ho

SRA: jeejee

SS: community mein aap service provide karte ho aap un ko batatey ho k haan yeh yeh hamari sahulat hai sahulatkaar mein aap aayein is tareeke se aap baat karte ho toh us un maaon k aur sahulatkaar k darmiyaan aap ka kia kirdaar tha

SRA: coordinate karte hain hum toh bas aur kia karte hain hum toh itna kuch un ko provide karte nahi hain ziadatar yeh k counseling karte coordination rakhte hain un se achi hum log dusra yeh hai agar joh hai who pick n drop un ko de dein toh un ki immunization bohat behtar hojaayegi kyun k bohat saare log isliye nahi a sakte yahan aane mein un ka kiraya itna kharch hota hai toh phir who nahi aate

SS: toh kabhi aisa hua hai k matlab k naraz hogaye hon ya bilkul bhi aana nahi chah rahe ho refuse pick n drop nahi mil raha aisa kuch

SRA: jee worker batatey hain who kehte hain k pehle milta tha aura b nahi milta lekin direct meri tarhan ki baat un se nahi huyi na

SS: sahi

SRA: lekin chahte hain who pick n drop ho yeh bhi chahte hain checkup ho yeh bhi chahte hain delivery ho chahne mein toh sab kuch chahta hai lekin yeh mein samjhti hun k thora kuch hamein un ko provide karna chaiye kam se kam antenatal checkup karein un ka aur medicine thori behtar dein dusra yeh k bachon yahan pe dikhaane aate hain kehte hain k in ko panadol drops k elawa aur nasal drop k elawa chahe koi bhi beemari ho yeh do cheezein deite hain jab k bache ko antibiotic ki zaroorat thi inhon ne kaha hum yahan le k aaye yeh cheez de di phir us ko falah jagha le gayi sultan le gaye 5 number le gaye toh wahan antibiotic di gayi bacha sahi hogaya aur mein kehti hun aisa nahi hai antibiotic maujood hai aur agar aap ko need hoti toh who zaroor deite aisa thori hai agar hai aap k liye center hai mein kehti hun joh doctor beithy hain agar aap waise jaoge toh aap ko pata chale kitni fees leite hain kitna aap ka time waste hota hai joh aap ja k un ko dikhaate ho without fees aap dikha rahe ho aap ko isliye qadar nahi hai na asal mein toh mein is tarhan kehti hun toh kuch hanste hain kuch bura maante hain phir mein unko samjhaati hun k deakhein yeh bohat zaroori hai aap yeh samjhein k doctor aap ko joh de raha hai na who doctor hai aap apni marzi se dawaiyaan nahi le sakte toh joh doctor ne behtar samjha wohi diya kyun k ziadatar shikaayat yehi hoti hai k do cheezein deite hain

SS: ok aur yahan jab aate hain yahan pe joh k un k sath maaon k sath matlab un k aa joh behavior hai who kaisa raha

SRA: haan yahan ka staff joh hai yahan mujhe asal mein AG mein aaye huye mein ne join AG mein kiya tha lekin abhi AG mein aaye huye mujhe ziada arsa nahi hua mein july mein AG mein aayi thi toh mein IH mein thi toh yehi shikaayat karte thy k joh hai na attitude dikhaate hain yahan par bhi ek doctor k baare mein bolte hain who attitude dikhaate hain

SS :kahan pe yahan

SRA: yahan par bhi

SS: acha

SRA: jee aur do joh hai ek joh hai who bohat achi hain un ki tareef karte hain k who bohat achi hain mashaallah bohat achi tarhan se samjhaati hain bohat achi tarhan batati hain deakhti hain who kehte hain matlab hamara bacha agar mau dhoya hua nahi hota toh who us ko hath lagana pasand nahi karti k bhae kaise le k agaye mau kyun nahi dhoya is ka aur kapre kaise pehnaaye huye hain is tarhan ki baatein karte hain toh kia agar agar tum ache kapre pehan lo mujhe phir refusal pe kaha k agar aap ache kapre pehan lo toh is mein hamara kia kasoor hai aur agar hum bure kapre pehan rahe hain toh aap ko koi haq nahi hai k aap hamein kuch kahein

SS: aur yeh kab ki baat hai

SRA: yeh IH mein thi toh yeh us waqt ki wahan ki baat hai yahan ki nahi unhon ne mujhe kaha toh mein ne kaha bilkul aap bilkul sahi keh rahi hai aap ka bhae haq hai k aap joh dil chahe pehno lekin saaf suthra rahein is mein koi bura maanne ki baat nahi hai leikin kehne ka tareeka hota hai ek aap baat kar rahi unhon ne jis tarhan bache ko hath nahi lagaya aisi deakh k kaha k han dawa is ko de dou toh baaki bache ki condition thi who aisi thi k insan hath lagaate kahe lekin doctor hai us ka kaam hai us ko chaiye k who us ko ziada se ziada maa jab a rahi thi garmi k din thy koi aisa masla nahi tha lekin bas apna apna who insan ki baat hai

SS: yeh toh chalo IH ki baat batai yahan pe aisa kuch

SRA: yahan pe nahi abhi tak toh aisa kuch nahi hai attitude k baare mein kuch nahi hai dikhaate hain bas time ka kehte hain time lagta hai toh who toh time lagega aap kisi bhi doctor k paas jao kabhi foran toh aap nahi dikha paate na

SS: sahi acha aa aisi konsi cheezein hain joh aap k kaam k hawale se k joh aap sunti hain joh aap ko acha lagta hai aap ki hausla afzai horahi hai aur aap kehti hain k jaise hausla afzai horahi hai toh aap chahte hain k aur mazeed kaam behtar se karein

SRA: acha us mein yeh hai k abhi hamare yahan nay eh malika joh hain who vaccinator hain aur who har do din k baad a k clapping karti hain kehti hai mashaallah VR ka joh staff hai itni achi couselling kar raha hai jab yeh log jaate hain aur un se poochte hain k bhae card dikhaayein aap k paas card hai jab who card deite hain jab yeh entry kar rahe hote hain toh yeh sirf apne kaam se kaam nahi rakhte sirf entry nahi kar rahe hote hain is ko batatey hain k deakhein yeh date hai is date pe aap ko yeh teeka lagega is date pe yeh teeka lagega agar nahi lagwaaya hota toh motivate karte hain aap ki bache ki age itni hai toh yeh teeka lagwaayein toh keh rahi hai k bohat ratio bohat barh gaya hai aur IH se thora hi kam hogaya hai mashaallah bohat acha log joh hai na immunization karwa rahe hain khud malika ne kaha 2 3 dafa a k

SS: sahi aur community mein kuch suna ho apne baare mein acha

SRA: community mein toh aisa kuch nahi

SS: lekin aap ko acha lagta hai k aap ki hausla afzai hoti hai aap k baare mein acha bolte hain

SRA: jee bilkul jee aur sab se bari baat yeh hai k insaaniyat bhi hai na mein worker ko bhi yeh bolti hun k sirf yeh nahi deakho aap ki duty kia hai kuch cheezein insaaniyat se bhi taaluk rakhti hai jaise pick n drop

nahi hai ek ghar mein hum gaye hain monitoring k liye wahan bache ko itna ziada high grade fever tha toh hum apni monitoring karte rahe yeh toh koi baat nahi huyi na hum apna kaam chor k us ko doctor k paas le kar aaye mein ne kahan koi baat nahi hum us ko le k jaate hain hum drop karwa deinge toh yeh kuch cheezein insaan ko us waqt decision leina parta hai chahe who aap ko permission na bhi dein aap k liye zaroori hai k who bacha mar jaaye us se behtar hai k aap us ko bachao insaaniyat yeh hai na worker bhi mashaallah bohat ache hain yahan par

SS: sahi thek hai acha kia aap surveillance k kaam k doraan aap ko koi rukaawaton ka saamna karna para tha community mein surveillance k doraan

SRA: surveillance k doraan yehi rukawatein hain k who log log joh hain who hamare upar trace hote hain ek dafa mein aur rehana block 1 mein monitoring kar rahe thy rehana thori si aage nikal gayi mein peeche thi mere hath mein TAB tha mein kar rahi thi monitoring who mere sath thi toh peeche se ek larka aaya kaha k zara aap apne TAB ko bacha k rakhein mera dil itna dar gayi mein itni tez tez hote huye rehana k paas gayi zahir hai meri toh ek age hai experience hai mujhe itna dar laga toh phir larkiyon ko bhi lekin phir bhi who do hoti hain na toh un k sath itna masla nahi hota jitna hum logon ko hota hai yeh hota hai dusra who dog peeche lag jaate hain toh who itni lambi lambi row hain ek gali mein joh hai kam se kam 20 20 40 40 ghar hain bhaag bhi nahi paata banda itna

SS toh kia kehte hain log

SRA: who peeche ek dam saare lag jaate hain bache shararati hote hain who khud peeche laga leite hain pathar maarte hain yeh karte hain who karte hain matlab cheir chaar karte hain larke hote hain comments paas kar rahe hote hain aray yaar hamein bi de dou itna acha TAB diya hua hai yeh sab cheezein toh zahir hai itni is ko toh ignore hi karta hai banda

Saima: aur kabhi aisa laga k aa corona ki wajha se bhi kuch aisa keh rahe ho

SRA: corona ki wajha se jab mein monitoring k liye gayi toh mujhe unhon ne kaha k yaar aap k ghar waale ko aap aziz nai ho mein bhi mask hi pehni huyi thi aur mein ne kaha nahi kyun aap aise kyun keh rahi hain toh kehne lagi duniyan ghar mein beithi huyi hai aur aap joh hai na is waqt aap yahan par log darwaza bhi kholna pasand nahi kar rahe aap keh rahi hain toh mein ne un ko kaha k deakhein agar sab ghar mein beith gaye toh bahar kaam kon karega sab ghar mein toh nahi beith sakte kuch arsey k liye ghar mein beithe honge leikin yeh toh hamara kaam hai toh phir unhon ne kaha k aap ko duaaon mein yaad rakhongi mujhe itni khushi huyi k kitni achi aunty hain keh rahi hain mein aap ko duaon mein yaad mein ne kaha bas duaon mein yaad rakhein nahi nahi mein aap ko duaon mein yaad rakhungi

SS: kisi ne aisa refuse kiya ho ghar aane se corona ki wajha se

SRA: nahi nahi aisa nahi kabhi bhi nahi bilkul bhi nahi bilkul sahi hai hum monitoring k liye jaate hain who chaai tak kehte hain chai lein thanda lein gents joh thy who mujhe kehne lage PW outcome k liye EDD approaching k liye toh kehne lage baho ko in ko kuch khilaya pilaya nahi mein ne kaha uncle aap ne mujhe izzat se bithaya joh mujhse baat ki mere liye yehi kaafi hai unhon ne kaha nahi mein aap ko jab tak nahi jaane dunga jab tak kuch khilaonga pilaonga nahi who itne mere peeche par gaye jaldi bataein

jaldi phir mein ne socha yaar kia karon mujhe toh bohat kaam hai chaai ka kahongi dil bhi chah raha tha sardi bhi thi toh time lagega phir mein ne kaha uncle aap thanda pila dein jab mein ne kaha toh fata fat juice la k mujhe diya toh mein ne who piya itne ache log bhi hote hain joh aap ki itni care karte hain

SS: jaise aap ne kaha tha k shuro mein bataya tha refuses k hawale se aap ne bataya tha

SRA: jee

SS: thek hai na joh k aap k kaam mein bhi dushwari aayi thi refusals k hawale se ab joh hai gali mein joh hai larke who bhi taunt karte hain

SRA: jee bilkul

SS: sahi aur is k elawa bataein aisa waqiya bayan karein joh hua ho surveillance mein rehte huye

SRA: surveillance ka toh nahi hai ANISA mein thi us waqt hua tha surveillance mein aisa koi waqiya nahi hua k qabil e gor ho

SS: ok sahi aap k khayal mein konsi cheezein community mein CHW k kirdaar ko behtar bananey mein madad kar sakti hai

SRA: community mein ek tou yeh un ko knowledge honi chaiye kuch aap pooch rahe hain toh aap chup na rahein aap apne hisaab se koi na koi jawab de k us ko farig karein aap chup raheingi kuch keh nahi paayeingi toh matlab un ki knowledge zaroori hai knowledge zaroori hai dusra un ka bolna baat karna mein un ko kehti hun itna zor se toh aap bolein loudly saamne wali ko aap ki awaaz aaye bolna aana chaiye aur yeh k knowledge ki bohat zaroorat hai

SS: zaroorat hai aur kia hum CHW k kirdaar ko behtar bananey mein madad kar sakte hain konsi cheezein joh who khush hon unhein kia zaroorat hai kis cheez ki zaroorat hai

SRA: acha un ko deakhein ek toh yeh hai zahir hai training ki zaroorat hai un ko un k mukhtalif session hon workers k bhi us mein un ko knowledge milegi aur certificate aap un ko deinge who bhi khush hongi

SS: sahi yeh toh baat hogayi CHW ki thi mein chahti hun k joh aap se poochna chah rahi hun who hai community se related hum aisi konsi cheezein community mein dein jin se CHW k kirdaar mein behtari a sake madad mil sake community

SRA: community mein jis se refusals rate kam ho aap ka matlab hai toh us mein mein sab se bari baat yeh hai k ek toh yeh hai joh matlab un ko jis cheez ki zaroorat hai who yeh keh rahe hain agar hamein need hai farz karein aap k paas nahi hai service lekin hamein emergency ki surat mein agar aap ki gaari chaiye hum delivery hai aap who us waqt deinge gaari un ko provide kar dein kisi tarhan bhi karein rent pe leike dein un ko dein who afford nahi kar sakte mein yeh samjhti hun dusra yeh hai who vaccine k hawale se k who pick n drop jaise de dein toh bohat behtar hojaaye vaccine k hawale se ya sick baby ho us k hawale se bhi toh agar aap provide kar dein pick n drop k liye van toh who behtar hojaayega

SS: toh is se CHW k kaam mein bhi

SRA: CHW k kaam mein asaani hojaayegi refusal rate kam hojaayega log bhi samjheinge k hamein kuch mil raha hai

SS: jaise aap ne shuro mein kaha k kia faida

SRA: Jee haan aap deite kia ho yeh jumla worker ko kehte hain k aap deite kia ho

SS: Sahi matlab yeh cheezein agar hum deina shuro karein toh phir behtari aana shuro hogi

SRA: bilkul Alhumdullilah zaroor

SS: sahi aur aap ki raaye mein household ki sateh par hamal aur nauzaida bachon k hawale se konsi maloomaat aur unhein deini chaiye

SRA: maloomaat toh yehi hai k aap un ko joh hai breast feeding k hawale se bataayein immunization k hawale se bataayein k kab kab kis age mein konsa teeka aap k liye zaroori hai lagna bache ko kis tarhan se aap feed karaani hai din aur raat mein upar se aap ko koi cheez nahi deini hai koi giza nahi deini hai do do maheeney mein khilaana shuro kar deite hain k bacha sehatmand hojaayega toh yeh sab cheezein un k liye zaroori hai k aap un ko motivate karein k kia karna hai kia nahi karna

SS: acha koi aisi cheez joh aap batana chahti hon jis se aap k kaam mein aur behtari a sake

SRA: us mein sirf yeh hai k assistant de dein gaari nab hi dein assistant de dein us se hamare kaam mein aur behtari ajaayegi bohat ziada hamara time bachega toh kaam ziada kar sakeinge

SS: acha aap k paas koi aur raaye ya khayaalaat hain joh aap surveillance k hawale se batana chahti hon

SRA: nahi aur toh koi nahi hai joh thi who aap k sath share ki

SS: joh bhi zehan mein hai aap k joh batana chah rahi hon koi bhi raaye khayaalaat surveillance k hawale se

SRA: nahi koi aisach toh nahi hai bas yehi hai k behtari k liye zaroori hai k training session hona chaiye us k zariye thori behtari aayegi community mein worker mein hum mein bhi zahir hai training ki zaroorat toh sab mein hoti hai aur dusra yeh hai k thora san a encourage kare yeh log thora aisa lagta hai mujhe jaise k na encourage nahi karte acha kaam bhi karo na toh bas normal behave rakhte hain aur bura kaam karo toh sunni bohat parti hai toh is se thora na demotivate hota hai banda yeh hai mein yeh samjhti hun meri personal yeh raaye hai k agar ek baar aap mein koi deficiency hai toh thek hai ek dafa aap ne keh diya baar baar us ko le k deficiency ko kehte rahoge toh mein toh yeh samjhti hun thek nahi hai na yeh thora sa

SS: sahi aur koi raaye

SRA: nahi bas

SS: bas chalein shukriya…..

Date: 05112020

SS: acha shazia aap ki umar kitni hai

CHW: 26 matlab k abhi 27 hojaayeinge isi year mein

SS: acha aur aap ki taleem kitni hai

CHW: taleem BSC kiya hai aur abhi continue hai

SS: aur aap k kaam ka tajurba

CHW: tajurba takreeban waise toh 8 saal hogaye jaise hum log DSS ya surveillance us mein kaafi time se chal SShe hain 7 saal

SS: surveillance ko mila kar 7 saal

CHW: jee

SS: acha shazia mujhe bataiyega aap apne baare mein mujhe bataein joh 7 saalon mein aap ne kaam kiye hain us mein aap kaisa mehsoos kar SShi hain

CHW: mein field k hawale se jaise hum matlab k yahan se jaate hain field par toh kaafi acha hai response hamaSS field k hawale se bhi aur kaafi kuch bhi hum logon ne yahan matlab k seekha bhi hai aur bohat saare fawaid hamein bhi huye community ko bhi huye hain aur is k elaawa jaise kuch aisi cheezein thi pehle un ka itna idea nahi tha k phir baad mein kaafi matlab k aisi cheezein hum ne field par deakhi field site k hawale se aur baaki cheezein face ki toh us se hamein kaafi in cheezon ka mutalla bhi hogaya hai jaise hamein idea thi in cheezon ka

SS: kia cheezein aap ne deakhi

CHW: jaise hum log agar field par jaate hain toh us se pehle bhi hota tha hum matlab k community mein kisi ko itna jaante wageSS nahi thy toh us k hawale se kaafi logon se hamaSS introduction hua aur un k sath kia kia masail hote hain ya un ko hamein kis tarhan guide karna hota hai us k hawale se un ko bhi faida hua hamein bhi matlab k in cheezon se hamein nayi nayi cheezein seekhne ko bhi mili [

SS: acha toh unhein kaise faida hua

CHW: unhein jaise ab joh cheezin un logon ko maloom nahi hoti hai ya jaise matlab kisi ka baby hota hai toh un ko itna idea nahi hota feed karwaane ka ya matlab us k hawale se aur bhi kaafi cheezein hoti hain joh maayein akdar ghabSS jaati hain un ko hum log sahi guide karte hain tareekekaar un ko batatey hain bachon k hawale se toh acha kaafi hamein response milta hai is hawale se

SS: acha toh ghabSS kyun jaati hain jaise aap ne kaha na ghabSS jaati hain maayein

CHW: ghabSSna jaise matlab k aksar kuchyeh pehle logon ki k matlab hota hai na k hamein kia faida milega is se aap log aate ho apna hi faida le k jaoge likhoge karoge phir chale jaoge toh unhein hum pooSS guide karte hain is se aap ko kia kia faida hoga bachon k hawale se un ki vaccine k hawale se un k medicine k hawale se yeh saaSS hum log un ko guide karte hain toh pehle yeh hota tha k kaafi log vaccine apne bache nahi karwaate thy ab mashaallah se har koi apne bachon ki vaccine mukammal karwaate hain

SS: acha toh phir response kia hota tha jab aap kehte thy na k aap k liye faida kar SShe hain

CHW: un ka shuro shuro mein yeh response hota tha k hamein nahi karwaana ya jaise kuch log pehle hote thy sakht mizaaj k aksar bolte thy pathan log kabhi bhi vaccine nahi karwaate lekin hum kehte thy who ziadatar abhi matlab k is community mein ziada se ziada wohi log apne bachon k vaccine mukammal karwaate hain pehle who manah karte thy k is se shayad nuksaan ka hoga lekin hum un ko pooSS guide karte hain un k fawaaid batatey hain ek ek tikke ka kia kia faida hota hai who cheezein kaafi ab un ko itminaan hogaya aur center se bhi is hawale se yeh hai k koi nuksaan hamein nahi pohchaaiyenge hamare bache k faide k liye aate hain

SS: ab jab surveillance kar SShe hain who

CHW: us mein bhi yehi cheezein same pehle hum log matlab k vaccine k hawale se batatey thy lekin ab un k pooSS record hamein leina parta hai aur jab record un ka hum apne paas jaise hun log k kitne community mein aise bache hain jin ki vaccine mukammal hogayi hai kaafi cheezein change bhi hoti hai lekin bohat acha hamein kaam karne mein bhi achi cheez aur nayi se nayi cheez seekhne ko milti hai doosSS yeh hota hai k un logon ko bhi batani parti hai

SS: acha aur joh nayi cheezein aap ko seekhne ko mil SShi hai us mein kia seekh SShe ho

CHW: yehi k jaise hum log logon k ghar ki joh hum log apne paas record leite hain toh aksar pehle maayein poochti thi k hamein is se kuch bhi ziadatar aurtein kehti thi k hum logon ka is mein koi faida nahi hai aur sirf aap log bachon k hawale se karne aate ho toh maa joh hai un ko bhi batatey hain un ki bhi vaccine hoti hai center mein joh hamla aurtein hoti hain who bhi apni vaccine karwa sakti hain is k elawa un ko joh hum log medicine deite hain joh kamzori k lehaas se folic acid hogayi ya phaSSsulphate toh who hum log un ko deite hain who log bhi kaafi mutmaain hain

SS: acha aur mujhe bataein k yeh joh hamal aur nauzaida bachon ki deakhbhaal mein aap ka kia kirdaar hai

CHW: us mein hum log yeh karte thy jaise aksar joh pehle matlab k pregnancy hoti hai toh aksar maayein kehti hain k hamein is cheez mein kuch samjh nahi aSSha hai jaise khaane k hawale se kamzor aaj kal ki matlab jaise k kaafi kamzor bhi hoti hain women us ko hum log phir batatey bhi hain k kis tarhan khaana matlab k apni giza ko pooSS karna chaiye kyun k who log kehti hain k hamein bhook nahi lagti phir hamein maidey mein jalan wageSS hoti hai hum se khaya nahi jaata toh us ko phir hum log guide karte hain aap log khaana ada ada matlab k timing bana dein poore days kit oh us mein ada ada jaise aap log apni giza leiti SShein poore din mein aur paani ka istamaal sahi tarhan SSkheingi toh inshaallah tabiate

bhi sahi SShegi in cheezon ko kaafi aurtein samjhti bhi hai aur dusSS yeh k un k bache jab hojaate hain toh kuch ko deakhbhaal ka itna nahi pata chalta k bacha hamaSS matlab k doodh nahi pee SSha ya bahir ka doodh deite hain upar ka koi aur doodh pila de toh un ko phir hum yeh batatey hain k 6 maa tak sirf aur sirf apna hi doodh deina chaiye bachon ko aur is k elawa koi matlab bahir ka doodh na dein toh ziada behtar hai

SS: acha aur kia kia un ki deakhbhaal k hawale se batati hain unhein

CHW: un ka jaise hum log jaate hain un ka weight wageSS tempeSSture leite hain toh who cheezein matlab k un ko batatey hain k bacha bilkul thek hai ya phir agar Allah na kare koi masla bhi hota hai bache k sath toh un ko phir hum log guide karte hain k aap in ko center le k jaayein agar ziada bache ki tabiat khaSSb hai toh un ko hum log batate hain aap log center ajaayein bache ko steam ki zarooSSt hoti hai toh hote hain ya phir doctor ko dikhaayein lekin jaise matlab k aksar maayein ziada se ziada bahir ki bhaari medicine hoti hai powder wali joh aaj kal dawaaiyaan hoti hai who de SShi hoti hain ya phir apne joh totkey kar SShi hoti hain k aa koi ghutti wageSS bana k ya anda wageSS matlab is tarhan ki phir un ko hum batatey hain k pehle doctor ko dikhaayein checkup karwaayein]phir apni yeh cheezein matlab k thoSS bohat totka ghar ka gharelu totke karein koi aisa na kare jis se bache ko faida nahi nuksaan ho

SS: acha aur kuch bataein aur joh aap ko lagta hai k hamein aur bhi mazeed batana chaiye

CHW: aur aksar jaise hamein k aurtein kehti hain k aap log acha toh kar SShe ho lekin hamare liye bhi kuch karein jaise delivery k hawale se toh kaafi community mein aisi aurtein hain joh aaj kal hospital mein matlab k bill afford nahi kar pa SShin kuch aisi familiyaan hoti hain toh who kehti hain matlab k aap log bohat acha toh kaam kar SShe ho lekin thoSS bohat maaon ki taSSf tawajju dein ya jaise un ki delivery k hawale se who matlab k chahti hain k yahan par joh hamare center mein aisa kuch system ho k delivery ka case wageSS hosake ya kahin aur refer karna ho toh is tarhan ka kuch agar kareinge toh aur ziada aurtein hamare sath coopeSSte kareingi

SS: sahi acha mujhe bataein aap ko kia lagta hai joh rozmaSS surveillance k kaam k hawale se joh aap hamal aur nauzaida bachon k paas aap jaate ho thek hai jaise k aap ne kaha na aap un ko maloomaat deite hain aur mazeed hum aur kia maloomaat farham karein

CHW: bachon k hawale se toh hum log unko har cheez matlab k weight tempeSSture ka jaise k mausam change hote rehte hain us k hawale se bachon ko hum batate hain medicine k hawale se bachon ko matlab k maaon ko batate hain k aap in k liye yeh yeh cheezein karein who cheezein faidemand hoti hain ya phir joh cheezein nuksaan deity hain bache ko un se maaon ko bolte hain k aap yeh cheezein bachon k liye na karein toh behtar hai

SS: aur kia maloomaat hamein farhaam karna chaiye

CHW: aur hamein jaise ek toh vaccine k hawale se hum log farhaam karte hain aur dusSS medicine k hawale se medicine jitni kam ho lekin bachon ki care wageSS who karti hain maaein aksar

SS: acha aur

CHW: aur un ko kehte hain apne bache ki vaccine mukammal karwaayein

SS: acha sahi acha joh joh cheezein aap ne bataei jaise k aap ne kaha tha k hum un ko cheezein bataeinge toh hamaSS bhi faida hoga un ka bhi faida hoga joh maaon aur 5 saal se kam umar bachon k hawale se joh aap unhein maloomaat de SShi hain aap ko lagta hai k aap ko munasib tSSining haasil hai

CHW: jee

SS: kis kisam ki tSSining aap ko haasil hai

CHW: hamare jaise yahan joh bhi hamare round wageSS hote hain toh un k darmiyan mein bhi hamein tSSining di jaati hai toh us mein kaafi kuch acha matlab k hamein jab bhi aa tSSining hoti hai toh us mein new new cheezein hamein batatey hain guide karte hain k aap ko in ko yeh cheezein batani chaiye toh us k hawale se tSSining hamari sahi hai jaise projects k hawale se hamaSS joh matlab k surveillance jaise round complete hojaata hai toh us k baad phir hamare matlab k hote rehte hain 6 month joh is tarhan round hamare timing hai 2 2 months k toh baad mein hamari training di jaati hai

SS: acha aur mazeed aap chahti hain aap k khayal mein aur mazeed training honi chaiye

CHW: who toh abhi jaise 6 month jab round complete hojaata hai toh who phir waise hi tSSining hamein deite hain who jaise agar 6 month k baad refresh agar kiya jaata hai who ziada kuch cheezein agar kabhi hum logon matlab k bhool jaate hain ya phir koi aur nayi cheezein hum ne wahan se field se aurton se sunni parti hai k hamare sath yeh yeh masaail ho SShe hain pregnancy k hawale se toh phir hum un logon ko batatey hain k yeh cheezein matlab k us k zariye trainining k zariye un ko bata sakte hain na toh waise matlab k 6 6 month k baad jaise refresh ek hota hai toh ziada behtar hai

SS: aur kia mazeed sunni parti hai aap ko

CHW: jaise pregnancies k hawale se aurtein hamein matlab k kaafi cases batati hain kuch k matlab k joh pehle shuro se bilkul sahi se case hote hain lekin end mein jab dikhaati hain ultSSsound ya kisi ki wahan pregnancy hoti hai phir kisi ki pregnancy nahi hoti kuch aur ki matlab k un ko jaise aa cyst wageSS paayi jaati hai SSsoli who phir un ko hum log batatey hain ya is k hawale se hum log kehte hain aurton ki jaise medicine wageSS hum log joh deite hain haalanke folic acid best hai lekin us k hawale se hum log joh who log chahti hain aurtein joh chahti hain k hamare delivery k hawale se kuch karein toh who agar hoga toh aur ziada hum log se community mein aurtein hamein aur ziada acha response deingi

SS: acha matlab yeh cheezein hongi toh mother ka response bhi aap k sath acha hoga aap k sath sahi se

CHW: aur ziada behtar tareeke se abhi acha kar rahi hain lekin aur ziada behtar tareeke se aur bhi kar sakti hain coopeSSte kareingi air acha

SS: acha sahi acha mujhe bataeiyega k aap ko kia lagta hai k joh sahulatkar aur nauzaida maaon k darmiyan sahulatkar aur nauzaida maaon k darmiyan aap ka kia kirdar tha jaise k unhein aap facilitate karte ho community mein ja kar batatey ho service apni batatey ho

CHW: jee

SS: us mein aap ka kia kirdar hai

CHW: joh jaise nauzaida maayein hoti hain toh un ko matlab k hum log achi giza k hawale se batatey hain aur hamare joh center k matlab k aise bhi projects hain jin ko hum log matlab k un aurton k hawale se batatey hain guide karte hain aur un aurton ko bhi bolte hain phir who matlab k wahan pe aati hain hamari team hain isi center ki team hai toh vital ki team who phir jaati hain nauzaida maaon ko aur nauzaida bache joh hai un k hawale se who phir matlab k aa un ko giza k hawale se deity hain toh who phir jaise maayein istamaal kar SShi hoti hain matlab k kaafi ache asraat bhi aaye hain maaon mein aur bachon mein bhi giza jaise hum log deite hain un ko achi matlab k guide kar sakein medicine jaise hogayi ya shashe wagehera hote hain

SS: aur kia matlab k aap kia deite ho unhein

CHW: who bhi hamare projects mein hain alag hain center k hi projet hain joh who kamzor maayein hoti hain un ki matlab k giza ki kaifiyat maaloom karti hain height wageSS un ki leiti hain phir us k hawale se matlab k followup un k karti hain aur un ki giza k hawale se un ko deite hain medicine wageSS matlab provide karte hain ta k un k phir aane wale bache bhi aa sehatmand hon

SS: acha toh yeh center ajaati hain khudse bhi

CHW: jee

SS: jab aap un ko batatey ho

CHW: jee jee hum log jaise gharon mein jaate hain toh un k matlab k un ko guide karte hain achi tarhan toh who ajaati hain center mein aati hain

SS: acha kabhi aisa hua k chalo aap jis particular area mein aap kaam kar SShe ho wahan se aap ko lagta hai k response aap ko acha mil SSha hai kabhi aisa k mother ka response aap k sath acha na ho

CHW: han aisa bhi hua hai aur matlab yeh toh har cheez ek normally ek cheez yeh hoti hai aurton ki kabhi kisi ka mood khaSSb hota hai kabhi kisi aur hawale se bhi toh ab hamein nahi pata hota hum log achanak un k ghar mein chale gaye who un ka gussa hum pe nikaal deity hain is tarhan bhi hua hai phir agli dafa jab hum jaate hain toh who phir batati hain k sorry maaf kar deina hum log us time pe gusse mein thy aap par gussa nikaal diya tha aisa bhi hota hai aur community mein aisi bhi familiyaan hain joh bilkul hi refuse hain joh matlab k kabhi matlab k yahan pe jaise kehti hain k hamaSS toh aap k center se link nahi hai hum wahan aate nahi hain toh hamare ghar mein bhi na aayein jaise un k husband wageSS manah karte hain family ko aurtein bhi is wajha se phir kuch hain aisi families waise ziadatar community mein kaafi aurtein aati hain

SS: toh refuse aa hone ki wajha kia hai

CHW: matlab achi familiyaan hain jaise matlab k aa maali haisiyat k hawale se k hum log apne attiya hospital mein apne bache ko dikha SShe hote hain toh hum nahi aate hum yahan aate bhi nahi hai aap

log bhi nahi aaya karo toh is tarhan ki joh families hain commercial area mein toh who sirf matlab k karti hain refuse aur itne who nahi karte

SS: aur yeh aa kitne gharon mein aisa hua hai joh refuse hain

CHW: aise matlab k hain leikin kuch hi families hain jaise poori community k hawale se toh itni nahi hai jaise agar 50 percent hamare sath achi hain toh us mein se 10 percent aisi families hain

SS: phir aap unhein batatey ho

CHW: jee hum log phir bhi batatey hain apni taSSf se poori koshish karte hain agar aurtein hoti hain toh un ko hum log pooSS samjhaate hain ya kisi gents ko unse hamari baat hoti hai aur kabhi aisa bhi hua hai matlab k admi hamari baaton se aate bhi hain phir who matlab k ijazat bhi deite hain apne ghar mein aane ki bhi aur apna record wageSS who bhi hamein deite hain hum apne un joh maloomaat hum log haasil karna chahte hain who hamein deite bhi hain kuch aisi bhi families hain joh maan jaati hain

SS: acha toh kabhi kaam k doSSan surveillance k kaam k doSSan koi aur dushwaari aayi refuses k elawa

CHW: refuses k elawa

SS: kaam mein koi dushwaari

CHW: kaam mein na toh kabhi matlab k dushwaari nahi huyi hai han waise abhi aa beech mein jaise matlab k jaise hum log jaSShe hain hote hain kuch ghar aise hote hain joh aa bachelor type k hote hain toh un area mein jaise matlab k worker do hoti hain toh safe hai lekin kabhi jaise matlab k pehle aisa hua tha shuro mein toh wahan pe matlab jaise k ek ek worker ja SShi thi toh who phir jaise buildings hain ab aa ek poori matlab k building hai us mein hamein jana hota hai who ek larki jaati hai toh phir thoSS unsafe lagta hai warna waise toh worker dono sath hoti hai toh phir itna matlab masla nahi hota

SS: toh kia koi alag alag particular area mein bheja jata hai ya

CHW: nahi nahi sath mein hi matlab k do pair hain dono matlab k sath mein jaate hain

SS: acha toh mushkil aap ko kahan aayi

CHW: mushkilaat toh itni matlab k koi waise nahi hai kabhi TAB hai TAB k hawale se jaise matlab k us mein koi masla hogaya koi matlab k aa kabhi battery ka kuch masla hota hai bas itna who toh itna nahi hota ek do dafa hua tha k TAB mein matlab k who kuch apne hi who circle wageSS matlab k us mein update wageSS kuch hoSSha tha phir who off hogaya tha phir who on nahi hua toh phir us k liye jaise center aana paSS phir jana paSS bas who ek hi dafa aisa scene hua matlab phir nahi hua

SS: acha aur koi aisa jaise k community mein aap ko jaise corona ki wajha se kabhi koi aisi mushkil aayi

CHW: aa shuro mein jaise abhi yeh corona ka matlab k cases ziada barh gaye thy toh hum log waise toh matlab k surveillance toh off tha lekin us k hawale se jab hamaSS kaam shuro hua tha shuro shuro mein toh us mein matlab k kuch families aisi thin k who matlab k itna hamein manah toh nahi kar SShi thin

lekin sirf yeh keh SShi thin k aap log humse darwaaze se hi maloomaat lo bs aur andar na aao is tarhan shuro mein hua tha aur who takreeban ek hafta phir who sab set hogaye

SS: acha shuro ka joh dar tha who ab khatam hogaya hai ya ab bhi hai

CHW: nahi ab itna nahi hai waise kuch kuch jaise matlab k families mein hota hai lekin ziadatar hote nahi waise bhi hum log mask wageSS waise bhi laga k jaate hain apne sath senitizer bhi SSkhte hain use karte hain toh who phir sahi hota hai kuch areas aise hain hamari matlab k community mein jaise mere areas mein churi wali joh matlab k hoti hain toh un k area mein jana parta hai toh who thoSS sa matlab k wahan thoSS difficult lagta hai lekin phir kaam hai karna parta hai wahan jana parta hai

SS: difficult kyun lagta hai wahan pe

CHW: wahan k matlab k ziadatar na ladies kuch jaise hoti hain aur is k elawa matlab k un ka mahaul itna who matlab itna saaf suthSS nahi hota aur har jagha pe un k logon se ziada kuttey hain (laugh) un k area mein matlab k wahan pe logon se ziada kuttey bhi hote hain aur dusSS yeh hota hai matlab k joh ghar wageSS hain bilkul matlab k jaise lakriyon k baney huye hote hain aur parde hote hain toh who ghandey wageSS is tarhan k toh thoSS us mein hota hai

SS: acha jahan kuttey hote hain jis area mein toh who area mein jaate ho

CHW: jee jaate hain aur aisa bhi hua hai ek dafa (laugh) field par jaise mujhe k hamla bhi kiya attack kiya tha

SS: kis ne attack kiya

CHW: kuttey ne lekin who matlab k thoSS sa touch hua tha phir mein ne apni vaccine mukammal karwa li thi

SS: aur yeh kab ki baat hai

CHW: yeh waise abhi is ko 3 saal hogaye hain

SS: 3 saal

CHW: who field k hi matlab k time pe hua tha lekin who phir usi time apne hamare joh supervisors bohat achi hain coopeSSte karte hain toh unhon ne vaccine mukammal karwak di haanlake itna koi matlab k ziada nahi tha ziada kuch itna nahi thoSS sa jaise touch hua tha mamooli sa lekin achi matlab k us k hawale se bhi meri poori hogayi thi mukammal

SS: toh aur koi aap ko lagta hai is k elawa koi aur mushkil aayi ho community mein

CHW: nahi community mein bas yehi matlab k cheezein kuch face huyi thi us k elawa jaise hum log apna kaam kar SShe hote hain ya hum log apni timing pe chale jaate hain acha hai matlab k field par who bhi jaate hain itni koi mushkilaat nahi hai

SS: acha aisi konsi cheezein hain joh aap k kaam k hawale se aap sunti hain joh aap ko acha lagta hai aap ki hausla afzai hoSShi hai aur aap chahte ho k aur mazeed hum kaam behtar se karein

CHW: jaise mein ne pehle bataya k kaafi achi familiyaan hamare sath coopeSSte karti hain toh kaafi matlab k aisi familiyaan hain kuch gents bhi un mein hote hain toh jaise hamare projects hain us mein hamare yahan center mein heart patient bachon k bhi hum log yahan par matlab k vaccine wageSS un ka bhi yahan treatment hota hai toh kaafi achi response hamein us k hawale se milta hai who familiyaan bohat ziada appreciate karti hain aur bolti hain k aap logon ka kaam mazeed aur aage barhe aur hum log chahte hain aap log aur mazeed ache se acha karein auSS b jaise community mein sirf aurtein yeh chahti hain ek toh jaise projects k hawale se har bache ki matlab k jaise alag alag kar SShe hote hain jaise kaali khaansi ka hogaya aur heart patient hogaye is k elawa bachon ki hum log vaccine mukammal karwa SShe hain jaise joh pehle matlab k itna nahi hoti thi aur joh matlab k nauzaida bache hote hain un ki hum log khauSSak un ko mauiya kar SShe hain har kisam k alag alag hamare paas projects hain toh ab maayein yeh chahti hain sirf aur sirf agar delivery ka bhi hojaaye nay a jaise ultSSsound ya kuch aisa thoSS bohat matlab k ek gynaecologist ho center mein joh hamein matlab k bata sake k aap ko yeh yeh cheezein hum ne ya hamal k douSSan toh who phir aur ziada mazeed achi hamare sath coopeSSte kareingi

SS: aap ko lagta hai yeh sab karne se agar hum un ko sahulat dein toh aap k kaam k kirdaar aur behtar hosakta hai

CHW: jee jee bohat ziada ache hojaayeinge muje matlab yehi lagta hai kaafi aurtein yehi kehti hain k agar yeh cheez hogi aap logon ka center bohat acha matlab k kafi aurton ko faida hoga kyun k aaj kal joh matlab k haalaat hain jis tarhan hamari matlab chal SShe hain government ki taSSf se toh matlab k hospitals k bills wageSS joh ziada aSShe hote hain us ki wajha se bhi hote hain kuch aisi bhi matlab k ziada community mein hai joh afford nahi kar pa SShi hoti hain toh who chahti hain k aisi sahulat yeh sahulat bhi kar lein toh ziada matlab k aur ziada acha hoga

SS: acha toh aap ko lagta hai k yeh sab karne se aap ki hausla afzai hogi community wale joh hain who khush honge

CHW: jee aur ziada appreciate kareinge

SS: acha mujhe bataiyega k aap k khayal mein konsi cheezein community mein CHW k kirdaar ko behtar bananey mein madad kar sakti hai aisi konsi cheez hum unhein dein jin se aap k kirdaar mein madad mil sake unhen

CHW: un ko jaise hum log medicine wageSS de SShe hain folic acid hogayi phaSSsulphate hai aur is k elawa abhi joh matlab k hum log un logon ko vaccine mukammal karwa SShe hain is se bhi kaafi behtar hai aur kaafi matlab k hum se mutmaayin bhi hain familiyaan aur agar is se aur nauzaida maaye hain bache hain un k hawale se aur ziada hum log kaam kareinge ya jaise achi si unko medicine wageSS dein guide kareinge main cheez ek hoti hai k joh aa kuch familiyaan aisi hoti hain joh kehti hain k pick n drop joh hum log pehle pehle shuro shuro mein center mein deite thy patient k bachon k hawale se who matlab k jaise aurtein chahti pehle aap logon ki pick n drop thi joh ab nahi hai center mein toh who kehti hain kuch aisi families hain joh kehti hain who aap log dobaSS se kar lein kyun k hamein dur aana jaana

hota hai aur ya phir apni aap log matlab k gaari mangwa lein kyun k hamaSS toh kaam jaise paidal ka hi hota hai hamein bhi gaari sirf pick n drop karti hain toh phir who kuch families aisi bhi hoti hain k hamein bache ko dikhana hai agar aap ki gaari hoti toh hamein aur asaani hojaati yeh cheezein kehti hain aur agar yeh hogi dobaSS se toh aur bhi ziada mazeed achi matlab k hamein lagta hai hamare liye bhi asaani hogi aur patient k liye bhi

SS: acha aap ko lagta hai aap k liye bhi asaani hogi patient k liye bhi

CHW: kyun k kaafi matlab k aurtein ye kehti hain na k pick n drop pehle aap ki hoti thi ab nahi hai auSS b hojaaye kuch aisi maayein hoti hain joh akeli hoti hain ghar mein joh a nahi sakti toh us k hawale se who phir ho

SS: agar yeh cheezein hon toh aap k kaam ko aur behtar bananey mein madad mil jaayegi acha yeh mujhe batayein aap ki SSaye mein hamal aur nauzaida bachon k hawale se konsi maloomaat unhein deini chaiye

CHW: khauSSak k hawale se aur dusSS un ko joh cheezein matlab k kia kia suit matlab k joh faide de sakti hain kuch aisi maayein hoti hain na joh aa kuch aisi cheezein khaa SShi hoti hain khauSSak mein toh un ko jaise blood ki kami bohat ziada hojaati hai ya phir un ka BP high rehta hai ziada hojaaye ya kabhi sugar kisi ki high hojaati hai toh us k hawale se hum un ko guide karte hain k aap yeh yeh cheezein istemaal na karein aur in cheezon se aap parhaiz joh matlab k kin cheezon se aap ko parhaiz karna chaiye kuch cheezein joh aap ko istamaal karni chaiye un ka ziada ziada istamaal karein apni giza mein

SS: acha aur kia kia cheezein matlab unhein hamein maloomaat deini chaiye

CHW: aaron k hawale se k hamein matlab k kis tarhan se apne bache ko who guide karein matlab k un ki deakhbhaal kar sakein aur deakhbhaal k hawale se aur medicine joh mukammal matlab k apni who karti hain

SS: acha mujhe batayeiga k koi aisi cheez joh aap batana chahti ho jis se aap k kaam mein aur behtar hosake surveillance k kaam k baare mein kuch batana chah SShi hon aap

CHW: surveillance k kaam k baare mein yehi cheezein abhi matlab k joh batai k aa pick n drop ka aur dusSS yeh medicine joh hum log de SShe hote hain aurton ko who ziada matlab k hum log de toh SShe hain apni taSSf se aur aati bhi hain kaafi aurtein families aati bhi hain bas who log kuch matlab k who chahte hain k joh matlab k delivery case k hawale e agar yeh hojaaye toh who ziada ya koi matlab k aisi doctor ho joh hamein matlab k apne masaail k baare mein bata sakein pehle hi toh who aur ziada behtar hosakta hai

SS: aur kuch batana chaaheingi aap surveillance k hawale se

CHW: aur yehi k hamaSS aa achi matlab k hamaSS kaam joh chal SSha hai who acha hai behtar hai aur hum log apni taSSf se matlab k pooSS behtar se behtar kar SShe hain aur kaafi log bhi hum se bohat ziada mutaasir bhi hain kuch jaise families pehle refuse thin who abhi kaafi who hum logon se matlab mutmaayin bhi hain aur agar refusal bhi maan jaate hain aate hain vaccine wageSS who mukammal

karwa SShe aur apne bachon ki aur joh haamla aurtein hain who apni bhi matlab k medicine aur is k elawa joh tikke hain vaccine hain who mukammal karwa SShe hain

SS: acha aur kuch joh aap k demag mein aap k zehan mein chal SSha ho joh aap batana chah SShi hon joh aap k dil mein hon

CHW: demag mein aa yehi cheez joh humein jaise aurtein kehti hain k jaise hamein ache se acha matlab k hamal k doSSan hi joh matlab k cheezein hamare liye nuksaandey hain who hamein batai jaaye hamari koi report matlab k joh sahi nahi hoti ya ultSSsound ka joh matlab k us mein joh masaail aate hain who hamein guide karne wala koi ho joh hum log um ko bata SShe hote hain lekin kabhi kabaaar kuch aisi cheezein hoti hain joh ek doctor hi acha hamein bata sakta hai toh gynaecologist k hawale se agar yeh cheez hoti hai toh mein yeh sochti hun k aur ziada aurtein bhi matlab k is se khush bhi hongi aur un ki bhi matlab k mashkilaat asaan hosakti hai hamare through matlab k center mein

SS: aur kuch joh aap batana chah SShi hon

CHW: aa jaise pehle hum logon ka ek project tha kaafi time pehle a MANISA ka jis mein hum log zachki k hawale se joh kaam kar SShe thy us mein hum log aurton ko aa joh medicine wageSS de SShe thy joh matlab k kamzor maayein un ko hum yahan center mein bhi le k aate thy toh un ki doctor khud matlab k treatment karti thin gynaecologist thin toh who aurtein chahti hain k is tarhan ka bhi hamare sath hojaaye na toh hamal k doSSan hi hamein kaafi matlab k acha matlab k response de sakti hain

SS: aap ko lagta hai k yeh cheez matlan hongi toh aage bhi aap k kaam mein behtari

CHW: aur bhi behtari ho sakti hai

SS: sahi aur kuch

CHW: aur nahi

SS: bas chalein shukriya…

Date: 05112020

SS: acha NAME aap ki umar kitni hai

CHW: 28

SS: aur aap ki taleem

CHW: meri graduate

SS: graduate kis mein

CHW: mein ne sociology mein kiya hai

SS: acha aap k kaam ka tajurba kitne saal ka

CHW: mera waise toh mera joh hai 16 years hai lekin joh NWSR mein hun mujhe 8 years hogaye hain

SS: Aur surveillance mein aap ko kitne saal huye

CHW: is mein mujhe 6 years ho chuke

SS: acha SS mujhe bataiyega k surveillance k kaam k hawale se aap kaisa mehsoos karti hain

CHW: surveillance ka kaam joh hai who hum log ko joh hai who ek hi ghar mein baar baar visit karna hota hai matlab 2 months k gap se pehle toh hamara yeh tha k 3 maheeney ka gap deite thy lekin hum ab 2 month pe agaye hain toh surveillance ka kaam bohat acha hai hamari logon se bohat ziada understanding hogayi hai unhein pata hai hum aate hain har 2 2 month baad aur who data hamein comfortable araam se de deite hain hum par bharosa karte hain toh community mein hamein aisa kuch issue nahi hua kabhi kaam k hawale se

SS: acha kaam k hawale se koi issue nahi

CHW: jee aisa kabhi nahi hua

SS: toh us mein aap kaisa mehsoos karti hain

CHW: DSS mein bohat comfortable hun mujhe yahan par itna arsa hogaya hai yahan k logon ko hum achi tarhan jaan gaye hain aur log bhi hamein achi tarhan pehchaan gaye hain hum pe bharosa karte hain trust karte hain even apna personal data hamein easily de deite hain toh abhi tak hamare sath aisa issues matlab kuch aisa issues nahi aaya

SS: toh aap gharon mein jaate ho hamal ya nauzaida bachon ki deakhbhaal mein aap ka kia kirdaar hai

CHW: hum toh joh hai just sirf data collect karte hain na hum toh sirf data collect hain hum se un ko koi faida nahi hai logon ki yehi shikaayat hoti hai aap log aate ho itni informations leite ho hamare personal

ghar k hawale se bachon k hawale se NIC number tak lete hain mobile number bhi lekin hamein in sab cheezon se kia benefit toh hum un ki counseling karte hain k yeh sab data hamare paas hoga usi par hi aage hum research karte hain aap k elaake mein kia koi aisi beemari jis se bachon mein ziada amwaat horahi hain pregnancies yeh sab cheezein isi liye li jaati hai ta k hamare centers par joh doctors beithe hain jab aap log hamare center aate ho bachon ka treatment wagera bhi karte hain ek newborn ki team hai joh bachon ko check karti hai aur joh bacha sick milta hai toh hum refer karte hain phir hamare doctor us bache ko check kartehain aage refer bhi karte hain toh yeh sab cheezein hum ahista ahista jab hum un ki conselling karte hain toh who samjh jaate hain kuch log toh hai who still refuse karte hain k hamara joh hai is se acha humein panel mil raha hai hum wahan pe jaate hain aap k center nahi aate treatment k liye

SS: acha aap ne kaha tha k kehte hain baar baar ajaate ho

CHW: jee

SS: sahi hai na toh matlab who kis wajha se keh rahe hai k baar baar ajaate ho

CHW: kyun k deakhein ek sirf hamari hi team joh hai who kaam nahi kar rahi baaki aur bohat saare projects hain joh yahan pe chal rahe hain suppose ek bache ki death hojaati hai toh pehle RSV ki team hai who jaati hain us ka sample leiti hain us k baad agar hum ja rahe hain death ka poochne k liye us k baad wahan se ek aur team jaati hai others koi project toh log bhi irritate hojaate hain k itni teams baar baar a kar hum se detail leiti hain baar baar poochti hain toh is wajha se bhi hain na log thora irritate karte hain yeh hai k un ki conselling karni parti hai

SS: acha toh yeh koi particular koi jagha hai koi area hai jahan pe aap ko lagta hai k baar baar jaate hain irritate hojaate hain kuch aisi jagaein joh aap ko lagta hai log irritate horahe hain

CHW: aisi koi khaas jagha nahi hai poora matlab surveillance k andar ek ghar mein suppose death hojaati hai toh hum us k ghar mein jaayeinge toh who irritate huye honge khaas area nahi hai poora matlab elaake mein hi is tarhan k hamein milte rehte hain mile jule comments kuch log joh hain who hamari baat ko samjh jaate hain hum se coorperate karte hain kuch log joh hain still refuse kar deite hain who kehte hain k hamein aga khan se koi facilities nahi chaiye hum aap logon ko na data bataeinge aur na aap humse kuch poochein toh is tarhan ki bhi cheezein milti hain

SS: koshish aap ki hoti hai k refuse ko mana lein

CHW: bilkul hoti hai hamari jitni hum koshish end time tak karte hain lekin zahir si baat hai k hum kisi se zabardasti data nahi leite kisi se zabardasti bache ko check nahi kar sakte jab tak us k ghar wale us k parents permission na dein who hum pe trust or jab tak poora data na dein toh kisi pe hum zor zabardasti nahi karte hum un se yehi kehte hain aap khushi se jitna aap hamare sath cooperate kar sakti hain

SS: acha mujhe bataein k bachon ki deakhbhaal k hawale se aap unhein kia batate hain

CHW: bachon ki deakhbhaal yeh hai k hamari joh team hai newborn ki team hai sab se pehle hum pregnancies ko apne paas register karte hain pregnant women ko jab who hamare paas registered hojaati hain toh jab tak who delivered nahi hojaati tab tak hamare paas pregnant show hoti hain toh us ka jab outcome ajaata hai bache ko check karte hain us k andar agar koi danger sign milta hai toh hum un ki conselling karte hain k aap is ko le kar foran refer karte hain jab who maa nahi raazi hoti hai k hum yehi conselling kar rahe hote hain k aap k bache ki tabiat thek nahi hai ya heart beat ziada hai joh joh hum thermometer se hum fever bache ka check karte hain RR check karte hain weight karte hain bache ka weight kam hai toh breastfeeding k hawale se maa ko batate hain k doodh kis tarhan se inhein baar baar pilaana hai yeh sab cheezein joh hain yeh mothers se hamari team joh hai who counseling kar rahi hoti hain

SS: acha chalein yeh toh bataya tha aap ne k joh joh aap ne cheezein bataei hain who counseling se related batai duCHW mujhe bataein k aap ko kia lagta hai rozmara surveillance k joh kaam hai kaam k elawa zachki aur nauzaida bache k hawale se maloomaat unhein farhaam karna chaiye

CHW: bilkul karna chaiye hum karte bhi hain pehle karte bhi thy aur abhi bhi karte hain aur karni chaiye

SS: acha kis hawale se karni chaiye

CHW: maa ko batana chaiye k agar us k chote chote sign bata dein fever hai toh who kia kare agar us k paaon pe swelling horahi hai toh kia kare khana kis tarhan se lein folic acid kia kaam karti hai pharasulphate kia kaam karti hai is tarhan se choti choti cheezon k baare mein counseling karte rehte hain

SS: aur kia kia karte hain counseling

CHW: bas counseling mein antenatal care k andar joh joh cheezein aati hain who sab hum un ki counseling karte hain toh waqfan hum batatey rehte hain toh yeh yeh sab aap karein followup pe jaayein check up karwaayein end time tak agar yeh sign ho toh doctor se rujoo karein is tarhan ki counsel hai aur jab delivery hojaati hai first joh hai bache ko feed deina hai upar ki koi giza nahi deini koi cheez nahi deini gutti wagera yeh sab cheezon k baare mein unhein guide karte hain batatey hain

SS: aur koi maloomaat farhaam karna chaiye

CHW: aur mazeed matlab yeh hai k maa aur bache ki sehat k hawale se agar hai k hum joh counseling karte hain agar aur improvement hojaaye hamare workers ki trainings ho k aap field pe jaayein agar har 6 months k baad antenatal k upar ya bachon ki deakhbhaal k upar koi refreshment hona chaiye workers k knowledge mein izaafa hoga field mein ja k kis tarhan se maa aur bache ki counseling kar sakein

SS: acha yeh refreshment aap bata rahi hain training honi chaiye

CHW: training honi chaiye workers ki

SS: acha toh aap k khayal mein joh joh aap ne maaon ko nauzaida bachon ko aur joh 5 saal se kam umar bache toh joh joh maloomaat aap ne farhaam ki hain toh aap ko munasib training haasil hai

CHW: jee jee bilkul hai hamara refreshment hota hai jab hamara round start hota hai us mein hamari joh supervisor hain coordinators hain aati hain refreshment deity hain health k hawale se bhi batati hain field mein jaayein aap ko kis tareeke se matlab community mein kis tarhan se apna behavior rakhna hai yeh sab cheezein joh hain who batai jaati hai

SS: aur kin kin cheezon par training di jaaye aap k khayal mein

CHW: vaccination k upar bhi bata sakte hain aur yeh joh hamari danger sign hain bache k aur maa k hawale se in cheezon par agar hum joh hai refreshment rakhein waise toh hamari site RAs hain wohi deity hain agar jaise coordinator a kar koi ek point le lein matlab jaise breast feeding par le liya ya vaccination le liya us k upar specific session agar rakha jaaye toh yeh k knowledge mein workers k knowledge mein izaafa hoga hum se hat k hoga phir thora refresh hoga workers ka bhi ek routine ban jaati hai subha jaana hai aan ahai data deina hai RA ko phir chale jana hai agar is se hat k agar thora hamara hoga mind bhi thora refresh hoga aur knowledge mein izafa hoga workers bhi interest leingi in sab cheezon pe

SS: acha is se aap k surveillance k kaam mein aur behtari aayegi

CHW: aur behtari aayegi improvement aayegi

SS: acha toh aisa kuch hua hai kabhi maloomaat aap ne di ho maaon ko kuch aisa hai joh maayein joh keh rai hoti hain kia faida aap joh bata rahe ho maloomaat deine k hawale se

CHW: haan aisa hota hai ziadatar community mein females joh hoti hain pregnant women ka yeh concern hota hai aap log deliveries bhi free karwaayein aap toh sirf a k poochte ho phir chale jaate ho aur humain yeh sab cheezein bata deite ho agar hamare sath koi emergency ho jaati hai hum kahan jaayein aap k aga khan mein bache ka elaaj hota hai joh k panadol ek simple sa joh government hospital mein bhi de diya jaata hai who aap logon k center mein deite hain toh hum kyun aayein yeh bhi logon k hain concept toh agar aap log ultrasound wagera karein deliveries karein toh is se yeh hoga k community mein joh log hain data aur ache tareeke se deinge k un ko pata hoga k hamare aane se faida hota hai aur who apni pregnancy wagera bhi hamein khud se bataeingi kyun k abhi joh hain log chupaate hain who kehte hain kia faida kyun k yeh hamein tang kareingi baar baar

SS: acha aise kitne ghar hain joh aap ko lagta hai k kyun log chupaate hain

CHW: yeh khaas taur pe joh cattle colony hai labor square hain yahan k log thore well educated hai who kehte hain k hamein aap se ziada knowledge hai aur aap k hospitals se koi faida nahi yeh toh chota sa hai matlab is se acha hamara aga khan ka khud ka panel hai aur hum kyun aap ko yeh sab cheezein bataein hum apna time zaaya karein

SS: acha aur joh chupaate hain who

CHW: cattle colony labor square yahan pe joh log hain yehi reason hain matlab hamein batane ka kia faida hamein toh koi benefits nahi hai na sirf aap aate hain likhte hain parh k sab research ki cheezein aap ki hai yeh who is wajha se thora data nahi deite hum log joh hin ek ghar mein jaate hain sab se pehle

toh MAWRA 49 years joh hain married women ko register karte hain un k 5 saal se chote bache hain un ko register karte hain is k elawa un ka NIC mobile number sab cheezein kar leite hain aur vaccination card joh hai bache ka check karte hain is k elawa hamara koi aur aisa data nahi married women or 5 saal k chote bache

SS: kabhi aisa laga k direct data deine se inkaar kar diya ho

CHW: hota hai aisa bhi hota hai refusal rate bhi hain cattle or labor mein ziada hai refusal wahan k joh log hain who nahi batatey kam batatey hain hamein counseling karni parti hai mehnat karni parti hai

SS: acha aur kuch joh aap ko aisa laga ho k aap ne koshish ki unhein mananey ki un ki taraf se kia response tha

CHW: refusal ki taraf se agar hum chahte hain refusal ki counseling karte hain kaafi refusal joh hain who agree hojaate hain baat ko understand karte hain samjhte hain aur data de deite hain k aaya unhon ne itna mehnat ki hai toh phir kuch log aise hote hain yeh response hota hai k deakhein aap toh aate hain likh k chale jaate hain mera bacha agar beemar hai toh aap k yahan le k bhi jaayeinge toh aap refer kar deinge Jinnah hospital mein aap kisi ache hospital mein toh refer nahi karoge na aap mere bache ka sahi se elaaj toh nahi karoge aur matlab koi free facilitate hamein gaari douge community mein log ziadatar hamein har cheez free mile aura cha sa hamein aga khan mein dein yeh log panel wagera is tarhan k logon ki yeh khuwaishaat hai yahan pe mere bache ka elaaj na ho achi jagha ho toh phir yehi hai is tarhan k response milte hain

SS: is tarhan k joh log hote hain aap ko lagta hai k aap k kaam mein dushwaari a rahi ho

CHW: kaam mein dushwari aati hai kyun k zahir si baat hai ek hum ghar mein ja rahe hain ek maa ka bacha beemaar hai us ko toh us time pe ache us ki care ki zaroorat hai na k mere bache ka deakhbhaal yeh log toh aayeinge sirf likheinge chote se center mein refer kar deinge wahan se who log bache ko Jinnah bheij deinge ziadatar yehi hota hai k doctors deite hi kia hain panadol k elaawa kuch nahi deite toh community mein yahan par joh hai ziadatar yehi response hai itni dur paidal chal k aap k center aate hain hamein achi facilities nahi milti hai jab aap k center mein aate hain

SS: sahi toh surveillance k kaam k doraan koi rukaawatein joh aayi hon

CHW: surveillance k kaam k doraan aisi koi rukaawat nahi aayi hai 6 saal k andar aisa feel nahi hua

SS: surveillance mein community ki baat horahi hai koi bhi aisi rukaawat ya mushkil ka saamna nahi karna para

CHW: mujhe nahi hua haan yeh hai k dog issue ziada hote hain field site pe jaate hain toh dog issue hain wahan pe workers kuch elaakon mein jaati hain yahan pe doggyi hain is k elaawa to aise kuch mujhe nahi aisa kuch

SS: acha mujhe bataiyega k corona ki wajha se kuch aise masle huye hain community mein

CHW: corona k hawale se yeh hai joh hum log newborn ki team hai na un ko refusals milte hain k bahir side se arahe hain who kehte hain bache ko check nahi karwa rahe corona ki wajha se k aap bahir se a rahi ho hosakta hai aap k sath corona ho jaraseem ho hamare bache ko lag jaayeinge mujhe aise ek do mile hain case community mein unhon ne refuse kiya

SS: acha yeh kab ki baat hai

CHW: yeh jab corona start hua tha starting ki baat hai

SS: starting ki baat hai

CHW: jee

SS: acha aur kuch joh aap ko laga ho koi aur masaail jaise aap ne kaha na corona ki wajha se ghar mein aane nahi deite

CHW: jee ghar mein aane nahi deite newborn ki team joh jaise newborn ki team jaati hain followup k liye un ko unhon ne manah kiya tha do cases aise aaye thy k corona ki wajha se who maa dar rahi thi k bahir se aap aayi ho toh koi germs mere bache ko lag jaayeinge toh is wajha se unhon ne refuse kiya tha

SS: acha pehle dar tha ab abhi hain

CHW: ab aisa kuch bhi nahi hai ab normal hai sab toh asaani se kar leite hain followup bhi kar leite hain data bhi log de rahe hain abhi corona ki wajha se koi khauf nahi balke khud haath milaate hain hum se hum jab bolte bhi hain toh who kehte hain kuch nahi hai ab sab khatam hogaya

SS: acha mujhe yeh bataiyega aap ko kia lagta hai k sahulatkaar or nauzaida maaon k darmiyaan aap kia kirdaar ada karti hain jaise k aap ne kaha facilitate bhi karti hain bataya aap ne k center pe aayein bache ko dikhaayein services bhi aap deite hain us mein aap ka kia kirdaar hai

CHW: hum toh just refer kar deite hain aage joh hai who doctor khud apne checkup karte hain aage humara kaam hai sirf refer kar deina agar bacha hamein sick milta hai toh hum un ko yehi kehte hain k aap foran kisi bhi kareebi hospital mein doctor k paas aap chale jaayein bache ko le kar

SS: toh who ajaati hain koi problem toh nahi hoti

CHW: yahan pe joh hai na pick n drop nahi hai bhans colony mein hamare paas baaki jaise pehle mein RG mein thi RG mein pick n drop thi toh wahan pe joh hai na log ziada acha response deite thy bohat acha aur jab hum jaate thy log respect bhi karte thy hamein baqaida ghar pe bhi bithate thy data hamein sahi se araam se deite thy kyun k wahan par facilities ziada hai deliveries hai ultrasound hai bachon ki vaccination pick n drop hai yeh sab cheezein hain is wajha se hamein ziada acha response RG mein milta tha mein bhans colony mein aayi hun yahan pe ek bhi cheezein nahi hain yahan k community k log thora irritate hote hain irritate is wajha se bhi hote hain k yahan pe unhein facilities nahi milti hamein koi faida toh nahi hai aap k yahan aane se agar mein pregnant hun aur mujhe koi complication hai kia karogi aap toh sirf refer karogi na thori si counseling karogi yeh kar lein aap yeh kar lein is k elawa na aap mujhe pick n drop de rahi ho na kisi ache hospital mein refer kar rahi ho na koi treatment karwa rahi ho toh

mein kyun aap ko apna time itna dun aap ko apne baare mein saari history bataon mujhe toh is se kia faida hai mujhe is se koi faida nahi hai is wajha se aur yehi cheez RG mein thi toh RG mein joh hai who log hamein pakar pakar k khud bulaate thy agar hum kisi aur ghar mein gaye toh unhein pata bhi chal jaata tha k aga khan ki team aayi hai toh who kehte thy k mein pregnant hun hamara naam likho khud bula bula k likhwaate thy kyun k wahan pe facilities thi

SS: aura b facilities nahi hai

CHW: RG ki baat kar rahi hun wahan par toh hain abhi bhi

SS: toh mother ko problem hoti hai yahan a nahi sakte hain pick n drop ka masla hai

CHW: jee pick n drop ka masla hai

SS: toh yeh surveillance k doraan baat horahi hai joh aap bata rahe ho

CHW: jee surveillance k andar hamare paas pick n drop nahi hai na koi sick baby hamein milta hai toh us ko refer karte bhi hain toh hamare paas gaari nahi hai joh gaari mein hum apne sath bache ko le k aayein aur phir usay hospital le k jaayein wapis ghar chor aayein is tarhan ki facilities hamare bhans colony mein nahi hai RG mein hai

SS: acha mujhe bataiyega k aisi konsi cheezein hain joh aap k kaam k hawale se joh k aap sunte ho aap ko acha lagta hai aur aap ki hausla afzai horahi hai aur aap kehte ho k aur kaam ache se hona chaiye

CHW: agar koi hum kaam karte hain field site pe aur hamari coordinator hamein appreciate karte hain acha kaam kiya hai toh us se hamari hausla afzai hoti hai hum khush hote hain aur mazeed acha karne ki koshish karte hain

SS: aur community k hawale se unse aap apne baare mein kia sunti hain

CHW: haan kuch log hain joh acha response deite hain aur who hamein data bhi batatey hain aisa nahi hai k sab aise hi hain sab joh hain who irritate hote hain data nahi deite yehi kehte hain hamein kia faida kuch log joh hain who ache se bula k bithate hain batatey bhi hain aur hamare baare mein ache comments bhi hamein sunne ko milte hain jaise field sites pe hamari workers aati hain aga khan ki larkiyan aayin thi unhon ne yeh bataya k vaccination kitni lagti hai BCG kis din lagti hai hamare center ajao is tarhan se bhi matlab vaccine k hawale se bhi log humse poochte hain aur unhein batatey hain logon ki knowledge mein izaafa hota hai toh kuch log humse happy bhi hote hain

SS: acha aap ko lagta hai k haan meri yeh housla afzai horahi hai is se aap ka kaam behtar hota hai

CHW: behtar hota hai

SS: sahi aap k khayal mein konsi aisi cheezein community mein CHW k kirdaar ko behtar banaey mein madad kar sakti hai aisi konsi cheezein hain joh community mein dein toh CHW k kaam mein asaani hojaaye

CHW: community mein ek toh hamare paas jaise gaari ka issue hota hai har larki yehi kehti hai k gaari agar hamare sath ho kuch area aise hote hain jahan par jab mein field pe jaati hun mujhe aisa lagta hai k mere sath driver andar tak chale male ho joh is elaake mein andar tak mere sath jaaye toh mujhe thora yeh hoga k mere sath koi male hai kyun k female ka akele area mein jaana aur kuch area aise hote hain jahan par do larkiyan bhi agar sath jaaye toh unhein thora khauf hota hai k yeh area aisa hai andar kuch hamare yahan buildings wagera hain upar buildings mein andhera hota hai andar jaate hain toh us mein yeh ho agar ya toh koi driver ho koi male agar hamare sath ho jab hum aise areas mein jaayein toh larkiyon k sath us area mein ja k field visit karwaayein

SS: chalein yeh cheezein aap ne batai k gaariyon ka aap ko hai driver sath mein chalein community mein aap k khayal mein aisi konsi cheezein joh aap k kirdaar ko behtar bananey mein madad kar sakti hai

CHW: hamare paas jaise blood pressure ki ek machine hon agar ek maa hamein bol rahi hai mera BP high hai aap mera BP check kar sakti ho kyun k BP operator aisa hai larkiyon ko agar ek baar training di jaaye toh who kar leingi aur hamae paas bhi ho BP operator ho sugar ki machine ho yeh choti choti cheezein agar hongi is se bhi hamari joh community hai who hamein acha response deingi k chalo agar aga khan ki team aati hai hamara BP check hojaata hai wazan hojaata hai maa ka weight machine hon hamare paas yeh agar choti choti 3 4 cheezein bhi hon is se bhi bohat effect parega ta k who log hamein aur ache se response bhi deinge fark aayeiga

SS: matlab yeh sab cheezein aap provide kareingi toh

CHW: agar yeh cheezein provide karein toh yeh community mein agar use kareinge toh log hamein acha response deinge

SS: acha aap ki raaye mein gharon pe aap jaate ho hamal aur nauzaida bachon k hawale se aur konsi unhein maloomaat deini chaiye

CHW: mere khayal se joh hum maloomaat de rahe hain agar hum yehi sahi tareeke se unhein maloomaat dein aur us par amal karein yeh bhi bohat hai

SS: Kis tarhan

CHW: matlab bache ka kis tarhan se khayal rakhna hai breastfeed k kia kia faide hain bache ka weight karwaana hai bache ki heart beat toh teiz nahi hai bache ka fever yeh sab cheezein joh hum un ko bata sakte hain toh agar maa ko agar sahi se samjh ajaaye aur maa us par amal kare toh is se bhi bohat fark parega

SS: aur kuch joh unhein maloomaat deini chaiye joh unhein pata hi na hon aur who aap un ko de rahe ho information aisa kuch

CHW: aisa vaccination k hawale se polio k hawale se un ko batatey hain aur kuch amal bhi karte hain kuch refuse karte hain vaccine k hawale se yeh hamare baron mein manah kiya hua hai vaccine nahi karwaate is k yeh nuksaanaat hain agar is k baare mein bhi waise toh hamari teams counseling karti hai aur mazeed hamari counseling achi ho jaaye toh is se fark parega

SS: aap k paas koi aur raaye ya khayalaat hain joh aap apne surveillance k baare mein batana chahti hain

CHW: surveillance k baare mein yeh hai k pehle se hamara joh kaam tha pehle written work tha hamara kaam tab pe agaya hai toh hamara kaam bohat improve hua hai bohat acha hua hai is k elawa toh koi aisa nahi

SS: kuch bhi joh aap batana chahti hon ta k aap k kaam mein aur behtari hosake

CHW: yehi joh mein ne bataya refreshment ho workers ki hausla afzai ho coordinator aayein un ko appreciate karein is se bhi bohat fark parega

SS: acha aur kuch bas chailein shukriya….

BH-IDI 04

SS: acha zakia aap mujhe bataayeingi k aap ki umar kitni hai

ZA: meri 30

SS: 30 acha aur aap kitna parhi huyi hain

ZA: mein ne inter kiya hua hai

SS: inter kiya hua hai aur aap ki shadi ko kitna arsa hogaya

ZA jee 6 saal huye hain

SS: 6 saal huye hain kitne bache hain aap k

ZA: 3

SS: 3 bache hain sab se chota bacha kitna

ZA: woh 23 month ka hai

SS: acha thek hai toh zakia ghar pe koi ghar k kaam k elawa koi aur kaam karti hain

ZA: nahi

SS: kuch nahi karti hain acha mein na aap se joh hamari health worker ghar par aati hain us k hawale se sawal karongi toh mujhe yeh bata sakti hain k woh aap k ghar pe kitne arse baad aati hain

ZA maheenay mein ajaati hain ek dafa do dafa

SS: acha jab woh aati hain kis kisam ka kaam sar anjaam deity hain

ZAa: bachon ka naam poochna un k baare mein poochna unki tabiat k baare mein poochna

SS: hmm

ZA: aur ghar ka saara bio data leina

SS: acha us mein kia kia matlab

ZA: us mein yeh poochti hain ghar mein kitne afraad hain

SS: hmm

ZA: gair shadi shuda kitne hain shadi shuda kitne hain bachon ka poochna bas

SS: acha

ZA:polio pilaaya hai nahi pilaaya hai injection lagwaaye nahi lagwaaye

SS: hmm

ZA: card check karti hain injections k

SS: hmm aur aap k hawale se koi sawal karti hain

ZA nahi bas yeh poochti hain tabiat sahi hai bas khatam

SS: acha aap k sehat k hawale se nahi poochti

ZA: nahi bachon k baare mein poocha bas bachon ka bio data liya naam liya shohar ka naam poocha ghar mein kitne kunwaare hain kitne nahi bas yehi poocha bas

SS: bas yehi poocha acha aap hamal se hain nahi hai is tarhan k koi sawal

ZA: nahi is tarhan ka koi sawal nahi

SS: acha kabhi koi bacha zaaya hua ho kuch is tarhan poocha

ZA: nahi nahi nahi who bachon k lehaaz se hi aati hain

SS: achaa acha mujhe yeh bataayein k aap kaisa mehsoos karti hain jab aap k ghar aati hain aur yeh saari cheezein

ZA: hum logon ne kabhi is cheez ka mind nahi kiya bachon k baare mein poochti hain bachon k baare mein bata deite hain

SS: thek hai toh aap unhein kyun bataati hain ek a raha hai aap k ghar mein aise aap se bachon k baare mein pooch rahe

ZA woh hum jab hospital jaate hain toh hospital k tehat aati hain toh phir us ki wajha se bata deite hain

SS: toh aap ko un pe bharosa hai

ZA: haan

SS: acha kia wajha hai

ZA: kyun k bahir gaari aati hai team work aati hai aur ziadatar joh ladies aati hain woh jaan pehchan ki baaji yahin hai mashaallah ghar in ka hai parlor chalaati hain

SS: hmm

SS: toh mashaallah phir sab in ki jaanne wali hain jitni bhi aap k paas amla hai takreeban baaji ka jaanne wale hain

SS: acha toh is liye aap un ko jaanti hain

ZA: haan sab yani jitna amla aap ka yahan pe aata hai woh sab baaji ko jaante hain kyun k baaji ka parlor mashaallah 20 saal se chal raha hai toh woh sab k sab baaji ko jaante hain toh hum gair banda toh andar aata nahi hai

SS: hmm

ZA: toh phir yeh hai k aaj anjaan chehrey aaye hain lekin jitney bhi aate hain sab baaji ko jaante hain takreeban kyn k baaji yahan ki rehaaishi parlor chalana

SS: hmm

ZA: toh phir sab

SS: acha matlab k aap un ko is liye bata deity hain kyun k aap k mauhalley ki hain

ZA: haan jaan pehchaan hai aur phir baaji jaanti hain sab yani ghar waale jaante hain

SS: aap k ghar waale jaante hain is wajha se

ZA haan anjaan bando ko koi detail nahi deita hai ek toh woh team work aati hain

SS: hmm

ZA: phir baaji un ko jaanti hain toh phir is lehaaz se bata deite hain

SS: acha un ka rawaaiya kaisa hota hai aap k sath

ZA: jab jaanne wali hain toh ziadatar acha hota hai

SS: hmm kis tarhan se baat karti hai woh

ZA: achi baat karti hain jaise tareeke se baat hai salam kiya andar aaye bethe detail li

SS: hmm acha aur us doraan jab yeh pooch rahi hoti hain k kitne bache hain bachon ko teeka lagaya toh koi maloomaat bhi de rahi hoti hain woh aap ko

ZA: nahi yeh keh deity hain k injection 23 maheeney tak poore karne hain yeh is tareeke se poore karne hain agar koi bacha bimaar hai toh us ki report pooch leiti hain k kis wajha se bimaar hai

SS: hmm

ZA: meri choti beti thori bimaar rahi hai us ka pooch leiti hain phir us ko bol deti hai k agar tabiat sahi nahi hai toh us ko aga khan le jaaiyega is tareeke se guideness de deity hai

SS: yeh bata deity hain center le aao agar tabiat thek nahi hai

ZA hmm

SS: acha toh aap mujhe bataoge k aakhari dafa hamal se huye thy aap toh matlab jab pregnant huyi aap toh CHW ne koi kirdaar ada kiya

ZA: koi is tarhan ka hamare ghar mein kabhi nahi aaya

SS: koi nahi aaya

ZA: kabhi mein agar idhar bhi gayi mein ne shuro mein bachi ko TKB k injection lagte hain k bache ko demaag ka jhatka na lage kuch na lage woh mein ne aga khan se lagwaaye thy baad emin jab dusri bachi huyi

SS: hmm

ZA: toh doctor ne pehle hi manah kar diya tha k injection koi nahi lagana mere pait mein us ka paani kam tha yeh beech wali bachi hai us ka paani kam tha toh mujhe bhej bhi civil diya tha civil hi reports wagera mili thi meri aur phir yeh teesri bachi huyi tab yeh sahi thi is k baari mein mein ne koi tawajju koi teeke veeke injection pe di bhi nahi aur mein ne lagwaaya bhi nahi

SS: hmm

Zakia: bas

SS: aur health worker ne aap se a k poocha bhi nahi k aap pregnant ho nahi ho is tarhan se kuch

ZA: nahi is tarhan ka koi sawal nahi kiya

SS: aap k hawale se

ZA: nahi joh bhi aga khan se aayi hain jab bhi aayin hain unhon ne kaha hai hamare paas sirf bachon ka system hai hum kabhi apne lehaaz se bhi gaye unhon ne kaha k hamare paas sirf bachon ka system hai abhi pehle jab ladies ka tha us waqt meri nayi nayi shadi huyi thi

SS: hmm

ZA a: shuro mein jab aap logon ki team aayi thi na toh ladies ka hota tha us waqt meri nayi nayi shadi huyi thi koi mein ne tawajju bhi nahi thy nahi di thi kyun k hamare yahan kohi goth hai mashaallah udhar bhi nursing department sab acha hai mere cases wahin par hote hain toh mein saatwein month se naam wahin likhwa deity hun udhar jaati hun toh udhar ki health worker ki team bhi achi hai saari

SS: hmm

ZA: toh kabhi mein idhar gayi bhi nahi thi aur aap logon ka team work khatam hogaya tha kaam khatam hogaya tha is cheez ka na woh joh ap ka joh jaise rulling chal rahi thi kabhi ek team aati hai kabhi dusri ab jab bhi jao toh seema se meri bohat achi dua salam hai mashaallah woh bohat achi hain unse mein ne pata kiya tha keh rahi hai abhi system ban raha hai naye tareeke se dobaara se start horaha hai mein last time 25 tareekh pehle gayi toh unhon ne kaha k is ko bukhaar hai 100 k kareeb injection nahi lagega jumeraat ko lagega

SS: hmm hmm

ZA: phir 25 tareekh ko is ko injection laga hai toh is ko bukhaar nahi tha toh phir unse maloom kiya unhon ne kaha k ab yeh har cheez ka dobara se banega kyun k road pe agaya hai main road pe agaya hai bare system pe start horaha hai

SS: hmm acha aur joh chota bacha aap ka hua toh sabse chota bacha us k us mein koi kirdaar ada kiya ho us ki sehat k hawale se

ZA: meri woh bachi thi bimaar rahi hai koi kirdaar ada nahi kiya us ko qabz ka masla raha hai min jab bhi gayi unhon ne kaha hamare paas aap k doctor ne bhi yeh kaha hamare paas qabz ka koi elaaj nahi hai who bachi meri itni bimaar huyi woh muthiyaan band kar leiti thi kaan se us k paseena aata tha aur woh apne aap ko bohat mushkil se poti karti thi phir hum logon ne dr ejaz saariyo ko dikhaaya hai aap k aga khan k saamne hi hai

SS: hmm

ZA: phir unho ne bola is ka gosht aage agaya hai is ka cut scissor hoga phir mein ne foran dikhaya toh us waqt corona bhi tha toh unhon ne is ko admit nahi kiya unhon ne cut scissor kar k rui daal di unhon ne kaha koi bleeding nahi hogi kuch nahi kisi ko pata bhi nahi hoga is ko ghar le jao mein 2 ghante mein wapis le aayi lekin us k baad is ko motion huye is ka paani khatam hogaya gurdon ka gurdon ka paani khatam hua toh is ko demag ka jhatka laga hum 4 bajhe dobara le k gaye phir unhon ne meri garden se paani liya us ki qamar mein lagaya abhi bhi is ko injection ka nishaan hai

SS: hmm hmm

ZA: unhon ne kaha yeh ahista ahista dhaai saal ki umar tak bhi hosakta hai k yeh chale agar is ka demaag kaam karega yeh chalegi nahi toh nahi ab bazaahir hai is ko jhatka toh nahi laga unhon ne kaha k agar jhatka nahi lagega mein is ko aise bhi le k jaati thi unhon ne qabz ka koi elaaj nahi bataya kuch nahi bataya yehi bola woh joh glyceine ka nipo hota hai aap ki doctors ne kaha woh dou woh itni dabbiyaan mein ne khatam kar di phir koi elaaj is bachi ka nahi tha is ki wajha se bachi demaagi tor pe bimaar hogayi kyun k aap ko pata hai bas unhon ne mujhe yeh kaha tango pe zor dalta hai tabhi bacha nahi chalta mein jab bhi gayi hun bachi ko isi baat se dikhaya qabz ki wajha se ya motion ki wajha se tango pe zor dalta hai toh bacha nahi chalta hai

SSa: hmm hmm

ZA: phir mein ne is ko Jinnah mein dikhaya unhon ne jab is ki qamar mein paani lagaya agar yeh dimaagi tor pe chal gayi toh yeh tango se chalegi yeh khari hogayi yeh chalegi phir last time dobara is ko qabz ka masla hone laga phir unhon ne is ko dawaaiyaan wagera di is ka gosht phir agaya tha phir finger se na dawaai laga k cut k begair hi who andar kar k bola mashaallah bachi sahi hai is ki tango pe woh joh aap logon ne woh deakhi hogi machine se check karte hain saari nassein check karte hain saari nassein check ki unhon ne kaha bachi ahista ahista us mein aayegi joh bag hoti hai woh tango k liye asal wajha hoti hai is ki qamar mein joh injection laga hai jaise bare operation wali ladies hoti hain un ka masla banta hai is ki qamar ka bana hua hai toh idhar koi elaaj nahi mila mein saara elaaj apni beti ka Jinnah se karaya

SS yeh aap ne in ko bataya worker joh aati hain pooch k jaati hain unko bataya yeh masla

ZA: nahi yeh polio ka is ko manah kiya hua tha polion wali ko pata tha aur is ka paani kam tha is ko bhi polio se manah that oh is ko bhi polio ka manah tha isi liye unko aga khan walon ne kabhi poocha nahi aap ki ladies ne kabhi nahi poocha lekin jab hamein zaroorat thi toh mein idhar jaati rahi inhon ne koi mere ko woh nahi di sirf aap ki doctors joh hoti hain ek glasses wali thi naam pata nahi kia hai woh hamesha glycerine ka nippa likh k deity thi kehti hai ek subha do ek sham do us se bache ko alag takleef hoti thi aur who us se poti toh kar leiti thi lekin hamein main wajha nahi pata thi yahan dr ejaz saario ko dikhaya

SS: toh kia lagta hai is ko kis tarhan se behtar kar sakti hain joh worker aati hain aap ne kaha itna masla hogaya hai saara

ZA: deakho woh a k pooch toh leingi

SS: hmm

ZA: sahi hai aap ne bhi pooch liya woh bhi pooch leingi is tarhan se toh parosi bhi a k pooch leite hain bache ki tabiat kaisi hai qabz horahi hai nahi horahi hai behtar yeh hai k is cheez ka elaaj aap k hospital mein hona chaiye aur us ki dawaai provide honi chaiye jaise bukhaar ki hai khaansi ki hai nazle ki hai par ek cheez aur hai yeh joh aap log mask lagaate ho nab ache ko who baar baar mein ne deakha hai kabhi saaf karti hai kabhi nahi abhi last time mein bachi ko nebulize kara k hi nahi laai mein injection lagwaane gayi bachi ko khaansi thi

SS: hmm

ZA: mein ne nebulize nahi karaya waise hi utaarti hai wahan rakhti hai waise hi laga deity hain yeh cheezein woh kia hai aap ko pata hai corona bhi hai toh yeh cheez khatam honi chaiye mask baar baar wohi ek cheez lagaaye meri nand hai woh ek dafa choti thi na gayi toh kehti hai mask change karo mein us ko aise hi utha k le aayi kehti hai mein nahi lagwaongi ek jarasim dusre ko abhi mein ne last time deakha k yehi tha k mask ek k dusre ko lag rahe thy

SS: ab yeh keh rahi hain CHW toh pooch k chali jaati hain sab kuch bhi kar leiti hain lekin center pe jaayein

ZA: center pe haan haan

SS: aur dusra elaj ki facilities hain

ZA: deakho poochne ko toh parosi bhi a k pooch leiga bacha kaisa hai qabz hai k nahi hai sahi hai tek hai nahi hai

SSa: hmm

ZA: lekin is ka elaaj hona chaiye na

SS: hmm

ZA: poochne se kia hota hai poochne se toh koi bhi pooch leiga

SS: acha us k zariye hum koi maloomaat deina chahein toh aap k khayal mein kia maloomaat health worker k zariye joh aap ko ghar beithe mil jaaye woh deini chaiye

ZA: is ka ya toh elaaj batana chaiye ya koi cheez provide karna chaiye ab hum bacha center toh le gaye main doctor beithti hain aga khan se joh aati hain unhon ne bola k is ko Jinnah le jao ya phir is ko agar aap poti nikaalna chahti ho pait sakht horaha hai pareshan toh hun hi unhon ne bola isay ghar pe glycerin ka nippa de dou

SS: hmm

ZA: ab elaaj toh koi nahi tha un k paas ab mein udhar gayi unhon ne 160 syrup likh k dawaaiyaan likhi sab likha qabz ko torne ka tareeka likha lekin unko jab main wajha pata chali toh unhon ne foran cut laga k is ko ek teeka taang mein laga foran woh is ko laga k kiya woh bachi mujhe mehangi par gayi na meri bachi ko demaag ka masla hua phir qabz takreeban 6 maheeno se qabz rahi hai

SS: hmm

ZA: toh agar yahan se is ko le k jaate ya kuch kar leite toh toh aap k khayal mein ziada acha hota

ZA: haan dekho aap log yeh kehte ho bachon ko main problem ho hum log aga khan le jaate hain hamara naam likha hua hai yeh bachi ulti motion tha mujhe koi sahulat nahi mili bachi pait mein thi mein ne bataya mera paani kam hai koi sahulat nahi mili kyun k halaanke aap log woh joh bache mein immunity kam hoti hai aap logon ne andar khaancha banaya hua tha

SS: hmm

ZA: is k baari mein ne likhwaya tha kiran k paas bhi k bache k pait mein mere pait mein paani kam hai bache ko civil likh k diya hai kisi ne koi tawajju nahi di phir is ki baari mein bataya toh koi tawajju nahi thi toh phir bataane ka faida nahi tha

SS: acha sahi

ZA: mein ne yeh deakha hai aksar bimaar bachon ko aga khan ki sahuliyaat deite hain jab naam likha ho lekin mere bache ka naam likha hua hai kabhi bhi mujhe aga khan ki sahulat nahi mili bas yehi joh center se dawaai mil gayi ek naak mein daalne ka drop milte hain motion ki dawaai ya ek calpol bas

SS: bas is se ziada nahi

ZA: bas otherwise kuch nahi khaansi ho ya kuch bhi ho rejex ka syrup bottle mein daal diya nahi toh nahi

SS: acha muje yeh bataayein k CHW ne center se jora hai aap ko kisi ne bataya ho k bhae center hai idhar chale jaayein idhar yeh facility hai

ZA: nahi nahi aisi koi baat nahi hai bas naam pata yeh joh mein ne aap ko bataya yeh poochti hain baaji bethi huyi hain inse bhi pooch sakti hain itna humse maloomaat li jaati hai detailing li ghar ka pata likha aur bas khatam

SS: aur kuch nahi kiya acha kabhi aap ne yeh mehsoos kiya hai k aap ki taraf se koi rukawat ho rahi ho k aap joh hain woh CHW nahi bata rahe kisi cheez ki

ZA: nahi mere ko kis baat ki rukawat hogi mein ne aap ko saari detail di isi tarhan unko jitna pooch rahi hoti hain detail de k is tarhan

SS: extra kuch nahi bataati (laugh)

ZA: extra nahi is tarhan de k detail aur is tarhan woh cover kar k chali jaati thi

SS chali jaati thi aur kuch batati nahi hai

ZA: nahi baaki mein ne aap ko bataya baaji ka parlor hai 20 saal se toh sab jaan pehchan wali hoti hain is tarhan kabhi masla kabhi nahi hua hamein kitni detail leiti hain detail le k chali jaati hain kyun k sab goth ki hain kuch parhi huyi hain kuch nahi parhi

SS: toh mein ne aap ki baat ko samjha woh aati hain aap se poochti hain yeh bachon ka pooch liya teekon ka pooch liya aur chale gaye

ZA: bas itna hi hai

SS: aur koi aur kaam anjaam nahi deity hain

ZA: nahi

SS: thek hai na koi maloomaat deity hain

ZA: aap k table pe beithi huyi thi agle din kiran k sath joh patli si bethi hoti hai

SS: haan

ZA: mein is ko injection lagwa k mein ne us ko bola mein ne kaha meri bachi ko khansi horahi hai toh khansi ki koi dawai hai ya doctor hain toh unhon ne aage se bola k nahi khansi ki koi dawai nahi hamare paas naak mein daalne wali dawa hoti hai bukhaar ki dawai hoti hai dikhaane ka koi faida nahi aage se us ne mujhe saaf yeh bola k dikhaane ka koi faida nahi toh phir mein ne seema ko bola seema ne kaha shayad doctor ka time khatam hogaya hai shayad is wajha se problem arahi hai phir us ne sindhi mein kuch kaha toh us ne kaha doctor nahi hai na toh mein ne kaha is tarhan doctor nahi ya time khatam hogaya hamesha mujhe aage se yehi jawab milta hai k bas itna hi hai itna hi hai haan naam batao pata batao bas

SS: bas chale jao

ZA: bas chale jao

SS: sahi hai acha toh

ZA: is ki wajha se hum ne hispatal bhi change kiya kisan hospital hai yahan pe

SS: hmm hmm

ZA: hum udhar bache le jaate hain choti Jinnah ban gayi hai yahan pe aap ko pata chala hoga hispatal chorangi pe choti Jinnah hai

SS: hmm

ZA: mera husband keh rahe thy bachon ko koi problem wagera ho toh udhar dikha deina is tareeke se hum ne raddo badal karna shuru kar diya na agar hamein sahuliyaat milti bachi meri kutyaana mein daakhil rahi civil mein daakhil rahi tab bhi aga khan kahin pe bhi nahi tha is ka paani kam tha mere ko seema ne kaha tha naam likhwa deina

SS: hmm

ZA: toh jab bachi hogi koi problem hogi aga khan le jaayeinge

SS: hmm

ZA: mein naam likhwa k aayi thi koi sahuliyaat nahi thi mashaallah yeh huyi sahi thi koi problem nahi bani lekin kisi ne aage nahi poocha lekin yeh hai k jab mein hispatal gayi toh seema ne kaha bachi sahi hai mein ne kaha haan sahi hai us ki toh baari mein bata bata k aakhirkaar mujhe Jinnah jaana para bukhaar k liye jao toh woh injection bhar k panadol pila k phir unhon ne aage dawaai de k bas khatam

SS: acha

ZA: seene k liye woh saporex hai kia hai woh de deity hain bas

SS: toh thora sa mein aap ko is pe shayad yeh kabhi aap ko baat bataai ho thora sa mein aap ko batati hun yeh joh hamara center hai na har haspatal ki unit hoti hai jaise bara aga khan hai udhar aap jaayeinge aap ko har tarhan ki facility milegi thek hai lekin yeh joh is tarhan ki facilities hoti hai na in mein kuch limitations hoti hain jaise is mein bohat saari dawaaiyaan aap ko nahi mil sakti hain joh wahan pe doctors beithy huye hote hain

ZA: mere husband ne hamesha mujhe yeh kaha hai k agar dawaai nahi deite hain acha doctor check karein likh k de dou

SS: haan

ZA: likh k de dou hamare sath unhon ne woh corporate nahi kiya mein ja meri bachi hai nay eh aise raaton ko beth beth k khaansti thi bohat khaansti thi yeh baaji kisi ne bola ambrood karo kisi ne kaha shehad karo mein ne duniya k shehad dhood liye joh totka is bachi k liye bataaigayi is bachi ne waise sehat nahi pakri itne itne baazu thy yeh raaton ko beth k na aise khaansi jaise kisi ne kisi ko nasha nahi diya hota is qisam ka khaans hai

SS: hmm

ZA: mein in k paas gayi inhon ne mujhe rejex bhi nahi diya kuch nahi diya unhon ne kaha woh antiallergy hai woh hum nahi deinge hum khaansi ka syrup bahar ka likh k nahi deinge woh gurdon pe asar karta hai kisi doctor ne kuch nahi diya agar inhon ne kisi ko totka bataya hai shehar se shehad le k aayi hun daar cheeni pees k daalni thi is bachi ki khaansi us se gayi

SS: sahi toh isi tarhan ki cheez hai na jaise k ghar beith k is tarhan ki maloomaat aap ko mil jaaye k jis se

ZA: mere khayal se inhon ne gaali ki koi jagha nahi chori thi jahan se is k liye totka nahi dhunda tha toh woh is totke se behtar huyi aga khan ne koi bhi nahi hum ne doctoron ko bhi dikhaya ache doctoron ko bhi dikhaaya lekin baat yeh thi k aap ne apne center k hawale se poocha toh udhar mere shohar ne kaha un ko bolo hamein dawaai likh k de dou ki likh dou hamein un ki dawaai nahi chaiye laazmi nahi hai hum wahan dawaai leine gaye acha doctor check kar rahe hain yehi bari baat hai lekin unhon ne dawaai bhi nahi likh k di

SS: thek hai

ZAa: is tarhan se deakho ghar se time nikaalna parta hai udhar beith k wait karna parta hai phir bachon k sath nikalna parta hai toh thori si tawajju hojaaye toh ziada behtar hai

SS: hmm bilkul sahi hai yeh toh center k hawale se hogayi yeh joh worker aati hai pooch k chali jaati hain jab aap center aate hain toh woh facilities ya us tarhan se aap ko deakha nahi jaata aap ka elaaj nahi hota is ki wajha se thori si mayoosi hoti hai thek hai aur koi cheez kehna chaheingi bohat saari cheezein hamein bataai hain lekin hamara joh main maqsad hai hum yeh chahte hain k hamari gali gali mein joh larkiyan hain woh jaati hain aurton se milti hain sab se pehla joh hamare center ka joh contact person hai woh hamari CHW hoti hai na thek hai toh un k zariye hum un ko koi maloomaat pohcha sakein logon ki koi madad kar sakein k logon ka aitebaar joh hai woh center k upar ajaaye

ZA: yeh joh mein ne aap ko cheezein bataai hain joh elaaj k liye jis k liye ja rahe hain agar aap k paas dawai provide nahi hai toh aap log usko likh k de dou

SS: hmm

ZA: us ko jaise doctor check karta hai toh hum private doctor k paas bhi jaate hain unki fees 1000 bhi hai 800 bhi hai 200 bhi woh check kar k hamein medical store hi likh k deiga

SS: hmm hmm

ZA: thek hai toh chalo udhar chale gaye 19 20 dawaai mil hi jaati chalo ek time ka formula poora hojaata hai lekin dawaai likh k de dein toh yeh kehna k nahi yeh gurdey pe asar karegi demag pe asar karegi woh dawai nahi deina agar kabhi waaldein majboor hote hain toh store wale ko ja k bol deite hain khaansi ki dawaai de doy yeh dou woh de dou

SS: chalein shukriya bohat bohat zakia aap ka ………

BH-IDI-01

SS: acha NAME aap mujhe yeh bataayeingi k aap ki umar kia hai

Mn: meri umar 35 saal hai

SS: 35 saal hai acha aur NAME aap ki shadi ko kitna arsa hua hai

Mn: 10 saal hogaye hain

SS: 10 saal hua hai kitne bache hain aap k

Mn: 3

SS: 3 bache hain aur kia umar hai bachon ki

Mn: mera bara beta joh hai 8 saal ka hai beti meri joh hai 6 saal ki hai choti bachi joh hai woh abhi 1 saal ki hai

SS: 1 saal ki hai acha aur aap kitni parhi huyi hain

Mn: mein ne metric ki hai

SS: metric kiya hua hai acha aur ghar k kaam k elawa koi aur kaam karti hain koi hunar wagera

MN: hunar toh hai silaai ka kaam aata hai

SS: hmm

MN: lekin abhi bachon ki wajha se nahi kar pa rahi kuch bhi

SS: acha toh sirf ghar ka hi kaam karti hain

MN: jee bachon ki deakhbhaal karte hain yeh bhi ek kaam hai

SSa: haan

MN: bachon ko parhana un ko deakhna

SS: hmm hmm

MN: yeh bhi ek kaam hota hai maa ki zimedaari mein hota hai

SS: hmm bilkul acha ab mein na aap se joh hamari health worker aati hain na aap k ghar par un k hawale se thora sa baat karongi mujhe yeh bataayein k yeh joh workers hain kitne arsey mein aati hain aap k ghar

MN: takreeban mein toh unhein 10 saal se deakh rahi hun jab mera beta pehle paida hua tha

SS: hmm

MN: aaj se 9 saal pehle tab bhi yeh aayin thi bohat acha in logon ne khayal kiya phir jab meri beti huyi phir mein hospital mein thi mein peeche hospital gayi thi toh in ka tahafuz in ka aana bohat acha lagta hai in ka khayal karna BP check karna aur jab mein pregnant thi us waqt yeh mere paas baar baar aati thi aap ki tabiat kharab toh nahi hai aap ka kia haal hai kitna time reh gaya hai joh baby hai scissor se hote hain na toh phir in logon ne mujhse date le li phir mujhe call par poochna toh bohat acha lagta hai phir mein ne in ko call kar k bataya meri baby huyi hai mein hospital mein hun phir subha yeh mere paas pohanch gayi 9 bajhe hi mere paas thi bohat acha laga in ka aana kyun k yeh tahafuz hota hai hamare mulk mein yeh hamare liye hain

SS: hmm

MNn: toh aate hain un ka aana mujhe bohat acha lagta hai un ka kaam bohat zabardast hai

SS: acha jab yeh waise aap k paas kitne arsey mein aati hain

MN: takreeban maheeney mein 2 chakkar toh in k hote hain

SS: aati hain aur jab aati hain kia kia poochti hain kia kaam anjaam deity hain

MN: BP ka poochti hain bachon ka poochti hain mere shohar ka poochti hain meri age ka poochti hain phir mein kia kar rahi hun yeh poochti hain toh yeh k meri baby ko kuch toh nahi hua thek hain agar halka sab hi masla hota hai phir zid karti hain hamare saath chalein check kareinge saara treatment karti hain acha poochti hain

SS: sahi hai acha aur aap ko kaisa mehsoos hota hai yeh joh kaam kar rahi hoti hain aap se poochti hai

MN: bohat acha feel karti hun bohat acha mujhe lagta hai kyun k yeh isliye acha lagta hai hum un ko paise toh nahi deite lekin woh ek zimedaari k sath k jaise family se mohabbat ki jaati hai woh un ko pata chal jaaye k yeh thek nahi hai who baar baar aati hain maloomaat karti hain zid karti hain hamare center chalein wahan par doctor beithe huye hain wahan par aap k bache ko check kareinge aap ko check kareinge ek se do martaba mein in k sath gayi bhi

SS: hmm hmm hmm

MN: wahan par bhi bohat acha treatment karte hain

SS: acha acha

MN: gaari mein hamein apne sath le k gayi hain ek tahafuz hota hai kuch bhi nahi hota koi dar nahi hota in k sath jaane se koi aisa masla nahi hai

SS: aap ne kaha koi dar nahi hota kia wajha hai koi dar nahi hota kaise aitebaar karti hain

MN: bas woh bhi toh ek waaldein hain na jinhon ne hamare tahafuz k liye hamare kaam k liye apni betiyon ko bheja hua hai hum toh shadi shuda hain un mein unmarried bhi hoti hain married bhi hoti hain toh who ghar ghar jaati hain eke k ghar pe ja kar poochti hain un ka kaam hi yehi hai nahi woh chaaon deakh nahi woh dhoop deakhti dhoop mein piyaas mein apne rozo k ayaam mein jab un k roze chal rahe hote hain tab bhi woh aati hain who yeh nahi deakhti hum logon ne roze rakhe huye hain mein bohat in se mutaasir hoti hun jab woh apna kaam kar rahi hoti hain apne kaam ko sar anjaam deity hain toh hamein kia khatra un k sath chalne mein

SS: hmm hmm

MN: hamein jab woh kehti hain mein sath jaati hun un k

SS toh is wajha se aap un k upar bharosa karti hain

MN: jee

SS: un ka rawaiya kaisa hota hai aap k sath

MN: bohat acha hota hai

SS: hmm

MN: bohat pyar se bolti hain ache mizaaj se bolti hain kabhi aisa nahi hai bas kaam se insaan chirchira hota hai hum log kaam kar rahe hain hum ne jawab diya hai toh thek hai nahi toh who gayin

SS: hmm

Mn: har cheez ko bare disciplines k sath woh karti hain

SS: hmm acha jab aap aakhari dafa hamal se thi jab aap pregnant thi toh us mein CHW ne koi kirdaar ada kiya tha joh worker hai us ne kuch kiya aap k koi kirdaar ada kiya ho us mein k hamal k doraan kuch bataya ho kahin le k gayin ho

MN: mein ne bola na aati hain le kar jaati hain ek se do martaba mein nahi bhi gayi bache hote hain na chote

SS: hmm

MN: zid karte hain toh phir who ghar pe a k mera un logon ne fever check kiya hai

SS:: hmm

MNphir mujhe mera wazan ghar par check kar k gayin hain sab kuch unhon ne kiya bohat acha kaam karti hain

SS:a: acha toh unhon ne ghar par aap ko check kiya

MN: jee jee mera weight bhi check kiya hai mera fever check kiya

SS: hmm hmm acha aur joh nauzaida chota bacha hua joh 1 saal ka hai

MN: jee

SS: us k hawale se kuch kiya unhon ne

MN: haan tab bhi woh aati rahin lekin us waqt mein ghar pe nahi thi na ammi k ghar chali gayi thi toh phir mere sath wale parosiyon ne bataya tha woh arahin thi aap k maloomaat k liye arahi thi hum logon ne un ko bataya phir jaise mein ghar pe aayi thi phir woh unhon ne akar poocha bola bacha bhi thek hai mein thek hun

SS: hmm hmm

MN: ab sab kuch ok hai

SS: toh bache ko deakha unhon ne

MN: jee bache ko phir bhi deakha

SS: kia dekha

MN: wazan bache ka wazan check kiya tha bache ka fever check kiya tha us k hifazati injection ka mujhse poocha

SS: hmm hmm hmm

MN: yeh hai jab padaaish k waqt kuch hua toh nahi is ka wazan kitna tha

SS: hmm

MN: kahan par yeh paida hua hai

SS: acha yeh saari cheezein

Mn: yeh saari maloomaat un logon ne li thi

SS: acha mujhe yeh batao k jab yeh apna kaam kar rahi hoti hain aap se poochti hain k abhi aap hamal se toh nahi yeh saari cheezein aap se poochti hain koi miscarriage toh nahi hua aur aap pregnant hain agar aap bataate ho k aap pregnant hain aur bacha hai toh woh koi aur maloomaat bhi de rahi hoti hain aap ko sath sath kuch bata bhi rahi hoti hain

MN: jee jee is se pehle mera miscarriage hogaya tha na

SS: hmm

MN: us mein bhi unhon ne saari maloomaat li thi

SS: hmm

MN: phir mujhe unhon ne bataya k aap bed rest kiya karein aap ziada kaam nahi kiya karein un logon ne saari mujhse wajha poochi kyun bimaar huyi ho

SS: hmm

MN: aisa kia hua hai baar baar woh mere paas arahi thin

SS: acha acha

MN: mere bimaar hone ki wajha unhon ne dariyaaft ki phir un logon ne mujhse kahan hamare sath chalein doctor k paas le k jaate hain toh mein ne bola mere bache chote chote hain in ko koi rakhta nahi hai mein abhi sath nahi ja sakti un logon ne mere sath poora taawon kiya

MN: taawon kiya tha unhon ne aap ko bataya matlab maloomaat si

MN: jee jee

SS: aap ko lagta hai k joh woh aati hain un ko munasib tarbiat haasil hai

MN: jee jee bilkul

SS: k matlab un k andar qaabiliyat hai un k andar

MN: jee un k andar qaabiliyat hai

SS: qaabiliyat aap ko lagti hai

MN: jee jee lagti hai

SS: kis tarhan se

MN: jab hum kaam kar rahe hote hain kaam churwa kar kehti hain 10 min chaiye mein apna kaam chor kar un ko time deti hun

SS: hmm hmm

MN: toh bohat acha kaam kar rahi hain seekhti hain toh woh arahi hain na toh phir hum se sawalaat karti hain

SS: hmm hmm acha joh health facility hai is se jorne mein koi kirdaar ada kiya hai CHW ne joh center hai jis pe aap ko kehti bhi hain

MN: jee jee

SS: toh koi aap ko lagta hai k is k baare mein koi maloomaat farhaam kiye hon unhon ne

MN: jee

SS: kaise

MN: (laugh) kaafi karti hain

SS: hmm hmm kia bataati hain us k baare mein

MN: acha bataati hain kehti hain wahan par bhi hamari doctor hain jin se hum log seekhte hain hum log seekh k aati hain

SS: hmm

MN: wahan par hum aap logon k khidmat k liye wahan par doctor bethe huye hain who hamein bataate hain phir aage hum nikalte hain ek bohat bara idaara hai joh yeh kaam kar raha hai

SS: hmm hmm hmm

MN: yeh saari maloomaat leiti hain bataati hain

SS: acha aap ne yeh feel kiya hai k aap ki taraf se koi rukaawat horahi hai CHW ko kuch bataane mein koi nayi CHW ko jaanti nahi us ko bata douge aisa kabhi kuch hua ya aap ki taraf se koi rukawat joh aati hain CHW un ko bata deite hain aap

MN: jee

SS: aap kaise bharosa karti hain kia wajha hai

MN: mein ne bataya na woh aati hain hamari khidmat k liye aati hain baar baar aati hain

SS: hmm

MN: un ki mohabbat se pata chalta hai

SS: hmm

MN: un k ikhlaaq se pata chalta hai toh mein bharosa karti hun un par koi kare na kare mein bharosa karti hun mera pata karne k liye aati hain jab bhi mein doraane hamal hoti hun woh baar baar aati hain

SS: toh is wajha se un pe bharosa karti hain

MN: jee jab mera bacha ek saal ek ya do saal ka tha tab woh mere paas aati rehti hain

SS: hmm hmm

MN: bache ka poochti rehti hain phir gap hua toh poochti hain k pregnant toh nahi ho mein kehti hun nahi

SS: hmm acha is ko hum mazeed agar hum kahein k hum un k kaam ko na thora aur behtar banana chahte hain chahte hain k hum aap tak aur sahulat pohcha sakein koi maloomaat pohcha sakein toh aap kia mashwara deina chaheingi

MN: acha hai abhi tak woh jitna kaam kar rahi hain

SS: hmm us ko aur koi cheez joh aap ko lagta hai k agar yeh kar lein yeh toh kar rahi hain joh aap ko khushi hoti hai ache ikhlaaq se baat karti hain aati hain aur koi aisi cheez koi maloomaat aisi k agar who aati hain toh matlab koi patey ki baat bata dein koi mashwara de dein kuch is tarhan kis k hawale se aisa koi mashwara deina chaheingi

MN: haan mashwara yeh deina chahungi abhi jab woh ghar pe aati hain na toh woh BP joh check karti hai na toh yahan se nas se karti hain nas pakar kar deikhti hain aur ek un k paas woh pata nahi kaunsi cheez hoti hai yeh joh hamare log hote hain nay eh kehte hain yeh toh aise hi ajaati hain BP ka toh un k paas woh hota nahi jis k sath doctor bhi fikar karte hain yeh un k paas hona toh phir joh in ko log inko aise hi samjhte hain woh in ko phir ache andaaz se deakheinge kyun k in k paas BP check karne wala bhi hai fever check karne wala bhi hai toh is se acha hoga thora doctor ka samaan in k paas hoga na log in k upar ziada aitemaad kareinge

SS: acha kabhi aas paas k logon k khayalaat sune k kia hai in k baare mein

MN: ziadatar toh ache khayalaat hote hain lekin kuch anparh hote hain woh kehte hain in k paas kuch hota toh hai nahi

SS: hmm

MN: yeh aise hi hamara time waste karte hain aage chalo khud bhi yeh check kar sakti hain BP check nahi karti bas waise hi bata deity hain weight check karti hain in logon ko joh parhe likhe nahi hote in ko weight ka kia pata

SS: hmm

MN: weight hamara kitna zaroori hai kam hai ziada hai

SS: hmm

MN: lekin hamein toh is cheez ka pata hai na

SS: hmm kuch log is tarhan ki baatein karte hain

MN: jee

SS: aap ko lagta hai is tarhan ki un k paas aalaat hon

MN: jee jee in k paas kuch cheezein hongi na phir in ko log ziada ehmiyat aur ehsaas kareinge

SS: hmm acha aur koi cheez kehna chahoge MN

MN: nahi bas mein yehi kehna chahongi bohat acha hai

SS: hmm

MN: khuda in ko barkat dein un k sath ho

SS: hmm hmm hmm

MN: yeh hamare mulk k Pakistan k yeh bohat khidmat kar rahi hain

SS: hmm hmm acha aap log ka joh yeh elaaqa ka yeh konsa elaaqa hai

MN: yeh bhais colony road no. 6

SS: road no 6 hai aur yahan se aap ka center kitna dur hai

MN: woh 8 no. par parta hai

SS: yeh 10 hai

MN: yeh 6 no. hai

SS: 6 no. hai acha acha toh center jaane mein asaani hojaati hai

MN: haan asaani hai koi mushkil toh nahi hai

SS: jaate hain aap center

MN: jee mein jaati hun

SS: toh matlab in ki jaane ki wajha se jaate ho ya waise hi chale jaate ho matlab health worker aati hai is wajha se jaate ho k yeh udhar se a rahi hai waise

MN: nahi nahi waise hi jaate hain un k wahan par treatment acha karte hain

SS: hmm

MN: mein ne do martaba deakha hai mein 2 se 3 patient ko bhi le kar gayi thi kaam acha karte hain doctor joh hai woh sweet check karte hain

SS: hmm hmm

MN: idhar udhar jaane ki kia zaroorat hai jab doctor ache hain ek hamare mulk mein jab hai hamare liye hamari khidmat k liye rakha hua hai un logon ne woh hospital toh hamein wahin par jaana chaiye

SS: sahi hai chalein thank you so much bohat bohat shukriya aap ka……..

BH-IDI-02

SS: acha NA sab se pehle aap mujhe bataayein aap ki umar kitni hai

NA: meri umar hai 26

SS: 26 saal hai

NA: jee

SS: aur aap ko yahan rehte huye kitna arsa hua hai

NA: mujhe toh yahan shadi kar k 5 saal hogaye hain

SS: aur kitne bache hain aap k

NA: 2 bache hain

SS: 2 bache hain aur kitne bare bare hain

NA: ek 4 saal ka hai

SS: hmm

NA: aue dusra 8 maheeney ka hai

SS: 8 maheeney ka hai

NA: jee

SS: acha aur aap kitna parhi huyi hain

NA: graduation hai

SS: graduation ki huyi hain ghar e koi aur kaam karte ho koi hunar wagera

NA: nahi housewife

SS: housewife acha acha mujhe yeh batao k hum na aap se CHW s joh aati hain hamari un k hawale se sawal karongi yeh joh hamari health worker hai yeh aap k ghar pe kitne arse baad aati hai

NA: aati rehti hain bachon k mutalliq wagera un ka jab round lagta hai aati rehti hain

SS: matlab kitne time k baad aati hain

NA: ab yeh toh mujhe confirmed nahi pata k kitna time

SS: phir bhi

NA: aati rehti hain waise woh

SS: haan andaazan

NA: mein jab pregnant thi tab bhi aayin thi aur is k baad yeh jab paida hua tha tab bhi aayin thi 2 3 dafa a chuki hain

SS: is saal mein

NA: haan a chuki hain 3 dafa a chuki hain

SS: acha mujhe yeh batao k jab woh aati hain toh kia kaam karti hain kia poochti hain aap se

NA: bachon k mutalliq poochti hain aur joh un ka kaam hota hai un se mutalliq sab kuch

SS: maslan

NA: matlab k is ka wazan kiya tha aur is ki length ki thi toh aayin thi is k baare mein poocha tha kuch kahan paida hua hai kahan hai yeh sab kuch aur phir is ki length ki thi weight bhi kiya tha

SS: aur aap se kuch sawal kiya

NA: nahi

SS: aur aap keh rahi thi na k jab aap pregnant huyi tab bhi aayin

NA: jab aayin thi haan

SS: toh tab aap se poocha tha

NA: tab haan mujhse poocha tha k operation se hota hai normal hota hai kahan hota hai kitne bache hain aap k yeh sab kuch

SS: hmm acha aap k ghar mein sirf aap se poocha ya baaki joh hain aap k

NA: nahi aati hain meri joh yeh devraani hai tab un ka beta paida hua tha tab un k liye aayin thi un ka toh poora woh hua tha 4 maheeney ka

SS: hmm

NA: beta tha un ka project that oh woh aati rehti thin har haftey

SS: acha yeh mujhe bataayein k aap jab hamal se huyin CHW ne koi kirdaar ada kiya tha us mein

NA: matlab mein samjhi nahi

SS: matlab jab aap hamal se thi matlab aap pregnant thi toh us mein koi CHW ne aap k sehat k hawale se aap ko kuch bataya ho koi us ka role tha us mein

NA: nahi us ka toh koi role nahi tha

SS: toh aayi unhon ne poocha

NA: woh poochi k kitne maheeney pregnant thi ye aati thi

SS: hmm

NA: 2 dafa hi aayin thi 3 dafa

SS: hmm

NA: baazu ki golaai bhi kit hi unhon ne bas

SS: acha acha bas yeh kar k chali gayi aur kuch kiya tha

NA: kuch nahi kiya tha

SS: acha yeh mujhe batao k delivery kahan karwaai thi aap ne

NA: jee

SS: aap ne delivery kahan karwaai thi

NA: mein gulshan hadeed mein national hospital

SS: acha toh aap center par aati hain

NA: mein haan gayi thi in ko hifazati teeke wahin se lagwaaye us ko bhi lagaaye un ko bhi

SS: hmm is center ka aap ko kis ne bataya khud se pata hai

NA: haan pehle yeh center mein jeth hai na wahin pe tha

SS: acha acha toh yeh joh worker aati hain is se koi madad milti hai k aap ko koi sehat k hawale se koi maloomaat milti ho ya center pe jana hai kab jana hai kuch is tarhan ki maloomaat milti ho

NA: nahi 4.00

BH-IDI-03 \

SS: Assalam o Alaikum MM mera naam name hai mein aga khan ki university se aayi hun MM mujhe yeh bataayeingi k aap ki umar kitni hai

MM: meri umar 23 saal

SS: 23 saal hai aur aap k kitne bache hain

MM: meri 4 betiyaan hain

SS: 4 betiyaan hain

MM: jee

SS: aur sab se choti bachi kitne saal ki hai

MM: 1.5 saal ki

SS: 1.5 saal ki aur sab se bari

MM: 10 saal

SS: 10 saal ki hai shadi ko kitna arsa hua hai

MM: mere 10 15 saal huye

SS: acha parha hua hai kuch

MM: school nahi quran paak parha hai

SS: Quran majeed parha hai aur ghar pe koi kaam karti ho

MM: jee saara ghar ka kaam

SS: nahi ghar k matlab k ghar k kaam k elawa koi silaai karhaai is tarhan ki koi cheez

MM: yeh karte hain woh lace wais bana leite hain

SS: hmm

MM: aur dupatte laate hain us k upar kuch motiyaan wagera laga leite hain

SS: toh aap inhi k liye karti ho ghar k elawa bhi

MM: jee

SS: acha mein na aap se thore se joh hamari health worker aati hain un k kaam k hawale se sawal karongi acha mujhe yeh bataayein joh yeh health workers hain aap k ghar pe kitne arse baad aati hain

MM: aati hain kabhi na kabhi aise chakkar laga leiti hain phir chali jaati hain

SS: kitne time k baad aati hain

MM: kabhi maheena baad ajaati hain kabhi do maheeney baad bhi kabhi teen maheeney baad bhi aise

SS: hmm acha do maheeney teen maheeney k baad aati hain

MM: haan aise aate hain

SS: acha aur jab woh aati hain toh aap se kia kaam karti hain kia poochti hain

MM: yehi k aap k ghar mein kitne afraad hain koi aise bhi ajaati hain kitne log hain aap k saas sussar in sab k baare mein pooch taaj karti hain yeh in k baare mein bolti hain aur umar ka in cheezon ka pooch k chali jaati hain

SS: aur kuch poochti hain aap k hawale se

MM: haan pregnant ho k nahi yeh bhi poochti hain

SS: acha yeh joh poochti hain aap pregnant hain k nahi hain yeh kitne arse baad aati hain

MM: abhi toh bilkul nahi aati pehle bohat ziada aati thi ab bilkul bhi nahi kitne saal hogaye nahi aati hain

SS: nahi aati hain

MM: nahi

SS: aap log yahin par hain

MM: 3 saal hogaye us k baad koi itna ziada koi aata bilkul bhi nahi

SS: acha

MM: haan pehle bohat ziada aate thy jab meri shadi huyi thi tab bhi aate thy phir jab us k baad devraani ki shaadi huyi tab bhi aati thi aur phir bachi bimaar hoti thi un ko bol deite thy kuch karte bhi nahi thy

SS: hmm hmm

MM: abhi toh nahi aati hain

SS: acha abhi pichle ek maheeney mein koi nahi aayin aap k paas pichle do ya ek maheeney mein

MM: nahi koi bhi nahi aaya

SS: bhara ho koi form bhara ho

MM: nahi nahi koi bhi nahi aaya hamare ghar mein meri devraani pregnant hain par koi aaya toh nahi hai poochne

SS: hmm aap log yahin par hain kahin gaye huye toh nahi thy gaaon wagera

MM: nahi gaye

SS: pichle saal jaise

MM: haan pehle gaye thy jab corona tha tab gaye thy hum

SS: wapis kab aaye

MM: 10 15 din reh k wapis agaye thy phir apni gaari ki na

SS: hmm

MM: us mein hi agaye thy woh saari gaariyaan band thi

SS: hmm

MM: isi liye wapis phir agaye

SS: acha acha acha yeh mujhe bataayein k jab aakhari dafa hamal se huyin thi kab huyin thi

MM: hamal se

SS: hmm

MM: yeh abhi 1.5 saal ki hai toh is se peeche chale jaayein to kitna waqt hogaya

SS: 1 saal pehle na

MM: haan

SS: thek hai

MM: 1.5 saal pehle toh huyi thi

SS: us mein aap k hamal k doraan CHW ne koi kirdaar ada kiya kuch kiya ho aap k liye

MM: kuch bhi nahi kiya

SS: kyun aayi nahi aap k paas

MM: nahi koi bhi nahi kuch karti nahi sirf pooch k chali jaati hain ek insaan hota hai kisi k liye kuch kar leita hai ya dawaaiyaan deita hai taakat ki kuch deita hai toh phir bhi sahi hai pooch k a k chale jaaye is se toh kuch nahi hota waise aayi bhi nahi hai

SS: hmm

MM: hum bimaar pregnant hui na us mein toh aaye bhi nahi hai

SS: hmm acha toh jab woh poochti hain aap unhein bata deite hain sab kuch

MM: haan bata deite hain

SS: araam se

MM: jee

SS: acha toh aap ne unse kabhi sawal kiya yeh joh mujhse aap pooch k le ja rahi hain kyun pooch rahi hain

MM: bolti hain aap k liye hum log yeh kareinge aap ki dawaaiyon ka in cheezon k baare mein

SS: kia kareinge

MM: toh hum bolte hain toh who bolti hain hamari duty hai hum isliye poochte hain mein ne kaha acha sahi hai jaise abhi aap aayi hain isi tarhan kisi ka dil toh nahi khafa kar sakte hain na

SS: thek hai toh woh aap se poochti hain aap unhein bata deite hain

MM: jee

SS: aur us k elawa woh kuch nahi karti

MM: nahi kuch toh karti nahi

SS: acha

MM: bas pooch k chali jaati hain

SS: aur kia kia poochti hain

MM: yehi poochti hain aap ka kaunsa maheena hai konsi taareekh hai koi pregnant hai k nahi yeh sab pooch kar likh kar chali jaati hai

SS: acha aur bacha jab hua yeh choti bachi is k hawale se koi kirdaar ada kiya ho

MM: nahi mein khud gayi thi phi raga khan wahan pe is ko check kiya khara kiya yeh sab kuch wazan check kiya wahin par

SS: hmm

MM: yahan pe toh koi nahi aaya

SS: koi nahi aaya aap k paas

MM: hmm

SS: acha aap ko unhon ne kabhi center k baare mein bataya

MM: kaisi

SS: k hamara aga khan ka center hai kuch is k baare mein kuch bataya

MM: aayi hi nahi toh kaise bolte

SS: acha ek do saal mein nahi aayin

MM: nahi phir abhi abhi kuch din pehle ek aayi thi injection laga rahe thy

SS: hmm

MM: yahan par camp lagaya tha

SS: hmm

MM: phir doctor ko bola tha injection laga lout oh tab ek aayi thi un doraan pehle

SS: hmm hmm hmm yeh hamal aur bache k hawale se poochne aayi

MM: nahi nahi is k baare mein koi nahi aayi wohi aayi thi ek

SS: acha jab aati hain koi maloomaat

MM: aur katre wali pehle aati thi who bhi ab nahi aati

SS: nahi aati acha yeh mujhe batao k jab who aati hain toh koi maloomaat deity hain aap ko kuch batati hain

MM: nahi aise kuch nahi batati khaali pooch k phir wapis chali jaati hain

SS: acha

MM: jee

SS: na kuch batati hain na kuch deity hain kuch bhi nahi

MM: nahi kuch bhi nahi deity

SS: hmm

MM: insaan hota hai kisi pehle woh aati thi pehle pehle jab nayi nayi shadi huyi thi aati thi taaqat ki dawaaiyaan de k chali jaati thi aur yeh sab kuch pooch taaj k chali jaati thi bohat ziada sahi thi ab toh koi bhi nahi kuch bhi nahi

SS: hmm acha toh aap kia chahte ho k yeh CHW hain is k kaam se hum kis tarhan se behtar bana sakte hain aap k sehat k liye bhi acha ho bache ki sehat k liye bhi acha ho

MM: maa pregnant ho us k liye kuch taaqat ki dawaaiyaan dein insaan ki kabhi gunjaaish hoti hai kabhi nahi hoti hai agar nahi ja sakte un k liye koi tablet sahi se de dein toh sahi hai na aur bache bhi bimaar ho itne motion lage hote hain in ki bhi aga khan mein bhi ja ja k beth beth k kuch un ki dawaaiyaan khatam hogayi wapis bhej deite hain

SS: yeh center mein

MM: jee bilkul bhi gor nahi karti ulta hamein baatein sunaati hain yeh nahi do woh nahi do bache hain woh toh khaate hain yeh nahi do woh nahi do jao jao dawaaiyaan nahi hai aise toh log nahi karte yeh log aise karte hain tabhi hamara dil toot gaya hai ab nahi jaate kisi se karza le k bachon ko le jaate hain doctor k paas insaan ki gunjaaish nahi hoti isi liye yahan jaate hain na koi masti se toh nahi jaata wahan ja k beth k khuwaar ho k aur kuch de bhi nahi toh dil nahi khafa hogaya insan ka aise hi hum log ajaate hain kitni dafa hua hai is tarhan abhi tak yehi ja rahi hai wahan pe

SS: acha

MM: kisi na kisi ki jaan pehchaan hote hain un ko de deity hain joh apne rishtedaar hote hain au raise jaate hain un ko toh kuch bhi nahi milta beth beth k khuwaar ho k ajaate hain

SS: yeh kitni poorani baat hai aap gaye ho bache ko le kar

MM: abhi abhi gayi thi mein abhi abhi hum log gaye thy bola dawaaiyaan khatam hogayi jao aur aurton ko sahi de rahi thi sindhi yeh log jaate hain apni jis k rishtedar un ko toh

sahi milti hai hum log jaate hain kuch bhi nahi milta hamare jitney bhi pathan jaate hain hamare jitni bhaabiyaan hain who jaati hain hum log jaate hain hamein kuch bhi nahi milta beth beth k ghar ka kaam bhi chor deite hain wahan chale jaate hain kuch bhi nahi milta wahan hamein toh phir dil insaan ka khafa hojaata hai toh phir jaane ka faida kia kisi se karza le kar bachon ko le jaati hun jab mil jaaye paise toh who de deite hain aise karte hain

SS: acha toh yeh aap ko kis se pata chala joh aap keh rahe ho k joh sindhi hote hain in ko de deite hain

MM: hum khud gaye hain hum logon ne deakha hai na khud hi

SS: hmm

MM: hum ne khud deakha un logon ko milte hain hum logon ko nahi jaan pehchaan hoti hai un ka toh kaam hojaata hai aur hamein kuch bhi nahi milta bohat saari aurtein aisi gaali de k ja rahi hoti hain raaste mein hamare saath aise aur bhi bohat aurtein hoti hain bolti hain itna khuwaar hogaye yahan pe a k beth beth k kuch bhi nahi mil raha aise toh log nahi karte

SS: acha toh woh yeh aap ko batati hain is ko dawa ki zaroorat hai ya nahi hai is tarhan se kuch kehti hain

MM: bolti hain bas idhar udhar cheezein nahi khilaao nahi bimaar honge bache le jao yeh tumhari maa ho aise karti ho waise karti ho baatein suna deity hain

SS: gusse se baat karti hain

MM: haan gusse se karti hain

SS: konsi doctor hai yeh

MM: yahan pe jitni bhi beithi huyi hain jis k paas hum gaye who sab yehi baatein karti hain

SS: acha toh yeh mujhe bataayein kisi ka naam yaad hai aap ko

MM: hamein toh doctoron ka naam nahi pata yeh joh beithi huyi hoti hain

SS: acha toh wahan kabhi koi center pe larkiyaan ghar par aati hon wahan kisi ka chehra nazar aaya ho kabhi koi nazar aayi ho

MM: larkiyan toh bohat hoti hain wahan pe

SS: haan lekin who aap k ghar par bhi aati hon aisa koi kabhi nahi mila

MM: pehle pehle bohat aati thin ab toh nahi aati hain

SS: acha jab aap center pe jaate hain

MM: ek nazeera ki behan hai na woh aati hain woh katre water pilaane bas wohi nazeera us k paas bhi jaati hai who dawaaiyaan deity hain woh bhi thori bohat sahi hai

SS: nazeera kon hai

MM: yahan par joh goth mein rehti hai acha acha sindhi goth mein rehti hai bare aga khan ki hai who bhi

SS: acha acha

MM: woh dawaaiyaan de deity hain jab un k ghar chale jaate hain woh hoti hai toh nahi hoti toh woh bol deite hain

SS: acha acha

MM: haan aur yahan pe jab bhi aga khan gaye rul rul k aaye jitney bhi bache huye

SS: acha toh yeh mujhe batao k aap ne kaha k rul rul k aaye toh jab phir worker aayi aap k ghar aap ne un ko

MM: unko bhi isi tarhan baatein suna deity hun woh bolte hain wahan pe aane ka faida kia hai hum log jaate hain kuch milta nahi kia faida pehle jab aati thi isi tarhan milti thi us k baad kitna waqt hogaya aayi bhi nahi yehi injection k liye abhi abhi aayi thi pehle

SS: hmm hmm

MM: dino mein toh hamare bache k teeke poore hogaye thy hum log phir nahi gaye

SS: acha

MM: jee phir hum ne bola nahi laga rahe

SS: toh unhon ne aap se poocha nahi k kyun nahi lagwa rahe

MM: hum ne bola teeke poore hogaye

SS: bas is liye aap ne yeh nahi bataya k center jaate hain is tarhan se is liye nahi lagwa rahe

MM: nahi us bichaari ko kia bolte joh aur aati hain toh pehle aati thi un ko bolte thy hum toh wahan rul rul k kuch bhi nahi milta

SS: hmm

MM: yeh bana kis liye hai is liye toh bana hai k insaan k gareebon k kaam aaye dawaaiyon k liye bachon ki dawaaiyon k liye yeh sab isi liye toh bana hai itna bara

SS: hmm hmm hmm

MM: kitni dafa wahan pe bhi mein baatein suna k aayi hun hum log yahan pe a k beth beth k yahan se chale jaate hain yeh aap logon ne sho sha k liye banaya hua hai dawaaiyaan nahi de rahi aisi beth k apni dutiyaan kar rahi hon toh yahan pe kuch do toh sahi

SS: hmm hmm

MM: kuch bhi nahi milta wahan pe

SS: acha aap ko nahi lagta k doctor ko agar zaroorat mehsoos hoti hogi tabhi woh dawaai deite

MM: hamare bache itne kharaab hote hain motion lage hote hain woh ulti baatein hamein suna deity hain

SS: hmm

MM: baatein suna k bheij deity hain

SS: hmm acha toh acha yeh mujhe bataayein k phir hum is ko kaise behtar bana sakte hain

MM: abhi aap log deakh rahe ho bache itne bimaar hai aap dawaaiyaan sahi de dou maa bhi khush hogi who phir hamesha aap k paas hi aayeingi na toh isi se hi ehsaas toh karna chaiye

SS: acha toh hum yeh chah rahe hain k health worker k zariye kaam ko behtar banaayein kaise banaayein

MM: acha sa banaayein gareebon k kaam aaye aap log sab

SS: yeh joh larkiyaan aati hain na kia maloomaat yeh aap ko dein aap k liye faida hoga kia cheezein aap ko bataayein

MM: ab hum kia keh sakte hain

SS: nahi aap jaise aap ko pata toh hai na k bhae aap k kia masaail hain aap k bachon k kia masaail hain us k hawale se aap ko kis kisam ki maloomaat dein

MM: pehle aise karte thy hyroof mein bhi bachon ko le jaate thy wahan par un k test wagera le leite thy aur bolte thy phir ajaana dawaaiyaan de deinge ab nahi hai khatam hogayi hai sorry aise bol k bachon se khoon bhi nikaal leite thy kuch deite bhi nahi thy

SS: hmm

MM: toh phir kia faida isi liye hum ne jaana chor diya woh ek maheena pehle mein gayi thi tab bhi isi tarhan hua bohat waqt k baad gayi tab bhi yehi haal tha phir mein ne bola wohi poorana haal hai is se acha jaaye nahi

SS: hmm aur nahi mein yeh chah rahi hun k hum is cheez ko na larkiyan aati hain na ghar pe ek toh woh aana shuru ho jaayein aap k ghar pe aur us ko behtar banaayein ta k aap k joh masaail ho woh ghar beithe hal hojaaye

MM: haan dawaaiyaan woh ghar pe bhi dein toh acha hoga un k paas hon bache bimaar hon un ko bhi dein toh sahi hai

SS: acha dawaaiyaan bhi ek toh yeh hogaya dawaaiyaan dein is k elawa koi maloomaat dein hamesha dawai toh zaroori nahi hoti

MM: abhi kabhi kabhi kisi k paas gunjaaish toh nahi hota teeka lagaane ka ya un ko zaroorat ho drip ki bhi toh who bhi faida wahan par hojaaye kitna acha hoga aga khan mein in ki sahulat bhi hojaaye toh acha hoga na gareebon k liye toh phir ziada sahi hoga

SS: acha toh is se health worker k kaam pe koi asar parega agar yeh center pe yeh saari cheezein deinge toh

MM: haan bohat hoga gareebon ka faida hoga har koi jaayega

SS: acha

MM: yeh faida phir un ko bhi hoga

SS: acha aur yeh kia dein yeh joh worker aati hain yeh kia karein yeh kia karein aap k liye

MM: yeh bhi ghar ghar mein bol dein wahan pe sahulat hai aap log pehle aati thi pooch k chali jaati thi yeh bhi bol dein na kisi ko bata dein toh phir woh jaayeinge un ko sahulat milegi toh har koi phir khush hoga

SS: hmm

MM: na unhein kuch bol sakega wahan pe sahi sukoon se jaayeinge bohat log gareeb hote hain un k paas gunjaaish nahi hoti toh wahan chale jaate hain aga khan hi bhaagte hain na phir aur wahan se bhi kuch na mile yahan pe ajaaye toh

SS: matlab yeh kehna chah rahe ho k joh worker hain who aap ko yeh bataaye k center pe yeh yeh cheezein mil rahi hain ta k aap phir jaayein toh aap ka time zaaya na hon

MM: haan

SS: thek hai is tarhan se aur aap k hawale se koi jab aap hamal se hoti hain chota bacha hota hai is k hawale se kuch bataayein aap ko koi aap yeh chahte ho

MM: haan aisi bhi ho toh sahi hai na insaan k sahulat wahan pe bhi thori bohat ho toh sahi hai

SS: acha lekin worker aap ko matlab ghar a k koi maloomaat deina chahein toh kis kisam ki dein kis hawale se bataayein

MM: yehi insaan k

SS: kia faida hona chaiye kia kia kis hawale se bataayein toh aap k liye faidemand hoga chalo mein agar aap ki baat karon aap ki sehat k hawale se toh kia aap ko aisa bataaye ghar a k joh aap k liye faida hojaaye

MM: taakat waaqat ki syrup pehle deite bhi thy yahan pe sindhi goth mein mere woh khaala ki beti rehti thi us ka bacha hua

SS: woh taaqat ka syrup de dein

MM: woh bhi deite thy un k kapre kambal yeh joh baby lotion nahi hoti yeh saari cheezein yeh log deite thy

SS: kahan par

MM: yahin par goth mein sindhi goth mein un ka ghar tha na sab deite thy aur abhi pata nahi hamare ko toh zindagi mein nahi mila abhi abhi aaye thy 3 4 saal hogaye aati thi yahan goth mein goth waale logon ko milte yahan pe nahi milte kapre bhi bachon ko deite thy kambal bhi

SS: yeh konsa elaaka hai

MM: yeh sindhi goth

SS: nahi aapka konsa elaaka hai

MM: yeh TCS school k paas hi

SS: acha acha acha chalein koi aur cheez baaji koi aur mashwara deina chahoge k hum is kaam ko behtar bana sakein

MM: bachon ka ziada khayal rakhein un ko drip ki ya teeke ki zaroorat ho wohi lagaayein aura chi si dawaaiyaan dein insaan khushi khushi jaayega khushi khushi aayega itna khuwaar bhi nahi honge woh log

SS: hmm

MM: aur bare aga khan mein bhi insaan k bare baron ki bhi aisi hon na teeke veeke ki ya drip wagera ki zaroorat ho toh wohi lagaaye toh acha hoga

SS: chalo shukriya

MM: yeh gareebon k liye bana hai na

SS: hmm

MM: un k liye kaam aayega toh acha hoga waise itna bara hispatal bana k beithe huye hain aur kuch de hi nahi rahe toh insaan ka dil khafa hota hai pehle chota sat ha tab bhi yeh haal aur itna bara bhi kiya tab bhi yeh haal hum khush huye chalo itna bara hogaya abhi toh kuch na kuch milega hamein bachon k liye sahi faida hoga toh hum gaye tab bhi wohi haal

SS: hmm

MM: kuch bhi aisa nahi hua

SS: acha lekin mein yeh chah rahi thi k CHW ko woh toh center ki baat hogayi na wahan ka aap ne bata diya yeh saari cheezein hon yeh joh ghar pe aurtein aati hain larkiyan aati hai

MM: toh ghar pe bhi kuch nahi hota

SS: hmm

MM: kuch bhi faida nahi hota bachon ka yehi bas likh k umar likh k chali jaati hain

SS: hmm

MM: toh is se toh kuch nahi hota insaan ka kia faida is mein

SS: toh yeh kia karein

MM: in k yeh log bhi deakhein bacha ziada kharab hai toh un ko bol dein maa ko mashwara dein de wahan chali jaayein wahan pe bohat acha khayal rakheinge aap k bachon ka aur sahi sab kuch de rahe hain phir wahan pe chali jaayein wahin par deinge yeh larkiyan toh itna sab kuch nahi le k ja sakti apne sath ya tum le lou waise bhi mashwara bhi de dein tab bhi sahi hai insan ka khayal rakhe hafte mein chakkar lagaaye insaan k ghar mein deakhein un k bachon ki tabiat ziada kharab hai toh wahan pe in ko mashwara dein toh log chalein jaayein who toh yehi samjheinge wohi haal hai wahan pe

SS: hmm hmm

MM: acha kar dein toh acha nahi hoga phir

SS: chalein shukriya bohat bohat…..

BH-IDI-05

SS: acha Assalam o Alaikum shaista mera naam name hai mein aga khan hispatal ki taraf se aayi hun hum aap se na kuch tehqeeq k hawale se sawalaat kareinge

Interview: jee

SS: sab se pehle mujhe bataaiye k aap ki umar kitni hai

Interview:meri umar 28 saal

SS: 28 saal hai aur aap ki shadi ko kitna arsa hua hai

Interview: 5 saal hogaye hain

SS: 5 saal hogaye hain aur yahan rehte huye kitna arsa hua hai

Interview: 5 saal hai

SS: 5 saal hi huye hain acha kitne bache hain aap k

Interview: mere 2

SS: 2 bache hain

Interview:ek beta bara hai aur chota yeh raha

SS: aur kitne saal k bache hain

Interview: ek 4 saal ka hai ek 2.5 saal ka hai

SS: thek hai aur parhaai ki huyi hai aap ki

Interview: jee matric kiya hai

SS: metric kiya hua hai aur ghar pe koi kaam kati hain

Interview:nahi

SS: ghar ka kaam karti hai

Interview:ghar ka kaam

SS: is k elawa koi waise aamdani k hawale se koi kaam karte ho

Interview: nahi

SS: acha shaista mein aap se na kuch sawal karongi joh hamari health worker k hawale se hain

Interview: jee

SS: thek hai sab se pehle mujhe batayein yeh joh health workers hain joh CHWs hain woh aap k ghar pe kitni baqaaidgi se aati hain

Interview: yeh katre pilaane ko toh aati hain aur waise check karne k liye ek do baar computer woh tablet mein joh hota hai woh yeh hain bas aur ziada nahi kabhi kabhi chakkar lagta hai bas

SS: kitne arse baad lagta hai

Interview:yeh maheeney 1.5 do maheeney baad guzar jaate hain 3 maheeney guzar jaate hain

SS: acha joh aati hain woh kia kaam karti hain

Interview: kuch bhi nahi pooch k chali jaati hain pregnant toh nahi ho

SS: hmm

Interview:teeke complete hain bas yehi hai

SS: toh yeh joh pregnancy ka poochti hain aur bachon k teekon ka poochti hain yeh kitne arsey baad aati hain

Interview:yeh bhi 2 maheeney baad 3 maheeney baad aisay hi hota hai

SS: acha toh aur precsy pregnancy ka poochti hai is k elawa aur kuch poochti hain

Interview: nahi aur kuch bhi nahi

SS:koi aur sawal karti hon aap se

Interview:nahi bas yehi pooch k jaati hai pregnant toh nahi

SS: haan

Interview:upar meri devrani us ka pooch k jaati hai pregnant toh nahi

SS: hmm

Interview:complete hai injection wagera saare card wagera deakh k bas

SS: bas yeh poochti hain

Interview: haan

SS: acha aap k hawale se koi aur sawal karti hain

Interview:nahi

SS: koi sawal nahi karti aur bachon k hawale se

Interview:nahi

SS: acha toh aap kaisa mehsoos karti hain in ke kaam k hawale se

Interview: nahi thora sat oh hona chaiye na hamein toh koi faida hona hamein koi agar faida na ho toh phir jab mein pregnant thi ek tablet tak nahi mili mujhe folic acid bol k gayin likh k de gayi hain mein ne bola bhi tha hamein bhi de k jao kuch hamein bhi milna chaiye kuch bhi nahi de k gayin

SS: hmm toh aap ko lagta hai k kuch deity nahi hai

Interview: nahi kuch toh faida toh hona chaiye na hamein bhi hum apne elaake mein dusrn se sunte hain hamare elaakon mein itna woh hota hai hamein tablet bhi milti hai hamein sab kuch milta hai

SS: hmm

Interview:toh hamare elaake mein kuch bhi faida nahi

SS: yeh konsa wala elaaka hai

Interview: bhanc colony roll number 8

SS: roll number 8

Interview: hmm

SS: acha acha toh aap ko lagta hai is area mein kuch nahi karti

Interview: kuch nahi karti

SS: kuch deity nahi hai kuch karti nahi hai

Interview: nahi

SS: acha aur kia matlab in k kaam k hawale se kia lagta hai aap ko kyun kar rahi hain kia kar rahi hain

Interview: woh toh thek hai chalein aati hain pata kar k jaati hain pregnant toh nahi hain likhna hota hai sahi hai kuch faida toh hona chaiye na agar un ko udhar se milti hai hamein bhi toh deini chaiye na

SS: acha udhar se milti hai matlab aap ko lagta hai k un ko mil rahi hain

Interview: shayad milti hone k baar woh aaye thy poochne toh un ko mein ne bata that oh keh rahe hain hamein toh yeh complain milti hai lekin hamein toh bola gaya k deini hai

SS: hmm

Interview: par yeh kyun nahi deity yeh hamein nahi pata

SS: acha koi aisa kabhi hai jaise aap ko 5 saal huye pehle milti hon cheezein ab nahi milti

Interview:nahi kabhi bhi nahi mili ek baar bhi nahi mili

SS: acha kabhi bhi nahi aap ko kuch nahi diya

Interview: nahi kuch bhi nahi diya

SS: acha yeh mujhe bataayein k jab aap akhari dafa hamal se huyin thi toh is mein CHW ne koi kirdaar ada kiya tha

Interview:kuch bhi nahi

SS: aayin thi aap k paas

Interview: nahi us time nahi aayin

SS: kitni poorani baat hai

Interview: yeh 2.5 saal ka mera beta hogaya

SS: acha tab nahi aayin

Interview: nahi

SS: kuch poocha bhi nahi

Interview: nahi ek baar shayad aayin thi woh computer pe saara woh tablet pe kar k gayi thi bas

SS: aur yeh kitni poorani baat hai joh tablet pe kar k gayin

Interview:woh toh mujhe yaad nahi hai 3 4 maheeney hogaye is ko na aaye huye un k paas hoga record

SS: hmm hmm

Interview: tablet pe kar k gayin thi

SS: acha us se pehle nahi aayin

Interview:nahi

SS: acha aur aap ko center ka pata hai k yahan par ek center bhi hai bhanc colony mein

Interview:jee udhar aage police station k udhar aage

SS: haan

Interview: udhar hi injection lagwaane gaye thy udhar hi jaate hain

SS: kis cheez ka injection

Interview: bachon ka

SS: bachon ka hifazati

Interview: bachon ka hifazati teeke

SS: acha acha toh us ka wahan ka kaise pata chala aap ko k center hai

Interview: wahan ka bataya tha na kisi se

SS: kisi se pata chala worker se nahi pata chala

Interview:nahi

SS: health worker aayin us ne kabhi bataya

Interview:nahi unhon ne hum log kisi ne bataya tha bare bete ko le k jaate thy jab pata that oh chote ko bhi le k gaye hum log

SS: acha toh jab woh aati hain kuch center k hawale se maloomaat deity hain

Interview: nahi hamein toh kabhi nahi di pooch k chali jaati hain

SS: apna kaam kiya

Interview:jee

SS: acha aap ko kia lagta hai k kyun pooch rahi hain kabhi aap ne socha is baare mein

Interview: nahi hum ne bola in ki duty hai zahir si baat hai peeche se bolte hain pooch k aao toh woh bhi hamein pooch k chali jaati hain

SS: aap unse nahi poochte koi aap se a k pregnancy k baare mein pooch raha hai woh toh personal sawal hai na

Interview: jee

SS: toh aap bata deity hain

Interview:jee hum log toh bol deite hain jab hota hai toh bata deite hain nahi hai toh nahi hai

SS: toh aap unse kabhi nahi poochti k bhae kyun pooch rahe ho mujhse kyun aaye ho hamare ghar

Interview: nahi hum ne bola un ka bhi kaam hai unko bhi peeche se bheijte hain yeh elaake hain in k paas jao unse pooch k aao unko bhi peeche record deina hota hai

SS: hmm hmm

Interview:is wajha se phir hum log bhi bata deite hain

SS: aur kabhi aap ne unse kaha aap yeh pochti hain toh hamein bhi koi faida ho kuch is tarhan se

Interview:nahi mein ne un logon ko bola tha hamein chaiye tablet wagera folic acid agar aap likh k de rahe ho toh agar aap ko milti hai toh hamein bhi do

SS: hmm

Interview: mein ne un ko bola yeh joh thermometer yeh joh bachon ka aata hai mujhe woh ek la dou woh bhi nahi

SS: kuch bhi nahi hua

Interview: kuch bhi nahi

SS: toh phir kaisa laga aap ko

Interview: bura toh laga par mein ne bola chalo unki marzi hai har kisi ki agar diya toh sahi hai nahi diya toh phir bhi sahi hai

SS: toh phir aap log kia karte hain phir aap ka hamal mein aap ne kaha k health worker ne koi kirdaar ada nahi kiya

Interview: hum log khud bhaag dor khud hi karte hain kisi ne koi bhi kuch nahi diya

SS: toh kahan gayin thi aap delivery k liye

Interview:mein gayi yasmeen hai na dr yasmeen yeh majeed colony mein us waqt mera bara beta operation se hua yeh chota bhi operationhuye dono k

SS: toh yeh koi clinic hai

Interview: clinic hai chota sa hospital bata hai

SS: us mein kia kia cheezein hain available kia kia hai matlab kitne doctors hain

Interview:kuch bhi nahi hai ek hi doctor hai un k paas ek nurse hai

SS: acha thek hai toh woh wahan delivery karti hain

Interview: jee

SS: aur yeh kahan par hai

Interview:bhanc colony majeed colony mein

SS: acha toh woh free mein hota hai ya

Interview: nahi paise diye hain hum ne

SS: acha

Interview: free mein agar hona hota hum ne karwana hi tha na idhar se karwaana tha free mein kabhi hua hi nahi

SS: toh yeh aap ne jab yeh aati hain health worker aap unse nahi kehte bhae mein

Interview: poocha mein ne kitni baar poocha meri woh rehti hain udhar nand rehti hai woh hamein batati hai bohat si cheezein milti hai bachon k liye gift de k jaate hain

SS: acha woh konse elaake mein rehti hain

Interview: bhanc woh udhar lalabaad mein

SS: acha lalabaad bhanc colony

Interview:nahi lalabaad hai woh rehri goth hai

SS: acha de kar jaate hain

Interview: jee woh unhon ne hamein kaafi bataya yeh cheezein milti hai hum ne bola hamein toh kabhi kuch bhi nahi mila

SS: acha inse nahi kabhi aap ne poocha

Interview: mein ne bola hamein bhi zaroorat ki cheezein chaiye hoti hai kabhi unhon ne nahi diya

SS: acha aur jab aap ka bacha paida hua us k baad unhon ne koi kirdaar ada kiya

Interview: haan ek baar aaye thy bas wazan kar k gayi thi bara beta hua tha na us ka

SS: aur yeh kitne arse ki baat hai

Interview:woh 5 6 din baad aayin thi bas unhon ne bola a k injection laga leina bas

SS: sirf wazan kiya ya aur bhi kuch kiya

Interview: wazan kiya naap wagera kar k chale gaye machine le k aaye thy us pe leita k bas

SS: aur kuch bataya nahi is k hawale se

Interview: nahi unhon ne bola sahi hai weight wagera a k bas injection laga leina

SS: acha

Interview:us waqt injection lagega bas

SS: bas yeh deakha bache ko

Interview: haan

SS: acha thek hai acha bache k hawale se ya aap jab pregnant huyi toh aap ko koi maloomaat di unhon ne kabhi

Interview:nahi yeh ek baar aaye thy baazu ka naap le k gaye thy bas

SS: hmm

Interview:woh likh k chale gaye thy bas

SS: aap ka

Interview: jee

SS: jab aap hamal se thi

Interview: jee hamal se thi yeh hone wala tha

SS: bas aap ka yeh le kar chali gayi thi

Interview:baazu ka naap

SS: aur phir le k yeh jab liya us k baad aap ko kuch bataya

Interview: nahi kuch bhi nahi bataya

SS: kuch nahi bataya koi maloomaat di

Interview: nahi

SS: bache k hawale se

Interview:kuch bhi nahi kabhi bhi kuch bhi nahi

SS: acha toh aap yeh nahi sochti k bas le k chale gaye kabhi sawal nahi kiya aap ne

Interview:nahi hum ne nahi kiya

SS: kyun aisa kyun

Interview: goth k liye hai

SS: matlab

Interview: aap log k elaake hain idhar sab kuch aap ko mil jaata hai woh goths hote hain ziada gareeb hote hain un k liye sab kuch

SS: acha aisa

Interview:jee

SS: aap ne kabhi unse poocha yeh toh aap ne kisi se suna hoga na

Interview:nahi idhar hi baat huyi thi idhar hi beithe huye thy mein ne jab aap ko bola than a thermometer k liye

SS: haan

Interview: aur bhi cheezon k liye mein ne un ko bola hamein bhi chaiye dusron ko bhi milti hai toh hum ne suna hai kaafi k milti hai bola nahi hum log nahi deite

SS: yeh health worker ne kaha hum nahi deite

Interview: jee hum nahi deite

SS: acha unhon ne phir aap ko kaha center visit karein toh koi cheez milegi

Interview: nahi kabhi kuch nahi mila

SS: kuch nahi mila thek hai aur aage se yeh kaha k goth k liye hoti hain

Interview: jee jee

SS: acha waise normally jab aap ko koi masla ya pareshani hoti hai toh aap log kahan jaate hain center jaate hain

Interview: haan ek baar gaye thy hum log is bara beta mera na poti nahi karta tha feed karta than a mera toh 5 din se poti nahi ki udhar hum log le k gaye phir toh unhon ne syrup diya tha aur unhon ne bola tha koi baat nahi mother feed karta hai toh hojaayega sahi hojaayega magar bachon ko koi problem nahi hoti hai pait bhi is ka sahi hai sab kuch sahi hai toh phir us k baad hum log gaye nahi kyun ke udhar sahi se check wagera nahi hota

SS: kyun check nahi hota kia hota hai kia laga aap ko

Interview: hamein mujhe sahi nahi laga jaise mein ne deakha na pehle bitha diya unhon ne number aayega toh phir le k jaayeinge aur phir number aaya doctor ne bhi bola yeh nahi hai sahi bacha toh thek hai aap us ko aise hi le k agaye ho toh phir zaroori nahi samjha mein ne

SS: yeh kitni poorani baat hai

Interview:yeh mera bara beta 4 saal ka hogaya

SS: 4 saal ka doctor thi jinhon ne kaha yeh bacha aise le k agayin

Interview: jee

SS: acha kia naam tha doctor ka

Interview:mujhe yaad nahi kaafi time hogaya na is wajha se unhon ne bola bacha thek hai aap aise hi pareshan horahi hain aap bache ko le k agayin hain hamara bacha hai hamein pata hai tabiat sahi nahi hai is liye le k gaye

SS: hmm hmm

Interview: toh jaan booj k toh nahi jata na doctor k paas

SS: toh is wajha se us k baad

Interview:jee mein ab nahi jaati injection lagwane thy tab jaati thi bache ko le k abhi nahi jaati kabhi nahi gayin hum log idhar doctor k paas jaate hain munasib samjhte hain hospital mein udhar le jaate hain

SS: kyun ek wajha aap ne yeh bataai kia rawaiya sahi nahi lagta

Interview: un ka rawaiya acha hai woh joh beithti hain parchi likhti hain bohat achi hai

SS: hmm

Interview: sab k sath karti hain sindhi bolti hain shayad un ka toh naam bhi nahi pata woh bohat achi hain aur yeh joh jinhon ne bola tha bache ko kuch bhi nahi bas aise hi is ko le aaye ho yeh baat mujhe sahi nahi lagi

SS: doctor ki

Interview: jee doctor ki

SS: hmm toh us ki wajha se aap ka aitebaar uth gaya

Interview: jee aitebaar uth gaya

SS: acha aur lekin teeke lagane k liye aap jaate ho

Interview: bas injection lagwane

SS: teeke joh lagwaate ho woh thek lagta hai

Interview: jee

Ayesha: woh time pe lagta

Interview: jee time pe lagta hai fever wagera check kar k baad mein lagaate hain woh sahi lagaate hain

SS: acha regularly aap teeke lagwaati hain

Interview: jee dono beton ko lagwaaya abhi meri nand ki beti huyi hai devrani ki

SS: hmm

Interview: us ko bhi saare lagwaaye

SS: acha aap ko lagta hai yeh joh health worker aati hain us ko koi maloomaat deine ki zaroorat hai

Interview: jee bohat si maloomaat deine ki zaroorat hai

SS: jaise k kia

Interview: jaise k hamare hawale se hamare bachon k hawale se shadi shuda life k hawale se

SS: acha 3 cheezein hogayin aap k hawale se ek bachon k hawale se

Interview: jee hum ghar pe rehte hain bohat si cheezon k seekhne ki zaroorat hai ghar pe aati hain na hamein bata k jaayeingi

SS: aap k hawale se aap ko kia laghta hai kia maloomaat deini chaiye

Interview: hamein deni chaiye k kaise hamein bhi apne bachon ko treat karna chaiye kia kia karna chaiye hamein syrup de k jaayeinge agar ghar koi problem hai bachon ko toh yeh mein syrup pila diya karon tablet hai bachon ko de diya karon bohat si zarooratein hoti hain

SS: hmm toh un mein se ek do zaroorat ka naam bataayeigi k kis pe aap ko lagta hai ziada zaroorat hai is pe hamein maloomaat dein

Interview: hamein maloomaat dein syrup wagera hamein de k jaayein hamein bolein k yeh bachon ko pilaya karo hamein bhi zaroorat hoti hai jis k liye doctor k paas hum jaate hain aap ko milta hai hamein bhi dein

SS: hmm

Interview: paise toh nahi na laagane parte agar aap visit kar k ja rahi hain toh aap yeh bhi toh cheezein le k asakti hain na hamare paas

SS: syrup kis cheez ka

Interview:fever ka bhi hosakta hai flu ka bhi hosakta hai

SS: hmm toh aap yeh keh rahi hain aam ki cheezein un k syrup wagera dein

Interview: jee

SS: like bukhaar ka

Interview: bukhaar ka ghar pe ho toh hum log dukaan se le k aate hain doctor ko dikhaana parta hai phir

SS: hmm matlab k koi aisi cheez joh ghar pe hi handle hojaaye

Interview: handle hojaaye ghar pe hi

SS: us k liye aap ko

Interview: bhaagna na pare baar baar roz roz hamein idhar itne paise laga k doctor k paas jaate hain isko dikhaate hain agar ghar pe maujood ho hamein bhi sahulat pata ho k hamari bhi sahulat hai

SS: hmm lekin is mein aap se thori si baat karongi yeh joh dawaai hoti hai na fever hogaya ya flu hogaya woh deina thora mushkil hota hai us k liye bhi kyun k hum de dein jaise aap samjhdaar hai har koi aurat itni ziada samjhdaar nahi hoti hai

Interview: jaise hamein pata hai fever mein panadol deina hai hamein pata hai hamein woh deina hai dusra deina hai yeh syrup hai nazle k liye hamare paas ho maujood abhi mein pari hun mujhe pata hai mein deakh k bachon ko de rahi hun toh yeh aap ki baat sahi hai har koi aisa nahi karta

SS: lekin agar maloomaat ki hadh tak dein k kia cheezein hum aap logon ko bataayein k faidemand ho k hum chahein k CHW k zariye hum aap tak pohcha dein woh maloomaat

Interview: hamein bhi bataayein k hamein kitna kia karna chaiye hum pregnant hon waqfe k baare mein bataayein k agar aap ko waqfa karwana hai toh yeh kar lo yeh cheezein aap k liye zaroori hai nahi zaroori hai aisa karna aisa nahi karna chaiye bohat si baatein hain

SS: waqfe k baare mein aap ko bataya jaaye

Interview: jee

SS: aur

Interview: bachon k baare mein bataya jaaye

SS: hmm

Interview: bachon ko kis tarhan treat karna chaiye agar aap parhi likhi nahi hai us ko bhi syrup de sakte hain k yeh nazla zukhaam ka hai agar ho toh pila dein

SS: hmm

Interview: woh bhi pila deigi maa hai maa sab kuch deakh k bachon ko handle kar sakti hai chahe woh parhi likhi ho chahe na ho parhi likhi

SS: acha woh aap ko koli bhi syrup ya goli deigi aap ko un pe itna trust hai k woh joh aap ko bataayeingi aap maan leinge

Interview: jee mujhe itna toh pata hai konsi cheez bachon k liye achi hai konsi nahi joh achi hogi wohi lungi na

SS: haan

Interview: agar mujhe pata hoga yeh cheez nahi achi toh unko bol dungi k inki zaroorat nahi muje yeh nahi chaiye jis ki zaroorat hogi us ki date deakhungi deakh k phir unse lungi

SS: kyun k kuch log aise hote hain jinko matlab agar deite bhi hain toh unko aisa lagta hai k inka koi apna matlab hai is wajha se hamein yeh de rahe hain

Interview: nahi agar deakhein hum log itna trust kar k bachon ko injection lagaate hain na kaafi bache hum logon ne deakhein hain woh nahi lagwaate kehte hain k mere bachon ko pain hoti hai yeh hota hai pain toh hoti hai agar hamare bachon ki sahulat k liye laga rahe hain na

SS: hmm

Interview: un k toh apne beech mein koi faida nahi hai na bachon ka hi hai na is wajha se pain hogi ek din hogi do din hogi zindagi bhar ka araam hai

SS: hmm hmm

Interview: bachon ko

SS: acha sahi hai acha yeh mujhe bataayein kabhi aisa mehsoos hua hai aap ki taraf se koi rukaawat hai k aap apna koi information ya koi maloomaat hai woh CHW tak nahi pohcha parahi hain

Interview: nahi

SS: koi is tarhan ki cheez k bharosa nahi karte hain kisi se sun liya koi cheez

Interview: nahi nahi nahi kabhi aisa nahi hua

SS: woh aati hai aap logon ko araam se

Interview: jee

SS: aap k mauhaaley ki hoti hain joh aap k ghar aati hain

Interview: nahi

SS: aap unse poochti hain k aap kahan se aayi hain

Interview: nahi nahi mein ne kabhi nahi poocha

SS: acha

Interview: aati hain paani poochte hain dikhaate hain aur woh joh poochte hain unka jawab de deite hain bachon k hawale se pregnant toh nahi hai bachon ko injection lage hain katre wale aate hain katre pilaati hai aur bas

SS: aap unko de deite hain aap un pe bharosa karte hain

Interview: jee

SS: acha waise un ka rawaaiya kaisa hota hai aap k sath

Interview: bohat acha hota hai

SS: sahi tarhan se baat karti hain

Interview: sahi se baat karti hain pyar se karti hain rawaaiya acha hota hai

SS: acha yeh mujhe batao k unhon ne kabhi aap ko center se jora hai joh health facility hai k koi kirdaar ada kiya ho k aap ko health facility se jorne mein

Interview: nahi

SS: aisa kuch nahi hai aap khud se gaye hain

Interview: jee haan khud se gaye hain bas yehi bolte hain injection agar complete nahi hai toh udhar se lagwa lo

SS: aap ko bataati hain center k hawale se

Interview: jee nahi yeh bola k injection lagwa lo bas a k hamara udhar hai center udhar se lagwa lo 15 20 din hogaye hain aayi thi bas mein akeli thi toh bas us ka keh rahi thi 2 saal se choti bachi hai na us ka deakh lein toh woh choti hai upar devrani ki beti us ka deakh k phir chali gayi thi

SS: acha sahi aur aap ka deakh liya

Interview: jee mein ne dikhaya toh kaha nahi 2 saal se chote bache hain na un ka deakh lein toh phir inka deakh k chali gayi

SS: aap ka 5 saal se chota bacha nahi hai

Interview: mera ek 4 saal ka beta hai ek 2.5 saal ka hai 2 saal se chote ka keh rahi thi

SS: acha sahi acha yeh mujhe bataayein waise 5 saal tak k bachon ko deakhte hain thek hai sahi acha yeh mujhe bataaiye k aap koi mashwara deina chaheingi k hum is ko kaise behtar kar sakte hain

Interview: aap kar sakte hain hamein bhi asal mein shadi shuda hain hamein bhi chaiye zarooratein hum log ko bohat si cheezein hain joh chaiye

SS: jaise k

Interview: jaise k

SS: aap mujhe khul k bata sakti hain

Interview: yeh joh health worker hai na condom wagera bhi deite hain hamein woh bhi chaiye hote hain in k paas bohat saare hote hain aur yeh bachon ka mujhe thermometer bhi chaiye (laugh) woh bhi zaroorat hai aur thore table wagera thore se folic acid wagera agar hon toh woh bhi mujhe chaiye syrup panadol wagera calpol wagera koi bhi ho mujhe woh bhi chaiye

SS: hmm thek hai aur agar koi maloomaat k hawale se koi cheez k jaise aap ne waqfe k hawale se kaha k aap ko cheezon ki zaroorat hai

Interview: haan waqfon k hawale se agar koi mein ne yeh bhi unse poocha tha hamare paas nahi hai koi bhi sahulat aisi

SS: haan kyun k shayad yeh deite nahi honge lekin aap ko center pe may be mil jaaye

Interview: nahi unhon ne bola tha hum nahi karte bas bachon ka hi hai bachon ka 5 saal tak aap ko koi bhi 5 saal tak problem hai toh karwa lou nahi toh injection lagwa lo bas aur hum log kuch bhi nahi karte

SS: acha toh haan lekin hum yeh kar sakte hain k hum aap ko yeh bata sakte hain k kia cheez sahi hai kis tarhan se aap ko waqfa bhi karna chaiye toh kis tarhan se waqfa kar sakti hain

Interview: haan yeh kabhi unhon ne nahi bataya k kuch bata sakte hain kuch kar sakte hain mein abhi pregnant thi mera miscarriage hogaya

SS: hmm hmm

Interview: toh isi wajha se keh rahi thi k kuch pata toh chale kuch toh ho hamare paas koi aisi cheez ho kuch k hum log bhi kuch kar sakein

SS: sahi keh rahi hain matlab aap pregnant nahi hona chah rahe rahe hain

Interview: nahi hona chah rahe kuch bhi kuch toh pata ho

SS: aap ka miscarriage hua hai ya aap ne khud abortion karwaya

Interview: nahi miscarriage hua hai bed rest bola tha doctor ne bacha hai chota bed rest toh nahi hosakta

SS: lekin abhi tak shaista jitni bhi baatein boli aap ne apni sehat k hawale sekoi cheez nahi boli k mujhe bhi sehat k hawale se koi cheez honi chaiye

Interview: (laugh) bachon ko mil jaaye wohi bohat hai bachon k liye acha hon na sab kuch toh maayein khud hi sehatmad hojaati hai apne hawale se hojaati hai

SS: lekin agar maa sehatmand hon yeh aap ko nahi lagta aisa aap hongi toh bache bhi sehatmad honge

Interview: haan jee yeh toh hai yeh keh rahi hun hamein bhi itna paisa laga k hum log condom wagera le kar aate hain agar yeh sahulat hamein mil jaaye toh ab yeh lalabaad mein yeh sab milta hai

SS: hmm thek hai

Interview: aap ne bola na khul k baat karo toh yeh milta hai unko

SS: hmm hmm

Interview: toh woh hamein bataati hai

SS: hmm

Interview: woh ghar pe bhi aati hai PT test bhi kar k jaati hai agar woh pregnant ho toh phir unko tablet bhi de k jaati hai

SS: hmm sahi hai

Interview: woh baaji hamein bataati hai hamein tablet bhi milti hai taakat ki milti hai folic acid wagera sab kuch milta hai unko

SS: lekin yahan nahi

Interview: yahan kuch bhi nahi milta

SS: thek hai toh aap yeh chah rahe ho k hum is tarhan ki services bhi honi chaiye

Interview: jee kholni chaiye

SS: ta k aap logon k liye behtar hojaaye

Interview: behtar hojaaye

SS: chalein thank you so much shaista…

IE-IDI-01

RA: Assalam o Alaikum MA mera naam RA hai aur mein aga khan university ki taraf se aayi hun acha MA sab se pehle aap mujhe thora bataayeingi k aap ki umar kitni hai

MA: meri umar hai 29 saal

RA: 29 saal acha aap ki shadi ko kitna arsa hua hai

MA: meri shadi ko 5 saal hogaye hain

RA: 5 saal hogaye hain kitne bache hain aap k

MA: 2

RA: 2 bache hain aur sab se bari bachi ki kia umar hai

MA: yeh 5 saal mein poori hogayi abhi 6 saal mein lagi hai

RA: acha aur choti wali

MA: yeh 3 saal mein poori hogayi 4 saal mein lagi hai

RA: acha aap ne taleem kitni haasil ki hai

MA: matric

RA: matric kiya hua hai ghar k kaam k elawa aur koi kaam karti hain

MA: nahi kuch bhi nahi karti

RA: koi hunar wagera jaise aap aamdani aajaaye is tarhan

MA: aamdani kuch bhi nahi hai

RA: acha mein na aap se kuch joh hamari CHWs aati hain health worker joh aati hain ghar pe un k kaam k hawale se aap se kuch sawal karongi acha sab se pehle mujhe bataayeingi joh CHWs hain woh aap k ghar pe kitne arsey baad aati hain

MA: har maheeney aati hain

RA: har maheeney aati hain

MA: jee

RA: acha aur jab woh aati hain toh kia kaam karti hain

MA: yeh poochti hain jaise sawal k aap k bache kitne hain haamla hain

RA: hmm

MA: shadi ko kitne saal hogaye hain

RA: acha aap ki shadi k baare mein poochti hain hamal se hain is k baare mein poochti hain

MA: jee jee haan poochti hain

RA: aur aap k hawale se aur koi sawal karti hain

MA: bas yehi karti hain

RA: acha aur bachon k hawale se

MA: bas bachon ka poochti hain k kitne bache hain kia naam hai

RA: acha aur is k elawa

MA: poochti hain bachon ki bas

RA: bas yeh poochti hain acha toh yeh saare sawal jab aap se kar rahi hoti hain toh aap kaisa mehsoos karti hain

MA: bas yeh mujhse sawal karti hain un ko jawab de deity hun zaahir si baat hai

RA: haan

MA: doctor hispatal se aayeingi toh woh sawal kareingi toh jawab de deity hun

RA: acha lekin aap sochte nahi hain k yeh hamare bachon k baare mein kyun pooch rahi hain

MA: zaahir si baat hai sochti hun bachon k a k bataati hain k kaise kaise toh hamare ko kabhi jaise aaye nahi k bolti hain k checkup karne ka hai aisa kuch bhi nahi hai jaise yahan pe a k poochti hain na k hamla aurat k liye hai checkup

RA: hmm

MA: 1 saal se 5 saal tak k bachon ka checkup karne ka hai lekin aisa kuch bhi nahi hota sirf yeh entry kar k chale jaate hain phir checkup wagera toh kuch bhi nahi hai

RA: toh aap poochte nahi k aap entry kar k chale jaate ho

MA: nahi phir hum kuch bhi nahi poochte entry kar k chale gaye phir dobara nahi aati

RA: acha

MA: toh phir woh ek maheeney baad hi aati hain aur yeh k elaaj ka bolti hain elaaj toh aisa kuch bhi nahi hua

RA: aap gaye hain aap ne kabhi unse kuch poocha nahi sawal nahi kiya k aap yeh keh k jaati hain elaaj hoga yeh hoga kahan hota hai

MA: poocha hai hum ne unse lekin aisa koi jawab hi nahi diya

RA: acha aap ko tasalli baksh jawab nahi mila

MA: bas sahi hai bas yehi bolte thy na hum yeh sirf entry karne aaye hain

RA: hum sirf yeh entry karne aaye hain

MA: haan

RA: acha

MA: haan aise aur kabhi yeh hum ne poocha bhi ek do jaano se k aayin hain k bhae kahan se checkup hota hai jaise aap hum se itna pooch k jaati hain hamari kar k ja rahi hain sab kuch toh hamein is cheez ki knowledge hogi na

RA: hmm

MA: kahin jaana bache ko bimaar huyi chale gaye le gaye bhae yeh elaaj k liye hai na aisa kuch bhi nahi hai

RA: matlab aap yeh kehna chah rahi hain k woh aati hain pooch k chali jaati hain

MA: haan pooch k chali jaati hain

RA: lekin bache wagera bimaar hote hain toh unse koi sahulat nahi mil rahi

MA: nahi kuch bhi sahulat nahi mili

RA: acha toh aap kis kisam ki sahulat chahti hain

MA: jaise ek hota hai na bimaar ho ek saal se 5 saal ki umar tak k bachon ko har jis ki sahulat deinge dawaai kuch bhi nahi hum ne toh nahi deakha kuch bhi yeh bas entry kar k chale jaate hain aur kuch bhi nahi phir woh har maheeney k maheeney entry karne aate hain

RA: haan acha toh us mein aap kia chahti hain k woh aap ko yeh nahi bataati koi center hai idhar le k ajaayein

MA: nahi aisa toh kuch bhi nahi bataya aisa kuch bhi nahi bataya

RA: acha jab aap akhari dafa hamal se huyi thi toh us mein CHW ne koi kirdaar ada kiya tha

MA: nahi us doraan koi bhi nahi aaya

RA: acha

MA: us doraan koi bhi nahi aaya

RA: kitni poorani baat hai 2.5 saal ki hai choti

MA: yeh 4 saal ki hai 3 saal pehle ki baat hai

RA: hmm

MA: 4 saal mein lagi hai November mein

RA: hmm

MA: aisa kuch bhi nahi hua

RA: koi nahi aaya tha

MA: nahi

RA: phir aap ne kahan se karwaaya tha

MA: mere joh dono bache huye hain private hispatal mein huyein

RA: acha toh private mein kyun karwaya scissors se huye

MA: jee scissors se huye hain dono

RA: acha toh private hispatal mein kyun karwaaya yeh poochna chah rahi hun yahan se kareeb hai

MA: haan yahan se kareeb hai landhi mein hai

RA: acha toh kia wajha thi k hamara joh center hai aga khan ka center hai wahan par nahi gaye aap

MA: nahi gaye

RA: wajha kia thi

MA: hamein bataya hi nahi gaya toh kahan kaise jaate hamein bataya hi nahi gaya na

RA: hmm

MA: agar hamein pata hota idaare ka toh hum jaate kyun nahi jaate is ka hamein pata hi nahi chala aur jinnah se bohat dar lagta hai jinnah hum gaye bhi nahi saaf si baat hai phir hum private karwa liya

RA: acha kahin aisa toh nahi k aap log k nazar mein agar kahin free elaaj hota hai k pata nahi sahi se deakheinge k nahi deakheinge aisa toh nahi

MA: nahi joh acha hispatal hota hai jaise government ka hispatal hai free hai nay eh bhi toh

RA: hmm

MA: jaate hain Allah ka shukar aisa masla nahi hua

RA: acha aur joh sab se choti aap ki bachi hai is mein us ki sehat k hawale se koi CHW ne kirdaar ada kiya ho

MA: kuch bhi nahi

RA: acha

MA: mein aap ko bata rahi hun nahi aayi sirf entry kar k jaati hai us k baad nahi poochti kisi cheez ka yeh aisa hum log poochte bhi hain bhae aap itna entry kar k jaate hain hamari toh kisi cheez ki sahulat hi nahi hai aap bas entry kar k jaati hain hamein toh kisi cheez ki zaroorat nahi hai zahir si baat hai dawaaiyon k liye private hispatal jaate hain bachon ko le k hum log toh zahir si baat hai hum logon ko sahulat milni chaiye na is cheez se

RA: hmm acha yeh joh aati hain who aap k elaake ki hoti hain CHW

MA: yeh toh nahi pata hamare elaake ki hain yeh hamein nahi pata

RA: yeh nahi pata acha kabhi aap ne un ko jaise kabhi bacha bimaar hogaya ya aap ki tabiat kharab hogayi toh aap ne kabhi khud se CHW k paas gayin k yeh aati hain hamare ghar mein

MA: awwal toh yeh baat hamein toh nahi pata kahan se aati hai pehli baat toh yeh hai

RA: hmm

MA: sahi hai na hamein nahi pata kahan se aati hai aur hum ne un ko kabhi bataya bhi hai na toh koi woh nahi koi contact nahi hai joh hum kisi ko bataayein aati bhi hain Allah ka shukar bache sahi hote hain bimaar nahi

RA: kabhi aap khudse unse poochne ki koshish karti hain k bhae tum arahi ho pooch rahi ho kia kar rahe ho kahan le k ja rahe ho hamara data kahan le k jarahe ho

MA: poocha hum ne jaise yeh aap sab karti hain data kahan kia kar rahe ho toh aga khan se kehti hain aate hain

RA: acha toh aga khan ka naam sun k aap un par aitemaad karte hain

MA: itne bare hispatal se arahe hain zahir si baat hai acha hai sab kuch

RA: acha toh kisi bare hispatal se a k koi aap se maloomaat le raha hai toh yeh aap logon k liye thek hai

MA: zahir si baat hai bilkul thek hai bara hispatal se koi araha hai insaan poochne maloomaat leine toh yeh achi baat hai k pata chale zahir si baat hai k kia hai team kaisi arahi hai kaisi nahi

RA: hmm acha aur aap ko kia lagta hai yeh joh aati hain workers in k paas munasib taaleem hai ya inko itni training mili huyi hai joh aap se sawal karti hain sahi tarhan se poochti hain

MA: yeh toh kheir mujhe nahi pata zahir si baat hai inhon ne taaleem li huyi hai bas humse do alfaaz poocti hain

RA: hmm nahi jaise ek hota hai na ek bhae arhe hain sehat k hawale se pooch raha hai

MA: nahi is tarhan ka kuch bhi nahi poochte sirf yehi k aap hamal se hain bache kitne hain bache kitne saal k hain bas aur aap ki family mein koi aur dusra shadi shuda hain

RA: tab aap unse nahi poochti mere hamal se aap ko kia leina deina kyun pooch rahi hain

MA: hum ne kabhi aisa poocha nahi hai

RA: kyun nahi poochti yeh janna chahti hun yeh toh bari personal baat hai na aap kaise kisi ko bata rahe hain

MA: mein ne toh aisa nahi bola

RA: acha chalo thek hai acha yeh mujhe btaayein k center k hawale se kabhi CHW ne aap ko jora ho koi ek health center hai wahan par bachon ka elaaj karte hain

MA: kuch bhi nahi kiya kuch bhi nahi bolti elaaj ka toh bilkul nahi bolte

RA: acha agar woh aap ko bataayeingi k bhae center hai yeh facilities di jaati hain toh kia aap log aayeinge

MA: zaahir si baat hai bilkul jaayeinge kyun nahi jaayeinge acha hoga toh kyun nahi jaayeinge

RA: yeh joh acha hai is mein kia kia cheezein ajaati hain k kia aap expect karti hain k ek center hai CHWs mein kia cheez achi honi chaiye

MA: deakhein bachon ki sehat k liye na ab bache bimaar parte hain hum jaate hain bhaag kar

RA: hmm

MA: agar center pe koi ho hamara jaan pehchaan wala toh hum jaayeinge toh aur achi treatment hogi ache se deakhbhaal hogi bachon ki aur hamari bhi is lehaaz se

RA: toh wahan acha kia hota hai woh acha toh mujhe bataayein

MA: jaise mein jaati hun bachon ko le kar doctor k paas

RA: hmm

MA: dawaai itni achi deite hain nab ache ko ek hi khoraak mein araam ajaata hai phir dobara jaana nahi parta

RA: hmm hmm acha aur hum yeh CHWs hain in ka kirdaar kaise behtar bana sakte hain aur aisa kia karein k aap in k kaam se khush hon

MA: bhae yeh jab aati hain bhae jaise mein nahi koi aur pooch le k bhae in ka center kahan hai toh yeh sab nahi bataati batana chaiye k hamara center is jagha hai aap ja k kabhi deakh lein wahan zahir si baat hai koi insaan bimaar hoga toh wahin jaayega treatment karwaayein toh pata chaleiga k acha hai k nahi

RA: hmm thek

MA: aise hi pata chalega hum logon ko ghar mein rehte huye nahi pata chaleiga bilkul bhi

RA: yeh ghar par arahi hain center k hawale se kabhi maloomaat dein k bhae center pe yeh yeh cheezein hoti hain

MA: jee

RA: acha is k elawa koi aur maloomaat deini chaiye inhein bachon k hawale se

MA: haan bachon k hawale se deini chaiye bache chote hain 5 saal tak k bachon ka har cheez mein in ko maloomaat deini chaiye

RA: acha kis kisam ki maloomaat bachon k hawale se deni chaiye

MA: teeka hota hai teeke ki maloomaat deini chaiye kitne log aise hain aurtein jinhein teeke k baare mein nahi pata na lagwaati hain is baare mein batana chaiye aur joh injection lagte hain har cheez k yeh bhi batana chaiye

RA: kis cheez ki bachon mein

MA: jee bachon ki injection har cheez ki lagti hai kabhi typhoid ki injection wagera khasra ki injection hoti hai yeh cheezein bataani chaiye aisi aurtein joh nahi parhi likhi un ko pata nahi hota is cheez ka

RA: hmm hmm

MA: toh un ko batana chaiye

RA: acha

MA: joh parhi likhi hai woh toh samjh sakti hai na john ahi hogi parhi likhi woh kaise samjheigi

RA: acha yeh mujhe bataayein aap k hawale se ya hamla aurat k hawale se koi maloomaat deini chaiye

MA: zahir si baat hai deini chaiye

RA: kia maloomaat deini chaiye

MA: ab jaise nayi nayi shadi shuda larkiyan hoti hain toh is cheez ka pata nahi hota

RA:kis cheez ka pata nahi

MA: jaise hamal ka toh nahi pata hota na mein bhi pehli dafa pregnant huyi thi mujhe nahi pata tha is cheez ka meri ammi ne mujhe guide kara yeh sab pata hai un ko

RA: hmm

MA: yeh sab guide kara kia khana hai kia peena hai in dino mein kaise uthna hai kaise beithna hai

RA: acha is k elawa koi aurat hamal se nahi hai aur koi aisi cheez hai jis k upar aap un ko lagta hai k bhae a hi rahi hai toh hamein is k baare mein bhi thori maloomaat de dein

MA: joh khunwaari larkiyaan hoti hain un ko paani ki shikaayat rehti hai na paani ki shikaayat liquoria ki ab woh kia karein doctor k paas jaati hain woh dawaai de deity hain

RA: hmm

MA: aur koi elaaj kuch poochna chahein toh yeh khaayein dawaai sahi hojaayega koi maloomaat nahi hoti

RA: hmm

MA: larkiyan pareshan hojaati hain k yeh kia horaha hai hamare sath

RA: hmm hmm

MA: mensis ki problem hojaati hai

RA: hmm

MA: us cheez ka

RA: thek hai

MA: meri choti wali munni hai na pehle mere mensis poore 5 6 din aate thy

RA: acha sahi mensis k baare mein aur joh paani ki shikaayat hojaati hai us k baare mein thora sa bataayein aur koi waqfe wagera k hawale se

MA: mein ne waqfa bhi karaya hai 5 saal ka waqfa karaya hai na mera color itna dim kyun hogaya hai mera color itna dim tha hi nahi jab se mein ne yeh karwaya hai na mein thori healthy sib hi hogayi hun aur mera color bilkul jal gaya aisa kyun hua

RA: acha yeh sawal ka jawab mein aap ko baad mein dungi

MA: jee

RA: lekin mein aap se pooch rahi hun k in cheezon k baare mein batana chaiye CHW ko

MA: batana chaiye zahir si baat hai koi parha hua nahi toh un ko bhi is cheez ka ilm hona chaiye zahir si baat hai

RA: hmm aur koi cheez MA aap kehna chahoge aap batana chahoge k jis se hum in k kaam ko behtar bana sakein

MA: nahi bas yeh hai k aapjaise k bolein hamara center hai zahir si baat hai kisi ko pata hai kisi ko nahi pata center toh pata hona chaiye na

RA: hmm

MA: hamein ja k deakhna bhi chaiye center ek dafa

RA: hmm hmm

MA: kyun jhoot bolein zahir si baat hai aap itna likhne arahe hain yeh sab kuch karne hamein bhi toh ek dafa jana chaiye na

RA: hmm thank you so much…..

IE-IDI-02

RA: acha NA assalam o alaikum mera naam RA khalid hai hum aga khan ki taraf se aaye hain mein aap se CHW k kaam k hawale se kuch sawal karongi acha interview shuru karne se pehle aap mujhe bataayeingi k aap ki umar kitni hai

NA: meri 38 years

RA: 38 years hai acha aur aap ki shadi ko kitna arsa hua hai

NA: meri shadi ko 7.5 saal

RA: 7.5 saal huye hain kitne bache hain aap k

NA: 2 bache hain

RA: 2 bache hain sab se chote bache ki kia umar hai

NA: us ki sawa 3 saal

RA: sawa 3 saal hai acha aur aap ne kuch parha hua hai kitni taleem hai aap ki

NA: mein ne BA kiya hua hai

RA: BA kiya hua hai ghar k kaam k elawa koi aur kaam karti hain

NA: nahi ghar k kaam karti hun

RA: ghar ka kaam karti hain acha acha sahi hai acha mujhe yeh bataayein sab se pehle joh CHWs hain hamari woh aap k ghar pe kitne arsey baad aati hain

NA: woh kabhi ziada din k baad kabhi kam din k baad is tarhan un ka chakkar lagta hai kabhi 15 din baad kabhi hafte baad kabhi maheeney baad kabhi 3 maheeney baad is tarhan aati hain

RA: acha aur jab woh aati hain toh woh kia kaam karti hain woh kia poochti hain aap se

NA: question wagera poochti hain aur sawal jawab bohat saare poochti hain kabhi khaane peene ka mein ne kia khaaya kia peeya bache ki routine k mutaabiq mere routine honge is tarhan k sawal karti hain

RA: aur aap k hawale se koi sawal karti hai

NA: matlab

RA: matlab kuch aap k hamal k hawale se

NA: jee is tarhan k bhi sawal poochti hain k aap hamal se hain k nahi is ka jawab batate hain k nahi hai

RA: toh joh yeh hamal k hawale se poochti hain ya shadi shuda kitne afraad rehte hain ghar mein

NA: jee

RA: is tarhan ka sawal karti hain

NA: haan is tarhan ka sawal bhi poochti hain

RA: acha toh yeh kitne arsey baad aati hain kyun k mujhe pata hai different different CHWs aati hain yeh waale joh hai woh kitne arsey baad aate hain

NA: in ka mujhe confirm pata toh nahi ziadatar aise 3 maheeney baad aisa aana hojaata hai

RA: aisa hojaata hai k woh ajaate hain acha jab woh aati hain aur aap se aap k hamal k hawale se poochti hain ghar mein shadi shuda log hain k nahi hai bachon k hawale se

NA: jee

RA: bachon k hawale se kia poochti hain

NA: bachon k matlab agar hamal wagera ka poochti hain shadi shuda aur hain k nahi hain agar koi hai toh us ka hamal hai k nahi hai aur bachon k baare mein poochna k bache kitne hain sahi hai un ki tabiat wagera k baare mein poochti hain k tabiat wagera un ki set hai bachon ko nazla zukhaam aaj kal covid chal raha hai us k baare mein poochti hain

RA: aur teekon wagera k hawale se

NA: injection wager aka bhi poochti hain injection course hua hai ya nahi hua toh bas is tarhan k sawal jawab karti hain

RA: aap kaisa mehsoos karti hain jab yeh aati hain aur aap se yeh saare sawal karti hain

NA: hum log acha feel karte hain

RA: kia acha feel karti hain

NA: matlab k hamare bache k treatment sahi mind k baare mein pata chalta hai us k growth k baare mein pata chalta hai acha lagta hai k bhae hamare bache k baare mein sab ghar beithe hamein maloom horaha hai

RA: toh woh bache k growth wagera k hawale se bataati hain

NA: jee

RA: kaise kia bataati hain

NA: woh aati hain na kabhi aisi machine wagera le k aati hain us se check karti hain pehle meri devrani ka bacha hua tha dil ki dharkan wagera check kiya weight wagera check kiya mere dono in ka bhi weight aur in ki height aur is tarhan k test wagera bhi kuch in k is tarhan check kar k ghar beithe hojaata hai

RA: toh aap ko acha lagta hai

NA: acha lagta hai

RA: acha aur is k elawa koi cheez hai joh CHW k baare mein aap ko achi lagti ho k in k baat karne ka andaaz kaisa hai

NA: jee baat karne ka bhi thora acha hota hai andaaz un ka aur sahi lagta hai

RA: aap ko in pe bharosa hai

NA: jee

RA: kia wajha hai

NA: bharosa hai isi wajha se hum log in k sath corporate karte hain joh woh kehte hain un ki baat maante hain hum toh is liye woh bhi aati hain hamare sath ache se baat karti hain

RA: hmm leikin kia wajha hai joh aap un pe bharosa karti hain

NA: wajha yehi hai k bas woh hamein ghar beithe sab kuch ghar beithe hamara horaha hai toh aur ache tareeke se horaha hai acha idaara hai is wajha se hamein aitebaar hai bharosa hai

RA: jab woh aati hain bataati hain k kis idaare se arahi hain

NA: haan toh woh bataati hain na toh is wajha se k yeh acha idaara hai bara idaara hai

RA: acha toh jab aati hain larkiyaan yeh aap k mauhalley ki hoti hain aap log jaant hon inhein

NA: nahi nahi hamari inse koi waqfiyat nahi hoti nahi jaante hum log inko

RA: nahi jaante lekin woh idaare se aati hai

NA: idaare se aati hain bas

RA: acha kia yeh hosakta hai woh joh zaban bolti hain

NA: jee

RA: aap ki zaban bol rahi hain matlab sindhi hai ya pathan aayegi ya aap usi tarhan jawab doge

NA: jee bilkul baaki woh aati hai koi bhi unki language ho hamare sath toh urdu speaking hoti hai is liye hamein unki samjh ajaati hai

RA: hmm aap k khayal se is baat se fark parta hai k matlab joh worker arahi hai

NA: jee

RA: us ki zaban agar sindhi k paas ja rahi hai toh us se sindhi mein baat kare ya urdu aurat urdu speaking hai us se urdu mein baat kare toh us se farq parta hai joh worker hai

NA: nahi agar koi bhi us ki zaban ho woh zaroori nahi hota aage wale ki bhi wohi ho lekin agar yeh urdu mein baat karte hain toh samjh mein ajaati hai

RA: samjh mein ajaati hai acha jab aap akhari dafa hamal se huyi thi toh us mein CHW ne koi kirdaar ada kiya tha

NA: jee us mein bhi unhon ne kaafi kirdaar ada kiya kaafi help wagera ki

RA: hmm

NA: toh ultrasound wagera woh bhi 2 se 3 dafa hua hai test blood test yeh bhi ki unhon ne aur jinnah hispatal mein naam likhwaya matlab wahan pe test wagera apne sath le k gayin karwaya

RA: CHWs le k gayin thi

NA: jee gaari mein le k gayin thi unhon ne wahan ja k woh bhi karwaya kaafi help wagera kari unhon ne woh joh injection hoti hai woh joh hamal k doraan lagti hai woh bhi wahin pe gaari le k aayi gaari mein aate jaate thy who bhi lagwaayi

RA: gaari aap ko leine aati thi

NA: haan kuch matlab hum ne khud ja k lagwaaya kuch un ki gaari k zariye lagaaya

RA: acha toh yeh aap ne un ko bataya tha CHW ko aap ne bataya tha toh phir unhon ne aap ko gaari bheji is tarhan se hua

NA: nahi woh khud hi a k le jaati thi

RA: jinnah kaise gayin thi aap

NA: jinnah ka toh unhon ne bataya that oh hum log chale gaye thy wahan center toh wahan gaari mein le gaye thy

RA: acha toh CHW jab aati hai toh center k baare mein bataati hai k hamara center hai

NA: jee woh toh hamein pata hi hai woh jab first time aayi thi jab bataya tha yeh area mein hamara center hai toh wahan se hum log aaye hain toh apna taaruf karwaaya toh us se phir hamein maloom hua

RA: thek hai toh phir aap center gayin thi toh wahan elaaj karwaaya aur phir unhon ne aap ko jinnah refer kiya

NA: jee

RA: acha phir jab aap ka chota bacha hai sab se last chota beta hai toh us ki sehat k hawale se CHW ne koi kirdaar ada kiya

NA: jee us ki sehat k hawale se unhon ne kaafi kirdaar ada kiya jab us ki birth huyi tab bhi unhon ne test wagera kiye a k drip wagera le k aayin thi woh de k gayin

RA: acha

NA: jee aur us k baad phi raga khan hospital bara wala wahan le k gayin test kiye is k aur phir us ko wahan udhar le k gayin peads ward joh hoti hai chote bachon ki us k liye bhi le k gayin karwaane is ko

RA: is ko aga khan kyun le k jana para tha

NA: test tha unhon ne bola tha in ka karna hai is k liye le k gayin thi

RA: le kar gayin thi

NA: jee

RA: acha aur waise wazan wagera karne aati hain

NA: jee weight wagera karne k liye 3 saal kia 2 saal bhi aati rahi hain is ka weight bhi karna height bhi karni toh saara us ki tabiat k baare mein poochna woh sab kara

RA: toh aap ko waqt pe bata deity hain

NA: jee

RA: kabhi khud se aap ne CHW ko call ki ho bataya ho k bhae mera bacha bimaar hai aise kabhi kuch

NA: nahi aise nahi hua Allah ka shukar hai is ki tabiat waise set hi rehti hai

RA: hmm ya kabhi jaise kabhi khud se kabhi zaroorat mehsoos huyi k acha in ko call kar leite hain hamara masla hal hojaayega

NA: nahi aisa koi nahi

RA: aisa nahi hua kabhi zaroorat mehsoos nahi huyi

NA: nahi zaroorat mehsoos huyi

RA: acha jab aap k ghar pe joh larkiyaan aati hain aap ko lagta hai k unko munasib training mili huyi hai joh yeh kaam kar rahi hain ya in ko aur milni chaiye

NA: nahi hamein sahi lagta hai

RA: sahi lagti hai baat cheet sahi karti hain

NA: sahi karte hain

RA: baat wagera joh aap se karti hain aap mutmaain hoti hain

NA: jee

RA: acha acha mujhe yeh bataayein k joh center hai health facility hai us se jorne mein koi kirdaar ada kiya CHW ne k aap ko pehle nahi pata tha k center hai aga khan ka aur jab yeh aayin toh phir pata chala

NA: jee woh toh hamein nahi pata chala jab yeh aayin taaruf karaya tab pata chala

RA: toh unhon ne aap ko kia bataya k center pe kia kia cheezein hoti hain

NA: unhon ne hamein yeh bataya k jab hamal hota hai toh hum naam wagera likhte hain us k baad pehle ultrasound wagera sab kuch tha us k baad jab wahan pe aag wagera lagi toh us k baad khatam hogaya tha ultrasound ka toh kheir abhi nahi hua test wagera woh karte hain

RA: hmm

NA: toh us ka bhi hamein unhon ne sab bataya tha

RA: acha aur jab aap log center jaate hain toh aap log mutmaain hote hain center ki facilities joh mil rahi hai

NA: jee bilkul

RA: acha koi aisi cheez joh aap ko nahi achi lagti ho aap chahte ho k yeh behtar hojaaye health worker arahi hain toh in k zariye hum inko behtar bana dein ghar bethe hojaaye

NA: nahi aisa toh koi masla wasla nahi hai sab thek hai

RA: sab thek hai acha koi aisi maloomaat joh aap ko lagta hai k in k zariye hum aap tak pohcha sakein bachon k hawale se kuch ho

NA: nahi sab thek hai

RA: aur aap k hawale se

NA: nahi Allah ka shukar sab thek hai

RA: koi hamal k hawale se ya jaise aurton k dusre bhi masle hote hain

NA: jee

RA: un k hawale se koi maloomat jaise arahe hain ek do message de diye bata diya kuch is tarhan ki koi cheez joh aap ko lagti ho ya larkiyon k masaail hote hon un k hawale se

NA: nahi is k baare mein itni information nahi hai bas khaali yeh hai k jahan hamal se le k bache ki delivery tak aur us k baad bache ki treatment wagera yahan k unhon ne bataya yeh hota hai yeh masle hote hain

RA: haan toh yeh bataati hain hamal se le k 2 saal tak k baare mein bataati hain is k elawa

NA: is k elawa koi masle masaail hote hain unka treatment ka nahi pata

RA: aur aap ne kabhi poocha bhi nahi

NA: nahi

RA: toh wohi mein poochna chah rahi hun aurton k aur bhi masle masaail hote hain

NA: wahan pe mein center bhi gayi hun wahan pe mujhe aisa kuch nazar nahi aaya ek dafa mujhe masla hua tha na

RA: kia masla hua tha

NA: matlab jaise bache ki delivery hoti hai na us k baad phir joh hota hai na andar bahar ajaata hai na us ka mein ne ja k doctor se zikar kiya tha poocha tha

RA: hmm

NA: toh unhon ne bas yehi mujhe bataya k joh hai na hojaata hai tumhara bacha hua hoga us mein zor laga hoga us se koi tension ki baat nahi hai hoga toh pressure se karwaana parega us se set hoga warna aise nahi hosakta

RA: hmm

NA: toh yeh hai na us k baad phir mein ne zikar nahi kiya yahan pe is ka treatment hai nahi

RA: aap phir kahin aur gayin dikhaane

NA: haan us k baad mein ne kahin aur check karaya

RA: phir hogaya sahi

NA: haan sahi hai abhi hua nahi hai

RA: acha toh aap log kuch cheez mehsoos kar leiti hain yeh yahan par toh baat nahi honi wali nahi hoti toh aap log us cheez ka zikar nahi karte jaise aap ko laga k doctr k paas bhi gayi aur aap ne apna masla share kiya koi aap ko tassali baksh jawab nahi mila

NA: nahi mila

RA: acha toh mein yeh chah rahi hun na k jaise aap ne yeh apna masla bataya

NA: jee

RA: is tarhan se aur bhi cheezein hoti hain

NA: jee

RA: toh un k hawale se agar aap ko worker maloomaat dein toh kia cheez hai joh aap deina chahoge maloomaat k bhae chalo ghar arahi hai toh ek do cheezein yeh bhi hamein bata dein

NA: woh hamein maaloom hoga toh hum zahir si baat hai hum pick kareinge

RA: haan lekin koi cheez aisi hai joh abhi aap ko pareshan kar rahi ho koi sehat k hawale se aap chahti ho k mujhe is k baare mein pata hona chaiye koi aisi cheez

NA: abhi toh filhaal toh koi masla nahi sab thek hai

RA: aur bachon ki sehat k hawale se

NA: bache bhi mashaallah sab thek hai aur inko koi aisa masla nahi hai

RA: lekin kabhi agar bimaar hojaate hain kia cheez aap logon ko lagti hai k yar yeh hojaati

NA: nahi woh ghar mein hi rest kar leite hain ziadatar kia nazla zukhaam bukhaar yeh hota hai Allah ka shukar koi aisa masla nahi hota

RA: acha aur koi cheez joh hum in ko behtar kar sake community k andar aap k liye koi cheez joh aap chahte ho k haan yeh hojaaye toh achi baat hai hamein facility mil jaayegi

NA: yehi hai hum log toh yehi chahte hain k baaki joh poore 9 maheeney tak yeh karti hain pehle maheeney se le k 9 maheeney tak poora yeh le k chalti hain aage bache ki delivery ka bhi hona chaiye yeh bhi intezaam karna chaiye in ko yahan end time pe us ko chor diya un ka sath

phir kisi aur hospital mein jarahe hain thora riyaait karwa rahe hain toh is kamatlab hamara yeh kehna hai kyun k in ka bhi intezaam hona chaiye

RA: delivery ka bhi hona chaiye

NA: haan delivery ki facility honi chaiye is ka bhi intezaam karna chaiye aur jaise bache ko feed k hawale se hota hai jaise mere sar mein dard hai k nahi hai koi aur masla ho maa ko bache ko us k liye bhi thora bohat agar kar lein toh woh bhi sahi rahega acha rahega

RA: acha NA kabhi aisa laga hai k arahi hain aap se data le k jarahi hain aur aap ko koi faida nahi horaha kabhi aisa mehsoos kiya hai aap ne

NA: nahi joh kiya hai woh acha kiya hai faida hua hai

RA: hmm aisa kabhi woh nahi hua thek hai bas ek delivery ka aap ko lagta hai woh nahi hosakta ghar beith k woh nahi karwa sakti CHW center ka center pe hi hoga

NA: jee

RA: thek hai thank you so much aur kuch aap kehna chahti hon koi raaye koi mashwara

NA: nahi sab thek hai

RA: thek hai thank you so much……

IE-IDI-03

RA: acha Assalam o Alaikum ap kiya umer hai?

MR: 40 saal

RA: 40 saal hai acha aur aap ki shadi ko kitna arsa hogaya hai

MR: meri shadi ko 22 saal lagega 20 saal ka beta hai 21 saal kar lein

RA: acha kitne bache hain aap k

MR: 4

RA: 4 bache hain sab se chote bache ki kia umar hai

MR: 11 saal

RA: 11 saal hai thek hai acha yeh mujhe bataayein k joh CHWs hain acha aap ki taleem kitni hai

MR: mein ne nahi parha hua

RA: parha hua nahi hai aur ghar k kaam k elawa koi aur kaam karti hain

MR: nahi

RA: joh hamari CHWs hain woh aap k ghar pe kitne arsey baad aati hain

MR: us ka toh mere khayal mein visit hot eek maheeney ka aisa hoga ya 15 din ka hai jaldi ka hota hai itna woh nahi hai mere khayal mein shayad ek maheeney ka hai

RA: ek maheeney baad aati hain

MR: jee

RA: acha aur jab woh aati hain toh kia kaam karti hain

MR: bas maloomaat karti hain naam poochti hain un k paas computer hota hai jab woh naam leiti hain toh aage se hum bolte hain hum hi hain aap ki apni family hai shadi shuda geir shadi shuda hain hum ne kaha nahi bas filhaal toh apni families k baare mein bataya pooch k bas chali jaati hain

RA: acha aur bachon k hawale se koi kaam karti hain koi sawal karti hain

MR: nahi bachon k baare mein kuch nahi poocha

RA: sirf aap se poochti hain aap k hawale se poochti hain aur kia kia poochti hain

MR: bas yehi poochti hai naam entry hota hai toh us k baad bachon k liye poochti hain joh bache mere huye hain un k baare mein poochti hain aap ki apni family hai koi dusri mein ne kaha nahi bas itni si maloomaat hoti hai is k elawa nahi

RA: is k elawa kuch nahi acha toh yeh sab pooch rahi hoti hai toh aap ko kaisa mehsoos hota hai aap ko kaisa lagta hai

MR: mujhe toh shayad sehat k baare mein koi jaise elaaj k liye kar rahi ho is wajha se pooch rahi hain kabhi hum ne poocha bhi nahi joh woh cheez poochti hain hum bhi jawab de deite hain

RA: aap ne nahi poochi

MR: nahi poochi

RA: wajha is ki

MR: nahi poochi bas hamein toh kabhi is cheez k baare mein pata nahi hai na

RA: phir jab woh aap se pooch rahi hoti hain k aap k ghar mein koi aur shadi shuda toh nahi hai aap k hamal wagera k baare mein poochti hain

MR: hamal se poochti hain koi chota bacha hua toh nahi hai

RA: hmm

MR: yeh baatein poochti hain

RA: acha toh aap un ko bata deite ho aur aap sirf sochti hain aap k bhae hamare sehat k hawale se kuch kar rahi hongi lekin kabhi aap ne unse poocha nahi

MR: nahi

RA: wajha kia hai is baat ki k kyun nahi aap ko mehsoos hua k mujhe toh is se toh poochna chaiye yeh sawal mujhse kyun pooch rahi ho

MR: mujhe shayad aage se hamal wali aurtein in ka hi chal raha hai k joh shadi shuda bache bare hogaye jin ka hamal hai jin ka elaaj chal raha ho us k baare mein entry hoti hai is wajha se mein ne kabhi poocha nahi

RA: acha toh aur kia wajha hai ek toh yeh aap ne kaha aap ko lagta hai hamal wali jin k chote bache hain un k hawale se yeh sawal jawab karti hain lekin aap ko kia lagta hai yeh jab aap se pooch rahi hoti hai toh aap kyun inko apni saari maloomaat de deity hain kia wajha hai

MR: hamein toh yeh lagta hai hamare liye acha hai elaaj chal jaayega

RA: hmm

MR: kuch behtar hoga hamare liye aga khan behtareen hai

RA: acha kisi idaare ka naam leite hon toh us se farq parta hai aap logon ko k aga khan se arahi hain hum se pooch rahi hain

MR: jaise hum aga khan jaate hain joh mere bache hain pehle un ka elaaj hota tha le k jaati thi toh dawaai wagera mili toh yeh mujhe acha laga k yeh idaara acha hai hamein hosakta hai aage achi dawaaiyaan hojaaye

RA: hmm

MR: medical mil jaaye ya kuch hojaaye is liye hum bata deite hain

RA: kabhi mila hai yeh sab cheezein aap ko

MR: bas yeh jab bacha mera chota tha us ka 5 saal tak ka tha usay jab masla hota that oh le jaati thi dawaaiyaan de deity thi is waqt mera bacha ka masla behrapan ka hai padaishi

RA: hmm hmm

MR: waise bahar toh mein ne bohat dikhaya hai lekin abhi us ka joh hai na joh proper elaaj kaano ka masla hai

RA: hmm

MR: toh isi liye mein shayad apna itna afford nahi kar sakti mein indus bhi gayi thi indus se call mila is baare mein maloom kane k liye call karte rahe nahi kuch hua

RA: hmm

MR: toh abhi is ka yeh hai k doctor keh rahe hain waise is ki umar hogayi hai abhi mashaAllah parhai mein daala hua hai government school mein

RA: hmm

MR: ghar walon ne manah kar diya abhi mein yehi chah rahi thi toh isi wajha se yeh soch k k hosakta hai hamara elaaj inse acha hojaaye is mein bachon ki bhi entry hai kia pata elaaj mil raha ho

RA: hmm acha aap ko inka rawaaiya kaisa lagta hai

MR: bare ache tareeke se milti hai jaise aap mujhse baat kar rahi hain mein aap se kar rahi hun ache tareeke se milti hain

RA: ache tareeke se baat karti hain

MR: bas gate pe aati hain

RA: hmm

MR: phir gate pea aye gate pe khare ho k poocha phir bata diya bas itna hota hai

RA: aur aap ko lagta hai k aap k elaake ki hoti hain ya kahin aur se aati hain aap pehchante ho aap jaante ho

MR: nahi kabhi zehan pe nahi liya aaj kal toh pata hai joh aati hain shuru se wohi arahi hain

RA: wohi aati hain aap unko jaante ho k haan yeh aati hain

MR: haan pehchan mein aati hain

RA: haan

MR: joh bachon ki entry wali unse bhi achi salam dua aati rehti hain yeh jaise aap abhi bata rahi hain entry karne aati hain woh pehchan mein aati hain

RA: matlab k wohi aati hain chahe elaake ki hon lekin poorani arahi hain aap log un ko pehchaante ho haan yeh CHWs hain acha yeh mujhe bataayein k koi zaban ki agar CHW ho matlab aap ki hum zaban ho mere khayal se aap Punjabi hain

MR: Hindko bolne wale

RA: Hindko bolne wale hain agar woh ho toh ziada faida hoga ya koi urdu speaking aap se pooche

MR: nahi urdu nhi samjh leite hain hamare Hindko zaroor hai lekin ghar pe hai na shuru se urdu bachon mein hamare bache saare urdu mein baat kareinge

RA: hmm

MR: apni zaban mein koi bhi nahi kareiga

RA: toh urdu araam se aap ko samjh ajaati hai un ki baat woh sawal karti hain woh aap ko araam se samjh ajaati hai

MR: joh woh sawal kareingi joh samjh mein aaya jawab de deinge

RA: thek hai acha mujhe yeh bataayein jab aap aakhari dafa hamal se huyin toh kia us mein CHW ne koi kirdaar ada kiya tha

MR: meri us waqt shadi huyi thi yaad nahi araha kaafi time hogaya hai

RA: hmm

MR: lekin bache ko mein wahan dikhaati thi bache ka unhon ne 5 saal tak k kaha us k baad mein nahi gayin

RA: jab aap hamal se thi tab

MR: nahi hamal se mera nahi tha

RA: toh koi doctor ko nahi dikhaya aap ne

MR: jinnah mein hi hua bas jinnah mein is ka masla joh tha kar diya

RA: jinnah mein kar liya tha us waqt aap ne nahi dikhaya koi worker wagera nahi aayi thi aap k paas

MR: nahi worker nahi aayi thi

RA: thek hai aur bache k hawale se 5 saal tak aap bache ko dikhaati rahi hain

MR: jee

RA: hispatal mein acha yeh idhar center pe dikhaati rahi

MR: jee yeh joh hyderi mein hai na wahan pe dikhaati rahi

RA: wahan pe dikhaati rahi wahan pe us se aap mutmaain hai

MR: mere toh kaafi time hogaya yeh bache bohat kam jaate hain abhi is ki padaaish huyi hai yeh joh bachi hai meri bhatiji is ko 3 4 dafa aayin visit mein deakha hai lekin center par le k nahi gaye

RA: lekin is ko ghar a k deakh rahi hai

MR: haan ghar a k bache ka wazan le rahi hai deakh rahi hain

RA: hmm hmm

MR: sab kar rahi hain abhi in ki tabiat sahi nahi khaansi horahi hai bachi ko mein ne apni bhaabi ko bola aap jao aap ka naam hai bachi ka hai is ko dikhaao

RA: hmm

MR: bas yehi hai

RA: acha aap ko lagta hai k yeh joh worker aap k ghar par aati hai is ko munasib training mili huyi hai tarbiat hai is ki k bache ko is ka wazan sahi kar rahi hai ya aap se joh sawal kar rahi hai is ko sahi

MR: hamari woh poorani aati hai us ka naam nahi aata woh achi hai woh aati hai jab bacha hota hai na abhi toh un ka naam nahi yaad woh aati hai isi tareeke se check karti hai ache tareeke se check karti hain

RA: toh aap ko lagta hai k un ko munasib taaleem mili huyi hai un ki tarbiat matlab unko mazeed hamein trained karne ki zaroorat hai ya joh sawal aap se poochti hai aap k baare mein who thek tarhan se poochti hain ya unko mazeed zaroorat hai hum aur ache se unki training karein

MR: is k baare mein kuch keh nahi sakti

RA: aap ne kabhi socha hi nahi is baare mein worker a rahi hai pooch rahi hai acha yeh bataayein k center se jorne mein koi kirdaar ada kiya worker ne joh aga khan ka center hai

MR: jorne ka matlab

RA: matlab k aap ko udhar le kar gayin wahan aap ko bataya ho k bhae center hai yeh saari facilities milti hain is tarhan se bataati hain jab aati hain toh

MR: is tarhan se mere sath koi baat nahi huyi

RA: lekin aap ne kaha na jaise bache ko 5 saal tak center pe dikhaati rahin toh woh kaise aap ko pata chala k center hai

MR: woh toh jab yeh aati hai na baari aati hai na center ki hamare elaake mein toh woh aati hain hamal se pehle aati hain maloom karti hain

RA: hmm hmm

MR: phir jin ka shuru shuru ka hamal hota hai teesra maheena hota hai phir bataati hain k hum ultrasound bhi karte hain bachon ka entry hojaata hai phir jab normal case ho toh shayad sunne mein aaya ab toh cases bhi horahe hain

RA: hmm

MR: ab yeh maloomaat mili hai k case bhi horahe hain

RA: yeh sunne mein kis se aaya

MR: yeh toh jaise meri bhaabi ne bataya k aga khan mein ab yeh facility hone lagi hai

RA: acha

MR: jab is ki mama ka ultrasound hua tha shuru mein thi dusri bhaabi mehwish us ka toh 5 maheena tha manah kar diya k aap ka shayad nahi hoga phir hum ne jana chor diya jinnah mein karwa liya tha

RA: toh yeh saari cheezein joh bataai kisi worker k through pata chali ya aas paros se pata chali

MR: yeh inse mein ne poocha in ka nahi pata kaise pata chala

RA: acha yeh mujhe bataayein k aap ko lagta hai k hum in k kaam ko mazeed kaise behtar bana sakte hain aisi kia maloomaat hai jaise aap ne ek baat kahi thi k mujhe aisa lagta hai k hamal wali aurtein aur chote bache unhi k hawale se kaam karti hai toh aap ko lagta hai k koi cheez aisi yeh karein k aap ko koi faida ho joh abhi hamal se nahi hai aurtein woh kia cheez hain kia kar sakte hain

MR: jaise hamal k elawa dusre masle hote hain ladies k bhi hain waise bhi koi takleef hai un ka bhi elaaj toh ho us k baare mein kabhi poocha bhi nahi na hamein pata chala

RA: kaise koi ek aat masle k baare mein batayeingi k joh aap ko lagta hai in k baare mein toh hamein batana chaiye ya poochna chaiye jaise aap ne kaha na ladies k sau masle hain koi ek aat joh aap ko lagta ho haan is k baare mein mujhe CHW se poochna bhi chaiye

MR: aaj kal joh mahwaari ka masla chal raha hai bacha daani ki aant us k baare mein ladies ko is baare mein kabhi mein ne poocha bhi nahi na kabhi bataya hai bas mein ne suna bachon k baare mein bachon ka hi horaha hai bachon ko hi le jarahe hain

RA: haan sahi hai thek toh aap yeh keh rahi hain mensis wagera k hawale se ya joh bacha daani k joh masle joh hojaate hain un k hawale se hum se poochein hamein maloomaat dein is k elawa koi aur cheez joh aap ko apni sehat k hawale se lagti ho k bhae mujhse yeh poochein arahi hain toh mujhe thori si ehmiyat dein koi aisi cheez hai joh aap k zehan mein aati ho

MR: bas filhaal toh aur kuch nahi jaise yeh hamara bacha hai in ki entry horahi hai unka elaaj acha hojaaye

RA: hmm bas yeh hojaaye

MR: haan

RA: elaaj acha hojaaye aur bachon k hawale se koi mazeed maloomaat agar woh dein toh kis pe dein

MR: woh toh pehle file ban gayi thi na bachon ki

RA: hmm hmm

MR: ab in ki mein ne poocha in ki bata rahi hain bhaabi aisa kuch nahi hua mein ne kaha aap ki entry us mein huyi hai toh le jao

RA: hmm hmm

MR: wahan pet oh hai entry is ki

RA: acha aap k jaise bare bache hain toh aap zaroorat mehsoos karti hain k yeh toh bhae 6 maheeney se 5 saal tak k bachon ko deakhti hain aap k bache bare hogaye hain aap ko lagta hai un k hawale se bhi kuch poochna chaiye karna chaiye jaise 5 saal se bare hogaye hain na bache woh nahi poochti kyun k teeke wagera nahi lagte honge na us umar mein 5 saal tak lagti hai toh aap ko lagta hai k kyun k bache toh woh bhi hain un ki apni sehat hai toh us k hawale se koi cheez joh aap ek bare bache ki maa hain toh aap ko lagta hai mere bache ki bhi sehat k hawale se agar hamein ghar beith k mujhe koi maloomaat mil jaaye ya kuch pata chal jaaye toh mere liye asaani hojaayegi kuch aisa lagta hai aap ko

MR: kabhi yeh is baare mein socha nahi hua nahi zehan mein kabhi aaya nahi

RA: kabhi nahi aaya

MR: nahi

RA: kabhi nahi socha bache bimaar wagera hote hain

MR: haan koi na koi bachon mein masla hojaata hai zahir si baat hai doctor se dawaai likhwani parti hai woh masla toh insaan k sath laga hota hai

RA: hmm hmm toh koi cheez aisi joh worker k through woh hum hal kar sake koi matlab pehle se maloomaat hojaaye

MR: hum jaise aur toh koi masla nahi Allah ka shukar hai sab thek hain mein ne apne chote bete ka bataya us ka masla hai kaan ka toh mein toh yehi chah rahi thi k aisa koi ho joh mere bache ka elaaj ho

RA: hmm hmm hmm jaise bare specials bache hain toh un ka elaaj bhi ho

MR: jaise sau masle hote hain bachon mein aise bachon ka koi elaaj aga khan mein hojaaye us k liye hum kehte hain k hamein thori sahulat mil jaaye

RA: acha chalein thek hai aur koi cheez joh aap batana chaheingi

MR: nahi

RA: chalein thank you……

IE-IDI-

RA: Assalam o Alaikum aap mujhe bata sakti hain aap ki umar kitni hai

Interviewer: 19

RA: 19 saal hai aur aap k kitne bache hain

Interviewer: 3 betiyaan hain

RA: 3 betiyaan hain acha sab se pehle mein aap se yeh poochna chahungi k health worker aap k ghar aati hai

Interviewer: jee aati hain

RA: acha toh kitne arse baad aati hain

Interviewer: ek maheena 15 20 din baad aati rehti hain

RA: acha aur jab woh aati hain toh woh kia kaam sar anjaam deity hain jab woh aati hain toh kia poochti hain kia karti hain

Interviewer: interview leiti hain woh bhi humse

RA: hmm

Interviewer: tabiat wabiat poochti hain bache ki meri 5 maheeney ki bachi hai us ki tabiat poochti hain wazan karne aati hain wazan bhi karti hain

RA: thek hai joh yeh aap ki tabiat poochti hain us mein kia kia poochti hain

Interviewer: aap ko nazla wazla toh nahi hai zukhaam wagera toh nahi hai bukhaar wukhaar toh nahi hai

RA: hmm

Interviewer: meri beti ki maloom karti hain bachi ki bataati hun bukhaar wukhaar toh nahi hai

RA: hmm

Interviewer: konsa doodh pilaati hain

RA: acha

Interviewer: har cheez poochti hain

RA: aur aap k hawale se is k elawa kia poochti hain

Interviewer: aur toh kuch nahi poochti

RA: aap ki sehat k hawale se

Interviewer: meri sehat k hawale se poochti hain

RA: kia poochti hain

Interviewer: kia khaati ho koi acha khaaya karo fruit wagera bata k jaati hain

RA: acha acha

Interviewer: bas

RA: aur hamal ya is tarhan ki koi cheez poochti hain

Interviewer: nahi

RA: hamal se hain k nahi hain aisa kuch

Interviewer: nahi

RA: aisa kuch nahi poochti acha aur bachon k hawale se bachi k hawale se kia poochti hain

Interviewer: bachi k hawale se yeh poochti hain bachi ki care karo

RA: hmm

Interviewer: bachi ka wazan check kar k jaati hain

RA: hmm

Interviewer: bachi ko doodh woodh upar ka bhi lagaao aise bolti hain

RA: acha ek toh woh aati hain alag alag aati hain

Interviewer: alag alag aati hain

RA: joh wazan karti hai woh alag hoti hain

Interviewer: alag hoti hain

RA: acha

Interviewer: alag alag se aati hain matlab woh saari hoti hain alag alag se aati hain har cheez k liye

RA: thek hai

Interviewer: jaise woh ho kar gayin hain kal koi aur araha hai

RA: toh joh aap k ek maheeney baad ya do maheeney baad aati hain us hisaab se aap k hawale se ya kam umar like 5 saal se chote bache

Interviewer: in k woh le k jaati hain

RA: haan woh kitne arsey baad aati hain

Interviewer: 15 20 din lag jaata hai

RA: acha yeh mujhe bataayein aakhari dafa jab aap hamal se huyin thi toh us mein yeh health worker hai us ne koi kaam sar anjaam diya tha

Interviewer: nahi

RA: kuch nahi kiya kaise

Interviewer: bas kia naam hai bol rahi thi mera naam likha hua tha

RA: hmm

Interviewer: lekin keh rahi thi k teesre maheeney mein joh likhwata hai us ka wahan case hota hai

RA: hmm

Interviewer: aur mera toh mashaallah ghar mein hi case hua tha meri ammi dukaan mein joh beithi huyi hai unhon ne kara tha

RA: hmm

Interviewer: ghar mein hi huyi thi normal masla ban gaya tha 5 number gayi thi card nahi bana hua tha lekin wahan ka na corona chal raha that oh jaane nahi diya kisi ne bhi na hispatalon mein

RA: hmm

Interviewer: toh is liye bohat mushkil hogayi thi lekin 2 3 din lag gaye thy bas ghar mein hi na bohat takleef se thi ghar mein hi hogayi thi

RA: acha toh aap ne us doraan yeh joh CHW hai health worker unse contact nahi kiya

Interviewer: nahi

RA: aap k paas inka number hai

Interviewer: haan tha inka number

RA: hmm

Interviewer: ek dafa interview le kar gayin thi toh woh aisa parcha tha

RA: hmm

Interviewer: number wumber likhawa tha unka

RA: hmm

Interviewer: bas

RA: toh aap ne unko phone nahi kiya

Interviewer: phone kiya tha

RA: hmm

Interviewer: jab woh keh rahi thi jab munni hojaayegi na aap ka kuch hojaayega na toh aap phone kar deina ek do din baad hum na woh koi pata nahi kia chal raha tha bache ka na aant ka woh check kareinge pata nahi kuch check karne k obol rahe thy

RA: hmm

Interviewer: oxygen wagera kuch lagega

RA: hmm

Interviewer: aur kuch nahi hoga darna nahi yeh sab bol kar gaye thy

RA: acha toh aap k hamal k hawale se ya delivery k hawale se koi baat nahi ki

Interviewer: nahi

RA: aap ne unhein bataya k dar horahi hai aisa kuch horaha hai

Interviewer: nahi yeh nahi bataya

RA: aap ne nahi bataya aap ne kyun nahi bataya

Interviewer: bas who itni habbar dabbar lagi huyi thi aur phir sunne mein yehi araha tha k case nahi karte teesre maheeney se naam likhte hain toh karte hain waise nahi le k jaate

RA: acha toh aap ne yeh kis se suna k teesre maheeney se

Interviewer: meri behan hai ruksaar 51 C mein rehti hai unhon ne bhi yahin naam likhwaya hua hai aga khan mein teesre maheeney mein naam likhwaya tha toh isliye kia naam hai us ka udhar case hua hai ab mashaallah unka beta hua hai aaj 10 din ka hogaya

RA: hmm

Interviewer: toh who bhi bata rahi thi k kia naam hai doctor ne bhi teesre maheensy mein naam likhte hain na toh case hota hai phir hispatal apne le jaate hain woh log toh isliye mein dar gayi thi

RA: acha

Interviewer: toh isliye na phone kara na baat kari faida nahi hai na koi

RA: lekin agar aap health worker se poochti aap ka nahi khayal who aap ko behtar bata deity k hota hai teesre maheeney mein aisa kuch aisa nahi socha aap ne

Interviewer: nahi aisa nahi socha meri tabiat itni kharab hogayi thi meri ammi bata rahi thi tumhara operation hote hote bacha naal mein na mera bacha phassa hua tha

RA: acha

Interviewer: warna mere 2 joh bache huye hai na meri 2 betiyaan joh hain ziada time nahi laga tha ab teesri beti men na 2 din poora din laga diya tha

RA: aur woh dono bache aap k ghar par hi huye

Interviewer: haan teeno bache ghar pe huye

RA: acha toh yeh health worker aap k paas aati hain us ko batati hain k aap hamal se ho

Interviewer: nahi

RA: woh aap se poochti nahi

Interviewer: poochti hain toh bata deity hun

RA: toh tab woh aap ko mashwara deity hain k aap hospital se delivery karwaayein

Interviewer: haan bola tha laraib ki dafa meri choti beti thi phir mein gayi thi naam likhwaane

RA: hmm toh us waqt likhwaaya tha naam

Interviewer: haan ultrasound wagera bhi hua tha sab hua tha

RA: toh tab bhi aap ne hospital se delivery nahi karwaai

Interviewer: nahi

RA: wajha

Interviewer: bas

RA: kia wajha kia hai

Interviewer: nahi koi wajha nahi hai bas aise hi jab mere ko pata chala na kia naam hai teesre maheeney se hota hai wahan pe waise naam toh likha jaata hai lekin na woh nahi hota case nahi hota

RA: hmm aap hamal k doraan gayin hain apna checkup karwaane k liye

Interviewer: haan checkup karwaane meri choti beti checkup karwaane gayin thi khoon test hua tha mera ultrasound wagera hua tha

RA: acha toh aap ko center k baare mein kis ne bataya

Interviewer: center k baare mein toh kisi ne nahi bataya tha meri bhaabi hai na sobia bhaabi sobia Naveed unhon ne mujhe bataya mein un k sath hi gayi thi center

RA: acha toh wahan par bhi aap ko guide kiya k bhae kitne maheeney pe hona chaiye ya aap ne khud se poocha delivery joh hai who mein kaise karaaon

Interviewer: nahi mein ne ek dafa poocha tha toh keh rahe thy teesre maheeney mein naam likhte hain na

RA: acha.. acha yeh mujhe bataayein k jab woh worker aati hai toh matlab aap se pooch liya k hamal se ho k nahi ho is k elawa kuch bataati hai

Interviewer: nahi is k elawa nahi

RA: center k hawale se ya kuch

Interviewer: nahi

RA: nahi bataya tha kabhi aap ne unse khudse poochne ki koshish ki hai ya joh woh sawal poochti hain us ka jawab deity hain

Interviewer: bas sirf us ka jawab deity hun

RA: mujhe ziada nahi pata na bas meri choti bachi hai us ka naam likhwaaya tha

RA: hmm

Interviewer: dono betiyon k jinnah mein naam likhwaaya tha bas

RA: hmm toh aap yeh nahi sochti jab aap pocheingi toh aap ko matlab

Interviewer: haan bas itni woh hogayi hai inshaallah woh din aayega jab mein unse poochungi mujhe pata nahi tha pehli pehli dafa

RA: hmm acha waise jab yeh aati hain bachi ka checkup karne toh aap unse sawal karte hain kuch poochte hain k woh joh data le rahi hain ya joh pooch rahi hain woh kyun le rahe hain kis ki taraf se aaye hain

Interviewer: nahi woh toh meri bhaabi ki 2 betiyaan hain mashaallah un ka wahin naam likha hua tha ammi ne kara tha case lekin unhon ne mujhe bataya tha yeh hispatal se aati hain aga khan se aksar har koi araha hota hai matlab bohat saari hoti hai na koi na koi araha hota hai

RA: hmm

Interviewer: jaise aaj aap aayi ho na kal koi aur araha hai

RA: hmm

Interviewer: aise aate rehte hain

RA: aate hain poochne k liye

Interviewer: haan poochne k liye aate hain meri bhaabi ki 2 bachon ko mashaallah bari hogayi hai na toh woh chocolate wagera woh nahi hoti jis se taaqat banti hai chocolate woh de k jaati hain meri bhaabi ki betiyon ko juice saabun har cheez de k jaati hain

RA: acha toh aap log matlab itne saare log aate hain yeh poochne k liye toh aap ko aisa nahi laga k kyun arahe hain

Interviewer: nahi mashaallah bohat ache hain khayal karte hain hum deakhte hain na bhaabi ki betiyon ko bohat khayal karte hain

RA: hmm

Interviewer: har cheez karte hain

RA: hmm

Interviewer: haan woh bhi corona jab chala tha naak mein lakri daal k pulgum nikaal deite hain mashaallah sab cheezein karte hain bohat ache se karte hain

RA: acha yeh mujhe batao k health worker mein joh aap ki choti bachi hai joh 5 maheeney ki hai us ki sehat k hawale se koi kirdaar ada kiya ho kuch kiya ho

Interviewer: mashaallah jab meri beti huyi thi na 11 din ki thi mashaallah sahi thi

RA: hmm

Interviewer: mashaallah tandroost thi 11 din ki toh woh wazan check karte rehte thy height bhi mashaallah bohat achi hai iska wazan bhi bohat acha hai is ka khayal rakhna bohat ziada

RA: thek hai acha aap ko lagta hai k yeh joh aati hain inko munasib tarbiyat haasil hai yeh joh baat karti hain aap se poochti hain inki parhaai inki taleem itni hai k sahi kaam kar rahi hain aisa lagta hai aap ko

Interviewer: haan sahi tha mashaallah har cheez ka khayal karti hain bohat acha khayal karti hain

RA: hmm

Interviewer: joh bhi aata hai na mashaallah khayal bohat karte hain

RA: aap ko kia cheez unki sab se achi lagti hai

Interviewer: unki aadat unka har cheez karti hain mashaallah bache ka wazan check har cheez abhi joh corona chal raha tha har cheez kar k jaati hain bachon ko khush kar k jaati hain

RA: aur baat cheet mein kaisi lagti hain

Interviewer: sahi lagti hain mashaallah

RA: baat cheet sahi karti hain

Interviewer: haan

RA: aap ko unki zaban samjh mein ajaati hai

Interviewer: haan thori bohat ajaati hai

RA: aap urdu speaking hain ya sindhi hain

Interviewer: urdu

RA: urdu hain toh urdu mein baat karti hain aap se

Interviewer: urdu mein karti hain

RA: acha aap k khayal mein agar aap ki hum zaban hon urdu bolne wali ho ya sindhi toh us se koi fark parta hai

Interviewer: nahi

RA: nahi parta yeh joh aati hain ghar pe aap k elaake se hoti hain

Interviewer: idhar se hi hoti hain

RA: kahan se hoti hain

Interviewer: aga khan se

RA: aga khan se aap k elaake mein nahi rehti

Interviewer: nahi

RA: toh is se fark parta hai aap k elaake ki hon ya kuch

Interviewer: nahi nahi is se fark nahi parta is liye mein toh pehli dafa gayi thi udhar meri bhaabiyon k otoh 2 3 saal hogaye chakkar lagte rehte hain unhon ne hamein bataya hua hai deakho bharosa bhi ek cheez hoti hai unki aadat mashaallah bohat achi hai

RA: toh aga khan k naam se un pe aitemaad karte hain ya unki apni wajha se hi jis tarhan se kaam karti

Interviewer: nahi aga khan k hispatal se

RA: aga khan k hispatal se is se fark parta hai k aga khan se aayi hain

Interviewer: haan

RA: kyun

Interviewer: bas haspatal se har koi bolta hai sahi hai mashaallah acha hispatal hai bohat khayal rakha jaata hai isliye ek bharosa hota hai nahi waise woh bhi bohat achi hai

RA: hmm

Interviewer: unki aadat bhi achi hai jab bhi aayi hain koi aisi waisi baat nahi hai acha woh kar k jaati hain bachon ka bhi hamara bhi sab ka acha kar k jaati hain haspatal bhi acha hai

RA: wahan gayin hain aap yeh joh center hai hamara

Interviewer: haan gayi hun

RA: acha center se jorne mein koi kirdaar ada kiya CHW ne joh worker hain

Interviewer: nahi

RA: nahi toh kaise pata chala center ka

Interviewer: meri bhaabi le kar gayi thi

RA: bhaabi le kar gayin thi bhaabi ko kaise pata chala

Interviewer: meri bhaabi ko aaj se 4 5 saal toh hogaye shayad meri sahar huyi thi Naveed sobia ki beti us ki dafa mein bhi naam wahin likhwaya

RA: hmm

Interviewer: aur us se pehle joh hameeda aati hai na hameeda yousuf joh bewa hogayi hain meri behan hai woh unhon ne bataya shayad bhaabi ko meri

RA: acha toh jab worker aati hai yeh saare sawal karti hai toh woh bolti hai hamara center hai is pe bache ka checkup hota hai is tarhan ki koi baat karti hain aap se aise bataati hai aap ko

Interviewer: haan bataati hain

RA: acha koi number wagera ya is tarhan ki koi cheez share karti hain aap se

Interviewer: haan number maangti hain jab meri munni nahi huyi thi tab apna number de k gayin thi koi masla hojaaye toh toh aap hamein phone kar dein aur aap ko hojaaye toh ek do din baad phone kar dein bache ko check kareinge

RA: thek hai sahi toh yeh joh aap se poochti hain aap unhein bata deite ho phone kar k haan meri bachi hogayi hai us ko check kar lein

Interviewer: haan mein ne bataya tha lekin woh aayi nahi thi chuttiyon pe koi gaya hua tha us doraan chuttiyon pe gaya hua tha tab mein ne phone kara tha us dafa chuttiyon pe gaye

RA: lekin aap ne unko phone kiya tha

Interviewer: mein ne phone kiya tha

RA: acha.. acha yeh batao k aap ko lagta hai k is kaam ko joh health worker aap k ghar arahi hai aap se pooch rahi hai is ko hum mazeed behtar bana sakte hain joh aap ki sehat k hawale se joh aap ko kuch lagta ho k worker arahi hain is se mujhe thora faida hojaaye

Interviewer: bas sochte toh hain lekin woh Allah ki marzi hai joh honi hai

RA: kia kia sochti hain

Interviewer: bas yehi abhi is k papa gir gaye thy mein ne bataya tha

RA: hmm

Interviewer: jaise nida joh bewa hai na unko raashan deina shuro kar diye thy toh mere hai na husband teesri manzil se gir gaye thy toh un ki qamar pe lag gayi thi toh unse na utha ja raha than a beitha ja raha tha kahin bhi nahi hai

RA: hmm hmm lekin yeh joh sehat ka idaara hai sehat k hawale se kisi cheez ki madad chaiye ho koi maloomaat chaiye ho toh aap ko kia lagta hai kia cheez

Interviewer: poochna chaiye mashwara karna chaiye

RA: kis cheez k baare mein

Interviewer: sehat k hawale se

RA: unko koi maloomaat aap ko deini chaiye jab woh aati hain sirf aap se poochti hain kuch batati bhi hain aap ko

Interviewer: haan bataate hain

RA: kia bataati hain

Interviewer: yehi bataati hain waise toh bachi k baare mein poochti hain

RA: hmm

Interviewer: ziadatar

RA: bachi k aap k hawale se

Interviewer: mere hawale se poochti hain

RA: acha toh aap ko lagta hai aap k hawale se poochna chaiye

Interviewer: bas yeh lagta hai bachi k hawale se pooch rahi hain toh thek hai

RA: haan apne baare mein nahi sochti lekin aap ko lagta hai k matlab woh a hi rahi hain

Interviewer: toh pooch lein

RA: haan toh kia poochein aap ko apni sehat k hawale se kia aisi cheez hai joh lagti ho k haan yeh arahi hai toh mujhe bata dein

Interviewer: yeh jaise k mein paan waan khaati thi toh mujhe doctor bhi manah karti thi k is haalat mein paan waan nahi khao

RA: hmm

Interviewer: bache k liye sahi nahi hai tumhare liye sahi nahi hai yeh sahi nahi hai yeh samjha k jaati thi joh baat hai

RA: haan toh phir aap ne khana chora

Interviewer: bas thora woh hogaya tha lekin is bachi k time pe mera paani nahi chut raha tha warna mere dono betiyon k time chaale paani roti sab chut gayi thi

RA: hmm toh acha aur maloomaat deina chahein toh jaise aurton k masaail hote hain shadi se pehle bhi hote hain baad mein bhi hojaate hain toh un k hawale se koi maloomaat deina chaho

Interviewer: nahi

RA: nahi koi maloomaat aisi nahi joh aap chahti hon k yeh bata dein toh mere liye faida hojaaye

Interviewer: nahi

RA: koi aisi cheez joh aap ko pareshan karti ho sehat k hawale se bache ki sehat k hawale se agar yeh mujhe ghar beithe pata chal jaaye toh mere liye asaani ho aisa kuch hai

Interviewer: nahi

RA: acha aur koi cheez joh batana chaheingi health worker k kaam ko kaise behtar bana sakein kia karein aisa k aap khush hojaayein aur center aayein

Interviewer: nahi khush toh hain hum jab aap bulaayeingi hum ajaayeinge

RA: acha is kaam ko aur mazeed kaise behtar banaya ja sakta hai aap k khayal mein ya yeh sahi hai joh yeh kar rahe hain

Interviewer: joh aap behtar samjhein sahi hoga joh jab aap ka dil ho hum ajaayeinge khushi khushi

RA: acha chalein shukriya bohat bohat…….

IE-IDI-02

RA: SA sab se pehle mujhe yeh bataayein k aap ki umar kitni hai

SA: 30 saal

RA: 30 saal hai acha aur aap ki shadi ko kitna arsa hua hai

SA: 14 saal hone wale hain

RA: 14 saal hone wale hain acha aur aap yahan par kitne arsey se hain

SA: is ghar mein 4 saal se

RA: 4 saal se aur kitne bache hain aap k

SA: mere 5

RA: 5 bache hain acha sab se chote bache ki umar kia hai

SA: 2 saal 7 maheeney

RA: 2 saal acha taleem kahan tak haasil ki hai

SA: mein ne 2 ya 3 parhe huye hain

RA: acha ghar k kaam k elawa aur aise koi kaam karti hain koi aamdani k zariye

SA: meri apni koi aamdani nahi hai

RA: acha mein na aap se apni health worker k hawale se sawal karongi sab se pehle mujhe yeh bataaiyega k yeh joh health worker hai yeh aap k ghar pe kitne time k baad aati hain

SA: haftey mein ek do chakkar laga leiti hain

RA: haftey mein ek do chakkar laga leiti hain acha aur yeh jab aati hain toh aap se kia poochti hain kia kaam karti hain

SA: yeh kabhi jaise k hamara weight check karti hai

RA: hmm

SA: kabhi hamara yeh cheez hamare bachon ki

RA: hmm

SA: wazan ki aur is k elawa jab bimaar hote hain toh center le k jaati hain un ko dawaai wagera deity hain

RA: acha alag alag larkiyaan aati hain

SA: nahi alag alag

RA: alag alag aati hai acha koi aisi larkiyaan hain joh kuch arsey matlab gap de kar aati hain 15 din ek maheeney k baad aati hon

SA: nahi

RA: joh aap se sirf aap k hawale se sawal karti hon like k aap pregnancy se related ya aap k 5 saal se chote bache hain is tarhan k sawal karti hain

SA: haan yeh karti hain

RA: woh kitne arsey baad aati hain

SA: who aajaati hain maheeney mein ek dafa ya do dafa

RA: hmm

SAwoh meri beti ko de k jaate hain

RA: acha thek hai acha toh yeh mujhe batao k woh jab kaam karti hai us se aap ko kaisa mehsoos hota hai

SA: acha mehsoos hota hai

RA: kia acha lagta hai

SA: yeh acha lagta hai hamare ghar beithe hamari madad kar rahe hain aur Allah paak in ko himmat dein

RA: hmm

SA: k bhae hamari aisi madad karte rahein in k kaam mein tarakki dein bohat acha hosake

RA: acha toh aur is k elawa kuch kehna chaheingi k in k kaam k hawale se kaisa lagta hai aap ko

SA: acha lagta hai

RA: acha yeh aap k ghar beithe aap ki madad karti hain aap ko a k deakh leiti hain yeh cheez aap ko achi lagti hai acha toh woh sawal wagera aap se karti hain kuch bata sakeingi kia sawal karti hain aap se joh poochti hain sawal kis hawale se poochti hain aap se

SA: abhi jaise khaansi nazla chal raha hai corona ki wajha se jaise aap ko is hafte khaansi nazla toh nahi hua ya ulti motion toh nahi hai is hawale se sawal karti hain

RA: acha thek thek koi ek team aisi bhi hai joh matlab k ek maheeney k baad aati hai aur ek hi jaise sawal jaise aap hamal se toh nahi hai aap ne matlab aap yahan pe kitne arsey se reh rahi hain aap k ghar mein koi shadi shuda koi shadi ho kart oh nahi aayi hai aisi bhi team aati hai joh

SA: aisi bhi aati hain joh hamare poore ghar ki entry kar k chali jaati hain

RA: haan woh kia poochti hain woh wali team

SA: jaise aap k ghar mein kitne afraad hai

RA: hmm

SA: aap k ghar mein kitne afraad hain shadi shuda kitne hain gair shadi shuda kitne hain chote bache kitne hain hamare ghar shadi shuda 3 afraad hain bachon ka poochti hain bas pregnancy ka poochti hain

RA: un k kaam k hawale se aap ko kia lagta hai kyun k woh aap ko kuch deity nahi hai na woh toh sirf aap ka like interview karti hain toh woh un k kaam k

SA: nahi un ka kaam bohat acha hai

RA: acha aap ko un k kia cheez achi lagti hai health worker k hawale se

SA: un ki bhi cheezein achi lagti hai ghar mein a k hamari kuch toh madad kar rahi hai na agar hum pregnant hain hamein le ja rahi hain khoon test karwaane ultrasound karwaane woh saari zemaidaari unki hai mein aap k sath jaongi toh meri zemaidaari aap k upar hai aap yeh socheingi k meri zemaidaari hai unka ache se checkup karana

RA: hmm hmm hmm

SA: sahi hai in ko joh jagha nahi pata woh inko khud le k jana kyun k woh aap ki zemaidaari hai

RA: acha sahi hai toh mujhe yeh bataayein k unka zaban aap ko samjh ajaati hai

SA: nahi urdu speaking hai

RA: urdu speaking aap bhi urdu speaking hai aap ko lagta hai is se fark parta hai k hum zaban ho toh ziada behtar hota hai ya koi bhi sindhi agar aayegi aur aap se baat kareigi toh

SA: baat kar lungi

RA: aap baat kar leingi toh aap k elaake ki hoti hai workers

SA: nahi hamare elaake ki nahi

RA: acha lekin woh aap k elaake se nahi hai is se fark parta hai aap k elaake ki ho ya nahi

SA: nahi

RA: acha aga khan se aati hain

SA: aga khan se aati hai

RA: is se fark parta hai k aga khan se arahi hain kia fark parta hai aga khan k naam se kia fark parta hai

SA: hamari help kar rahi hain

RA: acha aap ki help kar rahi hain aga khan k naam se fark parta hai aap ko lagta hai k aga khan ka naam hai us ki wajha se aap log data de deite ho aap ko un k upar aitemaad kyun hai

SA: aitemaad hai kitne saal hogaye mujhe mashaallah se 4 5 bache hogaye mein wahin jaati hun bachon ko masla hota hai wahin jaati hun yahan tak k aadha aadha din poora poora din wahan reh k aayi hun mujhe wahan ka mahaul acha laga

RA: aap ko wahan ka mahaul acha laga acha yeh mujhe bataayein k jab aakhari dafa aap hamal se huyin thi toh CHW ne koi kirdaar ada kiya tha jab aakhari dafa aap hamal se huye thy toh aap ko doctor k paas le jaan ya koi aap ko masla hua ho pregnancy k doraan

SA: haan us ko bataya tha le k gayin thi

RA: le k gayin thi aap ne unse kaise contact kiya

SA: ghar pe a k mera naam likh k gayin thi

RA: aap k ghar aayin thi aap ka naam likh k gayin thi

SA: phir bol rahi thin k ek aat hafte mein aap ko le k jaayeinge ultrasound k liye test k liye

RA: acha

SA: toh woh le k gayin thi jinnah bhi le k gayin thi mujhe 2 saal pehle 2.5 saal pehle jinnah bhi le k gayin thi jinnah mein bhi naam likhwaya tha wahan pe khoon test wagera karwaaya tha aur hepatitis c ka bhi test karwaaya tha mera muje le k jaana mujhe le k aana

RA: thek hai toh aap k paas unka number wagera hota hai toh aap un ko call kar leiti hain ajaate hain

SA: a jaate hain

RA: intezaar toh nahi karna parta

SA: nahi

RA: acha mujhe batao yeh jab chota bacha hua us k baad us ki sehat k hawale se kuch kiya ho joh CHW hai bache ki sehat k hawale se jaise aap ko toh le k gayin jinnah mein le kar gaye test karwaaya ultrasound karwaaya bache ka kia kiya

SA: abhi meri choti wali ka unka weight kam hai umar unki sahi hai 2 2.5 saal ki hai is ka weight kam hai ab jab weight kam hota hai us k joh packet hote hain woh khatam hojaate hain mere ko jaana parta hai mein jaati hun toh packet de k chali jaati hain aur abhi khatam hua hai toh dobara de k chali gayin

RA: acha yeh 2 saal ki thi jab yeh 6 maheeney ki thi tab kuch kiya tha jab bilkul choti thi nayi nayi paida huyi thi tab kia kiya tha

SA: inka khoon test hua tha paidaaish se pehle aur ek 6 maah baad wala

RA: acha aur wazan wagera

SA: wazan wagera kar k jaati hain har maheeney

RA: acha sahi hai acha yeh mujhe batao k aap ko lagta hai k yeh joh worker aati hain in ko munasib training haasil hai matlab tarbiat hai inki

SA: tarbiat hai tabhi arahi hai na tarbiat nahi hoti toh

RA: kia cheez lagti hai k haan yeh joh kaam kar rahi hain inko sahi training mili huyi hai kaise pata chalta hai

SA: inka kaam aisa hai

RA: acha baat wagera sahi karte hain acha yeh wazan wagera joh karti hain ya jab aap se aap ka information le rahi hoti hain kuch bataati bhi hai ya poochti hain aap ko kuch bataati bhi hain ya sirf poochti hain

SA: nahi bataati bhi hain jaise abhi khaansi nazle ka chal raha hai corona mujhe abhi jaise samjh nahi aaya toh unse pooch sakti hun k bhae aap corona k hawale se pooch rahi hai ya us k elaawa woh mujhe bata deity hain

RA: aap joh poochti hain aap ko bata deity hain

SA: haan bata deity hain

RA: acha aap ko joh hamari health facility health worker hai us se jorne mein koi kirdaar ada kiya hai yeh CHW ne aap ko pehle se pata tha haan yeh hai center hai yeh joh worker aati hain woh bataati hain center k hawale se

SA: pehle se pata tha

RA: acha aap ko lagta hai k is kaam ko hum mazeed kaise behtar kar sakte hain

SA: mazeed aur acha ho raha hai mashaallah se

RA: kaise aur acha

SA: jaise yahan delivery case ka masla nahi hai

RA: haan

SA: delivery case horaha hai

RA: konsa wala

SA: delivery case

RA: delivery ka case

SA: delivery case hai abhi yahan nahi hai toh woh hojaaye

RA: delivery toh woh center pe hogi na thek hai jaise yeh ghar pe aati hai toh inke zariye koi maloomaat koi information de sakein ya koi cheez joh aap chahti hon k in k zariye hum kar lein kia kar sakte hain

SA: mein yeh chahti hun in ka kaam acha hojaaye

RA: kaise mazeed acha hojaaye kaise kia wohi toh poochna chah rahi hun mazeed kaise acha ho kia cheez aap ko lagta hai k kami hai thori si

SA: woh toh hai kami

RA: kia kami hai

SA: waise kami koi nahi hai mein bolti hun aura chi position pe jaayein is se ziada tarakee karein

RA: acha acha lekin yeh k aap k hawale se kia karein woh toh un ki tarakee hogayi

SA: jab in ki tarakee hogi toh hamare liye kuch kareinge na ab woh itni dur se hamare liye arahi hai na

RA: haan

SA: sahi hai na inki tarakee hogi toh hamare ko sahulat hogi

RA: kaise sahulat hogi in ki tarakee se

SA: hum ladison k paas jaayeinge

RA: hmm

SA: toh in ke aur kaam aayeinge joh hum nahi kar sakte toh un k liye hamare liye aur behtar hojaaye

RA: koi aisi maloomaat wagera ya koi aisi kisi bimaari k hawale se koi aap ka masla ho jaise shadi k baad hota hai hone k baad koi masaail hojaate hain sehat k hawale se aap ko lagta hai in k zariye hum is ki information aap tak pohcha sakein kia cheez aisi kis cheez k baare mein aap ko lagta hai k hamein maloomaat deini chaiye aur kuch

SA: maloomaat deine ajaati hain

RA: ajaati hain acha aur kuch kehna chaheingi kuch batana chaheingi k kis tarhan se hum CHW k kaam ko behtar karein

SA: nahi

RA: shukriya bohat bohat……….

IH-IDI-06 Recording: 22:08

SS: jee AA aap ki umar kitni hai aap ki umar

AA: umar ka toh pata nahi hai

SS: aap ki shadi ko kitna arsa hua hai

AA: meri shadi ko 4 saal hogaye

SS: 4 saal huye hain aur aap ki mahwaari kab aayi thi

AA: meri mahwaari

SS: hmm

AA: meri mahwaari aayi thi

SS: umar ka andaza laga rahe hain 20 25 hogi

AA: haan

SS: thek hai kitna parhi huyi hain aap

AA: kuch bhi nahi

SS: acha koi kaam karti ho ghar pe

AA: nahi

SS: koi nahi sirf ghar k kaam karti ho

AA: haan sirf ghar ka kaam

SS: achaa acha yeh mujhe bataayein k joh hamari CHWs aati hain woh kitni baqaaidgi k se aap k ghar aati hain

AA: woh aati hain hamare se poochti hain k tumhare ghar mein kon hamal se hai kon nahi hai toh jaise bataate hain hum hamal se hain ya koi nahi hai toh bas chali jaati hain phir ultrasound karwaane k liye jao toh 3 maheeney ka ultrasound hosakta hai wahan pe jao toh kehte hain k 3 maheeney se upar ka nahi hota hum log nahi kar sakte bas phir wapis kar leite hain file wagera woh kuch bhi nahi banaate abhi mera joh bacha paida horaha tha hamal hua hai file banaane k liye gaye who nahi banaayi thi

SS: hmm

AA: phir woh mera operation se bacha paida hua hai hamare 60 60 hazaar se upar lag gaye

SS: acha toh isi baare mein poochna chah rahi hun toh yeh ek maheeney baad aati hai aap k paas 2 maheeney baad kitne arsey baad aati hain

AA: ek maheeney baad aise ajaati hain

SS: ek maheeney baad aati hain aur aap logon ko yahan rehte huye kitna arsa hua hai

AA: hamein 8 saal hogaye hain

SS: 8 saal hogaye hain aur 8 saal matlab shadi ko kitna arsa hua hai

AA: 4 saal hogaye hain

SS: shadi ko 4 saal huye hain yeh aap ka pehla bacha hai

AA: haan

SS: acha toh yeh mujhe bataayein k joh yeh aati hain aap k ghar abhi sirf mein is pe kar rahi hun joh ghar pea ate hain aap se maloomaat leite hain toh kis kisam ki maloomaat leite hain sirf hamal k hawale se poochti hain

AA: haan hamal ka hi poochti hain

SS: acha aur kuch nahi poochti

AA: aur kuch bhi nahi poochti

SS: acha us mein kia poochti hain hamal se bhi

AA: yeh poochti hain k kitne maheeney hogaye kitne maheeney nahi huye agar file wagera banaani hain toh ajao jab jaate hain toh banaate nahi hain

SS: hmm acha aap ne kaha k banaate nahi hai

AA: haan

SS: thek hai yeh joh aap se poochti hain us k baare mein aap kaisa mehsoos karti hain aap ko lagta hai is se koi faida hai

AA: jab koi dawaai nahi deite filein nahi banaate hain toh faida kis cheez ka hai

SS: acha matlab k joh CHW aati hain kaam karti hain toh aap ko lagta hai koi faida nahi hai

AA: nahi hamein nahi lagta k koi faida hai us ka lekin ek bacha yahan paida hua tha meri bari bhaabi hai jab bhi aati hain us k baare mein hi poochti hain

SS: acha

AA: us bache ko 3 4 saal hogaye paida huye phir us k sar mein paani tha us ki file in logon ne banaayi thi bas jab bhi aate hain us k baare mein poochti hain bas

SS: aur kisi k baare mein nahi poochti

AA: kisi k baare mein nahi poochti

SS: aap ne unse poocha k aap baaki bachon k baare mein kyun nahi poochti

AA: nahi hum log nahi poochte

SS: kyun

AA:bas un ki marzi pata nahi kyun nahi poochte kia hai kia nahi hai jab jaate hain file hi nahi banaate toh phir shayad hamara naam nahi hoga is liye nahi poochte

SS: acha jab aap k paas aati hain toh aap k hawale se poochti hain aap se poochti hain k aap k ghar mein kitni shadi shuda aurtein hain aisa kuch poochti hain

AA: haan aisa poochte hain

SS: phir aap ne bataya aap bhi hain

AA: haan mein ne bataya mein shadi shuda hun yeh meri bhabi hai yeh bhi shadi shuda hai

SS: hmm

AA: ek bhabi joh bari hai jis ka naam a kar poochte hain woh gaaon mein rehti hai

SS: acha woh chali gayin hain yahan se

AA: haan woh chali gayi hain

SS: phir bhi us k baare mein poochti hain

AA: haan phir bhi us k baare mein poochti hain

SS: aap ko shikaayat hai is baat se

AA: haan toh jab bataate bhi hain yahan pe nahi hai hamesha k liye chali gayi phir baar baar a kar usi ka naam poochte hain

SS: hmm hmm

AA: toh us ka naam hai file mein hamara nahi hai

SS: aap ne poocha nahi us se k us ka naam hai hamara naam nahi

AA: nahi hum logon ne nahi poocha

SS: acha acha mujhe yeh bataayein k abhi akhari dafa yeh 4 maheeney ka bacha hai aap ka

AA: haan

SS: toh jab yeh hua toh is mein aap k akhari hamal mein koi CHW ne kirdaar ada kiya kuch aap ki madad ki is mein

AA: nahi kuch bhi nahi ki

SS: kuch bhi nahi ki

AA: nahi

SS: aap ne unhein bataya tha

AA: haan bataya tha gayi bhi thi un logon ne kuch bhi bas goliyaan di thi yeh khaati raho

SS: hmm

AA: ek baar ultrasound kiya hai toh bas kehne lage tumhari date upar hogayi hai ab ja kar apna delivery karwa dou

SS: acha toh aap kia deir se gayin thi center

AA: nahi mein shuro shuro mein 3 maheeney hamal ko huye thy mein gayi thi in logon ne ultrasound bhi nahi kiya file bhi nahi banaai kuch bhi nahi kiya aise bhaga diya phir 4 maheeney k baad phir se gayi phir se in logon ne bhaga diya

SS: hmm

AA: jab bhi jaate thy bhaga deite thy phir itna maheena shuro hua tabhi un logon ne ultrasound kiya kehte hain ek hafta tumhara date se upar hogaya hai ab tum jao apna delivery case karwa lou

SS: acha yeh mujhe bataayein k yeh joh unhon ne aap center pe gayi thi toh aap ko center ka kis ne bataya tha

AA: center ka kis ne bataya tha

SS: hmm matlab aap jab center pe gayin jab aap hamal se thi toh aap ko center ka kis ne bataya tha center pe ja k apna checkup kara lou

AA: woh toh hamein pata hai k wahan par checkup hota hai

SS: yeh CHW ne aap ko nahi bataya worker ne nahi bataya

AA: nahi

SS: acha toh yeh mujhe batao k phir wahan CHW aap ko center pe nahi le kar gayin

AA: nahi le kar gayi wahan par

SS: acha aap ne bataya aap hamal se hain

AA: haan yeh bataya

SS: is ne apne paas kia kiya likh liya

AA: haan yahan par likh kar gaye phir hum se nahi poocha tha aati thin AA bacha kab paida hua kab nahi hua bas yeh pooch kar chali jaati thin

SS: acha jab yeh chota bacha paida hua is ko a k kisi ne deakha

AA: nahi

SS: is ko bhi nahi deakha

AA: nahi

SS: koi nahi aaya is ka kad wagera karne k liye

AA: wazan karne k liye aaye thy bache ka

SS: acha kia kiya unhon ne

AA: wazan check kiya hai bas wazan check kar k phir chale gaye phir us k baad nahi aaye

SS: acha wazan wagera toh kar k gayi wazan kiya kad kiya

AA: haan

SS: acha aap ko aap k hawale se koi maloomaat di kabhi inhon ne

AA: nahi

SS: k yeh kha lou yeh kar lou pregnancy mein ya waise

AA: hamein kuch bhi nahi bataya in logon ne meri joh bari bhaabi thi na 3 4 saal se us ko bas bataya tha in logon ne

SS: aap logon ko kuch nahi bataya

AA: hum logon ko kuch bhi nahi bataya yeh hamal se huyi is k do bache hain is ka bhi in logon ne kuch nahi kiya na poocha tha na kuch mein hamal se huyi hun mera bhi kuch nahi kiya in logon ne yahan par yeh dusri taraf rehti hai mein aur woh sath mein gaye thy hum dono ko hi in logon ne nikaal diya tha k jao yahan par na ultrasound hota hai na kuch hota hai yahan par toh aag lag gayi thi machineyein jal gayin thi

SS: yeh kitni poorani baat hai

AA: abhi aap khud andaza laga lou mera bacha 4 maheeney ka hai

SS: toh yeh is se pehle pichle saal ki baat hai

AA: haan

SS: toh jab aap ultrasound karne k liye gayin toh ultrasound nahi kiya unhon ne

AA: haan nahi kiya phir 9 maheeney k baad gayi phir ultrasound kiya

SS: tab toh kar diya na jab 9 maheeney k baad gaye

AA: haan

SS: hosakta hai wahan waqai machine kharab ho

AA: pata nahi yeh pata nahi

SS: aap ko yeh bataya nahi unhon ne

AA: nahi

SS: acha ab agar aap ko center pe jaane ka boleinge toh aap jaayeingi

AA: abhi kis liye jaayeinge

SS: nahi waise agar aap hamal se ho jaate ho ya kuch

AA: jab center mein kuch faida hi nahi hai toh kis liye jaaye insaan khud ki taange bhi thaka k jao yahan se paidal chal k jaate hain wahan par jaayein koi kaam hi nahi hota phir wapis aise ajaate hain

SS: acha toh aap ko CHW se shikaayat hai koi

AA: bas yehi toh shikaayat hai ab yeh log kuch file wagera banaate toh shayad hamare itne paise nahi lagte na Jinnah mein le kar gaye unhon ne bhi raat k time nikaal diya tha nahi liya

SS: hmm hmm

AA: phir dusri hispatal mein le kar gaye wahan pe un logon ne hum se 60 hazaar liya hai

SS: hmmm

AA: sirf operation karne ka dawaaiyaan bhi hamari khud ki thi

SS: toh aap ko yeh lagta hai k agar yeh aap ko barwaqt bata deite toh aap ka elaj kahin kyun k yahan par aap ko pata hai delivery ki toh facility nahi hai chota hispatal hai na yahan nahi hosakti lekin agar barwaqt kisi aur hispatal mein

AA: le kar toh jaate hain na

SS: hmm

AA: yeh in logon ne likha nahi hai

SS: toh aap k paise ziada kharch hote hain

AA: haan

SS: acha aap mujhe batao k hum is ko kaise acha karein kaise aap ki narazgi ko dur karein kia karna chaiye

AA: hum toh yehi chahte hain k joh bhi jis ko bhi hum center mein jaayein naam likhein parwarish dein us ki

SS: hmm

AA: abhi har koi in k center jaise nahi hota har kisi k paise bas chalein jaayein

SS: hmm hmm

AA: ab yeh toh hamein pata hai na hum logon ne kaise jama kiye hain

SS: sahi hai aur kia aap ko aisa lagta hai k CHW joh hai woh aap ko center pe koi kirdaar ada karti ho kuch karti hai joh worker aati hain is k zariye bhi madad kar sakte hain aap ki is k zariye koi madad nahi kar sakte acha is k zariye koi maloomaat aap tak pohchaayein koi maloomaat aap ko aise ho k joh aap k liye asaani ho ya koi faida pohchaaye pohcha sakte hain

AA: yeh nahi hai

SS: inhon ne kabhi maloomaat nahi di aap leina chahti ho k yeh aap ko kuch bataayein aap ko is se faida hojaaye

AA: doctoron ka toh kaam hi yehi hota hai agar mareez ko kuch bata dein toh mareez aage se kuch na kuch kar leita hai pehel se agar nahi bataayeinge toh yeh kaam hoga joh mere sath hua tha

SS: hmm hmm hmm toh wohi toh poochna chah rahi hun k kia bataayein aisa yeh ek toh masla aap ne mujhe bata diya k bhae aap ki service aap ko un logon ne nahi di aap k paise ziada lag gaye ab mujhe samjh mein agai ab mein aap se pooch rahi hun koi maloomaat agar woh aap ko dein koi hamal k hawale se koi bache k hawale se toh faida hoga

AA: haan toh faida hojaayega na

SS: kia maloomaat dein

AA: yeh maloomaat dein k wahan par jaise hum log jaate hain checkup karwane k liye ultrasound karte hain kuch karte hain wahan par sab ko nazar aata hai kaise delivery hogi kaise nahi hogi

SS: hmm hmm

AA: yeh toh nazar ajaata hai yeh log bata sakte hain na agar likh nahi sakte toh bata sakte hain kyun k aage se insaan kuch banaane k liye tayar hojaaye

SS: hmm toh aap yeh chahti hain k agar koi masla ho toh joh health worker hai woh aap ko pehle se bata dein

AA: haan

SS: jab aap k ghar arahi hoti hain us time pe bata dein acha aur is mein aap ko facilities center mein le k jaane mein koi kirdaar ada karti hain ya nahi

AA: yeh bata toh rahi hun hum log khud hi jaate hain

SS: khud hi jaate hain yeh sab kuch khud karte hain aur aap ko bolti bhi nahi k center pe aao elaaj karao

AA: nahi kuch bhi nahi kiya in logon ne

SS: acha toh aap chahti hain is tarhan se bhi aap ko woh karein wahan le kar jaayein ya wahan pe jab aap jaayein wahan par maujood hon aisa chahte hain aap

AA: hum toh sab kuch chahte hain

SS: toh phir bataayein na kia chahti hain acha yeh batao is k kaam ko aur kaise behtar bana sakte hain k aap khush hojaayein health worker ko kis tarhan se aap k liye faida hojaaye

AA: mein bata toh rahi hun k har kisi ko pehle se yeh log bata dein ache se elaaj karein sab kuch karein k aage un ko pareshani na hon

SS: acha aur koi aur cheez is k elawa k bhae pehle se unhein bata diya jaaye un ko koi pareshani na hon aur koi cheez k wahan jaayein toh aap ki koi madad hojaaye center tak le k jaane mein koi madad hojaaye aisa kuch

AA: abhi toh aap ko khud ko pata hoga k pait wali aurat kaise chalti hai

SS: hmm

AA: jab sawari nahi hai kuch bhi nahi hai toh hamein chal kar jaana parta hai who toh tension nahi hai chalo chal kar bhi ja sakte hain us mein hamara faida shayad hojaayega wahan pe chal kar phir jaane se hamara faida nahi hota toh wahan par jaane ka faida koi faida nahi hai na

SS: hmm thek hai chalein shukriya bohat bohat aap ka……..

IH-IDI-03 Recording: 18:21

RA: acha mera naam RA hai mein aga khan university ki taraf se aayi hun thek hai mujhe yeh bataayeingi k aap ki umar kitni hai

AA: umar toh mujhe yaad nahi hai

RA: phir bhi thori andaazan kitni hogi

AA: 35

RA: acha thek hai shadi ko kitna arsa hua hai

AA: 19 saal

RA: 19 saal hogaye thek hai aap ka pehla bacha kab hua tha shadi k kitne arse baad hua tha

AA: ek saal baad

RA: aur aap ko shadi se pehle mahwaari kab tak aayi thi

AA: shadi k baad aayi thi

RA: shadi k baad aayi thi

AA: haan

RA: thek hai sahi hai acha mujhe bataayein aap ne taleem haasil ki huyi hai

AA: nahi

RA: nahi haasil ki huyi acha aur waise koi kaam karti hain ghar k

AA: ghar pe toh kaam karti hun saara

RA: acha ghar pe kaam karti hain is k elawa koi kaam

AA: nahi

RA: ghar se beith k koi kaam karti ho

AA: bas yeh ghar ka kaam hai

RA: hmm

AA: khana pakana bartan kaprey jhaaro paucha sab kar leiti hun

RA: hmm hmm

AA: aur dusra kaam nahi karti

RA: kitne bache hain aap k

AA: 6

RA: 6 bache hain aur sab se chota bacha kitni

AA: yeh hai 7 saal ka

RA: 7 saal ka hai acha aur yahan rehte huye kitna arsa hogaya

AA: shadi kar k aayi hun unhein 19 saal huye hai na

RA: toh yahin pe hi reh rahe hain

AA: haan

RA: thek hai acha mein na aap se thore se joh hamari CHW aati hain health workers aati hai na un k hawale se sawal karongi who kitne arsey mein aap k ghar aati hain

AA: kon who larkiyan

RA: hmm hmm

AA: who aati hain do maheena maheeney k baad aati hain

RA: maheeney do maheeney k baad aati hain

AA: haan

RA: acha thek hai aur who jab aati hain toh who kis kisam ka kaam kar rahi hoti hain kia poochti hain aap se

AA: yeh ghar ka hi poochte hain

RA: hmm kia poochti hain

AA: yeh khaane wager aka bachon ka

RA: hmm hmm

AA: sab pooch k chali jaati hain

RA: bachon ka kia poochti hain

AA: bachon ka yeh poochti hain kitne umar hai kitni nahi hai

RA: hmm hmm

AA: koi kaam pe jaata hai koi nahi jaata hai

RA: hmm hmm

AA: joh is ka abbu hai ek hi who kaam karta hai

RA: thek hai

AA: aur sab chote hain

RA: thek hai thek hai aur aap k hawale se kuch sawal karti hain kia poochti hain

AA: yehi poochti hain ghar ka khaane ka kia banaatey ho kuch kia nahi banaatey kia khaate ho kia bachaate ho

RA: hmm

AA: bahar se kitna aata hai kitna nahi aata yeh sab pooch k chali jaati hain

RA: acha aap ki sehat k hawale se aap se koi sawal karti hain

AA: nahi nahi aisi baat nahi

RA: aisa kuch nahi poochti

AA: nahi

RA: aa aap k bachon k hawale se k aap k kitne bache hain hamal se hain ya nahi is tarhan kuch poochti hain

AA: haan poochti hain k kitni umar hai kitni nahi hai

RA: haan

AA: chota bacha 5 saal se upar hai chota hai ya nahi hai

RA: hmm

AA: mein ne us ko bola nahi hai

RA: hmm

AA: 5 saal se upar hai chota nahi hai

RA: hamal se related kuch sawal karti hain aap se

AA: aisi toh baat nahi ki

RA: aisa nahi hai acha

AA: 2 saal hogaye hain abhi ziada mere paas nahi aati kyun k mere paas chota bacha nahi hai 5 saal se kam bacha nahi hai toh ziada nahi aati

RA: hmm

AA: bas pooch k chali jaati hain katra wagera pilaana hota hai toh pilaati hain aati thi abhi toh yeh bara hogaya hai nahi aati hain

RA: hmm hmm acha toh jab yeh aap se is tarhan k sawal poochti hain aur yeh joh apna kaam karti hain aap kaisa mehsoos karti hain is hawale se

AA: sahi hai bilkul

RA: sahi hai

AA: haan poochti hain is mein aisa masla nahi hai

RA: hmm aap ne bata diya

AA: haan

RA: araam se kia wajha hai joh un ko araam se bata deity hain

AA: haan bata deity hain

RA: kyun bata deite hain

AA: aati hain darwaaze pe aati hain hum log izzat karte hain is ko bithaatey hain kuch poochti hain hum log jawab bhi de deite hain

RA: acha

AA: aisa masla nahi hai kuch

RA: lekin yeh hai k koi bhi aayega aap se poochega aap bata dougi

AA: haan

RA: acha

AA: bata dungi

RA: thek hai lekin is mein yeh hota hai k yeh aap un ko jaante ho is baat se warq parta hai is liye aap unhein bata deite ho

AA: nahi nahi koi bhi aata hai toh hum log un ko bata deite hain

RA: aap bata deite ho

AA: haan

RA: thek hai

AA: bol deite hain bithatey hain un ki izzat karte hain bata deite hain us mein kia fark hai

RA: acha in k kaam se aap ko lagta hai k aap ka taluk hai ya aap k liye kuch karein kuch aisa mehsoos hota hai

AA: hum log bolte hain kuch bataayein toh us mein kuch faida hum logon ko bhi hai na isi liye hum log un ko bata deite hain

RA: kia faida hai aap ko kia faida hai lagta hai is se

AA: bola yeh k bachon k liye kuch na kuch magar kamzori mehsoos hoti hai bachon ko toh koi dawaai taakat ki deity hain isi liye hum bata deite hain

RA: acha

AA: elaaj hota hai bachon ka hum log gareeb hain pohanch hamari nahi hai kabhi samandar ka banda hai kabhi nahi hai toh free mein elaaj hojaayega bachon ko koi dawaai wagera takat ki mil jaayegi toh acha hai na isi liye hum log bata deite hain sab [IH- MWRA]

RA: thek hai toh yeh aap ko lagta hai k joh aurtein hain joh aap log hain yeh health workers aati hain

AA: haan

RA: toh bachon k liye koi na koi faida hojaata hai

AA: haan

RA: is wajha se aap un ko information de deity hain

AA: de deite hain

RA: thek hai aur apne liye kuch sochti hain aap k liye koi faida hai

AA: nahi nahi

RA: aap nahi sochti

AA: nahi

RA: apni sehat k hawale se nahi sochti

AA: nahi

RA: kyun

AA: hum log bole bachon ko faide huye toh acha hai na

RA: thek hai aap ko apni zaat se nahi lagta k koi faida hai

AA: haan

RA: acha lekin aap ki umar toh itni ziada nahi hai aap ko kyun aisa lagta hai k aap k liye koi faida nahi hai

AA: (laugh) bas aisa hi sochte hain

RA: kia wajha hai is k peeche

AA: kuch bhi nahi hai

RA: kuch bhi nahi hai acha who kabhi a k aap k baare mein poochti hain aap k hawale se sawal karti hon bataati hon k bhae aa aap ki yeh umar hai kitne bache hain us k hawale se aur bache karne hain ya nahi karne kuch is tarhan

AA: aisa nahi poochti

RA: aisa kuch nahi kehti

AA: nahi

RA: acha acha aap akhari dafa jab hamal se huyin tab CHW ne kirdaar ada kia tha us mein

AA: nahi nahi

RA: kuch nahi aap ne unhein bataya tha

AA: nahi

RA: kyun nahi bataya aap ne unhein

AA: kis ko

RA: joh worker aayi us ko bataya tha

AA: nahi nahi pehli martaba aayi thi na

RA: hmm

AA: yeh joh tumhare pehle se aayi thi poochne k liye wohi aayi thi mere paas

RA: jab aap hamal se thin

AA: haan who aayi thin mere paas poochne k liye k koi pregnant hai mein ne kaha haan mein hun na

RA: hmm

AA: kitna maheena hai mein ne kaha 6 maheeney

RA: hmm

AA: toh bole aayegi ladies tere paas poochne k liye bhale a jaaye

RA: hmmmm abhi hain ya pehle thin

AA: kon

RA: aap hamal se

AA: abhi hun

RA: abhi aap hamal se hain

AA: 15 20 din huye larkiyan aayin thi bata k chali gayin

RA: acha abhi toh hain lekin jab akhari dafa yeh sab se chota bacha hai na

AA: haan

RA: yeh is se jab hamal se thin tab CHW ne koi kirdaar ada kiya tha us waqt

AA: aayin thin haan us ka elaaj yahan par hua tha na 5 saal tak

RA: hmm

AA: thora kuch hota tha mein yahan pe jaati thi dawaai wagera leine k liye

RA: hmm

AA: haan phir 5 saal 6 saal hogaye phir hum logon ko bola k dawaai nahi milegi phir mein ne kaha sahi hai

RA: hmm

AA: yeh bhi meharbaani hai aap ki

RA: aur doraane hamal kuch aap k liye kuch hua tha

AA: nahi nahi

RA: kuch bhi nahi kiya

AA: nahi

RA: acha aap ne worker ko bataya tha us time pe aap hamal se hain

AA: nahi

RA: us waqt nahi bataya us waqt aati thin worker aap k paas

AA: nahi aati thin

RA: us waqt nahi aati thin thek hai aur phir us mein aap ne joh hamal k doraan rahin toh kahan dikhaaya kia kiya us mein

AA: mujhe shak hua na

RA: hmm

AA: shak hua bola k mein ne bola abhi nahi bacha ab nahi hoga 6 huye hai na jaldi jaldi hone wala tha abhi ek saal do saal k baad mujhe hota tha mein ne bola abhi yeh 7 saal ka hogaya hai abhi mujhe nahi hoga mahwaari ko ek maheena do maheena teen maheeney k baad aati thi

RA: hmm

AA: mein ne bola abhi mahwaari khatam hone wala hai tabhi nahi aati hai na toh phir aisa 5 maheena hua toh phir hilne laga

RA: hmm

AA: pait k andar mein ne bola abhi bacha mujhe mehsoos horaha hai na hai na mein gayi wahan par doctor k paas uktrasound k liye

RA: hmm

AA: toh us ne bola bola abhi 5 maheeney khatam hone wale hain 6 maheena shuru hogaya

RA: acha aise aap ko pata chala

AA: aise hogaya

RA: thek hai acha aur jab yeh toh aap khud se chali gayin worker ko aap ne nahi bataya

AA: haan

RA: us mein who aap ko nahi pata chal saka

AA: jee

RA: hum yeh janna chah rahe hain k worker ko aap agar bataaye toh us mein kia kirdaar ada kiya hua aap k hamal k doraan kis tarhan se aap ki madad ki ho center tak le k gaye hon aap ko kuch bataya ho aap k hawale se kabhi aisa kuch hua

AA: nahi nahi

RA: aisa kuch nahi hua aura a bachon k hawale se chote bachon k hawale se aap ko kuch bataya ho jab yeh bacha nauzaida tha ek maheeney ka tha bilkul paida hua huawa tha is k hawale se kabhi worker ne a k kuch kiya

AA: *Take pregnant women to HF* haan pehle aati thin bacha paida hota tha toh phir mujhe le k chalti thi

RA: acha

AA: hospatal bhi yeh sab hota tha check wagera naak kaan aankh wazan bukhaar sab check karte thy

RA: aa ko hispatal le k ja k

AA: haan le k jaati thin gaari mein bitha k

RA: acha

AA: phir a k gaari mein chor k chali jaati thin

RA: hmm acha mujhe yeh bataayein k joh abhi worker aati hain kaam karti hai aap se poochti hai bache se poochti hain

AA: haan

RA: thek hai us k elawa aap ko lagta hai un ko kuch aur karne ki zaroorat hai

AA: nahi nahi

RA: koi maloomaat aap ko deine ki zaroorat hai aap k hawale se

AA: nahi aisa kuch bhi nahi hai

RA: aisa aap nahi mehsoos karti

AA: 2 saa hogaye hain ziada nahi aati mere paas kyun k mere paas 5 saal se kam umar ka bacha nahi tha na aati thin poochti thin k bacha kitne saal ka hai mein ne bola 7 saal ka hai toh bola sahi hai

RA: thek hai toh koi aur baat aap ko nahi lagta hai bas a k mere paas chota bacha nahi hai toh thek hai agar nahi pooch rahi

AA: bas chali jaati thin

RA: toh aap ko lagta hai us ko aap k hawale se kuch bataane ki zaroorat hai ya chalo 5 saal se bare hogaye bache toh hai na us k hawale se koi

AA: nahi nahi kuch bhi nahi poochti thin

RA: who nahi poochti lekin aap ko aisa lagta hai us ko mujhe bhi batana chaiye

AA: nahi nahi yeh nahi socti

RA: nahi sochti kyun kia wajha hai

AA: mein ne bola abhi bacha bara hogaya hai na

RA: hmm

AA: toh phir mein us ko bol deity thi mere paas chota bacha 5 saal se kam umar ka nahi hai

RA: hmm

AA: toh phir chali jaati thin

RA: hmm acha aap ko waise aa elaaj karaane mein ya bache ki sehat k hawale se koi pareshani hoti hai

AA: nahi

RA: nahi hoti aap araam se kar leite ho

AA: haan

RA: ikhraajaat araam se utha leite ho

AA: haan

RA: dawaai le leite ho koi masla nahi hota

AA: haan

RA: us mein matlab bache ziada hain aap nahi sochte k ziada hai toh hamara free horaha hai center le jaayein ya kuch hamari madad hojaaye aisa socti hain

AA: aisa toh sochte hain lekin kia karon akele hun aur baaju mein koi bhi nahi hai choti bari joh khayal kare bachon ka

RA: hmm

AA: aisa toh mehsoos hota hai k bari beiti hai us ki shadi mein ne kar di yeh joh beti aayi thi chote ko le k who us ka beta hai meri beti ka

RA: yeh beti thi

AA: haan yeh beti thi bari beti hai

RA: hmm hmm

AA: 17 saal ki us ka yeh chota bacha tha

RA: hmm hmm hmm toh kia kia aap ko lagta hai k yeh is tarhan k masle aap logon k kuch hal hote hain k ikhraajaat ka masla hogaya ya kuch is tarhan ki cheez sehat k hawale se worker aati hai toh chalo center le jaati hai hamare liye yeh ek faidemand cheez hai ya nahi hain

AA: mein aisa samjhti hun bachon ka kuch ho kuch karne wala mein chali jaon

RA: hmm

AA: koi karne wala nahi hai phir nahi jaati

RA: hmm hmm

AA: haan ghar mein beithi hoti hun

RA: hmm hmm

AA: kahin bhi nahi jaati

RA: acha aap yeh lagta hai k aa kabhi aap ko aap worker ko kuch batana chah rahi hon lekin kisi wajha se aap nahi bata paayi hun koi rukawat aap ko kabhi lagi koi aa kabhi aap ne mehsoos kiya k worker joh aa rahi hain hamare kisi kaam ki nahi hai

AA: *sometime women hide* haan aisa mehsoos karte hain par mein kaam mein lagi hoti hun

RA: hmm

AA: toh phir chup jaati hun (laugh)

RA: acha

AA: (laughing) haan aise bhi bola joh tum beithi ho

RA: haan

AA: sawal kar rahi ho mein ne bola aise aayegi sawal kar k phir chali jaayegi phir mera kaam nahi hoga (laughing)

RA: hmm hmm

AA: abhi deakho mein khaana paka rahi thi aadha chor k mein yahan pe beith gayi hun

RA: hmm hmm

AA: aise mehsoos hota hai kabhi kabhi

RA: k yeh aayeingi poocheingi aur chali jaayeingi

AA: chali jaayeingi kuch faida nahi hoga (laugh)

RA: sahi bilkul sahi

AA: hamara kaam bekaar hojaayega para hai abhi

RA: hmm hmm

AA: aisa sochte hain

RA: toh abhi samjh lo mein pait se hun toh tum log aaye toh mein ne bola sahi hai yeh elaaj karwana hai na

RA: hmm hmm

AA: tabhi mein tere paas bheij gayi hun

RA: acha toh agar aap hamal se nahi hoti toh hamein bhi jawab nahi deity

AA: toh phir (laughing)

RA: toh phir aap chup jaati na worker k sath bhi aisi karti hain aap toh matlab k aap k chote bache nahi hote toh khud hi bhai se bol deity hain k mera chota bacha nahi hai

AA: haan (laugh)

RA: lekin aap yeh nahi sochti k chalo 5 saal se toh chota nahi hai

AA: haan

RA: bare hain kabhi aap ne yeh nahi socha bache toh who bhi hain

AA: jab mein farg hoti hun na

RA: hmm

AA: toh phir mein interview de deity hun

RA: acha

AA: jab farig nahi hote phir mujhe yeh kaam karna hai toh phir mein bolti hun (laughing)

RA: haan yeh rukawat aap k time masroofiyat hai joh

AA: haan

RA: aap ko lagta hai k yeh aise hi a rahe hain

AA: aise hi arahi hain

RA: acha sahi aur is k elawa aap kuch hamein batana chaheingi k koi cheez joh hum behtar kar sakein is k hawale se aap ko lagta hai k jaise center hai ek hamara na

AA: jee

RA: ya sehat k joh aa joh jagaahein hoti hain elaaj hota hai jarahe hote hain thek hai toh yeh health worker ka k koi kirdar hai is mein aap ko bataya ho k yeh center hain wahan jao is mein aap ko lagta hai k center se jorne mein in ka koi role hai in ki wajha se aap ko pata chala center ka

AA: aisa toh mein ne kabhi nahi suna

RA: aap ko pata tha pehle se center ka

AA: haan

RA: pata tha pehle se center ka

AA: haan

RA: acha aur matlab phir wahan kaise pata tha aap ko center ka kahin se suna tha aap ne

AA: yeh larkiyan aati thi na tabhi pata chala

RA: yeh joh worker aati hain

AA: haan

RA: thek hai inhon ne hi aap ko bataya center hai

AA: haan

RA: aur aur kia bataya center k baare mein idaare k baare mein kia bataya

AA: aisa bola k free mein elaaj hoga

RA: hmm

AA: bachon ka

RA: hmm hmm

AA: baron ka toh pehle nahi hota tha

RA: hmm

AA: maa ka toh nahi hota tha bacha paida hota tha us ka elaaj hota tha

RA: hmm

AA: toh phir hum log karwa leite thy

RA: hmm hmm

AA: aur kuch hota tha bas joh pait mein dard ya kaan mein dard joh thora kuch hota that oh hum log chale jaate thy wahan pe

RA: hmm hmm aur is k elawa aur koi cheez joh matlab idaare mein wahan gaye toh wahan kaisa laga udhar koi role hua k jaise aap hamal se ho toh us mein koi us waqt koi kirdaar ada kiya ho center k sath jorne mein

AA: nahi

RA: us waqt kuch nahi tha

AA: nahi nahi

RA: 7 saal pehle tha

AA: haan

RA: thek hai acha aur abhi abhi filhaal jab aapgayin center pe aap ko pata chala k yeh hai

AA: abhi toh nahi gayi hun na 2 saal hogaye hain ab nahi jaati

RA: toh abhi ultrasound joh aap ne kahin aur se karaya hai

AA: haan aziz doctor se

RA: acha toh yahan kyun nahi gayin

AA: yahan ka pata nahi tha na mujhe

RA: acha

AA: ultrasound karwa k aayi phir 5 6 din k baad phir pata chala k yahan pe ultrasound bhi hota hai

RA: ultrasound yahan par bhi hota hai

AA: haan

RA: yeh kis ne bataya aap ko ultrasound ka

AA: yeh joh worker aati hain na

RA: worker aati hain

AA: haan

RA: unhon ne bataya aap ko

AA: jee

RA: thek hai toh aap ko lagta hai k yeh joh worker hai yeh center se jorne mein yeh aap ki madad karti hain

AA: haan bohat karti hain

RA: kaise madad karti hain

AA: aati hain leine k liye wahan par bhi doctor k paas le jaati hain bachon ko le k jaati hain mujhe le k jaati hain

RA: hmm

AA: phir elaaj hojaata hai toh phir gaari mein a k chor jaati hai darwaaze pe

RA: thek hai matlab is tarhan ki madad karti hain aap ko wahan le k jaati hain acha aur center mein ja k madad karti hain aap ki

AA: haan wahan pe bhi karti hain

RA: kia karti hain wahan par

AA: jaise doctor k paas le k jaati hain mujhe bachon k sath

RA: thek hai

AA: haan doctor se baat cheet karwaati hain yeh bolti hain k doctor se baat karein

RA: acha agar worker na ho wahan pe thek hai

AA: jee

RA: toh aap ko bas yahan se gaari le k jaaye center toh phir phir kia kareingi aap

AA: chali jaongi doctor k paas

RA: khud se chali jaogi

AA: haan

RA: kar logi sab kuch wahin

AA: haan

RA: lekin agar worker se aap ko madad mil jaati hai

AA: haan

RA: thek hai lekin aap akele bhi kar sakti hain sab kuch kar sakti hain

AA: haan

RA: thek hai acha aap ko aise lagta hai k yeh koi cheez hai joh hum aa aap batana chahti hain worker ko lekin aap ko nahi mehsoos hota k mujhe zaroorat hai is ko batane ki aisa lagta hai aap ko

AA: nahi nahi

RA: aise nahi feel karti aap acha thek hai yeh mujhe bataayein k hum kis tarhan se joh yeh kaam hum kar rahe hain thek hai workers aap k ghar bhi arahi hain aap kole kar bhi ja rahi hain thek hai

AA: haan

RA: aur hum is ko kaise aap k liye behtar bana sakte hain jis mein aap ko lage k aap ka faida ho aur aap chupe nahi (laugh)

AA: aisa toh mat sochein aap faida hai na

RA: kia kia kaise kai cheez aap ko lagi yeh mera faida hai

AA: mera elaaj hoga free mein

RA: hmm

AA: khoon wagera zaroorat paregi toh sab masla wahin hal hojaayega

RA: hmm hmm

AA: pareshani ziada nahi hogi isi liye bola elaaj karwa lo

RA: thek hai matlab aap ko laga k free mein joh elaaj hojaata hai

AA: haan

RA: agar who is tarhan ki services mile toh CHW ka kaam aur behtar hoga aur ghar kaise aati hain who aap k ghar aati hai center pe nahi hoti ghar bethe aap ko CHW arahi hai aap k paas us ko hum kaise behtar banaayein k aap k liye faida hojaaye kia karein hum aisa kia cheez aisi karein joh ghar pe CHW arahi hain us k zariye hum behtari la sakte hain kuch kar sakti hain aisa behtar

AA: hmm

RA: nahi matlab center pe elaj kaise kar sakte hain

AA: haan

RA: thek hai aur is k elawa koi koi bhi baat agar aap hamein batana chahein apne hawale se CHW k hawale se center k hawale se kisi khayal ka izhaar karna chahein toh bhi aap kar sakti hain aap bata sakti hain koi aisi cheez hai joh aap hamein batana chahti hain

AA: nahi nahi

RA: koi cheez nahi hai aisi koi behtar banaaney k liye k haan is kaam ko is tarhan se karein toh acha hoga kuch is tarhan kar sakte hain kuch batana chaheingi

AA: nahi

RA: chalein thek hai thank you so much AA….

IH-IDI-05 Recording: 22:08

RA: acha aa FA mujhe aap yeh bataaiye k aap ki umar kitni hai

FA: meri ab hojaayegi 38 saal hojaayegi

RA: 38 saal hojaayegi thek hai aur aap yahan pe kitne arse se reh rahi hain

FA: mein yeh meri ammi ka ghar hai toh mein yahan pe takeeban 8 saal se reh rahi hun shadi hochuki hai meri

RA: hmm

FA: lekin mein apni ammi k sath hi reh rahi thi toh aami abbu ka intekaal hogaya sirf abhi bhai yahin pe hi hain

RA: thek hai

FA: jee

RA: thek hai kitne arsey se reh rahe ho 8 saal se reh rahe ho

FA: jee

RA: acha aur aap ki taleem hai kuch

FA: taleem bohat thori si hai yeh hai k tution wagera parhti thi quran shareef parha tha na toh bas urdu wagera likh leiti hun parh leiti hun bas itni hi hai

RA: aa school nahi gayin aap

FA: nahi

RA: acha kabhi school nahi gayin tghek hai acha is k elawa aap koi kaam karti hain

FA: nahi ghar mein bas ghar k kaam kaaj aur kuch nahi

RA: aur kuch nahi karti

FA: jee

RA: aur aap k husband kia karte hain

FA: mere shohar joh hain woh mazdoori ka kaam karte hain

RA: thek hai mujhe hai na ab aap se thora sa joh hamari workers hain un k hawale se baat karongi

FA: jee

RA: aap mujhe bataayeingi k kitne arsay mein aap k ghar pe aati hain

FA: kaafi arsay se arahi hain takreeban koi meri beti joh hai mashaallah se 6 saal ki hochuki hai us se pehle se aana jana hai

RA: acha aur kitne maheeno k baad aati hain

FA: bas jaise jaise unka visit ka hota hai pehle toh jaldi jaldi aati thin jab se corona virus ka chakkar hua na toh un ka visit kam hogaya warna toh aati rehti thin aur jab mein pregnant thi jab toh woh aati rehti thi maloomaat karne k liye na k bhae aap ki tabiat kaisi hai kaisi nahi hai

RA: hmm

FA: aur ab yeh corona ki wajha se kam arahe hain

RA: thek hai acha woh kis tarhan ki maloomaat leiti hain aap se

FA: bas who kabhi sehat k mutalliq ya phir aur hai na kuch din pehle yeh corona virus k chakkar mein koi naya form tha fill karna tha us k liye aayin thi bas in cheezon k liye aati rehti hain

RA: acha aur sehat k hawale se poochti hain kia kia poochti hain aap se

FA: bhae aap ki tabiat thek hai ghar mein bache thek hain kisi ko kuch nazla zukaam ya bukhaar wagera is tarhan k abhi taqreeban koi 4 5 din pehle bhi raat k time call aayi thi

RA: hmm

FA: aga khan center se toh k aap log ki tabiat thek hai ghar walon ki sab ki mein ne bola jee Allah ka shukar sab thek thaak

RA: nahi joh health worker aati hain joh worker aati hain who kia poochti hain sehat k hawale se

FA: woh jaise aaj kal toh corona ki wajha se koi bukhaar toh nahi hua kisi ko khaansi toh nahi huyi zukaam toh nahi hai is tarhan ka ziadatar rehta hai ya jab kabhi woh sawalaat karti hain toh kis kaam ko karne mein aap ko thakan mehsoos hoti hai is tarhan k bhi kuch sawalaat hote hain

RA: hmm

FA: bas woh yehi

RA: aur aap k hawale se aap k bache k hawale se koi sawal karti hain

FA: haan jaise in k bhi tabiat ka poochti rehti hain

RA: hmm

FA: toh jab tabiat kharab hoti hai toh bata deite hain nahi hoti phir yehi

RA: yeh corona se hat k hai

FA: jee

RA: jab corona nahi tha tab kia tha sehat k hawale se sawal kiya karti thin ya abhi bhi sehat k hawale se

FA: ziadatar toh bas yehi hota tha woh form le k aati thi na

RA: hmm

FA: toh us mein se who sawalaat maloom woh parhti jaati thi aur poochti jaati thi aur poochti jaati thi

RA: un mein kia hote thy

FA: k jaise k bhae k aap ne kia khaya is haftey mein sabzi kitni baar li aalu kitni dafa liye aur gosht aap ne konsa liya aur kitni dafa liya is tarhan k sawalaat wagera woh poochti thi

RA: aur hamal wagera k hawale se poochti hain

FA: jee

RA: kia poochti hain

FA: jaise aksar a kar darwaaze se aap k ghar pe koi pregnant toh nahi hai

RA: hmm

FA: nameera jaise naam likhe huye hain bhabiyon k meri nahi mein ziadatar yeh hota hota hai joh nahi hai phir nahi hai

RA: thek hai

FA: bas yeh itna poochti hain aur phir chali jaati hain

RA: aur bachon k hawale se k yeh hifazati teeke ya is tarhan ki koi cheez poochti hain

FA: ab wohi na kaafi arsa hogaya na ab yaad bhi nahi hai kaafi baatein toh

RA: hmm

FA: han yeh hai k jab in k injection wagera lag rahe thy jab course nahi hua tha jaise maloomaat karti thin toh pooch liya bhae card dikha dein aap ne agar injection lag gaya kitne reh gaye kitne nahi ab toh yeh k ab jab hote thy mein bata diya karti thi

RA: hmm hmm

FA: ab toh in ka course poora hochuka hai

RA: hmm acha toh aap kaisa mehsoos karti hain in k kaam k hawale se joh worker ghar pe aati hain aap se poochti hain aap ko kaisa lagta hai

FA: sab sahi lagta hai kyun k is se kaafi zahir si baat hai maloomaat un ko bhi hoti hai aur jaise who pregnancy mein le k jaati thin ultrasound karwaana khoon test karwana unhon ne jitney bhi who joh marhalley samjh lein aate thy na hum ne un ka poora sath diya hai

RA: hmm

FA: mein ne bhi meri choti wali bhaabi is ne choti bhaabi ne bhi haan ek bhaabi ne nahi sath diya tha us din thora dar laga tha k jab yeh huye thy in ki aeiri ka khoon nikaal k test wagera karte hain

RA: hmm

FA: toh us ka bhi kia karte toh unhon ne bhi karwaaya tha mein ne bhi jitney bhi ab tak joh bhi kaam huye hain hum ne karwaaya bhae mujhe toh sahi lage

RA: hmm hmm

FA: kyun k who le k gayin jab yeh nahi huye thy toh

RA: hmm

FA: is ki bhi who dil ki dharkan check kari unhon ne mera bhi kaafi acha that oh sahi raha

RA: aap ko sahi kia cheez lagi kia cheez aap ko lagi k haan mujhe sahi lagta hai

FA: sab se ziada acha yeh lagta hai k jab wahan jao na bohat ache tareeke se baat karte hain

RA: hmm

FA: sab se bari joh doctor hain un k paas jao bohat ache tareeke se kyun k aam hospital mein jao na toh ache tareeke se lady doctor baat nahi karti joh mujhe sab se achi baat lagi na k wahan jaon na toh bohat ache tareeke se baat karte hain

RA: hmm aur worker kaise baat karti hain

FA: who bhi bohat ache tareeke se baat karti hain

RA: hmm

FA: yehi sab se achi baat hai

RA: thek hai is se aap kehti hain k hum yeh baat pooch rahi hain aur is k elawa kuch cheez hoti hai joh aap kehte hain k haan in k kaam se hum khush hain ya na khush hain dono cheezein

FA: nahi na khush hone wali toh aisi kabhi kisi ne baat hi nahi kari

RA: hmm

FA: baaki yeh hai k joh bhae who maloomaat karti hai k bhae haan ab bataayein kia cheez hai toh is se hota yeh hai k bhae is se hamara masla hoga maloomaat leiti hain toh hamein bataayeinge bhi

RA: hmm

FA: kyun k hamein toh is cheez ka nahi hai na k apna k kia karna hai kia nahi karna bas aisi zindagi guzar rahi hai guzaar lo

RA: hmmm

FA: bas is cheez ka toh mujhe toh sahi lagta hai

RA: acha toh kabhi unhon ne aap ko bataya apna koi masla bataya aur unhon ne aap ko bataya ho masla hal hua hai kabhi

FA: han bas jab mein in k time pe jab mein jaati thi mere bohat takleef jaise arahi thi toh mein ne doctor ko bataya tha

RA: hmm

FA: toh unhon ne medicine wagera likh kar di toh mein ne phir istemaal kari mujhe kaafi fark para tha

RA: hmm

FA: baaki us k elawa koi aisa masla nahi hai

RA: hmm acha aap jab hamal se thin aakhari dafa in se thin

FA: Jee Jee

RA: kitna time hua

FA: jee yeh 2 saal ke hogaye hain

RA: 2 saal k huye na

FA: jee

RA: toh us mein joh hai woh aap k matlab k joh worker thi us ne koi kirdaar ada kiya aap k hamal k doraan

FA: kis lehaaz se jaise

RA: jaise hamal mein

FA: hmm

RA: koi cheez joh aap ko faida pohchaai ho health worker ne us k kaam k hawale se aap ko koi faida hua ho

FA: nahi aisa toh kuch bhi nahi who aayin hum un k sath chale gaye bas hamara ultrasound wagera hogaya le gayi hamein bas yehi

RA: matlab yeh kirdaar ada kiya aap ko center tak le k chali gayin

FA: jee wahan le k gayin wahan andar mujhe le k jaana tha le ke gayin hamara karwaya kaam

RA: hmm matlab woh worker ne udhar center pe bhi ja k madad ki

FA: jee

RA: doctor tak le k chali gayin aap ko

FA: jee

RA: wahan ja kar aap ko samjhaaya

FA: haan bithaya kahin bulaayeinge aap ko toh phir aap ko andar jana hai

RA: hmm

FA: joh doctor bole aap ne karna hai mein ne bola thek hai bhae

RA: hmm

FA: toh phir hum unhi hisaab se chale gaye

RA: hmm

FA: jab wapsi ka aaya toh unhon ne phir unhon ne wapis bhi bhijwaya

RA: aur unhon ne wapis bhi bhijwaya

FA: jee

RA: yeh kirdaar unhon ne ada kiya

FA: jee

RA: aap ko yahan se a k le kar wahan tak le kar gayin

FA: jee

RA: acha yeh mujhe bataayein k agar worker nahi aaye

FA: jee

RA: aap hamal se hojaati hain dobara agar worker nahi aati hain

FA: hmm

RA: toh kia center tak jaayeinge aap

FA: haan agar hua toh mein zaroor chali jaongi kyun k joh aap ki gaari mein aana jaana hota hai na mere ko mere se nahi jaaya jaata bilkul bhi mera foran BP low hojaata hai chakka aane lagte hain

RA: hmm

FA: toh phir mein joh aakhari dafa test karwaate hain delivery k baad jab bulaate hain wahan aga khan hospital bhi le k jaate hain toh us k liye mein jab gayi thin a toh mein apne shohar k sath bike pe gayi thi

RA: hmm

FA: jab unhon ne mujhe bulaya tha na toh mein ne bola k mein wahan nahi ja sakti toh phir magar unhon ne ek dafa toh wahin kar liya tha phir ek dafa le k gaye thy toh meri bohat tabiat kharab huyi thi

RA: hmm

FA: toh mujhse bilkul safar nahi hota

RA: acha is gaari mein safar nahi kiya jaata

FA: jee

RA: aap kehti hain apni gaari mein chali jaon

FA: haan mein phir in k pappa k sath bike pe chali gayi thi

RA: acha

FA: acha jab bacha chota tha

FA: jee

RA: joh nauzaida chand din ka tab health worker ne in ki sehat k hawale se koi kaam kiya ho koi kirdaar da kiya ho jaise aap ko toh hamal k doraan le kar jaati rahin

FA: jee

RA: center pe jab yeh paida hogaye

FA: jee

RA: phir koi unhon ne kaam kiya ho madad ki ho kisi tarhan se

FA: bas ziada toh mujhe yaad nahi bhae yeh tha aate rehte thy in k baare mein maloomaat karti rehti thi kuch masla toh nahi hai jaise check up k liye teemein aati rahin weight wagera check kiya sab kuch bohat ache se check kar k gaye aur ab abhi bhi jab woh aate hain jab abhi bhi joh last mein aaye thy yeh sab cheezein check kar k gayin thin

RA: acha thek hai

FA: jee

RA: saari ceezein is ka wazan wager kar k gayin thi

FA: jee

RA: acha is se koi faida hota hai aap ko lagta hai k joh bache ko check karti hain aap ko hamal k doraan le kar jaati hain koi faida hai is cheez ka

FA: hamal k doraan who joh le k jaati thi toh yeh k woh ultrasound wagera joh bhi kuch wahan hota hai us se yeh pata rehta hai haan bhae koi masla toh nahi hai kyun k jaldi jaldi toh hum bhi nahi karwa sakte hain k ultrasound karwaate rahein pata chalta rahe

RA: hmm hmm hmm

FA: toh yeh hai k phir woh is se yeh faida hojaata hai hamare ko haan bhae pata chal jaata hai k agar weak mein koi masla hai ya waise bhi who kehte hain khuda na khwasta tabiat kharab ho toh aap ajaao

RA: hmm hmm

FA: toh aise mein yeh hai k kaafi help hojaati hai insaan chala jaata hai doctor deakh leiti hain

RA: hmm hmm

FA: aur ultrasound karna pare toh kar leiti hain kyn k meri beti inse joh bari hai toh meri jab horahi thi thori tabiat kharab na toh who unhon ne mera ultrasound kiya tha

RA: hmm

FA: toh yeh tha koi khuda na khuwasta masla nahi tha

RA: hmm hmm hmm aur bache k hawale se kia lagta hai aap ko kia lagta hai wazan karti hai sar naapti hai kia faida hai is se

FA: wazan se toh bas yeh pata chalta hai wazan karne se k haan bhae un se pooch leiti hun bhae sab kuch sahi hai

RA: hmm

FA: aap ne yeh sab kuch kiya hai toh who bata deity hain k haan mashaallah sab normal hai sab thek thaak hai

RA: hmm

FA: kyun k jab meri bari beti huyi thi toh us ka toh yeh sab nahi hua tha na

RA: hmm

FA: toh hamein toh nahi pata tha na k yeh sar naapne se yeh cheezein kitni honi chaiye joh bilkul normal ho joh who karti hain toh pata chal jata hai haan bhae hamara bacha sahi hai abhi

RA: hmm aap ko kabhi hichkichahat toh nahi hoti hai worker ko apni baat batane mein

FA: nahi koi aisi baat bhi nahi hai aur phir jab keh diya bhae k aap ki baat joh hai woh raaz mein hi raheigi toh bharosa toh phir karna parta hai na

RA: bharosa karne ki kia wajha hai kaise bharosa karti hain un pe

FA: bas who itne ache tareeke se jaise who baat vaat karti hain toh khud hi dil ko bharosa sa hojaata hai ya shayad bhae naik niyatein se karti hain toh Allah taala khud hi bharosa de deita hai dilon mein

RA: isliye bharosa karti hain

FA: jee

RA: waise yeh hota hai k who aap ki hum zaban hai ya aap k elaake ki hain is se bhi fark parta hai

FA: nahi nahi is se koi fark nahi parta ab deakhein aap baat kar rahi hain

RA: hmm

FA: pehli martaba mili hun na

RA: hmm

FA: leikin dil mein aisi koi baat nahi hai k aap ki taraf se koi buraai ho dil mein k bhae yeh toh pehli martaba aayi hai yeh hamare ghar mein agayin

RA: hmm

FA: bharosa hai baaki Allah ki zaat hai

RA: thek hai aap ko lagta hai k who itne ache tareeke se aap ki baat sunti hain

FA: jee

RA: toh aap un pe bharosa kar leite hain

FA: jee

RA: baat cheet kar leite hain lekin hum zaban hona ya hum elake ka hona koi is se koi fark nahi parta

FA: nahi

RA: thek hai acha yeh mujhe bataayein k aap ko lagta hai k jaise joh CHW health worker arahi hain aap k ghar par toh is k through hum aap ko koi maloomaat de sakte hain ya hamein deini chaiye joh who a k aap se pooch k jaati hain

FA: jee

RA: toh who koi aur maloomaat bhi dein toh koi faidemand hosakti hai

FA: haan joh hamare kaam ki baat hai woh hamein bataayein

RA: hmm

FA: toh aur agar hum us pea mal kar k faida haasil ho toh us mein koi buraai nahi hai

RA: toh kia cheez aap ko lagti hai k kaam ki hosakti hai

FA: kaam ki sehat k mutalliq ki honi chaiye k joh jaise bache hain un ki seena kharab hojaata hai in dino mein ziadatar ab yeh hai k bhae bahar se dawaaiyaan woh de de k powder wali syrup toh kyun k ziadatar joh gharon mein bare boorhein hote hain who kehte hain bhae yeh jitney

faidemand hote hain utne hi nuksaandey bhi hote hain toh is cheez ka yeh k kuch aisi ho dawaiyaan joh bachon ko dein toh asaani bhi ho hamare liye aur bachon k liye nuksaande bhi na hon

RA: CHW k through aap chahti hain k dawaaiyaan bhijwaayein

FA: hmm joh asaan ho joh sahi hon bilkul na bachon k liye hon

RA: acha aur maloomaat

FA: maloomaat yeh k sehat k mutaaliq k bhae aap ghar mein in k liye aisa kar lein toh is mein asaani hogi k kisi time mein dawaai na deini pare gharelun totkon se bache ko hum cover kar sakein kyun k zaroori toh nahi na itni si bhi cheenk ajaaye toh hum foran dawaaiyaan hi dein

RA: hmm hmm

FA: is cheez ki bhi maloomaat thori si honi chaiye joh k abhi hamein itni nahi hai

RA: hmm hmm hmm

FA: toh agar jaise pata chal sake kyun k wahan hum jab center pe jaate hain wahan joh doctor hoti hai who bohat saari dawaaiyaan nahi deite who ziadatar hota hai aap aise kar lein aise kar lein

RA: toh who kehti hain joh baat aap ko center mein bata rahi hain who ghar pe hi aap ko pata chal jaaye

FA: haan agar pata chal sake toh bohat achi baat hai

RA: thek hai acha aur aap k aap ki sehat k hawale se kia maloomaat dein

FA: hamari sehat k joh bataayeingi who toh takreeban pata hi hai k bhae achi khoraak lo insaan ko ziada tension free hona chaiye

RA: hmm hmm

FA: yeh cheezein in cheezon ka pata hai k mere liye kia karna chaiye

RA: hmm aur apne liye koi zaroorat nahi hai CHW ki

FA: nahi (laugh)

RA: aisi yeh kia baat huyi aisi kyun baat ki aap ne

FA: bas aise hi

RA: kyun aisa lagta hai aap ko

FA: is liye k ziadatar bachon k ziada masle masaail rehte hain

RA: hmm

FA: beemariyon ki kyun k bhae aise apne marzi se bhae insaan bachon ko har cheez nahi de sakta k joh khuda na khuwaasta bachon k liye nuksaande ho apna toh pata hai k khoon ki kami hai ya waise hamein kamzori hai toh hum aise acha khana khaayein aur khush rahein sab se bari baat zehan joh hai na kisi kisam ki pareshani who nahi hon na toh insaan sahi rehta hai

RA: aur CHW aap ki pareshani dur nahi kar sakti aap ko lagta hai

FA: nahi (laugh)

RA: kyun

FA: kuch pareshaniyaan aisi hain joh meri dur nahi kar sakte

RA: hmm thek hai lekin koi aisi cheez jaise agar who pareshaniyaan aap ki sehat ko mutaasir kar rahi hai toh zaahir si baat hai k us pareshaani ka hal hoga aur woh pata hogi

FA: k jaise meinzehni tor pe pareshan hoti hun na

RA: hmm

FA: toh mein yeh sochti hun k Allah mein kia karon mujhe koi aisi medicine mil jaaye khaon toh mera zehan joh hai na pursukoon hojaaye kyun k mein joh hun na choti choti baat pe bohat jaldi pareshan hojaati hun

RA: hmm

FA: toh us ki wajha se na mera demaag joh hai na phir bas pareshan hoti rehti hun k kia karon kia karon toh mein is mein kehti un k kyun k choti choti cheezein aisi hoti hai jin pe zaroori nahi k insaan hadh se ziada pareshan ho

RA: hmm

FA: aur mere mein bohat buri aadat hai mein bohat ziada pareshan apne aap ko pareshaniyon mein mubtala kar leiti hun aur nikaalna bhi chahon na toh nahi nikaal paati ab us ki wajha se na din ba din mein hai na kamzor hoti ja rahi hun

RA: hmm

FA: warna khaane peene ki toh koi aisa masla nahi hai Allah paak ka shukar hai sab kuch hai lekin bas sirf haan is cheez ka hai k koi aisi medicine wagera ho joh insan kyun k asal mein jab mein doctor k paas jaati hun na toh bhae woh kehte hain neend ki joh dawaai deite hain us se neend bohat aati hai

RA: hmm

FA: abhi toh bachon ka sath hai sau thori na sakti hun 24 ghante

RA: hmm

FA: bachon ko deakhna ghar ko deakhna

RA: hmm

FA: toh bas yeh cheez hai thori si baaki aur kuch nahi

RA: acha yeh aap ne bola k CHW ka joh kirdaar hai maloomaat k hawale se who yeh hai k bachon k hawale se koi maloomaat dein sehat k hawale se gharelu totka ya is tarhan ki cheez jis mein hamein dawaaiyon ka istemaal na karna pare ziada aur aap k is se aap ko pata hai k aap ko kia khana hai kia nahi khana

FA: jee

RA: joh pareshaniyan hain agar us k hawale se koi masla hal hosake aap k

FA: jee

RA: thek hai acha yeh mujhe bataayein k center se joh yeh sehat farhaam karne wale joh centers hote hain ya idaare hote hain inse jorne mein aap ko lagta hai CHWs joh health worker hai un ka koi kirdaar hai

FA: haan zaahir si baat hai kyun k woh hamein toh bhae pata bhi nahi tha woh aati thin hamein maloomaat deity thin hamein bataati thin toh hamein pata chala na is ka warna hamein toh nahi pata tha k kon kahan par hai center kahan pe nahi hai kyun k woh hi aati thi unhon ne hamein bataya woh hi le k gayin

RA: hmm

FA: aur unhon ne wahan par sab hamein bataya toh hamein pata chala hamein toh nahi pata tha

RA: hmm acha thek hai aur is k elawa koi aisi cheez hai joh rukaawat banti ho CHW ko batane mein apni koi cheez share karte ho jab woh aati hain aap se maloomaat leite waqt toh us waqt koi rukawat banti ho koi cheez

FA: nahi hamara toh aisa kuch bhi masla nahi hai jab bhi koi aata hai hum toh araam se baat kar leite hain

RA: jaise aap ne bhaabi ka bataya tha

FA: jee jee jee

RA: wajha bataai thi joh unhon ne nahi di

FA: bas woh jaise log baahir aap ko pata hai jab insaan baahir mard khaas tor pe nikalte hain toh koi kuch kehta hai koi kuch kehta hai jaise bhai ne baat kari hogi toh unhon ne yeh baat kahi k nahi aisa hojaata hai aisa hota hai nahi deina chaiye kuch un ko dara diya

RA: kia aisa waisa kia cheez

FA: k bhae koi beemari hojaayegi khoon nahi deina chaiye is tarhan jaise log kehte hain kyun k jis waqt meri beti huyi thi takreeban woh mashaallah 6 saal ki hogayi toh wahan se unhon ne jab

bola tha jab aap ki delivery hojaaye toh aap hamein pehle phone kijiyega magar mein ne dar k maare nahi kiya kyun k jaise meri joh maloomaat thi toh unhon ne bola nahi nahi who joh naaf hai na naaf ki jagha se khoon leite hain aisa karte hain waisa karte hain Allah mein itni khaufzada hogayi mein ne phir nahi phone kiya magar woh phir khud jab aaye unhon ne meri beti ka check up wagera kiya toh koi aisa masla nahi hua mein ne kaha yeh toh mein ne galti kari matlab mein toh bata deity ziada acha rehta na

RA: hmm

FA: lekin woh yeh tha k khud agai thin phir meri beti ka checkup kiya toh koi aisa masla nahi

RA: aap ko phone bhi aaya

FA: jee

RA: unhon ne kaha k delivery ho toh bata deina

FA: jee

RA: lekin aap ne dar se kyun k aap ne suna tha k woh khoon leite hain

FA: jee

RA: toh is wajha se aap ne un ko nahi bataya

FA: hmm ab wohi baat hai na mein meri likhi hoti toh mujhe samjh hoti is ki bhae is tarhan ka test agar karwana chahte toh kyun karwana chahte hain

RA: hmm

FA: ab unhon ne jab baad mein aate rahe jab yeh mera beta hua toh unhon ne is k aiery ka khoon liya mashaallah mera beta bilkul theek thaak hai Allah ka shukar hai

RA: hmm

FA: toh phir mein ne un ko karne diya mein ne kaha haan aap kar lein

RA: aap ne kabhi in se joh khoon le rahe thy toh aap ne unse poocha tha k khoon kyun le rahe hain

FA: haan mein ne is cheez ka nahi poocha k bhae aap kyun le rahe ho lekin us k baad jab meri beti huyi thi jab toh mein ne nahi bulaya tha us k baad unhon ne polio ki vaccine hoti hai katre hote hain unhon ne pehle un ka khoon liya phir unhon ne us k baad injection lagaya phir us k baad unhon ne bola ek maah k baad aap dobara aayeingi toh khoon test hoga toh us se phir pata chalega teston se k yeh injection joh laga tha lagne se pehle kia position thi jism mein or lagne k baad kia position hai

RA: yeh kis ko laga tha aap k beti ko

FA: jee haan

RA: acha aur kyun lagaya tha unhon ne injection

FA: polio ka

RA: polio ka injection lagaya tha

FA: jee

RA: us k baad unhon ne test bhi kiya tha

FA: jee do dafa test kiya tha ek pehle kiya tha phir injection lagaane k ek maah k baad bulaya that oh phir lagaya tha unhon ne

RA: acha thek aur toh is wajha se aap ko thora sa lagta hai joh yeh health worker aati hai woh khoon leite hain

FA: haan hum log toh karwa leite hain meri bhaabi hai na woh zara aisi hain thori si is maamle mein na k bhae log khoon nikaal leite hain itni mushkil se banta hai aisa hai waisa hai who thori si katraati hain

RA: toh joh khoon nikaalte hain toh woh khoon nikaalne se pehle aap logon ko bataatey hain yeh saari cheezein

FA: jee jee bilkul bataate hain abhi kuch arsay pehle ki baat hai mein gayi huyi thi toh kisi peeche team aayi thi meri bhaabi hai na in ka in k shohar ka meri Lahore se bhaanji aayi huyi thi woh thi un ka beta in logon se in ka khoon test kiya tha in logon ka toh in se in ko bata k toh phir inhon ne ijaazat di thi toh woh blood sample le gaye thy aur test hogaya tha

RA: or phir us k baad hamari worker aayi CHW aga khan se aayin araam se sab bataya aap ki bhaabi ne bataya

FA: jee unhon ne bataya hoga

RA: acha jee aur aap log araam se bata deite hain

FA: jee

RA: is k elawa koi cheez kahin se sun liya koi baat k bhae worker a rahi hain aisa hai waisa hai

FA: nahi nahi aisa toh aaj tak kabhi nahi suna hai kisi se k kisi ne kuch galat kuch bataya ho k bhae yeh aisi hain inhein nahi aane dou

RA: hmm

FA: aisa toh kuch bhi nahi hai

RA: acha ab aap mujhe yeh bata sakeingi yeh k hum is kaam ko aap k khayal se aur kaise behtar bana sakte hain yeh joh health worker ka kaam hai is ko mazeed aap hamein mashwara deina chaheingi hum is ko aur kaise behtar banaayein

FA: is cheez ki koi samjh nahi (laugh) k kia aap ko jawab dun

RA: nahi joh aap ko lagta ho joh aap mehsoos karti hon k haan yeh arahi hai toh jaise aap ne bohat achi baat bataai k bhae dawaaiyon k bajaaye gharelu totka gar hamein in k through pata chal jaaye toh hamein doctor k paas jaane ki zaoorat hi na pare

FA: jee bilkul sahi baat hai kyun k baaz time woh cheez toh bohat choti si hoti hai lekin us se faida hamara ziada hojaata hai

RA: hmm

FA: toh dawaaiyon se bhae bache bhi ghabraate hai na dawaaiyaan peete huye k bhae yeh kia rona shuro hojaate hain in cheezon ki wajha se

RA: hmm hmm hmm

FA: toh is wajha se

RA: thek hai

FA: baaki yeh hai k kaam toh bohat acha hai un ka bohat acha kar rahe hain

RA: hmm hmm aap log khush hain

FA: jee

RA: acha koi aisi cheez john a khushi wali ho who bhi bata dein ta k hum us ko behtar kar sakein

FA: (laugh) nahi mere nazdeek toh koi aisi baat nahi yeh koi aisi cheez nahi hai

RA: haan

FA: sab thek hai

RA: thek hai chalein thank you so much……

IH-IDI-05

FA: 34 years

2 bache 1 miscarriage, 4 months ka bacha hai ghar ka kaam karti hun

RA1. Kia household visit k doraan CHW bagaaidgi se ghar aati hain?

FA: Aati hain ek do maheenay mein bache k baare mein poochti hain teekon k baare mein poocti hain bas yeh sawal poochti hain

RA2 Aap is baare mein kaisa mehsoos karti hain?

FA: sahi hai bas kuch khaas nahi yeh aati hain sirf pooch k chali jaati hain hamein koi sahulat nahi mil rahi bas aati hain pooch k chali jaati hain yehi teeka wagera k hawale se maloomaat deity hain

RA: ap kay haway say ya hamal kay hawalay say?

FA koi maloomaat nahi deity hamal k hawale se or kuch bataya nahi.

RA acha ap kiu worker trust kiun karti

FA: workers pe trust hai aga khan ki taraf se aati hain who batati hain bachon k liye teekon k liye bas yeh sawal karti hain k hamal se hain koi zaaya toh nahi hua bas yehi poochti hain.

RA acha ap kay akhiri bachy ki paidaish main koi kirdar ada kiya worker nay?

FA: Is ki paidaaish k baad koi aaya hi nahi thaw azan check kiya phir us k baad nahi aaye shuro mein kiya tha yeh paida huyi hai do baar aaye mere paas.

RA: hmmm

FA: agar koi pregnant hai aurat pooch k chale jana thek nahi sirf pooch k na jaaye koi sahulat dein delivery k hawale se check up k hawale se bas pooch k chali jaati hain yeh nahi poochti k aap hamare saath chalein doctor ko dikhaayein kuch maloomaat wagera ho bas likh k chali jaati hain.

RA : aur jab ap hamal say thien tab?

FA: worker sirf do baar aaye hain bachon ko deakh k pooch k chali jaati hain hae maah aaye check kare par nahi aati hain bas poocha chali gayin.

RA acha jab who ati hain tou ap ka rawaya kaisa hota hai?

FA: who aati hain hum sahi se jawab deite hain bharosa bhi hai

RA acha ap ki sehat kay hawaly say pochti hain?

FA: aise toh kuch sawal nahi karti k aap beemaar hain BP hai bas pregnant ki hadh tak poochti hain likh k chali jaati hain kehti hain hamein aage jawab deina hota hai k hum har maheeney gaye thy ya nahi. Bas yeh poochti hain pregnant k hawale se sirf likhne ki hadh tak toh nahi hota na bas likhti hain chali jaati hain

RA acha aur kuch kehna chahiye gi?

FA nahi bas aur kuch nahi

IE- Date: 29102020

RA: acha name aap ki umar kitni hai

CHW: 30 plus

RA: aur aap ki taleem

CHW: BA

RA: kaam ka tajurba kitne saal ka

CHW: 2003 se le k abhi tak continue hun 15 16 saal se ziada hi hogaye hain

RA: 15 16 saal se ziada huye hai

CHW: jee ziada huye hain

RA: surveillance mein aap ko kitne saal huye

CHW: abhi toh surveillance mein hun matlab mein ne 3 saal HR mein kaam kiya tha us k baad se mein surveillance mein hi DSS ko deakh rahi thi joh ab VR mein change hogaya hai pehle yeh DSS k naam se jana jaata tha ab is ko VR kehte hain vital registry

RA: acha name mujhe bataaiyega surveillance k kaam k hawale se aap kaisa mehsoos karti hain

CHW: surveillance ka kaam bohat acha hai yeh hamara basic data is mein se milta hai aur isi basement se aage hamare projects aate hain matlab hum joh bhi data capture karte hain MWRA ko matlab joh married women ko capture karte hain newborn capture karte hain unhi ko le kar aage projects pe kaam hota hai yeh hamara baseline data hai joh hamara surveillance hai

RA: acha kaise capture karte hain data

CHW: hum door to door jaate hain interviews leite hain mothers se milte hain unse poora un k baare mein poochte hain k kitene married hain bache kitne hain bachon k hawale se poochte hain un k ghar k mahaul yeh sab cheezein deakhte hain

RA: acha is elawa aur kia cheezein deakhte hain

CHW: shadi shuda khawateen joh 13 se 49 saal k darmiyan hoti hain un ka hum poora data leite hain un ki pregnancy un ko kite bache huye abhi woh hamal se hain ya nahi hain 5 saal se chote bache kitne hain hum un se poochte hain

RA: toh aap surveillance mein rehte huye acha mehsoos kar rahi hain

CHW: surveillance mein rehte huye hum bohat acha mehsoos karti hun kyun k surveillance se hi hamara saara data tayar rehta hai hum door to door jaate hain information leite hain usi base pe hamare saare kaam aage chalte hain surveillance k bhi aur joh hamare naye projects aate hain woh isi base pe aate hain isi data ko le kar

RA: aur joh community mein joh aap ki routine hai aap ka kaam hai surveillance k elawa bhi toh us mein aap kaisa mehsoos karti thi

CHW: community mein bhi hamara acha role hai hamein acha response milta hai achi families bhi milti hain har tarhan k log hamein milte hain kuch log bohat ache bhi hote hain joh bohat izzat se baat karte hain toh kuch log hamein aise bhi milte hain joh bohat bure tareeke se bhi baat karte hain jaise k kuch toh aise bhi hote thy joh mau (face) pe yun darwaza de k maarte thy k jao hamein kuch nahi karwana phir bhi un se muskura ke un ko ache se guide karte thy k is mein aap logon ka faida hai hum logon ka faida nahi hai hum aap k faide k liye niklein hain gharon se aap k liye achi facilities le k niklein hain phir hum unhein guide karte thy har cheez k baare mein

RA: phir is se aap ko laga k woh aap ki baat maan rahe hain

CHW: jee kaafi log toh is cheez ko le k hamein appreciate bhi karte thy aap bohat acha kaam kar rahe hain yeh khidmat e khalq ka kaam hai toh bohat log toh appreciate bhi karte thy bohat acha kaam kar rahe ho is se hamari qaum ka bhala hota hai mulk mein achi facilities a rahi hain

RA: acha bataein mujhe aap apne household visit se hamal ya nauzaida bachon ki deakhbhaal mein kia kirdar mehsoos karti hain

CHW: hum unhein hamal k hawale se information deite hain k hamal k doraan aap logon ko kis tarhan se ehtiyaat karna chaiye kis tarhan se aap ko khayal rakhna chaiye unhein khaane peene ka khayal rakhna chaiye apne pregnancy mein aap ko kia kia khana chaiye kis cheezon se aap ko protein wagera aur aisi gizaayat milegi un cheezon k baare mein un ko counseling karte hain aur bachon k baare mein unhein batate hain k kia kia cheezein bachon k liye honi chaiye bachon ko kis tarhan se un ki deakhbhaal karni chaiye yeh cheezein batatey hain hum un ko

RA: aur kia kia batatey hain in k elawa

CHW: takreeban har lehaaz se unhein batatey hain agar k even kuch log aise bhi milte hain k personal issues bhi hum se discuss kar leite hain k matlab yeh hamein acha suggestion deity hain toh phir hum hal nahi kar paate toh atleast advise toh de sakte hain na un ko achi k aap yeh karo toh aap ka yeh problem hal hojaayega aise bohat se matlab hamare kaam ko le kar deakh kar bohat ziada attachment bhi feel karte hain

RA: aisi konsi problem joh aap se share karte hain hal bhi nikaal leite hain

CHW: matlab jaise hamare bachon k hote hain kaafi issues k hamare bache aise bigar rahe hain sunte nahi hain mothers ka yehi hota hai k hamare bache sunte nahi hain toh us ko batatey hain k un k sath kaise deal karo k bachon ka ek toh hum k bachon k zehan ko samjh jaayeinge na toh hum un k har tarhan se un k sath friendly hosakte hain kuch bache hote hain bohat jaldi samjh jaate hain kuch ko thora time lagta hai samjhne mein toh is tarhan se hum unhein guide karte hain k deakho bacha kis type ka hai kis tarhan ki baatein k who gor se sunta hai kin cheezon ko ziada who apne paas acha mehsoos karta hai us tarhan se unse baat karein

hamein phir unhein aise raaye deite hain toh unhein acha lagta hai un pe amal bhi karti hain aur us pe jab amal karti hain toh us ka nateeja jab hamein batati hain toh who khush bhi hoti hain k deakhein is baat se itna acha faida hua k aap ne hamein raaye di thi k bache is tarhan se deal karo is tarhan se guide karo toh hum ne who kiya hamein us mein acha bachon ka bhi acha response mila aur hum bhi relax hogaye bachon ki pareshaniyon se kuch sawalaat se

RA: acha aap ko kia lagta hai k rozmara surveillance k kaam k elawa joh zachki hain aur nauzaida bache k hawale se maloomaat farhaam karna chaiye

CHW: jee kyun k bohat se log aise hote hain matlab kuch uneducated hote hain jin ko kuch bhi pata nahi hota pregnancy k hawale se ya bachon ki deakhbhaal k hawale se jab hum jaate hain toh unhein hum cheezein batatey hain guide karte hain toh who hamara shukriya ada karti hain k aap ne hamein bohat achi achi cheezein bataein aur hamein is cheezon k baare mein nahi pata tha ziada kyun k kuch first pregnancy wali khatoon joh kam umar ki bhi larkiyan bohat milti hain toh who first pregnancy ho ya kuch aise bhi hote hain jin k pregnancy 3 4 ya 5 bhi hoti hain leikin joh cheezein hum bata rahe hote hain unhein guide kar rahe hote hain anc k hawale se bachon k sehat k hawale se toh who kehti hain k haan yeh cheezein hamein pehle pata nahi thi toh yeh hamein aap se pata chalin yeh achi baat hai hamein aap ne yeh cheez bataayi is tarhan se who hamari baton pe amal karti hain toh hamein acha lagta hai hamein bhi hota hai k hum guide karein logon ko mazeed bataein ta k aane wali naslein joh hamari who achi tarbiyat yaafta hon

RA: toh matlab k aap k khayal mein joh joh aap ne unhein information di hain aap ne joh joh cheezein bataein hain toh us se aap ko lagta hai k aap ko munasib training haasil hai

CHW: thora hamein sikhaya bhi gaya hai leikin hamein mazeed kuch sikhaayeinge thora trainings hongi toh acha rahega na matlab kaafi cheezein toh pata hai itna tajurba toh kaafi saalon se kar rahi hun surveillance mein toh kaafi idea hai leikin proper nahi mili jaise hota hai medically

RA: kis kisam ki training honi chaiye

CHW: matlab bachon k health k hawale se anc k hawale se

RA: toh abhi joh aap ko training mili hai who aap ko abhi de rahe hain ya pehle se

CHW: nahi pehle bhi waqtan waqtan training hoti rehti hain temperature k hawale se aur RR k hawale se un k bachon ki sehat k hawale se k hum danger sign kaise deakh sakte hain toh who danger sign hamein agar lagta hai k waqai mein yeh danger sign bache mein hai phir us hawale se mother ko guide karte hain k bache mein yeh yeh problems hain aap hamare center laayein ya kareebi kisi clinic mein dikha lein kyun k mostly hamara center thora dur parta hai na hamare extension k lehaaz se toh thora pick n drop ka issue hota hai toh phir hum kareebi clinic ka bhi advise deite hain k deakho agar aap wahan nahi la pa rahe ho k hum aap ko kisi tarhan ki facilities nahi de pa rahe toh aap zaroor kisi doctor ko dikhao kareebi dikhao ya kisi hospital le jao jahan aap ko easy lage toh aap jab is tarhan se guide karte ho toh unhein acha lagta hai hamein bhi acha lagta hai k hum ek zindagi save kar rahe hain

RA: matlab aap keh rahi hain k aap ko training mazeed milni chaiye health se related

CHW: health se related milni chaiye kyun k aaj kal toh ek se barh k ek facilities a rahi hain aaj kal mein naye naye tareeke hain nayi nayi cheezein hain toh pata hona chaiye logon ko kynn k hum field pe hain joh toh hum aage dusron ko jab batate hain toh matlab khud us cheez ki knowledge honi chaiye ta k hum aage community mein phela sakein

RA: jaise aap ne kaha na facilities joh nayi nayi cheezein aayi hain

CHW: jaise anc mein kaafi cheezein aisi hain joh pehle daur mein kehte hain aise nahi karo aisa hojaayega bachon ko ziada lappeit k nahi rakho ziada khol k nahi rakho toh medical mein hamein bataya tha k kis tarhan se bache ko rap karna hai kis tarhan se bache ki deakhbhaal karni hai mother ko kia kia cheezein pregnancy mein khaani chaiye pehle toh yeh hota tha nahi yeh garam cheezein se yeh mat khao andaa mat khao aur gosht mat khao yeh mat khao bohat saari cheezein se manah karte thy leikin hamein medical yeh batata hai k yeh cheezein khaani chaiye pregnancy mein protein milte hain hamein gizaayat milti hai mother ko toh yeh cheezein khaani chaiye mother ko

RA: sahi aur aisi cheez joh aap chahti hon k han hamein agar yeh training mil jaaye jis se hamein faida ho

CHW: training agar milti hi rahe toh faida hota hai kyun k knowledge barhti hai aur jitni ziada knowledge hogi surveillance mein hum utna acha kirdaar ada kar sakeinge jitni knowledge ho utna kam hi hota hai perfect toh koi bhi nahi hota knowledge toh burhaape tak bhi milta rahe toh acha hi hota hai k us se hamein bhi faida hota hai aur hum jis community mein kaam karte hain community ko bhi faida hoga

RA: acha aap k khayal mein maaon or nauzaida bachon ki deakhbhaal mein sahulatkaar k taur par aap ka kia kirdaar hai

CHW: hum counseling karte hain unhein k mothers ko kis tarhan se rehna chaiye kis tarhan se bachon ki deakhbhaal karni chaiye aur yahan hum medical k hawale se batate rehte hain k agar bache ki tabiat kharab hogi yeh konse sign hon foran doctor k paas le k jana chaiye waise bhi aaj kal toh khaansi zukaam ka season chal raha hai yeh bhi bohat khatarnaak hai toh is k liye bhi hum guide karte hain k maamooli agar khaansi zukaam ho phir bhi aap ek dafa doctor k paas a k laazmi dikhaayein k aaj kal bilkul environment hamara bura chal raha hai bohat ziada aaloodgi wagera is tarhan ki cheezein paayi jaati hain toh foran hamein doctor se zaroor rooju karna chaiye dono maa aur bache ko apni sehat ka khayal rakhna chaiye doctors k paas jana chaiye aur apni sehat ka khaas khayal rakhna chaiye

RA: yeh medical joh aap bata rahe ho yeh aap joh cheezein unhein batati hain aap ko bataya gaya tha pehle se hi cheezein joh aap ne unhein batai hai

CHW: nahi hamein jab hum shuro mein aaye thy toh hamein bhi thora bohat pata tha itna ziada nahi jab hum field pe jaate thy beech beech mein waqtan ba waqtan trainings hoti hain hamein danger sign ya medical k hawale se medical mein yeh cheez hai is tarhan se hamein karna chaiye haamla khawateen k liye hamein bataya jaata tha guide kiya jaata tha ta k yeh yeh

cheezein hain joh haamla khaatoon karein toh un k liye faide mand hongi kin cheezon se matlab ek pregnant hain joh matlab shuro mein abhi conceive hi kiya hai toh wazan wagera kam uthaayein is tarhan se hamein bataya jaata tha iron ki cheezein sabziyaan daalein in cheezon ko khaane se unhein sehat proteins or vitamins wagera ziada mileinge matlab kaafi cheezein hamein nahi pata thi joh hamein is field mein a k pata chali or hamein matlab trainings di gayi hain inshaallah hum umeed karte hain aage bhi di jaayeingi

RA: acha yeh bataiyega k surveillance k kaam k doraan aisi konsi cheez jinse aap ki hausla afzai honi chaiye

CHW: jee kyun k hum community mein response deakhte hain toh hamein acha lagta hai k community bhi khush hoti hai k hamare liye bhi koi araha hai jaise hum medical idaarey mein jaate hain hum wahan pay karte hain lekin jab hum field pe jaate hain toh un ko hum free mein har cheez provide karte hain counseling karte hain maaloomaat deite hain aur ghar mein ja k bache ki poori assessment hoti hai maa ki anc wagera karte hain toh hamein acha feedback milta hai field par k hamare liye itni saari facilities a rahi hain toh hamein bhi khushi hoti hai yeh cheezein deakh kar aur hamare bosses bhi hamare kaam ko deakh kar appreciate karte hain toh hamein acha lagta hai k hamare surveillance k kaam ko appreciate kiya jaraha hai

RA: aur community ko kaisa lagta hai

CHW: community ko bhi acha lagta hai hamare liye muft mein facilities mil rahi hain thek hai joh demands hain community ki woh hum poori nahi kar paate kyun k un ki demands bohat bari bari hoti hai bohat matlab achi achi medicines chaiye agar bache ko halka sa khaansi zukaam bhi hai unhein powder wali dawa chaiye demands hain hum bemari k lehaaz se doctor ko dikhaate hain who phir us lehaaz se medicines deite hain toh who thora sa karte hain lekin phir bhi free mein un ko medicines milti hai is se bhi who khush hote hain k hamare liye kaafi help hoti hain

RA: agar demand poori nahi karte phir

CHW: phir thore se refusal hote hain

RA: acha is tarhan se koi aaya hai refuse

CHW: is tarhan k bohat ziada toh nahi lekin yeh k hamara area thora dur ka hai toh who thora karte hain k hamein conveince di jaaye hamein achi medicine di jaaye ya aksar ki toh yehi demand hoti hai k hamein ghar pe la k cheez di jaaye lekin phir hum unhein guide karte hain k hum har cheez ghar pe la k nahi de sakte for example jaise k agar aap ko bukhaar horaha hai khaansi zukaam ho raha hai chalo hum bukhaar toh check kar leinge leikin khaansi zukaam kis nauyat ka hai yeh toh doctor check kareinge doctor check kareinge toh medicine deinge toh hum is trahan se ghar mein medicine nahi de sakte toh hum unhein guide karte hain k aap ko center aana hai doctor ko checkup karwaayeinge phir hum joh who dawai recommend kareinge who aap ko di jaayegi matlab koi bhi dawa hoti hai who bohat hard matlab agar mujhe sirf flu hai toh mein aap ko de dun powder wala syrup us se khuda na khwasta koi nuqsaan hojaaye toh isliye hum koi bhi medicine ya treatment nahi deite doctor k paas aayein check up karwa kar phir us lehaaz se treatment dein phir kuch toh maan jaati hain kuch toh kehte hain hum nahi a rahe bohat dur hai hamein aane jaane ka issue hota hai transport nahi hai yeh who hamein

khud le kar jao chor k bhi aao aur agar hum center aayein toh hamein first priority mile k nahi hamein pehle deakho

RA: koi aisa waqiya jab aap community mein gaye ho surveillance k doraan koi mushkil aayi ho koi rukaawat aayi ho

CHW: mushkil toh bohat aati thi jaise jaate hain hum aksar toh gharon mein bhi mushkilaat aati hai k hum gaye haamla khaatoon k paas us ko hum ne agree kiya yahan scan karwaane k liye lane k liye woh toh agree hogayi lekin tayar bhi hogaye hum lane k liye lekin jab hum nikalne lage toh foran se saas peeche se agayi k nahi aisa nahi hosakta who nahi jaayegi hamare gharon mein aurton nahi jaati scan nahi hota yeh nahi hota woh nahi hota aisi hamein bohat saari cheezein milti hain bohat saari problems hoti hain phir hum unhein guide karte hain k deakhein k scan machine itni bari kahin nahi ja sakti na toh hamein le k jana parega lekin kuch hoti hain k agree hojaati hain joh samjhti hain aur kuch nahi agree hoti

RA: aap ne kaha k saas ne manah kiya

CHW: saas ne diya kyun l k ja rahe ho who kehti hain k hamare yahan riwaaj nahi hai maslan kuch traditional baatein ajaati hain kuch ana parusti k nahi hum muft mein ja k kyun karwaayein hum paison se karwa leinge kaafi saari cheezein hoti hain joh hamein face karni parti hai manah kar

RA: yeh aap ko surveillance mein rehte huye face karna para

CHW: je je surveillance mein rehte face karni parti hai aur aksar hamare sath kaafi field pe aate jaate bhi issues hote hain k jaise kisi ne keh diya k hum raaste mein ja rahe hain bohat saare larke khare hain hooting karty hain yeh who k abhi jaise corona ka season chal raha hai aksar yeh misaal de rahe hain hum mask laga k ja rahe hote hain na field pe mask toh hum utaarte nahi hain medical se related hai toh agar hum in cheezon ko follow nahi kareinge toh hum pe bohat saari hooting hoti hai who corona jaraha hai who deakho who corona jaraha hai toh is tarhan se kaafi cheezein face karni parti hain hamein har tarhan k comments sunne parte hain field par mothers k logon k galiyon mein khare ho k

RA: yeh joh comments aap pe kar rahe hote hain jaise corona agaya toh kia maayein aap k sath istarhan se kar rahi hoti hain treat

CHW: nahi mothers nahi joh galiyon mein beithe hote hain larke ya bas samjh lein koi kaam nahi hain un ko faarig inhi kaaom k liye beithe hote hain comments karne k liye

RA: acha aur koi mushkilaat aayin surveillance mein rehte huye

CHW: mushkilaat toh bohat aati rehti hain lekin mushkilaat ko maaat deine k liye hamare surveillance walon ka kaam hai jaise field par aksar hum jaate hain toh areas aise hain k guzarna bohat mushkil hota hai gattron ki problem kutton ki problem logon ki problem matlab refusals abhi recently mein monitoring kar rahi thi toh ek uncle aaye toh hum ne gate knock kiya data leine k liye toh jaise unhon ne gate khola toh ek dam hyper hogaye k kyun aate ho tum log nahi batana hamein kuch bhi chale jao yahan se hum ne unse pyar se bhi baat ki k uncle aap

hamari baat toh sunein uncle toh kuch sunney ko tayar hi nahi thy bas daant pe daant nahi aaya karo hamare ghar kitni dafa bola hai tum logon ko delete kar dou hamara naam kyun rakha hai tum logon ne hamara naam filon mein phaar dou saari filein hamara naam mat rakho hamein nahi jana us idaare mein hamein nahi chaiye koi bhi facilities hamein kaafi surveillence mein reh kar bohat saari problems face karni parti hai

RA: aur yeh kitne gharon mein aisa hua hai ek dou ghar hain

CHW: surveillance mein kaafi hota hai agar un k(respondent) a mood nahi hai data deine ka toh woh suna deite hain phir jab acha mood hota hai tab appreciate bhi kar dete hain k aao beitho lekin kuch toh aise hote hain totally refused hotay hain..kehtay hain hamare gate k aas paas bhi nahi dikhna

RA: aisa bhi kehte hain

CHW: aisa bi kehte hain agar hamare gate k paas dikhe toh hum aap pe case kar deinge aisa bhi hota hai surveillance ek bohat tough job hai log isay lightly leite hain shayad k surveillance mein hai kia karna kia hai ghar pe ja k toh data hi toh leina hai leikin who data bhi kis tarhan ka milta hai yeh hum log jaante hain kin kin problems ko face karte hain hum kis tarhan se hum gharon pe pohanchte hain traffic k issues phir raaste kharab raaste mein sau (100) tarhan k masle masaail tab ja k ek ghar pe hum pohanchte hain aur us ghar mein pohanch k data leine mein bhi problems hoti hai agar ghar mein 3 afraad hai ek agree hota hai data deine k liye toh dusra kahe jao hamein nahi deina data phir teesra k nahi le lou nahi rehne dou is tarhan se un ko guide karna mushkil hota hai lekin unhein guide kar k hum data le k aate hain surveillance is a tough job

RA: acha toh aap ko lagta hai k surveillance tuff hai

CHW: lekin acha hai hamein kaafi tajurbaat huye surveillance mein rehte huye

RA: aap ne kaha na kaafi face karna para

CHW: bohat saari problem face karni parti hain matlab har lehaaz se ab toh yeh corona ka season chala us ko le k face karte hain toh us se pehle bhi kaafi problems chalti rehti hain toh un ko hum face kar k aage barh rahe hain step by step aage

RA: aur is k elawa

CHW: convince ki problem hamare area mein k matlab convince honi chaiye center lane le jaane k liye treatment k liye ta k hum itni dur kaise jaayein hamare paas toh convince nahi hoti hum unhein guide karte hain deakho har waqt toh convince nahi hai aur agar hum hospitals mein jaate hain jaise hum for examples Jinnah gaye wahan toh hamein koi convince toh nahi de raha na Jinnah wale toh nahi a k hamein le ja rahe hamein khud hi jana parta hai khud hi jaate hain lineon mein lagte hain wahan beithte hain medicine phir leite hain toh yahan toh itna dur bhi nahi hai toh aap log aao achi facilities hain hamare idaare ki taraf se hum aap ko muft mein de rahe hain is k liye hum aap se pay nahi maang rahe k hamein itni fees chaiye hamare yahan itne paison mein elaaj hoga bilkul muft doctor check karte hain medicine bhi muft milti hain sirf

aap ko kiraya hi kharch karna parega toh is tarhan hum unhein guide karte hain toh kaafi families agree bhi hojaati hai khud se yahan aati hain dikhaati hain doctors ko mothers aati hain haamla khaatoon hum unhein batate hain k yahan hamari gynae ki achi doctors hain who aap ko check kareingi medicine bataeingi likh kar bhi deingi toh hamare centers se milti bhi hai medicines toh aap k liye achi bhi rahegi

RA: acha aap k khayal mein konsi cheezein community mein CHW k kirdaar ko behtar bababey mein madad kar sakti hai

CHW: matlab un ko guide karna k un k trainings karna alag alag tareekon se un ko batana anc k hawale se batana neonatal babies k hawale se un ko batana k nomaulood bache ki deakhbhaal kaise karni hai un ko kaise rakhna hai toh yeh saari cheezein worker ko training waqtan ba waqtan deite raheinge toh woh aage community mein in cheezon ko provide kareingi surveillance mein community mein acha change aayega

RA: acha lekin aap ka kia khayal hai community mein aisi konsi cheezein hain joh aap ko lagta hai k aap k kirdaar ko aur behtar bana sakti hai

CHW: community se hamein kaafi madad milti hai jab woh hamein acha response deite hain hamari baton ko appreciate karte hain toh hamein kaafi madad milti hai apne kirdaar ko aage barhaane ki is se improvement karne ki aur kis tareeke se hum un k liye soch sakte hain jaise un ko liye hum mazeed hum kis tarhan se un ko facilities kar sakte hain kia kia facilities hum unhein de sakte hain apni taraf se apne idaare ki taraf se joh facilities milti hain hum kis tarhan se un tak pohcha sakte hain

RA: sahi toh yeh hai aap chahte hain hum aage joh hain facilitate kar sakein

CHW: Jee apne community ko facilitate kar sakein har lehaaz se aur un ko counseling kar sakte hain achi aur booklet k zariye un ko dikha sakte hain suggest kar sakte hain jaise k hum surveillance mein kar bhi rahe thy hamein haamla khatoon ki anc ki book di gayi thi us se hum unhein guide karte thy dikha k un cheezon ko deakh k samjh k kaafi improvement aata hai community mein

RA: sahi acha aap ki raaye mein household ki sateh par hamal aur nauzaida bachon k hawale se konsi maloomaat deini chaiye

CHW: un ki sehat k hawale se aur education k hawale se ta k jis mein improvement a sake har elaake mein k hum kis tarhan se un ki zindagiyon ko behtar kar sakte hain

RA: matlab improvement kis tareeke se laani chaiye

CHW: in ko achi achi facilities se aagha kar k k aaj kal bohat saari facilities hain agar aap in ko use kareinge un mein improvement aayeingi kaafi bohat saari cheezein hain jaise sehat k hawale se bohat saari maloomaat nahi hoti jaise mein ne bataya tha k jaise haamla khaatoon ko for example lein us ki first pregnancy ya 3rd 4 hai toh kuch cheezein medical k hawale se bata rahe hote hain unhein un cheezeon k baare mein pehle nahi pata hota that oh who first time un cheezon ko deakh rahi hoti hain toh un cheezon se khush hoti hain hamari counseling se

hamari provide ki huyi maloomaat se khush hoti hain k hamein yeh cheezein pehle pata nahi thi acha hua aap ne bataya in cheezon k baare mein ainda in cheezon par madd e nazar rakhte huye aage barheinge

RA: sahi acha kia aap k paas koi aur raaye ya khayalaat hain joh aap apne surveillance k baare mein batana chahti hain

CHW: surveillance k hawale se mein yeh kehna chahti hun k surveillance ek aisa part hai jis mein hum jitni bhi improvement karte rahe kam hai for example trainings k zariye aur counseling k zariye k hum kis tarhan se unhein (Community) guide kar sakte hain hum unhein achi achi cheezein guideline k liye samjhaayein counseling karein toh kaafi improvement ho sakti hain hamare field sites mein surveillance mein

RA: aur kia kar sakte hain improvement

CHW: sehat k hawale se deakhein hum is tarhan improvement chahte hain k log aksar mein ne deakha hai k bache chote chote joh bahir galiyon mein khel rahe hain ghoom rahe hain mitti dust yeh saari cheezein hum unhein yeh cheezein samjhaate hain k bahir bohat ziada bache mitti mein raheinge nange paaon sardi lagne ka jaise aaj kal season shuro hogaya hai sardiyon ka toh sardi lag jaayegi bache ko toh bacha beemar par jaayega aap is ki thori deakhbhaal karein kapre wagera pehna k rakhein aisi cheezon se hum unhein guide karte hain

RA: aur surveillance mein kia hona chaiye

CHW: surveillance ko hum mazeed behtar bana sakte hain hum is tarhan se k hamare joh household hain toh eke k ghar mein ja kar counseling karein un ko samjhaayein aur unhein sehat k hawale se bataein

RA: nauzaida bachon k hawale se

CHW: nauzaida bachon k liye mostly kuch mothers ko toh bache ko deakhna hi nahi aata feed karwana nahi aata toh yeh cheezein hum unhein samjhaate hain feeding ka tareeka batatey hain k kis tarhan se feed karwana hai hamein trainings mein bataya jaata hai usi tareeke se hum unhein batatte hain un ko kaafi madad milti hai nauzaida bachon ko un ki deakhbhaal karne mein toh is cheez se hamari surveillance se log kaafi khush bhi hai k aap achi maaloomaat deite ho hamein guide karte ho

RA: aur koi raaye joh aap batana chahti ho

CHW: aur koi raaye nahi

RA: bas

CHW: bas

RA: chalein shukiya bohat bohat…

Date: 20102020

RA: jee name aap ki umar kitni hai

CHW: meri 24

RA: aap ki taleem

CHW: inter

RA: aur aap k kaam ka tajurba kitne saal ka

CHW: aa 4 years

RA: surveillance aap ko kitne saal hogaye hain aap kaam kiye huye

CHW: mujhe chotha saal chal raha hai

RA: chotha saal chal raha hai

CHW: jee

RA: ok .. toh aa mujhe bata sakti hain name k aap ki joh rozmara ki joh routine hai aap ka kaam hai us hawale se aap mujhe bata sakti hain k aap kaisa mehsoos kar rahi hain

CHW: jee hamein acha feel hota hai k hum community mein jaate hain un ko hum batate hain k hum aga khan health center se aaye hain aap ko kia kia sahulat di jaati hai k hamare center mein aur hamara is se knowledge barti hai k hum deakhna chah rahe hain k log hum se kia improve le rahe hain kia facility hum un ko de rahe hain aur ziadatar log joh hai na who hamein yeh bolte hain pehlay k hamare kuch project aise hote thy jis mein hum pehle mein thi AMANI mein AMANI mein jis project mein thi wahan pe hum daily ek aurat ko follow karte thy us ka daily visit hota tha us ka hamein 1 day ka baby chaiye hota that oh hum us PW ko daily daily follow karte thy ta k who ek din ka baby miss na ho hamein who 0 day ka baby chaiye hota that oh hum daily un ko follow karte thy aur unhein gaari bhi deite thy aur unhein check up karwana ho toh unhein le k bhi aate thy aur le k aate thy un ka ultrasound wagera bhi karwaate thy leikin ab who project nahi hai toh who facility bhi nahi hai gaari wagera ki toh abhi hamein wohi log bolte hain pehle toh aap log gaari mein le k jaate thy PW ko ya un k sath un k koi ghar wale family member joh hote thy who bolte thy acha aap ja rahi hain toh mein bhi sath chalti hun hum apne bachon ko check karwa leinge toh yeh unhein bohat achi lagi service hamari thi yeh sahulat un ko bohat achi lagi lekin hamare paas ab who service nahi hai gaariyon ka abhi hamein kuch issue horaha hai toh log bolte hain k aap log hamein le k nahi jarahe hum afford nahi kar sakte joh private mein jaayein aur govt hospital dur hai aga khan ka health center bohat acha parta hai hum wahan jaate hain doctor se bhi acha rujoo hota hai elaj bhi sahi hota hai kyun k elaj bohat acha karti hain leikin masla sirf yeh hai aane jaane ka agar hum afford kar paate toh hum kisi bhi kareebi private clinic mein ya hospital mein ja paate leikin hum afford

nahi kar pa rahe toh hum jab hi nahi pa rahe isliye aap log phir se wohi survey hamare sath rakho ya hamein gaari mein le k jao leine aao ya hamare bachon ko le k jao toh yeh sahulat phir se muhaiyaa ki jaaye leikin hum ne kaha who project k hisaab se aati hai sahulat abhi filhaal project aisa koi hai nahi agar aap log khud se asakte ho koshish karo k aap log khud se ajao phir hamare yahan wohi elaaj wagera horahe hain kyun k aap khud se aana chah rahe ho toh asakte ho leikin filhaal hamare paas gaari nahi hai joh aap ko hum le k jaayein yahan pe le k elaaj karwa k phir chor k aayein leikin inshaallah aage project aayeinge toh zaroor koi na koi sahulat hum aap ko zaroor deinge

RA: yeh toh aap ne kaha jab aap AMANI k project mein aap deakh rahe thy toh us waqt aap ko pareshaaniyan aayin thi

CHW: je je nahi abhi bhi a rahi hai

RA: abhi bhi a rahi hain

CHW: haan jee abhi who isliye a rahi hain kyun k hamare paas pehle yeh sahulat thi na gaari mein patient wagera le k aate thy aur abhi nahi hai toh hamein saare patients yehi bolte hain k aap log aate ho hamare yahan se visit karte ho naam likhte ho aur hamara kia faida hai toh hum unhein bolte hain k aap log aayein hamare center visit karein bachon k elaj hote hain padaish se le k 5 saal tak aur hamare doctor hain PW k checkup wagera karti hain who bolte hain hum kaise aayein bohat dur parta hai aur na hi hum ne deakha hai ziadatar aurton ne deakha bhi nahi hai kyun k yahan pe project k hisaab se gaariyan khud le k aati thin un ko raaste nahi pata ziadatar log ko raaste nahi pata aur bolte hain k waise hi kiraya itna kharch hoga toh hum paas kahin chale jaate wahan itna dur center kaise aayein sab ka yehi ziadatar issue hai toh hum ne kaha inshaallah aage project aise zaroor aayeinge joh aap logon ko phir le k bhi aayeinge center leikin filhaal toh aisa nahi hai hamare paas itni gaariyan nahi hai joh aap logon ko le k bhi aayein center toh abhi mein is field ki baat kar rahi hun jis mein hum jaate hain toh log yehi bolte hain k aate ho naam likhte ho naam likh k chale jaate ho is mein hamara kia faida hai hum bolte hain faida hai leikin aap logon ko khud aana parega

RA: hmm toh surveillance k kaam joh aap kar rahe ho yeh toh aap ne bataya tha project k hawale se thek joh ab surveillance mein kaam kar rahe ho us hawale se aap mujhe bata sakti hain kaisa mehsoos kar rahi hain aap surveillance mein yeh toh aap ne bataya tha AMNI ka project tha yeh problems aayin aura b bhi a rahi hain

CHW: haan

RA: surveillance mein aap ko kaha is tarhan se

CHW: nahi ziadatar toh nahi hai leikin kam log aise hain joh refuse karte hain. Refuse isliye kar rahe hain kyun k NIC joh hota hai who hamara zaati cheez hai joh kisi ko toh geir logo ko nahi deina chaheinge kyun k ziadatar abhi aa haadsaat wagera bhi bohat ho rahe hain har kisi pet oh insaan trust bhi nahi kar sakta toh hamare paas card hota hai card pehna hota hai toh hum un ko dikhaate hain hum aise jaati log nahi hai hum aku se hi aate hain toh who hamein ziadatar aurtein dikhaati hain abhi joh hum kar rahe hain na survey us page mein ek NIC ka bhi option hai NIC ka number likhna hai toh compulsory nahi hai leikin agar deina chahein toh who

hum likh sakte hain toh hum jab bhi NIC maangte hain na toh woh bolte hain NIC hum who kyun dein haan ziadatar har kisi ka yehi sawal hota hai NIC NIC kyun dein hamara toh personal cheez hai aap ko kyun dein toh NIC kyun maang rahe ho hum bolte hain nahi hum us mein se date of birth deikhege toh date of birth khud hi bata deite hain NIC nahi de rahe phir koi koi deite hain koi ziadatar nahi deite who bolte hain nahi NIC aisi cheez hai who number likhne nahi deinge kyun k logon ko dar hota hai demaag mein un k zehan mein hum NIC kyun dein

RA: acha dar kyun hota hai

CHW: who bolte hain na k hum ne suna hua hai k kisi k NIC number ki wajha se koi misuse koi cheez huyi hai ya un k number ki wajha se koi maamlaat huye hain is wajha se hum apna number nahi de rahe nahi likhwana chahrahe aap logon pe trust hai aap log kaafi dafa aaye ho kaafi time se is jagha pe kaam kar rahe ho survey kar rahe ho hum aap logon ko jaante hain aap logon ne card dikhaya aap logon pe poora trust hai leikin hamara dil nahi manta k hum NIC ka number dein

RA: acha sahi toh phir kia hota hai agar aap nahi karte hain sahi tarhan se NIC nahi deite hain phir aap kaise convience karte hain

CHW: hum bolte hain aisa kuch nahi hota hum aku se aate hain aku ka health center hain Ibrahim hyderi mein agar aap logon ka dil mutmaain na ho toh aap log hamare center visit kar sakte hain hum koi dokhebaaz ya jaali log nahi hain aap log visit kar sakte hain hum itne saalon se a rahe hain

RA: matlab NIC aap logon ko chaiye kyun tha deakhna kyun parta tha

CHW: who mam us mein number bhi likhna hota hai hai NIC ka joh abhi hum kar rahe hain na kaam abhi joh hamara VR ka joh page fil horaha hai us mein NIC ka number bhi likh rahe hain aur NIC se un ki date of birth bhi likh rahe hain toh is wajha se NIC ki need par rahi thi leikin agar koi aurtein nahi deina chah rahi who compulsory nahi hai likhna who matlab koi deina chahein maangte har kisi se hain agar koi matlab de deite hain toh likh deite hain agar nahi deina chah rahe toh hum convience karte hain aur agar hojaati hain toh de deite hain

RA: kabhi aisa bhi hota hai k kisi kisi k paas NIC nahi hon

CHW: han han

RA: toh phir kis tareeke se leiti hain data

CHW: sirf number leite hain un ka contact number leite hain aur un ka naam un k husband ka naam aur kitne bache hain kitni dafa hamal se huyin ya kitne zaaya wagera yeh cheezein leite hain phir NIC number nahi leite

RA: surveillance mein aap ko lagta hai k acha mehsoos kar rahi hain badnisbat joh aap ka pehle joh project tha us se

CHW: je pehle bhi hamara yehi tha MWSR joh change hua hai VR pehle bhi mein is mein thi phir us mein AMNI k project mein gayi phir mein dusre kisi project mein gayi yeh who ANA project mein aku ANA joh project tha us mein gayi us k baad phir se mein yahan agayi

RA: acha ANA project mein kia hota tha

CHW: ANA project mein hum blood test karte thy jis mein khoon ki kami hoti thi na un ko yahan le k aate thy un ko venofer lagti thi us mein sirf hamare poore project mein chaar log thy do worker aur ek doctor aur ek midwife

RA: toh us mein aap ka role kia tha

CHW: mera ANA project mein worker hum jaate thy field pea a hamein list di jaati thi k aap logon ko in gharon mein visit karne hain phir hum un gharon k visit karte thy PW ki list hoti thi phir hum un k test karte thy test karte thy jin ki bhi blood ki kami aati thi un aurton ko hum le k aate thy center pe who midwife doctor se checkup karwati thi phir midwife unhein drip lagwaati thi venofer ki

RA: sahi acha mujhe batana name aap apne household visit se hamal ya nauzaida bachon ki deakhbhaal mein aap ka us mein kia kirdar tha aur kaisa mehsoos karti thi

CHW: samjh nahi aaya

RA: aap apne household visit se hamal ya nauzaida jis ko kehte hain newborn bachon ki deakhbhaal mein kia kirdar mehsoos karti thi matlab kaisa mehsoos kar rahi hain us waqt k aap ne kaha na hum log jaate thy community mein hum un ko batate thy k hum un ko sahulat dein aisa kuch aap ne bataya toh kis tarhan se bachon ki deakhbhaal k liye maaon ko batati thi

CHW: bachon ko kaise deakhbhaal karni chaiye ya kaise rakhna chaiye un ki sehat ka khayal toh hum jaise field pe gaye agar hamein chota bacha mil gaya newborn toh hum aurat se pehle poochte hain k aap isay apni feed karwaati hain ya upar ki koi feed deity hain toh who aurat bolti thi k nahi mein toh upar ki deity thi mujhe kisi ne bataya nahi k apni bhi deini hai toh hum un ko batate hain k apni feed deini hai aur upar ka toh abhi 6 maheeney tak nahi deina milk agar aap ka milk nahi a raha toh aap de sakti hain who bhi aap doctor se likhwaayein aap aise kisi k kehne pe yeh pilaya that oh aap bhi yeh le lo toh aisa nahi karein aap doctor se likhwaayein feed who pilaayein aur safai ka khaas khayaal rakhein aur thori thori deir baad agar poti peeshi karte hain toh un ko baar baar saaf karein change karein aur ek side se 15 min tak feed karwaayein dusre side se 15 min tak feed karwaayein aur thori thori deir baad jaga jaga k milk dein ta k joh un ki padaishi who hoti hai who kia bolte hain peelia wagera who khatam hojaaye un ko joh bhi danger sign ya masle honge wazan nahi bar raha bache ka who bhi maa ki feed se wazan bhi bar sakta hai toh un ko joh bhi hamein knowledge hai hum saari mothers ko deite hain k yeh yeh aap cheezein karein ta k aap k bache ki sehat ache ban sake

RA: sahi toh jaisa k aap ne kaha aap ko knowledge hai aap ko koi munasib training haasil thi

CHW: nahi aise jaise hamare koi project wale ya hamari NWSR ki training wagera hoti thi us mein hamein bataya jaata tha kia kia bachon ko giza deini chaiye hamal k doraan maa ko kia

giza deini chaiye toh us se hamein thora bohat pata hai k yeh maa k liye yeh cheez achi hai who cheez achi hai maa hamal k doraan kia kia kha sakti hai ya jin maa ko hamal mein khoon ki kami hoti hai who kin cheezon se khoon ki kami poori kar sakti hain ya bachon ko kaise treat karna hai newborn k bhi follow kiye hain newborn check kiye hain newborn k followup kiye toh is mein hamara idea hai k kia kia logon ko batana chaiye k kia kia unhein knowledge deini chaiye

RA: sahi toh aap ko training milti thi kitne kitne arse mein ya koi aisi training mili hai

CHW: jee jee do do teen teen maheeney baad jab se mein kaam pe lagi hun na tab se do do teen teen maheeney baad hamein training di jaati thi k abhi koi bhi new aata tha kaam k aap logon ko yeh form fil karna hai pehle form hote thy abhi toh nahi hai abhi toh Tab hai toh pehle forms wagera hote thy toh abhi yeh form aaya hai aur do teen maheeney baad form change hota that oh us ki training deite thy us mein bhi yeh gizaayat k sawal hote thy k agar yeh cheez khaani hai kia nahi khaani haftey mein yeh cheez kitni dafa khaai hai us hawale se hamein training di jaati thi

RA: aap ko lagta hai k aap ko munasib training haasil hai

CHW: jee

RA: joh joh aap ko samjhaya jis tarhan se aap ko training deite thy ya bas yehi training di gayi

CHW: bas yehi training di jarahi thi

RA: acha toh is training se aap ko kia knowledge mili hai

CHW: yehi k pehle hamein andaza nahi tha hamal k doraan hum direct school se yahan agayi aur mein ne inter bhi private kiya hai toh yeh andaza nahi tha k hamal k doraan kia kia khana chaiye ya bachon ko jaga k bhi feed karwana cahiye bache ka peelia maa k dood se khatam hoti hai yeh bhi mujhe nahi pata tha matlab joh joh cheezein hamein yahan a k pata huyi hai ghar mein ya apne muhaullo mein kehti hun k yeh yeh masle horahe hain toh un ko mein batati hun k yeh hamare center mein koi doctor aayin thi ya koi supervisor aayin thi toh logon ne yeh cheez bataai hai is se faida hai aap logon ka toh mein ghar mein bhi batati hun hamare muhalley mein kisi ko koi masla hota hai ya who khud bhi aati hain poochne k liye aap health center mein kaam karti ho hamare sath yeh masla horaha hai agar aap ko bataayein masle ka hal baataein toh mein unhein bata deity hun k aap apni feed karwaayein yeh bache ko aise saaf rakhein un ko gandagi se dur rakhein baar baar leikin joh pata nahi hota toh zahir hai mein bolti hun k aap center visit karein doctor se checkup karwa lein

RA: acha toh who maan jaati hain

CHW: jee hamare poore mohalley ki aati hain ladies mein yahan pe qareeb hi rehti hun

RA: sahi kabhi aisa k who manah kar rahi hon aap yahin checkup kar lein

CHW: nahi nahi hamare mohalley ki saari aati hain

RA: aa jaati hain sahi acha aap ko kia lagta hai k rozmara joh aap surveillance ka kaam kar rahe ho jis mein nauzaida bache bhi hai maa bhi hain aur joh hamal mein khawateen bhi hain toh un k hawale se aap mujhe bata sakti hain k unhein maloomaat farhaam karna chaiye

CHW: jee karna chaiye joh joh unhein cheez pata nahi hai who unhein who cheezein batani chaiye k yeh yeh cheezein hain aap log karein hamal k doraan kia karna chaiye ya newborn hota hai 1 day ka baby us k sath kia karna chaiye who joh first milk hota hai unhein who deina chaiye who sehat k liye bache k liye acha hota hai yeh unhein saari maloomaat deini chaiye kyun k ziadatar yahan pe gaaon ki aurtein hain na un ko yeh cheezein nahi pata hoti who joh first hota hai hamal who toh gira deity hain k yeh hum nahi pilaate yeh peela peela doodh hai ziadatar who nahi pilaati toh unhein who deity hain training wagera k yeh milk hai yeh zaroor yeh deina chaiye yeh bache ki sehat k liye acha hai is mein bohat taaqat wagera hoti hai toh who unhein deini chaiye ziadatar aurton ko nahi pata hoti joh IH k log hain na un ko ziada yeh cheezein nahi pata hoti in k bachon ko kaise rakhna hai

RA: matlab pehle yeh unhein nahi pata tha is baare mein toh jab aap log aaye ho toh unhein laga hai k ab kuch behtari aayi hai

CHW: jee jee bilkul unhein yeh bhi andaaza hogaya hai k yahan pe ache doctors hain team achi hai aati hain hamare yahan checkup wagera karti hain newborn joh team hain who ghar pe aati hain visit karne yeh un ko bohat sahulat achi lagi k hamare bache ko ghar par hi check karne aati hain ta k joh un ko cheez nahi pata hoti ziadatar aurtein who poochti bhi hain k yeh yeh karna chaiye ya nahi karna chaiye phir hamari team unhein batati hain han yeh waaqe karna chaiye agar ziada koi masla hota hai toh un ko hamare center refer bhi karte hain batate hain k aap hamare center jaayein bache ko yeh thora masla hai ya unki koi danger sign joh hoti hai un ki heart beat teiz ho ya un ki pasliyaan chal rahi hon koi bhi aisa masla hota hai toh hum unhein boltein hain k aap hamare center jaayein wahan pe visit karein doctor hain checkup karwaayein toh ziadatar aurton ka yehi hota hai k hum kaise jaayein hamein le kar jaayein sab ka yehi issue hai

RA: acha aur koi issue

CHW: nahi ziadatar yehi hai

RA: aur koi complain aisi nahi hoti k haan yeh cheez hamein nahi mil rahi

CHW: nahi nahi hamare elaake mein thore kuch refuse hain joh matlab apna data hi nahi deina chahte who shuro se refuse hain

RA: matlab refuse hone ki wajha toh hogi k data nahi deina chahte

CHW: who bolte hain k hamara panel hai toh hum un mein karwa rahe hain hum kisi bhi idaare ko history nahi deina chah rahe kuch bhi nahi likhwaayeinge

RA: acha

CHW: koi matlab PAF base mein hogaya ya army mein hogaya us k apne panel hote hain ya kisi ki Jinnah wagera mein job hoti hai koi kisi ka panel wagera bhi nahi hota na toh who bolte hain nahi hum nahi likhwa rahe ghar aise bhi hote hain joh bilkul bhi data nahi deite

RA: sahi toh un gharon ka karte kia hain aap

CHW: un gharon ka do teen baar visit karte hain agar nahi maante toh refuse mein deite hain

RA: phir refuse kar deite hain

CHW: jee jee

RA: matlab aap mananey ki koshish karte hain

CHW: jee

RA: aur koi aisi mother joh lagta hai k deakhne se hi lag jaata hai k yeh nahi maanney wali kia un ghar ko visit karte hain ya chor deite hain

CHW: je je visit karte hain toh koi koi daantta bhi hai (laugh) baatein bhi sunate hain bolte hain k ek baar manah kar diya phir se aagaye aise bhi bolte hain leikin hamein toh har dafa visit karna hota hai k kia pata logon ka mood change hojaaye likhwa lein toh hamein har dafa visit karna hota hai leikin agar nahi maante toh hum refuse kar deite hain

RA: toh mujhe bata sakti hain surveillance k kaam k doraan aap ko koi rukawaton ya mushkil ka saamna karna para tha surveillance ki baat kar rahi hun

CHW: nahi sirf mein ne bataya na k chand ghar refuse hain baaki aisa kuch nahi joh achi achi family se hain who saare data bhi deity hain apni har cheez batati hain joh bhi hum un se sawalaat karte hain sahi sahi bas easily jawab mil jaata hai

RA: toh aap ko lagta hai k matlab joh bhi aap keh rahe ho un ko samjh a rahi hai aap ki cheezein

CHW: je je haan hum un ko batate hain k hum aga khan health center se aate hain hamara maqsad kia hai yahan aane ka hum kyun aate hain hamare center mein kia kia sahulat di jaati hai abhi yeh joh new project aaya hai is mein PW bhi le rahe hain kuch kam month wale hum unhein yeh bhi batate hain phir ab yahan pe agar visit karne aate hain un ko kam month hote hain yahan pe bhi enrolled hojaati hai joh aana chahein leikin hamara area thora dur hai na toh ziadatar wahan ki aana nahi chahti yahan pe who bolte hain aap log khud le k chalo gaari mein extension area hai na wahan pe thori mushkil hai aane mein jaane mein logon ko

RA: acha aap ko lagta hai gaariyon ka bhi thora sa issue hai

CHW: je je mere khayal se ek gaari rakhni chaiye joh patients hum yahan le k aayein ta k hamara wahan pea cha rujoo banein who hamein sahi se data bhi dein kyun k aksar yeh bolte hain k hamein toh kuch faida nahi hai kyun k hum wahan khud se nahi a sakte ek toh center dur hai agar hum afford nahi kar sakte toh hi hum wahan aana chaheinge k hamara free mein acha elaaj hojaayega agar hum afford kar paate toh hum kahin private chale jaate leikin afford nahi

kar pa rahe tabhi hum aap logon ko keh rahe hain k hamein le k chalo aur aap log le k nahi jaate ziadatar aurtein yehi bolti hain

RA: sahi aap kehte ho k surveillance k doraan aap ko koi rukawatein nahi aayin

CHW: nahi

RA: koi aisi mushkil nahi aayi

CHW: nahi nahi

RA: sahi toh mujhe bata sakti hain k aap k khayal mein maaon or joh newborn hain un ki deakhbhaal mein sahulatkaar k taur par aap ka kia kirdaar hai ek toh community ki baat ho rahi hai aur sahulatkaar facilitate joh karte hain un mein aap ka kia role tha

CHW: matlab hamare center mein joh facilities di jaa rahi hain un mein aur joh hamare area k log hain un mein mera kia kirdaar hai

RA: han

CHW: mein un ko batati hun k hamare center mein yeh yeh cheezein hoti hain mein un ko guide karti hun k aap hamare center aayein kaafi time se wahan kar rahe hain survey toh un ko aitebaar hojata hai k haan yeh sahi keh rahi hain wahan jana cahiye koi koi maan bhi jaate hain leikin dur ki wajha se nahi aana chahte bas masla sirf yeh hai toh mera kirdar yeh hai k mein un ko batati hun guide karti hun k hamare center mein sahulat di ja rahi hai padaish se le k 5 saal tak bachon k elaaj hote hain aur poori vaccine bhi lagti hai poora course complete hota hai vaccine ka aur mothers ko saatwein aathwein mein vaccine lagti hain aur abhi ultrasound bhi horahe hain toh yeh cheezein sab un ko batati hun misaal k taur pe agar hum survey karne wahan nahi jaate toh yeh yeh sahulat unhein pata kaise chalti k yeh cheezein hamare center pe ho rahi hai yeh joh poori worker jaati hain un ka yehi kirdaar hai k who unhein guide karti hain k yeh yeh sahulat hamare center mein di jaati hain bilfarz wahan worker nahi jaati toh unhein pata hi nahi chalta k yeh wahan pe kia horaha hai aga khan mein

RA: hmm jab aap ne sab kuch samjhaya toh kia kabhi inkaar kiye hain jaise k aap ne bataya hai k yahan pe sahulat mil rahi hai

CHW: masla yeh hai na k ek dafa mein hum gaye aurton ko bata k aaye k yeh yeh sahulat hamare center mein di jaati hai aur agli dafa mein hamare baad do teen din baad koi dusri team chali gayi wahan pet oh wohi cheezein us ne bataai toh bolti hain acha yeh cheezein hain hum ko pata hi nahi hai matlab aise log ghoom jaate hain pata nahi kyun k nahi hamein toh pata hi nahi hai

RA: dusri team konsi

CHW: matlab hamare baad koi bhi wahan guzarta hai dusre projects ki ya koi bhi toh who un ko rok k bolti hain k acha aap log kahan se aaye ho toh who bolte hain k hum aga khan hospital se aaye hain acha wahan kia hota hai poochti hain toh agar hamari team batati hain k wahan bachon k elaaj hote hain hamla khawateen ka checkup wagera bhi hota hai toh bolti hain acha

hamein toh nahi pata yahan pe koi nahi aata directly yehi bolte hain yahan pe agar koi nahi aata toh data hamare paas kaise aaya aise karti hain aurtein(laugh)

RA: acha mujhe bata sakti hain k kia aap surveillance k kaam k doraan jaise aap ne bata diya tha rukawaton ka nahi hua aisa kuch toh refuses bhi toh rukawat hi huyi na k aap ko mushkil aayi refuses ki

CHW: haan refuse sahi sahi

RA: aur surveillance k kaam k doraan aisi koi cheez jinse aap ki hausla afzai honi chaiye huyi ho aap ko lagta hai jaise aap ne kaha na community joh hai aap se khush hain aap k kaam se aap ko lag raha hai

CHW: khush hai bhi aur ziadatar nahi bhi

RA: acha zahir si baat hai k aap fakar mehsoos kar rahi hongi aap ki taareef bhi horahi hai community mein

CHW: je je matlab hamare mohalley ki baat le lein kyun k hamare khandaan mein koi bhi job nahi karta leikin un ko acha feel hota hai mein job karti hun health center mein joh cheezein hamein nahi pata hoti toh who hamein pata kar k batati hain joh hamein pata nahi hoti who doctor se pooch k deity hain sehat k hawale se cheezein toh is se meri ammi bhi yahan job karti thi ab who retire hogayin toh un ko fakar hai k mein yahan pe job karti hun

RA: aap ko lagta hai k joh community mein log hain who aap se khush hain leikin joh khush nahi hain woh

CHW: woh ziadatar refuse (kuch khush nahi hain)

RA: toh matlab aur koi aisa waqiya bayan kar sakti hain

CHW: nahi kuch aurtein aapas mein laraai kar rahe hote hain ya un ka mood khayab hota hai toh ek dafa hum 2 block mein gaye thy toh mere sath hum do team thy pehle hamara ek survey tha who kar rahe thy yeh wali cheez nahi thi ab VR nahi that oh hum ek ghar mein gaye ghar pe mein ne knock kiya toh andar se awaaz aayi k ek min ruk jaao toh mein ruk gayi thori deir baad 10 se 15 min phir knock kiya toh koi awaaz nahi aayi toh mujhe laga who andar chali gayi shayad bhool gayin toh mein ne 3 baar aur knock kiya who jaise aurat gusse se nikli jaise mujhe maarne wali ho who chilla k mujhe boli kia hogaya darwaza toroge kia mein ne kaha acha aunty sorry mujhe pata nahi tha k aap andar hi hain apna kaam kar rahi hain sorry aunty mujhe maaf kar deina woh aunty thin toh who keh rahi thi acha kia darwaaze torne k liye aaye ho yahan pe kia hogaya hamare darwaaze toroge itni baar koi karta hai knock mein ne kaha acha sorry aunty maaf kar deina 3 baar toh kiya hai zahir si baat hai hum un se toh lar nahi sakte kyun k community hai hum health worker hainn hamein apni zabaan achi rakhni chaiye toh mein ne kaha acha aunty maaf kar dein sorry aap ki bahu hai us ka poochna tha who shayad PW ka followup that oh who hamal se hai delivery huyi hai ya nahi han han hogi toh hum khud hi bata deinge leikin yeh baar baar nahi aaya karo who aunty thora gussa karti thin

RA: acha toh aap ko laga k yeh problem aayin yeh kab ki baat hai

CHW: yeh takreeban 1 - 1.5 saal pehle ki baat hai

RA: surveillance mein hua hai is tareeke se

CHW: nahi nahi yahan pe aisa nahi hua sirf ek k sath hua tha un ko kutte ne kaata tha

RA: acha kin ko kata tha

CHW: yeh hamari worker hai ruksana baaji

RA: acha yeh surveillance mein rehte huye aap bata rahe ho

CHW: je je

RA: ok aap k khayal mein konsi cheezein community mein CHW k kirdar ko behtar bananey mein madad kar sakti hai

CHW: gaari (laugh) joh un ko hum sahulat dein hamare patients ko joh PW hon ya koi sick bohat ziada sick baby ho jin ko foran se hospital le k aana pare un k liye gaari honi chaiye who hamare kaam ko aur behtar bana sakti hain ta k who ek doosre ko bataeingi k yeh cheez abhi sahulat bhi de rahi hainn gaari mein bhi le k jarahi hain ta k un ko bhi sahulat mil jaayegi na toh who refuse bandon ko bhi bataeingi k yeh hamein sahulat de rahi hain toh un ko yeh lagega k haan yeh hamara bhi acha soch rahi hain hamein bhi likhwana chaiye toh refuse log bhi agree hojaayeinge

RA: sahi aap ki raaye mein gharon ki sateh par hamal ya nauzaida bachon k hawale se konsi maloomaat deini chaiye

CHW: un ko yehi deina chaiye k hamal k doraan woh yeh cheezein khaayein joh un ko faida dein agar un mein blood ki kami hai toh who red phal khaayein aur kaleijee khaayeinge jin se un ka blood barhe aur sabziyaan khaayein patto wali sabziyan phal khayin

RA: yeh cheezein aap chah rahe ho k yeh maloomaat unhein deini chaiye

CHW: je unhein deini chaiye

RA: aur is k elawa

CHW: is k elawa newborn ko mother feed ki need hoti hai feed karwaayein aur woh joh doodh gira deity hain first wala who na giraayein un ko dein aur upar ki feed agar karwana chahti hain toh who doctor se likhwaayein agar nahi a rahi apni feed toh upar se koi bhi feed deina chahrahi hain toh who doctor se likhwa k dein khud se na dein aur safai ka khaas khayal rakhein yeh maloomaat unhein deini chaiye

RA: acha toh aap k paas koi aur raaye ya khayalaat hain joh aap apne surveillance k kaam k baare mein batana chahti hain

CHW: mere khayal se bas ek gaari honi chaiye aur bohat sick baby yahan pe le k aayein aur joh inkaar karti hain yahan aane se kyun k bolte hain na extension se patient nahi aate kyun k

extension ka area bohat dur hai aur who bolte hain k hum afford nahi kar pa rahe kaise aayein masla saara yeh hai k ek gaari honi chaiye unhein le k aane wali phir hum who ek din rakh leinge kyun k hamari jitney bhi survey ki team hai un mein mein haftey mein har ek team ko yeh kaam diya jaaye k ek haftey mein aap yeh patient loge ya Allah na kare bohat ziada sick baby milta hai toh hum ek dusre ko aapi ko bata deinge lubna aapi ko k yeh yeh ghar hai is mein bohat sick baby hai toh un k paas agar gaari ho yay eh gaari hamare paas ho toh hum unhein inform kar k hum baby yahan le k aayein phir us din hamara kaam disturbe hoga leikin jab hum unse data le rahe hain un ka kaam kar rahe hain apna kaam karein toh unhein bhi koi facilities deini chaiye

RA: acha aap k khayal mein joh surveillance mein joh aap ki raaye ya khayal hai who hai gaari

CHW: je je

RA: yeh honi chaiye

CHW: je je yeh honi chaiye

RA: acha aur is k elawa aur kia zehan mein hai aap k khayal mein joh aap batana chahti hain

CHW: nahi nahi

RA: kuch bhi khayal nahi joh aap chah rahi hon k aage hum behtar bana sakte hain

CHW: bas

RA: bas chalein shukriya…

Date: 29102020

RA: acha name aap ki umar kitni hai

CHW: 22

RA: aur aap ki taleem

CHW: inter hai

RA: aur aap k kaam ka tajurba kitne saal ka

CHW: 4 years

RA: surveillance mein aap ko kitne saal huye

CHW: surveillance mein mujhe 2 - 2.5 saal huye

RA: 2 - 2.5 saal huye

CHW: jee

RA: thek. Achaname mujhe bataein surveillance k kaam k hawale se aap kaisa mehsoos karti hainn

CHW: surveillance ka kaam ek toh yeh hai k field pe jana parta hai door to door jana parta hai hamein logon se baat cheet karni parti hai har tarhan k log milte hain wahan par toh aisa hai k har nasal k log hain un ko hamein face karna parta hai deakhna parta hai k hamein unhei n kis tarhan treat kiya jaaye toh is hawale se kaafi kuch tajurba mila hai kaam se k kis tarhan logon se baat ki jaaye aur agar koi refuse karta hai toh kis tarhan hamein bardash kar k un ko manaya jaaye kaam k liye un ko bataya jaaye k hum sab aap k faide k liye kar rahe hain hamein is se koi faida nahi hai joh bhi hai community ko faida hai us k liye yeh sab hum kar rahe hain toh confident bhi kaafi aaya hai field k kaam se toh matlab tajurba mila hai hamein

RA: tajurba kis tarhan ka mila hai

CHW: jaise yahin k confident ek toh yeh tajurba hai aur kis tarhan se logon se baat cheet ki jaaye kis tareeke se treat kiya jaaye kyun k yahan pet oh eh hi log nahi hai na har tareeke k log hain yahan pe jaise har zaban k log hain toh zaban mein bhi kaafi fark parta hai baat karne se

RA: acha aur joh aap ne kaha than a k kabhi face bhi karna parta hai aise logon se toh

CHW: jee

RA: phir kis tareeke se aap un ko treat karti hain kaise aap apne baare mein batati hain k aap kon hain kia hain

CHW: jaise kaafi log darte bhi hain hum kisi k ghar darwaaze par knock karte hain toh ziadatar who sahi response nahi deity hain phir hum unse politely baat karte hain hum unhein bulaate hain k hamari baat sun lein hum apne bare mein introduction deite hain batate hain aur hum unhein itna bharosa dilate hain hum apna naam bhi bata sakte hain hum aap k dost hain hum aap ki madad kar rahe hain apni community k liye kar rahe hain phir ja k un ko sahi mehsoos hota hai acha feel hota hai toh who hamein data batate hain phir kaafi saare ghar hain hamare paas joh matlab aise refuse karte hain leikin hum politely ache tareeke se baat kar k samjhaate hain toh who sahi se samjh jaati hain

RA: acha toh aap baat karte ho unhein samjhaate ho toh kia unhein samjhaate ho jis se who raazi hojaati hain

CHW: jaise k abhi joh zamana hai har koi darta hai toh hum unhein yeh batate hain darne ki zaroorat nahi hai hum yahan kaafi saalon se kaam kar rahe hain community k liye kar rahe hain toh hum un ko faide batate hain koi nuksaan nahi hai hum unhein har cheez show karte hain hamara card wagera bhi hota hai toh who hum unhein show karte hain dikhaate hain hum apna naam batate hain toh sab kuch un k saamne rakhte hain aap logon ko darne ki zaroorat nahi hai who har zheez deakhte hain who har cheez jaante hain samjh k phir who deakh kar raazi hote hain kyun k hum koi fraud toh nahi kar rahe hum joh bhi kar rahe hain community k behtreen k liye kar rahe hain

RA: sahi acha toh aap apne household visit se hamal ya nauzaida newborn bachon ki deakhbhaal mein kia kirdar mehsoos karti hain us mein aap ka role kia tha jaise aap ne kaha na jaate ho jaise maaon ko bhi deakhne jaate ho hamal aurton ko deakhne jaate ho nauzaida bache joh newborn hain un ko deakhne jaate ho us mein joh aap ka kaam hai us mein aap kaisa mehsoos kar rahi hain

CHW: us mein yeh hai k na matlab jaise hum koi pregnant hoti hain hum un ko check karne jaate hain toh hum unhein batate hain k aap k liye hamare paas yeh yeh faide hain aur aap ka bacha 5 saal tak hamare paas aap k bache k liye yeh yeh faide hain hum unhein batate hain toh who joh hai na yahan pe aati hain yahan pe unhein acha response bhi milta hai yahan treatment wagera bhi hota hai toh is se matlab k is se yeh hota hai k jaise aa hamein who jaan jaati hai toh hum pe unhein bharosa hojaata hai phir us k baad hamein ziada response bhi acha milta hai agar wahan par bachon k hawale se

RA: acha toh kia batati hain aap unhein joh aap keh rahe ho na k matlab hum un ko batate hain yeh karein who karein

CHW: zidatar toh yeh hota hai na k jaise jis area mein meri field hai extension kit oh wahan par ziadatar log refuse hote hain kyun k who kehte hain k aga khan wale kuch nahi karte bas aise hi aate hain pooch k chale jaate hain toh hum unhein is baat ki awareness deite hain k jaise aap hispatalon mein jaate ho hospitals mein toh aap ka kiraya bhi lagta hai wahan pe ja k aap paise bhi deite ho apna treatment bhi karwaate ho aur khuwari alag si hoti hai yahan pe toh aap aaoge aap ka bas kiraya lagega aap ko na paise deite pareinge yahan aap ka treatment bhi ache se hojaayega yahan doctor bhi ache aate hain yahan par beithte hain yeh yeh timing hai aap log aaoge acha aap ka treatment hojaayega ache tareeke se toh hum un ko yeh sab batate hain whohum itne saalon se kaam kar rahe hain yahan par isliye who hamari baton pe bharosa karti hain yahan pe aati hain agar koi anjaan k paas jaayein toh itna bharosa nahi kartein leikin yeh hai hum unhein batate hain k itne saalon se hum kaam kar rahe hain aga khan walon ko har koi jaata hai sab ko pata hai k itne saalon se larkiyan aati hain yahan pe field karti hain gharon mein aati hain beithti hain poochti hain toh is tarhan

RA: acha is tarhan se lagta hai k aa aur koi aisi kuch maaloomaat deity hain jaise hamal k mutalliq bache se mutalliq

CHW: han jaise agar bache ko kuch ho koi aur beemaari ho toh who share karti hain hum kuch unse poochte hain na k hum yeh de rahe hain toh who usi waqt share karti hain hamare bache ko yeh yeh cheez hain toh hum kia karein toh hum un ko suggest karte hain k aap wahan jaayein doctor beithte hain hum toh doctor nahi hai hamara kaam hai data leina toh wahan pe doctor beithte hain wahan par jao wahan pe har cheez milegi wahan avail milegi wahan aap checkup wagera bhi karwaoge aur is k elawa aap ki koi mushkil ho ya aap ko baat wagera karni ho toh is hawale se aap doctor se pooch sakti hain wahan pe doctor aap k available honge

RA: sahi toh asaani se aap ko lagta hai k who maan jaati hain aap ki baat joh keh rahe ho ya kabhi aisa k nahi maanti aisa kuch

CHW: waise bohat se ghar hain joh nahi maante hain toh baat sunne ko tayar bhi nahi hote hain leikin kuch ghar aise hain k jin ko hum agree kar leite hain apne tareeke se baat kar k ek toh matlab zaban ka bhi hota hai na k insaan kis tareeke se baat kar raha ho

RA: zaban ka matlab

CHW: jaise yahan pe toh sindhi community hai toh koi urdu jaaye toh us ko toh bhaga hi deinge (laugh) toh koi sindhi ja k baat kare toh koi sindhi hoga toh mein agar sindhi hunt oh mein us ko bolongi sindhi mein agar kuch bolongi na toh agar nahi sunne wali hogi Tab bhi mujhe bitha deigi han tum bhi sindhi ho mein bhi sindhi hun tab baat sun legi zaban ka bohat bara masla hota hai

RA: matlab aap ko lagta hai k zaban se bhi fark parta hai agree hojaate hain

CHW: jaise hum gaye thy ek ghar mein toh wahan pe ja k mein toh urdu mein baat ki leikin who thy hazare wale toh mere sath meri dost hazare wali thi toh who agree nahi horahe thy toh meri dost ne un se us zaban mein jawab diya tab ja k unhon ne hamein enter hone diya phir hum ne ja k bache ko check wagera kiya phir unhon ne khaane peene ka bhi poocha humse toh zaban ka toh bohat bara kirdar hai

RA: matlab aisa horaha hai k jaise aap ke rahe ho k aap sindhi mein baat karoge toh joh sindhi hai who aap ko andar aane deingi toh koi aur zaban se baat karein toh phir

CHW: who phir bharosa nahi karte hai nay eh log (laugh) community aisi hai na k bharosa dilana parta hai ziadatar bharosa apni hi matching zaban ka hi aata hai

RA: acha toh yehi hai toh mushkil bhi aap ko isi mein hi arahi hai

CHW: nahi is mein itni mushkil nahi hai kyun k yahan par mere liye mein urdu speak toh nahi hun sindhi hunt oh yahan par toh ziadatar tar sindhi hai na toh is mein mushkil nahi a rahi

RA: acha mujhe bataein k aap ko kia lagta hai k rozmara surveillance k kaam k elawa zachki aur nauzaida bache k hawale se maloomaat farhaam karna chaiye jaise k joh aap ka kaam hai surveillance k kaamm k elawa ki baat horahi hai k joh nauzaida bache hain maayein hain joh hamal se bhi hain un ko maaloomaat unhein farhaam karna chaiye

CHW: hamein jaise k hamare paas treatment hain na sirf humse ziada joh aurtein hain aisi joh afford nahi kar paate toh who poochti hain k yahan par aur kia hota hai test wagera bhi hote hain ya phir koi aur cheez bhi hoti hai ya toh phir pehle joh project aate thy toh yeh log test wagera poore karte thy ab test wagera nahi horahein test wagera karte thy phir un ko gifts wagera bhi deite thy ya samjh lo kuch kharchi de di paise wagera de diye toh abhi log poochte hain toh hum un ko kehte hain k abhi who nahi hai matlab ab khatam hogaya hai toh un ko phir bura lagta hai yeh hona chaiye k un k liye matlab test k liye bhi kuch hona chaiye un ko agar test wagera bhi karwaane ho toh hone chaiye

RA: ziada masla matlab kahan a raha hai

CHW: ziada masla yahan par araha hai na hum log jab un k gharon mein jaate hain toh kehte hain k hamare liye toh kuch karte nahi hain bas aate hain poochte hain pooch k chale jaate hain aur yeh hai k hum jaate hain check up karwaate hain hamara check up hota hai ek do dawai deite hain aur bhaga deite hain toh matlab yeh hai k thori in mein barai jaaye

RA: kis project mein aap ne kaha k kuch deite bhi thy gift bhi deite thy

CHW: pehle AMNI ka project tha aisa jis mein placenta wagera leite thy toh un ko test wagera unse leite thy blood k urine wagera k un ko gifts bhi deite thy jab bacha hota tha aur paise bhi deite thy

RA: toh matlab aap ko lag raha hai k pehle jab hum deite thy aura b jab aap ja rahe ho aap ko sahi se matlab jawab nahi de rahin

CHW: nahi de rahin pehle joh deite thy na toh jin ko mila (incentive) hai na who kehti hain k haan in logon ne hamein yeh yeh diya hai that oh yeh log ache hain aur jin ko nahi mila toh phir un ko gussa aata hai k inhon ne hamein nahi diya tha hamare liye toh kuch nahi karte toh bhaga deite thy

RA: acha toh aap ko bhaga deite thy

CHW: jee bhaga deite thy (laugh) hum un ko manate hain bohat force karte hain manate hain apne aakhari hadh tak unhein manate hain leikin phir bhi kuch ghar aise hote hain joh bilkul darwaza mau pe thap kar k band kar daytay hain (laugh)

RA: acha toh matlab is tarhan se aap k sath

CHW: yeh hai na k matlab hamare paas yeh hona chaiye matlab pregnancy k doraan hote hai na matlab pregnant women tou un k liye yeh hona chaiye matlab thore test wagera karwaane hain joh log afford nahi kar sakte toh aur yeh hai un (pregnant) ko service ka bhi hot ahai masla toh un k liye gaari bhi honi chaiye patient ko hum le k aayein toh yahan a k koi ultrasound ya test wagera karwaayein test k liye hum un ki madad kar dein aur kuch dawaiyaan joh woh nahi le sakte toh who bhi hum available rakhein apne paas phir acha response milega har ghar se koi refuse nahi hoga (laugh)

RA: sahi yeh bhi thek hai. acha aap k khayal mein aap maaon or nauzaida or 5 saal se kam umar bachon ko maloomaat farhaam karne ki aap ko munasib tarbiat training haasil hai

CHW: je training toh hamein poori di gayi hai is k elawa matlab hum khud apna experience bhi un pe aazmaate hain matlab hamein training joh mili hai us tareeke se na maanein toh phir hum apne tareeke se unse baat karte hain politely un ko manate hain thora sa force karte hain toh kahin maan jaaye hum aap k faide k liye kar rahe hain hamara toh is mein kuch nahi hai

RA: kis tarhan ki training di gayi thi aap ko

CHW: kaam k hawale se k kis tareeke se hum counseling karein ghar ja k pehle hum apne baare mein bataein k apne baare mein k hum kahan se aayein hain k kia karte hain hamara kia kaam hai kis hawale se hum aap k paas aayein hain yeh sab unhein hum counseling kareinge

us k baad unhein hum benefits wagera bhi bataeinge k aap ko yeh yeh faide hain community ko yeh yeh faida hai hamare yahan se

RA: aur kis kisam ki training kitne arse mein di jaati hai

CHW: aa yeh hai k jab start hua hai toh hamein first training mili hai 2 3 dino mein training mili hai yahan par jab start hua hai toh tab k hawale se aur kaam k hawale se counseling k hawale se har cheez hamein batai hai aur pregnancy bachon k hawale se k kis tareeke se hamein in se sawaal karna hai kis tareeke se enter karna hai in ko k matlab koi cheez miss nahi honi chaiye aur jab kaam ek circle complete hota hai ek round complete hota hai toh phir is k baad refreshing training milti hai k aap log hum se feedback leite hain k kis tarhan mushkilaat aur error wagera toh nahi

RA: training aap ko mili kab thi

CHW: yeh first joh first training j0h thi yeh june mein ya may mein mujhe thek se yaad nahi

RA: isi saal aur us se pehle

CHW: us (this year)se pehle toh kaafi time pehle mili thi us saal mein mili thi kaafi time pehle mili thi toh bohat bara gap aaya hai

RA: acha sahi toh aap ko lagta hai k aap ko joh bhi training di gayi toh munasib tareeke se di gayi aur aap aage chahte hain training ho

CHW: nahi jaise jitni bhi training hoti hai ache tareeke se munasib tareeke se hoti bhi hai di bhi gayi hain aur hum chahte hain k aage bhi aur ache tareeke se training di jaaye ta k hum aur cheezein seekhein seekhne k liye banda jitna bhi seekhein kam hai, nahi jaise k hamein training mein matlab k refreshing training wagera hoti hain hamein sikhaya jaata hai aur agar koi nayi cheez ajaaye us k liye bhi hamein alag se sikhaya jaaye key eh cheez aisi hogi toh us se errors kam honge na

RA: acha aap k khayal mein maaon or nauzaida bachon ki deakhbhaal mein sahulatkar kay tor par ap ka kia kirdar hai yeh community ki baat horahi hai joh aap deakh rahe hote ho maaon or bachon ko dusri taraf baat horahi hai sahulatkaar in dono k darmiyaan aap ka kia kirdar hai aap apne aap k hawale se kia batate ho

CHW: jaise hum kaam k hawale se ja k un ko yeh batate hain k aap k liye hamare paas yeh benefits hain hamein toh koi benefit nahi hai hum aap k liye aate hain community k liye kaam karte hain toh aap k liye itne saare benefits hain un ko wahan field pe batate hain un ko k community ko itni saari sahulat mil rahi hai hamare wahan se aap le sakte hain

RA: toh phir kia kehti hain who aap kis tareeke ka benefit de rahe ho

CHW: benefit agar woh poochti hain toh hum bata deite hain rakam wagera toh hum nahi deite koi is tareeke ka toh nahi hai yeh hai k aap hospitals mein jaate ho wahan pe ja kar rakam kharch karte ho lekin aap k paas nahi bhi hota hai afford nahi kar paate ho leikin yeh hai k jaana bhi laazmi hota hai toh who sab sahulatein hum aap ko de rahe hain free mein

RA: toh un ka response sahi rehta hai aap k sath matlab aap ko sahi se jawab de deity hain jab aap ne kaha is tarhan se

CHW: jab hum counseling karte hain mehnat karte hain toh hamein ache tareeke se us se response milta hai jab hum ache tareeke se unhein samjhaate hain k aap k liye yeh yeh benefit hain yeh sahulat hain aur yeh sab ho bhi free mein raha hai ya pe koi kharcha nahi horaha hai jo gussay mein hoti hain woh bhi ache se jawab de deity hain

RA: acha jaise aap bata rahi thin a zaban ka who aurtein joh aap se sindhi mein baat karti hain ache se who

CHW: sindhi mein baat karti hain na who bhi refuse hoti hain manah karti hain leikin yeh hai k matlab jis tareeke se aap ki kia zaban hai jaise aap ki bhi wohi zaban hai meri bhi wohi zaban hai toh koi aur zaban wala banda aap se baat karega toh aap pe gussa honge pehlay se hi k kuch nahi karte jao bas jaane dou leikin zaban match hai aur aap unhein apni zaban mein bata rahe ho k hum bhi aap k jaise hain aap se hi maashrey se taaluk rakhte hain jahan pe aap rehte ho wahin pe rehte hain is tarhan se bharosa kar sakte ho phir ja k response deite hain

RA: acha phir response deity hain

CHW: kuch log aise hain joh matlab k zaban k hawale se bhi maan jaate hain sab k baare mein nahi keh rahi is tareeke se toh urdu speak bohat ziada hain leikin counseling kar k unhein bhi mana leite hain

RA: acha surveillance k kaam k doraan aisi konsi cheez jin se aap jis se aap ki hausla afzaai honi chaiye koi bhi aisa waqiya bayan karein joh aap ko lagta hai jaise aap ne kaha k zaban ka masla hai aisa kuch nahi bataya aap ne leikin lagta hai community mein joh aap k zahir si baat hai aap bhi acha mehsoos karti hongi k mere community k log hain who aap k baare mein acha bol rahe hain

CHW: jaise matlab hum jaate hain toh woh kehte hain aga khan wale agaye toh kaafi jagahon pe yeh hai k aga khan wale agaye toh darwaza khulta hi nahi hai phir woh hamein jis tarhan aap ko bataya k counseling kar k manana parta hai (laugh) kaafi log aise hote hain joh moun (face) pe hi band kar deite hain k aga khan wale aaye hain band kar dou

RA: kabhi laga k aap ko koi mushkil aayi ho koi aisi rukawatein aayin hon jaise aap ne kaha k darwaza band kar deite hain toh who kyun kar deite hain band

CHW: woh isi hawale se na hum matlab unhein knock karte hain toh poochte hain kon hain toh hum batate hain apne baare mein k hum aga khan se aaye hain toh who kehte hain k hamein nahi karwana tum log chale jao hamein kuch nahi karwana hum un se poochte hain k kyun kia wajha hai aap hamari ek baar baat toh sun lein hum aap k liye aaye hain toh phir woh kehte hain k aga khan wale toh kuch karte hi nahi hain woh kuch deite nahi hain hum wahan par jaate hain toh bas beith beith k ajaate hain wahan response nahi milta yeh woh toh yeh sab in ki wajha hoti hai

RA: yeh us waqt surveillance k hi hawale se pooch rahi hun joh aap ko us time laga tha joh aap ko mushkil aayi hai aur koi aisi mushkil joh aayi ho

CHW: jee

RA: aur koi aisi mushkil joh aayi ho surveillance mein rehte huye

CHW: aur toh aisi koi mushkil nahi hai bas yeh k agar hamare patients ko na yahan par thori aur sahulat di jaaye na toh woh maan jaayeinge

RA: sahulat kaisi

CHW: jaise k log kehte hain k hum wahan pe a nahi sakte joh a k ultrasound karwaayein hamein chaiye bhi toh un ko gaari bhi provide ki jaaye gaari ek jaaye patient ko lein pick n drop dein patient ko aur test wagera case k hawale se delivery k hawale se sahulat ho yeh sab

RA: toh phir aap ko lagta hai k who maan jaayeingi

CHW: kisi hadh tak joh ghar refuse hain woh maan jaayeinge kyun k kaafi hadh tak ghar isi wajha se refuse hain k bhae aap log kuch karte nahi ho bas aate ho hamara mattha khaate ho tang karte ho phir chale jaate ho data wagera le k chale jaate ho poochte ho hum kyun bataayein aap toh kuch karte nahi ho

RA: toh phir aap log chale jaate ho refuse hote hain

CHW: chale nahi jaate hain hum hum unhein counseling karte hain proper tareeke se

RA: acha toh joh aap keh rahi hain k yeh masla hai refuse ka aap ko mushkil huyi hai surveillance k kaam k doraan

CHW: jee abhi toh kaafi hadh tak yeh masla hai

RA: yehi masla horaha hai aur is k elawa community mein

CHW: ek toh yehi masla ziadatar pesh aaya aur koi kaam k hawale se masla nahi. Jitney bhi thy yehi thy jaise un ko chaiye sahulatein aur ziada toh hum un ko agar deinge facilities toh maaneinge aur ziadatar yehi masla hai k kuch karte nahi hain

RA: matlab karna kis tarah matlab kia who chah rahe hain hum un k liye kar sakte hain

CHW: woh chah rahe hain k woh aate hain na hamein medicine milti hai yahan pea ate hain toh na sahi response milta hai aur hum yahan par aate hain matlab woh apni us pe transport pe aate hain toh woh bhi hamein dur parta hai woht toh un ko gaari bhi chaiye toh yeh sab sahulatein hum deinge unhein toh kaafi hadh tak behtareen ajaayegi

RA: behtaree a jaayegi

CHW: jee

RA: toh aap chahti hain aisa kuch ho

CHW: je

RA: thek acha aap k khayal mein konsi cheezein community mein CHW k kirdar ko behtar bananey mein madad kar sakti hai jaise aap kehte ho na k community mein refuses k liye koi hal nikal aaye toh behtaree a sakti hai

CHW: saari cheezein yehin thi k jaise kaam un ko toh cheez chaiye hum agar woh humse cheez hi maangte hain k hamein yeh cheez chaiye k aap log yeh nahi de rahe ho hum yeh unhein deinge hum unhein bataeinge k bhae han kyun k hum abhi jaate hain toh who hamein kehte hain k case hoga yeh hoga hum kehte hain k nahi aisa kuch nahi hai hum aate hain poochte hain yeh yeh treatment hoga jaise pregnancy aur 5 saal tak k bachon ka batatey hain k yeh yeh hoga toh who kehte hain k nahi in k elawa bhi chaiye hamein aana bhi parta hai hum unhein ja k aur ziada counseling kareinge aur provide kareinge cheez k hum aap k liye yeh bhi laayein hain bhae aap ko gaari bhi a k legi yahan pe aap ko medicine bhi milegi toh is tareeke se is tarhan se CHW ka bhi behtar hojaayega

RA: k cheezein kis tarhan ki chah rahe hain kis tarhan ki cheezein honi chaiye

CHW: cheezein yeh honi chaiye jaise woh hospital mein jaate hain toh un ka kharcha lagta hai jaise doctor ko check karana hai toh hum yahan pe provide kar rahe hain leikin is k elawa yeh k medicine wagera bhi chaiye hoti hai kehte hain k hamein who bhi chaiye aur in k elawa un ko transport bhi chaiye k itna dur parta hai kaise aayein hum gaari bhi chaiye toh yeh cheezein provide un ko karni chaiye aur case ka sab se bara masla hai k hum kahan pe ja kar naam likhwaayein joh matlab achi jagha ho aur free mein bhi mile toh delivery ka bhi masla hai

RA: delivery ka bhi masla hai phir aap kia kehte ho

CHW: hum yeh kehte hain k hamein yahan pe delivery k liye hona chaiye sahulat patients k liye

RA: acha aur ziadatar aap ko joh lag raha hai joh masla woh isi wajha se bhi horaha hai

CHW: isi wajha se horaha hai ziadatar yehi keh kar bhaga deite hain na k aap log kuch karte nahi ho toh hum unhein kuch bata bhi nahi paate k sab joh aap keh rahe ho hum nahi de pa rahe toh hum unhein bata nahi paate

RA: jab k aap ne bola k hum toh treatment bhi de rahe hain is k elawa bhi

CHW: is k elawa bhi un ko aur ziada chaiye toh hum kehte hain aur ziada rakho ta k unhein bataein k bhae han aap ja sakte ho hum yeh sab bhi de rahe hain

RA: kitne gharon ki baat horahi hai ek ghar ki baat hai

CHW: ek ghar ki baat nahi hai kaafi gharon mein hota hai ziadatar matlab joh korangi side jis side pe hum jaate hain wahan pet oh ziadatar log aise hote hain jin ko matlab yeh cheezein ziada chaiye hoti hai un ko woh bharosa bhi nahi karte toh un ko mananey mein thora lagta hai toh un k liye cheez bohat kam hai unhein aur ziada chaiye waise toh har insaan yehi kehta hai k bas yehi karte ho phir chahe yahan ka ho ya out ka ho

RA: acha toh matlab yeh k unki cheezon ko poori kareinge matlab phir aap ko jaise k aap ne kaha than a k pehle hum deite thy gift deite thy rakam deite thy toh log khush thy toh ab jab nahi de rahe hain toh phir aap k sath

CHW: ab nahi de rahe hain toh un ko phir yeh hai manah karte hain k nahi bhae yeh sab pehle karte thy aura b nahi kar rahe ho ab koi hamein faida nahi hai aap hamein faida dou toh hum ko data wagera dein hamein bhi faida dou

RA: acha who is tarhan se kehte hain aap ko

CHW: je

RA: acha aap ki raaye mein aise jin gharon mein aap jaate ho hamal hain nauzaida joh newborn bache hain un k hawale se konsi maloomaat unhein deina chaiye

CHW: jaise un ki zaroorat k hadh tak jitni bhi cheezein un ko ja k hum maloomaat deinge k ab joh hai na hamare center pe yeh bhi horaha hai ultrasound bhi horaha hai aur yeh k aap ko sahulat mil rahi hai delivery ki bhi aap bolein toh hum jaise contact bhi deite hain toh aap gaari bhi bola sakte ho jab bhi aap ko zaroorat pare matlab jaise last month chal raha ho

RA: matlab aap ki raaye yeh hai k yeh honi chaiye

CHW: yeh honi chaiye un ko transport hamein deini chaiye

RA: aur is elawa

CHW: is k elawa yeh hai k jis tarhan medicine wagera un k liye honi chaiye aur jaise unhein test wagera karwaane hote hain toh woh bhi mehange hote hain na toh un ko bhi sahulat deini chaiye hum deite hain unhein proper tareeke se deite hain warna who kehte hain hamein nahi milta kuch

RA: acha proper tareeke se kaise deite ho

CHW: jaise yahan pe hum log hamla aurton ka treatment kar rahe hain thek hai leikin yahan pe aurtein aati hain treatment hota hai matlab ultrasound hogaya aur in k elawa aur bohat se test hote hain na joh woh kehti hain hamein karwaane hain toh abhi woh sab nahi hai jaise blood test wagera toh hamein woh cheezein bhi deini chaiye aur last tak hamein un ko deakhna chaiye matlab delivery tak toh hamein un ko proper treatment deina chaiye test wagera delivery bhi toh is tareeke se refuse bhi kam hoge na

RA: thek kia aap k paas aur raaye ya khayaalaat hain joh aap apne surveillance k kaam k baare mein batana chahti hain

CHW: filhaal toh jitney bhi thy sab bata diye

RA: surveillance ka joh kaam kar rahe ho us hawale se aap mujhe batana chaheingi aap k khayal mein k aisa bhi hona chaiye

CHW: kaam k hawale se

RA: koi bhi raaye ya khayaal joh aap ko lagta hai k haan apne surveillance ko aur behtar bananey k liye yeh cheezein honi chaiye

CHW: matlab yeh k hum gharon mein jaate hain na toh hamein kehti hain aurtein k hamein need hai is cheez ki yeh dawai jo un ki daily rotine use ki hoti hain toh hamare paas kuch medicine wagera bhi honi chaiye hum unhein ja k dein wahan par kuch medicine ya koi matlab aisi cheez joh pregnancy k doraan woh khaati ho toh pregnant women ko ja k who gharon pe hamein provide karni chaiye

RA: thek hai yeh toh gharon ki baat thi mein aap ki raaye janna chahongi k surveillance k baare mein aap kia chahti hain kia hona chaiye koi bhi raaye bata dein joh aap k zehan mein chal raha ho

CHW: ab jitney bhi sawal thy (laugh) kaam k andar na acha yeh hona chaiye k waise toh pehle hamare upar burdun ziada tha leikin ab kam hogaya hai Tab ki wajha se toh yeh bhi toh achi hi baat hai

RA: leikin burdun kis tarhan kam hua

CHW: pehle paper work hota than a toh us mein yeh hai k bohat saari cheezein le jao folder wagera hath mein ho pen wagera toh woh sab phir koi hawa wagera aaye toh kaagaz urte hain toh paper work mein mushkil hoti hai leikin abhi Tab pe kaam a gaye toh yeh easy aur yeh hai k forms bhi ab chote hain itna ziada kaam bhi nahi hai kam hogaya hai matlab kam hogaya hai easy hogaya hai

RA: sahi aur toh aisa kuch nahi

CHW: nahi bas

RA: bas ok shukriya

CHW: jazakallah..

Date: 26102020

RA: Acha NAME mujhe aap sab se pehle batayeingi k aap ki umar kitni hai

CHW: meri umar hai aa 36

RA: 36 thek hai. Acha or aap ki taleem kitni hai

CHW: master

RA : master kiya hua hai kis cheez mein master kiya hai

CHW: IR . International relation

RA: Aur aap ko kaam karte huye kitna arsa hua hai

CHW: mujhe kaam karte huye 2011 mein aayi thi matlab 9 saal hogaye

RA: Acha surveillance mein kaam karte huye kitna arsa hua hai

CHW: 2016 mein 15 mein aayi hun surveillance mein pehle matlab ANISA mein alag alag kaam hota tha PW ka newborn ka phir baad mein jab who project khatam hua toh phir hamein matlab surveillance NW mein dala gaya

RA: 2011 se aap kaam kar rahi hain waise aur surveillance mein 2015 se kaam kar rahi hain

CHW: Je

RA: Acha thek hai . Aa ab mein thora sa aap se aap k kaam k hawale se baat karongi toh mujhe yeh batayein k rozmara k surveillance ki activity karti hain household visit ka structure visit karti hain toh is kaam k hawale se aap kaisa mehsoos karti hain

CHW: Matlab aa acha mehsoos karte hain kyun k hamara ek matlab hum kisi ko apne taraf se asaani dein matlab un ka bhala kar rahe hain matlab un k ghar pe jaate hain PW capture karte hain matlab toh aa aa matlab un k ultrasound wagera k liye matlab koi kahin tak pohanch nahi pata kisi k who wasail nahi hoti kyun k hum hamare us se na acha kaam karein kyun k kisi ka bhala karein

RA: Hmm. Acha aap ne kaha aap kisi ka bhala karti hain toh yeh aap ko kaisa lagta hai kaise bhala karein

CHW: matlab aa matlab bohat se log aise hain joh matlab afford nahi kar sakte jaise ultrasound hota hai aur saare test hote hain bachon k jaise matlab hum check karte hain bachon ko agar fever ho kuch bhi ho toh who un k otoh ghar pe beithe se kisi ko kuch pata nahi hota toh who hum cheezein check kar k batate hain toh who ayein waqt pe kahin pe pohanch jaate hain matlab ek un ko asaani hoti hai hamari wajha se matlab

RA: aap ki wajha se un ko asaani ho jaati hai

CHW: Je

RA: Acha. Aur kia mehsoos karti hain apne kaam k hawale se ek toh aap ko yeh lagta hai k asaani kar deite hain aap log aap apne hawale se kaisa mehsoos karti hain

CHW: matlab hamein bhi awareness milti hai k alag alag project hote hain alag alag hamein training di jaati hai hare k matlab har field mein alag alag kaam hota hai jaidse pehle hum PW ka kaam karte thy toh hamein Anc ka bhi pata chal jata tha PW ko kia kia cheezein batani hoti hai toh who matlab anc mein kis tarhan ki care karni hai mother kit oh woh cheezein bhi hamein matlab ek hamari knowledge bhi barh jaati hai aur newborn ka jab hum us team mein jaate hain toh newborn ki care joh karte hain us k hawale se joh hamein joh bhi jaati hai anc toh who

dsaari cheezein matlab who hamare liye bhi bohat ziada faidemand hai ghar mein bhi hum matlab un ko sahi tareeke se kar sakte hain

RA: Aa sahi tareeke se kaise kia matlab

CHW: agar hamare ghar mein koi aisa matlab bacha ho ya kuch ho toh kuch aisi cheez ho fever ho kuch bhi toh bohat saari aisi cheezein hain hamein pehle nahi pata thi is kaam ki wajha se who saari cheezein khud hi ajaati hain kisi ko kuch hai toh haan matlab itni knowledge toh de sakte hain k aap k bache ko yeh hai yeh hai toh matlab yeh cheezein hamarein ko

RA: do cheezein hogayin aap ki ek toh aap ko lagta hai surveiilance ka kaam hai umm us ki wajha se aap logon k joh community hai us ka bhala kar sakein aur dusra yeh k aap ki apni knowledge bhi barh jaati hai aur is k elawa aur koi cheez hai joh apne household visit ya surveillance k hawale se aap kehna chahti hain k kaam k hawale se kaise lagta hai

CHW: aa matlab kaam k hawale se ek yeh bhi hai matlab aa

RA: kaisa mehsoos karti ho

CHW: matlab acha hai matlab ek hum ek matlab community ko hum ek achi cheez bana rahe hain matlab hum un ko awareness de rahe hain toh matlab who saari aisi aurtein hoti hain joh bohat saari cheezein pata nahi hoti toh hamari wajha se unhein bhi bohat saari matlab pata chal jaati hai

RA: Acha aap k kaam k sath awareness bhi de rahe hote ho

CHW: haan matlab agar koi cheez hamein pata hai aisi cheez hain matlab safai k hawale se ya kuch aisi cheezein toh hum matlab un aurton ko batate hain k aa paisa aisa karo aisa aisa matlab

RA: aur aur kia kia cheezein batati hain

CHW: Aa aur bohat saari cheezein jaise bachon k hawale se k koi bohat si aurtein hiti hain joh medicine wagera k hum se pooch rahi hoti hain k matlab ya toh ultrasoung wagera kuch aisa hota hai toh hamein matlab dikhati bhi hain k is mein kia hai kia hai toh woh cheezein bhi hamein matlab agar hamein jis hadh tak dsamjh mein aati hai toh hum unhein bata deite hain k hamein bhi deakh k batao aa matlab position ka batao aur kabhi kabhi aisa hota hai na k aurtein ultrasound dikhati hain k batao is mein kia likha hai matlab hamari position batao wagera bache ki k sahi hai k nahi toh who bhi hamein jitni hadh tak humein parhna aata hai toh hum parh k bata deite hain agar hamein kuch pata nahi hota toh hum unhein batate bhi hain k doctor se pooch k dusre din a k aap ko cheezein batayeinge joh matlab hamein samjh mein nahi aati matlab yeh k ek hadh tak bhi sahi hai k un ki cheezon se hamein bhi faida horaha hai k aisi cheezein joh hamein bhi nahi pata hoti hai ultrasound hamein likhe ya report mein toh matlab hamein bhi pata hai chalta hai phir doctor se a k poochte hain k un ko batate hain ya hamein pata hota hai toh batate hain

RA: Acha yeh koi aisi cheez hai k acha toh mehsoos karti hai koi aisi cheez joh apne kaam k hawale se nahi achi lagti ho

CHW: Nahi saari cheezein achi hi hain (laugh).

RA: Nahi sab kaam karne mein acha mehsoos horaha hota hai haan ghar ghar jaane mein

CHW: Dhoop mein thora dhoop pareshani hoti hai k agar matlab k ziada jis din dhoop hoti hai toh hum bolte hain k thore ghar kam hojaate hain toh toh matlab us wajha se thora woh hota hai

RA: Kam hojaate hain matlab

CHW: matlab kabhi kabhi aisa hota hai na k kaam ziada hota hai matlab kabhi aisa hojaata hai k itne week karne hai toh kabhi kabhi aisa hota hai k tabiat agar thori down hojaati hai toh bolte hain k thora kam kar lein matlab toh is wajha se pareshani hoti hai waise nahi toh limited hai sahi hai hamein jitna diya jata hai utna hum kar leite hain

RA: lekin acha yeh k itne target aap ko diya jaata hai k itna karna hai toh us ki wajha se aap keh rahe ho k thora sa

CHW: matlab nahi kar toh leite hain k kabhi kabhi aisa hota hai na k mausam bohat ziada garmi bhi bohat ziada matlab jaise nahi bhi horaha hota hai hmm toh matlab wapis aayein toh yeh bol deite hain bhaii gharmi ki wajha se hum ne itna nahi kiya hai aisa

RA: Hmm toh aap k kaam k hawale se k aap ko lagta hai ek toh aap joh aurtein hain bache hain un ka bhala kar rahe ho

CHW: Je

RA: thek hai unko cheezein provide kar rahe ho ya un k sath link bana rahe ho dusra yeh k aap ki apni knowledge bhi barh rahi hai

CHW: Je

RA: Thek hai

CHW: matlab hum unhein vaccine ka bhi batate hain jaise vaccine bohat si aurtein hoti hain un k card hote hain who parhi likhi nahi hoti na toh unehin nahi pata k kab vaccine lagni hai toh bohat si aurtein aisi hain k vaccine card dikhati hain toh hum unhein bata deite hain k falah din lagega falah din lagega toh yeh bhi matlab un k liye asaani hojaati hain hmm matlab yeh bhi hum un ko bata deite hain cheezein

RA: Acha thora sa mujhe yeh bataogi k joh aap ka surveillance ka jih kaam hai is mein aap log karte kia ho

CHW: hum matlab aa joh matlab married women who 13 saal se le k 29 saal tak ki jitni bhi shadiyan wali family hoti hain hum unhein enter karte hain or joh under 5 joh hote hain matlab bache unhein enter karte hain or agar hamein joh matlab jis married women ko enter karte hain toh who agar PW hai matlab pregnant hai toh hum usay enter karte hain phir aa un ka agar dubara jaate hain jaise visit toh us ka outcome aata hai who hum enter karte hain matlab newborn check karte hain hamari newborn ki team hai check karte hain shayad hum nahi karte.

RA: hmm. toh yeh kaam karte hain

CHW: Je

RA: acha aap ko lagta hai aap newborn or aap ghar ghar jaate ho

CHW: Je

RA: Thek hai or din mein aap log takreeban kitne household visit karte hain.

CHW: hum log matlab hamein jaise abhi hamara task tha k hamein 15 matlab conduct karne hi thy leikin aa hum matlab 15 conduct karte thy toh us k hisaab se jaate jaate hum matlab kabhi 30 ghar kabhi 40 ghar eke k banda kar leita tha kyun k log bhi milte thy 9 family bhi milte thy is ewajha se matlab hum 15 karte toh thy 15 se bhi barh jaate thy kabhi kabhi matlab hum aage kar bhi leite thy matlab 15 tak kabhi jaate jaate kabhi 40 ghar bhi kar leite ho kabhi 20 ghar bhi kar leite ho hmm matlab kar leite thy

RA: aur yeh asaani se hojaate thy

CHW: Je je asaani se hojaate thy

RA: koi mushkil toh nahi hoti

CHW: nahi nahi mushkil nahi hoti. bas kabhi mausam aisa ho toh phir (laughing)

RA: Acha kause mausam mein masla hota hai

CHW: garmiyon mein ziada jaise beech mein nahi itni bohat ziada garmi thi tab thora matlab ab toh thanda mausam hojaaye tab bhi kar leinge (laughing)

RA: Is se bhi ziada kar leinge

CHW: kyun k mausam k hidsaab se bhi banda kar leita hai

RA: toh aap log sirf ja k aa joh aap ki aa married women hoti hain un k baare mein data leite hain or joh under 5 bache hote hain data leite hain acha toh kia aap k khayal mein k yeh aap kaam kar rahe ho is k elawa bhi aap logon ko joh nauzaida bache hain jaise newborn hote hain or joh maayein hain un ko koi taleem ya information deine ki zaroorat hai

CHW: Je matlab bohat saare agar parhe likhe log hain toh un ko nahi hoti itni leikin matlab kuch aise log hain joh matlab aa jaise bachon ka safai ka khayal nahi rakh rahe hote hain toh pehle jaise ANISA mein kaam karte thy na toh hum anc deite thy aur joh matlab hum hi matlab newborm ki team hote thy or PW ki team hoti thi na PW ki team joh jaati thi na woh mother ko matlab shuro se 3 maheeney se le kar 9 maheeney tak anc diya karte thy matlab apne kaam k sath PW ka followup karte thy na ghar mein jaate thy toh matlab us se bohat faida mila tha matlab teeke lagao matlab shuro se le k folic acid joh hoti hai who zaroori hai farasulphate who zaroori hai toh woh saari cheezein anc mein thi toh hum unhein batate thy pehle matlab is tarhan hota tha or teeke lagao saatwein maheeney mein ta k matlab un ko beemari se bachata hain toh who saari cheezein hum un ko baar baar har baatr followup karne jaate thy na 9 month tak followup karte thy toh unhein har baar ja k batate thy toh who bhool bhi jaati thi dubara unhein bata deite thy un k zehan mein tha hmm matlab us se itna acha hua tha k matlab log tetanus k joh teeke hain matlab joh maayein lagati hain saatwein aathwein maheeney mein toh who hamari wajha se matlab who ziada un ko

RA: aap baar baar ja k un ko batate thy

CHW: Je who un ki team hoti thi who jab newborm paida hota tha toh hum 0 day mein capture karte thy toh hum 0 day mein hi ja k joh pehla dhood joh hota hai who zaroori hota hai maa ko pilana toh who saari information diya karte thy toh us se matlab bohat ziada faida hua

RA: acha yeh toh aap ANISA (CHW) Je (RA) project mein karte thy aur surveillance mein aisa kabhi kuch hua tha

CHW: survelleince mein hum toh ziada tar matlab hum toh batate thy aurtein jitni information poochti thi toh woh hum batate thy k matlab who poochti hain k hum teeka lagane kab jaayein k hum bache ko matlab k yeh hai toh hum matlab kaise us ki karein toh saari cheezein kyun k hamara farz hai community mein kaam kar rahe hain k hum aise idarey mein kaam kar rahe hain k joh cheezein unhon ne hamein bataya k hamara farz hai hamein na bola karein phir bhi hamara farz hai k un ko awareness dein ta k matlab maashire mein matlab achi cheez toh ho

RA: thek hai toh aap joh agar aap se koi poochta hai

CHW: haan who batati hain bhale hamein time lage us ghar mein hum woh cheezein unhein batate hain bohat si aisi aurtein hoti hain joh ultrasound wagera who bhi hum beith k bhale hum hamein jana hota hai aage lekin phir bhi bolte hain unhein batate hain jab hum dubara aayein toh un k talukaat ache ho baaki unhein ne hamein kuch bataya toh aage bhi un ka karein

RA: acha is se aap ko lagta hai k aap ka taluk joh hai woh acha hota hai

CHW: acha hota hai haan. kyun k hum unhein sirf hum salam kar k pooch k thek ho chale jaayeinge toh boleinge chale gaye toh un ka nahi hai na unse baat kareinge ache tareeke se kuch poocha hai toh us ka sahi tareeke se jawab deinge aage bhi woh hamein bithayeingi ghar toh is tarhan se

RA: toh aap k khayal se aap ko lagta hai k yeh saari malumaat joh farhaam karte ho joh poochti hain us ki aap ko munasib training haasil hai

CHW: je jitni hum abhi tak jitney bhi kaam kiye jin jin project mein matlab toh woh joh joh hamein malumaat hai jitni hai who de deite hain un ko

RA: acha. Toh joh abhi is mein waise toh nauzaida bachon k hawale se ya maaon k hawale se aap ko munasib training haasil hai

CHW: matlab thori bohat hai jitni hamein matlab

RA: waise training acha toh thori bohat training mein aap ko kia bataya gaya hai

CHW: matlab ek toh shuro mein toh jaise kisi k mutalliq jaise PW hai aap ko 3 maheeney mein matlab folic acid khaani hai phir jab 6 maheeney hojaayeinge toh aap ko parasulphagte khani chaiye joh kyun k khoon ki kami k liye hoti hai who aap ko kia kia matlab khizaayein joh hoti hai woh khaani chaiye saatwein aathwein maheeney mein ja k aap ko teeke lagwane chaiye aap ko zaroori hai agar aap matlab pehla hamal hai matlab aap ka zaroori hai k aap ja k naam likhwao hospital mein yeh nahi k daayon se ya kisi se bhi delivery karwao zaroori zaroori hai k aap kisi bhi hospital mein ja k delivery karao toh itni information hoti hai toh woh hum matlab un ko batate hain newborn k baare mein agar bacha paida hota hai joh pehla ma aka doodh hota hai woh aap bache ko zaroor pilao kyun k who bache ko bohat si beemari se bachata hai toh hum us k bhi baare mein batate hain jab paidaishi teeke joh hote hai woh aap zaroor lagwao matlab un k teekon k baare mein or us ki joh matlab umbilical hoti hai us ko matlab bohat se aise pata nahi kuch kar rahde hote hain shuro mein agar woh matlab blood a raha ho kuch toh us k

RA: kia karte hain log

CHW: matlab who jaise hamare yahan poorane tareeke hain toh matlab tale laga rahe hote hain aur pata nahi kia kia cheezein toh who to sahi nahi hota

RA: in cheezon ka bata sakti hain ek toh tale lagate hain aur kia karte hain

CHW: aaa koi namak bhi lagate hain hmmm aur pata nahi kuch aur bhi bohat saari cheezein totkey hote hain woh cheezein kar rahe hote hain toh usay bhi hum un ko batate hain pehel mili thi training agar aap us pe lagane k liye joh matlab medical se joh cheezein milti hain woh aap lagao ziada us ko saaf suthra rakho matlab toh yeh saari cheezein jitni hamein pata ho bata diya

RA: aur yeh training aap ko kitne kitne arsey baad milti hai

CHW: yeh abhi toh itni nahi milti pehle jab hum matlab jaise NW sath mein thy na toh un ko matlab tab yeh surveillance ka hota tha k matlab married woman joh hain enter karni hai death matlab death joh hamein milti hai matlab who enter karni hoti hai aur newborn outcome PW yeh saari cheezein surveillance mein hai matlab PW aur newborn team mein thy tab yeh training hamein baar baar di jaati thi

RA: matlab kitne arsey pe

CHW: aa matlab kitne arsey aa har hmm hare k circle jaise matlab hogaya matlab 5 6 maheeney hogaye toh dubara se matlab woh revise karwaate thy matlab joh joh cheezein aap bhool gaye ho is tarhan who karte thy.

RA: aur circle kitne arsey ka hota tha

CHW: pehle hamare jab hum NW k sath hote thy toh hamara circle 3 maheeney ka hota tha matlab 3 maheeney mein circle hamara jab folder pe kaam kiya karte thy toh 3 maheeney mein circle khatam hojaate thy

RA: Hmmm acha 3 maheeney lagte thy aur 3 maheeney k baad phir aap ko training di jaati thi

CHW: haan Je dobara

RA: aur us training mein main cheezein kia kia hoti thi

CHW: matlab jab hum abhi jab hum NWs mein aaye matlab tab bhi hamein hamari hoti thi joh hamare supervisor matlab joh hote thy who hamein batate thy k aap ko itne din k andar death capture karne hain newborn k karne hain toh us k hisaab se who saari cheezein bataya karte thy.

RA: acha aur is k elawa sirf death k baare mein batate thy newborn k baare mein batate thy aur un ko batana kia hai is k upar bhi koi training hoti thi jaise aap log jaate ho joh aap ne kaha na hamein ultrasound ki cheez dikha deaiti hain

CHW: is tarhan ka kabhi nahi hua

RA: toh yeh aap log khud se batate thy

CHW: khud se batate thy matlab yeh cheezein itni jitney bhi kaam kiya hai us meinyeh cheezein matlab hamare yahan nahi thi yahan hum khud se batate hain hmmm matlab ultrasound deakthte toh hamein jitna parhna aata hai toh ab tak hum ne jitna kaam kiya hai toh poochi hain toh hum bata deite hain

RA: toh is ka matlab is k upar aap ko

CHW: hona chaiye agar mujhe pata hai toh un k otoh pata nahi hoga na k ultrasound mein kia kia position hai k kahan likhi hoti hai kia likha hai kitne weeks likhe hote hain matlab thori bohat matlab complication hai kuch bhi hai kahan likha hai kisi kisi ko pata hota hai na matlab hum har ghar mein jaate hain toh bohat si aurtein hum se poochti hain matlab yeh zaroori hai har kisi ko har cheez ka pata hona chaiye matlab yeh aap ko pata ho ta k aap kisi ko bata sako agar koi hum se poochta hai usay thori bohat kuch bata toh sake

RA: ek toh ultrasound k baare mein aap se poochti hain aur kia kia cheezon k baarein mein aurtein poochti hain

CHW: ziada tar toh pregnant women hi apne masail bata rahi hoti hain hamein BP k baare mein pehle hum blood pressure wagera bhi check kiya karte thy AMNI ka ANISA se related tha hum BP ka bhi batate thy baar baar ja k check kar k bloodpressure aap ka sahi hai ya sahi nahi hai toh agar low hai toh aap ko matlab thora bohat yeh karna chaiye woh karna chaiye toh is wajha se matlab woh saari cheezein hum se poochti hain abhi matlab hum check toh nahi karte us mein nahi hai magar pehle jab hum karte thy joh check karte thy

RA: leikin aap abhi bhi jaate hain toh is tarhan ki koi cheezein aap se poochti hain

CHW: je je poochti hain k hamein yeh horaha hai matlab un ko hum yeh bolte hain k hum doctor nahi hai thek hai hum aap ko yeh nahi bol sakte k aap matlab yeh khao woh khao aap jao medicine ki jagha pe aap pehle ja k check karao k pata toh nahi hota low hai matlab high hai na pehle check karao phir aap ko doctor joh medicine suggest karta hai PW ho na woh deakh k khao kisi se pooch k bhi aap yeh cheez kiya karo toh yeh saari cheezein bata deite hain

RA: toh in cheezon pe aap ko lagta hai k matlab aap ko itna pata ho k aap community mein bhi bata sakein

CHW: haan hona chaiye aisa matlab har thori bohat training honi chaiye sab ko matlab hum PW k paas jarahe hain na ta k hum unse agar woh poochte hain na hamari itni knowledge ho k hum unhein bata sakein

RA: acha ek toh is k hamal k doraan joh bhi information deini hoti hai us k bare mein knowledge honi chaiye aur bachon k hawale se kia kia cheezein aurtein aap se poochti hain

CHW: bachon k baarein mein ziada tar teekon k baare mein poochti hain

RA: sahi

CHW: k yeh teeke kyun lag rahe hain kabhi kabhi yeh bhi poochti hain k agar yeh teeka miss hogaya hai matlab dobara lag jaayega hamein pata hai jaise hamein pehla teeka joh hai who ek saal tak lag sakta hai thek hai aur baaki joh teeke hain who baad mein bhi lag sakte hain leikin joh pehla teeka hai who ek saal tak lagta hai us k baad nahi lagta toh who saari information hamein pata hai toh babate hain agar teekon ka matlab kisi kis ko nab hi pata ho k dhaai maheeney mein lagte hain saare 3 maheeney mein lagte hain toh who saari cheezein hum se poochti hain joh jis ko pata hita hai who bata deite hain

RA: acha teekon k hawale se poochti hain aur kisi cheez k hawale se poochti hain bachon ki

CHW: aur aur agar matlab fever ho ya umblical infection hota hai toh matlab koi koi hote hain dikha deite hain k bache ko yeh yeh cheezein hain toh hamein kia karna chaiye hum suggest kar deite hain k aap doctor k paas jao is tarhan

RA: acha aur aap kehti hain k doctor k paas jao toh who chali jaati hain

CHW: nahi kuch kuch jaati hongi matlab kuch kuch toh apne ghar mein hi joh sahi lagta hai who kar leiti hain leikin parhe likhe matlab joh samjhte hain log who chale jaate hain (slight laugh) ab toh aise hote hain toh nahi

RA: acha toh mein yeh poochna chah rahi thi k aap ko kia lagta hai k as a sahulat kar thek hai na nauzaida maaon k darmiyan aap kia kirdar ada karti hain jaise ap facilitate karti hain na aap community se service k center par ajao toh us k hawale se aap kehna chaheingi k kia kirdar ada karti hain aap

CHW: matlab hum ek matlab who log hamein doctor keh rahe hote hain doctor a gai doctor a gai (laugh) hum unhein kehte hain k hum doctor nahi hain hum matlab ek hum matlab ke razatarana taur par matlab yeh kirdar ada kar rahe hain toh who yehi cheezein

RA: acha matlab aap ko kia lagta hai k jaise aap ne bataya k aap pehel aap ne baat kit hi na k bhala karti hain who ultrasound dikhaati hain ya doctor k paas jaane kuch bhi blood pressure ka poochti hain kuch bhi aap joh hain us ko matlab batate ho k doctor k paas chale jao us k hawale se mein thora jaanna chahti hun k aap ko kia lagta hai yeh k kirda kaise behtar kar sakti ho us mein aap kia kia karti hain us hawale se

CHW: acha matlab hamein bohat si aisi cheezein joh nahi pata thi matlab aisi training joh baar baar honi chaiye jis mein hamein matlab PW ya newborn k matlab kuch aisi cheezein joh hamein nahi pata thi joh doctor ko pata hoti hai hamein nahi pata toh matlab har baar batani chaiye matlab agar hum kisi ghar mein jaayein toh hum se pooche toh hamein knowledge ho toh hum unhein bata sakein joh hamein nahi pata hai who cheezein

RA: hmm thek hai yeh toh baat hogayi aap ko istarhan ki training ho agar aap se aurat kuch pooche toh joh bhi aisi cheezein joh aap foran se bata sakte ho who aap unhein bata sako thek hai is k elawa koi aisi cheez joh who bhi aap ko pata hona chaiye joh aap udhar halt oh nahi kar sakte toh matlab agar center pe aap bheij deite ho toh us k hawale se thora poochna chah rahi thi

CHW: je pehle tha k matlab gaari hoti thi

RA: thek hai

CHW: matlab hum log newborn matlab agar koi matlab sick hota hai kuch hota hai toh hamari team hoti thi joh usay le jaati thi le k aati thi aaj kal aurtein yeh ziadatar masla bolti hain k pehle toh aap log gaari deite thy matlab hum khud chale jaate thy ab aap log bolte ho k chale jao hum ne kaha pehle hamara project matlab aesa tha k gaari di jaati thi ab matlab yeh sahulatin hai matlab aap khud uasay apne faide k liye wahan ja sakte ho matlab agar dobara se aesa hoga toh achi baat hai

RA: acha matlab aap un k liye sahulat yeh farham karti thi k un k liye gaari de deity thi

CHW: je. Pehle aisa hota tha koi matlab bacha sick hota that oh us k liye matlab alag si team hoti thi us ko le k jaate thy aur center pe le aate thy

RA: acha aura b aisa nahi horaha

CHW: nahi

RA: acha is k elawa koi aisi cheez joh sahulat aap ko lagta ho joh community mein de rahi hain ek toh gaari thek hai is k elawa joh center aur community k beech mein aap ek kirdar ada kar rahi ho who kia hai

CHW: who matlab kuch aisa hai na joh abhi ek project tha aman ka thek hai toh aman k project mein ziada who nahi matlab kuch medicine aisi hoti thi joh matlab who unhein de deite thy who matlab who bolti thi k beemar hai ya kuch matlab aisi hain matlab hum bhi medicine de deite thy toh matlab log un ko ziada karte thy k hum jaate thy na toh kehte thy k aap aman se aaye ho hum ne kaha nahi aga khan se aman se aati thi matlab yeh medicine de k jaati thi

RA: aman foundation ki baat kar rahi hain

CHW: je toh matlab yeh cheezein de k jaati thi matlab kuch deine se bhi matlab aurton ka who hota hai k who aati hai kuch matlab deine

RA: aap ne kuch diya hai

CHW: hum log jab PW k followup karte thy toh us mein folic acid deite thy aur paracitric deite thy bas aur juch nahi deite

RA: aur is ki wajha se aap ko lagta tha k center joh aurtein aati thi center pe

CHW: han thora bohat tha aur ziada tar pehle joh tha blood pressure check karne k liye matlab hamare paas machine hoti thi check karne jaate thy toh us se bhi aurtein ziada khush hoti thi k ghar mein aati hain larkiyan BP check karne aati thi toh matlab yeh cheezein matlab kuch un ko mil raha hota hai na toh who khush horahi hoti hain matlab agar pooch k sirf chale jao aate hain kia karne aate ho pooh k chale jaate ho (laughing)

RA: toh abhi aap ko lagta hai k agar is tarhan ki koi cheez di jaaye blood pressure check karna dawaiyan toh us se koi fark parega

CHW: je us se bohat fark parega

RA: kia fark parega

CHW: matlab un k taaqat hum se ache honge who humse aaj kal bhaagte hain na toh matlab aaj kal aurat kehti hain aate ho aur pooch k chale jaate ho kuch deite toh nahi ho (laughing) is tarhan thora who hota hai k hum kehte hain k hamare paas bhi kuch aisa ho k hum ne kuch diya hai k dobara jaayein aap log yeh de k gaye thy cheezein toh istarhan karte

RA: surveillance k kaam se aap ko nahi lagta k koi cheez matlab center ya services deine mein madad de sakein

CHW: who toh hum kar rahe hain jaise hum ja rahe hain hamari wajha se hi PW matlab enrolled rorahi hain vital matlab PW ka ultrasound wagera horahe hain yeh bohat acha kiya hai is ki wajha se bohat acha jaise yeh log abhi karwa rahe hain na 18 haftey mein joh mil rahi hain toh is wajha se matlab nab hi batati ho agar pregnancy chupati hon toh is ki wajha se matlab bata deity hain han hamein agar faida hoga toh cheezein bata deite

RA: thora sa khul k bataogi k kaise bata deity hain kaise pata chal jata hai

CHW: matlab kuch kuch aisi aurtein hoti hain matlab kuch faida nahi horaha k aati hain pooch k chali jaati hain agar pregnant bhi hongi nahi nahi nahi hai nahi hai bas matlab chale gaye thy

matlab un ko matlab dusri jagha se bhi pata chala hai k yahan pe ultrasound bhi horahe hain k matlab yeh log delivery bhi karwa rahe hain asaani se gaari bhi hai matlab saari cheezein matlab ab hua hai na vital ki wajha se toh us ki wajha se matlab aurtein han sahi hai haan matlab agar shuro k month mein hamein nahi batati thi toh who kehte hain na shuro k month mein sahi nahi hota batane ka toh matlab un ko pata hai shuru k maheeno k matlab un ko pata bhi nahi hai phir phir bhi bol deity hain k han han hamal se hai hamara test kar k deakho phir naam likhwa leite hain

RA: do cheezein ek toh aap ne kaha k shuro k maheeney mein batati hain

CHW: haan

RA: yeh kia concept hai

CHW: matlab kuch kuch aurtein hoti hain k shuro mein matlab nahi batana chaiye shuro mein matlab pregnancy ko chupana chaiye agar bata deite hain matlab un k mind mein hota hai k kuch na kuch hojata hai matlab shuro mein nahi batana chaiye k leikin abhi matlab yeh hai k PW hoti hain toh un ko pata hai k shuro k maheeney mein jaayeinge toh naam likha jaayega toh phit matlab who bata deity hain han bata dein ta k hamara naam bhi likha jaaye toh is wajha se asaani horahi hain

RA: aur ek aur aap ne kia cheez horahi thi joh dusre logo se sunti hain

CHW: han sunti hain matlab agar ek paros mein PW enrolled huyi hai k han hamare paas falah ek team aayi thi mujhe khaane k liye bhi kuch cheez diya mujhe delivery k waqt gaari mein le k jaayeinge yeh saari cheezein matlab un ko pata hoti hai k han in ko sahulat mili hai toh hum bhi chaheinge toh is tarhan matlab kuch bhi PW aati hain naam likhwane k liye joh hum ne bhi capture nahi ki who bhi khud chal k a rahi hain

RA: toh yeh aap ko lagta hai k aap ki ajha se horaha hai

CHW: han je hamari wajha se matlab hum community mein jaate hain hum hi bata rahe hain na k yeh yeh cheezein hamare yahan pe horahi hai hamari wajha se aage un ko faida horaha hai a rahe hain matlab

RA: hmm acha nasreen mein aap se poochungi k aisi konsi cheezein hain joh sunte ho aap k kaam k hawale se joh aap ko acha lagta hai k aap ki hausla afzai hoti hai aur aap kehte ho k haan hamein yeh kaam aur ache se karna chaiye

CHW: matlab log bolte hain kahin se hum sunte hain han matlab aga khan wali aisi aayin thi worker unhon ne matlab hamein yeh karwa k diya who karwa k diya matlab dusron se tareef matlab kisi aur ghar pe gaye achi tareeke se matlab bohat si aisi aurtein hoti hain phir bohat saari izzat bh karti hain han han hamein unhon ne yeh kiya jahan pe pehle joh newborn hote thy matlab joh unhon ne matlab operation wagera bhi karwaye huye hain toh who log bhi bohat saari izzat deite hain unhon ne hamare liye bohat kuch kiya hua hai hum un k ghar pe jaate hain izzat se bithate hain matlab aise

RA: acha who acha kia bolti hain aap k baare mein aap ko acha lagta hai

CHW: matlab who dusri un k paas koi beithi hoti hain aurat matlab jaise hum gaye toh dusri aayeingi k haan yeh yeh cheezein karti hain yeh bohat achi hain matlab who hamein unhon ne hamara naam likhwaya tha bache ka iaj karwaya tha yeh karwaya that oh matlab dusri aurton

se matlab bata rahi hoti hai agar un k paas koi aati hai beithti hain batate hain toh aisa bhi hota hai

RA: is k elawa aur koi cheez hai koi aisa waqiya joh aap batana chaheingi joh acha laga ho apne kaam k hawale se

CHW: aur matlab hamare supervisor hamari tareek wagera kar deite hain toh who cheez bhi hamare liye achi hoti hai

RA: hmm acha yani supervisor tareef karti hain

CHW: bata rahi hoti hain k aap acha kaam kar rahe ho upar se matlab horaha aap ne acha kaam kiya hai toh who cheez hamare liye achi

RA: aur aisi konsi cheezein jis ki wajha se joh rukawat banti hai aap k kaam mein mushkilaat jab kaam kar rahe hote ho toh mushkil ka saamna karna parta hai ya kaam mein dushwari hoti hai

CHW: kaam karne mein dushwari ziada tar gaariyon ki bhi wajha se hoti hai k kabhi matlab driver hote hain k matlab kabhi jab bhi jana hota hai ya kabhi gaari nahi aayi hai toh matlab us ki wajha se waise kaam mein koi rukawat nahi hoti

RA: acha gaari se kia matlab

CHW: matlab kabhi kabhi aisa hojaata hai k driver hote hain na k hum yahan nahi jaayeinge wahan nahi jaayeinge kahin pohanch nahi paate matlab aisa hota ha ya kabhi driver matlab kehte hain deir se aate hain ya hamein jaldi jana hai k matlab hamein kaam karna hai k matlab who hota hai

RA: driver ko jaldi jana hota hai k wapis aur aap ko kaam karna hota hai

CHW: han kaam karna hota hai k thora who hota hai

RA: acha community mein toh chalo ek issue driver wala hai community mein ja k kia mushkilaat hoti hai

CHW: aa itni matlab thori bohat hoti hai

RA: kia hoti hai thori bohat

CHW: matlab yeh hai k who jaise kehte hain kuch deite toh nahi ho sirf pooch k chale jaate ho k matlab hamari wajha se bhi matlab kuch aisa mile matlab koi cheez jab dobara jaayein toh hamein bole k aao han han beitho darwaza khol k daakhil karte yeh cheez thori ho

RA: koi asi cheez joh jaise abhi aap ne kaha k refusal bhi hote hain

CHW: han refusal hote hain

RA: toh who kia kehte hain aap se

CHW: who kuch kuch toh ziada tar refusal itne nahi hai matlab joh matlab hote hain joh kehte hain hum afford kar sakte hain phir hum kyun aap ko bataein aur aaj kuch kuch halaat bhi aise hain aap ko pata hoga daketi wagera kuch log is wajha se bhi matlab refuse karte hain k andar pata nahi kon hai hamare paas card hai matlab aaj kal halaat aise hai na k card dekh k bhi koi yaqeen nahi karta k pata nahi kisi ka le k aaye ho matlab ziada tar who log refuse karte hain jin

logon ko dar laga hota hai k andar ajaayeinge kia kareinge toh who refusal hote hain ya toh jin ka panel hota hai ziada tar who refuse karte hain hamara falah jagha penel hai toh hum log aap se kaise baat karein yeh karein who karein toh aisa hai

RA: acha ek toh gaariton ka masla hai

CHW: je

RA: k driver ka masla hai joh aap k kaam mein dushwari paida karta hai rukawat paida karta hai aa dusra aap ne baat batai k koi cheez deite nahi hain

CHW: han

RA: community ko toh is wajha se bhi ke kehti hain aurtein k rozana a jaati hain

CHW: (laughing) is wajha se bhi

RA: aur kuch log aise hain joh afford kar sakte hain dusri jagha se is wajha se bhi aap se baat nahi karte thora say eh security ki wajha se bhi hota hai is k elawa koi aur cheez community mein chale gaye phir mushkil ka saamna karna para hai koi community k mahaul ki wajha se ya kisi aur cheez ki wajha se

CHW: je aisa bhi hota hai k matlab aurtein toh sahi hoti hain leikin un k joh shohar hote hain k matlab kuch aur hote hain toh matlab who un ki wajha se thora hota hai k aurtein maang rahi hoti hain matlab lekin un k shohar manah kar rahe hote hain k hamein kuch nahi karwana yeh who toh matlab un ki wajha se bhi thora aesa hota hai hum matlab ladies hoti hain toh aadmiyon se itni baat nahi kar paati matlab aurton se toh kar leite hain matlab admi ho toh un se alag baat nahi kar sakte matlab yeh hota hai k matlab unse koi aur baat kar le matlab aise toh

RA: aaa matlab aadmiyon se baat karne mein dushwari hoti hai

CHW: je je

RA: who kia kehte hain aadmi kia kehte hain

CHW>: koi koi apni un k mind mein hota hai k nahi le k jao falah se suna hai k aisa karte hain

RA: kia kia suna hai

CHW: who aisa hota hai na k blood ki wajha se bohat se log refuse karte hote hain jaise kisi kisi project mein nahi bache ka blood wagera sample wagera leite hain toh is wajha se kuch log refuse karte hain pehle jin ka le chuke nahi nahi nahi le jana khoon leite hain yeh karte hain who karte hain toh is wajha se bhi refusal hote hain

RA: aap k sath kabhi kuch aisa hua k aap ko kiai koi waqiya aisa hua ho

CHW: aisa toh koi waqiya nahi hua refusal thora bohat aise k hamara penel hai toh matlab nahi karwate kuch aise bhi hote hain matlab pehle koi enrolled tha toh un ki wajha se aisa kuch ho chuka hoga matlab misbehave matlab aisa hota bhi hai na kabhi kabhi

RA: toh misbehave hua hai pehle

CHW: jaise aayin ho kisi ne koi acha response na diya ho center pe jaise receiption pe ya doctor nahi matlab kuch bhi aisa matlab us ki wajha se bhi refusal hote hain

RA: us ki wajha se aap ko rukawat hoti hai

CHW: Han k hum aap k center mein jaate hain kuch nahi kiya falah ne hamein kuch nahi deakh na yeh kiya na who tum log yahan pe ajaate ho yeh karne who karne (laugh) toh aise hota hai

RA: thek hai toh acha ab mujhe batao k ab is ko behtar bananey k liye aap k kirdar ko behtar bananey k liye k aap k khayal mein hum kia kar sakte hain

CHW: matlab hum matlab jaise who cheez hum bhi un k liye kuch karein kuch bhi koi achi cheezein yeh aur hamari bhi

RA: achi cheezein kia

CHW: matlab medicine wagera ta k hum bhi kuch un ko dein k who hum se maange aur matlab hamare yahan ka bhi receiption hai ya doctor hai ya aise andar ka bhi mahaul ho acha ho k patient aayein toh un se ache se baat karein k matlab un ko acha response dein ta k agar hum dobara un k ghar jaayein toh who bhi hamare sath ache ho matlab kisi aur ki wajha se hum se badghumaan na ho aise matlab yahan k mahaul ka center ka bhi acha hona chaiye

RA: hmm do cheezein ek toh aap ne kaha k un ki imdad karo koi cheez dein thek hai dusri cheez joh aap aurton ko refer karo apne center pe center pe un ko achi tarhan samjh paaye ta k aap ki joh kirdar hai joh aap ka kaam hai who asaani se ho

CHW: asaani se ho

RA: thek hai acha is k elawa aap k khayal mein jaise joh maayein hain nauzaida bache hain pregnant aurtein hoti hain hamal se toh un k aap ko aisi konsi maalumaat hai joh aap ko un ko deini chaiye apne kaam k sath

CHW: matlab hamein aaa yeh safai k baare mein batana chaiye un k diet k hawale se matlab giza k hawale se matlab konsi giza khaani chaiye aur bachon k care k hawale se saari cheezein matlab yeh saari cheezein batani chaiye

RA: thek hai apne kaam k sath sath diet ko bhi batana chaiye aur safai k baare mein batana chaiye aur bache k hawale se aap ne kia bola tha

CHW: bache ki care kaise karni chaiye matlab un k teekon k hawale se k un ko matlab agar fever ho toh kuch bhi ho toh un ko kahin le k jaaye aur mtlab teeke joh lagwate hain who bhi aap ko zaroor lagwane chaiye falah falah beemariyon se bachaate hain who bhi sab ko pata hoga k han teeka falah beemari se bachata hai toh who us ko bata sake han is ko pata hai k falah teeka joh lagta hai falah beemari se bachata hai ta k who us ko bataayegi k han us wajha se matlab saari information honi chaiye

RA: haan jaise aap kaam kar rahe hote ho target bhi diya jaata hai k itne structure karne hain itne household visit karne hain

CHW: je

RA: toh us k sath aap yeh information kis tarhan asaani se de sakti hai

CHW: matlab yeh le hum aa hamare target thore kam ho matlab yeh ho k aap apne kaam ko acha karo matlab ache tareeke se karo phir jab aap yeh nahi hai k matlab thora sa kiya ache se jitna matlab kar sakte ho utna karo lekin us k liye team alag bhi ho

RA: aa joh maloomaat who team alag ho

CHW: han alag bhi ho

RA: thek hai aur aap alag ho

CHW: je

RA: isi kaam k sath aap aa kuch thora bohat maaloomat de who bhi behtar tareeke se de sako

CHW: haan behtar tareeke se hamare paas matlab yeh ho k aap apne kaam ko jitna acha kar sakte ho utna karo phir aap se jitna ho sakta hai utna karo (laughing)

RA: acha nasreen is k elawa koi aisi cheez joh kehna chahti hon batana chahti hon apne kaam k hawale se aur hum ne nahi poochi ho toh aap bata sakti hain

CHW: aur toh kuch nahi

RA: koi aisi cheez koi aap ko lag raha ho kaam k hawale se ho community ko behtar bananey k hawale se ho joh aap k demag mein ho joh aap kehna chahti hon

CHW: bas (laughing)

RA: thek hai thank you so much ..

Date: 26102020 Recording: 34:01

RA: Acha NAMEaap ki umar kitni hai

CHW: 37 years

RA: aur aap ki taleem

CHW: matric

RA: acha matric kiya hai

CHW: je

RA: acha aap k kaam ka joh tajurba hai who kitne saalon ka hai

CHW: 9 saal

RA: 9 saal

CHW: Je 2011 se

RA: yeh surveillance ko mila kar ya alag

CHW: nahi surveillance ko mila k us mein bohat saare project thy matlab ANISA k project mein hi hire huye thy

RA: acha. Toh joh aap k kaam ka joh tajurba tha aap ne kaha 9 saal

CHW: han

RA: surveillance mein aap ko kitne saal huye

CHW: surveillance mein us k baad matlab reh toh surveillance mein hi thy

RA: hmm hmm

CHW: ANISA mein DSS ki team thi toh us mein hum log DSS hi karte thy ghar ka survey k baad hum ne PW mein kaam kiya newborns mein kaam kiya tha jitney bhi project aaye ANISA k baad MANISA aaya AMNI aaya toh us mein joh followup thy usi followup mein rahe us k baad phir matlab tab ka joh surveillance shuro hua NWSR ka toh mein ne who kiya VR mein kaam kar rahe hain vital registry mein toh matlab itna ho chuka hai

RA: kitne saal huye surveillance ko mila kar

CHW: 9 saal

RA: 9 saal hogaye hain

CHW: je

RA: thek hai acha acha mujhe bataiyega aap k joh rozmara routine mein joh aap ka kaam chal raha hai community base mein aap kaam kar rahe hain ja rahe hain aap community mein toh aap kaisa mehsoos kar rahi hain

CHW: aa matlab hum toh who kar rahe thy na hamesha who kar rahe hain kaam abhi joh tab aaya tha us mein hum dar rahe thy k kaise sahi kar paayeinge ya nahi kar paayeinge toh mashaallah se hamare trainer bhi ache thy bohat acha unhon ne hamein trained kiya tab mein kaam karna sikhaya bohat ache se

RA: acha dar kis cheez ka

CHW: dar us cheez ka tha matlab nayi cheez thi us ko chalane mein dar gaye thy tha matlab ache se guide kiya gaya us k baad koi masla nahi raha sahi chalraha hai

RA: bilkul sahi chal raha hai

CHW: je

RA: acha yeh toh aap rozmara ki routine bata rahi ho aur koi joh aap ko lag raha ho problems a rahi hain ya aisa kuch

CHW: nahi nahi

RA: surveillance se related

CHW: nahi nahi

RA: surveillance mein aap kaisa mehsoos kar rahi hain

CHW: behtar hi mehsoos kar rahi hun jaise matlab who kaam tha us mein joh seekha hum ne toh waisa yeh bhi hai is mein bhi toh wohi kaam kar rahe hai na pregnant thy un k newborns thy or child hain under 5 years un ko follow kar k hamein aage barhna hota hai information leini hoti hai matlab agar who pregnant wagera hai toh un k pregnancy k hawale se matlab hamein un ko add karna hai tab mein toh matlab sahi hai ek had tak hum ne aku mein bohat kuch seekha hai

RA: acha aap ne kaha k surveillance mein mother ko kis tarhan information deite ho

CHW: information toh matlab is tarhan se un ko guide kar deite thy k is tarhan se center pe ultrasound ki facilities hoti hai jaise pehle yahan pe matlab blood test wagera urine test wagera bhi hote thy toh awareness kafi un ko bhi mil jaati thi center a k pehle itni nahi thi k pehle matlab dehaat thy pehle un ko awareness nahi thi toh hamein matlab acha laga counseling karna un ko guide karna pregnancy k doraan aur pregnancy k baad bhi un ko newborn follow karte rahe un ko guide karte rahe newborn k baare mein aur refer karne mein masla hota tha

RA: acha to refer matlab kis tarhan se karte thy

CHW: matlab AMNI mein pregnant women k matlab karte thy na follow phir yahan dr farzana thi un ko joh doctors hain who check karti thi toh matlab sahi tha

RA: aap ko lagta hai bilkul sahi tha

CHW: bohat sahi tha. Kafi log aaye kafi logon ne refuse bhi kiya

RA: acha refuse kyun karte thy aap ko kia lagta hai k matlab aap par aitemaad nahi tha

CHW: is tarhan ki koi baat nahi un ka yeh kehna tha k private karwa rahe hain toh hamein need nahi hai na aap k center mein kyun k kuch log aa

RA: han kuch log kia

CHW: matlab kahin pe joh karte thy panel ka that oh is wajha se refuse kar deite thy k hum apne panel k zariye apna elaj kar rahe hote hain toh matlab hamara kaam tha information deina k aap log a sakte ho health k hawale se matlab kaam horaha hai toh aap log yahan se faida utha sakte hain

RA: aap ko sahi se jawab de rahe thy

CHW: je matlab sahi bhi milta tha phir refuse bhi kar deite thy aur yeh chalta raha

RA: refuse ka joh reason hota tha who kia wajha kia thi refuse kar rahe hain

CHW: wajha toh yeh horahi thi k matlab who nahi aana chah rahe thy yahan center pe na k who kehte thy k yahan pe treatment sahi nahi chalta log darte hain treatment nahi hota toh is liye hum bahir se hi karwa leite hain toh phir matlab un ki marzi hoti thi

RA: acha aur aa aap ne kaha refuses k hawale se k yeh cheezein huyi hain hamare sath panel hai khud kar leinge aane ki zaroorat nahi hai acha is k liya matlab aap ko lagta hai is tarhan se jaise chahte hain k haan aur thora sa hum behtar karein un ko manayein hum is tareeke se kuch kiya

CHW: je chahte hain matlab refusal hote hain joh toh pehle CHWs jaate hain un k ghar visit karte hain kuch nahi nahi maante toh hum log un ko refuse mein le jaate hain kyun k bilkul bhi agree nahi hote hai na yahan aane k liye ya koi bhi hamein

RA: acha agree karne k liye aap kia karte ho basically community mein jaate ho

CHW: toh awareness deina

RA: acha kaise awareness deite ho

CHW: awareness yeh deite hain matlab ghar mein koi hamal se hai toh matlab hamal k doraan yahan yeh facility mil rahi hai pregnant k matlab ultrasound wagera bhi free mein horahe hain joh abhi vital Pakistan ki taraf se joh project aaya us mein delivery bhi karwa rahe hain free of cost koi charges nahi hai toh matlab un ko leina chorna registration hospital mein karwana sab unhi k through ho rahe hain toh matlab a sakte ho yahan se faida utha sakte ho

RA: acha toh aa yeh hai k yeh jitni bhi un ko maloomaat de rahe hain aap ko lagta hai k yeh unhein pehle se maloom tha un cheezon ka han hum center pe aana chaheinge yahan se hamein sahulat milegi ya aap k through matlab pata chala

CHW: hamare through hi pata chala kyun k hum log joh VR kar rahe hain VR mein jitni bhi pregnant mil rahi hain toh who kar rahe hain

RA: acha aur is k elawa joh laga k aur hamein kuch behtar karne k liye unse aur mazeed aap ne baat ki ho ya aisa kuch ya maan gayin hon refuses kabhi aisa bhi hota hai k han refuses nahi bhi aap ne manane ki koshish ki aur is k elawa aur bhi cheezein bataein

CHW: je aur bhi bataein hain matlab bachon k treatment hota hai toh us mein free mein hota hai koi matlab charges pay nahi karne hote dawaiyan free hoti hain matlab vaccination wagera bhi hamare yahan center mein horahi hai toh aap log asakte ho yahan par vaccination karwane k liye bhi agar aap refuse bhi kar rahe ho na phir bhi aap log yeh matlab joh hai faida utha sakte ho facilities

RA: acha aap k khayal mein k maaon aur nauzaida bachon aur 5 saal se kam umar bachon ko maloomat farhaam karne ko aap ko munasib tarbiyat ya training haasil hai

CHW: matlab pregnancy k doraan toh hamein yahan pe anc ki joh training mili huyi thi dr neelofar aayi thi tab

RA: kis tarhan ki training thi

CHW: toh us mein unhon ne matlab bataya tha k istarhan se maaon ko karna hai guide k pregnancy k doraan unhein tetanus k teeke lagwane hai iron supplyment leine hai is tarhan us mein matlab pregnancy k who matlab saving karni hai matlab pehle toh yeh project nahi thy project toh baad mein aaye jis mein who registration karwa rahe hai na pehle unhon ne hamein yeh sikhaya tha k aap ko is tarhan pregnant walon ko bolna hai k saving kar lein kahin bhi hospital jaane k liye paise hon k who us time pareshan na ho gaari ka arrangement wagera bhi ho aur tetanus k teeke joh laazmi lagte hain saatwein aathwein maheeney mein who lagwa lein aur matlab bachein ko joh newborn hote hain un ki care ka bataya karte thy ma aka joh pehla dood hai bache ko pilana hai dood zaya nahi karna hai toh is wajha se hamein bohat kuch sikhaya toh hum aurton ko field k doraan itna matlab kar sakte thy guide k yeh yeh cheezein hain joh aap k liye behtar hai

RA: aur kin cheezon pe training horahi hoti hai. In k elawa ki training bataein mujhe

CHW: in k elawa anc ki huyi thi training hamari aur

RA: acha joh joh aap ko training mili hai k aage kahin community mein cheezein bataein hain

CHW: je batai hain

RA: aur aap apne kirdar k hawale se batate hain k haan yeh yeh cheezein hum ne ki hai hamein idea hai training huyi hai aur is par hum kaam kar rahe hain

CHW: han batate thy saara kuch

RA: acha toh phir who kia kehte thy

CHW: matalab hamare area se jitni bhi pregnant thi jin ko need hoti thi toh un ko hum batate thy toh who yahan aati thi matlab facilities un ko milti thi matlab un ko injection bhi mil jaate thy un ko tablet wagera bhi mil jaati thi aur un ka ultrasound matlab AMNI jab start hua that oh un ka ultrasound bhi free mein hojata that oh yeh unhon ne facility haasil ki

RA: acha aur koi aisi maalumaat joh sahulatkaar aur aap k darmiyan aap ka kia us mein kirdar tha jaise community mein sahulatkaar se mil kar aap ka kia kirdar tha kia role tha aap ka

CHW: matlab hum log toh surveillance ki team rahe hai na toh pehle AMNI ka joh project tha us mein follow up kiye koi bhi masla hota tha women ko toh hum unhein refer kar deite thy dr shamim k paas toh phir wohi un ko treatment ya guide karti thi

RA: sahi toh kis tarhan ki training milti thi

CHW: hum toh un ko kehte thy k agar blood pressure ka masla hota that oh hum bolte thy hamare center pe joh doctor hain who best hain aap apna treatment karwa sakte hain toh matlab joh patient hoti thi apne sath le k aate thy toh joh dr shamim hai un ko dikhate thy k mam

yeh yeh aise women aayi hai un ka matlab yeh yeh masla hai who un ko karwati thi ya tablet wagera deity thi

RA: sahi toh kabhi aisa hua hai k community mein aap gaye ho yeh aap ne cheezein bataein zaroor hain aisa hota hai na k baaz auqaat agar bohat ziada gussa hojaati hain bhae aap kyun aate ho is tarhan ka kuch hua

CHW: je je abhi bhi hote hain matlab bolte hain k aap k aane ka matlab koi nahi hai hum kisi aur se karwa leinge matlab ziada tar hamein labeling k liye bolte hain k hamari deewarein kharab karte ho is tarhan (laughing) bohat issue yeh raha hai k labeling nahi karna hamare darwaze kharab kar k jarahe ho toh is tarhan se hoti hai toh ignore kar leina chaiye na

RA: toh phir aap ignore kyun karte hain aap un ko batate kyun nahi

CHW: batate toh hai na mam k matlab k yeh structure number hum likheinge nahi toh hamein kaise pata chalega k konsa ghar hai kis structure ka hai

RA: toh who maan jaati hain asaani se

CHW: je yeh kehte hain k kahin kone pe likh dou na hamari deewarein kharab karna na hi darwaza kharab karna

RA: acha is k elawa koi mushkilaat aap ko lagi k haan is tarhan se bhi hua hai k rukawatein koi aisi mushkil kabhi aayi ho community mein

CHW: nahi nahi

RA: aisa kuch hua hoga jaise k aap batana chahein toh

CHW: nahi nahi aisa kuch bhi nahi

RA: koi bhi aise hawale se nahi surveillance se aap mujhe bataein k surveillance se aap ko koi matlab aap k kaam ka kirdar kia tha aur sab kuch kaisa lag raha hai aap ko

CHW: acha lag raha hai matlab kaam bohat kuch sikha hai

RA: kia sikha hai aap ne bohat kuch

CHW:yehi k matlab tab chala sakte hain jaise who matlab joh nets wagera hain who sab kuch sahi samjh a gaye hain

RA: aur is k elawa.

CHW: (silent)

RA: joh aap ko lage k aap ki hausla afzai horahi ho joh aap karna chah rahe hain matlab kaam us mein hausla afzai horahi hai k haan aap ka kaam bara acha lag raha hai aap ko aur aap ko seekhne ko mil raha hai aisa kuch

CHW: je bilkul k matlab seekh rahe hain na hum abhi

RA: acha abhi seekh rahe hain kia seekh rahe hain

CHW: (laughing) matlab jitna un 9 saalon mein seekha hai who matlab pehle socha nahi tha na k kabhi itna matlab seekh paayeinge han yahan a k hum ne bohat kuch seekha hai abhi joh digital joh work hai

RA: koi ek aisi cheez joh aap ko lagta ho k 9 saalon mein joh seekha ho us se aap ko bohat faida hua

CHW: je bilkul yeh k matlab yahan a k awareness mili hai na hamein pehle is tarhan nahi that oh jab hum matlab jab hamaein awareness milegi toh dusron ko bhi hum de sakeinge matlab cheezein zaroori hai hamare liye joh aap apne lehaaz se karwa sakte ho

RA: kia ek aat kisi cheez ka naam bataoge

CHW: kis cheez ka

RA: jaise aap keh rahe ho na awareness mili hai

CHW: han na

RA: us mein ek aat cheez batao konsi cheezein hain joh bohat zaroori hai

CHW: yahan pe joh matlab joh pregnant women hain un ko sahi se guide kar rahe hai na doctor toh yeh keh jaate hain k awareness nahi hai aise bhi log hain jin ko kuch pata hi nahi hai na who teeke lagwate hain na who goliyan khaate hain na who ultrasound karwate hain toh phir yahan pet oh matlab sahi hai na jaise matlab facility mil jaayegi toh guide karte hain hum log k yahan pe aap log karwa sakte ho

RA: un ko joh teeke nahi lagwate hain

CHW: jeje je hum bolte hain k teeke kyun zaroori hai k jhatke ki beemariyon se bachte hain laazmi lagwani chaiye

RA: hmm toh jaise yeh kuch log aap ne bataya toh yeh particular area mein aise hain ya koi aisi community hai koi aisi zaat hai

CHW: community mein toh ziada tar log is tarhan se hi hain matlab k aap log a rahe ho kyun a rahe ho kia karte ho toh hum log un ko guide bhi karte hain matlab is wajha se aap k paas a rahe hain k jab tak hum log registration aap ki nahi karwayeinge tab tak hamara kaam aage nahi jaayega aur joh pregnant aap k ghar mein hai aap k paas chote bache hain ya koi chota bacha hai toh jab hum follow nahi kareinge toh hum log aage nahi barh paayeinge

RA: toh koi aa ziada tar kia cheez aisi hai joh aap ko sunne mein milti hai joh aap ne bataya na kyun a rahe ho

CHW: (laughing)

RA: is k elawa

CHW: is k elawa matlab joh pregnant hai who ziada keh rahi hain k matlab joh delivery abhi joh karwa rahe hain vital pakistan mein na toh hamein bhi matlab is tarhan se joh matlab nahi a sakti joh first se le k 4.5 maheeney ki joh pregnant hai un ki hai un k elawa matlab study mein nahi le rahe toh matlab kaafi aurtein hain joh matlab chahti hain k hamara bhi ho 8 month hai 7 month toh un ki study mein nahi hai toh hum un ko manah kardeite hain k study mein itne month tak hai aage nahi le sakte yeh log na toh who apni majbooriyan wagera batate hain toh matlab

RA: aap jab nahi leite toh kia kehte hain

CHW: hum un ko study ka hi batate hai na k matlab yeh study hai jis mein un ko hadh tak joh pregnant hai un ko leina hota hai us se aage nahi barh sakte matlab who chup hojaati hain study ka hai toh koi masla nahi toh who yeh majboori yehi batati hain k khaash hamara bhi hojaata

RA: toh us k liye aap ko kia lagta hai k hum kaise behtar kar sakte hain is cheez ko

CHW: is k liye toh yeh hai mam toh un ko facilities mile toh sahi rahega

RA: kis tarhan ki facilities

CHW: matlab delivery wagera ki

RA: aap ko lagta hai k tamamaam aurton ko milni chaiye

CHW: lagta toh hai matlab madam who toh project k enrollment pe depend karta hai na

RA: nahi leikin aap k khayal mein kia hai project ko hata k aap khud kia sochti hain

CHW: aa mein khud yeh sochti hun k matlab karwani chaiye kisi ki help hojaayegi is tarhan kisi k paas matlab paise nahi hote toh who log bohat pareshan hote hain kiraya bhi nahi de paate matlab who log apni majbooriyan bhi hamein batate hain toh matlab kaaf dukh hota hai k kaash un ko bhi yeh sahulat mil sake

RA: toh aap as a jaise madad karte hain kuch aur center tak le k ajaate hain toh un ko bata deite hain center pe le k ajao toh aap is hawale se aap kaisa mehsoos karti hain kia kia kirdar mehsoos karti hain

CHW: acha mehsoos hota hai k hum ne kisi ko sahi maloomaat di hai joh matlab sahi time pe yahan aate hain

RA: toh aur kia kia cheezein aisi hain jis k jaise pregnancy hogayi pregnant aurat hai aap ne un ko bata diya k center pe a jaayein is k elawa aur kia cheezein hain

CHW: newborn hai matlab joh nauzaida us ko matlab yahan pe hmm matlab check up us ka hojaayega koi masla hua toh doctor yahan pe joh hain un ko check kar k bataeinge

RA: hmm

CHW: bache ko sahulat milegi

RA: acha aur is k elawa hum yeh jaanna chah rahe hain k aap k khayal se sahulatkar as a facilitator community mein jaate ho toh aap us mein kia aa kia apna mehsoos karte ho aap kia role ada kar rahe ho kia kirdar hai aap ka k aurton ko center tak le k aane mein kis tarhan se aap madad kar rahe ho

CHW: hum log toh counseling kar rahe hain kyun k hum log un ko pick n drop nahi de rahe kyun k hum log un ko le k nahi a sakte apne sath matlab hum jis area mein survey kar rahe hain agar survey k doraan koi mil jaaye agar hum us ko counseling karte hain k aap hamare center pe yeh sahulat le sakti hain baaki raha pick n drop ka toh matlab pick n drop toh matlab hum nahi de sakte

RA: hmm toh aap ka aap ko lagta hai k sirf aap unhein maloomaat deite hain

CHW: je maloomaat deite hain ta k who aayein hamare center pe

RA: hmm

CHW: khud se aayein hamare center

RA: toh is pe aap joh unhein batate ho aap ko lagta hai k aurtein aap ki sunti hain

CHW: kaafi ajaati hain aur kaafi yeh keh k khamosh hojaati hain k un k paas paise nahi hain joh kiraya de k yahan Ibrahim hyderi goth tak aayein kyun k hamari noorani basti hai joh chakra goth tak hai wahan se un ko matlab paise deine parhte hain kiraaye k liye toh un k paas kiraya nahi hota joh de k yahan aayein toh un ki ziadatar yehi masla hota hai k aap gaari bhejo gaari mein matlab chal k aap k center pe aayein

RA: thek hai acha ek cheez aap ne batai thi k aurton ko maloomaat deity ho toh who maloomaat deine k liye aap ko aap k khayal se aap ko munasib training haasil hai

CHW: mere khayal se mam pregnancy k doraan joh teekon ka hai matlab shuro k maheeno mein joh folic acid wagera deite hain toh sahi se hum log guide kar rahe hain matlab 3 maheeney tak folic acid kha sakte ho iron supplement who bhi aap doctor se mashwara le k kha sakte ho khud se nahi matlab yeh k tetanus k baare mein hum log yeh batatey hain k saatwein aathwein maheeney mein har maa ko lagte hain joh k jhatkey k beemariyon se har aurat lagwa sakti hain

RA: thek hai toh is ki training aap ko mili hai

CHW: jee dr neelofer aayin thi unhon ne yeh kaafi saal pehle huyi thi mere khayal se ANISA tha ANISA ka project tha

RA: toh year yaad hai aap ko

CHW: year yaad nahi mere khayal se 2012 ya 13 hoga

RA: acha aur is k elawa aap logon ki training hoti rehti hain

CHW: nahi hoti

RA: acha

CHW: ANISA ki training hoti thi us doraan anc ki bhi training huyi thi us k baad joh matlab surveillance kiya tha us ki bhi training huyi thi

RA: hmm thek hai toh aap k khayal mein yeh training honi chaiye

CHW: je bilkul tab toh hum seekheinge

RA: aur kia hai us k baare mein batana chahogi k training kyun honi chaiye

CHW: (laugh) trainings hongi toh us se hamein maloomaat milegi toh behtar tareeke se logon ko guide kar sakeinge

RA: acha aur kitne arsey mein honi chaiye

CHW: matlab (laugh) who toh aap log select karoge na (laugh)

RA: nahi leikin phir bhi aap k khayal se

CHW: har saal honi chaiye ta k hum log naya seekhein aap logon se aur naya hi matlab community mein awareness di jaayegi

RA: acha aur kin kin cheezon mein aap ko batana chaiye training mein k aap jaate hain community mein log aap se poochte hain toh konsi aisi cheezein hain joh aap ko lagta hai bohat eham hai jin k baare mein aap ko maloomaat honi chaiye ta k aap foran se un ko bata dein

CHW: han yeh k matlab ziadatar logon ko yehi masla hai pregnancy k doraan delivery ka aur is k elawa kuch bhi nahi hai agar bacha beemar bhi hota hai na toh yeh keh k bol deite hain k kahin nazdeek ja k bache ka elaaj karwa leinge aap ka center hamein dur par raha hai hum nahi a sakte toh matlab joh zaroori hai who hai pick n drop agar pick n drop hoti toh hum har us bache ko refer kar deite center pe ta k us ka proper elaj hosake

RA: yeh toh ek rukawat a rahi hai

CHW: je rukawat a rahi hai

RA: thek hai mein abhi aap se yeh pooch rahi hun aap k khayal mein training aap k liye kin cheezon pe eham hai is pe hamari trainings honi chaiye ta k aap ko us ki maloomaat aap ko khud hogi toh aap community mein douge na koi ek aat cheez konsi aap ko lagta hai joh aap ko batana chaiye jaise aap ne kaha na pregnancy k hawale se aap se aurtein poochti hain toh is k hawale se aap se poochna chah rahi thi aisi konsi cheezein hain jin mein lagta hai k aap ko haan hamein training milni chaiye

CHW: mam is tarhan se toh bohat saari hai na

RA: jaise

CHW: jaise k (laughing) aap hamein matlab seekhaeinge who hamare liye sahi rahega na (laugh)

RA: seekhaeinge toh hamein pata chalega k konsi cheezein hain jin pe aap ko seekhane ki zaroorat hai tabhi toh seekhayeinge

CHW: Silent

RA: acha chalo koi baat nahi aa yeh mujhe bataein

CHW: yeh bohat tuff tha (laughing)

RA: nahi nahi

CHW: matlab bohat saari mam is tarhan ki hain na jaise hum ko yeh sawal karne

RA: nahi nahi bata dein koi is mein ghabrane ki baat nahi

CHW: (laughing)

RA: kyun k yeh who nahi asal mein hum karna isliye chah rahe hain k aap ko hum yeh deakhna chahrahe hain k hamein bohat saari cheezein aise pata chalti hai hum aap k mau se sunna chah rahe hain ta k hum us par behtar radoamal kar sakein koi step le sakein

CHW: je

RA: thek hai toh jaise training bohat eham hai eham cheez hai toh is mein poochna yeh chah rahe hain agar aap hamein bataein aisi konsi cheezein hain jin pe aap ko lagta hai k thori bohat aap ko taleem deine ki zaroorat hai koi information deine ki zaroorat hai thora train karne ki zaroorat hai ta k aap ka kaam assan hojaaye

CHW: mere khayal se mam hamein anc ki dobara hai training deini chaiye

RA: je thek hai anc ki training deini chaiye aur bachon k hawale se

CHW: aur bachon k hawale se bhi deini chaiye

RA: hmm aisa aap ko kyun lagta hai k anc aur bachon k hawale se deine ki zaroorat hai

CHW: kyun k anc agar hum logon ko sahi se aayegi ta k hum dusron ko guide kar paayeinge aur joh bache ki hai matlab newborns k hawale se toh agar un ko koi complication hai toh aap log hamein guide karoge ta k hum un ko refer kar sakein

RA: toh abhi aap joh bhi batate ho joh aap ko pata hai us k hawale se aap bata deite ho

CHW: je jitna hamein maloom hai k matlab bache ka joh pehla dood hai who zaya nahi karna hai aur bache ko padaishi teeka foran se lagwana hai ta k TB ki beemari se bacha rahe aur joh vaccination hai who bohat zaroori hai joh 15 maheeney tak di jaati hai toh yrh maloomaat un ko hum deite hain

RA: hmm acha yeh mujhe batao k yeh saari cheezein aap ne bataein

CHW: jee

RA: aisi konsi cheez hai joh aap sunte ho apne kaam k baare mein joh aap ko acha lagta hai community se ya center se jis se aap ka dil karta hai k aap aur ache kar sakti hain

CHW: community se suna hai mam k yeh aga khan k worker hain hamara bhi yahan hua tha ultrasound wagera test bhi karwaate hain aap bhi naam likhwa lou aap ka bhi hojaayega (laughing) community k joh log hain who agree hojaate hain matlab community mein apas mein karte hain baatein toh phir who agree kar k hamein jawab de deity hain

RA: acha toh yeh cheez achi lagti hai ultrasound hojaaye

CHW: (laughing) mam un ko chaiye bhi toh yehi na facilities kyun k bahar se joh ultrasound karwati hain charges lagte hain na toh matlab yahan toh free mein hojaata hai

RA: aur is k elawa ultrasound k elawa

CHW: ultrasound k elawa ab toh deliverian bhi horahi hain toh ache se milega jawab response aage se bhi

RA: aur koi aisi cheez joh kaam karne mein acha lagta ho is se ab next time aura cha kaam karongi

CHW: who toh hai mam ab toh kaafi acha kar chuke hain (laugh) inshaallah aage bhi acha hi kareinge

RA: acha yeh mujhe batao k yeh toh ultrasound horaha hai delivery horahi hai is ki wajha se aap k kaam mein asaani horahi hai aur aap ko acha lagta hai

CHW: je mam

RA: rukawatein kia hai kaam karne mein dushwari kahan aati hai

CHW: dushwari tab aati hai jab matlab joh labeling hai who nahi hai gharon pe ek ghar pe number hai beech mein bohat saare structure hain jin k number nahi hai phir last mein ja k number deakhna hota hai aur phir wahan se labeling ka kaam karte jaayein us mein bohat thak rahe hain labeling mein labeling hoti toh hum itna pressurize nahi hote

RA: hmm aap log joh number daalte ho us mein aap ko lagta hai k thora sa masla hai

CHW: thora sa masla hai par inshaallah taala who bhi hal hojaayega

RA: acha aur is k elawa community ki taraf se

CHW: community ki taraf se toh filhaal kuch nahi (laughing)

RA: jaise abhi toh bataya tha na k kehte hain rozana a jaate ho

CHW: (laughing) who toh kehti hain aajaate ho tang karne k liye toh phir matlab bura bhi lagta hai kia karein

RA: kia kehti hain koi aisa ek aat waqiya batao k kia hua kabhi k han is aurat ne mujhe yeh bola ho

CHW: (laugh) mam is tarhan se toh bohat saare hain joh kehte hain k mau utha k a jaate ho karte kuch bhi nahi ho kaagaz bhar k le jaate ho toh matlab hum sun k chup hojaate hain koi jawab nahi de sakte kyun k hamara kaam hai un se

RA: hmm toh matlab yeh sunna acha nahi lagta

CHW: acha toh nahi lagta matlab mam hamara kaam hai na hum phir chup hojaate hain

RA: sab se ziada kon bolta hai community mein koi particular zaat aisi hai joh bolti hai

CHW: nahi mam har blocks mein ek aat ghar aisa hoga joh matlab is tarhan se sonata hai

RA: hmm us ki wajha lagti hai aap ko matlab ek toh k bhar k chale jaate hain kuch deite nahi hai is k elawa kia wajha hai

CHW: is ki wajha yeh hai hamein bhi yehi bolte hain k bas apna salary wagera barhaney k liye ajaate ho hum log toh hamare paas hai joh khud se treatment karwa sakrte hain toh aap log mat aaya karo yahan pehle nahi hai matlab joh itna afford kar sakte hain toh hamein yehi jawab deite hain k aap logon k aane ka koi faida nahi hai hum private hi karwaate hain

RA: hmm matlab apni salary bharhane han hum afford kar sakte hain

CHW: toh is tarhan se bohat se sunne parhte hain

RA: kisi aur ki wajha se kabhi aisa laga ho k aap k kaam mein rukawat bani hai koi aur kaam ki wajha se

CHW: no mam is tarhan se nahi hua

RA: acha aur kia cheez hoti hai ek toh aap ne kaha bhae rozana a jaate hain labeling karna masla horaha hai

CHW: labeling isliye mam who kehte hain k abhi abhi hum ne gate pe color karwaya hua hai toh aap marker se kharab kar k chale jaoge toh hum unhein kehte hain k bas thora sa hum ne likhna hai phir baad mein aap color karwa lijiyega baad mein (laugh) color kar deina abhi toh likhne dou na ta k hum structure sahi se sequence de sakein

RA: hmm ek toh yeh dewaar pe likhne mein masla hai baaz aukaat dewaaron pe likhne se manah karti tabhi masla hota hai phir aap ko kaise pata chalega aur aur koi cheez k rozana a jaati hain

CHW: who toh hum batate hain na hamara kaam hai toh is k liye jab tak aap response nahi deinge toh hamara kaam aage nahi barhega toh isliye matlab poore area mein

RA: aurtein ziada aisa karti hain ya k aadmi

CHW: mard bhi is tarhan se hote hain matlab kuch log

RA: kia kehte hain

CHW: yehi k matlab agaye ab demag kharab karne toh field pet oh ignore karna parhta hai field k doraan is tarhan se bohat se aise log hote hain matlab logon k taane sunne parte hain koi kuch bol de koi kuch bol dein field hai us mein ignore karo aage barho apne kaam k liye

RA: taane kis kisam k

CHW: taane matlab joke hote hain un k joh peeche se maarte hain par unko ignore kar k aage barhte hain

RA: kia jin jin galiyon mein ja rahe hote ho us mein taane deite hain

CHW: je je

RA: acha kis kisam k taane deite hain

CHW: (laughing)

RA: han khul k bata dou joh cheez hai na ta k hamein bhi toh pata chale

CHW: mam is ko chor dou na

RA: (laugh)

CHW: (laugh)

RA: yehi toh poochne aayein hain yeh kaise chor dein

CHW: yehi na k mam salary barha rahe ho apna faida soch k hamara yeh matlab poora data le k chale jaate ho yeh who matlab toh bohat saare NIC card pe bhi issue karte hain toh who log nahi deite NIC card apne

RA: kyun nahi matlab aap toh itne arsey se community mein kaam kar rahe hain

CHW: phir bhi matlab mam un ko bharosa nahi shayad k aaj kal koi kisi ka bharosa nahi kar sakta hai toh us wajha se abhi joh hai matlab NIC card nahi deita hai ziadatar mobile number toh de deite hain matlab joh NIC ka card hai who nahi dikhaate

RA: nahi deite

CHW: nahi deite

RA: acha aur is k elawa

CHW: (laugh)

RA: aap k paas koi aur raaye ya khayalaat hain joh aap apne surveillance k kaam k baare mein batana chahti hain koi bhi aisi raaye ya khayal koi aisi cheez joh aap batana chahte ho jis se aap ka kaam behtar ho sake

CHW: trainings

RA: acha trainings acha aur ek toh training hogayi is k elawa

CHW: (silent)

RA: kisi bhi community mein aap jaate hain toh aisi kia cheez karni chaiye jis se aap ka kirdar community mein behtar kar sake jaise nauzaida bache hain aur maaon k hawale se aisi kia cheez joh hum aap ko dein jise CHW ka joh kirdar hai community k andar who behtar hosake

CHW: facility un ko milni chaiye na community ko matlab hamare center se un ko achi facilities milegi toh hamein matlab joh CHW hain un ko bhi matlab sahi response milega na hamari community se

RA: facilities mein kon kon si cheezein

CHW: facilities mein mein ne (laugh) aap ko bataya na mam pehla toh matlab delivery ka hi hai yehi hai

RA: k har har aurat ko facilities milni chaiye

CHW: han yehi kehte hain k aap log delivery karwao

RA: acha aur is k elawa delivery who hogayi aur ek aurat ka hoga na jaise pregnant hogayi un ki delivery ho aur joh pregnant nahi hai us k liye kia kehte ho

CHW: ma who bhi sunati hain na k bas matlab pregnant ka hi elaaj hoga hamara nahi hoga

RA: hmm

CHW: toh hum un ko batate hain hamare yahan pe research chalti hai jahan pe matlab pregnant woman hain matlab hamal k doran aur joh chote bache hain un ka hota hai 5 saal k chote bachon ka nauzaida bacho ka treatment hota hai baaki logon ka nahi hota un ko phir refer kar deine hain govt matlab mashwara deote hain k apna treatment Sindh govt hispataal se karwa sakti hain

RA: acha aap refer bhi karte ho

CHW: han sindh govt yahan nazdeek hai toh hum un ko yeh bata deite hain k aap Sindh govt mein ja sakte ho

RA: hmm ek toh delivery ki facility honi chaiye

CHW: aur dusra pick n derop

RA: pick n drop bhi hona chaiye joh aap k liye bohat eham hai us k elawa

CHW: filhaal toh yehi hai

RA: aur bachon k hawale se

CHW: bachon k hawale se yeh matlab joh team hai newborn ki who toh follow kar rahi hain shayad who bhi deite honge refer hum toh VR ki team hain hum apna survey ka kaam hi kar rahe hote hain toh joh matlab agar hamein koi bacha sick milta hai toh hum log is tarhan refer karein center pe yehi k agar gaari hogi joh jis team ko bol sakein k falah wali jagha se aap patient ko le k center jao toh yeh matlab kaam hota toh aur matlab behtar response milta community se

RA: hmm thek hai refer karna

CHW: je refer karna center pe is tarhan chahe patient pregnant hon chahe who newborn ho

RA: hmm toh us se aap ko lagta hai k aap community mein aur ache se

CHW: kyun k un ko faida hoga toh who dusron ko bhi boleinge k haan bhae hamein bhi is cheez se faida mila hai agar aap log bhi apna elaaj karwaoge toh aage aap k liye bhi faidemand hai

RA: acha thek hai thank you nasreen aur koi cheez joh aap batana chah rahe ho dil mein ho toh aap bata sakti hon

CHW; (laughing) nahi

RA: nahi chalo thek hai thank you ..

Date: 26102020 Recording: 30:19

COMMENTS: This CHW confused also hesitate that’s y cant speak more

RA: Je Acha NAME aap ki umar kitni hai

CHW: 45

RA: aur aap ki taleem

CHW: Matric

RA: Matric k elawa koi course

CHW: course mein ne silaai karhaai ki hai

RA: aap ka kaam karne ka tajurba kitne saalon ka

CHW: dus saal

RA: dus saal. Surveillance mein aap ko kitne saal

CHW: sueveillance mein mujhe 5 saal hogaye hain

RA: 5 saal thek hai . mujhe bata sakti hain surveillance k kaam k hawale se aap kaisa mehsoos karti hain

CHW: pehle kuch mushkilaat thi leikin ab bas hal ho chuke hain phir toh hamare liye asaani hogayi hai

RA: kaise asaani hogayi

CHW: pehle hum gharon mein jaate thy toh ajnabi hote thy hum log bhi who log bhi baat karne mein hoti thi pareshani leikin hum apna rawaiya sahi rakheinge apna face wagera smile wagera rakheinge ya rawaiya apna acha rakheinge toh phir koi pareshani nahi hoti

RA: acha toh aap ko lag raha hai ab pareshani nahi hai

CHW: nahi

RA: bilkul bhi nahi acha

CHW: kuch aise refuse ghar milte hain kuch agree karne mein time lag jaata hai agree bhi hojaate hain

RA: acha kis tarhan se agree karte hain

CHW: sab se pehle toh hum center ka batate hain phir hum joh project mein kaam kar rahe hain us ka batate hain

RA: aur is k elawa aur kia batate hain aap

CHW: aa joh koi kaam kar rahe hain project ka usi k baare mein batate hain k hum log kyun aate hain aap k paas

RA: acha who phir kia kehti hain aap se baat karti hain aap batate hain na k hum kyun aayein hain kis liye aate hain toh phir

CHW: haan health k silsiley mein batate hain k bachon ko teeke lagte hain un ka elajj hota hai toh who phir agree hojaati hain

RA: acha teeke lagte hain elaaj k liye aap bata deite ho toh who agree hojaati hain. Acha kahin pe aap ko lagta hai k koi agree bhi nahi hoti

CHW: han kuch ghar aise hote hain joh agree nahi hote

RA: abhi aap ko surveillance mein aisa laga k koi joh aap ko lagta hai k kuch maayein hain joh k jaise aap ne bataya refuse bhi niklein hain toh is tarhan koi problem huyi ho jaise aap ko lage k mother willing nahi hain bache ko center mein lane mein

CHW: bas gaari ka problem hota hai k aap ko pata hai community ka who kehti hain k hamein gaari provide ki jaaye toh hum aap k center a sakte hain aisa kuch aurtein kehti hain

RA: acha aur kia kehti hain

CHW: center ka toh hum bata deite hain un ko k bache ka elaj wagera bhi hota hai toh kehti hain k agar gaari ho toh center a sakte hain gaari ka masla hamein batati hain aane mein problem hoti hai dur hai

RA: acha aap bata sakti hain k hamal ya naizaida maaon aur bachon ki deakhbhaal mein aap ka kaisa mehsoos karti hain joh aap ka role hai joh kaam hai

CHW: jaise bacha paida hota hai toh sab se pehle hum anc batate hain foran aadhey ghante mein joh maa ko dood zaroori hota hai who pilana chaiye aur kis tarhan us ki care karni chaiye

RA: acha thora sa bata sakti hain care k hawale se aap unhein kia guide karti hain k kis tarhan se care honi chaiye

CHW: aa teekon ka batate hain hum aur dood ka babate hain joh sab se pehle joh ma aka dood diya jaata hai who zaroori hota hai who pilana chaiye aur teekon ki information hum batate hain

RA: aap se poochti hongi k teeke kyun lagate hain

CHW: han hum batate hain paidaishi teeka joh hota hai who bache ko dammey ki beemari se bachata hai BCG ka toh who foran bache ko lagwana chaiye

RA: aur kahan lagana chaiyeyeh poochti hongi

CHW: haan par hamara health center hai wahan bhi lagte hain

RA: toh aap ko kia lagta hai bachon ki deakhbhaal se related batati hongi unhein k kis tarhan se mazeed behtar karna chaiye

CHW: hum log batate hain

RA: sahi kis tarhan ka awareness deity hain aur kuch bataein

CHW: k 5 saal k bachon ko teeke lagwa sakti hain

RA: aur koi maloomaat joh di hon teekon k elawa aur

CHW: han k safai ka khayal rakha karein chote bachon ka

RA: acha aur kuch bataein surveillance mein aap kaisa mehsoos kar rahi hain aur is k elawa

CHW: agar koi sick bacha milta hai toh batana parta hai k aap center le jaayein doctor k paas le ja sakte hain

RA: toh kia who willing hoti hain kabhi aisa joh manah kiya ho

CHW: nahi ziadatar log manah nahi karte

RA: manah nahi karte aur koi pareshani joh aayi ho

CHW: nahi aisi toh koi pareshani nahi

RA: acha thek . aap ko kia lagta hai surveillance k kaam k elawa zachki or nauzaida bachon k hawale se maloomaat farhaam karna chaiye

CHW: han yeh zaroori hai karni chaiye

RA: acha karni chaiye kis tarhan ki maloomaat farhan karni chaiye

CHW: aa maa ko anc deini chaiye agar who hamal se hai toh teeke lagwa sakti hai aur apni giza ka khayal rakhe aur chote bache ka us ka bhi khayal rakhe

RA: acha jaise aap ne teekon k hawale se bataya deakhbhaal k hawale se bataya aur koi maloomaat di ho

CHW: who delivery k silsiley mein who kehti hain k hamari delivery hojaaye toh

RA: acha agar delivery ho toh khush hongi acha aur in k elawa kia kehti hain

CHW: aa kafi aurtein poochti hain k aap k center mein jaise delivery wagera hoti hain is silsiley mein poochti rehti hain

RA: acha aur kia kehti hain

CHW: hamare center mein koi aisa nahi. Project toh aate rehte hain

RA: un ki taraf se kia response aata hai aap k paas

CHW: toh phir yehi poochti hain toh hum bachon ka hi bata deite hain k hamare center pe chote bachon ka hi elaj hota hai jaise hamein training wagera di jaati hai bachon k sehat se mutalliq toh hum un ko batate hain

RA: kis tarhan ki training aap ko mili hai

CHW: ANISA mein di jaati thi hamein aur ANISA ki training di yeh batate thy

RA: kia batate thy aap

CHW: aa chote bachon ko baare mein bachon ka yehi k paidaish k foran baad dood deina chaiye bachon ko teeke lagwana chaiye

RA: aur

CHW: aur maa ko deite thy

RA: toh aap ko lagta hai k joh joh aap ko training di gayi hai aap ko munasib training haasil hai ya mazeed deini chaiye

CHW: agar mazeed deina chahein toh sahi hai

RA: kis kisam ki aap chah rahi hain k aap ko training milni chaiye

CHW: bache aur maa k mutalliq deite hi rehte hain wohi hum batate rehte hain

RA: aur kia batate hain aap jab aap jaate hain community mein

CHW: sab se pehle hum batate hain k hum kahan se aayein hain aga khan health center se aaye hain batate hain apne project k baare mein jaise hum log surveillance mein karte hain toh yeh VR karte hain aur 13 se 49 age tak hum un ka VR karte hain aur agar who pregnant hoti hain toh hum pregnant ncd add karte hain

RA: acha

CHW: aur 6 maa se chote bache hote hain toh phir un ko bhi add karna hota hai

RA: acha aap ko lagta hai k aur hamein mazeed community mein aur bhi cheezein batani chaiye who training hamein haasil ho jis tarhan aap chahte hain agar yeh hamein milegi toh aage bhi hum community ko bata sakeinge aur mazeed awareness de sakeinge

CHW: sehat k mutalliq toh honi chaiye (laugh)

RA: acha sehat k elawa jaise aap ne kaha tha na hum jaate hain community mein mother ko k aap ache se bache ko dood pilaayein deakh bhaal k hawale se batate hain giza khilaayein aur is k elawa

CHW: bachon ki sehat k baare mein aurtein poochti rehti hain ziadatar chote bachon k

RA: acha kis poochti rehti hain

CHW: k bache ko kia khilana chaiye ziadatar joh sick bache hon un ka hi batate hain k hospital le jaayein doctor k paas elaj karwaayein

RA: toh kabhi aap ko aisa laga k haan jaise maaon aur nauzaida ki deakhbhaal mein sahulatkar aur community mein aap ka kia kirdar tha

CHW: hamein joh yahan se training wagera joh di jaati hai who information hum wahan tak pohchahte hain toh pehle jaise log teeke nahi lagwate thy bachon ka sehat ka khayal nahi rakhte thy leikin hum log ghar ghar ja k batate hain toh ab maaein bachon ka khayal rakhne lagi hain teeke bhi lagwa rahi hain aur un ki sehat ka bhi khayal rakh rahi hain

RA: kabhi aisa laga k maayein teeke nahi lagwati thi toh us ki wajha kia hai

CHW: yehi k bachon ko bukhaar aata hai toh hum un ko yeh batate hain k yeh mamooli sa bukhaar hota hai toh saari umar k liye bachon ko takleef nahi hogi isi wajha se who awareness deite hain teeke kyun lagwai jaati hai kis liye lagwai jaati hai

RA: aap ko kia lagta hai k maayein asaani se maan jaati hain

CHW: ha nab kaafi agree ho chuki hain pehle bukhaar ki wajha se bukhaar aata hai toh hum teeke nahi lagwate thy

RA: aur koi pareshani joh aap ko community mein huyi ho aisa kuch

CHW: nahi community mein toh aisi koi pareshani nahi huyi

RA: acha aur jaise surveillance k kaam k douraan aisi konsi cheez jin se aap ki hausla afzai honi chaiye aap ko lagta hai k haan k hum acha kaam kar rahe hain phir bhi community se sunni par rahi ho

CHW: who toh community mein hum apna rawaiya sahi rakheinge toh toh community mein aise challenge aate rehte hain

RA: koi aisa waqiya batein joh pareshani aayi ho community mein

CHW: nahi kuch refuse ghar milte hain who hamein batate hain hum apna focus bata deite hain jaise koi masla hota hai toh k aap ko kyun aate ho rozana daily aate ho phir hum bata deite hain k is silsiley se aate hain aur who agree hojaati hain

RA: jaise aap ne bataya than a kaam ka tajurba kitne saal

CHW: 10 saal

RA: 10 saalon mein joh matlab ek toh aap ko pareshani ka samna karna para in 10 saalon mein

CHW: shuro mein jaise hum log naye log naye chehre deakhte hain gharon mein jaate hain phir kuch pareshani huyi thi lekin phir jaise un ko hum batate hain kahan se aate hain yeh kia kaam karte hain phir nahi hoti hamein pareshani

RA: maayein joh refuses karti hain kyun who kyun karti hain kis wajha se reason kia hai joh refuse kar rahi hoti hain

CHW: us ko pata nahi hota kyun k naye log joh aate hain gharon mein shift ho kar un ko kuch pata nahi hota center k baare mein

RA: jaise aap ne kaha tha na k problem bhi huyi thi refuses bhi a rahe hain phir un ko kia batatna chaiye ta k refuses bhi kam hojaayein

CHW: toh hum un ko center k baare mein batate hain k hamare center mein kia kaam hota hai elaj wagera bhi hota hai toh who agree hojaati hain

RA: toh phir maayein khush hoti hain han mere bache ki behtari k liye a rahe hain toh kabhi kehti hon k aap kyun a rahe

CHW: haan kuch ghar aise hote hain (laugh) k aap log roz roz kyun aate ho

RA: surveillance mein kabhi kuch aisa masla raha

CHW: nahi

RA: acha aap yeh bata sakti hain k surveillance k kaam k doraan aap ko koi rukawaton ka saamna karna para tha

CHW: nahi

RA: koi aisi mushkilaat aayein ho jaise aap ne bataya tha na k mother ne kaha tha k delivery k liye ultrasound hona chaiye pregnancy k liye kuch hona chaiye is k elawa koi aisi mushkil

CHW: nahi aisi koi mushkil nahi aayi

RA: k jaise yahan pe aane k masail hote hain maaon ko

CHW: han joh dur hain area who kehti hain k aap log hamein sath le chalein center

RA: aur koi problem aayi ho joh aap ko lage k community ko aitemaad nahi aisa kuch

CHW: kaafi logon ko aitemaad hai kaafi kuch

RA: poori tarhan se aitemaad hai

CHW: je haan jaise k yeh log aate hain bachon k baare mein poochte hain hamari sehat k baare mein poochte hain

RA: toh kabhi aisa nahi hua k mother ne kaha k aayein kyun hain kia faida

CHW: kabhi koi aisa bata deita hai phir hum usay agree bhi kar leite hain

RA: kis tarhan se agree kar leite hain

CHW: yehi k bachon k silsiley mein aate hain koi chota bacha hai toh agar us ka wazan wagera check karein larkiyan jab ghar pe koi bacha paida hota hai woh bukhaar check kareingi aur hamare center pe teeke lagte hain yeh batate hain

RA: teeke k elawa wazan wagera check kiya is k elawa

CHW: bas yehi

RA: aur kia cheezein aap unko farhaam karte ho

CHW: sehat k mutalliq deite hain

RA: sehat k elawa chalo sehat kis se related bachon se related

CHW: maaon se

RA: acha aur bachon se related

CHW: bachon se related bhi batate hain

RA: aur kia unhein mazeed maloomaat deini chaiye

CHW: aurton ko sehat k mutalliq

RA: aur kabhi aisa k surveillance se related koi aisi maloomaat joh deini chaiye ho

CHW: aisa toh kuch nahi

RA: aap k khayal mein konsi cheezein community ko behtar bananey mein madad kar sakti hai konsi aisi cheez

CHW: sehat k mutalliq

RA: sehat k elawa joh lagta hai k hum community ko behtar bana sakte hain koi bhi aisi cheez joh un k liye faida ho ta k aage ja k shikayat ka mauqa na mile hum un ko faida kaisa pohcha sakein jaise aap ne bataya tha na k jaisa aap ne kaha k pregnant women ko hamein sahulat deini chaiye

CHW: silent

RA: kia hum kar sakte hain aap k khayal mein community ko behat bananey k liye

CHW: sehat k mutalliq toh hum batate rehte hain un ko

RA: aur is k elawa joh aap ko lagta hai k maaon ki joh shikayat hai joh pareshaniyan hain un ko behtar kar sakein

CHW: silent

RA: jaise aap ne kaha aap ko training deini chaiye

CHW: haan sehat se mutalliq deini chaiye

RA: acha yeh bataein aap ki raaye mein gharon ki sateh par hamal ya nauzaida bachon k hawale se konsi maloomaat deini chaiye

CHW: hamal k muttaliq anc batate hain unhein k teeke kaise lagaane chaiye pregnant ko aur kia kia giza k baare mein batate hain giza k mutalliq kia khana chaiye sehat ka khayal rakhna chaiye

RA: giza k hawale se aap kia batate ho unhein

CHW: k aap proper cheezein lein vitamins ho jis mein bas yehi

RA: aur kia batati hain chalo sehat k lehaaz se teeke ka bhi bataya aur kia maloomaat de sakti hain

CHW: jaise delivery k liye kisi aur hospital mein apna naam likhwa lein kyun yahan ziadatar aurtein joh daayon k paas hi delivery karwaati hain

RA: acha daayon k paas se karwati hain

CHW: han

RA: toh phir aap ne bataya k delivery aap yahan karwa rahi hain

CHW: han hospital mein karwani chaiye

RA: hospital mein karwani chaiye acha aur is k elawa

CHW: silent

RA: kuch bhi joh aap unhein batana chah rahe ho maaloomaat farham kar sakein hamal ya nauzaida jaise aap ne kaha k sehat k hawale se giza k hawale se aur is k elawa kia behtar hum un ko maloomaat de sakte hain

CHW: silent

RA: in 10 saalon mein matlab joh in 10 saalon mein aap ki joh routine hai joh aap ka kaam hai aap ne wahan ja kar community mein maaon ko aur kia maloomaat di

CHW: in 10 saalon mein hamein training wagera di jaati thi sehat k mutalliq joh bataya jaata tha wohi hum batate thy k apne sehat ka kis tarhan se khayal rakhna chaiye

RA: matlab joh joh aap ko training di gayi thi sehat se related aap batate thy

CHW: han bachon ki aur maa k

RA: aur is k elawa koi aisi training joh aap ko haasil ho

CHW: surveillance k baare mein di jaati hai jis tarhan gharon mein hum jaate hain

RA: aur surveillance karne gharon mein jaate hain aur is k elawa konsi training joh aap ko lagta hai

CHW: nahi bas yehi

RA: acha thek hai kia aap k paas koi raaye ya khayalaat hain joh aap apne surveillance k baare mein batana chahti hain koi aise khayalaat joh apne kaam k hawale se batana chahti hon aap abhi kaam kar bhi rahe ho

CHW: silent

RA: bataya tha aap ne maaon se related aur pregnant aurton ki sahulat farhaam honi chaiye aur kia cheez honi chaiye

CHW: silent

RA: acha kabhi aisa suna hai k community mein kabhi aap ne apne hawale se acha suna hai k who kia kehte hain

CHW: haan kehte rehte hain (laugh) aurtein toh batati rehti hain k jab bacha beemaar hota hai k foran center le jao phir who kehte rehte hain k aap ki wajha se hum center le k gaye bache ki sehat sahi hogayi toh phir hum apne kaam se apne andar jaise feel karte hain k hum sahi behtar kaam kar rahe hain

RA: ap ko lagta hai k chalo yeh toh aap ne k un ko lagta hai k sahi se kaam kar rahe hain toh kabhi mushkil mehsoos huyi kabhi

CHW: han mushkil aise huyi k kahin kuch mushkilaat pesh aati rehti hai k manah kar deite hain k hum log nahi aana chahte aap k center mein toh phir manah bhi kar deity hain

RA: matlab manah karne ki wajha joh manah karte hain

CHW: ziadatar toh bache ka joh kehti hain teekon k baare mein agar who log jin ko pata nahi hota chali jaati hain dur hoti hain pareshan hojaati hain kyun k bache ko le k aati hain

RA: jaise k teekon k hawale se batate hain aap kabhi aisa kuch hua k teekon k liye refuse kiya ho nahi karwana hamein

CHW: haan bohart kam hoti hain refuse karti hain teekon k baare mein

RA: acha

CHW: aa kuch maayein hoti hain who 2 3 teeke lagwa kar hum ziada teeke nahi lagwaate bache ko bukhaar a jaata hai

RA: acha toh phir aa chalo phir teekon ki wajha se manah kar deity hain k bukhaar a jata hai aur joh laga ho aap ko bohat mushkil aayi ho hamein in k elawa

CHW: bas ziada kuch nahi

RA: aap ki raaye k hamein aisa kuch kia karna chaiye community ko behtar banane k liye k aap kia chahte hain aur kis tareeke se behtar kar sakein aitemaad mein laayein kis tarhan maaon ko convience kar sakein bata sakein aisa kuch

CHW: maaon ko toh hum awareness deite rehte hain k bachon k baare mein apne sehat k baare mein

RA: hmm

CHW: k aap log aayein hamare center aur hum apna batate thy k kyun aayein hain

RA: acha toh aap batate ho k kyun aayein hain toh phir wahan se response kia aata hai

CHW: acha aata hai behtar koi ek aat refuse hote hain ziadartar toh log sahib hi hote hain

RA: acha toh aap ne koshish poori hoti hai k refuses kam ho

CHW: haan hum kehte hain k refusal ko bhi mana lein hamari toh koshish yehi hoti hai

RA: acha aur koi joh aap ko lagta ho refuse k elawa aap ka joh role hai joh aap ka kaam hai joh aap ka kirdar hai us ko kaise hum behtar bana sakte hain

CHW: hum apne rawaiye se hi sab se pehle hum apna rawaiya sahi rakhna chaiye

RA: acha kabhi aisa laga k aap ko aap k rawaiye se un ko aise koi masaail huyi

CHW: nahi hum aisa rawaiya nahi rakhte (laugh)

RA: ya unhon ne aap k sath koi rawaiya ikhtiyaar kiya ho

CHW: nahi ziadatar nahi kyun k ab 10 saal ho chuke hain toh sab log hamein jaante hain

RA: jaanne k bawajood aisa kuch k aap ko laga ho k napasand kar rahe hon

CHW: kabhi kabhi (laugh) jaise kehte hain roz roz aajaate ho koi kaam wagera ghar pe ho toh manah kar deite hain

RA: acha who kehti hain k aap a kyun jaate ho

CHW: haan k hum kaam kar rahe hain baad mein ajaaiyega aisa kabhi keh deite hain

RA: acha toh phir kia hota hai

CHW: hum phir who dobara chale jaate hain ghar mein toh who busy wagera hoti hain toh manah kar deity hain

RA: acha jab who busy hoti hain aap dobara jab aate ho toh kia response milta hai

CHW: phir who sahi hoti hain

RA: acha phir asaani se maan jaati hain

CHW: jee

RA: aur mazeed bataein aur kia raaye honi chaiye nauzaida or maaon se related

CHW: chote bachon ka toh k kehte hain k ziadatar hum log jaise yahan pe le aate hain bachon ko toh dawaon ka bhi poochti rehti hain

RA: aur dawaon k baare mein poochti rehti hain

CHW: hmm bache k dawaon k baare mein poochti rehti hain chote bacha beemaar hota hai toh hum un ko center ka hi batate hain

RA: acha aur koi joh aapko lagta hai aur mazeed behtar karna chaiye

CHW: silent

RA: kuch bhi bata dein koi isi cheez aap k khayal mein joh aap ko lagta ho

CHW: silent

RA: deakhein joh bhi hum aap se pooch rahe hain joh aap ka kaam hain us kaam k hawale se hum aap se pooch rahe hain jaise aap ne kaha na k mother k liye bohat acha hojaayega k who delivery k hawale se pooch rahi thi yeh sahulat honi chaiye giza k hawale se aap ne batate rehte ho sehat k hawale se batate rehte ho aur kia cheez karni chaiye

CHW: silent

RA: koi bhi aisi cheez jo aap ko lagta ho k haan hamare liye yeh behtar hosakta hai is mein hamari behtari hai aur hum aage bhi maaon ko bata sakte hain aisa kuch bhi

CHW: gaari ka poochti rehti hain k agar gaari ho le jaaye center chorne aaye is ka poochti hain

RA: acha poochti hain gaari k hawale se

CHW: hmm

RA; kabhi aisa hua k gaari aa matlab kuch rukawat ka masla hua ho gaari k masail huye hon

CHW: nahi gaariyon ka masla nahi time pe kar leite hain

RA: acha aur kuch

CHW: bas

RA: bas chalein shukriya ….

RG-CHW1 Date: 02112020 Recording: 39:21

RA: acha name aap ki umar kitni hai

CHW: 22 saal

RA: aur aap ki taleem

CHW: mera BA continue hai

RA: aur aap k kaam ka tajurba kitne saal ka

CHW: surveillance ka toh saare 3 saal waise vital walon k sath bhi thora sa kaam kiya hai mein ne 5 6 maheeney

RA: sahi thek mujhe bata sakti hain aap k surveillance k kaam k hawale se aap kaisa mehsoos karti hain

CHW: surveillance ka kaam toh mujhe bohat hi acha lagta hai aur bohat comfortable bhi hai vital walon k sath bhi kaam kiya tha lekin us se kaafi yeh easy lag raha hai kaam aur matlab koi problem hoti hai masle hote hain toh hamare senior staff bohat ache hain koi bhi cheez poochein toh bare tafseel se aur easily bata deite hain matlab koi problem nahi hoti aur matlab koi mistake wagera ho bhi jaati hai toh ache se samjha k settle kar deite hain

RA: aap keh rahi hain na aap ko pehle se ziada yeh easy lag raha hai kaise

CHW: hum log joh hai na DSS k doraan jab hum ne TABLET pe kaam karna start kiya that oh hum log ghar mein maujood tamaam afraad ka data leite thy chahe who koi bhi ho un k sath reh rahe hain starting se chacha hain bhai hai bare bhai hain yahan tak k matlab joh over age hamare paas hote thy na buzrig un ko bhi hum log matlab joh us ghar mein sab ka data leite thy kaafi time bhi lagta tha aur kaafi saare issue aise aate thy k elaaj joh hai who aap sirf haamla aurton ka 5 saal chote bachon ka who sab ka le k jaate hain is wajha se matlab kaafi saare refuse wagera bhi hojaate thy aur is tareeke k kaafi aur who TABLET baar baar hang hojaata tha kaafi ziada hone ki wajha se is tarhan k bohat saare issue aate thy phir VR k doraan jab VR ki training wagera huyi who kaafi easy lag rahi thi kyun k VR mein hum joh hain shadi shuda aurat aur us k 5 saal se chote bachon ko refer kar rahe hain aur pregnancy ki joh tamaam pregnancy huyi hain un sab ko hum apne paas note kar rahe hain aur death aur newborn wagera bhi aur PW wagera kar rahe hain is tareeke se

RA: acha mujhe yeh bataein k pehle mein aur ab mein kaisa mehsoos kar rahi hain jaise aap ne kaha na pehle aap ko laga tha bohat mushkil hai ab aap ko asaan laga

CHW: VR k doraan kaafi easy hogaya hai hamare liye kyun k pehle hum log matlab tamaam afraad ka data jaise leite thy toh us mein bohat mushkil hojaati thi abhi toh hum 5 saal se chote bache hain un ka data le rahe hain vaccination wagera k baare mein pooch rahe hain un ki vaccination card mil jaata hai toh toh un ki date daalte hain nahi toh kisi tareeke se maa se pata kar k pooch k un ki vaccination ko hum deakh rahe hote hain aur is k elawa aur joh us ki aurat hai us ka hum ziada data leite hain husband k baare mein hum itna nahi poochte toh yeh hai k

is tareeke se kaafi poochte bhi hain na toh hum un ko easily bata deite hain hum aap ka data ziada isliye le rahe hain kyun k hum ne aap ka elaaj karna hai aur joh 5 saal se chote bache hain un ka elaaj karna hai vaccination wagera k kaam hamare center mein hote hain is tareeke se hum log kaam karwa rahe hain pehle hum log jaise jaate thy kisi k ghar pe kheir kheriat maaloom karne k baad hum apne baare mein bata rahe hote thy yeh batatey hain k hum 5 saal se chote bachon pe kaam karte hain aur hamla aurton pe waqfe wagera k kaam hamare center mein jitney project chal rahe hain sab k baare mein batatey thy vaccination k baare mein batatey thy har cheez k baare mein lekin phir yeh hota tha k jab hum unse data baaki logon se leite thy na toh who is cheez k liye bolte thy k bhae k tum log joh kaam bachon pe aurton pe kar rahe ho data sab ka le rahe ho is tareeke se kaafi log bohat irritate hojaate thy aur abhi joh hai na hum jis cheez k baare mein batatey hain usi cheez ka data bhi le rahe hote hain jaise hum aurat k baare mein poochte hain toh hum us ka data leite hain aur us ka elaaj hamare center mein horahe hote hain 5 saal se chote bache hain agar hum un ka data le rahe hote hain toh un ka elaaj hamare paas horaha hota hai yahan pe isi liye who easily data hamein de deite hain aur hum NIC wagera leite hain maa ki date of birth likhte hain apne paas NIC number mobile number toh is cheez pe bhi kaafi matlab kaafi log toh nahi de paate NIC who dar jaate hain aur kaafi matlab mil jaate hain mil rahe hain kaafi log matlab itna shaoor hogaya hai un mein jaan gaye hain toh who de deite hain pehle starting mein nahi de rahe thy

RA: acha toh dark i wajha kia thy

CHW: who NIC matlab elaaka aisa hai na yahan pe kaafi waardaatein bhi hojaati thi matlab hum log dawood goth mein kaam kar rahe thy toh ek aurat bhaagti huyi hamare peeche aayi k abhi hamare ghar mein do aurtein aayin thi aur who sona wagera sab kuch le k chali gayi kahin who aap log toh nahi thy jab hum ne card wagera dikhaya tab un ko tasalli huyi is tareeke se log bohat dare huye hain aur yahan pe bohat saari scheme kaam kar rahi hai toh who kuch sahi hoti hain toh kuch nahi hoti toh is wajha se log dar jaate hain

RA: jab aap ne card dikhaya that oh phir unhein tasalli huyi

CHW: haan kyun k hamare center wale kaafi arsey se kaam kar rahe hain is area pe aur kaafi saari logon ko sahulat mil jaati who aga khan walon ko pehchaan jaate hain toh is tareeke se log bohat dar jaate hain aur NIC ziadatar log deite bhi nahi hum pooch leite thy k aap ki date of birth us mein deakh leite ya zabani bata deite date of birth agar ziada force karo toh issue bana deite hain kyun aap NIC maang rahi hain kia kaam hai aap ka NIC se is tareeke se aur mobile number jahan tak hai toh who mil jaate hain hamein ghar pe kisi ek afraad ka mobile number mil jaata hai ziadatar aurtein apne husband ka number hi deity hain toh who hamein mil jaate hain mobile number aura b toh NIC bhi milne lag gaye kyun k joh card wagera hum batate hain na toh usi se hamara masla hal hojaata hai

RA: acha matlab mushkil kahan arahi hain

CHW: mushkil hamein vaccination card mein a rahi thi vaccination card ziada nahi dikhaati thi aurtein dur rakha hua hai ami k ghar mein rakha hua hai aur abhi hum kaam kar rahe hain ulmaari k andar hai dhoondna parega yeh who is tareeke se vaccination card dikha deity hain jin k paas saamne pare huye hote hain dikha deity hain joh kaam kar rahi hoti hain us tareeke

se nahi dikha sakti toh vaccine ki date joh hai na who mil jaati hai lekin kam milti hai leikin yeh hai k koshish hamari yehi hoti hai k who hamein kisi tareeke se vaccination card de dein ta k hum apne paas date note kar lein kyun k phir who waise hamein poochna parta hai k paidaish k foran baad wala injection aap ne lagwaya tha aur phir raano mein kitni baar lagwaaye thy phir 9 maheeney mein joh teeka lagta hai who lagaya tha 15 maheeney mein joh teeka lagta hai who lagaya tha is tareeke se who khud bhi confuse rehti hain aur hamein bhi confuse kar deity hain aur data hum enter bhi kar leite hain phir jab hum back checking hoti hai toh us mein kaafi misunderstand hojaati hai patients k sath isliye hamari koshish yehi hoti hai k hamein har cheez unse mil jaaye proof k taur pe koshish hoti hai NIC mil jaaye toh date of birth accurate hogi toh who agar nahi bhi dikhate toh hum unse pooch bhi leite hain k kitni umar mein shadi huyi thi nikaah naama agar saamne rakha hai toh who dikha dein jab aap ki shadi huyi thi jab kitne saal ki thi aap, aap ka pehla bacha kis umar mein hua tha us tareeke se hum is tarhan age nikaalne ki koshish karte hain k aap ki shadi itne saal mein huyi thi aur pehla bacha aap ka itne saal ka hai toh us tareeke se hum nikaal deite hain date aurat ki aur NIC mil jaata hai lekin thora who karti hain NIC deine mein dar jaati hain lekin jab hum poori information deite hain toh nikaal k de deity hain

RA: jaise k aap ne unhein maaloomaat de di har cheez ki information aap ne de di hai toh aap ko kia lagta hai k matlab who raazi hojaati hain

CHW: hojaati hain agree hojaati hain kuch joh nahi hote bohat kam hamein refusal milte hain aur hojaati hain aur abhi refusals is cheez pe mil rahi hain k baar baar data le rahe ho itna time hamare paas nahi hai naam likhwaya hua hai hum ne baar baar kyun k surveillance k doraan hum ne data liya poore poore ghar ka is k baad kuch time hum ne surveillance kiya us k baad VR agaya us mein dobara dobara se data leina start kiya us tareeke se kaafi log irritate hone lag gaye k baar baar aap log arahe ho data le rahe ho yeh kia Mazak hai aur phir hamari vital wali jarahi hoti hai who apne forms wagera kar rahi hoti hain toh us cheez se who kafi aksar log toh hamein deakh k hi darwaza band kar deite hain yeh aati hain itna time waste kar deity hain hamara

RA: acha darwaza band kar deity hain

CHW: hmm who hum darwaza bajaate bhi raheinge phir response nahi deite hain ache se phir hum log unhein refusals mein daal deite hain

RA: acha matlab aur kia kia kehti hain

CHW: matlab yehi abhi hum joh hai na is tareeke se bol deity hain k bohat time leite ho baar baar aate ho aap log baar baar data leite ho baar baar hum data nahi de sakte itna time kisi k paas nahi hota aur hamara matlab yeh hota hai k koshish hoti hai k un se poora ata leite rahe na toh who is wajha se bhi k aap log joh hai na har 2 maheeney baad a jaate ho aur dusre din a jaate ho data leine k liye baar baar poochne k liye ajaate ho is tareeke se kaafi kuch suna deity hain joh refuses hain kaafi log toh bohat ache bhi hote hain ache se response deite hain bitha k izzat se poora data hamein de deite hain who cheez hum maangte hain who la k bhi de deite hain aur kuch toh darwaaze pe hi khara kar deity hain phir jaati hai andar se kuch deina bhi hota hai toh jaati hain le k aati hain toh itni deir hum log dhoop mein khare rehte hain phir kabhi unka

mood hota hai kabhi kabhi aisa hota hai k aadha data de deity hain hamein phir husband wagera koi aata hai toh refuse kar jaate hain us cheez mein bhi manah kar deity hain nahi de rahe hamare shohar manah kar rahe hain

RA: acha information kia deite ho unhein

CHW: hum log jaise jaate hain toh pehle hamare paas sheets wagera hoti hain hamein di huyi thi joh hamara poorana data tha is ghar mein yeh aurat rehti hain pehle hum dua salam wagera kar k apne baare mein batatey hain k hum aga khan center ki taraf se aayein hain hamare center mein joh joh kaam horahe hain hamla aurton pe kaam kar rahe hain 5 saal se chote bachon ka har kisam ka elaj karte hain vaccination wagera bhi hote hain waqfe ka kaam hamare paas hote hain kamzor bachon k liye hote hain joh kamzor maayein hoti hain dood pilaane wali un k liye hamare joh hai na hospital mein kaam wagera horaha hota hai yeh saari information hum deite hain phir us k baad hamare paas joh sheet hoti hai us mein naam hote hain toh hum unse poochte hain aurat yahin pe reh rahi hai agar who bolti hai han yahin pe hunt oh phir hum unse data leite hain hum unka naam poochte hain un k husband ka naam poochte hain NIC mangwaate hain hamare paas us mein joh DSS hamari sheet mein likha hota hai us ko complete who DSS daalte hain hum NIC mangwaate hain NIC number daalte hain us ki date of birth daalte hain us k baad un ka phone number joh who de deity hai who likhte hain hum log apne paas TABLET mein aur konsi zaban ghar mein bolte hain who likhte hain phir hamara dusra option aata hai us ki pregnancy k baare mein poochte hain k matlab aap ki shadi k baad matlab poori total pregnancy aap ki life mein k aap ki live birth kitni hai matlab zinda bajhe kitne hain ya zinda bache kitne paida huye thy kuch aisa hota hai paida hone k baad intekaal hojaata hai acha koi bacha zaya toh nahi hua pait se mara hua bacha toh paida nahi hua aap ne khud se bacha zaya toh nahi karwaaya ya aap ko kisi ne bola ho k aap k bache mein masla hai toh zaya karwa dou is tareeke se who information leite hain us k baad hum un se poochte hain k aap k paas 5 saal se chote bache kitne hain phir un k bare mein poochte hain who bata deity hain k itne bache hain hamare un ka naam likhte hain un ki date of birth likhte hain aur joh hai na vaccination card maangte hain vaccination card milta hai toh hum vaccination ki complete date daalte hain yeh hamara matlab hota hai ek form is mein humare log events hote hain aurat k liye the hain pregnancies hoti hai un ki live birth still birth abortion aur files pregnancy hote hain hamare log death hota hai us mein aur joh bache ka hota hai us mein ek hi event hota hai who hota hai log death maa k do events hote hain log pregnancy log death aur bache ka sirf log death hota hai

RA: acha ye jo bi information batayi hai une tu ye ap matlab unko phele se hi malom thi information jo jo cheeze apne batayi hai ye ap aye ho tabi.

CHW: q asa hota hai na k shift ho jate hain log baher se arahe hote hai tu is liye hum log humara do maheeney baad visit tu hum sub kuch renew kr dete hai us se hume kuch aurton ko waqfa krwana hota tu wo apana naam likwati hain k hume waqfa krwana hai tu yaha hum vital team us aorat ka naam DSS wagera dete hai jate hai or is pe visit karte hain or us k family planning pe kam krte hai hamla aurtein hoti hain is tareeqe se mil jati hai or kuch aisa hota hai k do maheeney k andar un ko pregnancy hoti bhi hai who miscarriage bhi hojaata hai is tareeke

se who hamein mil jaati hain agar detail mein un ko batate hain na k yeh yeh project hai is tareeke se hota hai toh who hamein bata deity hain Is tareeke se

RA: sahi acha aap ko kia lagta hai k rozmara surveillance k kaam k elawa maaon nauzaida bachon k hawale se joh bhi maloomaat aap de rahi hain toh kis tarhan maloomaat de rahi hain

CHW: who isi hawale se hum bata rahe hote hain pregnancy k baare mein bata rahe hote hain hum aap ka mukammal poora elaaj kareinge service deinge gaari aap ko ghar se pick karegi hospital le jaayegi wahan se aap ko ghar pe le k aayegi aur joh hai na starting k month se le k 9 maheeney tak aap ka poora complete elaaj hoga hamare paas aap k visits honge aur delivery k liye bhi le k jaayeinge koi bhi ho eid ho hartaal ho kuch bhi problem ho gaari aap tak pohanch jaayegi delivery bhi aap ki hojaayegi aur aap ka jab bacha paida hoga who aap ko 2 din rakheinge wapis aap ko ghar pe bhi le aayeinge us k baad 6 maheeney tak aap k bache ka ghar pe a kar larkiyan check kareingi thek hai aur ache se counseling aur agar newborn hote hain us ki counseling karte thy 6 maheeney tak bache ko koshish karein sirf or sirf apna doodh pilaayein baahir ki koi cheez bhi na dein is tareeke se agar hum detail mein unse baat karte hain toh patient bhi khush hojaate hain k itni saari services hamein mil rahi hain yahan se

RA: in services mein kabhi koi mushkil aayi

CHW: jee mushkil yeh aati hai na k matlab who hamesha ek hi cheez ki shikayat karti hain k itna kuch bata k le jaati hain emergency hoti hai hamari call pick nahi ki jaati call pick karte hain toh wait pe bitha deite hain aur itne mein bacha phir ghar mein hi hojaata hai bacha ghar mein hi paida hojaata hai aur matlab is tareeke se kaafi saari matlab aurtein who bohat ziada shikaayat karti hain k aap itna kuch bata k ja rahi hoti hain phone karte hain toh phone pe ek dafa baat kar k dusi matlab ache se response nahi deity bohat matlab bure tareeke se baat karti hain aur dusri gaari mangwa dou aaj aayegi gaari batatey nahi k kis time tak aayegi late hi beithe huye hote hain kabhi kabhi gaari pohanchti bhi nahi patient k paas aur emergency hoti hai delivery ka time hota hai us time toh banda wait bhi nahi kar sakta yeh bhi nahi kehte k aap apni convience pe chale jaayein aur wait pe bitha deite hain hamein matlab gaari pohanchti bhi nahi hai hamare paas is tareeke se hota hai aur hum buri haalat mein hote hain aur joh hai na yeh yeh masle matlab hamare sath kar rahe hote hain aap log ki worker team itna itna wait karwaati hain k aurat ko joh hai na itna wait nahi karwaana chaiye haalat us ki aisi horahi hoti hai riaz goth mein hum kaam kar rahe thy toh who itni achi family thi hum har month un k paas jaate thy un k visits wagera ho rahe hote thy who har visit pe aati thi yahan pe matlab mukammal us ka elaaj kiya jab us ka delivery ka time aaya toh us ko kaha k aap apni convience pe Jinnah chali jaayein toh un k paas koi convience nahi thi yahan se bike pe beith k unhon ne bataya bhi k hamare paas bike k elawa koi convience nahi hai who yahan pe aayi hai bike pe beith k yahan se wapis gayi hai us haalat mein keh rahi thi k hum us haalat mein poora din ghumtey rahe hain who bacha bhi expire hogaya aur aurat ki bhi buri haalat hogayi aur who hamein ghar mein bhi nah ichor rahe thy aur us ne hamein yeh bhi bola k hum poore mauhalley mein ja k baat bata deinge k yeh bohat hi la parwaai karti hain is tareeke se aurat ki aisi condition horahi thi pehle kaha bike pe bitha k le aayein center yahan se hum gaari pe bheij deinge wahan se toh bike pe bitha k le aaye center yahan se toh gaari nahi di hamein toh phir hum log bike pe waapis gaye Jinnah toh Jinnah walon ne hamein matlab koi tha nahi jaane

wala unhon ne hum se sahi se baat bhi nahi ki aur isi tarhan bike pe jhatke watke se bacha bhi expire hogaya un ka is tareeke ki bohat ziada shikaayat karte hain

RA: acha aur shikaayat kahan community mein ki gayi hai ya aap k center mein a k ki

CHW: nahi center mein nahi jab hum jaate hain na door to door visit pet oh tab hum se yeh shikaayat karti hain itni achi achi families hoti hain refuse hojaati hain isi wajha se k itna wait karwaate ho phir jab center jao toh yeh ache se baat nahi karti hain kjaj wali un ka bol deity hain aur bolti hain gaari ki joh hai na service milti nahi hai bol k jaate ho koi aata nahi hai yahan pe poochne k liye bhi aur is tareeke se waqfe ka bol k rakhwa deity hain jab nikalwana hota hai toh masla ho toh nikaalti nahi hai bas bol deity hain wait karein wait karein is tareeke se phir

RA: acha aap ko bohat si problems aayin

CHW; hmm is hawale se bohat problems aati hain

RA: acha mujhe bataein aap k khayal mein joh joh aap ne information di maaon ko nauzaida bache 5 saal se kam umar k bachon k hawale se mother k paas aap gaye ho maloomaat aap ne di aap ko kia lagta hai joh bhi aap ne maloomaat farhaam ki hain us se munasib aap ko training haasil hai

CHW: jee training deite hain hamein

RA: acha kis tarhan ki training deite hain pehle hamare yahan session hote thy phir har din jaise ek din vaccination k baare mein batate thy hamein aur kabhi 5 saal chote bache k baare mein aur danger signs wagera k baare mein pehle bohat deite thy abhi nahi horahi hamare session trainings hamari bohat kam hogayi session hamare bohat kam hogaye hain starting mein hamein bohat deite thy session batatey thy hum se poochte bhi thy kia baat samjh aayi PW k baare mein batatey thy un ki care k baare mein batatey thy un ko aap ne yeh yeh ja k batana hai starting mein hamari bohat saari trainings bhi huyi thi session bhi hote thy hamari RA’s hain who hamein bata deity thi vaccination k baare mein bata deity thi har cheez k baare mein bata deite thy toh trained karte thy hamein yahan se trained kar k bhej phir hum ja k un ko batatey thy

RA: acha toh aap k khayal mein yeh training aur mazeed honi chaiye

CHW: session hona chaiye kyun k is se matlab bandey ko yaad aata rehta hai kyun k hum ek hi kaam mein lage rehte hain na hamein usi k baare mein pata rehta hai aur agar session beech beech mein hote raheinge na toh us se hamein matlab har cheez k baare mein mera khayal hai hamein math milti raheingi science mein har cheez mein changing aati rehti hai toh is liye mujhe lagta hai k hamein is tareeke se maloomaat milti raheingi

RA: matlab aap ko kis tarhan ki training dijaaye

CHW: session jaise pehle hamare session hote thy vaccination k baare mein hamara session hota tha vaccination k baare mein hamein sab batate thy chote bachon k hote thy 5 saal k chote bache phir danger sign wagera batatey thy is tareeke k bache ko deikhein pasliyaan chal rahi hain phir is tareeke se hamein bata deite thy who log har cheez k baare mein PW k baare mein

joh joh project hain waqfe k baare mein batatey thy aur joh project chalte hain na un sab k baare mein session milta tha

RA: toh matlab aap ko lagta hai k itni training joh hai aap ne samjhaayi hai batai hai information di hai yeh aap k liye itna kaafi hai training

CHW: matlab kaafi toh nahi hai mere khayal se hamein training milti jaaye milti rehni chaiye ta k hamari bhi knowledge mein izaafa ho hum logon ko aur bhi ache se samjha sakein

RA: sahi matlab aap chahti hain k aage bhi knowledge mein izaafa ho

CHW: hmm izaafa hota rahe aur hum joh hain na logon ko samjhaate rahein kyun k jitna hamein samjhaaya jaayega us se bhi aage hum logon ko samjha sakeinge jaise agar hamein bataya jaata hai vaccination kitni zaroori hai ziadatar log nahi karwaate bache ko bukhaar hojaata hai bacha poora din pareshan karta hai un ko is cheez k baare mein nahi pata hota aage ja k kitne bare problems ka saamna karna parta hai who jaise hamein ache se bataeinge yeh vaccination kitni zaroori hai un ko ja k bataeinge apne bachon ka vaccination card complete kareinge vaccination complete kareinge 6 ma se joh chote bache hain bache 2 maheeney ka hota hai toh maaein bachon ko bahir ki cheezein khilaana shuro kar deity hain bacha beemar hojaata hai ultiyaan motion lag jaate hain is tareeke se hamein pehle session diye jaate thy 6 maheeney tak maa bache ko apna dood pilana chaiye is k elawa aur kuch bhi nahi pilana chaiye ma aka yeh khayal hota hai bache ko piyaas lag rahi hai toh jab hamein yahan pe session diya tha bata rahe thy k shuro k 10 min 20 min tak bacha feed kar raha hota hai us ko sirf paani milao jis se piyaas khatam hojaaye us mein gaara dood aata hai us se bache ki bhook khatam hoti hai hum agar ja k patient ko yeh samjhaate hain na toh who is cheez se samjh jaati hain thek hai mere bache ki piyaas bhi khatam horahi hai bhook bhi khatam horahi hai aur joh aksar yahan pe community mein joh jaise hi bacha delivered hota hai pehla dood zaaya kar deite thy toh us k baare mein hamein kaafi bataya gaya hai k hum ne aage logon ko bataya ab who bhi nahi kar rahe bache ko pila rahe hain dood pehle wala

RA: acha sahi mujhe bataein k aap ko kia lagta hai k as a sahulatkar or nauzaida maaon k darmiyaan aap kia kirdaar ada karti hain jaise k aap facilitate karti hain community mein jaati hain service deity hain toh us mein aap ka kia kirdaar tha

CHW: hamara yehi hai k hum un ko matlab hum un ko bata deite hain ek kisam ka shaoor de rahe hain unhein kyun k yeh sab cheezein jab hamein training nahi thi hamein bhi in sab cheezon k baare mein nahi maaloom tha hamein maloom hua phir hum ne ja k is cheez k baare mein bataya tab un ko ja k pata chala vaccination kitni zaroori hai bache k liye 6 maa k bache ko sirf dood hi deina hai kitna zaroori hai is k elawa hamla aurat joh hai us ka khayal rakhna kitna zaroori hai is tareeke se hum ne ja k sirf hamara joh hai yeh kirdaar hai hum un mein ja k shaoor paida karte hain hamari counseling se agar hamari counseling achi hai agla banda joh hai jis tareeke se hum bol rahe hote hain us tareeke se ho us pe amal karte hain toh matlab achi counseling agar hai hamari toh hamara patient joh hai na who hamari baat ko samjhega bhi aur suneyga bhi us pe amal bhi karega toh yeh un k liye acha hai joh sehat hai who phir khud sehatmand raheinge bache bhi sehatmand raheinge is cheez k liye aur baaki pick n drop service joh hai who joh hai na hamari gaariyan jaati hain un ko hospital le k bhi aate hain wapis

le k bhi jaate hain yahan pe mukammal elaaj bhi horaha hota hai aur bachon ko bhi deakh rahe hote hain un ki deakhbhaal bhi horahi hoti hai kabhi elaaj horaha hota hai bachon ka bhi elaaj horaha hota hai toh yeh bhi kaafi sahulat hai kyun k phir aane jaane public transport yahan hai nahi toh aane jaane mein bhi kitna kiraya wagera lag jaata hai us cheez ko bhi deakh k aksar log hospital nahi ja rahe hote hain k bhae itna nahi hai hamare paas hum ghar mein hi totka wotka kar leite hain us se aur bhi kharab hojaata hai is tareeke se joh hamari pick n drop service hai us se kaafi logon ko yeh bhi sahulat hai waqt pe doctor k paas aate hain waqt pe un ka check up hota hai waqt mein un ki beemari ko pakar liya jaata hai

RA: acha jaise surveillance k kaam k doraan aap ko aur koi rukaawaton ka saamna karna para jaise aap ne kaha k aap ghar jaati hain toh maayein joh hain who sahi se jawab nahi deity aur koi aisi mushkil ka saamna karna para koi aisa waqiya banayan karein

CHW: bas is k elawa toh matlab kuch nahi joh refuse hote hain who isi wajha se horahe hote hain aur k matlab bata k itna kuch jaate ho hamara time bhi leite ho aur phir hamein service bhi sahi se nahi de rahe ho aap log aur aksar patients yahan pe joh hua tha who waqiya yahan pe hamare community mein hua tha who ek aurat thi us ko hamal tha us ka weight ziada that oh yahan pe jab who aayi thi toh unhon ne matlab thora sa Mazak banaya toh is wajha se bhi refuse hogayi k mera weight ziada hai toh wahan pe joh hai mera Mazak uraati hai jab hum gaye thy toh who pregnant thi hum ne unse kaha tha k aap hamare center pe visit karein us ne kahan mein center pe visit nahi karongi mera weight ziada hai mera Mazak banati hain Mazak uraati hain is tareeke se phir mein ne poocha bhi kon Mazak uraati hain unhon ne bataya bhi nahi hum ne kaha aap hamein batayein ta k hum aage ja k baat karein aaj yeh masla aap k sath hai kal ko kisi aur k sath hoga is tareeke se hamare refusal bar rahe hain unhon ne bataya bhi nahi

RA: yeh joh Mazak joh kar rahe hain toh yeh kahan pe

CHW: bas mein ne un se sirf itna hi kaha tha k aap hamare center pe visit karo toh unhon ne kaha hum ne visit kiya center pe aur mera weight ziada tha aur weight waaqi mein ziada tha 100 se bhi ziada tha us ka weight aur who pregnant bhi thi us ki bhalai k liye bhi bola ho k apna weight lose karein aur agar ache se bata deite unhein samjhaate k is is tareeke se weight loose karna hai ta k aap ko delivery k time problem na ho aur who keh rahi thi k mujhe aisa bola hai k tumahara wazan hi itna ziada hai bacha kaise paida karogi is tareeke se

RA: acha toh un ko aisa laga k un ka Mazak banaya hai

CHW: Mazak banaya hai han is tareeke se

RA: acha phir kia kaha

CHW: phir unhon ne kaha k mujhe pyar se batati bolti k aap ko delivery mein masla hoga is tarhan se kuch toh joh hai na phir thek rehta wahan itne saare patients beithe huye thy un k darmiyaan mein aisa bola k tumhara toh wazan itna ziada hai tum bacha kaise paida karogi is tareeke se matlab

RA: acha toh unhein bura lag gaya

CHW: jee bura laga yeh cheez

RA: acha aur koi aisi mushkil joh aayi ho in k elawa

CHW: starting mein toh hum toh kar rahe thy jab toh bohat hi ziada log shikaayatein kar rahe thy abhi joh hum kar rahe hain survey in dino hamein matlab itni ziada shikaayatein nahi mil rahi jab mein ne aap ko bataya yeh bhi hamare VR k doraan ek mili thi joh bacha expire hogaya tha aur yeh joh hai nay eh jab mein ne aap ko bataya VR se pehle ki baat hai

RA: aur jab aap surveillance k liye jaate ho community mein kabhi aisa suna k corona ki wajha se jaise aap jaate ho raaste mein koi corona keh kar

CHW: hmm nahi who aise joh raaste mein larke hote hain who toh waise vootings wagera karte rehte hain k bas mask wagera lage huye hote thy jab hum field pe ja rahe hote thy joh is cheezpe larke joh hain aisi choti moti shararat karte thy corona k hisaab se waise kisi ne hamein serious mein bola nahi hai matlab kyun k who jaante hain na hamein shuro se aur hum log is cheez ka kaafi khayal bhi kar rahe thy hum data le bhi rahe hote thy toh hum social distance ka khayal bhi rakhte thy hum log toh is wajha se kisi patient ne hamein nahi bola corona k hisaab se

RA: jab aap log jaate ho raasye mein aap pe vooting karte hain

CHW: haan who larke toh aap ko pata hai waise bhi kar rahe hote hain kuch na kuch shararat toh is tareeke se

RA: acha aur koi mushkil joh aayi ho

CHW: is k elawa toh koi mushkil nahi

RA: aur koi mushkil nahi acha aisi konsi cheezein hain joh aap k kaam k hawale se aap sunti hain joh aap ko acha lagta hai k aap ki hausla afzaai horahi hai aap aage bhi aap chahoge k kaam aur ache se karein

CHW: hmm patient k hawale se hamare staff k hawale se

RA: joh bhi

CHW: patient k hawale se kaafi saare patient ko achi services bhi mil jaati hai na toh jab hum wahan jaate hain hamari bohat ziada izzat karte hain aur matlab bohat ache se poochte hain bithate hain bohat ache se baat karte hain toh us mein hamein bohat acha lag raha hota hai bohat respect deite hain itni saari duaaein bhi deite hain k aap ki wajha se hamara joh hai na is tareeke se patient ne hamari itni taufeeq nahi thi ache hospital mein le k jaate aap log kaafi ache se hamare sath ho k bache ko deakhne aati hain visit karte hain hamara khayal itna ziada karti hain toh us cheez se kaafi matlab ache se hausla afzai bhi karte hainn matlab aur dil karta hai jab sab log isi tareeke se hamein duaaein karein joh refusal hain hum un ko bhi thora who kar dein ta k who bhi hamare paas aana jaana shuro karein aur hamara staff bhi jab hum acha kaam kar k aate hain toh bohat appreciate karte hain hamein us se yeh hota hai hamein aur bhi acha kaam karne ko dil karta hai is se bhi acha kaam kar k aayein

RA: acha kia sunti hain aap apne baare mein

CHW: community wale who toh is tareeke se acha kehte hain k matlab aap log ki wajha se itni saari achi sahulat mil gayi pick n drop ki service mil gayi hai aur itne ache ache hopitals mein hum ja rahe hain ache se hamari delivery horahi hai bohat acha maahaul hota hai matlab hamara who nahi hota hum budget nahi hota k ache hospital mein jaaye agar aap logon ki service na hoti toh zahir si baat hai kisi daai se delivered ho rahe hote ya kisi aap si clinic mein jaate matlab hamari gunjaaish nahi hoti bas phir ek toh hamein karza leina parta hai phir ache se delivery bhi nahi ho paati hai daai se delivery kahan ache se ho paati hai k phir kisi choti si clinic mein kahan aur Allah na kare kabhi emergency mein aisa hota hai bache ko joh hai na sheeshe ki zaroorat hai maa ko khoon ki zaroorat hai toh is tareeke se who bohat ziada appreciate karte hain har cheez hamein time pe mil jaati hai aur bohat ache se hamara kaam hojaata hai bache ka bhi bohat khayal rakhte hain maaon ka bhi bohat ziada khayal rakhte hain aur ache hospital mein le k jaate hain is tareeke se

RA: sahi acha aap k khayal mein konsi cheezein CHW k kirdaar ko behtar bananey mein madad kar sakti hai jaise aap community mein jaate ho jaise aap kehte ho na k yeh hum service bhi farhaam karte hain

CHW: abhi toh filhaal aisa nahi hai kyun k hum log jitni bhi un ko de rahe hain services who services matlab yeh hai k in areas k elawa kahin pe bhi nahi mil rahi ab jab hum jarahe hote hain kisi k ghar mein toh koi bahir ka banda araha hota hai hum apni counseling karte hain apne baare mein batatey hain toh who bilkul aise hairaan hojaate thy k kitni achi services aap ko yahan mil rahi hai aap ko shukarguzaar hona chaiye un ka is tareeke se matlab bohat acha lagta hai k aise areas mein jahan pe bilkul kisi cheez ki sahulat nahi hai ziadatar yahan pe gaari ki sahulat nahi hai jis ki wajha se patient time pe hospital nahi pohanch sakta who sab se bari service hum log de rahe hain yahan pe waqt pe gaari agar pohanch gayi patient k paas toh who waqt pe hospital bhi pohanch jaate hain us ka elaaj bhi hojaata hai aur who bach bhi jaate hain kabhi aisa hota hai who pata hai yahan pe gaari dhoondne mein bhi itna time lagta hai gaari milti bhi nahi aur agar gaari kismet se mil bhi jaaye toh who deakhte hain na k hum ek hi hain in ki majboori hai toh itna in logon ko bohat ziada hojaata hai toh who yeh services deakh k bohat khush hojaate hain log k chalo hamein achi services wagera mil rahi hain

RA: aise koi joh nakhush hon

CHW: nakhush toh bohat kam log hain abhi toh hamare refusals aur bhi kam hote jarahe hain who aisi cheezein hain k call pick nahi karte emergency hoti hai hamein thek hai aur call pick kar lein toh bata deite hain k han ajaayeingi gaari aap k paas hamein yeh bata dein k waqai mein gaari aayegi ya nahi gaari kabhi kabhi pohanch bhi nahi sakti kyun k patients bohat ziada hote hain phir who aise raaste mein khare hojaate hain toh is tareeke se matlab is cheez ki shikaayat karte hain k hamari codition aisi hoti hai k hum itna time khare nahi hosakte beith nahi sakte ziada aur kabi agar bohat ziada emergency ho toh who gaari phir bhi apne time pe hi aati hai agar aati hai toh is tareeke se koi agar patient hai jaise bata deite hain us ki pregnancy delivery ka time nahi hai leikin us ko bleeding hogayi hai aur bleeding us ko horahi hai aur joh hai na gaari ko phone kiya gaari aayegi apne hi time pe tab tak us ka miscarriage wagera bhi

hojaata hai zida hojaati hai bleeding is tareeke se shikaayat karte hain bas yeh ziada shikaayat wagera karte hain

RA: acha yeh bataein k kia aap k paas koi aur raaye ya khayalaat hain joh aap apne surveillance k baare mein batana chahti hain koi aisi cheez joh aap batana chahti ho jis se aap k kaam aur behtar hosake

CHW: aisa toh matlab joh hamari yeh saari services hai yeh toh kaafi achi hain bas ek yeh cheez hain matlab call waqt pe pick hojaaye aur ache se patient se baat ki jaaye toh who hamare liye bohat easy hojaayega is tarhan se refusal hamare bohat kam hum jinti bhi koshish karte hain na refusal kam karne ki toh who yahan center mein ajaate hain us tareeke se aur yahan pe center mein jab mein ek din aayi thi subha subha k time toh jaise mein wahan se andar huyi toh joh hamari joh maasi hain who patient se itni badtameezi kar rahi thi bohat ziada us ne kaha kmujhe token de dein is tareeke se kaha mujhe sahi se yaad nahi hai token pe us maasi ne patient k sath bohat ziada badtameezi ki jab mein aayi toh mein ne maazi ko kaha k aap ko chaiye k aap patient se ache se baat karein kyun k aap yahan pe staff hain jab hum un k gharon pe jaate hain toh joh aap ka hai na who hum se le rahi hoti hain k hum se ache se baat nahi hoti toh who hamein beech road pe darwaaze pe khare kar k itni insult karti hain hamein acha nahi lagta agar aap log inse rawaiya acha hoga token maanga tha aap un ko bol deity wait karein mein de deity hun token toh un se itni badtameezi ki k jao jao yahan se nahi hai bana rahi hun de dungi tum logon ko samjh nahi aati ek baar mein tum logon ko jao ja k wahan pe beith jao jab baneyga toh mein de dungi tum logon ko token is tareeke se bohat matlab bohat rudely baat ki thi maasi ne jab mein ne kaha toh us ne kaha tumse merakoi kaam nahi tum jao apne kaam se kaam karo mujhe upar se order aaya hai phir mein ne sir se baat ki thi mein ne sir se kaha tha k yeh yeh is tareeke se masle face karne par rahe hain un ko bitha k samjhaaya tha k patient hai toh hamara kaam chal raha hai patient nahi hai na aap ka kaam hai na mera kaam hai is tareeke se toh sab refuse hojaayeinge aur phir zaahir si baat hai deakhein k ek patient aur itne saare patient beithe huye hain toh who sab k sab hi thek hai who ghabra jaate hain toh yeh choti choti cheezein hain nay eh kaafi bare masle ban jaate hain na agar hum choti choti cheezon ko deakheinge un ko hal kareinge toh hamara hosakta hai acha hojaaye

RA: jaise aap k khayal mein choti choti cheezein konsi joh hum hal kar sakein aur behtar kar sakein

CHW: jaise matlab nasamjhi mein banda patient se badtameezi kar deita hai ek toh yeh patient hote hain beemar hote hain beemar banda ek toh chirchira sab hi hota hai unse badtameezi k yeh hai k thorasa hum deakhein ache se baat kareinge patient se toh who kabhi refuse nahi honge who joh refuse honge un ko bhi bata deinge ache se sab kaam horaha hai yahan pe pehle refusal bohat ziada thy ab bohat kam hochuke hain abhi hamare kaam mein kaafi behtari agayi hai yahan pe bhi ache se baat karte hain patient se hamare refusal abhi bohat kam hochuke hain

RA: acha aur kia behtari la sakte hain kaam mein

CHW: bas yehi mere hisaab se yeh hai k waqt pe pick n drop de dein aur patient se ache se baat karein aur matlab aisa na karein phir hum jab un k gharon pe jaayein who hum se

badtameezi karein matlab isi tareeke se baat karein kyun k who patient hote hain pehle se beemar hote hain itna chirchira hua hota hai upar se hum unse badtameezi karte hain toh who ghabra bhi jaate hain zahir si baat hai yahan pe a k ghabra jaate hain aur jab hum ghar pe jaate hain toh who hamein is tareeke se bol rahe hote hain bas mere hisaab se toh yehi

RA: aur is k elawa

CHW: bas

RA: bas yehi chalein shukiya bohat bohat…

Date: 02112020

RA: acha name aap ki umar kitni hai

CHW: 25

RA: acha aur aap ki taleem

CHW: matric

RA: aur aap k kaam ka tajurba

CHW: 2 saal

RA: aur surveillance mein aap ko kitne saal huye

CHW: surveillance mein yeh 2 saal huye

RA: surveillance mein hi 2 saal huye

CHW: jee

RA: thek hai acha mujhe bataeiyega name surveillance k kaam k hawale se aap kaisa mehsoos karti hain

CHW: bohat acha mehsoos karti hun sab se matlab yahan pe jitney bhi projects hain sab se behtar yehi hai acha hai

RA: aap acha mehsoos kia karti hain

CHW: jee field k hawale se matlab baakiyon ki field hai un se hamari field bohat ziada achi hai

RA: acha field mein kia cheez achi hai joh aap keh rahe ho k aap bohat acha mehsoos kar rahi hain

CHW: aa gharon pe jana jaise baaki larkiyon k paas machines wagera hoti hain who saamaan hamare paas nahi hai isliye

RA: acha aur kuch joh acha mehsoos karti hon aap surveillance mein rehte huye

CHW: bas yehi hai

RA: toh aap jab household visit pe jaate ho hamal ya nauzaida bache ki deakhbhaal mein aap ka kia kirdaar hai deakhein jab aap jaate ho gharon mein community mein jaate ho hamal ya nauzaida bachon ki deakhbhaal mein aap ka kaam kia tha

CHW: hamara kaam yehi tha joh hamal se aurtein hoti hain un ka naam likh kar aur joh newborn naye bache hote hain un ka naam likh kar aate hain ta k aage se team ja sakein na aur un ko capture karein jaldi se sirf un ki hamein entry karni hoti hai ta k jaldi se entry karein ta k yahan se teamein jaayein un ko capture karein aur bas yehi entry karte hain un ki aur agar jaise starting month hai toh un ko folic acid deite hain aur agar matlab 3 maheeney se ziada hote hain toh pharasulphar

RA: un ko koi jaise k koi aisi maaloomaat deite ho clinic mein le aane k liye

CHW: jee hum un ko samjhaate hain na yahan pe matlab RG k log ghar pe delivered karwaate hain hum un ko samjhaate hain k delivery hospital mein hi karwaani chaiye un ko yeh samjhaate hain

RA: kia un ka response sahi hota hai aap k sath

CHW: han sahi hota hai kuch kuch aurtein hoti hain joh matlab refuse kar deity hain k hamein gaari time se nahi milti ziadatar gaari ka issue hota hai k center walon ko hum call karte hain 2 ghante k baad gaari aati hai 4 ghante k baad ya call receive nahi karte yeh gaari ka issue hota hai ziadatar

RA: acha aur koi issue joh aap ko lagta ho k matlab joh aap information de rahe ho unhein maaon ko bachon ko gaari k elawa koi mushkil

CHW: nahi aur koi mushkil nahi gaari ka issue ziada hota hai

RA: gaari ka issue ziada hai

CHW: jee

RA: acha toh who kia kehti hain

CHW: who kehti hain jab hum gaari bulaate hain aap log hum jaate hain na toh un ko bolte hain gaari aap logon ko ghar se uthaayegi aur ghar pe choragi lekin jab call karte hain toh gaari nahi aati yeh ziada issue hota hai is tarhan se matlab kaafi deliverian ghar pe bhi hojaati hai gaari ko call karte hain gaari nahi pohan chti time pe

RA: acha toh mujhe yeh bataaiyega k aap ko kia lagta hai k aap ka joh rozmara ka kaam hai surveillance ka thek hai jis mein aap maaon nauzaida bachon k hawale se joh aap unhein maloomaat kis tarhan se farhaam karna chahoge

CHW: hum jis tarhan se kar rahe hain us tarhan se matlab sahi kar rahe hain

RA: jaise

CHW: jaise har 2 maheeney baad round lagaate hain abhi bhi VR k doraan hum har 2 maheeney baad ghar ka visit karte thy aur confirm karte thy k koi hamal se toh nahi hai ya nayi shadi wali family toh nahi aayi kisi ka bacha toh nahi hua hai

RA: aur jaise bachon ki sehat k hawale se

CHW: bachon ki sehat k hawale se sirf hum un ko samjhaate thy aur baaki yahan ki teamein who hai na deakhti thi un ko k kaise saaf rakhna hai bache ko bache ko feed baar baar karwaaye har aadhe ghante baad feed karwaaye ziadatar toh who cerelac wagera khilaate hain na toh who manah karte hain un logon ko

RA: acha aur kia kia cheezein aap batate ho unhein

CHW: sehat k hawale se bachon k liye bas yehi batate hain k har aadhe ghante baad feed karwaayein 6 maheeney tak bache ko feed laazmi karwaayein aur bache ki sehat ko saaf suthra rakhein yehi batatey hain un logon ko

RA: yeh joh joh cheezein aap unhein batate ho kia aap ko munasib training haasil hai

CHW: nahi koi training haasil nahi hai

RA: kyun training haasil nahi yeh joh joh information aap bata rahe ho aap ko training di gayi

CHW: nahi is pe koi training nahi di gayi thi

RA: toh aap kia chahte hain honi chaiye

CHW: haan honi chaiye

RA: kis kisam ki aur kis tarhan ki training honi chaiye

CHW: aa maa bache ki sehat pe training honi chaiye na ta k hamein bhi pata chale ta k hum un ko kaise bataein kaise samjhaayein is lehaaz se training honi chaiye is pe

RA: acha jis tarhan se aap samjha rahe ho kis tarhan se

CHW: who hum un ko batate hain k kis tarhan se bache ko saaf rakhna hai jitna hamein knowledge hoti hai har matlab aadhe ghante baad bache ko feed karwaayein usey cerelac wagera kuch na dein bache ko jitna pata hota hai utna hi batatey hain

RA: aur koi maloomaat joh deina chah rahi hon

CHW: nahi bas yehi

RA: bas yehi

CHW: jee

RA: aur koi training k hawale se joh aap chahti hon k aur bhi mazeed training honi chaiye ta k hum kaam ko behtar bana sakein

CHW: haan training toh honi chaiye matlab har cheez pe training honi chaiye maa bache ki sehat pe honi chaiye bohat achi training honi chaiye ta k hamari samjh mein bhi aaye k hum kia samjha sakte hain un logon ko

RA: sahi acha aap ko kia lagta hai k as a sahulatkar aur nauzaida maaon k darmiyan aap kia kirdaar ada karti hain jaise k aap facilitate karti hain community mein ja k services ka batati hain toh us hawale se aap ka kia kirdaar tha

CHW: ek worker ko matlab sehat k baare mein batana un ko

RA: jaise k aap jaate ho aap un ko apne kaam k hawale se batate ho services k hawale se batate ho aur kia kia cheezein hain joh batate ho yeh mera kaam hai is tareeke se

CHW: haan aur waqfon k baare mein batatey hain k waqfa karwana hai yeh who bas yehi hota hai

RA: aur koi aisi cheez joh aap unhein aur maloomaat de rahe ho

CHW: yehi k bachon ki hifazat matlab hamla joh aurtein hoti hain un ko kehte hain k delivery hospital mein karwana waqfey wagera in ka hi batatey hain

RA: acha surveillance k kaam k doraan aap ko koi riukawaton ka saamna karna para koi mushkil aayi community mein

CHW: han bohat ziadatar yeh lalabaad mein area hai jabal ka yahan par aayi hai yahan pe larke chichore hote hain tang karte hain aur koi rukawat nahi hai

RA: acha matlab larke kia kehte hain

CHW: ulta seedha bolte rehte hain ek matlab uncle tha who baar baar mujhe keh raha tha field k doraan k ajao hamare ghar pe lunch karo aur us k peeche takreeban sath aur bhi larke thy who bohat tang kar raha tha hum ne driver ko bulaya un ko kaha k yeh hamein tang kar raha hai aap yahan khare hojao ta k hum kaam karein bohat tang karte hain

RA: matlab who tang kyun karte hain

CHW: aa yeh toh pata nahi kyun karte hain lekin ziada tang karte hain larke

RA: who kia keh k tang karte hain aap ko

CHW: kabhi bolte hain number dou kabhi bolte hain hamara elaaj bhi karo hum bhi bache hain alag alag tarhan se who chairte rehte hain

RA: aur jab aap community mein jaate ho wahan koi mushkil aayi

CHW: in areas mein koi mushkil nahi aayi zaiadatar yeh lalabaad ki taraf hai jaise yeh RG k ho gaye yahan pe koi rukawat nahi aayi yahan par aisa who nahi tha

RA: aur community mein

CHW: wahan par bhi koi rukawat nahi aayi matlab community mein hum un ko bolte hain yeh cheez de dou NIC agar maayein koi sawal nahi karti toh who araam se de deite hain phone number wagera who araam se hi de deite hain

RA: kahin aisa k aap ko lagta ho k who NIC deina nahi chah rahe ho dikhana nahi chah rahe ho

CHW: nahi aisa koi bhi nahi hai yahan par hum un ko sirf bolte hain na who de deite hain yahan par yeh sahi hai

RA: acha aap ko lagta hai matlab sab sahi hai

CHW: han yahan par community mein koi mushkil nahi hai ziadatar yeh lalabaad yeh jabal ki taraf hai mushkil

RA: yeh toh chalo aap ne bataya tha k larke aap ko tang karte hain mein janna chahti hun k surveillance mein jab aap kaam karte ho community mein jaate ho toh wahan kahin mushkilaat aayin

CHW: nahi wahan par koi masla nahi aaya

RA: koi bhi aisa masla nahi aaya

CHW: nahi

RA: koi aisa waqiya nahi joh aap k sath hua ho

CHW: nahi aisa kuch nahi

RA: in 2 saalon mein aap ne joh kaam kiya hai us mein aisi koi problem

CHW: nahi aisi koi problem nahi aayi

RA: acha mujhe bataein k aisi konsi cheezein hain joh aap k kaam k hawale se aap sunti hain joh aap ko acha lagta hai k is se aap k kaam mein hausla afzai bhi hoti hai aur aap chahte bhi ho aur mazeed hum kaam behtar karein ache se karein ta k aur mazeed hamari hausla afzai ho

CHW: aisi toh matlab kabhi kabhi session hote hain who shauq se sunte hain ta k hamein bhi knowledge ho kuch

RA: aap apne hawale se aisi koi cheez k han yeh name bohat acha kaam kar rahi hain aisa kuch joh aap ko lagta ho aap k kaam k hawale se koi

CHW: kaam k hawale se who toh tareef toh hoti hai joh acha kaam karte hain un ki tareef bhi hoti hai

RA: aur is k elawa

CHW: bas

RA: joh aap ko lagta hai k hamein mazeed kaam ko behtar banana chaiye aura cha karna chaiye

CHW: hmm aisa toh kuch nahi

RA: aisa kuch nahi

CHW: nahi

RA: acha aap k khayal mein konsi cheezein community mein CHW k kirdaar ko behtar bananey mein madad kar sakti hai aap k khayal mein matlab jab aap jaate ho community mein

CHW: hmm

RA: toh aisi konsi cheezein hain joh lagta hai k haan hum un ki madad kar sakte hain is tarhan se hamara kirdaar bhi behtar hosakega aisa kuch

CHW: aisa toh kuch nahi hai

RA: community mein kia madad kar sakte hain aisa k aap ko lage k haan mera kaam ache se behtar hosakta hai

CHW: mein sochti hun matlab community mein ache se acha kaam karon ta k un ko har sahulaat mil sake aur us k liye wohi training wagera ho toh achi baat hai

RA: acha sahulaat kis tarhan ki mil sake unhein

CHW: un logon ko har sahulaat mil sake joh k hifaazat k liye matlab jaise who complain karti hain k gaari wagera nahi aati toh who un ko time se jaise call karein bhale 1 ghanta 1.5 ghanta lag jaaye un ko milni chaiye

RA: aisa laga aap ko k community mein aap gaye ho chalo aap ne kaha aap ko koi problem nahi huyi jab aap jaate ho maayein ache se baat karti hain kabhi aisa laga aap ko k maayein aap se gussa hogayi hon aap kyun a rahe ho is tarhan se kuch

CHW: haan is tarhan se hota hai matlab ek martaba visit kar k agaye dobara gaye toh bolte hain baar baar chale aate ho abhi toh kal toh aaye thy tang karne ajaate ho hum bhi kaamon mein hote hain hota hai aisa kaafi gharon mein

RA: acha phir aap kia kehte ho unhein jab who aisa kehti hain

CHW: phir hum un ko samjhaate hain matlab hum ne joh data liya tha us mein thora masla hogaya hai isliye dobara aaye yeh samjhaate hain un ko

RA: aur yeh kin kin gharon mein ek aat gharon mein aisa hua hai

CHW: aa ek aat gharon mein

RA: aur kuch joh aap ko lagta ho k community ko behtar bananey k liye kuch aisa karein k who humse khush hon

CHW: community mein behtar bananey k liye who har sahulaat toh de rahe hain na matlab har alag alag teamein hon har sahulaat un ko mil rahi hai

RA: aap ki taraf se kia un ko sahulat mil rahi hai

CHW: hamari taraf se bas yehi k jab hamal se hote hain un k naam likhte hain ta k yahan pe entry ho teamein jaayein un ko capture karein jald se jald hamari taraf se sirf yehi sahulaat mil rahi hai un ko

RA: aur koi aap ki raaye mein joh household ki sateh per hamal aur nauzaida bachon k hawale se konsi maloomaat deini chaiye

CHW: un logon ko yeh maloomaat deini chaiye jaise yahan joh community hai bachon ki hifazat matlab un k bache gande hote hain safai nahi rakhte un logon ko yeh session milne chaiye k bachon ko saaf suthra rakhein un ki parwarish sahi se karein yahan par safai nahi hoti ziadatar bachon ki aur ziadatar feed bhi nahi karwaate wohi bahir ka juice wagera hi pilaate hain bahir ki cheezein ziadatar khilaate hain

RA: aap kia maloomaat deity hain hamal ki khawateen hoti hain unhein

CHW: un logon ko hum yehi samjhaate hain k matlab ziada se ziada sabziyan khao fruit khao hum yehi maloomaat deite hain un ko

RA: aur koi joh aap ko lagta hai joh joh aap maloomaat de rahe ho lagta hai aap ko k who amal bhi kar rahi hain

CHW: aaa kuch toh karti hain amal yeh lagta hai aur kuch toh nahi karti

RA: aur kuch joh nahi karti who

CHW: phir un ko yahan se joh PW wali team hoti hain jaati hain un ko samjhaati hain

RA: acha aur koi joh aap ne bataya tha na k jab aap community mein jaate ho toh larke joh hai who aap ko is tarhan se matlab tang karte hain thek hai aap kehte ho k gharon mein jaate hain maayein sahi se jawab deity hain aur kuch aise joh who kehti hain k aap baar baar ajaate ho

CHW: kuch aise bhi ghar hote hain jaise hum jaate hain na toh larke aate hain who bolte hain hamein toh aurat se data leina hota hai larke aate hain k kehte hain hum se data lo matlab kisi ko bula nahi rahe aise bhi ghar hote hain aur ziadatar refuse bhi karte hain k hum nahi likhwa rahe aap k center mein

RA: acha toh refusal ki wajha kia hai

CHW: refusal kuch ki toh yeh wajha hai matlab koi ghar mein jaate hain who maar deite hain aurton ko kuch ki wajha yehi hai aur kuch kehte hain k wapis aise choti choti bachiyan hoti hain delivery karwaane k liye toh hamein nahi karwana matlab alag alag hoti hain refusal

RA: acha aur koi masaail aayin hon joh aap ko lagta hai center pe aane se who darti hon

CHW: nahi center se aane who kehte hain k yahan pe time bohat lag jaata hai hum beithe rehte hain subha mein aate hain phir 2 bajh jaate hain 3 bajh jaate hain toh hum jaise aaye waise hamara kaam hojaaye kuch aurtein yeh bhi kehti hain

RA: acha aur kia kia kehti hain

CHW: kuch aise refuse matlab kuch kehte hain k hum yahan se nahi karwana chahte hum private se karwa leinge kuch yeh manah karti hain k sarkaari hai hamein yahan se nahi karwana toh alag alag tarhan k refusal hote hain

RA: acha mujhe bataeiyega k koi aisi cheez joh aap batana chahti hon jis se aap k kaam aur behtar hosake aap k paas koi aur raaye ya khayalaat hain joh aap pane surveillance k kaam k baare mein batana chahti hon

CHW: mein surveillance k baare mein yeh batana chahti hun k do larkiyan hon matlab sath mein hon ta k acha kaam hon dar var na hon bas yehi batana chahti hun

RA: do larkiyan sath mein matlab

CHW: matlab abhi hum ek ghar mein ja rahe hain aur dusri dusre ghar mein jaati hain alag alag hote hain na toh sath hone chaiye larkiyan

RA: acha jab aap alag alag jaate hain toh aap ko us waqt kia mushkil aati hai

CHW: aaa jaise mera area hai na toh wahan pe mujhe dar lagta hai k koi matlab larke chichorapan na kare ya tang na karein yeh dar laga yahan par toh koi dar nahi hota toh koi aisa issue nahi hota

RA: acha toh yeh surveillance mein rehte horaha hai

CHW: surveillance mein

RA: surveillance mein rehte huye

CHW: jee

RA: acha aur kia batana chaheingi aap aur mazeed kaam ko behtar bananey k liye

CHW: aur kaam toh behtar hai

RA: aur kuch bhi joh aap k demaag mein ho k haan mujhe yeh bhi kehna hai

CHW: bas mujhe yehi kehna tha k do larkiyan sath honi chaiye ta k kaam acha ho behtar kaam ho koi dar var na hon

RA: dar aap ko kis cheez ka lagta hai wahan larke hain isliye

CHW: jee is wajha se kyun k kaafi larkiyon k sath badtameezi kar chuke hain larke joh jis area mein mein kaam kar rahi hun wahan par

RA: acha aur mazeed aur is k elawa

CHW: aur waise yeh kaam acha hai acha chal raha hai

RA: acha chal raha hai aur is k elawa bachon k hawale se kuch bhi joh aap batana chahti hon

CHW: bas

RA: bas chalein shukriya…

RG-SRA3 Date: 02112020 Recording: 25:37

Ss: Taj bibi aap mujhe bata sakti hain k aap ki umar kitni hai

SRA: meri 32

Ss: aur aap ki taleem

SRA: MA

Ss: Master kis cheez mein kiya hai

SRA: women studies mein Karachi university se

Ss: thek hai acha aap k kaam ka tajurba kitne saal ka hai

SRA: abhi yahan pe 10 years hogaye hain

Ss: 10 years hogaye hain surveillance ko mila kar

SRA: nahi nahi others projects mein pehle mein thi wahan par hips study mein wahan par bhi field ka kaam kar rahe thy field ka bhi kaam kar rahe thy wahan par bhi phir mein polio mein gayi 3 months ka project tha us k baad mein yahan surveillance mein aayi hun surveillance mein takreeban 2012 ya 13 se mein yahan par hun

Ss: 12 ya 13 saal se yahan pe ho acha surveillance k kaam k hawale se aap kaisa mehsoos karti hain jaise aap ne kaha aap ne polio pe kaam karti thi aur bhi dusre projects pe kaam karti thi aur jab aap surveillance mein aaye ho toh aap bata sakti hain k aap kaisa mehsoos kar rahi hain

SRA: kaam toh sahi hai lekin yeh thora say eh hai field karna field pe jana phir abhi yeh bhi hai k hamein akele field pe jana hota hai toh us mein bohat si mushkilaat hoti hai kyun k hamare

sath worker hua karti thi un k sath ja k itna measure issue nahi horaha tha akele ja k VR ko find karna bohat mushkil hojaata hai

Ss: matlab mushkil aap ko tab arahi hai jab aap akele jaate ho

SRA: jee

Ss: acha kahan aap ko lag raha hai muskilaat arahi hai

SRA: akele jana kyun k ghar ko find karna aage matlab wahan par mukhtalif log bhi hote hain aage kaisa area hai hamein kuch nahi pata aur ghar ko find karna kaafi time lagta hai toh is cheez k liye bohat who horaha hai

Ss: matlab aap ko mushkil arahi hai ghar ko dhundne mein

SRA: han mushkil bhi yeh mushkil sirf ghar ko dhundna nahi hamari security ka bhi issue hota hai na k aage kia ho kuch bhi hamein toh nahi pata hamare sath koi ek bhi hoga hamare sath koi ho toh hamein ek safety bhi feel hoti hai hamare sath hai toh chalo sahi hai na kuch areas aise bhi hai jahan par gaari bhi nahi jasakti toh banda akele ja k kaam kaise karega mein soch soch kar bohat pareshan hojaati hun

Ss: acha toh aur kia matlab pareshani arahi hai aap ko ek toh yeh k chalo aap ne kaha akele mein jaane mein masla horaha hai security ka bhi issue hai aur kia community mein aap ko lagta hai aisa

SRA: community mein han kuch areas aise hain jahan par hamein security ka bohat issue hota hai jaise ilyaas goth hai wahan par mostly wahan par nahi jaate hain toh kia bolte hain community k log bahir beithe huye hote thy phir un ki nazrein ajeeb si hoti thi toh phir unsecure feel karte hain kabhi kabhi mein apne sath drivers ko bhi le jaati hun k aap ajao hamare sath raho who a k hamare sath kaam karwa deite hain isi tarhan se hum apna kaam kar leite hain

Ss: acha toh aap ko yeh masaail arahe hain yeh surveillance mein hote huye

SRA: jee jee

Ss: us se pehle bhi

SRA: us se pehle aisa nahi tha kyun k pehle hamare sath larka tha jab pehle do projects mein larke thy toh un k sath hum ja k saara kaam kar leite thy ab yahan aaye us k baad hum yahan par bhi hamare sath larke hote toh itni problems nahi aati jab se yeh VR start hogaya hai us mein koi larki nahi bohat mushkil hota hai

Ss: acha toh aap k sath aur koi nahi hota

SRA: koi bhi nahi hota

Ss: acha toh yeh pehle aap k sath masaail nahi hote thy

SRA: nahi kyun k hamare sath pehle larkiyan thi is k baad larke thy toh us mein hamare liye itna problem nahi hota tha ab akele jana zahir si baat hai mushkil hojaata hai yeh baat hum ne aage bhi batai thi

Ss: aage batai thi aap ne

SRA: han na dr Zahra logon ko sab ko pata hai k yeh problems hain hamare sath

Ss: acha phir kia kaha

SRA: aaa (laughing) yeh aap khud poocho k kehti hain hamare paas k aise matlab sources nahi hai k hum larkon ka arrange kar k aap ko larkein dein ab hum ne bohat koshish ki k hamein extra koi larka worker dou ta k hum apna kaam toh kar sakein

Ss: acha sahi acha apne household visit se hamal ya nauzaida bachon ki deakhbhaal mein kia kirdar mehsoos karti hain aap jaise aap jaate ho community mein hamal ya nauzaida bachon ki deakhbhaal k hawale se joh aap ka kaam hai us mein aap kaisa mehssos karti hain

SRA: yeh hai k matlab kisi k aaa agar koi ek min mein confuse ho rahi hun

Ss: dekhein mein asaan alfaaz mein kahongi k jaise aap community mein gaye ho aap ka joh kaam hai kia hai surveillance mein

SRA: bachon ka follow ups karna koi sick baby ho un ko refer karna check karna aur agar koi bacha paida ho outcome deina aur yeh saari cheezein hain us mein

Ss: toh us mein aap ka kia kirdar tha

SRA: abhi yehi hai k monitoring karna hai hamara kaam field pe phir joh pregnancies hai un k outcome deakhna check karna larkiyon ka kaam kaisa horaha hai kia kar rahe hain aur kaam sahi kar rahi hain ya nahi kar rahi koi data miss toh nahi horaha hai koi bacha miss toh nahi kar rahe vaccines laga rahe hain joh data le rahe hain who sahi le rahe hain ya nahi yeh saara kaam hum kar rahe hain koi ghar lock hai refuse hai toh hum un pe ja k visit karte hain k yeh waqai mein refuse hai lock hai kia hai toh hum yeh daily ja k visit karte hain

Ss: chalein yeh toh aap ne bata diya tha k joh aap ka daily ka kaam hai who aap ne bataya k hum yeh cheezein karte hain

SRA: jee

Ss: visit bhi karte hain log bhi check karte hain mein janna chahongi k hamal ya nauzaida bachon ki deakhbhaal k hawale se aap ka kia kirdaar hai un ko kia batatey ho aap k kaam k hawale se

SRA: k hum jaate hain hum joh kaam karte hain yahan pe center mein kia facility provide karte hain patients k liye joh hamal se hoti hain un k liye joh bache hain un k liye kia kia cheezein hoti hain jaise vaccines hoti hai sahi hai na vaccination toh hum un ko yeh batate hain k aap log a k yahan vaccines lagwa lein apne bachon ko le k jaaye agar koi problem horahi hai toh joh numbers diye gaye hain un pe call kar k aap matlab emergency mein koi bacha sick hai ya koi

hamal se hai aurat hai agar un ko gaari pick n drop ki need hoti hai toh aap call kar k aap ja k yeh saari facilities aap le sakti hain yeh saari cheezein hum bata deite hain mothers ko agar koi folic acid hai farasulphate hai toh un k baare mein bhi mashwara deite hain k yeh aap ko cheezein khaani hai aur pregnancies mein kia kia cheezein khaani chaiye aur vaccines joh pregnancy mein lagte hain who saari cheezein hum bata deite hain maaon aur bachon ko

Ss: acha aur kia kia cheezein batate ho un ki deakhbhaal k hawale se

SRA: deakhbhaal means

Ss: jaise maaon aur bachon ki deakhbhaal k hawale se aur kia kia cheezein batate ho

SRA: I think bas I think yehi bata deite hain

Ss: acha aap ko kia lagta hai k joh rozmara surveillance k kaam k elawa joh maaon aur nauzaida bachon k hawale se joh aap maloomaat farhaam kar rahe ho aap joh bhi maloomaat farhaam kar rahe ho aap ko munasib training haasil hai poori tarhan se

SRA: aaa itni toh nahi hai training lekin jitni information joh hamein pata hai who hum un ko provide kar deite hain yahan se sahi hai na joh hamein bataya gaya hai k jaise k aap kisi bhi ghar mein jaate ho toh aap un ko bata dou k matlab hamare center mein joh joh facilities hai unse agaahi deina sahi hai na toh who log a bhi rahe hain force kar k jis tareeke se bhi ho who log a k matlab apne joh bhi cheezein hain kar leite hain yahan se a k kyun k hum log un ko inform karte hain na hamare center mein yeh yeh kaam horaha hai who log aate hain toh us mein un logon k liye bohat saare faide hain

Ss: aap ne kaha jaise koi training nahi di gayi

SRA: nahi

Ss: acha toh joh bhi aap maloomaat di hai who kia khudse aapne di

SRA: jee yeh toh hum apni taraf se kar leite hain na kyun k hamein pata hai na k joh kaam horaha hai usi hisaab se kar leite hain

Ss: acha aur is k elawa joh aap ko lagta ho

SRA: matlab is hawale se hamein koi session wagera ho toh phir hamare liye aur bhi easy rahega na k hum us mein mazeed aur behtari kar sakte hain jaise k matlab hamal se khawateen hoti hain un k liye aur mazeed maloomaat ho joh provide hum kar sakte hain us k liye aur bachon k liye jitna humse hosakta hai hum kar leite hain mazeed aur behtar banana chahein toh koi training wagera ho toh

Ss: acha aap ko kia lagta hai k as a sahulatkaar aur nauzaida maaon k darmiyaan aap kia kirdaar ada karti hain jaise aap facilitate karte hain community mein ja kar services k hawale se batate hain k aap center aayein toh yeh yeh cheezein aap ko mileingi is hawale se aap ka kia kirdaar hai aap ka kaam kia hai

SRA: joh hai bas itna who nahi hai bas sahi hai enough hai I think mere hisaab se joh cheezein hum deite hain us se ziada kyun k bachon ko le k who log aate hain mothers bhi aate hain

Ss: in 10 saalon mein joh aap ka kaam tha basically tha kia community se related

SRA: pehle hum log center base pe hote thy matlab 2 3 saal se hum field pe jaate thy itna hamein who nahi wahan par field pe wahan pe ja k PW k gharon mein ja kar un k baare mein poochte hain un ki sehat k baare mein poochte hain aur abhi bhi yahan par bhi yehi hai lekin yahan par thora sa kaam change hua hai abhi un larkiyon ka data check karna yeh saari cheezein hain

Ss: aur kia cheezein joh aap ko lagta hai aur mazeed apne kaam ko behtar banana chaiye

SRA: I don't an idea

Ss: acha chalein acha mujhe bataiyega surveillance k kaam k doraan aap ko koi rukawaton ka saamna karna para

SRA: phir wahi cheezein hain so shuru mein kahi

Ss: yehi cheezein hain chalo yeh toh aap ne bola tha na k do log hone chaiye sath mein ta k asaani hojaaye survey karne mein

SRA: jee

Ss: thek hai na aur is k elawa poochna chahongi koi aisa waqiya bayan karein

SRA: nahi aisa koi waqiya hua nahi hai mein apni taraf se kyun bolon

Ss: surveillance mein community mein ja kar

SRA: nahi koi aisa waqiya nahi hua

Ss: aisa kuch bhi nahi joh aap ko laga ho yehi masla hai aur koi masla nahi

SRA: abhi toh filhal koi masla nahi

Ss: acha community mein jaate ho kabhi aisa kuch hua hai corona k hawale se k kahin aap ko lag raha ho k kaam pe bhi rukawat arahi ho

SRA: yeh shuro shuro mein jab yeh kaam start hua tha VR toh hum field pe jaate thy abhi bhi jab hum mask pehan k jaate hain toh peeche se bache ho ya larke hon koi bhi ho phir bolte hain corona aaya corona aaya bhaag jao yahan se

Ss: acha aise bolte thy

SRA: aise jab hum shuro shuro mein field pe aaye kyun k ziada who tha abhi toh us mein hamein ek ghar mein mere sath ek waqiya hua k han ilyaas goth mein hum log gaye thy na toh phir wahan par bache k followup karne gaye thy matlab bache ko check karne gaye thy deakhne k liye k bacha kab paida hua toh data le k hum jaise ghar pe knock kiya toh phir andar

se aurat aayi us ne hamein andar aane nahi diya manah kar diya sahi hai na phir hum ne mushkil se un ko convince kiya hum ne reason poocha k aap kyun manah kar rahe hain hamare zehan mein yeh bilkul bhi nahi tha k hum ne mask pehne huye hain mask ki wajha se who log manah kar rahe thy k corona hai aap log corona ki wajha se aaye hain aap matlab kia bolte hain us ko aap logon mein corona hai sahi hai na andar nahi aana kyun k aap ki wajha se us se hum bhi effect hojaayeinge sahi hum ne bohat mushkil se un ko convince kiya convince karne k baad hum andar gaye aur wahan pe ek aunty beithi huyi thi aunty ne kaha mere bache par hath bilkul nahi lagana bilkul touch hi nahi karna hum ne kaha k aunty hum log bilkul bhi hath nahi lagaayeinge sahi hai na hum dur se beith kar aap ka data leinge aap khud deakh leina joh bhi hum kar rahe hain phir hum ne un ko samjhaaya toh unhon ne hank aha tabhi un ka data le k gaye unse bache ko hath tak lagaane nahi diya

Ss: acha kia yeh ek ghar ki baat thi ya aur gharon mein bhi aisa hai

SRA: nahi filhaal mere sath joh waqiya ek ghar mein hua us k baad mere sath yeh cheez nahi huyi lekin yeh hai k hum jab mask pehan k jaate hain na phir bolte hain corona corona corona

Ss: acha is tareeke se kehte hain

SRA: jee

Ss: acha toh phir aap ko lagta hai k is se kaafi aap ko dushwari huyi hai kaam mein

SRA: kaafi mushkilaat aayi abhi toh thore se logon ki habitual ban gaya hai is mein itna who nahi karte shuro shuro mein bohat ziada tha ab nahi hai ab thora sa who hogaya hai sahi hua hai

Ss: acha aur koi aisa joh aap ko laga ho maaon se related k ghar aate hain toh kuch

SRA: yeh hai k lekin mein yeh hai k mein field pe jaati hun na toh aurtein hath milaane ki karti hain toh mein manah kar deity hun (laugh) kyun k phir mein un ko batati hun un ko pata hona chaiye k aaj kal yahan hath milana manah hai kyun k corona ki wajha phir who bolte hain aisa kuch bhi nahi hai aap log aise hi khaam ma khaam dar rahe ho

Ss: acha toh matlab jab aap jaate ho toh aap se sahi baat karti hain aap hath nahi milaate phir un ka response kia

SRA: phir mein un ko sorry keh deity hun sorry aap logon ko pata hona chaiye k yeh cheezein hain thora sa ehtiyaat karna bohat laazmi hai toh phir who hans kar who cheezein taal deity hain

Ss: kahin pe aisa laga ho aap ko k maayein sahi jawab nahi de rahi hon chalo aap ne bataya tha k maayein sahi jawab deity hain

SRA: han

Ss: ek jagha kahin aisa laga ho k sahi jawab bhi na de pa rahi ho aap ko

SRA: joh refusal hai who sahi jawab nahi deite hain na aap log chale jao yahan se hamein kuch nahi karna hai mostly yeh aga khan ka naam sun k who bolte hain

Ss: toh un ki wajha corona toh nahi hai

SRA: nahi corona nahi hai who aise hi kar deite hain

Ss: shuro k maheeno mein than a

SRA: jee lekin abhi aisa kuch bhi nahi mere sath shuro shuro mein thora problem hogayi thi is k baa dab sahi hai

Ss: acha ab sahi hai aap ko lagta hai

SRA: jee sahi hai

Ss: acha aap k khayal mein konsi cheezein community mein CHW k kirdaar ko behtar bananey mein madad kar sakti hai

SRA: CHW k liye un k awareness k community mein kis tarhan se baat karni hai aap pehle jaate ho toh apne aap ko introduce karna phir ja k joh aap kaam kar rahe ho jis idaare mein aap kaam kar rahe ho us idaare ka introduction bohat laazmi hota hai kyun k mostly ek cheez mein ne note ki hai k direct larkiyan jaati hain assalam o alaikum bhi nahi boltin assalam o alaikum kaise ho mein aga khan se aayi hun bas aga khan se kyun aayein hain kis kaam k liye aayein hain kia wajha hai sahi hai nay eh cheezein hain toh un ki awareness k liye session hona laazmi hai CHW k awareness k liye ta k un ko pata chale k field pe ja k logon se kis tarhan se communicate karte hain aur kis tarhan se karna hai ta k hamein aura cha response mil jaaye

Ss: acha toh is se aap ko lagta hai k yeh awareness milne session karne se yeh hoga k aap k kaam mein aur behtari aayegi

SRA: jee bilkul kyun k bohat se CHW aise hain joh direct na salam na kuch direct ja k joh apna kaam hai who jaan churaane k us mein kar leiti hai toh us mein hamare liye bohat who ho jaata hai masla kyun k kuch patient hai joh bhi hai who us cheez ko deakh k phir who kar leite hain direct refuse kar deite hain kyun k un ko nahi pata na k kahan se aaye hain kis idaare se aaye hain kis wajha se gaye hain hum un k paas toh un ko toh nahi pata hota hai toh introduction deina laazmi hai lekin kuch workers aisi hoti hain joh yeh kaam nahi karte toh ek awareness session hoga toh thora sa behtar hojaayega

Ss: sahi aap ne kaha awareness session hona chaiye acha is k elawa mujhe bataein k aisi konsi cheezein hain joh community ko hum behtar bananey k liye hum un ki madad kar sakte hain matlab hum kia un k kuch kar sakte hain aisa joh k haan CHW k kirdaar aur behtar hosake

SRA: jee community k behtar k liye acha yeh hai k joh communities check up wagera k liye aati hai who sahi hai na toh un k liye I think mujhe nahi pata hai k doctors ka kirdaar sab se laazmi hai kyun k checkup wagera yeh saari cheezein doctors karte hain toh kuch patients satisfied nahi hote hain hamein kaafi cheezein sunne ko milti hai k hum jaate hain wahan par medicine deite nahi hain sahi se matlab sahi mau se baat bhi nahi karte kuch aisi cheezein bhi hoti hain

phir doctor k hawale se bhi hota hai yeh cheezein bohat saamne ajaati hai aap k center aate hain hamein jaldi faarig nahi kar deite hain ya sahi medicines nahi deite hain toh is wajha se bhi yeh hai k joh hamare doctors physicians hain un ki bhi thori si who ho counseling

Ss: acha aur kia kia kehti hain mothers

SRA: mothers yehi na k centers aate hain matlab kuch kuch mein pick n drop ka system bhi hai sahi hai toh un ko call kartehain pick n drop gaariyan wahan late pohanhti hai toh is wajha se bhi patients bohat refuse hojaate hain

Ss: acha jaise who call karti hain

SRA: jee call karte hain woh wahan pe un ka wait kar kar k din nikal jaata hai ya phir 2 ghante k baad pohanch jaati hai gaariyan toh services ko behtar karna laazmi hai patient k pick n drop community mein joh hum facilities deite hain na un ko aur behtar bananey ki zaroorat hai jaise pick n drop ki I think ek do gaariyan hon toh aur barha k 2 se 3 4 gaariyan aisi hon k patient ko ek dam se pick n drop dein phir who a jaayein jis kaam k liye aate hain

Ss: acha aap chahti hain aisa hon k

SRA: kyun k last time jab mein ne call ki na toh aage se ek bande ne mujhe call ki behan mein ne call kiya tha meri wife wahan us gali mein khari ho ho ho k chali gayi toh mujhe acha bhi nahi lagta saamne aadmi log bethe hote hain hamari aurton ko is tarhan se bethna allow nahi hai aap log itna who kar leite hain phir hum ne maazirat ki next time aisa nahi hoga toh hum kia karte un k liye

Ss: toh yeh surveillance mein hua hai ya phir yeh pehle se hi

SRA: yeh I think pehle se hi hai pick n drop hum deite nahi hain lekin hum who karte hain na calling PWs joh pregnant women hoti hain hum un k outcomes check karne k liye calls karte hain toh aage yeh cheezein hamein sunne ko milti hain

Ss: aur kia kia cheezein behtar bananey k liye mazeed chahte ho k aur mazeed behtar hona chaiye community mein ya CHW k kirdaar ko aur behtar bananey k liye yeh cheezein honi chaiye chalo aap ne awareness kahi toh aap ne kaha tha session hina chaiye awareness k elawa

SRA: us k elawa hmm

Ss: aap k khayal mein

SRA: filhal mujhe who itna nahi araha

Ss: chalein thek hai aap ki raaye mein household ki sateh par hamal aur nauzaida bachon k hawale se konsi maloomaat unhein deini chaiye jab aap gharon mein jaate ho aap k kayal mein

SRA: hmm un k diet k hawale se unhein batana chaiye sahi hai na aur un k saaf suthraai k hawale se k bache ko kis tarhan se rakhna hai kia karna hai sahi hai na aur bachon ko kia diet deini chaiye jab mother hamal se hoti hai toh un ko kia karna chaiye apne safai ka khayal

rakhna chaiye aur yeh saari cheezein joh bachon aur maa se related cheezein hain na un ko batana chaiye sahi hai aur vaccines k baare mein kyun k mostly kuch aisi hoti hain joh vaccines bhi nahi lagaati hain aur kuch aise hain joh diet ka bilkul bhi khayal nahi rakhti sirf chaaliyan wagera khaati rehti hain sahi hai nay eh cheezein agar hum un ko bata deite lekin yeh hai k mujhe nahi lag raha change hone wala hai kyun k is se pehle bhi kaafi log haan haan bolte hain baad mein phir wohi kar leite hain

Ss: acha aap keh rahi hain diet k hawale se matlab bataein bhi sahi toh koi faida nahi

SRA: mushkil nahi kareinge ek suggestion toh de sakte hain k hamara joh farz hai hum un ko bata sakte hain k aap ko yeh yeh karna hai

Ss: acha yeh toh aap ne bataya tha k mushkil batai thi aap ne unhein asaan kaise kiya jaaye un ko maloomaat kis tarhan ki di jaaye

SRA: implement karein amal karein agar hum maheeney mein maheeney mein takreeban ek do session kar lein agar baar baar in ko reminder daalein may be possible who us par amal karein ek dafa karne se toh nahi hoga agar baar baar in k batane se ya reminder deine se hosakta hai k thori si changing ajaaye may be possible

Ss: acha kia aap k paas koi aur raaye ya khayalaat hain joh aap apne surveillance k kaam k baare mein batana chahti hain koi bhi aisi cheez joh aap ko lagta hai k mujhe batana chahti hon jis se aap k kaam aur behtar hosake koi bhi aisa khayal joh aap k demaag mein hon

SRA: filhaal toh kuch bhi nahi hai (laugh)

Ss: surveillance k kaam k baare mein kuch bhi nahi hai

SRA: nahi pehle se abhi behtar hai pehle bohat mushkil hota tha na ab behtar hai isliye thori si itna who nahi hai jab pehle hamare sath joh problems hoti thi who hum ne dr Zahra walon ko batai toh

Ss: kis ko bataya tha

SRA: dr Zahra walon ko

Ss: acha

SRA: kyun k pehle hum yeh hard copy pe bohat ziada focus karte thy ab saari cheezein mostly khatam huyi hai ab saara kaam hum tab k andar kar rahe hain toh itna mushkil nahi horaha hai

Ss: acha itna mushkil nahi hora

SRA: jee

Ss: acha aur koi surveillance k hawale se

SRA: nahi ab behtar hai

Ss: behtar hai ab

SRA: ab behtar hai

Ss: acha aur kuch batana nahi chaheingi bas chalein shukriya …

Date: 27-01-2021

SS: Assalam o Alaikum NN

NN: Walaikum salam

SS: mera naam SS hai mein aga khan hispataal ki taraf se aayi hun thek hai

NN: jee

SS: aaj mein aap se aa joh hamari CHWs aap k ghar aati hain un k hawale se baat cheet karongi thek hai hamari joh yeh tehqeeq hai hamara maqsad yeh hai community mein joh hamari workers kaam kar rahi hain un k zariye is kaam ko aap tak kis tarhan se behtar banaya ja sakta hai thek hai

NN: hm

SS: aaj joh bhi mein aap se baat cheet karongi yeh hum joh hai kisi k sath share nahi kareinge sirf is ki research hogi is k joh bhi anjaam hai who likha jaayega lekin aap ka naam kahin nahi aayega thek hai

NN: hmm

SS: aur aap ki joh recording kar rahe hain yeh bhi hamare elawa koi aur nahi suneiga thek hai acha sab se pehle mein aap se yeh poochna chahungi k aap ki umar kitni hai

NN: Meri umar 22 saal

SS: acha 22 saal hai acha thek hai aur aap ko yahan rehte huye kitna time hogaya hai

NN: shadi hote huye saat saal huye

SS: aur yahan reh rahe ho saat saal se

NN: jee

SS: aur aap ne taleem li huyi hai aap ne

NN: jee mein ne thori li huyi hai

SS: acha kahan tak parha hua hai

NN: middle parha hua hai

SS: middle parha hua hai thek hai yeh toh bohat achi baat hai acha thek hai acha mujhe yeh batao k koi kaam wagera karti ho

NN: machine chalaati hun

SS: matlab kapre wagera seeti ho

NN: jee haan

SS: acha kahan see k bheichti bhi ho ya aise hi

NN: nahi nahi joh deita hai us ko sil k de deity hun

SS: joh bhi deita hai us ko sil k de deity ho

NN: jee han

SS: acha

NN: seelai ka kaam karti hun

SS: seelai ka kaam karti ho aur is k elawa koi aur kaam karte ho

NN: pehle karhaai kiya karti thi pehle ghar k ikhrajaat bohat hua karte thy toh phir us ki wajha se machine chalaati hun

SS: thek hai yeh toh achi baat hai abhi bhi karhaai kiya karo (laughing)

NN: nahi karhaai mein bohat time lagta hai

SS: bohat time lagta hai us mein ziada time lagta hai bache hain aap k

NN: jee

SS: kitne bache hain

NN: 3 bache hain

SS: 3 bache hain thek hai aur kitne bare bare hain

NN: bare 1 beti hai 6 saal ki dusri 4 saal ki aur ek beta hai 17 maheeno ka

SS: 17 maheeno ka abhi hua hai

NN: jee

SS: acha thek hai acha mujhe yeh bataaein k joh CHWs hain who baqaaidgi se ghar aati hain

NN: jee haan

SS: aati hain acha aur jab who ati hain toh who kis kisam ka kaam karti hain

NN: agar yani delivery se hote hain who pehle delivery k doraan pooch taach karte hain hamara yani k khayal rakhne k liye aate hain agar bacha paida hojaata toh phir who baar baar bache ka checkup karne aate hain jab un ki date hoti hai hamein le k jaate hain wahin pe agar hamara hifazati teeke nahi lage huye hote toh un k baare mein hamein bataate hain phir hamein yahan se le k jaate hain

SS: aap ko le kar bhi jaate hain aap k paas aati hain delivery k doraan bhi aati hain jab aap hamal se hoti hain tab bhi aati hain aur jab bacha paida hojaata hai toh bache ka checkup karne

NN: jee jee tab un ka time hota hai 6 7 maheenay tak jab un ka time hota hai who regular se aate hain jab un ka date hota hai toh regular aate hain bache ka bukhaar check karte hain

SS: haan

NN: aur us k baad wazan karte hain bache ka bukhaar check wazan wazan

SS: acha aur hamal k doraan jab aap hamal se hoti hain toh who us waqt kia kaam anjaam deity hain

NN: us doraan bhi mere khayal se baazu check karte hain BP check karte hain aur hamein agar yani 3 maheeney hojaate hain toh hamara form bhar k jaate hain kehte hain k aga khan mein aana aur aap ka naam likhungi

SS: acha

NN: phir us k baad wahin pe hamara card ban jaata hai phir who hamein deite hain aur agar hum wahan pen a pohanch sakte toh un ko hum apna msg deite toh who aage tak pohcha deite hain gaari aati hai hamein le k jaati hai

SS: thek hai acha yeh mujhe bataayein k aur yeh kitne kitne arsey baad aati hain aap k paas

NN: yeh toh musalsal aati rehti hain

SS: aati rehti hain matlab ek maheeney do maheeney hafte baad

NN: haftey baad jab bacha mera chota hota hai hafte hafte aati thi bacha jaise hi bara hota jaata hai phir who usi time k hisaab se aati hain

SS: aati hai acha aur aap ko kaisa mehsoos hota hai in k kaam k hawale se joh yeh kaam kar rahi hain

NN: kaam hamein acha lagta hai yani k aise ikhraajaat hote hain toh agar is mein hamara naam darj hota hai toh agar bacha bimaar hojaata hai toh un k support hum jaate hain ya phir delivery k doraan tabiat kharaab hojaati hai phone karte hain toh gaari aati hai toh hamein le k jaati hai sahi mehsoos hota hai

SS: aap ko acha lagta hai jab aap hamal se hoti hain aap phone karti hain gaari ajaati hai aap ko le k chali jaati hai toh yeh lagta hai joh worker aap k ghar aati hain in ki wajha se aap ko sahulat hoti hai

NN: jee haan batatey hain hum apne masaail aage who batatey hain aur worker hamein apne area k aura cha lagta hai

SS: k aap k area ki worker hai aap unse baat kar sakti hain

NN: baat kar k apne masaail bata sakte hain

SS: acha masaail hote kia kia hain

NN: masaail yani bache achanak se bimaar hojaate hain yani k ghar mein deakhna parta hai toh husband hota hai us ka bhi khayal rakhna parta hai toh agar paidal jao toh wahin pe time lag jaata hai toh phir in ko agar bolte hain toh hamein gaari waari mein bhi le k jaate hain

SS: acha aur time pe ajaati hain gaari

NN: time pe waise toh pehle aaya karti thi bohat arsa hogaya mein nahi jaati hun pehle toh time se aaya karti thi

SS: pehle time se aaya karti thi yeh joh pehle hai toh kitna pehle tha

NN: ab mera bacha yani bara hogaya hai na aur mein khud bhi apne hisaab se nahi jaati hun un ka mein kyun jab hum unhein kaha karte thy na abhi bhi mere bache k hifazati teeke rehte toh who larkiyan aayin thi gaari bhi le k aayi thin aur mein khud se gayi nahi time nahi tha

SS: kia wajha time kis wajha

NN: wajha is liye mere shouhar berozgaar hogaye thy toh bache ko deine k liye is liye mein ne raat bhar machine pe khayal rakhti thi bache k hifazati teeke k liye nahi gayi who aayi thi gaari aayegi aap tayar ho k rehna par mein gayi nahi

SS: phir bhi nahi gayi toh hifazati teeke toh paise se nahi lagte

NN: paise se nahi mein apne kaam mein masroof thi

SS: itna sab hi time nahi tha k bache k hifazati teeke laga leiti acha yeh mujhe bataaein k koi aisi cheez hai jab jaise worker aati hain toh aap ne kaha k acha lagta hai koi aisi baat joh aap ko achi nahi lagti ho ya aap un ko badalna chahti hon

NN: badalna mujhe sirf yeh acha nahi lagta yani jab paise hote hain na koi bhi aurat paise hote hain har kisi ko giza ki zaroorat parta hai chahe sehatmand ho ya kamzor phir jab us k pait mein bacha chala jaata hai toh us ko bhi usi hisaab se apne bache ka khayal who sirf mujhe yeh baat achi nahi lagti who kahe aap ka baazu sahi hai aap ko koi giza ki cheez nahi milti yeh mujhe acha nahi lagta

SS: acha toh k yeh cheezein aap ko jab baazu se check karti hain sehatmand hoti hain keh deity hain toh aap ko cheez milti nahi hai

NN: haan yeh mujhe acha nahi lagta

SS: warna who kia deity hain

NN: yani jab pait k doraan toh kisi ko kamzori par jaati hai toh pehle toh biscuit wagera diya karte thy packet wagera deite toh har pregnancy mein har kisi ko zaroorat par jaati hai giza leine ki

SS: hmm

NN: yeh mujhe acha nahi lagta

SS: k kisi ko deite hain agar koi sehatmand nikalti hai toh us ko nahi deite

NN: us ko nahi deite

SS: leikin aap ka yeh khayal hai k har aurat ko

NN: har aurat ko pregnancy mein us ko sehat ki zaroorat hoti hai

SS: hmm

NN: mein agar kitni bhi sehat mein hun na toh bacha jab andar aayega us ko bhi sehat ki zaroorat paregi bache ko yeh mujhe acha nahi lagta hai

SS: phir acha who aap ko nahi deite hain toh phir who aap se kia kehti hain

NN: who kuch kehti nahi hain who bas yani hamara checkup karte hain test wagera karte hain toh yeh batate hain k aap ko is cheez ki zaroorat nahi

SS: phir us k elawa kuch nahi batati yeh toh biscuit ki zaroorat nahi ya joh who packet hai us ki zaroorat nahi hai us k elawa who batati hain

NN: who batati hain kehti hain aap dood leina yani seib wagera leina

SS: hmm

NN: achi koi giza kha leina

SS: han

NN: yeh batate hain

SS: toh phir aap leiti hain

NN: agar hamare afford mein hota hai toh leite hain agar nahi hota toh hum nahi leite

SS: acha jaise k aap ka bacha 17 maheeney ka hai toh joh aap ki aakhari hamal se jab aap huyi thi th us mein workers ne koi kirdaar ada kiya tha

NN: han yani mujhe BP ka masla hota hai who hamal mein khaas se khayal rakhti thi aur gaari bar bar aati thi k aap ka BP barha hai ya nahi barha hai yani khayal rakhte thy

SS: khayal rakha tha us mein unhon ne k BP ki wajha se acha toh us mein aap ko who le kar jaati thin ya aap ka bloodpressure a k check karti thi

NN: nahi jab date diya karte thy na mein apne time se jaya karti thi wahin pe jis tareekh ko date diya karti thi phir who aage muaaina karte thy jahan pe worker ki larkiyan beithti hai na

SS: wahan pe who muaaina karti thi

NN: jee han

SS: aap khud se jaati thi ya CHW aap k ghar aati thi toh us se koi faida hota tha us k zariye aap wahan tak jaati thi

NN: yani yeh toh form bhar k jaate thy na phir us k baad hamara card ban jaata tha

SS: hmm

NN: card ban jaata tha phir who hamein aage tak date deity thi

SS: hmm

NN: yani larkiyan toh apne time k hisaab se aayingi na toh zahir si baat hai hamara bhi bacha hai hamara bhi haq banta hai sara din hum un k intezar mein toh nahi beitheinge na

SS: hmm

NN: toh hum apne bache k liye hum khud apne time se chale jaate par wapsi mein who gaari deite thy hum aate thy

SS: acha aur bache k hawale se kia kirdaar ada kiya tha CHW ne

NN: yani bache k hawale se bohat acha karte hain bacha paida hota hai toh who wahin pe bhi bache ka check wagera karte bache ko khuda na khaasta koi beemari weemari hoti hai toh who wahin pe kisi aur hospital mein rakhwaate hain mere bache k sath kabhi aisa masla hua nahi hai mere khud ka bacha Alhamdulliah sahi paida hua

SS: bacha sahi hua toh us k baad unhon ne kia kiya

NN: who hamein hifazati teeke paidaishi teeka pehla lagt hai usi k liye bhi le k jaati thi

SS: hmm

NN: aur baar baar aaya karte thy bacha chota hota hai toh phir regularly aaya karti thi hafte mein 4 din baad bache ko bukhaar hai ya bache ka seena bache ko dood k elawa 6 maheeney tak kuch na deina yeh sab batati thi

SS: yeh sab baatein batati thi aur a k checkup karti thi

NN: private hospital mein jaise jaate hain

SS: hmm

NN: aap ko aisa lagta hai hamare bache ko powder milta hai woh powder aap k bache k sehatmand nahi agar bimaari hogi khatregi toh hum hi aap ko bataaeinge aap k bache ko khatra pohanchne wala hai

SS: hmm thek hai acha yeh mujhe bataein k yeh toh k matlab who aap ki madad karti hain yeh saari cheezein aap ko deity hain who aap ko ghar par pohchaati bhi hain koi aisi cheez joh aap ko lagta hai k jaise CHWs hain acha mujhe bataaein k yeh saari cheezein who karti hain na is k elawa aap ko lagta hai un ko aur maaloomaat deine ki zaroorat hai bache k hawale se hamla khatoon k hawale se jis se aap ko faida

NN: yani jaise pait k doraan saare hamein batatey hain faide ki cheezein k aap ko khuda na khaasta qamar mein dard ho ya paani wagera chute aisa nahi k aap 9 maheeney tak intezaar karo yani khatre ki cheezein aap ko bata k jaate hain toh woh baaki aage tak mujhe pata nahi

SS: bas yeh bata k jaati hain aur is k elawa kuch aur milta hai

NN: aur bache k bache ko agar jhatka lage toh aap yani hath pe hath rak k na beitho phone karo uzma baaji ko k aap ko le k jaaye kahin ya a k bache ko dikhaayein

SS: hmm

NN: aisi yani khatre k liye bata k jaate hain hamari faide ki cheezein toh phir hamein bhi karni parti hai

SS: hmm matlab aap yeh keh rahi ho k who aap ko yeh bata k jaati hain k aap joh hai who aa agar bache ko kuch ho toh phone karein center par le kar aayein ghar pen a beithi rahein hamal k doraan aap ko batati hain acha hamal k doraan aap ne giza k baare mein bataya tha who aap ko batati hain thek hai is k elawa aur koi cheez batati hain

NN: han kehti hain time se khaana khana yani jab larkiyan aate hain toh yeh batate hain yani ab aap ek roti kha rahe ho toh aap abhi hamal se ho aap ko do ki zaroorat hai

SS: hmm

NN: aise saari cheezon k baare mein bata k jaati hain

SS: acha mujhe yeh bataaein k joh teams aati hain joh yeh workers aati hain yeh kitni aati hain matlab do larkiyan aati hain

NN: do larkiyan aati hain

SS: do larkiyan aati hain aur yeh ek hi tarhan k sawal karti hain ya alag alag kisam k kuch aur a k do do team ek hi larkiyon k do team aati hain ya alag alag do do team

NN: alag alag ek hi who larkiyan aati hain

SS: wohi larkiyan aati hain aur who kis hawale se sawal karti hain joh leiti hain kaam kar rahi hoti hain toh who aap se poochti kia kia hain

NN: agar pait k doraan pregnancy k doraan pait k hi sawal karti hain agar bacha paida hojaaata hai yeh sawal karti hain aap k bache ko bukhaar hua hai ya us ko motion huye hain ya us ko ulti lag gayi hai is bachon k baare mein sawal karte hain

SS: acha nahi lekin is k elawa aap k matlab pregnancy wagera pregnant hogaye toh poocheinge agar pregnant nahi ho toh phir kia karti hain

NN: phir toh bache k baare mein poochti hain kehti hain k aap acha sa logi yani bache ko dood pilaane mein aap se bache ki madad hogi 6 maheeno tak agar aap ache khaana toh waise acha khaana chaiye

SS: hmm

NN: par yani bacha chota hai 6 maheeno tak aap yani sooji lo yeh lo toh aap ko dood bache ko deine mein

SS: hmm

NN: sahi lagogi

SS: aap ko lagta hai k joh bhi who bataati hain ye kaafi ai aap k liye ya mazeed aur kuch karna chaiye is ko behtar bananey k liye

NN: mujhe toh yeh bhi acha lagta hai aage ka toh mujhe pata nahi

SS: joh aap ko who batati hain who aap ko acha lagta hai

NN: han

SS: acha aur in ki joh worker aati hain aur aisi kia baat hain joh aap ko lagta hai k yeh achi hai yeh honi chaiye worker k andar

NN: worker aate hain toh hamein yeh acha lagta hai who time se ajaati hain phir hum apna bacha le k chale jaate hain ya hamein date bataati hain date ka hamein bhi pata hai k hamein kis time pe date phir who bataaeinge agle bache ki muaaina karne ki tareekh is tareekh pe jana phir hum jaate hain

SS: acha yeh aap k liye achi baat hai who a k batati hain aur aap ne ek baat yeh bhi kahi thi k who aap k apne elaake ki hain

NN: hm yani unse baat cheet karne mein sahi lagta hai

SS: aur agar aap k elaake ki na hon

NN: phir bhi ache hoti hain

SS: haan

NN: aksar aise hota hai ek hamare area ki larki aati hai rehri goth ki aur ek bahar ki larki aati hai

SS: hmm toh who thek rehta hai

NN: yani kisi ko urdu aati hai kisi ko nahi aati hai sab ko toh urdu bhi nahi aati hai yani hamare area k hote hain toh batane mein kisi ko masla nahi hota hai na phir aisa hai k mere bache ko yeh hai

SS: hmm toh aap ko matlab aitebaar hai un pe jin ko aap jaanti hai

NN: jee

SS: agar kisi aur area se aayeingi toh aap us ko bata deingi

NN: bata dungi

SS: bata deingi koi matlab us mein masla nahi hai

NN: nahi nahi

SS: acha .. acha mujhe yeh bataaein k jaise joh hamari workers hain who aap k ghar aati hain aur hamara center bhi hai na toh aap ko kia lagta hai k is mein un ka kia kirdaar hai joh unhon ne aap ko center se joura hai hamara idaara hai sehat ka ya dusre idaaron se journe mein aap kaisa mehsoos karti hain is mein in ka kia kirdaar hai

NN: kis cheez mein

SS: matlab yeh joh hamara center hai jis pe aap jaate ho mujhe yeh poochna hai k jaise joh hamara center hai is k baare mein pehle toh nahi pata hoga na thek hai ya aap ko pehle se hi pata tha k yahan par joh hai aga khan ka ek joh hai joh sehat k hawale se ek center hai yahan par yeh yeh cheezein di jaati hain toh is mein aap ko worker ne bataya us mein kia madad haasil huyi aap ko

NN: yani pehle hum jab chote chote hua karte thy toh aga khan toh pehle mujood tha

SS: acha

NN: jab hum bhi chote hua karte thy yani vital ki taraf se abhi pata chala hamari shadi hone k baad jab hum pregnant huye phir hamein vital walon ki taraf se pata chala k pregnancy k doraan yeh hamare aurat ko le k jaate hain khuda na khaasta khoon chut jaaye yani pehle toh kuch aise log hua karte thy joh kehte thy pehle bhi khuda ka nizaam chalta tha na magar aage se in workeron ne bataya toh bohat se logon ko samjh agai

SS: hmm

NN: k yani yeh baat sahi hai k mard ghar mein hote hain ya kabhi aise ikhraajaat bhi hojaate hain k delivery agar hospital ki bhi agar ghar mein karwaani par jaaye toh yani nuksaan hojaata tha toh yeh toh masla nahi hai na kisi bhi time dard aaye toh phone karo toh gaari ajaati hai

SS: hmm toh yeh matlab is mein worker ka kia kirdaar hai matlab worker ne kia kaam kiya hai is mein

NN: worker ka yeh kirdaar hai yani kuch aurat aisi bhi hoti hain joh date ka intezar karti hain worker agar aate hain deakhte hain patient ko zaroorat hai center jaane ki hospital toh who khud se hi le kar jaate hain

SS: aur agar un ki delivery kareeb arahi hoti hai acha toh who center pe le k chali jaati hain worker ka kirdaar hain k who center tak le k chali jaati hain aur center pe jab aap log jaate hain toh aap khush hain wahan par joh sahuliyaat milti hai

NN: mein toh bohat se log honge na

SS: hmm

NN: un ko achi response milti hai kuch ko na milti hai who toh har kisi ka apna apna masla hota hai na yani agar mujhe ache rawaiye se pesh aayein sab ka toh mein zimma nahi le sakti na kuch aise hain jin ko pata nahi bichaare ko nahi hota un k sath acha rawaiya na ho who toh un ko hi pata hoga na

SS: haan leikin aap ka kaisa raha matlab aap apne baare mein bataaein jab aap center pe gayin aap ko doctor ne sahi tarhaan se deakha aap k bache ko sahi se deakha mein aap k baare mein poochna chah rahi hun

NN: mere baare mein toh who sahi batate hain agar khuda na khaasta bache ko pait k doraan hi koi khatra hota hai toh bata deite hain ya phir patient ko toh bata deite hain aage se le kar bhi jaate hain

SS: thek hai acha mujhe yeh bataaein abhi tak toh bari achi achi baatein ki aisa matlab mein aisi kahongi k hamara joh bhi kaam hai who bohat acha chal raha hai koi aisi cheez joh aap ko achi na lagti ho ya hamein un ko behtar bananey hum is kaam karne ka maqsad ye hai k hum is community mein kaam ko aur behtar kaisa banaaein

NN: mujhe aisa acha nahi lagta mein ne aap ko pehle bhi bataya kuch aurtein hamare yahan bohat saari anparh hai joh center mein shikaayat nahi laga sakti humse laga sakti hain yani hamein batati hain k hum isi liye center nahi jaate k yani aage wahan pe jaate hain toh aage yani patient ko patient kar k nahi deakha jaata agar hamare bachon ko sehat ki cheezon ki zaroorat hoti hai toh who nahi di jaati aap logon k sath acha rawaiya hai kyun k hamein bura lagta hai patient sab patient hote hain yani joh kaam karte hain un ki duty hai na

SS: hmm hmm hmm

NN: toh log darte hain k kahin hum un ki shikaayat aise log hain hum toh nahi darte hain hum ne aage duniyan deakhi huyi hai hum ne NGOs deakhe huye hain zindagi maut khuda k hath mein hain koi NGOS walon ki taraf se who kia kareinge ziada se ziada patient ko le kar nahi jaayeinge na kyun nahi le k jaayeinge zahir si baat hai in ka kaam hai le kar hi jaayeinge na toh bohat si aurtein shikaayatein bhi karti hain

SS: who kia kehti hain

NN: who kehte hain k hamare bache yani hum batate hain joh masaail who masaail hamare hal nahi hote par hum aage tak kehte hain un ka khud ka masla hai agar hamare paas kuch chahein toh hum khud ja k bataein worker ko hum worker ka koi masla nahi hota worker kehte hain hamara kaam hai le kar jana aage toh joh aap k

SS: acha toh workers ka kia rawaiya kabhi aap ne suna k jaise center wali aurat ne bataya ho

NN: yahan pe joh worker aati hain wohi center pe joh worker hain

SS: joh yahan aati hain ghar pe aati hain

NN: who toh bohat ache rawaiye se pesh aate hain

SS: acha toh un mein se kabhi aisa hua k worker ne kisi se sahi tarhan se baat na ki ho aap k sunne mein aaya ho

NN: nahi nahi

SS: is tarhan se nahi

NN: haan

SS: k worker yahan sahi hote hain jab center pe jaate hain toh log thora nakhush hojaate hain

NN: han

SS: thek hai aur phir jab wapis worker jaati hain toh center pe bulaane k liye

NN: toh aurtein workeron ko sunatein hain workeron ka yani kasoor hi nahi hota hai

SS: hmm hmm. Yeh bari aap ne achi baat bataai k workers k sath koi nahi hota jab center pe jaate hain joh kuch aurtein aisi hain joh nakhush hoti hain k bacha hai us ko sahi tarhan se nahi deakha gaya masla tha

NN: han who masla hal nahi hua

SS: hmm hal nahi hua phir aap log kia karte ho phir aap bache ko

NN: bohat gussa aata hai k aage shikaayat laga dein hamein unse koi leina deina nahi hai

SS: thek hai

NN: hamare rishtedaari hi toot jaayegi yani unka kaam hai apna kaam kare na

SS: hmm hmm hmm

NN: apni duty nibhaaye

SS: hmm

NN: aise masaail bohat si aurtein pesh karti hain

SS: acha phir shikaayat lagane k liye kia hota hai k shikaayat kaise

NN: who tab hum shikaayat laga deite hain hum bolte hain hamare paas time hoga hum aap ki shikaayat laga deinge par hum time bhi nahi hota hai aur kehte hain hosakta hai idaare mein aamne saamne toh rubaru na thy kaise hum

SS: matlab aapas mein baat karte hain lekin aap logon ko yeh nahi pata k kaise baat ja k karni kis se haiagar koi masla araha hai

NN: han shikaayat kis se lagaaein

SS: hm acha yeh aap logon ko nahi pata

NN: nahi pata

SS: thek hai acha yeh bataein k kabhi aap ne yeh mehsoos kiya hai aap worker ko apni maloomaat deina chah rahi hain batana chah rahi hain lekin koi rukaawat hai aap nahi bata saki koi bhi wajha hai kisi wajha se aap ne na bataai ho jaise aap ne kaha tha na k le kar toh aayi hifazati teekon k liye le kar ja nahi saki k mein apne kaam ki wajha se masroof thi toh is tarhan ki koi cheez hai joh maloomaat deina chah rahi hon worker aayi hon who apna visit karti hain regular lekin aap ne us ko maloomaat nahi di hon kia wajha hai

NN: aise bohat si aurtein yahan pe thy mere elawa mein nahi mein khud ko toh nahi aise thy k center walon ki wajha se hi bachon ka elaaj nahi karwaate thy kehte hain hamein yahan kehte hain k elaaj k liye bacha le kar aao aur wahan pe jaate hain toh hum patient kar k deakhti tak nahi hain k hum un k kia patient hain

SS: toh isliye maloomaat nai deity hain aurtein

NN: haan isi gusse ki wajha se bhi nahi deity phir hum unhen samjhaate k yani aap ka bhi faida hai aap ka khud ka bhi bacha hai k aap yani apne se bhi yani who bhi naraz hojaati hain kehte hain hamein baat karne nahi aati phir hum pe akkar jaati hain k agar bacha ziada rou jaaye phir bhi kehti hain aap toh jaahil ho aap ko yeh pata nahi bacha rou bhi jaata hai

SS: hmm

NN: toh un ka kaam hai ache rawaiyon se patienton se pesh aana

SS: hmm toh aap ko lagta hai k ek joh CHWs ko agar deini hoti hai maloomaat toh aurtein isliye nahi deity k jab center pe jaayeingi toh koi faida nahi hoga sunani jaayegi baat gussa kareinge hum pe toh hum ne deina hi nahi hai aur is k elawa koi lagta hai k aap k elaake ki nahon koi aur masla hon is ki wajha se

NN: elaake ki agar na bhi hon phir bhi abhi aayi hai elaake ki pehle elaake ki nahi hua karti thi who bhi acha kaam karti worker hamesha ache hote hain

SS: hmm

NN: joh bhi yahan pe aate hain na patienton k sath kabhi unhon ne galat rawaaiya nahi kiya agar le kar bhi jaate hain agar wahan pe workeron ka din guzar jaata hai gaari k liye toh worker aage ja k baat bhi karte hain k yeh kab se beithi huyi hain un ko gaari do

SS: aur ghar walon ki taraf se kabhi aisa hua k aap jana toh chah rahi hon par aap k joh ghar waale hain who manah kar rahe hon

NN: ghar wale toh aage toh aisa kuch nahi karna chahte par kuch yani who kehte hain agar aap jana chahte ho toh aap k bache ka joh masla hai agar aap ka hal nahi hota toh kyun jaati ho us se phir ghar waale bhi gussa karte hain zahir si baat hai wahan se jab thak k aate hain toh batatey hain k hamara kaam nahi hua toh who gussa karte hain kehte hain phir nahi jao na kisi mehnat kar k private hospital chale jao aap jaati ho aura a jaati ho kehti ho k hamara kaam nahi hua isi liye ghar wale bhi gussa karte hain ek ko deakh k dusra bhi manah karta hai k deakho falah gayi thi us k bache ka elaaj nahi hua who aayi tum bhi nahi jao

SS: hmm yeh toh bari aap ne baat bataai k ghar wale thora is baat pe bhi manah karte hain k jab aap ka kaam nahi hota pura din guzaar kar ajaate hain toh is wajha se kehte hain koi aisi baat suni ho kabhi kisi se toh ghar walon ne manah kar diya aap k shohar ne manah kar diya ho k nahi bhae in ko nahi batana joh worker aayegi us ko koi cheez bataane ki zaroorat nahi hai

NN: nahi nahi aisa nahi hua

SS: aisa kabhi nahi hua thek hai acha yeh mujhe bataaein k hum aap k hawale se CHW k kaam ko kaise behtar kar sakte hain kia aisa karein

NN: aisa behtar sab se acha yeh k patienton k sath acha rawaaiya karein un ka dil bhare k ek se dusre tak who khabar pohchaaye k center mein acha kaam chal raha hai aap dus aage nahi hai dus wala doctor aap ne suna khatija hospital hai sindhi mein hamare yahan aurat aksar dus wala doctor kaha karti thi kehte hain wahin par agar jaate hain hamein yani who private hai zahir si baat hai rawaaiya acha kareinge na toh phir wahin pe chali jaati hain yani ek se dusri khabar pohchaaye yahan pe sara din kyun beithi ho wahin pe chali jao

SS: who joh aap ne dus number wala doctor bataya khatija wala aaa who paise leite hain

NN: mein toh kabhi nahi gayi par yani dusri aurton k sath suna hai k yani who us ki dus rupiya fees hai tabhi toh us ka dus wala naam rakha hai

NN: (laughing)

SS: achaaa

NN: yani itni fees leita hai dus bees itni toh wahan ki joh dawaiyan wagera mere khayal se free mein deita hai

SS: acha toh who aap yeh keh rahi hain behtar is tarhan banaya jaa sakta hai CHW toh kaam karti hain lekin center mein jaayein aurtein toh un ka acha rawaaiya rakhein

NN: acha rawaaiya yani patient ko patient kar k dekha jaaye agar bacha ro raha hai toh us mein ma ka who joh karni maa hai pehle koshish kon maa kaheigi k mera bacha roye ya 5 aurton mein tang kare toh us pe dusre agar gussa kare toh bura lagega na 5 aurton k saamne ek ko daanta jaaye toh usay bura lagega na toh us ko jaane k liye dil nahi karega na patienton k sath acha rawaaiya kiya jaaye

SS: hmm is k elawa ek shuru mein aap ne baat ki thi mujhe abhi yaad aayi joh pregnant women hain who

NN: hosakta hai upar se sehatmand andar se kamzor ho

SS: hmm toh us k liye kia kiya jaaye

NN: k pregnancy mein mera khayal hai aksar aurtein humse yehi shikaayat karti hain hum jaate hain saara din falah ko deakho us ko zaroorat hai who bhi toh meri jitni sehatmand hai hosakta hai un k khayal mein baat na aati ho who hamein dood ka kehti hain hamare paas ddod hota toh hum un k paas kyun jaate

SS: hmm hmm hmm

NN: aise aurtein hamein baatein batati hain

SS: k who aap ko keh deite hain bol kar

NN: who kehte hain hum achi achi baatein batate hain saib khaao apni sehat ka khayal rakho yeh sab kuch who batate hain peeli goliyan deite hain kehte hain peeli goliyan khaao aur jaise aap ko do maheeney teen maheeney lag jaaye k a k apna checkup karwao

SS: hmm hmm toh lekin aap yeh keh rahi hain k hamare paas yeh sab cheezein hotin toh un k paas kyun jaate

NN: haaan har kisi ki apni apni soch hoti hai yani mein samjh sakti hun hosakta hai dusri na samjhe baat ko

SS: us ki kia wajha hogi aap kyun samjh sakti hain

NN: mein ne yani

SS: thora parha hua hai

NN: thora parha hua hai toh kabhi hansi bhi a jaati hai yani kabhi socha bhi jaata hai k yani who kehte hain agar hum sehat k liye toh sab ko pregnancy k doraan sab ko sehat ki zaroorat parti hai

SS: hmm

NN: toh sab ko deini chaiye agar un ko bahar se 28:18-28:19 horahi hai agar deini chaiye nahi samjhti hai isi wajha se apna center chor k beithi huyi hai toh sab ko ek hi nazar se deakha jaaye toh hosakta hai upar se sehatmand ho andar se who kamzor ho

SS: andar se who kamzor ho acha thek hai is mein matlab who jaise unhon ne kaha k yeh packet sab ko nahi deite aur un ko us se hath ko check kar k kehte hain k nahi aap ko zaroorat hai who bhi un ka bhi hota hogana is ko deina hai is ko nahi deina

NN: haan who toh sahi who toh apne hisaab se sahi honge par yeh aurtein nahi samjhti

SS: kia wajha hai who kyun nahi samjhti hain aap k khayal se

NN: mere khayal se toh ek parhi likhi nahi hai

SS: hmm

NN: yani k kuch baat ko samjhte nahi hain k jitni aap packet se sehat maang rahe ho utni achi dood se sehat milegi

SS: hmm hmm

NN: par who nahi samjht hain is baat ko gusse ki wajha se who apna naam nahi likhwaati itna bara nuksaan kar deity hain kehti hain delivery honi hogi toh hamare ghar mein bhi hojaayegi toh who dr suraiya kitna leigi 3000 leigi hamari delivery hojaayegi aise gussa karti hai

SS: hmm hmm hmm acha toh ek toh aap ne kaha k who packet nahi deite dusra un ka rawaaiya sahi nahi hota jis ki wajha se CHW k kaam k upar bhi asar parta hai jab CHW dobara ghar par aati hain toh phir log manah kar deite thy k hum ne naam nahi likhwaana thek hai yeh waali baat acha aur aap ne kaha k thora sa parhi likhi nahi hoti hain aurtein

NN: parhe likhe ache hosakta hai in ka kuch kasoor ho zahir si baat hai saara toh un ka bhi nahi hoga na yeh bhi anparh hai par zahir si baat hai jab insaan apne aap ko deakhega tabhi who duniyaan ko jaanega na zahir si baat hai gaaon se aayi huyi hain yahin pe beithi huyi hain ya gutka wagera kha rahi hain toh in ka bhi kasoor hota hai yani kisi dustbin mein hosakta hai un ko maloom na ho aur kahin bhi un ko achae rawaaiye se bataya jaaye yani unse akkar k baat ek na samjh insaan us se aur nadaani karoge toh who toh bas kia karega khud ghar mein beith jaata hai aur chaar baatein aur laga deiga who toh khuda jaane

SS: thek hai yeh joh aap ne kaha k un ko araam se bataya jaaye un ko yeh bataya jaaye k bhae aap jaise koi gutka kha rahe ho us ko dustbin mein pheko joh bhi choti moti baat toh yeh hum agar CHW k through karwaayein un ko yeh maloomaat farhaam karein aap k khayal mein who samjh jaayeingi

NN: jee han kyun nahi insan hain kyun nahi samjh jab mein samjh sakti hun baatein jab un ka dil mera bharosa deita hai k hum NN ko yeh baat bataaeinge k NN hamein bataeigi pyar se mein agar gusse se kon us ko bataaegi us ne toh hamein daant diya sharam toh sab ko aayegi chahe who anparh ho chahe jaahil sharam toh sab ko aati hai

SS: hmm toh worker k through who bhi hum ache se baat karein achi tarhan se maloomaat farhaam karein toh kaam ko behtar bana sakte hain

NN: jee haan aur hamein yeh maloomaat sab se laazim mujhe karni mere paas agar time hota toh men khusd center mein kaam karti mujhe yani mein yeh chahti hun hamare maashirey mein aisi aisi aurtein hain joh apne masaail ghar walon ki wajha se ya aage ki wajha se nahi bata sakti k mera dil karta hai yani agr mere ghar k halaat sahi hote toh mein khud agar salary k hisaab se na karti mein apne dil k shouk se kar leiti k kisi ko meri wajha se faida pohche par mein khud ghar pe masroof hun na

SS: hmm hmm

NN: toh bohat si aurtein aati hain yani shikaayatein karti hain

SS; kia shikaayatein karti hain

NN: yehi shikaayatein joh mein ne aap ko bataai k yani pregnancy mein yani poore jism mein dard aate hain yani phir wahan jaate hain toh bolte hain kuch bhi nahi hai

SS: hmm

NN: aisi aisi yani

SS: toh yeh CHWs ko batati hain jab un k ghar pe aati hain k bhae yeh yeh hamein dard uth raha hai yeh masla horaha hai

NN: hosakta ho batati ho

SS: aap ne kabhi unse poocha

NN: mein ne kabhi poocha nahi par aap ne cheezein bohat si bataai hai k hum kuch aise bhi log hote hain dar ki wajha se bhi nahi batate hain hum bata deinge toh kahin naam na kat jaaye aise bhi hota hai na toh hosakta hai aage se mein maloomaat lungi aur mujhe is baat ka laazim khayal rakhna hai k shikaayat kis se karni hai toh aage kon masla hal karta hai yeh mujhe laazim baat ka pata karna hai

SS: matlab hum aap shikaayat aap ko lagta hai aap kisi worker ko batao apna masla toh who hal ho jaayega

NN: nahi nahi worker toh kehte hain hamara kaam nahi hai yahan se le kar jaana aage ka kaam hamara nahi hai

SS: toh worker aap ka masla hal nahi kar sakti aap ko sirf center tak pohcha sakti hain

NN: jee han jee han

SS: toh is baat pe aap ko kaisa lagta hai toh worker aap ko yeh keh deity hain k mein toh center tak aap ko pohcha sakti hun

NN: aage toh hamara kaam nahi hai

SS: phir kia aap ko lagta hai k agli dafa aayegi toh mein us ko apni baat bataongi

NN: who kaheingi mein apna naam katwa dungi mein nahi jaongi yehi kehti hain aurtein

SS: acha toh is baat pe workers jab keh aap ko jawab deity hain k hum bas yahan tak aap ki madad kar sakte hain

NN: han yahan tak kehti hain aage jaao uzma baaji se baat karo

SS: acha toh who yeh aap ko kehti hain k is se baat karo thek hai phir masla hal hota hai

NN: mein toh khud ka karti hun mera toh masla hal hojaata hai par kuch aurtein joh nahi baat kar sakti un ka nahi hota hai

SS: ek baat aap ne aur kahi thi ghar k bhi kuch masaail hote hain toh ghar k kia masaail hote hain kia aap ko lagta hai k ghar ki wajha se kia aurton pe masle hote hain jis ki wajha se center nahi ja sakti CHWs se baat nahi kar sakti

NN: yeh mein ne aap ko bataya k agar koi aurat aati hai wahan pe elaaj wagera na hua toh ghar ka masla barh jaata hai hai agar mard sunte hain agar nahi hota hai toh kyun aage jaati ho

SS: hmm hmm hmm thek hai aur aur mein yeh chah rahi hun worker k through hum masle wagera hal karein ya is tarhan ki kuch mashwara de sakti hain hamein k kis tarhan se worker k zariye hum community ko behtar bana sakein

NN: mein ne aap ko pehle bataya na k masla sab se pehle is baat pe k worker patienton k sath acha rawaaiya kare wahan pe le k jaate hain toh wahin pe bhi workeron ko yani k aap ko yahin jana hai yahan jana hai worker toh kuch aise hain k aap se poochne in se poochna kahan jana hai kis jagha dawai leini hai isay nahi pata hota hai toh workeron ka kaam hai agar le k jaate hain toh aage bhi un ka khayal karein

SS: matlab aap ko center tak chor na dein aage bhi aap ki madad karein

NN: han

SS: thek hai sahi acha aur is ko behtar bananey mein kuch aur kehna chahogi CHW k kirdaar ko kaise behtar bana sakein

NN: kirdaar toh sab se toh mein khud se khayal se k kirdaar patienton k sath acha rawaaiya kiya jaaye k un ka dil barhe

SS: hmm

NN: k who shikaayat na kare who pagal nahi hain joh shikaayat kareinge yani un ko sath mein kuch masla hota hai tabhi aage k ghar mein shikaayat karte hain

SS: hmm hmm center pe thek ho lekin center pe wahan aan hai na thek hai lekin yahan se le kar jana toh hamari worker ka kaam hai sahi hai na k worker CHW ko center pe le kar aayegi toh us ko k jaise agar kis ko gussa a raha hai kisi ko koi masla hai center pe gayi naraz hogayi toh us ko hum worker k through kaise sahi kara sakte hain kis tarhan se behtar kar sakte hain kyun k worker toh hamare elaake ki hoti hai

NN: han worker toh yahan se le kar jaate hain

SS: hmm

NN: aap pregnancy k doraan pooch rahe ho ya bachon

SS: dono bacha dono k hawale se agar bacha beemar hai tab bhi agar haamla khaaton hain tab bhi worker kia aisa kaam kare k who thek hojaaye hamara masla hal hojaaye kyun k hamara maqsad toh yehi hai na k hum aap ko araam dein aap ko shikaayat ka mauqa nahi dein aur yeh hamein kis k zariye se karwaana hai yeh hamein worker k zariye karwana hai is ko kaise behta banaayein

NN: kehte hain pregnancy mein yahan se toh khud se jaate hain wahin se aati hain

SS: hmm hmm

NN: gaari se wahin se le kar yahan se khud worker paidal jaate hain

SS: hmm hmm

NN: pregnancy k doraan bhi aur jab nawaan maheena lag jaata hai hum phone karein toh wahin se gaari aati hai worker le kar aate hain le kar jaate hain ya kisi patient ki tabiat kharaab hojaati hai phone karte hain toh who aajaate hain worker aur patient ko le kar jaate hain

SS: thek thek thek sahi hai chalein shukriya bohat bohat NN aap ka hamein bohat saari baatein bataai inshaAllah hum in ko joh hai

NN: aap ka shukriya aap ne time diya par yeh aap se request karni thi k aage tak shikaayat kis tak pohcha sakte hain ……….

Date: 27-01-2021

RA: acha NN aap ki umar kitni hai

NN: 33 saal

RA: 33 saal aur aap ne taleem kahan tak haasil ki hai

NN: 3 classes parhi hun

RA: 3 classes parhi hain

NN: hmm

RA: aur aap k kitne bache hain

NN: mashallah chaar bache hain mere

RA: chaar bache hain

NN: haan 3 zaaya hochuke hain

RA: 3 zaaya hochuke hain

NN: aur chaar mere paas hain

RA: chaar hain aap k paas

NN: hmm

RA: sahi hai acha NN mujhe bataaiyega k aap k paas joh health worker aati hain hamari thek hai kia who baqaaidgi se ghar aati hain

NN: nahi aati hain jab phone karo toh gaari leine k liye aati hai waise nahi aati hain hamare paas

RA: waise nahi aati

NN: waise nahi aati

RA: acha toh yeh aap ko kaisa laga matlab kab kab aur kitne kitne arsey mein aati hain aur kab kab aati hain

NN: jab gaari mein phone karo toh aati hai jab waise koi bhi nahi aata yahan pe

RA: waise koi bhi nahi aata

NN: koi nahi aata

RA: acha jab aati hain toh gaari k hawale se aati hain

NN: haan gaari k hawale se aati hain le k jaati hain aur waise chor k jaati hai who

RA: hmm

NN: waise nahi aati mere paas

RA: kuch aap se a k maloomaat nahi leiti

NN: nahi koi maloomaat nahi leiti is baar koi maloomaat nahi mein ne un k paas call bhi ki toh phir maloomaat nahi horahi

RA: acha aur kitna arsa hua hai koi CHW aap k ghar nahi arahi hai

NN: yeh pehle bache mein toh aayi thi is bache mein nahi arahi hai

RA: acha sirf bache k hawale se ya

NN: waise nahi aati bacha bhi hojaaye toh phir bhi nahi aati hain jab call karo toh aate hain gaari le k warna nahi aati

RA: thek hai acha hamesha jab who aati hain ek jaisi aati hain ya alag alag aati hain

NN: alag alag aati hain

RA: alag alag aati hain kabi koi aisi aayi hai joh aap se sirf aap k hawale se baat karti hon aap se pooche

NN: nahi nahi bilkul bhi nahi

RA: k aap k kitne bache hain

NN: nahi nahi yeh bilkul nahi poochti koi bhi nahi aata

RA: acha thek hai kabhi aisa hua ho k who aayi hon

NN: nahi mein ghar pe nahi hoti hun toh ammi k ghar pe paas pe mere ajaati hain waise ghar pe hoti hun toh ammi bol deity hain k nahi hai ghar pe nahi hai kal ajana kal sham tak ajaayeingi yeh who aise

RA: jaise yahan rehte huye kitna arsa hua hai

NN: mujhe 10 saal hogae

RA: 10 saal hogaye hain in 10 saalon mein aap k paas CHWs nahi aayin

NN: nahi

RA: acha aur aap k kitne bache hain joh zinda hain is waqt

NN: chaar bache hain

RA: chaar bache hain in chaar bachon mein kabhi jab hamal se huyi tab

NN: haan hamal se hoti hun tab bulaati hun toh tab ajaate hain waise poochne k liye who nahi aati

RA: khud se nahi aati

NN: khud se nahi aate mere paas

RA: acha aur jab hamal se hoti hain aap toh us mein who koi aap ko maloomaat wagera kia kaam karti hain us mein jab aap phone kar k bulaati hain toh kia hota hai who kia leiti hain kia information aap se leiti hain

NN: bas jab call karti hun toh bolti hun gaari bhejo dawai leini hai who ajaati hain phir le k wahin pe chor deity hain hum log khud hi wahin pe kuch karte hain phir

RA: acha aap ko koi maloomaat wagera nahi

NN: nahi kuch maloomaat nahi karti isliye bulaya hai kyun kyun bulaaya hai bacha hone wala hai kia masla hai kuch masla hai kuch bhi nahi poochti kuch le k jaati hain mujhe yahan se wahin pe chor deity hain aage phir khud karte hain joh kuch bhi hota hai wahan dawai leini hoti hai toh aage khud jana hota hai

RA: acha aur pichle ek maheeney mein koi aisi aap k paas koi CHW nahi aayi jis mein aap se yeh poocha ho k aap k kitne bache hain

NN: nahi koi bhi nahi aaya aise poochne k liye yeh ek dafa ek martaba poochne k liye aayi bache ko joh hifazati teeke lage hain nahi lage mein ne kaha k mere bachon ka course poora hua hai bas wohi poochne k liye aayi thi yeh do dhaai maheeney pehle aayi thi

RA: hmm

NN: phir us k baad koi nahi aaya mere paas

RA: acha do dhaai maheeney pehle aayi thi

NN: do dhaai maheeney pehle aayi thi

RA: acha toh yeh aap ko kaisa lagta hai k yeh joh CHWs hain jab aap phone karti hain tab aati hain

NN: haan sab aate hain acha hai wahan pe jao toh achi dawai deite hain checkup acha karte hain un ka ikhlaaq bohat acha hai jaise bacha hota hai wahan pea chi tarhan deikhte hain deakhbhaal karte hain

RA: aur joh ghar par aati hain un k hawale se

NN: joh ghar pe aati nahi hai call karo toh aati hain waise warna koi nahi aata

RA: koi nahi aate acha aur aap k hamal k doraan kabhi

NN: jaise kal parso se meri tabiat thek nahi hai mein who pareshan mein ne kaha gaari bahar aati hain in se number le lo ja k toh mein call kar k baat kar lun gaari mangwa lun dawai leine k liye toh abhi tak koi nahi aaya mere paas toh koi bhi nahi aata

RA: acha aap abhi hamal se ho

NN: haan hamal se hun na

RA: acha

NN: isi wajha se hamal se hun na mein kuch khaati hun na toh upar ki tarhan who hoti hai isi wajha se mein ne kuch le k aaon kuch checkup kara k aaon toh phir tabiat thek hojaati hai

RA: hmm hmm

NN: isi wajha se koi aata nahi hai pehle aate thy pehle mere bache huye thy na us mein aate thy jab yeh bacha mera paida hua na toh koi bhi nahi aaya

RA: yeh kitna bara hai

NN: yeh 3 saal ka hai

RA: acha

NN: toh ab koi bhi nahi aata hai mein ne kaha mein uzma baaji ko bolon k mere paas toh koi bhi nahi aata hai call karo toh phir gaari ajaati hai warna koi bhi nahi aati yahan pe

RA: hmm acha yeh mujhe bataaein k jab hamal se yeh toh ek bacha hua aur bache

NN: han mera ek hi bacha hua hai mera

RA: haan hamal k doraan kabhi CHW ne koi a k kirdaar ada kiya aap ki koi madad ki ho

NN: is bache mein aayi thi bari gaari le k aaye thy na

RA: hmm

NN: toh tab blood pressure high hogaya tha na isi wajha se usi doraan le k gaye thy mujhe khud rehri goth mein le k gaye thy gaari mein

RA: hmm hmm toh bas sirf yehi kiya

NN: haan yehi kiya

RA: aur yeh kitni dafa hua kitni baar aayi

NN: ek baar aayi

RA: ek dafa aayi 9 maheeney mein

NN: haan 9 maheeney mein ek dafa aayi jab hi mein call karti hun jab gaari aati hai warna checkup k liye koi bhi nahi aata mere paas

RA: koi bhi nahi aata kabhi koi bache ko wazan naapne

NN: who jab bacha hojaata hai na ek do din k baad aate hain wohi checkup karne k liye ek dafa wohi aate hain

RA: sirf ek dafa

NN: ek dafa aate hain phir

RA: acha regular matlab har maheeney do maheeney baad nahi araha

NN: nahi nahi who nahi aate sirf call karo toh phir aate hain wohi bata rahi hun call karo toh ajaate hain warna koi nahi aata

RA: acha aa toh mein yeh janna chah rahi hun k hamare paas aap ki information toh hai

NN: haan number nikaalna pare mere paas toh nahi koi bhi nahi aaya na mein khud chali gayi neeche mein ne kaha mein toh aati jaati rehti hun na aap naam nikaalo hamara naam bhi hoga beech mein us ne naam nikaala toh wahin pe checkup kiya baazu ka bhi checkup hua na toh un logon ne nahi kiya

RA: center pe jab gayi thi

NN: haan jab center pe neeche gayi toh sab kuch banaaye uzma se baat ki na mein ne kaha mein toh pehle aayi thi mera naam nikaalo hoga beech mein naam nikaala toh naam toh tha beech mein toh un logon ne bohat wait karaya bohat 3 4 ghante wait karaya us k baad ja k unhon ne suna phir

RA: hmm acha yeh mujhe batao k center tak kaise gayi jab call kar k

NN: nahi center mein toh bike pe gayi thi mein

RA: khud gayi thi

NN: khud gayi thi mein wahan pe

RA: acha

NN: number bhi nahi tha gaari ka toh aur jana bhi tha mein ne kaha pata nahi kyun menses wagera arahe hain na

RA: hmm

NN: toh bache ka masla tha isi wajha se jana para wahan pe mein ne kaha khud chali jaati hun number bhi le k ajaaongi kuch masla bhi hoga who log bata deinge k kia masla hai jab kuch hai wagera un logon ne kaha bacha hai isi wajha se un logon ne file bana k di mujhe toh isi doraan 3 4 ghante wahin pe zaaya hogaye

RA: acha toh

NN: acha yeh toh masla nahi hai k 3 4 ghante lag gaye jab call karo toh aate hain warna toh waise checkup k liye koi bhi nahi aate poochne nahi aate tumhari tabiat kaisi hai kaisi nahi hai yeh nahi poochne k liye aate hain

RA: hmm toh aap ko lagta hai k un ko aana chaiye

NN: aana chaiye na aana chaiye pata karna chaiye na un ko patient ki tabiat kaisi hai kaisi nahi hai zaroori toh nahi k insaan k paas kabhi balance hota hai kabhi nahi hota hai isi wajha se in ko pata karna ek do din mein pata karna chaiye yahan pe

RA: hmm hmm acha agar yeh aayeingi aise regular basis pe aap k paas aayeingi aap k hawale se maloomaat leingi kia aap un ko apni maloomaat deingi

NN: jaise aap poochne aaye ho waise poochne k liye

RA: nahi aise nahi jaise k aap k kitne bache hain abhi aap hamal se toh nahi hain

NN: who aati hi nahi hai

RA: nahi aise aayeinge toh phir aap bataaeingi unhein

NN: poocheinge toh bataaeinge phir

RA: aur aap acha mehsoos kareingi is baare mein agar who aap se poochein

NN: haan poochein pata chalega na yeh aage hamare ko jaante hain kuch masla hai jaise jaante nahi hon phir aise neeche aise chale jao na toh neeche sunte bhi nahi hain

RA: hmm

NN: boleinge number nahi hai card nahi hai who nahi hai yeh cheez nahi hai who cheez nahi hai pareshani ban jaati hai hamare liye isi wajha se

RA: hmm hmm toh aap kia chahti hain k CHW aap k ghar aayein

NN: aayein le jaayein khud hi aayein 2 3 baar chalo roz nahi aaye haftey mein aayein 15 din k baad aayein checkup karne k liye who sahi hota hai agar call karo toh ajaate hain warna nahi aate hain yeh log

RA: hmm hmm acha hum asal mein aaye isliye hain aur is ko kaise behtar bana sakte hain aap ka mashwara hai hum kia aisa karein

NN: hamara mashwara toh yehi hoga 15 din baad 8 din baad khud hi aaye checkup k liye

RA: thek hai

NN: yeh sahi hoga aap ki dawaiyaan hoti hai joh hoti hai checkup wagera hote hain reportein wagera hoti hain who khud le jaayein itni sahulat banaayein is se aage bhi yeh bhi sahulat kar dein meharbaani hogi aap ki yeh bhi

RA: hmm hmm

NN: yeh bhi acha kaam hoga yeh bhi acha hi kaam horaha hai

RA: hmm

NN: sahulat yeh bhi hai sab kuch hai yeh bhi hojaayega aage

RA: acha yeh mujhe bataaein k waise CHW se kaise taluqaat hain aap logon k

NN: ache taluqaat hain mein ne toh ek hi bacha wahan pe paida kiya hai toh acha un logon ne deakhbhaal ki

RA: yeh joh health worker ghar pe aati hain in se

NN: yeh toh aati hi nahi hai na

RA: in se koi baat nahi hoti

NN: nahi baat hi nahi hoti hai aati hain call karo toh gaari mein bahar beithe rehte hain andar bhi nahi aate

RA: acha

NN: phir bahar jaldi ajao hum phir chale jaate hain wahan pe

RA: acha toh in se koi taaluq hi nahi hai

NN: nahi hamara inse koi taaluq nahi

RA: kisi health worker ko nahi jaanti

NN: nahi jaanti jab aate nahi jaante kaise

RA: hmm

NN: wahan pe joh le jaate hain peeche bhaago aap le k aayi hain mujhe mein dikha dun hamara kaam jaldi hojaaye bolegi aage joh beithe hain unse ja k baat karo hamare sath baat nahi karo

RA: aap k elaake mein health worker rehti hain

NN: han na rehti hain na in ki beti younis ki beti rehti hain naam bhi us ka mujhe nahi aata toh wohi rehti hain mauhalley mein ek amma bhi rehti hain who kaam karti hain

RA: joh center mein kaam karti hai

NN: center mein kaam karti hai

RA: who health worker toh nahi hai joh younis ki betiyan hain who health worker hain

NN: nahi who doctor bani huyi hai na

RA: acha

NN: han doctor bani huyi hai na who rehti hain

RA: who rehti hain

NN: wohi rehti hain who toh sunte hi nahi hain andar kon hai kon hai aise nahi bolte mauhalley k aaye huye hain k kuch hain kuch bhi nahi who nahi sunte

RA: acha aur kia pareshani aap logon ko huyi

NN: hamein koi pareshani nahi hai hum log wahan pe jaayein toh mashallah hamara kaam, acha hojaata hai hamein yeh pareshani hoti hai k gaari nahi aati leine k liye khud

RA: hmm

NN: yehi pareshani hai aur hamein koi pareshani nahi hai

RA: hmm aur health worker nahi aati aap k paas

NN: nahi aati andar nahi aati bahar aati hain gaari mein beithi rehti hain le k chali jaati hain aise poochti hain k tumhare ko kia masla hai kia masla nahi hai yeh nahi poochti hain

RA: hmm acha toh hum yeh chah rahe hain k gar yeh aap ko pooche nahi thek hai aap ne jaise kaha khud pooch liya karein k tabiat thek hai k nahi

NN: thek hai kia masla hai bacha hone wala hai kia masla hai aurat mein pregnancy hoti hai kitna masla hota hai is mein bohat pareshani hoti hai sufaid paani ki shikaayat bhi hoti hai us ko bohat masla hota hai uthne beithne mein bhi masla hota hai yeh bhi nahi poocheinge kia masla hai kis liye bulaaya hai mujhe kyun bulaya hai

RA: hmm hmm

NN: kia hai kahan le k jana hai tumhein rehri le k jana hai gulgoth mein le k jana hai kahan le k jana hai yeh nahi poocheingi jahan yeh le k neeche chor deingi bas gaari mein utro bas tumhara hamara kaam khatam

RA: acha

NN: yehi kaam hota hai un ka

RA: yahan se faasla ziada hai center ka

NN: han yahan se fasla bohat dur hai

RA: hmm

NN: dur sa parta hai

RA: aur aap khud se chale jaate ho

NN: khud se hum log bike pe chale jaate hain aisa masla nahi koi bimaar ho toh bukhaar ho mujhe koi masla ho toh mein bike pe chali jaati hun gaari nahi hai toh mein bike pe chali jaati hun

RA: hmm hmm hmm acha aur agar koi health worker aap k elaake ki aisi ho joh kareeb mein rehti ho us se aap ko faida hoga

NN: rehti ho toh ab rehti toh koi nahi hai jab hai toh who aati nahi hai

RA: hmm

NN: bas yehi masla hai

RA: joh elaake ki hain nahi aati

NN: who bhi nahi aati

RA: kia wajha kia hai aap ko pata hai kuch

NN: kia masla hoga mujhe kia pata ab kia masla hai kyun nahi aati hain ache nahi lagte hum log pata nahi kia hai kuch hogi us ko masla

RA: hmm hmm

NN: toh koi aise hote hain na ache nahi lagte bolte hain hum log nahi ja rahe is k ghar pe nahi jaayeinge

RA: hmm kabhi aisa hua k who aayi hon aur aap logon ne manah kar diya ho

NN: nahi nahi aisa hamare ghar ki aadat aisi nahi hai gaari mein joh aate hain na hamare ghar pe foran andar chor deite hain us ko pata hai kaam aate hain itni dur se aate hain kaam aayeinge na aise kon aata hai

RA: hmm hmm

NN: isi wajha se yeh log aate hain

RA: acha

NN: is mein koi pareshani nahi hai is mein mujhe yeh pareshani lagti hai jab call karo toh gaari aati hai aisa koi time hota hai balance wagera nahi hota hai insaan k paas toh gaari ko call karni hoti hai mein kehti hun 8 din baad 10 din baad khud hi aayein a k le jaaye pooche bhi sahi k kaisi tabiat hai kaisi tabiat nahi hai

RA: zaroorat hai k nahi hai

NN: zaroorat hai k nahi hai hamein zaroorat hogi toh hum bol deinge hamein zaroorat nahi hogi hum nahi boleinge hamein zaroorat nahi hai

RA: hmm hmm

NN: hamein kaam nahi tabiat thek hai agle hafte chalein jaayeinge kal chale jaayeinge kuch kaam kar rahe hain isi wajha se yehi masla hai aur hamein koi masla nahi hum log neeche jaate

hain mashallah hamara kaam acha hojaata hai bacha paida karne k liye gaye hamara kaam acha hogaya

RA: hmm acha yeh bataaein aap k 3 hamal huye na

NN: 4 hamal huye hain 3 zaaya huye

RA: 3 zaaya huye thy toh us mein se zinda bache aap k 3 hain

NN: 4 hain

RA: 4 hain aur sab se chota aap ka 3 saal ka hai

NN: han 3 saal ka hai

RA: thek hai aur ek ki delivery aap ki yahan pe huyi

NN: ek ki delivery yahan pe huyi

RA: aur joh 2 pehle huye who kab huye

NN: han yeh 9 saal pehle huye thy

RA: acha 9 saal pehle huye thy

NN: 9 saal pehle yeh dono twins thy nay eh dono

RA: acha acha

NN: yeh twins thy

Ayesah: acha yahan nahi thy

NN: yahan nahi thy yahan ki maloomaat hamein nahi thi na aur dusra bacha hamara do dhaai saal k baad paida hua tha who bhi hamein maloomaat wahan ki nahi thi

RA: leikin thy toh yahin na elaake mein

NN: elaake mein hum log isi ghar mein thy aur is center ka hamein maloomaat nahi thi

RA: thek hai

NN: aur jaise gaari aayi toh hamein poochne k liye jaise aap log aaye na poochne k liye aise yeh log bhi aaye kuch arsa pehle 4 saal pehle poochne k liye aaye toh phir hum ne us ko bataya

RA: hmm

NN: isi wajha se un ko phir is bache k doraan kabhi kabhi aate thy kabhi kabhi jaise 3 maheeney baad 4 maheeney baad yeh log aate thy

RA: hmm hmm hmm

NN: waise un se baat bahi hoti thi jab delivery thi toh un ki drip lagi thi toh who khud ghar se a k le k gaye thy mujhe

RA: acha yeh 4 saal poorani baat hai

NN: haan 4 saal poorani

RA: kyun k who bacha chota tha ab koi bacha nahi hai lekin is hamal mein aap k paas koi nahi aaya

NN: koi bhi nahi aaya 3 maheeney hogaye koi nahi aaya mere paas

RA: acha aap se pooch k yeh bhi nahi kaha k abhi filhaal hamal se toh nahi hain yeh bhi kisi ne nahi poocha

NN: koi nahi pooch k gaye

RA: acha

NN: insaan kyun jhoot bole han mujhe pooch k gaya bata dun who bata dun k mujhe koi nahi pooch k gaya hai

RA: thek hai acha aa center pe hi mulaaqaat hoti hai kisi health worker se ya kisi se

NN: joh mein bata rahi hun na joh yahan se le k jaate hain na jab gaari tak hote hain hote hain gaari se jaise insaan utar jata hai toh who un ka raabta khatam hojaata hai hamare se who aage bolte hain khud hi jao khud hi ja k kaam karo

RA: saare kaam apna karo

NN: apna apna karo

RA: aage aap

NN: aage kuch samjh aaye ya na aaye hamein ab bhi toh change hogaya hai na wahan pe banaya hai yahan pe banaya hai bahar a k beith gaye mein pehli dafa gayi toh mujhe kuch samjh mein nahi a rahi 3 4 saal baad gayi mujhe toh samjh mein nahi aayi mein ne kaha kia masla hua hai waise deir se beithe beithe beithe beithe mujhe kia pata tha token leina tha token ki wajha se mere 3 4 ghante zaaya hogaye mere

RA: oh haan

NN: us ne bola pehle token le aao token le aayi baazu ki naap ki usi wajha se 3 4 ghante

RA: aur gaari mein thi unhon ne bhi aap ko nahi bataya wahan ja k token leina hoga

NN: token leina hoga nahi kisi ne nahi bataya ab jab hum log gaye toh hamein pata lag gaya na jaate hi token le lein insaan

RA: hmm

NN: pata lag jaaye yeh number hai hamara isi number pe hamara number aayega

RA: hmm acha aas paas ki joh aurtein hain who jaati hain center pe un k paas aati hain health workers

NN: han yeh hamare parosi hain toh un k yahan pata nahi jaati hain ya nahi jaati

RA: kabhi aapas mein health worker ki baat cheet hoti hai k yeh center se aati hain larkiyan

NN: nahi nahi aati hi nahi hai jab aati hain meri cousin hai who toh bata deigi

RA: k paros mein bhi nahi aati

NN: aati nahi hai ziada is taraf gaariyan aati nahi hai

RA: acha is elaake mein nahi aati hai

NN: aati hain who neeche ki taraf jaati hain upar us side pe jaati is gali mein nahi aati call karo jab gaari aati hai is taraf

RA: kia is elaake k mein aisa kia hai who center waale is taraf nahi aate

NN: koi bhi masla nahi hai sab hi ghar hai koi call kare tab hi aate hain warna nahi aate jaise bacha paida ho toh khud checkup k liye ajaate hain

RA: hmm

NN: warna koi nahi aata

RA: aur aap logon ki taraf se koi rukaawat nahi hai k aap apni maloomaat deine mein sharam mehsoos kar rahe ho

NN: nahi nahi is se sharam ki kia baat hai jab itni mushkil se aap log bhi mushkil se nikal k aaye ho hamare paas hamare kaam k liye aaye na hamare kaam k liye bahar nikle huye hai na hamari sahulat k liye nikle huye hai na toh isi wajha se aap se sharmaane bataney wali kia baat hai is mein is mein toh koi pareshani nahi hai

RA: hmm hmm

NN: is mein toh koi pareshani nahi yeh toh hamari sahulat k liye aap log support kar rahe ho

RA: hmm hmm hmm

NN: isi wajha se mujhe koi pareshani nahi hai mein neeche jaati hun sab kuch mujhe center vmein koi pareshani nahi hoti hamein yeh pareshani hai gaari hafte mein 15 din mein 8 din mein gaari khud aaye checkup k liye poochne k liye aaye kia masla hai kia masla nahi hai

RA: hmm hmm thek hai yeh aap ne bara achi baat kahi aap ne kaha k health worker toh aati nahi hai jab tak phone na karein

NN: haan call nahi karo nahi aati

RA: nahi aati hain thek hai toh phir who aap ko maloomaat kia deingi thek hai k kia maloomaat deingi jab ghar aati nahi aap ko hamal k hawale se

NN: jab hamal k doraan nahi aati toh phir hamara kia faida aage peeche aane ka hamara toh koi faida nahi in ka phir kis liye aayeingi toh phir nahi aayeinge bacha toh paida hojaayega kis liye aayeinge who

RA: hmm

NN: sirf bache ki checkup k liye aayeinge who log

RA: acha aap ko lagta hai k sirf hamal k doraan aap ko health worker ki zaroorat hai is k elawa

NN: nahi kyun nahi zaroorat insaan jaise bacha bimaar hota hai insaan khud bimaar hota hai le k jaayein sahulat yeh toh honi chaiye hai na jab gaariyan aati hain leine k liye toh sab ko le k jaayein sab se poochein k tumhare ko kia masla hai tumhara neeche naam hai kia masla hai tumhare ko kyun call kiya hai kis wajha se call kiya hai un ka a k poochna hamare se un ka farz banta hai hamare se poochne k liye

RA: thek hai aur koi cheez joh aap batana chaahein koi k is k elawa toh who nahi aati ya aur koi cheez joh aap chahti hain jab who aana shuru karein toh who yeh yeh yeh kaam karein

NN: han na a k pooche un ka haq banta hai a k poochna un ko pata hota hai yeh bimaar hai thek hai yeh hamal se hai

RA: hmm

NN: in ko dawaai deini hai nahi deini hai is ka test karana hai kia masla hai in ko khoon ki kami hai in ko toh pata lagta hai na toh yeh mein kehti hun us ki hamare ko yeh sahulat chaiye gaari a k khud hi hamein le jaayein

RA: thek hai

NN: han yehi sahulat chaiye

RA: aur health worker aap k ghar khud se aaye

NN: haan khud se aaye poochne aaye who toh aati hain mauhallay mein toh aati hain jab hamare ghar nahi aati hum toh khud toh nahi bula sakte na toh hamare ghar mein bhi aao

RA: hmm hmm hmm

NN: unka haq banta hai na peeche aane ka poochne ka hamare ghar pe bhi aana un ko zaroori hai

RA: thek hai hmm chalein shukriya bohat bohat NN aap ka …..

IH-IDI-02 Recording: 13:30

RA: acha NN mujhe yeh bataaiye k aap ki umar kitni hai

NN: meri 29

RA: 29 hai acha aur aap ki taleem kitni hai

NN: meri metric hai bas

RA: metric kiya hua hai

NN: jee

RA: aurr ghar pe koi kaam karti hain

NN: ghar pe koi bhi kaam nahi karti bas housewife hun bache ko sambhaalna bas yehi

RA: thek hai aur kitne arsey se aap reh rahe ho yahan

NN: mere ko 13 saal hogaye shadi ko

RA: aur shadi k baad yahan par hi hain

NN: jee yahan par hi hun 13 saal

RA: haan kitne bache hain aap k

NN: mere 4 bache hain

RA: 4 bache hain sab se chota bacha kitni umar ka hai

NN: yeh saare 3 saal ki hai

RA: saare 3 aur sab se bara

NN: bara hai mera 12 saal ka beta

RA: 12 saal ka beta hai acha aur acha yeh mujhe bataayein aap k shohar kia karte hain

NN: mera shohar mazdoori karta hai

RA: mazdoori ka kaam karte hain acha acha ab mein aap se joh health worker hai us k hawale se baat karongi yeh kitne arsey mein aap k ghar aati hain

NN: yeh maheeney mein do maheeney mein aise aati hain hamare ghar pe aksar aati hain pata karti hain maloomaat karti hain k aap hamal se hain ya phir aur koi naya who pemaana hai is k baare mein maloomaat karti hain

RA: thek hai aap se hamal k baare mein

NN: jee bare tareeke se pyar se baat karti hain aati hain poochti hain pooch k phir chali jaati hain

RA: thek hai aaa aap k hawale se sirf maloomaat leiti hain ya bachon k hawale se bhi leiti hain

NN: bache ka poochti hain kitne bache hain aap k aap k shohar kia kaam karte hain bache k baare mein poochti hain

RA: bachon k hawale se kia poochti hain

NN: bachon k hawale se ziada nai poochti yeh poochti hain kitne bache hain aap k bache bimaar hain ya sahi hain kabhi mashwara bhi deity hain k yahan pe clinic hai yahan pe dawaai wagera le aao aise kar k mashwara bhi deity hain

RA: thek hai aur aap k hawale se kia kia cheezein poochti hain

NN: bas yeh joh mein ne aap ko bataya k hamal k baare mein poochti hain bas ziada nahi aati hain 5 min bhi nahi khari hoti aati hain pata kar k chali jaati hain

RA: thek hai

NN: aati hain laazim maheeney mein 1.5 maheeney mein do maheeney mein aati hain

RA: thek hai acha yeh mujhe bataayen aap kaisa mehsoos karti hain in k kaam k hawale se aap k ghar a rahi hain aap se pooch rahi hain

NN: acha feel hota hai lekin yeh k saamne wale masle masaail ziada hain thek hai acha feel hota hai aati hain baat karti hain poochti hain phir chali jaati hain aise

RA: hmm hmm aur kia masaail hai kia matlab

NN: yani k aap log kaise ho kaise nahi ho bare pyar se bare tareeke se yani baat karti hain dil khush hojaata hai aati hain ghar lekin ab ghar k maahaul ka pata hai

RA: ghar k maahaul ki wajha se

NN: haan mein in ko time nahi de paati

RA: thek hai acha yeh mujhe bataayein k jab aap akhari dafa hamal se huyi thi

NN: jee

RA: toh koi kirdaar ada kiya tha CHW ne aap k hamal k doraan

NN: nahi unhon ne kuch bhi nahi kiya tha bas who aaye thy khoon nikaala tha bachi ka meri ka aur unhon ne color ki dibiya aur ek bag de k gaye thy meri bachi ko khoon le k gaye thy bola aap k bachi ka khoon test hoga who le k chale gaye thy

RA: thek hai

NN: hmm

RA: lekin hamal k doraan jab bacha nahi hua tha

NN: nahi nahi kuch nahi

RA: aap ne unhein bataya tha hamal se hain

NN: jee mein ne bataya tha un ko leikin aisa kuch nahi hai kuch nahi karaai

RA: kia koi maloomaat nahi di koi maloomaat wagera di

NN: haan

RA: acha yeh mujhe bataayein k aa aur jab nauzaida bache thy tab koi

NN: nai aisa koi tab nahi tha

RA: nahi koi aap ki madad nahi ki

NN: nahi kuch bhi aisa nahi kiya madad kin a kuch kiya yani k yeh thin a mere pait mein yeh mere khayal se ek maheeney 1.5 maheeney ki thi toh who apni gaari mein bitha k le k gayi thi mujhe

RA: hmm

NN: ultrasound wagera karwaya tha unhon ne mujhe karwaya tha mujhe yaad hai is ki dafa mein

RA: thek hai thek hai

NN: hmm

RA: acha yeh mujhe bataayein k aap ko aisa lagta hai k yeh joh aap se poochti hain 5 min bhi nahi beithti chali jaati hain joh aap ne kaha toh un ko koi maloomaat deine ki zaroorat hai unko

NN: wahan pe register mein a jaata hoga yahan pe a k maloomaat karein aman walon ki taraf se aap ne kiya

RA: nahi

NN: hum toh aga khan ki taraf

NN: aga khan ki taraf se aayein hain toh haan yeh maloomaat karti hain phir chali jaati hain bas

RA: acha aap ko koi hichhichayat ya rukawat mehsoos hoti hai

NN: nahi kabhi nahi mehsoos hua mujhe

RA: aa un ko bataaney mein

NN: nahi mujhe kuch mehsoos nahi hua

RA: jaise aap ne abhi bataya k ghar k mahaul ka masla hai

NN:jee jee haan

RA: kyun yeh aisa kyun hai k yeh kyun nahi batana chahti

NN: kon

RA: yeh aap ki saas hai

NN: nahi bolti hain inko jaise pooraane khayaalaat ki hain na who bolti hain koi kuch bolega koi kuch bolega apne is mein gor nahi karti yani kisi k kehne pen a amal kar leiti hain in ko ghar mein ghusne nahi deina hai corona wale hain yeh kar jaayeinge dawai pila deinge lekin mein toh samjh sakti hun in baat ko aise thori na pila k chale jaayega dawaai wagera toh mein yeh samjhti hun ab joh k sath hai na family toh phir dar lagta hai thora sa na

RA: toh yeh cheez hoti hai acha mujhe batao k aisa aap ko lagta hai k is k through hum kuch behtar kar sakte hain koi logon ki soch mein tabdeeli la sakte hain

NN: nahi la sakte is tarhan k logon mein mere khayal se tabdeeli nahi a sakti

RA: nahi a sakti

NN: yeh aise hi raheinge

RA: acha aur joh CHW hain unhon ne koi kirdaar ada kiya ho aap ko center se joh hamare idaare hote hain us mein koi role ada kiya ho

NN: mere khayal se aisa koi role ada nahi kiya kyun k aisa kuch kara hi nahi hai koi banda bole k haan bhae bohat acha kuch kara ho bas yeh aati hain pata karti hain maloomaat kar k chali jaati hain

RA: aur chali jaati hain

NN: baaki aisa kuch nahi hai unko hum se faida ho humein unse faida ho aisa kuch nahi hai yani

RA: hmmhmm hmm acha mujhe yeh bataayein k aur kia cheez hum kar sakte hain k faida aap ko lage k haan joh yeh arahi hai inse koi faida hua

NN: koi hamal wali aurtein un k liye mein yeh keh sakti hun k aap na aayein har maheeney unka test wagera karein chechup wagera karwaayein ya phir apne clinic le k jaayein un ko ultrasound wagera karwaayein bas yeh un k faidemand hain ya koi goli achi si un ko dein ta k un ko faida ho aane wale bache k liye bhi faida ho

RA: aisa kuch nahi karti

NN: aisa kuch jee aisa kuch ho mein yeh chahti hun aisa kuch ho

RA: acha

NN: lekin aisa kuch abhi hai nahi aati hain maheeney mein aati hain pata kar k chali jaati hain aur joh hamal wali aurtein hain who toh bichaari aane jaane mein bari dikkat hoti hai pareshani hoti hai toh yani k apni gaari le kar aayein sahulat k liye un ko bithaayein or ultrasound wagera karwaayein takleef hai toh karwaayein

RA: hmm

NN: agar yani k apne nahi hai gunjaaish nahi hai dawaaiyan wagera bhi dein

RA: hmmmm

NN: mein yeh chahti hun k is tarhan kuch karein

RA: acha agar who is tarhan ki koi cheez bataayeinge toh aap log amal kareinge us pe

NN: abhi toh mere sath koi masla nahi haan agar kuch bataayeinge kuch kareinge toh amal toh karna parega sehat k hawale se hamare bache k sehat k hawale se achi mashwara deinge

RA: toh jaise kyun k yeh saari cheezein hamare center pe hoti hain aap ko kabhi kisi ne yeh bataya nahi

NN: nahi

RA: is tarhan ki

NN: is tarhan ki kabhi koi who nahi hua inse koi faida nahi hua mujhe bas mein ne bataya tha jab meri munni pait mein thi na

RA: hmm

NN: toh jab mein mujhe ultrasound karwaane k liye le k gaye thy yeh log ultrasound karwaaya tha gaari mein phir gaari mein hi chor k gaye thy

RA: acha wahan jab gaye toh kaisa wahan ka maahaul kaisa tha

NN: maahaul toh wahan ka sahi tha bilkul tareeke se baat karna tareeke se bulaana yani k banda bheer mein khara hota hai rash mein khara hota hai aisa kuch nahi tha jhamela kuch nahi tha yai k mein sahi gayi thi bilkul sakoon e beith gayi thi paani wagera piya mera number aaya ultrasound wagera karwaaya phir ultrasound karwaane k baad phir mein apne ghar agayi thi gaari mein beth k

RA: nahi kabhi koi aisi cheez huyi ho jis ki wajha se joh gussa hai ya k kyun agaye kyun interview le

NN: nahi mujhe kabhi gussa nahi hua

RA: haan

NN: nahi kabhi nahi aaya mujhe acha lagta hai aate hain poochte hain maloomaat karte hain

RA: hmm

NN: aur insaan ko pata bhi chal jaata hai knowledge mein izaafa hi hota hai toh mujhe toh acha laga lekin aage jis ki jaisi soch

RA: toh is ko hum change kar sakte hain aap ko lagta hai koi maloomaat hai agar hum hisakta hai is wajha se bhi aap ko na bata sakti hon k aap k ghar walon ki taraf se who aata hai na k kyun aaye kyun nahi aaye

NN: hmm

RA: is wajha se who maloomaat nahi de paati yeh aap ko lagta hai rukawat hai

NN: jee jee haan rukawat hai jab who ghar wale aise hain na thora sa who hojaate hain bas meri saas mujhe baatein sunarahi hai mujhe aisa lagta hai

RA: acha aur bachon k hawale se jab yeh choti thi is k liye aayin

NN: jee is k liye jab yeh choti si thi na meri beti jab who aayin thi mein ne aap ko bataya tha na jaise jab mein pregnant hoti hun na aati hain aksar pata karti hain maloomaat karti hain register mein naam wagera bhi hain in k shehar ka shohar ka ghar ka sab maloomaat hai unk paas

RA: thek hai

NN: hoti hai un k paas

RA: thek hai jab aap jaate hain center pet oh mil jaate hain

NN: jee jee sab maloomaat hoti hain yani aati hain na toh kia naam hain dikhaati hain ab banda parh bhi leita hai likha hua hota hai sab kuch

RA: hmm

NN: dikh jaata hai k haan bhae ID card hai yani k meri devraani hai toh unse kitne bache hain kitne bache nahi sab record un k paas maujood thy

RA: hmm hmm hmm acha aur aap ko kia lagta hai k yeh joh kaam kar rahi hain

NN: hmm

RA: k is se aap ko koi faida hai

NN: kheir mujhe toh koi faida nahi hai is se faida nazar bhi nahi aata k faida hai yeh hai k achi baat hai aati hain maloomaat karti hain bas

RA: kyun nahi lagta is se aap ko koi faida hai

NN: faida aisa hota na jaise koi bimaar hota hai mein ne aap ko bataya na masle masaail pregnant hota hai toh le k jaati hain sahulat hoti yeh sahulat kuch bhi nahi hai mere khayal se toh

RA: yeh sahulat nahi hai

NN: jee sahulat nahi hai

RA: lekin abhi toh aap ne kaha k jab hamal se thi

NN: bas jab thi toh ab mein ne baakiyon se suna hai nahi le k jaati lekin meri munni ab saare 3 saal ki hogayi hai

RA: hmm

NN: aati hain waise toh lekin yeh mein ne nahi suna k bachon ko le k jaana gaari mein bitha k is tarhan toh kuch nahi hai na

RA: hmmm

NN: aisa kuch nahi hai

RA: yeh sahulat nahi hai

NN: jee sahulat nahi hai

RA: bas pooch k chale jaate hain

NN: haan pooch k chale jaate hain jaise insaan hain aata hai pata karta hai maloomaat karta hai k kia masle masaail hai bas poochna likhna yeh sab kuch thori na hota hai aap aaye poocha likha aur chale gaye lekin kuch sahulat toh dein na gaari ki ya phir ultrasound ki koi toh sahulat dein aisa koi sahulat nahi hai meri nazar mein toh

RA: thek hai koi sahulat nahi hai

NN: jee

RA: acha aur bachon k hawale se yeh toh ultrasound tak hogaya bachon k hawale se kia faida hai ya nahi hai

NN: lekin who aisa poochti nahi bachon ka poochti hai bas

RA: kitne bache hain

NN: kitne bache hain aap k aap k shohar kia kaam karte hain kitne bache hain bas aur kia karti hain aap mein ne kaha ghar pe kuch bhi nahi karte bas bache ko sambhaalne ki zimedari hai meri

RA: agar hamal se nah on

NN: jee

RA: toh koi aur cheez poochti hain aap se

NN: aur toh kuch nhai poochti

RA: aap k sehat k hawale se kuch bataati hain

NN: haan NN aap thek hain bare tareeke se bolti hain hum kehte haan hum thek hai Allah ka shukar hai bache bohat thek hain Allah ka shukar hai aur kia kar rahi hain aap mein ne kaha kaam wagera ghar k bohat saare hote hain who kar rahi hoti hun bache sambhaalna

RA: jee agar who aap se nahi poochti toh aap ne khud se socha k aap unhein bataayein

NN: nahi mein ne kabhi nahi socha k mein unhein khud se bataon khud hi bichaare aate hain khud hi poochti hain who humse

RA: hmm kyun nahi socha k aap unko khud se bataayein

NN: kyun k mein time de nahi sakti na mere 4 bache hain mashaallah parhne bhi yeh kare who kare lekin mein is tarhan lagi huyi hun ..yeh mere liye keemti hain meri saas ne shalwaar di hai na seeni hai machine mein ne utha k rakh di

RA: hmm

NN: bache ko parhne bheja parhne se mere bache aaye khaana banaane lag gayi khana diya unko ab mujhe machine mein beithne lagi un k kapre wagera sahi karne thy saas k who karongi phir sham ka time hojaayega bache ajaayeinge un ko khana deina hai phir tution bhejna hai bohat masle masaail hain aap ko pata hai zindagi mein banda kuch kar leita hai

RA: lekin jaise who toh khud arahi hain

NN: jee who khud arahi hain

RA: tab aap ne kabhi socha mein yeh shuro horaha hai yeh aurton k bohat saare masaail hain toh mein health worker a rahi hain toh mein pooch leiti hun

NN: nahi mein ne kabhi aisa mehsoos nahikiya

RA: wajha kia thi

NN: khud hi nahi poocha dil hi nahi kiya poochne ka toh khud hi aage se maloomaat kar rahi hain ab mein kia un ko karon

RA: toh jab ek cheez hoti hai na ek toh aap k ghar e chal k arahi hain

NN: chal k arahi hain meharbaani hai un ki

RA: toh jab who aati hain toh aap ne kyun zaroorat mehsoos nahi ki k aap isi ko bata dein shayad koi hal nikal aaye kuch aisa kabhi hua

NN: nai nahi nahi aisa kabhi unse baat share nahi kari koi baat unse share nahi kari unhon ne khud ne poocha mein ne kuch nahi bola aage se meinn ne kuch nahi bola wohi aati thi bichaariyan ek do aurtein aati hain

RA: kia aitebaar ki kami hai

NN: nahi aisa koi masla nahi hai bas apni taraf se khud hi mehsoos nahi hua in se kuch poochun

RA: acha

NN: jee aisa koi masla hota toh doctorni k paas jaati hun aisa ziada eham masla hota hai

RA: hmm

NN: doctorni se poochti hun k bhae yeh masla hai mere sath lekin unse mein ne share nahi kari discuss nahi kari

RA: khud se nahi kari

NN: nahi kuch nahi

RA: aap ko zaroorat hi mehsoos nahi huyi

NNnahi

RA: acha waise mujhe yeh bataayein k aa jaise hamara center hai udhar yeh cheez aap ko kabhi nahi bataai

NN: jee

RA: aap ne kaha k udhar who hoti hai aur aap ne kaha k hamein in k through ultrasound wagera ki service mil jaaye ya hamein le k chale jaaye center

NN: jee

RA: toh yeh faida ho

NN: jee faida hona chaiye

RA: thek hai aur koi cheez

NN: bas aur kuch nahi

RA: k hum is ko aur kaise behtar bana sakte hain

NN: ab yeh toh mein aap ko bata nahi sakti aap khud mashaallah samjhdaar hain

RA: aap ko lagta hai k joh worker aati hain in ki taleem itni hai taleemyafta hain

NN: jee jee taleem yafta hain

RA: k aap unhein khud se bataati nahi hai who joh pooch liya toh pooch liya

NN: nahi mein ne khud se kabhi un ko kuch nahi bataya

RA: haan toh us se kia yeh baat hai k aap ko lagta hai k un mein itni taleem nahi hai k who aap ka masla hal kar sake kia yeh wajha toh nahi

NN: nahi nahi aisa kabhi nahi mujhe mehsoos hua

RA: aap k elaake ki hoti hain joh workers aati hain

NN: elaake ki nahi hoti pata nahi mein ne kabhi in ko deakha nahi elaake ki nahi hai hamari jahan se aap aayi hain na wahin se aayi hain

RA: hmm

NN: toh bas who aati hain pata karti hain pata kar k chali jaati hain maheeney do maheeney mein aise aati hain maloomaat karti hain phir chali jaati hain

Aesha: acha aap un pe bharosa karti hain

NN: jee

RA: aap unhein maloomaat de deity hain

NN: de deite hain

RA: kia wajha hain kyun de deity hain

NN: deity hain bata rahi thi itne pyar se baat kari meharbani hai k who arahi hain khud chal kar aaye hum se seedhe mau baat nahi karte buri lagti hain na isliye insaan k naate bande ko khuda k naate hona chaiye baat karni chaiye tareeke se mein kehti hun

RA: hmmmm thek hai aur koi cheez joh aap hamein batana chahein

NN: aur koi aisi cheez hi nahi joh bataon aap ko

RA: chalein thank you k aap ne hamein itna time de diya

NN: aap ki bhi meharbani aap log hamare ghar aaye…

Date: 27-01-2021

RA: acha NN aap ki umar kitni hai

NN: 33 saal

RA: 33 saal aur aap ne taleem kahan tak haasil ki hai

NN: 3 classes parhi hun

RA: 3 classes parhi hain

NN: hmm

RA: aur aap k kitne bache hain

NN: mashallah chaar bache hain mere

RA: chaar bache hain

NN: haan 3 zaaya hochuke hain

RA: 3 zaaya hochuke hain

NN: aur chaar mere paas hain

RA: chaar hain aap k paas

NN: hmm

RA: sahi hai acha NN mujhe bataaiyega k aap k paas joh health worker aati hain hamari thek hai kia who baqaaidgi se ghar aati hain

NN: nahi aati hain jab phone karo toh gaari leine k liye aati hai waise nahi aati hain hamare paas

RA: waise nahi aati

NN: waise nahi aati

RA: acha toh yeh aap ko kaisa laga matlab kab kab aur kitne kitne arsey mein aati hain aur kab kab aati hain

NN: jab gaari mein phone karo toh aati hai jab waise koi bhi nahi aata yahan pe

RA: waise koi bhi nahi aata

NN: koi nahi aata

RA: acha jab aati hain toh gaari k hawale se aati hain

NN: haan gaari k hawale se aati hain le k jaati hain aur waise chor k jaati hai who

RA: hmm

NN: waise nahi aati mere paas

RA: kuch aap se a k maloomaat nahi leiti

NN: nahi koi maloomaat nahi leiti is baar koi maloomaat nahi mein ne un k paas call bhi ki toh phir maloomaat nahi horahi

RA: acha aur kitna arsa hua hai koi CHW aap k ghar nahi arahi hai

NN: yeh pehle bache mein toh aayi thi is bache mein nahi arahi hai

RA: acha sirf bache k hawale se ya

NN: waise nahi aati bacha bhi hojaaye toh phir bhi nahi aati hain jab call karo toh aate hain gaari le k warna nahi aati

RA: thek hai acha hamesha jab who aati hain ek jaisi aati hain ya alag alag aati hain

NN: alag alag aati hain

RA: alag alag aati hain kabi koi aisi aayi hai joh aap se sirf aap k hawale se baat karti hon aap se pooche

NN: nahi nahi bilkul bhi nahi

RA: k aap k kitne bache hain

NN: nahi nahi yeh bilkul nahi poochti koi bhi nahi aata

RA: acha thek hai kabhi aisa hua ho k who aayi hon

NN: nahi mein ghar pe nahi hoti hun toh ammi k ghar pe paas pe mere ajaati hain waise ghar pe hoti hun toh ammi bol deity hain k nahi hai ghar pe nahi hai kal ajana kal sham tak ajaayeingi yeh who aise

RA: jaise yahan rehte huye kitna arsa hua hai

NN: mujhe 10 saal hogae

RA: 10 saal hogaye hain in 10 saalon mein aap k paas CHWs nahi aayin

NN: nahi

RA: acha aur aap k kitne bache hain joh zinda hain is waqt

NN: chaar bache hain

RA: chaar bache hain in chaar bachon mein kabhi jab hamal se huyi tab

NN: haan hamal se hoti hun tab bulaati hun toh tab ajaate hain waise poochne k liye who nahi aati

RA: khud se nahi aati

NN: khud se nahi aate mere paas

RA: acha aur jab hamal se hoti hain aap toh us mein who koi aap ko maloomaat wagera kia kaam karti hain us mein jab aap phone kar k bulaati hain toh kia hota hai who kia leiti hain kia information aap se leiti hain

NN: bas jab call karti hun toh bolti hun gaari bhejo dawai leini hai who ajaati hain phir le k wahin pe chor deity hain hum log khud hi wahin pe kuch karte hain phir

RA: acha aap ko koi maloomaat wagera nahi

NN: nahi kuch maloomaat nahi karti isliye bulaya hai kyun kyun bulaaya hai bacha hone wala hai kia masla hai kuch masla hai kuch bhi nahi poochti kuch le k jaati hain mujhe yahan se wahin pe chor deity hain aage phir khud karte hain joh kuch bhi hota hai wahan dawai leini hoti hai toh aage khud jana hota hai

RA: acha aur pichle ek maheeney mein koi aisi aap k paas koi CHW nahi aayi jis mein aap se yeh poocha ho k aap k kitne bache hain

NN: nahi koi bhi nahi aaya aise poochne k liye yeh ek dafa ek martaba poochne k liye aayi bache ko joh hifazati teeke lage hain nahi lage mein ne kaha k mere bachon ka course poora hua hai bas wohi poochne k liye aayi thi yeh do dhaai maheeney pehle aayi thi

RA: hmm

NN: phir us k baad koi nahi aaya mere paas

RA: acha do dhaai maheeney pehle aayi thi

NN: do dhaai maheeney pehle aayi thi

RA: acha toh yeh aap ko kaisa lagta hai k yeh joh CHWs hain jab aap phone karti hain tab aati hain

NN: haan sab aate hain acha hai wahan pe jao toh achi dawai deite hain checkup acha karte hain un ka ikhlaaq bohat acha hai jaise bacha hota hai wahan pea chi tarhan deikhte hain deakhbhaal karte hain

RA: aur joh ghar par aati hain un k hawale se

NN: joh ghar pe aati nahi hai call karo toh aati hain waise warna koi nahi aata

RA: koi nahi aate acha aur aap k hamal k doraan kabhi

NN: jaise kal parso se meri tabiat thek nahi hai mein who pareshan mein ne kaha gaari bahar aati hain in se number le lo ja k toh mein call kar k baat kar lun gaari mangwa lun dawai leine k liye toh abhi tak koi nahi aaya mere paas toh koi bhi nahi aata

RA: acha aap abhi hamal se ho

NN: haan hamal se hun na

RA: acha

NN: isi wajha se hamal se hun na mein kuch khaati hun na toh upar ki tarhan who hoti hai isi wajha se mein ne kuch le k aaon kuch checkup kara k aaon toh phir tabiat thek hojaati hai

RA: hmm hmm

NN: isi wajha se koi aata nahi hai pehle aate thy pehle mere bache huye thy na us mein aate thy jab yeh bacha mera paida hua na toh koi bhi nahi aaya

RA: yeh kitna bara hai

NN: yeh 3 saal ka hai

RA: acha

NN: toh ab koi bhi nahi aata hai mein ne kaha mein uzma baaji ko bolon k mere paas toh koi bhi nahi aata hai call karo toh phir gaari ajaati hai warna koi bhi nahi aati yahan pe

RA: hmm acha yeh mujhe bataaein k jab hamal se yeh toh ek bacha hua aur bache

NN: han mera ek hi bacha hua hai mera

RA: haan hamal k doraan kabhi CHW ne koi a k kirdaar ada kiya aap ki koi madad ki ho

NN: is bache mein aayi thi bari gaari le k aaye thy na

RA: hmm

NN: toh tab blood pressure high hogaya tha na isi wajha se usi doraan le k gaye thy mujhe khud rehri goth mein le k gaye thy gaari mein

RA: hmm hmm toh bas sirf yehi kiya

NN: haan yehi kiya

RA: aur yeh kitni dafa hua kitni baar aayi

NN: ek baar aayi

RA: ek dafa aayi 9 maheeney mein

NN: haan 9 maheeney mein ek dafa aayi jab hi mein call karti hun jab gaari aati hai warna checkup k liye koi bhi nahi aata mere paas

RA: koi bhi nahi aata kabhi koi bache ko wazan naapne

NN: who jab bacha hojaata hai na ek do din k baad aate hain wohi checkup karne k liye ek dafa wohi aate hain

RA: sirf ek dafa

NN: ek dafa aate hain phir

RA: acha regular matlab har maheeney do maheeney baad nahi araha

NN: nahi nahi who nahi aate sirf call karo toh phir aate hain wohi bata rahi hun call karo toh ajaate hain warna koi nahi aata

RA: acha aa toh mein yeh janna chah rahi hun k hamare paas aap ki information toh hai

NN: haan number nikaalna pare mere paas toh nahi koi bhi nahi aaya na mein khud chali gayi neeche mein ne kaha mein toh aati jaati rehti hun na aap naam nikaalo hamara naam bhi hoga beech mein us ne naam nikaala toh wahin pe checkup kiya baazu ka bhi checkup hua na toh un logon ne nahi kiya

RA: center pe jab gayi thi

NN: haan jab center pe neeche gayi toh sab kuch banaaye uzma se baat ki na mein ne kaha mein toh pehle aayi thi mera naam nikaalo hoga beech mein naam nikaala toh naam toh tha beech mein toh un logon ne bohat wait karaya bohat 3 4 ghante wait karaya us k baad ja k unhon ne suna phir

RA: hmm acha yeh mujhe batao k center tak kaise gayi jab call kar k

NN: nahi center mein toh bike pe gayi thi mein

RA: khud gayi thi

NN: khud gayi thi mein wahan pe

RA: acha

NN: number bhi nahi tha gaari ka toh aur jana bhi tha mein ne kaha pata nahi kyun menses wagera arahe hain na

RA: hmm

NN: toh bache ka masla tha isi wajha se jana para wahan pe mein ne kaha khud chali jaati hun number bhi le k ajaaongi kuch masla bhi hoga who log bata deinge k kia masla hai jab kuch hai wagera un logon ne kaha bacha hai isi wajha se un logon ne file bana k di mujhe toh isi doraan 3 4 ghante wahin pe zaaya hogaye

RA: acha toh

NN: acha yeh toh masla nahi hai k 3 4 ghante lag gaye jab call karo toh aate hain warna toh waise checkup k liye koi bhi nahi aate poochne nahi aate tumhari tabiat kaisi hai kaisi nahi hai yeh nahi poochne k liye aate hain

RA: hmm toh aap ko lagta hai k un ko aana chaiye

NN: aana chaiye na aana chaiye pata karna chaiye na un ko patient ki tabiat kaisi hai kaisi nahi hai zaroori toh nahi k insaan k paas kabhi balance hota hai kabhi nahi hota hai isi wajha se in ko pata karna ek do din mein pata karna chaiye yahan pe

RA: hmm hmm acha agar yeh aayeingi aise regular basis pe aap k paas aayeingi aap k hawale se maloomaat leingi kia aap un ko apni maloomaat deingi

NN: jaise aap poochne aaye ho waise poochne k liye

RA: nahi aise nahi jaise k aap k kitne bache hain abhi aap hamal se toh nahi hain

NN: who aati hi nahi hai

RA: nahi aise aayeinge toh phir aap bataaeingi unhein

NN: poocheinge toh bataaeinge phir

RA: aur aap acha mehsoos kareingi is baare mein agar who aap se poochein

NN: haan poochein pata chalega na yeh aage hamare ko jaante hain kuch masla hai jaise jaante nahi hon phir aise neeche aise chale jao na toh neeche sunte bhi nahi hain

RA: hmm

NN: boleinge number nahi hai card nahi hai who nahi hai yeh cheez nahi hai who cheez nahi hai pareshani ban jaati hai hamare liye isi wajha se

RA: hmm hmm toh aap kia chahti hain k CHW aap k ghar aayein

NN: aayein le jaayein khud hi aayein 2 3 baar chalo roz nahi aaye haftey mein aayein 15 din k baad aayein checkup karne k liye who sahi hota hai agar call karo toh ajaate hain warna nahi aate hain yeh log

RA: hmm hmm acha hum asal mein aaye isliye hain aur is ko kaise behtar bana sakte hain aap ka mashwara hai hum kia aisa karein

NN: hamara mashwara toh yehi hoga 15 din baad 8 din baad khud hi aaye checkup k liye

RA: thek hai

NN: yeh sahi hoga aap ki dawaiyaan hoti hai joh hoti hai checkup wagera hote hain reportein wagera hoti hain who khud le jaayein itni sahulat banaayein is se aage bhi yeh bhi sahulat kar dein meharbaani hogi aap ki yeh bhi

RA: hmm hmm

NN: yeh bhi acha kaam hoga yeh bhi acha hi kaam horaha hai

RA: hmm

NN: sahulat yeh bhi hai sab kuch hai yeh bhi hojaayega aage

RA: acha yeh mujhe bataaein k waise CHW se kaise taluqaat hain aap logon k

NN: ache taluqaat hain mein ne toh ek hi bacha wahan pe paida kiya hai toh acha un logon ne deakhbhaal ki

RA: yeh joh health worker ghar pe aati hain in se

NN: yeh toh aati hi nahi hai na

RA: in se koi baat nahi hoti

NN: nahi baat hi nahi hoti hai aati hain call karo toh gaari mein bahar beithe rehte hain andar bhi nahi aate

RA: acha

NN: phir bahar jaldi ajao hum phir chale jaate hain wahan pe

RA: acha toh in se koi taaluq hi nahi hai

NN: nahi hamara inse koi taaluq nahi

RA: kisi health worker ko nahi jaanti

NN: nahi jaanti jab aate nahi jaante kaise

RA: hmm

NN: wahan pe joh le jaate hain peeche bhaago aap le k aayi hain mujhe mein dikha dun hamara kaam jaldi hojaaye bolegi aage joh beithe hain unse ja k baat karo hamare sath baat nahi karo

RA: aap k elaake mein health worker rehti hain

NN: han na rehti hain na in ki beti younis ki beti rehti hain naam bhi us ka mujhe nahi aata toh wohi rehti hain mauhalley mein ek amma bhi rehti hain who kaam karti hain

RA: joh center mein kaam karti hai

NN: center mein kaam karti hai

RA: who health worker toh nahi hai joh younis ki betiyan hain who health worker hain

NN: nahi who doctor bani huyi hai na

RA: acha

NN: han doctor bani huyi hai na who rehti hain

RA: who rehti hain

NN: wohi rehti hain who toh sunte hi nahi hain andar kon hai kon hai aise nahi bolte mauhalley k aaye huye hain k kuch hain kuch bhi nahi who nahi sunte

RA: acha aur kia pareshani aap logon ko huyi

NN: hamein koi pareshani nahi hai hum log wahan pe jaayein toh mashallah hamara kaam, acha hojaata hai hamein yeh pareshani hoti hai k gaari nahi aati leine k liye khud

RA: hmm

NN: yehi pareshani hai aur hamein koi pareshani nahi hai

RA: hmm aur health worker nahi aati aap k paas

NN: nahi aati andar nahi aati bahar aati hain gaari mein beithi rehti hain le k chali jaati hain aise poochti hain k tumhare ko kia masla hai kia masla nahi hai yeh nahi poochti hain

RA: hmm acha toh hum yeh chah rahe hain k gar yeh aap ko pooche nahi thek hai aap ne jaise kaha khud pooch liya karein k tabiat thek hai k nahi

NN: thek hai kia masla hai bacha hone wala hai kia masla hai aurat mein pregnancy hoti hai kitna masla hota hai is mein bohat pareshani hoti hai sufaid paani ki shikaayat bhi hoti hai us ko bohat masla hota hai uthne beithne mein bhi masla hota hai yeh bhi nahi poocheinge kia masla hai kis liye bulaaya hai mujhe kyun bulaya hai

RA: hmm hmm

NN: kia hai kahan le k jana hai tumhein rehri le k jana hai gulgoth mein le k jana hai kahan le k jana hai yeh nahi poocheingi jahan yeh le k neeche chor deingi bas gaari mein utro bas tumhara hamara kaam khatam

RA: acha

NN: yehi kaam hota hai un ka

RA: yahan se faasla ziada hai center ka

NN: han yahan se fasla bohat dur hai

RA: hmm

NN: dur sa parta hai

RA: aur aap khud se chale jaate ho

NN: khud se hum log bike pe chale jaate hain aisa masla nahi koi bimaar ho toh bukhaar ho mujhe koi masla ho toh mein bike pe chali jaati hun gaari nahi hai toh mein bike pe chali jaati hun

RA: hmm hmm hmm acha aur agar koi health worker aap k elaake ki aisi ho joh kareeb mein rehti ho us se aap ko faida hoga

NN: rehti ho toh ab rehti toh koi nahi hai jab hai toh who aati nahi hai

RA: hmm

NN: bas yehi masla hai

RA: joh elaake ki hain nahi aati

NN: who bhi nahi aati

RA: kia wajha kia hai aap ko pata hai kuch

NN: kia masla hoga mujhe kia pata ab kia masla hai kyun nahi aati hain ache nahi lagte hum log pata nahi kia hai kuch hogi us ko masla

RA: hmm hmm

NN: toh koi aise hote hain na ache nahi lagte bolte hain hum log nahi ja rahe is k ghar pe nahi jaayeinge

RA: hmm kabhi aisa hua k who aayi hon aur aap logon ne manah kar diya ho

NN: nahi nahi aisa hamare ghar ki aadat aisi nahi hai gaari mein joh aate hain na hamare ghar pe foran andar chor deite hain us ko pata hai kaam aate hain itni dur se aate hain kaam aayeinge na aise kon aata hai

RA: hmm hmm

NN: isi wajha se yeh log aate hain

RA: acha

NN: is mein koi pareshani nahi hai is mein mujhe yeh pareshani lagti hai jab call karo toh gaari aati hai aisa koi time hota hai balance wagera nahi hota hai insaan k paas toh gaari ko call karni hoti hai mein kehti hun 8 din baad 10 din baad khud hi aayein a k le jaaye pooche bhi sahi k kaisi tabiat hai kaisi tabiat nahi hai

RA: zaroorat hai k nahi hai

NN: zaroorat hai k nahi hai hamein zaroorat hogi toh hum bol deinge hamein zaroorat nahi hogi hum nahi boleinge hamein zaroorat nahi hai

RA: hmm hmm

NN: hamein kaam nahi tabiat thek hai agle hafte chalein jaayeinge kal chale jaayeinge kuch kaam kar rahe hain isi wajha se yehi masla hai aur hamein koi masla nahi hum log neeche jaate

hain mashallah hamara kaam acha hojaata hai bacha paida karne k liye gaye hamara kaam acha hogaya

RA: hmm acha yeh bataaein aap k 3 hamal huye na

NN: 4 hamal huye hain 3 zaaya huye

RA: 3 zaaya huye thy toh us mein se zinda bache aap k 3 hain

NN: 4 hain

RA: 4 hain aur sab se chota aap ka 3 saal ka hai

NN: han 3 saal ka hai

RA: thek hai aur ek ki delivery aap ki yahan pe huyi

NN: ek ki delivery yahan pe huyi

RA: aur joh 2 pehle huye who kab huye

NN: han yeh 9 saal pehle huye thy

RA: acha 9 saal pehle huye thy

NN: 9 saal pehle yeh dono twins thy nay eh dono

RA: acha acha

NN: yeh twins thy

Ayesah: acha yahan nahi thy

NN: yahan nahi thy yahan ki maloomaat hamein nahi thi na aur dusra bacha hamara do dhaai saal k baad paida hua tha who bhi hamein maloomaat wahan ki nahi thi

RA: leikin thy toh yahin na elaake mein

NN: elaake mein hum log isi ghar mein thy aur is center ka hamein maloomaat nahi thi

RA: thek hai

NN: aur jaise gaari aayi toh hamein poochne k liye jaise aap log aaye na poochne k liye aise yeh log bhi aaye kuch arsa pehle 4 saal pehle poochne k liye aaye toh phir hum ne us ko bataya

RA: hmm

NN: isi wajha se un ko phir is bache k doraan kabhi kabhi aate thy kabhi kabhi jaise 3 maheeney baad 4 maheeney baad yeh log aate thy

RA: hmm hmm hmm

NN: waise un se baat bahi hoti thi jab delivery thi toh un ki drip lagi thi toh who khud ghar se a k le k gaye thy mujhe

RA: acha yeh 4 saal poorani baat hai

NN: haan 4 saal poorani

RA: kyun k who bacha chota tha ab koi bacha nahi hai lekin is hamal mein aap k paas koi nahi aaya

NN: koi bhi nahi aaya 3 maheeney hogaye koi nahi aaya mere paas

RA: acha aap se pooch k yeh bhi nahi kaha k abhi filhaal hamal se toh nahi hain yeh bhi kisi ne nahi poocha

NN: koi nahi pooch k gaye

RA: acha

NN: insaan kyun jhoot bole han mujhe pooch k gaya bata dun who bata dun k mujhe koi nahi pooch k gaya hai

RA: thek hai acha aa center pe hi mulaaqaat hoti hai kisi health worker se ya kisi se

NN: joh mein bata rahi hun na joh yahan se le k jaate hain na jab gaari tak hote hain hote hain gaari se jaise insaan utar jata hai toh who un ka raabta khatam hojaata hai hamare se who aage bolte hain khud hi jao khud hi ja k kaam karo

RA: saare kaam apna karo

NN: apna apna karo

RA: aage aap

NN: aage kuch samjh aaye ya na aaye hamein ab bhi toh change hogaya hai na wahan pe banaya hai yahan pe banaya hai bahar a k beith gaye mein pehli dafa gayi toh mujhe kuch samjh mein nahi a rahi 3 4 saal baad gayi mujhe toh samjh mein nahi aayi mein ne kaha kia masla hua hai waise deir se beithe beithe beithe beithe mujhe kia pata tha token leina tha token ki wajha se mere 3 4 ghante zaaya hogaye mere

RA: oh haan

NN: us ne bola pehle token le aao token le aayi baazu ki naap ki usi wajha se 3 4 ghante

RA: aur gaari mein thi unhon ne bhi aap ko nahi bataya wahan ja k token leina hoga

NN: token leina hoga nahi kisi ne nahi bataya ab jab hum log gaye toh hamein pata lag gaya na jaate hi token le lein insaan

RA: hmm

NN: pata lag jaaye yeh number hai hamara isi number pe hamara number aayega

RA: hmm acha aas paas ki joh aurtein hain who jaati hain center pe un k paas aati hain health workers

NN: han yeh hamare parosi hain toh un k yahan pata nahi jaati hain ya nahi jaati

RA: kabhi aapas mein health worker ki baat cheet hoti hai k yeh center se aati hain larkiyan

NN: nahi nahi aati hi nahi hai jab aati hain meri cousin hai who toh bata deigi

RA: k paros mein bhi nahi aati

NN: aati nahi hai ziada is taraf gaariyan aati nahi hai

RA: acha is elaake mein nahi aati hai

NN: aati hain who neeche ki taraf jaati hain upar us side pe jaati is gali mein nahi aati call karo jab gaari aati hai is taraf

RA: kia is elaake k mein aisa kia hai who center waale is taraf nahi aate

NN: koi bhi masla nahi hai sab hi ghar hai koi call kare tab hi aate hain warna nahi aate jaise bacha paida ho toh khud checkup k liye ajaate hain

RA: hmm

NN: warna koi nahi aata

RA: aur aap logon ki taraf se koi rukaawat nahi hai k aap apni maloomaat deine mein sharam mehsoos kar rahe ho

NN: nahi nahi is se sharam ki kia baat hai jab itni mushkil se aap log bhi mushkil se nikal k aaye ho hamare paas hamare kaam k liye aaye na hamare kaam k liye bahar nikle huye hai na hamari sahulat k liye nikle huye hai na toh isi wajha se aap se sharmaane bataney wali kia baat hai is mein is mein toh koi pareshani nahi hai

RA: hmm hmm

NN: is mein toh koi pareshani nahi yeh toh hamari sahulat k liye aap log support kar rahe ho

RA: hmm hmm hmm

NN: isi wajha se mujhe koi pareshani nahi hai mein neeche jaati hun sab kuch mujhe center vmein koi pareshani nahi hoti hamein yeh pareshani hai gaari hafte mein 15 din mein 8 din mein gaari khud aaye checkup k liye poochne k liye aaye kia masla hai kia masla nahi hai

RA: hmm hmm thek hai yeh aap ne bara achi baat kahi aap ne kaha k health worker toh aati nahi hai jab tak phone na karein

NN: haan call nahi karo nahi aati

RA: nahi aati hain thek hai toh phir who aap ko maloomaat kia deingi thek hai k kia maloomaat deingi jab ghar aati nahi aap ko hamal k hawale se

NN: jab hamal k doraan nahi aati toh phir hamara kia faida aage peeche aane ka hamara toh koi faida nahi in ka phir kis liye aayeingi toh phir nahi aayeinge bacha toh paida hojaayega kis liye aayeinge who

RA: hmm

NN: sirf bache ki checkup k liye aayeinge who log

RA: acha aap ko lagta hai k sirf hamal k doraan aap ko health worker ki zaroorat hai is k elawa

NN: nahi kyun nahi zaroorat insaan jaise bacha bimaar hota hai insaan khud bimaar hota hai le k jaayein sahulat yeh toh honi chaiye hai na jab gaariyan aati hain leine k liye toh sab ko le k jaayein sab se poochein k tumhare ko kia masla hai tumhara neeche naam hai kia masla hai tumhare ko kyun call kiya hai kis wajha se call kiya hai un ka a k poochna hamare se un ka farz banta hai hamare se poochne k liye

RA: thek hai aur koi cheez joh aap batana chaahein koi k is k elawa toh who nahi aati ya aur koi cheez joh aap chahti hain jab who aana shuru karein toh who yeh yeh yeh kaam karein

NN: han na a k pooche un ka haq banta hai a k poochna un ko pata hota hai yeh bimaar hai thek hai yeh hamal se hai

RA: hmm

NN: in ko dawaai deini hai nahi deini hai is ka test karana hai kia masla hai in ko khoon ki kami hai in ko toh pata lagta hai na toh yeh mein kehti hun us ki hamare ko yeh sahulat chaiye gaari a k khud hi hamein le jaayein

RA: thek hai

NN: han yehi sahulat chaiye

RA: aur health worker aap k ghar khud se aaye

NN: haan khud se aaye poochne aaye who toh aati hain mauhallay mein toh aati hain jab hamare ghar nahi aati hum toh khud toh nahi bula sakte na toh hamare ghar mein bhi aao

RA: hmm hmm hmm

NN: unka haq banta hai na peeche aane ka poochne ka hamare ghar pe bhi aana un ko zaroori hai

RA: thek hai hmm chalein shukriya bohat bohat NN aap ka …..

Date: 27-01-2021

RA: AA k aap ki umar kitni hai

AA: jee 22 saal

RA: 22 saal huye hain

AA: jee 22 saal

RA: thek hai aur aap ne taaleem haasil ki huyi hai

AA: ziada nahi ki

RA: phir bhi kitni

AA: yehi 2 class tak parha hai

RA: acha 2 class tak parha hua hai acha aur kitne bache hain aap k

AA: mere dou

RA: do bachein hain kon

AA: yeh ek hai choti hai

RA: acha pehli yehi is ki kia umar hai

AA: is ka abhi 6 saal shuro hua hai

RA: thek hai aur yeh choti wali ka

AA: is ka bhi yeh teesra maheena hai

RA: teesra maheena hai acha sahi hai acha aap ko yahan rehte huye kitna time hogaya hai

AA: yahan pe shadi se pehle thi

RA: shadi se kab se

AA: yeh 12 13 saal hogaye hain

RA: 12 13 saal hogaye hain acha thek hai acha mujhe yeh batao yeh joh CHWs hain who yahan par baqaidgi se aati hain

AA: jee

RA: aap k ghar par aati hain

AA: jee

RA: aur jab who aap k ghar par aati hain who kis kisam k kaam karti hain kia poochti hain aap se

AA: yehi poochti hai k jab pregnant hote hain toh khaane wane k baare mein poochti hain goliyaan bhi deity hain peele wali hoti hai who bhi deite hain chocolate wali bhi deite hain sahi treatment karti hain

RA: acha yeh karti hain aur is k elawa who chocolate kyun kaise deity hain matlab kuch karti hai ya direct chocolate deity hain

AA: nahi bas us ko pata naam wagera toh pata hota hai bas who chocolate sath mein goliyaan bhi de deity hain kha leite hain hum log

RA: who joh bhi deity hain aap kha leite hain

AA: jee

RA: acha aur aap se poochti kia hai chocolate deine se pehle ya pregnancy k doraan kia poochti hain

AA: who kehti hai k is se din mein do khaao is se sehat acha rahega bache pe bhi asar sahi hoga goliyon se bhi sahi asar hoga bache ko aap ka bhi sahi hoga aur wahan pe jab jaate hain toh matlab jab ultrasound wagera karte hain tab bhi sahi hai kuch masla

RA: acha aur kitne arsey mein aati hain aap k ghar pe

AA: yehi bas jab kabhi ek din k baad aati thi kabhi do din k baad aati thi

RA: acha waise kitne ek din do din matlab jab aap hamal se hoti hain tab ek din chor k ek din pe aati hai

AA: kabhi matlab kabhi kabhi ek din chor k aati hai kabhi har din aati hai is tarhan

RA: acha har din a k kia karti hai

AA: bas pooch leiti hai phir chali jaati hai

RA: poochti kia hai

AA: k tabiat the hai hum kehte hain thek hai

RA: acha

AA: blood pressure wagera check karte hain phir

RA: yeh hamal k doraan a k karti hain

AA: jee han

RA: agar hamal se nahi hoti ho phir aati hain

AA: nahi phir nahi aati

RA: koi nahi aata

AA: nahi

RA: koi kuch nahi poochta

AA: bas kabhi kabhi kuch aurtein aati hain jaise abhi aap log agaye is tarhan a k naam wagera likhte mardum shumaari yeh wagera wagera

RA: haan toh yeh aap ka joh naam poochti hain agar aap hamal se nahi hoti hain aap k shouhar ka naam poochti hain

AA: jee haan

RA: thek hai toh yeh kitne kitne arsay baad aati hain

AA: abhi toh bohat arsay baad tak toh nahi aayi bohat arsa hogaya

RA: hmm

AA: matlab kabhi kabhi ajaati hain abhi kia pata kal ajaaye is tarhan se joh hai pata nahi hota

RA: thek hai aur who sirf aap se aap k hamal k baare mein bache k baare mein poochti hain

AA: jee haan joh pregnancy k doraan 03:43-03:46 woh joh kehte hain mardum shumaari who bas naam wagera bacho ka naam shouhar ka aur mera naam bas likhte hain kehte hain bas chali jaati hain

RA: thek hai acha abhi abhi kareeb kareeb mein koi aisa a k poocha hai k is mein sirf aap k hawale se poocha aap k kitne bache hain aap k shouhar ka kia naam hai

AA: nahi nahi kabhi kuch nahi

RA: aisa nahi hua

AA: nahi nahi

RA: thek hai acha aur bachon k hawale se kia kaam karti hain joh yeh workers hain jaise agar bacha hua hai

AA: bacha hua hai ajaati hai toh is ka is ko naap tol bhi karti hai aur is ka baazu bhi naap karte hain sar naap kar k chale jaate hain

RA: kuch kehti nahi hai aap se bas yeh karti hain chali jaati hain

AA: haan chali jaati hain

RA: aap poochti nahi ho unse kuch

AA: nahi bas yeh kehti hun k kitan wazan hai in ka toh bolti hain k itna itna hai sahi hai wazan

RA: wazan sahi hai

AA: haan

RA: acha kabhi is ko koi masla masaail hua ho

AA: nahi bas is tarhan koi masla masaail nahi bas seena kharab who bhi shukar hai sahi hogaya

RA: who deakhti hain seena wagera

AA: han is ka checkup kar leiti hain

RA: acha

AA: toh phir cheezein wagera la leiti hain phir sar pe rakhti hai ek cheez pata nahi kia hai 04:51-04:53 mera blood pressure check karti hain

RA: aap ko hamal k doraan aa kaise check karti thin

AA: who bhi wazan kiya blood pressure check kiya hum log tareekh deite hain neeche jaate hain na toh wahan pew azan karta hai aur blood pressure check karta hai phir jab ultrasound pe maheena ultrasound kar leit hain aur wapis ajaate hain

RA: acha aur ghar pe a k kia karti hain who

AA: bas khaas kuch nahi bas naam wagera likh leiti hain pehle aurtein aati thi likh leiti thi bas aur kuch nahi

RA: aur kuch nahi acha aap yeh in ka joh yeh kaam hai is k hawale se aap kaisa mehsoos karti hain matlab aap k liye kaisa hai yeh kaam

AA: bas kia kahein hum log ziada matlab is ko tawajju nahi deite ya phir kisi ko takleef bhi ho toh ziada who nahi karte

RA: acha k

AA: matlab

RA: yeh a k poochti hain ya ziada tawajju

AA: haan tawajju nahi deity toh phir hum log ziada private ko chale jaate hain

RA: is ki kia wajha hai kabhi koi aisa waqiya kia hua hai kuch aisi baat huyi hai joh aap in ko itna tawajja nahi deite kia wajha hai

AA: us din hum beithte beithte thak jaate hain (laughing)

RA: kahan beithte beithte wahan pe beithte beithte bas jab time ajaaye ultrasound karte hain ya nahi karte matlab who toh maheeney mein time se karte hain nay eh joh goliyaan wagera kehte hain k jab hoti hai toh de deite hain nahi hoti toh kehte hain khatam hogayi wapis ghar ajaate hain dusra tareekh le k bas

RA: bas yeh hota hai toh phir jab yeh worker aati hain ghar pet oh phir aap in ko nahi tawajja deite

AA: nahi(laughing) ziada nahi deite

RA: kyun k center pe aap ko who karne k time ziada lagta hai

AA: haan bohat ziada time lagta hai 3 bajhe ajaate hain kabhi 3:30 bajhe kabhi 4 bajhe bohat ziada time lag jaata hai

RA: isliye aap private chali jaati hain

AA: haan is liye private chale jaate hain

RA: acha toh hum is cheez ko worker aap keh rahe ho joh worker aati hai who aap se kia kehti hai hamare center aayein kia matlab mein yeh janna chah rahi hun

AA: mashallah se sab doctor bohat ache hain wahan pe insan k meezaaj se pata chalta hai yahan pe joh larki aati hai who bhi bohat achi hain meezaj mein bas matlab

RA: aap ka apna hai kia kia wajha kia hai koi toh baat hogi na jis ko hum sunna chah rahe hain ta k us k upar kaam kar sake

AA: bas koi takleef ho na koi cheez se log sahi hojaata hai matlab teeke wagera se kyun k mujhe bhi jab dard jab zor ziada neeche chaale jaata tha is bachi mein kyun k mere 5 saal k baad toh mera is ka wazan joh hai na hamesha mera na yahan tak hota tha pait k neeche

RA: hmm

AA: toh us k baad teeka lagaati thi yeh log mujhe nahi lagaate thy is ki wajha se mein private chali jaati thi wahan pe jab jaati thi aur aati thi bilkul sahi

RA: hmm acha yeh batao k yeh joh aap se a k pooch rahi hoti hain cheezein in mein deine mein aap ko acha lagta hai kaisa lagta hai jab yeh poochti hain

AA: sun leite hain hum(laughing)

RA: aap sunti hain

AA: sun leite hain

RA: matlab aap ko sunti ho sochti bhi toh hogi na kyun pooch rahe hain kia wajha hai

AA: sahi hai yeh baat toh thek hai log kehte hain pata nahi kia hua kabhi toh bohat ziada log dar rahe hain polio mein joh hai na is mein jarasim bache us se marte hain abhi kehte hain polio log nahi pilaate toh abhi who joh teeke hifazati bachon k us mein lagate thy hum bohat is cheez se bohat darte hain

RA: toh worker aati hain toh is ko us se who karti hogi polio wali hain ya who hain

AA: abhi yahan pe mere abhi joh yeh hajra hai na who 5 saal tak mere bete ko pilaaya bas Allah pe yaqeen hai aur us aurat pe abhi in ko bhi who pilaati hai

RA: acha

AA: us ne abhi kaha k aaj camp laga hai bachon ka checkup kareinge teeke lageinge mein kaha is ko teeka neeche center se lagaaye aur mein neeche se lagaaongi aur kisi jagha se nahi kyun k us se wahan pe larkiyon ko bhi jaante hain hum log aur bohat arsa hogaya hai wahan pea ate jaate rehte hain is liye kisi aur jagha se nahi lagaaongi wahan se lagaaongi aur abhi is ka teeko ka card pata nahi kahan pe hum log teeke lagaate hain polio us se pilaati hun kabhi center mein bhi nahi pilaayi

RA: haan who toh thek hai who toh thek hai polio k hawale se toh hum nahi baat kar rahe bas hum chahte hain k hum apne kaam ko joh hamari worker hain kyun k na who polio lagaati hain na teeka lagaati hain

AA: polio joh hai na who neeche se lagaate hain hum log center se lagaate hain

RA: haan

AA: polio joh hai who nahi

RA: haan yeh hamari worker kaam nahi karti hai

AA: sahi hai

RA: thek hai toh mein yeh janna chah rahi hun who a k aap se maloomaat deity hain who aap se kehti hongi k center par aayein jab hamal k doraan

AA: han us ne kaha k phone karo gaari a k tumhein khud le k jaayeinge

RA: hmm

AA: yeh who log keh rahe thy k bachon ko joh bhi beemari ho aap log tabiat thek nahi jab k hamal se call kar k a jaayeinge hum log ko le jaayeinge

RA: kabhi aap ne worker se baat ki kabhi masla hua bache k hawale se hum apne hawale se worker ne aap se baat ki ho aap ne un ko bataya ho k yeh horaha hai hamein center jaana hai ya koi kuch is tarhan ki baat

AA: nahi is tarhan koi baat nahi huyi

RA: nahi hua aisa nahi hua acha aap k hamal k doraan joh hamari worker hai us mein koi madad ki ho aap ki koi kirdaar ada kiya ho

AA: nahi kuch bhi nahi

RA: aap ne us ko poocha aap ne us ko bataya k mein hamal se hun

AA: jee haan naam wagera likh leite hain phir joh hai na jab chale jaate hain card ban k phir wapis ajaate hain us ne toh kuch nahi kaha han yeh keh deity hai thora sab hi takleef ho phone kar k hamare log ajaayeinge aap ko leine k liye aur toh kuch nahi

RA: aur nahi kuch hota acha lekin aa aap ne unhein bataya k aap ko takleef thi aap keh rahi thi aap ko dard hota hai yeh aap ne kabhi worker ko bataya who toh har maheeney aati hain na aap k paas aap ne bataya

AA: nahi mein neeche doctoron ko dikhaati hun us ko batati us ne kaha neeche ajao doctor ko baat karo jab chali jaati hain toh doctor ka usay batate they

RA: acha thek hai acha yeh mujhe batao k aap ko chocolate bhi di inhon ne saari chhezein aap ne khud se bataai na toh yeh kaise aap k ghar hi aayi hogi

AA: jee han

RA: thek hai

AA: who bhi hum log chale gaye thy us ne yeh kaam karna hai who khud se le kar ajaate thy

RA: khud se le kar a jaati thi ok thek hai is k baare mein kia lagta hai aap ko kaisa mehsoos hota hai

AA: bas sahi hai

RA: sahi hai yeh hona chaiye aap k khayal se ya nahi hona chaiye

AA: mujhe nahi pata aap logon ko ziada pata hoga (laugh)

RA: nahi hum aap ko janna chahte hain na k hamari community kaisa mehsoos karti hain kyun k hum un ko behtar banana chahte hain

AA: sahi hai achi cheez hai

RA: achi cheez hai honi chaiye acha kia lagta hai faida hua koi aap ko

AA: sahi hai matlab blood pressure kam hojaata hai dood mein pee leite hain sharbat se pee leite sahi hai us ka zaiqa pehle peeti thi mein phir beech mein hamal se insaan kitna chir hota hai phir mein nahi peeti thi phir baad mein thora thora pee leiti thi

RA: acha acha acha choti bachi hai abhi toh kitne maheeney ki hai

AA: abhi teesra maheena

RA: teesra maheena hai na toh is k hawale se koi worker ne kirdaar ada kiya ho

AA: nahi bas yeh paida hogayi

RA: hmm

AA: toh ek dafa larki a gayi is ko tol kar k is ka naam likh liya phir mein ne is ko teeke k liye le kar gayi mein

RA: hmm

AA: peer k din han pehla teeka us ne kaha phir us k baad abhi yeh do dafa aaye phir nahi aaye

RA: phir nahi aaye is k baad

AA: bas matlab us k sath sirf 3 dafa

RA: 3 dafa aayin aur unhon ne wazan wagera kiya acha us k sath is hawale se kuch bataya aap ko

AA: nahi mein ne us se kaha is ka seena bohat kharab hai toh us ne kaha k bas phone kar neeche le k aana phir doctor deakheinge

RA: thek hai sahi hai toh aap ko lagta hai k joh hamari worker aati hain yeh toh aap k ghar arahi hain na toh yeh aap ko maloomaat aap ko de sakti hain bata sakti hain in ko kia maloomaat deini chaiye aap k khayal se

AA: yeh log toh hum log ko kuch nahi deite na

RA: koi matlab koi aap aap ko batate nahi is ka seena kharab hai toh yeh kar lo pregnancy mein yeh horaha hai toh yeh bata dou koi is tarhan ki baat karte hain

AA: nahi nahi is tarhan ki koi baat

RA: kuch bhi aap k khayal se in ko pata honi chaiye yeh baatein

AA: who joh aurat aati thi who kehti thi acha khoraak khao yeh khao who khao lekin har kisi ne bas ek joh aati thi who jis us ka kaam yehi hoga joh logon ko call wagera mein us ka toh who karti thi

RA: acha jis liye joh khaane jin k hawale se aati thi who yeh batati thi

AA: sirf batati thi

RA: aur joh dusri aati hain who apna kaam karti hain chali jaati hain

AA: jee

RA: aur koi maloomaat aap ko nahi deity

AA: nahi

RA: aap k khayal se k joh ziadatar aati hain un ko yeh baat aap ko kuch bataani chaiye aap ki sehat k hawale se bache ki sehat k hawale se us ne nahi bataya mein aap se aap ki soch jaanna chah rahi hun kia unhein batana chaiye

AA: haan batana chaiye kyun k joh hamare liye bachon k liye sahi hoga wohi kareinge aur toh kuch nahi

RA: who bataaeingi toh amal kareinge

AA: han kyun nahi kareing (laugh) jab bache ko deakho maa kehti hai k zehar khaane se mera bacha sahi hojaata hai toh mein zehar kha leiti hun ta k us k dood mein chati hun bacha sahi hojaaye maa kitni hoti hai apne bache k liye

RA: hmm

AA: toh is ki wajha se bachon k liye joh bhi sahi ho who hamein pata hota hai

RA: acha

AA: apne liye nahi bachon k liye toh log kar sakte hain

RA: hmm hmm acha yeh mujhe batao k aur kis kisam ki cheezein aap ko lagta hai agar hum aap ko center jana pare ya center pe jaayein toh kia cheez hum aisi karein joh hum worker ko bhi ghar ghar bheijte hain na ta k hamari joh aurtein hamal se hain bache un k huye hain un k liye asaani hojaaye toh hum worker k through aisa kia karein k hum un ko mazeed asaan bana sakein un k liye sahulat ko behtar bana sakein

AA: bas jab bache ko kuch bhi ho aap log in ka sahi se treatment karo aur kuch nahi chaiye

RA: hmm

AA: is ki wajha se hum log jab bache bhi bimaar hon toh hum private le k jaate hain kyun k yahan pe kia yahan pe seena kharab hai toh who naak ka who dawaai de deite hain k yeh daal k seena sahi hojaayega bas har kisi ko ek ki dawaai deity hai jaise calpol deity hai bas ek powder ka sharbat deity hai ek ek deity hai ziada aur nahi aur jab bache ka seena bohat kharab ho toh is ka naak band hai toh who dawai deite hain toh is ki wajha se hum log saaf baat hai k nahi aate

RA: acha phir nahi aate

AA: nahi hum log bas private le k jaate hain who dawaai wagera likhte hain hum log who pilaate hain bache sahi hojaate hain

RA: acha aur phir who yeh karti hai aap k sath k jab worker aati hai ghar pe phir poochne k liye

AA: who toh muft mashware itne deity hai k bas kuch bolo mat

RA: acha

AA: muft k mashware deity hai aur kuch nahi deity

RA: kuch aur nahi deity mashware

AA: aur jab bache ka seena kharab ho toh bas yehi katra nahi pata nahi kia naam hai mujhe us ka naam bas who de deity hai naak ka katra

RA: hmm hmm hmm nasal drops

AA: haan yehi deity hai

RA: acha

AA: aur bache ka jab seena kharab ho toh abhi jab hum log private le k jaate hain toh is k mehange mehange dawaai aati hai toh us se sahi hojaata hai seena abhi bhi is ka seena bohat ziada kharab tha hum log le kar gaye do dafa hispatal Allah ne sahi kiya

RA: thek hai aap yeh kehti hain muft k mashware deity hain aur kuch nahi deity

AA: nahi bas (laughing)

RA: acha aur mashware deina bhi ek tarhan se un ka kaam hai na un k kaam mein aata hai toh joh mashwara who deity hain aap us pea mal karte hain

AA: han kehti hai k bhaanp toh hum log de deite hain ghar pe

RA: hmm

AA: us se mashaallah bohat sakoon milta hai

RA: hmm

AA: bas aur

RA: aur yeh mashwara joh hai aap ko center mein milta hai ya yeh joh worker ghar pe aati hai

AA: nahi yeh center mein joh doctor nahi beithi huyi who kehte hain k matlab bachon ko bhaanp dou aur yeh dawaai daalo is se seena sahi hojaayega is ka seena kharab nahi hai naak band hai

RA: hmm hmm

AA: bas hum log phir private le k chale jaate hain who kehte hain is k seene se awaazein nikal aati hai

RA: hmm

AA: us ka toh bache ka toh seena kharab bulgum saara jam jaata hai seene mein toh is se naak band nahi hota nay eh toh seena kharab hota hai mein toh is ko nahi le kar gayi meri bari beti thin a who feeder peeti thi dood bhi peeti toh feeder se bache ka seena bohat kharab hota hai

bas us ki wajha se mein ne jab yeh center chora is ka bohat ziada seena kharab tha itni awaazein arahi thi us ne kaha k nahi is ka naak band hai phir private is ko thek injection laga powder k bas us ki wajha se mein ghabraai gayi hun na bas abhi is ko mein ne bilkul bhi ek teeka lagwaaya sirf aur bilkul nahi le k gayi is ko joh bhi hota hai private le kar jaati hun

RA: acha thek hai acha mujhe yeh bataaein k joh yeh toh sirf center ki baat hogayi na hum worker k baare mein baat karein joh ghar par aati hai toh yeh mujhe batao k us ko kuch kar sakte hain k hum aap k liye koi behtar sahulat farhaam kar sakein

AA: kuch aisa k bache us se sahi hojaaye

RA: kuch aisa karein

AA: jee han

RA: kis matlab maa k hawale se

AA: maa k hawale se maa k sehat ka

RA: haan toh kia bataaein kuch bataaein koi aur cheez kare

AA: bas who sirf itna bataaye k yeh acha hai toh hum khud hi kareinge

RA: acha

AA: koi baat nahi

RA: haan

AA: lekin bas itna ho k ta k koi kehta hai yeh khaao toh yeh khaa leite toh yeh kehte hain yeh nahi sahi hai who khao who kha leite toh is mein beech o beech insaan phans jaata hai

RA: hmm

AA: toh bacha maa bhi kharab hojaati hai bacha bhi kharab hojaata hai

RA: aap ko matlab aap ko koi masla koi yeh keh raha hai yeh bataayein yeh bataayein confusion

AA: haan bas yeh kehte hain yeh bohat acha hai tumhare sehat k liye aur jab bacha saans nahi le raha bache ko yeh khaao is se sahi hojaaogi BP bohat ziada low horahi hai toh yeh khaao is se sahi hojaayega toh yeh kha leite hain phir who kha leite hain phir yeh kha leite hain kehte hain k bas us se tabiat ziada kharab hojaata hai

RA: acha toh is tarhan ho k ek cheez ho ta k hamein pata lage hum jab bhi hamal se phir in log ko batane ka koi masla bhi nahi hoga hamein pata hoga hum khud le kar aayeinge hum kaheinge kyun k in logon ne bataya hoga k kuch bhi na karo sirf yeh cheez khaao

RA: thek hai acha joh worker aati hain unko jaante ho aap

AA: jee haan

RA: hmm jaanti hain kaise matlab

AA: matlab ziada nahi jaante lekin center mein deakh leite hain toh pehchaan leite hain warna is tarhan nahi jaante

RA: is arhan nahi jaante

AA: ziada nahi jaante

RA: aap k idhar ek ghar pe hai who alag alag workers aati hain

AA: nahi ek bas ek hoti hai toh hamare ghar mein bhi aati hai aur dusre auron k gharon mein bhi jaati hain bas wohi ek hi aati hai

RA: us ko jaanti nahi

AA: kabhi matlab ek dafa jaise aap log aaye aap k baad aur log aayeinge toh us k baad aur log aayeinge ek nahi ho dusre abhi is ka wazan do dafa hua na ek din hogaya phir who log aayeinge phir jab kiya us se pehle toh aur log thy phir joh agaye who aur log thy

RA: acha alag alag aati hain

AA: jee

RA: acha thek hai toh yeh aap k elaake ki hoti hain

AA: yeh toh mujhe pata nahi

RA: unhein jaanti nahi hain

AA: nahi mein usay nahi jaanti

RA: acha aap jaanti nahi hain is ka matlab who aati hain alag alag aati hain workers

AA: haan

RA: aur alag alag maloomaat le kar jaati hain bache ka wazan karti hai koi kuch karti hai

AA: nahi dono ne ek jaisa bache ka wazan bhi kiya jis tarhan us ne kiya is tarhan is ne kiya

RA: aur aap k hawale se a k koi aur poochta hai

AA: nahi

RA: aap k hawale se kisi ne kuch nahi poocha

AA: nahi jab hum log jaate hain hamara hawala toh khatam hojaata hai toh phir bache ka shuro hojaata hai (laugh) phir is k hawale se matlab koi hawala nahi bas wazan kar leite hain naap kar leite hain bas

RA: acha yeh hota hai acha mujhe yeh batao k joh hamari worker hai thek hai na joh yeh a rahi hain is ne aap ko center se jora hai aap ko lagta hai k is ki wajha se aap center jana shuro huye

AA: nahi

RA: nahi inhon ne aap ko koi maloomaat nahi di

AA: nahi bas yeh ek aurat aati hai ajaati hai toh naam likhti hai bas

RA: who aurat kon hai

AA: us ko mein nahi jaanti matlab meri behan jaanti hai mein kisi ko nahi jaanti kyun k mein ziada aati jaati nahi meri behan k mashallah yahan pe bache hote hain bache sirf ek yahan pe huye yeh koi govt saare private mein huye lekin ziadatar who yahan pe aati jaati hai matlab har maheeney is tarhan toh is k liye hum log nahi jaate rehri goth mein is mein gayi thi mujhe thora khoon nikal aaya is liye gayi thi toh is ka padaaish ka time nahi toh kehte hain k tumhare 36 haftey poore hogaye aur baar baar check karne se tabiat ziada kharab hogi toh is ki wajha se mein wahan se bhaag gayi chutti bhi nahi maangi bhaag gayi mein wapis ghar agayi yeh kia bas private kuch din k baad private chali gayi aur Allah ne paida kiya

RA: yeh normal hua ya operation

AA: yeh normal hua

RA: yeh center wale aap ko kohigoth bheijte hain

AA: jee haan

RA: acha toh is wajha se bhi narazgi huyi hai

AA: han bas yeh center joh hai center sahi hai lekin yehi kohigoth yeh bilkul sahi nahi hai

RA: kia wajha hai

AA: is mein toh pehle badmizaaj doctor hain us ka toh mizaaj hi sahi nahi hai

RA: hmm

AA: kehte hain k bas bikhaari jaise beithe ho bheek maangte ho Allah ne insaan ko itna diya hai k apna k paida jab bacha deite hain toh wasila bhi ban jaata hai na hum logon ne kaha tum log kia shukar hai hamare paas itna hai k hum log apna elaaj acha kar sakte hain

RA: hmm

AA: is mein mein gayi thi toh bas do din thi ek din mein ne raat guzaari dusre din mein bhaag gayi hum logon ne chutti maangi doctor ne kaha nahi tumhare 36 haftey tum bacha paida kar k jao bacha nahi horaha toh zabardasti nikaale phir mein ghar agayi mein ne apne shohar ko bola bas ab sab cheez le k jao ghar aate hain hum log ghar aaye phir 10 dn k baad joh hai na meri beti hogayi

RA: hmm toh yeh batao abhi mein 10 din wahan beithti jab tak yeh paida nahi hoti hum log toh chutti kahi who kehti hain sabar karo madam khana kha k phir chutti deity hai meri behan jaati hai kehti hai sabar karo sabar karo

RA: kis baat pe sabar karo

AA: chutti k liye

RA: hmm hmm

AA: pata nahi do ghante teen ghante subha se un ko yeh kehte hain mein apni behan ko kehti thi koi dar nahi hai mein kis liye beith jaon

RA: hmm

AA: toh is ki wajha se hamein chutti do toh who kehti hain nahi abhi sabar karein madam ka khana nahi poora shadi ka khaana tha

RA: acha yeh baat aap ne joh mujhe bata rahe ho yeh kabhi aap ki delivery aap gaye thy kohigoth gaye thy toh yeh kabhi kisi worker ne aap se poocha k bhae delivery kaisi huyi aap ki bacha hua aisi baat poochi aap ka dil karta hai k who aap se pooche

AA: mera toh wahan pe nahi hua meri behar yeh azra joh hai na is ka operation joh hai na kohigoth mein hua tha

RA: hmm

AA: mein ne toh na kabhi delivery ki hai phir dusre kamre mein shift kiya or operation k jaghae mein joh hai na yahan dabao de rahe hain bohat ziada dard hua kehte hain k sahi nahi hai

RA: who sahi nahi hai lekin mein yeh janna chah rahi hun k yeh saari cheezein joh hoti hai na aap k sath center pe log aap se ache se baat nahi karte hospital jaate ho jaise

AA: nahi center mein sab ache hain center mein koi bhi is tarhan nahi sab k mashaallah bohat ache hain

RA: hmm hmm hmm

AA: lekin yeh kohigoth nahi hai yeh sahi nahi hai

RA: haan toh who aap ne kabhi center pe a k hamari joh worker aati hain is ko kabhi bataya k wahan hum gaye thy toh yeh hamare sath hua

AA: haan usay bataya abhi yeh amna doctor bohat achi doctor hai phir us ne kaha tum ne acha kiya tum ghar agayi toh phir us ne hai na mera card bhi le kar agayi bohat achi doctor hai

RA: hmm

AA: us ne kaha sahi hai

RA: hmm hmm

AA: matlab mujhe joh bhi masla hota tha usay batati who mera sahi elaaj karti

RA: hmm hmm aur worker ko bataya worker ne kaha

AA: nahi mein ne usay nahi bataya

RA: nahi bataya aur us ne poocha bhi nahi

AA: nahi

RA: toh aap ko lagta hai k agar yeh aap se poochein is tarhan ki koi baat aap ko koi masla pareshani in ko bataaeinge toh aage aap ki baat chali jaayegi

AA: bas abhi koi zaroorat nahi hai (laugh)

RA: zaroorat aap ko lagti nahi hai

AA: nahi

RA: kyun aisi kia wajha hai

AA: mera shohar abhi kehta hai k bas abhi yahan pe nahi jaogi is logon ne tumhein zalil kiya

RA: acha

AA: mera shohar gusse ka bohat teiz hai na toh us ne kaha k bas

RA: haan yeh mein janna chah rahi hun k aap ko kabhi lagta hai k jaise joh aap maloomaat deina chahte ho toh us mein koi rukaawatein hain CHW ko nahi batatey kia wajoohaat hai

AA: nahi bas is tarhan koi bhi nahi hai

RA: jaise ek toh aap ne bataai na apni mau se bataai k bhae wahan gaye thy udhar un ka rawaaiya acha nahi tha

AA: han kohigoth ka tha rawaaiya acha nahi tha

RA: rawaaiya acha nahi tha

AA: aur bas kuch bhi nahi hai

RA: k hai na matlab zaban na hon worker aap se sahi tarhan se baat na karti hon

AA: nahi sab bohat ache hain center k joh hai na who sab bohat ache hain lekin yeh kohigoth nahi hai is ka sirf badmizaaj hai

RA: hmm

AA: aur yeh center neeche aga khan ka is ka sab bohat ache hain

RA: acha

AA: sab larkiyan doctorein bhi achi hain larkiyan bhi achi hain

RA: lekin mein yeh janna chah rahi hun phir kyun nahi aati center pe

AA: bas (laugh) bohat ziada intezaar karna parta hai hamare akele ghar jab akela ghar hota hai toh shohar ko sambhaalna bachon ko sambhaalna ghar ko sambhaalna itna mushkil hota hai

RA: thek hai intezar karna parta hai toh is mein kabhi worker ki madad leine ki koshish ki

AA: hmm nahi

RA: kyun

AA: hum log nahi leite kehte hain bas jab ajaaye time pet oh chale jaayeinge warna beithte rehte hain hum log is tarhan nahi karte kuch ziada bas who log token deite hain 50 number bhi kyun na hon beithti hun bas intezar karti hun aur jab pata chal jaaye intezar ka toh waqt khatam hogaya aur abhi mein ziada nahi ruk sakti phir wapis chali jaati hun phir mein nahi rukti

RA: mujhe yeh bataayein is ko worker k kaam ko behtar bana k aap ki madad kar sakte hain aap ko lagta hai k hum k aap ki joh pareshania hain is tarhan worker k zariye kuch aap ki madad kar sakte hain is ko behtar bana sakte hain koi mashwara deina chahogi in k hawale se

AA: matlab bohat ziada log hote hain jis ko bohat zaroorat hoti hai na matlab bohat ziada zaroorat hoti hai toh us se poocho mein abhi kia kahon

RA: aap ko zaroorat nahi hai

AA: nahi zaroorat nahi

RA: aap matlab mein yeh is liye chah rahi hun k ek hamari who hai k hum hamari worker ghar ghar pe jaati hai hum chahte hain k aurton ko us se madad mile thek hai na aur hum yeh chahte hain aap ko center pe le k aayein toh jaise k aap ne kaha intezar ziada karna parta hai toh hum is k hawale se kuch kareinge k bhae hum kis tarhan se is tarhan ka kaam karein k joh hamari aurtein gharon se uth k kaam chor k arahi hain us ko behtar karein

AA: jee

RA: is k through hosakta hai koi aise kaam ho joh aap k ghar par hi hal hojaayein

AA: haan bas sahi hai bilkul sahi(laugh)

RA: toh who kaise karein kia mashwara de sakti hain

AA: aap log doctor hain aap logon ko ziada pata hoga

RA: acha chalo chalo thek hai aur koi cheez

AA: nahi nahi

RA: kaam k hawale se batana cha rahi hon

AA: nahi

RA: chalein thank you…..

Date: 27-01-2021

RA: Assalam o Alaikum

AA: Wa alaikum us salam

RA: aap a naam

AA: AA

RA: AA aap mujhe yeh bataaeingi k aap ki umar kitni hai

AA: meri umar 25 saal

RA: 25 saal hai acha aur aap ko yahan rehte huye kitna arsa hogaya hai

AA: mein yahan paida yahan pe huyi thi

RA: paida yahan huyi thi shadi bhi yahin hogayi hai

AA: jee

RA: acha thek hai acha yeh mujhe bataayein kitne bache hain aap k

AA: 3

RA: 3 bache hain aur sab se chota bacha kitni umar ka hai

AA: 4 saal ka

RA: 4 saal ka hai aur mujhe yeh bataaiye k aap ki taleem hai

AA: jee nahi 5 parhe thy is k baad nahi parhe

RA: 5 class tak toh parhi thi na naam toh likh sakti hain aap apna

AA: je

RA: acha sahi hai acha aaj na thora sa hum aap se health worker joh hamare aap k aate hain ghar us k hawale se baat kareinge thek hai acha mujhe yeh bata sakti ho k joh health workers hain community health workers hain hum CHWs kehte hain aa who aap k ghar par kitni baqaaidgi se aati hain

AA: who apne time se aate hain jaise hamein leine jana hota hai le k bhi jaate hain aur dawaai toh ab aise dawaai bhi de jaate hain

RA: hmm

AA: larkiyan jaise hafte le k jaati hain a k chor jaati hai a k poochti hain k aap ki tabiat kaisi hai kaise aap ka chal raha hai matlab jaise waqt pe a k kar jaati hain larkiyan

RA: lekin

AA: hamein un se koi shikaayat nahi

RA: acha thek hai aa joh phir bhi hafte mein aati hain do hafte mein aati hain ek maheeney baad aati hain

AA: har hafte aati hain hafte k baad a k poochte hain aise pooch taach kar k jaate hain hamein toh hum jaise aga khan center se bohat khush hain mera jaise pehla bacha hua tha who ulta tha na hamare bhi yahan kitne kaam hain hum hospital k kharche khud utha sakein toh jaise mera operation hua kaafi pareshan thy gaari k liye khoon k liye aise kharche hospitalon k liye bohat pareshan thy lekin ab pareshani nahi hoti nahi rehti k mashaallah aga khan chale jaayeinge hamara yeh saara masla hal hosakta hai toh us wajha se hum bohat khush hain

RA: acha mujhe yeh batao k ek hi worker aati hai aap k ghar pe ya alag alag workers aati hain

AA: alag alag aati hain

RA: acha

AA: mujhe leine k liye alag larki aati hai poochti hai who aur joh hai purriya wagera de rahe hain shaashey who alag aate hain poochne k liye who bhi waqt se de k jaate hain poochte hain aap k paas hai k nahi hai who alag se aate hain aur ek aur bhi hai larki who alag se aati hai

RA: who kia karti hai

AA: who bhi yehi poochti hai aap ka konsi aap ko date di hai checkup k liye

RA: hmm

AA: kab jana hai aap ka

RA: hmm

AA: a k poochti hai aur yeh shaashey alag lagate hain dawaon deine wale alag aate hain larkiyan

RA: acha aura cha jab who aati hain toh kia kia kaam sar anjaam deity hain kis hawale se sawal aap se poochti hain

AA: yehi poochti hai k aap hamal se hain apne khaane peene ka khayaal rakhna

RA: hmm

AA: jaise aap waqt se kaam karna koi bhaari cheez na utha leina aap k nuksaan k liye hosakti hai nuksaandida cheez toh aise aise batati rehti hain waqt se khana khao waqt se dawai leina poochte rehte hain

RA: hmm acha aur bache k hawale se maloomaat leiti hain

AA: haan

RA: kia poochti hain

AA: bache k liye bhi aati hain kehti hain bache ka apne paas teeka lagwaya bache ko 6 maheeney tak koi khaana wagera nahi deina hai in ko dood hi pilaana aise poochte hain a k

RA: acha yeh joh aap bache k hawale se poochti hain ya aap k hawale se poochti hain yeh bhi kia har hafte a k poochti hain ya yeh kuch maheeney do maheeney baad

AA: abhi yeh toh meri 4 saal ki bachi hogayi hai jin k chote bache hain ho sakta hai un k ghar pe aati ho abhi jaise hamare ghar pe chota nahi hai choti sab se meri beti who 4 saal ki hai

RA: hmm hmm

AA: ab jab se meri bhaabi hamal se thi toh us k liye who baar baar aate thy hamal walon ka pata hai jaise na toh ab yeh hamari bhateeji hai who hamal se hai bhaabi chali gayi toh ab yeh bhateeji hai who aate hain bachon wale jaise ab kitne time pe aati hai hota toh abhi mujhe pata nahi hai meri 4 saal ki bachi hogayi hai

RA: thek hai lekin aap k hawale se poocha toh koi jaise aap abhi hamal mein hain

AA: nahi abhi nahi

RA: abhi aap ka hamal nahi hai agar aap ka hamal nahi hai lekin 5 saal se chota bacha hai abhi 4 saal ka hai toh aap se milne k liye aati hain CHWs aap k hawale se poochti hain

AA: haan who aate hain na hamal ka joh poochte hain who kehte hain aap ko abhi hamal nahi hai

RA: hmm

AA: a k poochte hain

RA: hmm

AA: hamal se nahi hai mein ne kaha nahi hai abhi hamal se nahi hai toh phir chale jaate hain hamal wali hain un k paas aate hain joh hamal wale se un k paas aate hain baar baar a k poochte hain un ka khayal bhi rakhte hain yeh sab kuch

RA: thek hai acha aap kaisa mehsoos karti hain k jab yeh aati hain aur yeh apna kaam karti hain aap ko kaisa lagta hai

AA: hamein bohat khushi hoti hai k hamare mahaul mein yeh a rahe hain hamare liye yeh kaam kar rahe hain hum bohat khush hote hain in se

RA: hmm

AA: hamein koi shikaayat nahi hai un se

RA: lekin joh khushi hoti hai who sab se ziada kis cheez pe hoti hai

AA: who toh us pe hoti hai k mera pehle pehla operation mera waqt pe nahi hua jaise mein bohat paison ki wajha se pareshan thi toh mera bacha bach nahi saka jaise toh ab is baat ki khushi hai waqt pe hospital pohanch jaate hain waqt pe hamara kaam hojaata hai is baat pe khush hain

RA: hmm acha k bhae ab joh hai waqt pe aap apne center chale jaate ho doctor k paas chale jaate ho is baat se khushi hoti hai

AA: jee haan

RA: aur waise aapas mein kabhi bhi baat karte hain k worker a rahi hain yeh kaam kar rahi hain waise

AA: haan waise toh workeron se hamari dosti bhi hogayi hai

RA: acha

AA: who aate hain toh khush hote hain zahir si baat hai dost aayega toh khush raheinge dosti bhi hogayi jaan pehchaan bhi hogayi jaise aate hain pooch leite hain jaise yeh parveen kis kaam se aayi hai koi kaam hai toh batao waise hi poocha jaata hai in se

RA: hmm dosti kis wajha se huyi

AA: who hamara kaam kare waise aana jaana hota hai baar baar un k sath jaate thy baar baar un k sath jaate thy jaise who hamein pehchaan gaye hum in ko pehchaan gaye toh khush hote hain pehchaan wala log apne ghar agaya

RA: hmm

AA: toh khush hojaate hain

RA: acha aur pehchaan hone k baad kaisa lagta hai k aap ka joh kaam hai ya aap ki sehat k hawale se joh aap ki dosti hogayi hai us pe kia farq para hai us pe kia kia lagta hai k jab pehchaan gayi ho CHW ko toh us se farq para hai

AA: haan usay yeh para hai k pehchaan waale wahan agar aga khan jaati ho toh yeh pehchaan wale jaise AA pehchaan gayi hai toh us ne mujhe pehchaan liya AA kis kaam se aayi hai checkup karwaane aayi hai toh yehi hota hai farq pehchaan se

RA: hmm hmm

AA: toh wahan pe bhi tumhari pehchaan hai toh pehle se hi jaise

RA: hmm

AA: toh is wajha se pehchaan ka farq parta hai

RA: hmm hmm

AA: agar anjaan hota hai toh who nahi pehchaanta toh us se who hota hai na insaan wahan pe bhi boring lagta hai pehchaan wala mil jaata hai toh khushi hoti hai k yeh mujhe pehchaanta hai yahan pe

RA: hmm hmm acha in k kaam k hawale se aur kuch bata sakti hain aap k aur yeh kia kia karti hain aur aap ko kaisa lagta hai aap ki kaise madad hojaati hai un k kaam se

AA: jee who toh mein bata rahi hun hum bohat khush hain un k kaam se hamare madad ho paati hai toh hum na bahar aate hain na jaate hain center in logon k sath hi jaate hain phir ghar ajaate hain bas

RA; hmm hmm acha yeh mujhe bataayein aap ka joh aakhari hamal hua us mein joh CHW thi unhon ne aap ki madad ki

AA: jee haan meri yeh khoon k liye pehle pareshan hote thy khoon arrange kar liya us k liye paise bhi chaiye hote thy

RA: hmm

AA: toh un wajha se jaise mera joh hamal hua na operation hua toh us ka in logon ne khoon k paise in logon ne bhare thy keh rahe thy k aap log donor laao

RA: hmm

AA: toh hum dono bande sath mein le gaye thy khoon hamare band hone diye thy paise in logon ne bhare

RA: hmm

AA: toh us wajha se yeh bhi hogaya hamare liye toh us wajha se hum kuch deite k pehle jaise hum pareshan rehte thy toh ab sahi hai is wajha se

RA: toh aap yeh kehna chah rahi hain sab se ziada joh akhraajaat hain us ki wajha se CHW k zariye aap ka who cheez khatam hogayi

AA: khatam hogayi jaise pehle hum pareshan rehte thy k hamare paas paise nahi hai hamare meeyaan ki kamaai nahi hai toh yeh hum operation ka kharcha kaise le sakte hain gaariyon k jaise time pe 2000 gaari leiti hai hospital le k jaane mein

RA: hmm

AA: toh who waqt pe nahi hote toh insaan pareshan rehta tha ab who pareshani nahi hai ab gaari time se mil jaayegi

RA: hmm

AA: jaise hum bulaate toh mil jaayegi gaari bhi na toh pareshani pehle khoon wala masla hota tha ab who bhi sahi hai jaise

RA: toh yeh khoon ka masla gaari ka masla kis ko bataate ho

AA: uzma ko

RA: uzma ko batate ho acha us k zariye

AA: who kehti hai k jis time bhi koi bhi masla ho mujhe call kar deina

RA: thek hai phir who ajaati hai gaari

AA: a jaati hai aur jab bhi bulaao ajaati hai

RA: thek hai acha yeh bataayein aur bachon k hawale se joh aap ka chota bacha hua us mein CHW ne koi madad ki ho a k

AA: haan yehi na mera jaise pehla bacha mera har masla har cheez pe jaise who yaad aata hai na apna bacha toh who normal nahi tha jaise toh hamare paas itne akhraajaat nahi thy jaise dusre hospital le k jaaye or NICU mein rakhe bache ko toh who nahi kar sake hum waise toh ab joh meri bachi paida huyi thi woh 11 din k liye NICU mein rakhi uzma ne rakhwaai thi wahan pe

RA: hmm

AA: private hospital tha wahan pe rakhwaai thi phir is ka kaafi elaaj hua wahan pe jaise ab bachaya hai toh Allah ne hi bachaya lekin kisi na kisi wajha se bachi us ka waqt pe elaaj hua bachi ka yeh

RA: hmm

AA: toh unhon ne wahan pe rakhwaya bachi jaise paida huyi toh who usi bete ki tarhan thi jaise hai na mujhe bolte hain aap k pait mein paani kam hota hai bacha jaise ghoom nahi sakta toh us wajha se normal hota hai mera toh us wajha se pehla bete ko bhi aise kaha tha k demag ki haalat sahi nahi hai demag k tawazun sahi hai behtar nahi hai toh us wajha se fout hogaya phir beti huyi thi who itni kamzor itni k jaise usay NICU mein rakhwaya toh us k baad jaise phir baad mein joh elaaj hota tha yeh log khayal karte thy le k jaate thy kahin pe doctor bolte thy toh wahan pe le k jaate thy toh un wajha se jaise hamari kaafi madad huyi yahan se

RA: hmm hmm acha joh bacha hua tha us waqt center CHW nahi aaya karti thi aap k paas

AA: nahi tha who 14 mein hua tha 2014 mein

RA: hmm hmm

AA: tab nahi tha

RA: tab nahi tha aur yeh kab se behtar hua yeh ziada CHWs aane lag gayi

AA: 15 mein aaye yeh log

RA: hmm

AA: vital Pakistan wale joh yeh aaye huye hain na aga khan center 2015 mein aaye huye hain jab mein hamal se thi na

RA: hmm

AA: tab yeh aaye huye hain pehla joh mera beta hua tha yeh log nahi thy yahan pe aga khan mein

RA: aur us waqt aap kahan gaye thy

AA: us waqt bhi kohigosh ka pata laga na toh wahan pe hum log gaye thy

RA: joh beta hua tha

AA: beta hua tha tab bhi wahan pe gaye thy lekin jaise apne kharche se gaye thy gaari li khoon ka bandobast kiya aur baaki bhi kharche aaye ab jaise yeh le k jaate hain us pe checkup k liye ultrasound ka yeh karo who khud hi le k jaate hain Khalid hispatal mein le jaate hain attiya mein le jaate hain toh un ka kharcha hota hai jaise

RA: hmm

AA: gaari bhi deite hain aur le k bhi jaate hain

RA: hmm

AA: pehle nahi tha waise pehle yeh nahi tha jab mera beta hua tha

RA: hmm hmm acha toh aap ne kaha k joh matlab hamal k doraan koi masle masaail hote hain toh ab aap jab bhi center pe pohanch jaati hain CHW k zariye phone kar deite hain number pata chal jata hai gaari ajaati hai who aap ko le k chale jaate hain akhraajaat kam hogaye hain gaari ka kharcha nahi karna parta yeh madad aap logon ki huyi hai jis se aap ko acha mehsoos hota hai

AA: acha mehsoos hota hai is wajha se hamein acha mehsoos horaha hai

RA: haan

AA: k ab jaise hamari delivery ka masla hamare upar nahi rehta

RA: hmm hmm

AA: ab pareshan nahi rehte pehle yeh gareeb log hain kahan se kareinge kahan se aayega is wajha se ab yeh pareshani kam hogayi

RA: hmm hmm acha yeh mujhe bataayein k koi cheez aisi hai joh kabhi dil ko buri bhi lagi ho

AA: aisa toh hum nahi mehsoos karte kabhi

RA: kabhi kuch bhi joh bhi hua kisi aur k sath kabhi kisi se sun liya ho ya kuch aisa hua ho ta k hum us ko behtar kar sake

AA: nahi nahi who hamare jaise zameer gawaahi nahi de raha who log hamare liye itna kuch karte hain itni si koi wahan pe mistake hojaati hai toh who hum bata dein toh who hamara zameer gawaahi nahi kabhi kabhi hojaata bhi hai pareshan wahan se agar gaari ka masla hota hai toh chale jaate hain bahar un ka kaam hai gaari chali gayi wahan pe chali gayi toh wahan pe beithna parta hai pareshani hoti hai ghar nahi gayi ghar pe khana kon banaayega yahan pe deir hogayi aise toh kabhi masle ho bhi jaate hain lekin itna saara who hamare liye kar rahe hain aur hum yeh itni si baat agar tang kareinge toh jaise acha nahi lagta hamein na who log hum pehle kitne pareshan thy aur us pareshani se nikle toh itni si baat pe hum bhi toh bardaash kar sakte hain

RA: haan lekin

AA: toh pareshan us wajha se rehte hain kabhi kabhi wahan se jaise gaari nahi milti toh ghar pe pohanchna hota hai time zaaya hogaya ghar pe shohar daanteinge khana nahi banaya wahan pe bohat deir lage is wajha se kabhi pareshan bhi hojata hai yeh toh hum nahi keh sakte yeh kyun hota hai hota hai itne saare kaam mein logon k upar bhi zimedaariyan hai kabhi kar bhi sakte hain lekin joh hamare pareshaniyan hain us time pe hamare sath who thy

RA: hmm hmm lekin poochne ka sirf yeh hai k hum behtar kar sake na

AA: jee

RA: thek hai

AA: toh who us wajha se hota tha

RA: acha kabhi aise toh gaari ki baat ho kabhi worker ki koi baat ya worker se madad na mili ho kabhi aisa hua aap ne hamesha jab bhi apni baat bataai unhon ne ache se sun li aap ki madad ki

AA: who karti hain

RA: kaise karti hain

AA: who log aate hain yahan pe jaise hamara kaam hota hai toh hamein le k jaate hain wahan pe chor deite hain wahan pe to humara kaam checkup karana beithe karein

RA: hmm

AA: un ka toh kaam hota hai le k jaate hain

RA: hmm hmm hmm

AA: phir ghar pe a k chor jaate hain un se koi shikaayat nahi

RA: who aap ko le k chali jaati hain aur ghar a kar aap ko chor deity hain

AA: jee

RA: toh yeh un ka kaam horaha hai aap ko lagta hai sahi tareeke se kaam kar rahi hain

AA: haan

RA: acha koi aisi cheez joh aap chahti hon worker k through hum kara dein ya hojaaye aap ki madad hojaayegi us se ya aap behtar hojaayega aap k liye aur koi asaani hojaayegi aisa kuch hai joh aap ko lagta hai

AA: mujhe toh aisa nahi lagta jab mein hamal se hojaaongi tab pata lagega worker ka (laughing)

RA: acha yeh mujhe bataayein k worker aati hai hamal k doraan who aap ko bataati hai yeh sirf aap se poochti hain ya kuch bataati bhi hain

AA: han bataati bhi hain

RA: hmm

AA: kehti hain waqt se khana khaao waqt pe dawaai lo apna khayal rakho pehle jaise aap nahi hai aap k sath dusri jaan bhi hai toh aap ko dono cheezon ka khayal rakhna hota hai

RA: thek hai

AA: toh who bataate bhi hain a k jaise dawaai wagera pehle de deite thy kehte thy yeh le lou time pe kha leina yeh ab kehte hain aap log time pe nahi leite ho jaise hum khud a k aap ko de deinge

RA: hmm hmm

AA: ab khud a k de deite thy jaise hamari thin a bhaabi ab who chali gayi hai wahan pet oh kitna kharcha hai delivery nahi huyi operation hua is ka emergency mein

RA: hmm

AA: pehle wahan pe doctor keh rahe thy aap ka normal hojaayega aur jab us ko foran hua na dard toh us doraan unhon ne keh diya is ka nhi hoga bacha mashaallah is ka sehatmand hai bache ko pata nahi haddi ka masla joh bataate hain who hai us ka nhi hoga us ko le k jao operation karwaao toh un doctoron ne nikaal diya phir le k gaye emergency 45000 rupey liye un logon ne

RA: hmm hmm hmm

AA: us ka scissor hua hai aaj who ghar chali gayi hai hospital se

RA: acha mujhe yeh batao k hamal k elaawa agar aap hamal se nahi ho toh tab who aap se poochti hain ya tab aap ko sehat k hawale se koi baat bataati hain

AA: nahi tab toh nahi poochte bas yehi poochte hain k aap mein se koi hamal se hai agar hai toh hamein foran se bata deina

RA: hmm hmm hmm

AA: bas aise nahi poochte

RA: nahi poochte is k elawa k bhae abhi bacha hogaya hai us k baad aap ko kuch bataati hain

AA: nahi

RA: nahi bataati ya matlab bacha ek saal ka hai do saal ka hai toh koi aap ko us k hawale se maloomaat deity hon bache k hawale se

AA: yehi bataate hain k bache ka khayal rakho bachae ko waqt pe khana peena khilaao is ko hifaazati teeke lagwaao k jaise bimaariyaan nahi hoti jaise hote thy pehle bachon ko ab nahi hote kam hote hain

RA: hmm

AA: toh yeh karo bachon ka khayal

RA: aur waqfey k hawale se kuch bataati hain kab baatati hain waqfey k hawale se

AA: yehi jab hamal se hote hain tab bhi kehte hain kehte hain waqfa karwaaogi toh aap ki bhi sehat ka jaise bache ki bhi sehat ka agar bacha baar baar rota rahe toh pehle wale bache ka khayal nahi rehta maa ko

RA: hmm hmm hmm

AA: agar waqfa karogi toh aap k bache ki sehat ki baat hai aur apni bhi sehat ki baat hai baar baar maa hamal se hoti hai toh kamzor par jaati hai un k ghar ko bhi deakhna hota hai bachon ko bhi deakhna hota hai aap log agar waqfa karwaaoge aap ki hifaazat hojaayegi toh yeh log bataate hain

RA: aap amal karte ho un baaton pe

AA: karte hain kisi pe karte hain kisi pe nahi karte (laughing)

RA: acha kon hai aisa lagta hai konsi worker aisi hai joh lagti hai joh aap ko waqfe k baare mein bataaye hamal k doraan cheezein bataayein aap maan leite ho haan yeh keh rahi hai toh maan leite hain

AA: nahi worker yehi yahan pe nahi aati thi raheela parveen

RA: hmm hmm

AA: yeh dono mein jab hamal se thi yehi aate thy hamare paas baat karne k liye toh ab bhi larkiyan aa rahi hain who bhi mashaallah se sahi hai jaise who aati hai salma yahan pe shaashey deine

RA: hmm hmm

AA: who bohat pyar se baat karti hai who mujhe achi lagti hai bohat

RA: acha kia cheez achi lagti hai

AA: who bohat pyar se baat karti hai jaise na

RA: hmm

AA: who achi lagti hai baat karna us ka bohat acha lagta hai

RA: hmm aur kia cheez aisi hai

AA: mujhe toh who workeron se who wahan pe sister beithi huyi hai sister arsla

RA: hmm

AA: who bhi bohat achi mujhe who lagti hai who meri dost hai

RA: acha thek hai kyun achi lagti hai

AA: abhi jab bhi jaon na

RA: hmm

AA: toh mein un se zaroor milti hun dua salam karte hain kehti hain ab aap hamal se nahi ho mein ne kaha nahi (laugh)

RA: matlab us ki baat pea mal kiya hua hai

AA: haan

RA: acha waqfa liya hai aap ne

AA: haan toh kehti hai waqfa karo baar baar tumhare scissor horahe hain

RA: hmm

AA: waise toh bache bhi sahi nahi hote na toh un k liye bhi tum pareshan rehti ho joh bar bar scissor hota hai toh tum waqfa karwao waqfa mein pehle jaise challa joh rakhte thy na who rakhwaya that oh us k baad mein sahi nahi rahi thi jaise takhno mein dard hogaya 25 saal meri umar hai 70 saal k baad bhi yeh dard hota tha pehle ab 25 saal mein yeh haal hogaya hai hamara abhi yeh kapre dho k uthungi na phir in ko beith nahi sakti

RA: hmm

AA: phir toh khana bhi banana hota hai yeh who jaise paani bohat zaaya hua tha

RA: acha toh yeh baat aap ne bataai apni worker ko k jab se mein ne yeh rakhwaaya hai toh mujhe

AA: haan unhon ne kaha hosakta hai k sahi nahi ho toh phir aap ja k wahan dikhaao mein ne doctor ko wahan pe dikhaaya tha toh phir unhon ne nikaal diya

RA: thek hai phir us k baad

AA: us k baad phir hamal hogaya tha phir yeh bachi paida huyi us k baad ab nahi

RA: abhi bhi waqfa liya hua hai aap ne

AA: abhi nahi hai

RA: ab waqfa nahi kiya hua

AA: nahi

RA: thek hai acha yeh mujhe batao

AA: abhi na mere ko gurdey mein pathari toh us ki joh dawaai wagera le rahi hun na toh us wajha se joh maahwaari hai who teiz aati hai

RA: hmm

AA: who teiz aati hai toh mein ne kaha tha ek baar yahan pe ja k aga khan mein dikhaaongi abhi gayi nahi hun jaise mein ja k dikhaongi mein ne wahan ultrasound karwaaya tha na

RA: hmm

AA: toh unhon ne kaha k aap joh lady doctor hai us ko bhi dikha dou

RA: toh yeh mein poochna chah rahi thi jaise aap abhi hamal se toh ho nahi thek hai aap k sath masla toh hai na kisi hadh k upar toh farq parta hai

AA: mujhe kaha tha us doctor ne hai na us ne kaha k beta hamal se ho mein ne kaha hamal se nahi hun toh mahwaari ka kaha who har maah aati hai toh phir us ne kaha k aapka koi masla hai bacha daani ka aap ja k lady doctor ko dikhao ja k

RA: hmm

AA: mein joh hun na darti bohat hun hospitalon se (laugh) ab is wajha se mein gayi nahi jaongi mein ek baar sister aslaan se baat karongi who hosakta hai ultrasound karwa k de dein

RA: hmm

AA: toh us ne mujhe kaha that oh mein abhi tak gayi nahi hun

RA: kabhi aap ko is k hawale se koi maloomaat di hon joh CHWs ne is k hawale se k mahwaari ziada kyun arahi hai aap ko yay eh saare masle us mein kabhi kuch bataya aap ko aap ne un ko bataya

AA: nahi mein ne un ko nahi bataya

RA: acha wajha kia thin a bataane ki

AA: yehi keh rahi hun k mein doctoron se darti hun waise mujhe dar lagta hai bohat kuch bol na dein bas yeh gurdun mein takleef thi is wajha se mein wahan pe gayi who ultrasound karwaaya ab unhon ne kaha civil hospital chali jao

RA: hmm

AA: toh nahi jaati mein wahan pe

RA: kyun

AA: dar lagta hai bohat wahan pe jana toh keh rahi hai gurdey mein pathri hai gurda barhte barte kharab hojaayega

RA: hmm

AA: toh phir kia karongi mein mein ne kaha joh Allah ki marzi mujhe bohat dar lagta hai

RA: acha

AA: jab bhi dard hota hai painkiller ki koi injection laga leiti hun ya tablets le leiti hun

RA: haan

AA: aur abhi tak gayi nahi bas ek baar takleef huyi na toh wahan pe gayi thi ultrasound kiya

RA: lekin aisa kabhi hua k kyun k unhon ne aap se poocha nahi health worker ne toh aap ne unhein bataya nahi k who joh aap se poochti hain aap sirf un ko wohi batati ho

AA: wohi batate hain joh who poochte hain wohi bata deite hain mein toh hamal se nahi hun joh un ko bolon yeh hua yeh hua

RA: lekin aap yeh nahi sochti k agar mein hamal se nahi bhi hun toh tab bhi apna agar mujhe sehat k hawale se masla hai

AA: toh who keh rahi hun na mein wahan pe jaane se dar rahi hun ab jaise baar baar kehti hun koi jaayegi na us k sath chali jaongi mein

RA: hmm

AA: sister aslan se yeh baat kahongi phir kehti hun nahi nahi phir pata nahi kuch masla bata de toh

RA: hmm

AA: toh phir agar masla hoga toh doctor hai aap ko goliyan deinge koi wajha bataayeinge chali jao wahan pea mmi bohat daanti lekin nahi jaati wajha yehi aap ko bata rahi hun lekin nahi jaati mein is wajha se

RA: hmm hmm acha ek wajha toh aap ne bataai k who aap khud darti hain toh mein agla yehi sawal karti hun aisi kia wajha hai jis ki wajha se aap joh batana chahti hain apni koi maloomaat deina chahti hain CHW ko who aap nahi bata paati ek toh bata di aap darti hain aur is k elawa

AA: toh yehi bas us k elawa kuch nahi mujhe khud apna jaise dar lagta hai doctor se mein darti hun waise

RA: hmm hmm

AA: bohat jab mera akhari scissor hua tha na toh us mein mera saans bohat phul raha tha jitna theater mein dar laga

RA: doctoron se kyun dar lagta hai

AA: yehi bas doctor k paas nahi jana chahti (laugh)

RA: kia kuch hua hai kabhi

AA: nahi

RA: phir kyun dar lagta hai

AA: yehi k theater mein bohat dar lagta hai

RA: acha

AA: haan salatein parh parh k do baar toh behonsh hogayi thi pehle mein jaise bohat kar diya tha na jab pehla scissor hua tha tab toh doctor keh rahe hain kuch nahi hoga tum kyun itna dar rahi ho abhi tumhara bacha hojaayega abhi tum khush rahogi deakhna beta kyun dar rahi ho beta apna BP barha rahi ho yeh kare bohat jaise mere sath baatein kare lekin BP pata nahi kaise upar chali gayi phir un logon ne injection lagaya tha shayad

RA: acha mujhe bataayein k ek toh aap ne kaha dar hai aur toh koi masla nahi hai ek yeh bhi shayad aap mujhe bataayein who joh sawal aap se poochti hain thek se jawab deity hain

AA: jee

RA: is k elawa aap khud se bataane ki koshish nahi karte

AA: nahi khud se jaise hamal se hote thy toh karte thy un se baatein yeh hua yeh hua yeh hua hamal se nahi hun toh nahi karti mein unse baat

RA: toh aap ko aisa lagta hai k who aati hain sirf joh hamal se aurtein hain un ka kaam sirf yehi hai k hamal ki joh aurtein hain un ko deakhein unse baat karein un ko maloomaat dein ya joh

chote bache hain nauzaida bache joh hote hain unse baat karein aur aap aap k liye joh agar hamal se nahi hai lekin aap 25 saal ki hain thek hai choti umar hai aap ki aur aap k 3 bache hain aap ko bhi sehat ka masla hosakta hai thek hai toh aap kabhi nahi sochte k aap un ko bata do who arahi hain unse koi mashwara kar lo

AA: hum ne nahi kiya jaise hum ne nahi bataya hamare sath yeh masla hai

RA: kyun zaroorat nahi

AA: wohi bata rahi hun na k bas agar un ko masla bata do who kaheinge chalo wahan pe aap ko doctor bataayeinge toh us wajha se nahi bataati

RA: acha is mein koi yeh baat toh nahi matlab like akhrajaat barh jaayeinge maali who hojaayega who dart oh nahi hai

AA: toh yeh toh hai k kahin phir khuda na khuwsta koi dusre hospital bol dein aap ko bacha daani ka masla horaha hai kahin dusre hospital chale jao yeh karo yeh hojaaye is wajha se hamein dar lagta hai hum nahi jaate

RA: lekin sehat bhi toh hai na agar who aap ko bataayeinge aap ko nahi lagta us mein aap ka faida hai

AA: who toh hai mera hi faida hai lekin mein bata rahi hun doctoron se dar lagta hai bohat ab joh hoga khuda ki karni leikin dar lagta hai nahi jaati is wajha se

RA: acha aisa bachon k baare mein bhi sochti hain kabhi bache ko kuch hua tab bhi nahi bata sakti

AA: nahi bachon ko Allah na kare bachon ko kuch hojaaye th foran bacha le jaati hun

RA: CHW ko bata deity ho

AA: haan un ko bata deite hain ya hum khud hi se chale jaate hain

RA: acha joh hamari workers aati hain in pe kitna aitebaar hai aap ko

AA: yeh who sahi hai hamare liye kaam kar rahi hai hamare liye kar rahi hain hamare bhale k liye kar rahi hain toh hum kehte hain hamari sehat k baare mein who bhi karein

RA: aur kia wajha hai aitebaar karne ki kia cheez aisi lagti hai joh aap ko lagta hai k haan yeh aitebaar k qabil hai

AA: toh hum jaise mein ne aap ko dusri yeh baat bataai hai k hum pehle bache se mein dari huyi hun ab mera bacha bach gaya foran se operation hogaya do baar hum ne operation karaaye toh ab un pe mera isi wajha se bharosa rehta hai k insaan ko waqt pe le k pohanchte hain

RA: hmm

AA: toh is wajha se un logon pe bharosa rehta hai toh hum bhi unse acha sulook mehsoos karte hain who bhi hamare sath toh hamein unse koi shikaayat nahi

RA: acha kal ko kabhi nayi CHW aayi ya na aayi joh aa rah hain thek hai aap k elaake ki na hon us ki zaban bhi nahi ho aap ki tarhan kia tab aap tab bhi bharosa kar k apni saari cheezein bata doge

AA: yehi toh hum ko aisa lagta hai hamare liye kar rahe hain who bahar se aaye chahe hamare elaake ka ho hamare liye who sab kuch kar rahe hain na hum isi wajha se bharosa karte hain

RA: matlab elaake ka hona zaroori hai

AA: nahi elaake ka toh phir bhi sahi rehta hai thora leikin bahar wale bhi sahi hai yeh joh salma bata rahi hun who hamare elaake ki nahi hai bahar ki

RA: hmm aur us ki kia baat achi lagti hai

AA: who bohat pyar se baat karti hai isliye acha lagta hai

RA: acha acha is k elawa aur kuch batana chahti ho k hum behtar kar sakein itni achi achi baatein aap ne ki hain hum is kaam ko aura cha karna chahein

AA: yeh hai

RA: acha aap ka beta hai

AA: beta nahi beti hai (laughing)

RA: (laugh)

AA: sab beta hi kehte hain bahar jaate hain toh kehte hain aap ka beta hai mashaallah mein ne kaha beti hai(laugh)

RA: acha acha tabhi baal aise hain haan toh mashaallah se pyaari hai toh aap is ko aura cha kaise karein kuch mashwara dein hamein kaise hum CHW k kaam ko acha karein aur faidemand hojaaye kia koi cheez hai aap k zehan mein aisi

AA: mujhe toh aisa kuch nahi araha joh mein keh sakon mere liye joh tha unhon ne who behtar kiya toh mujhe wohi acha laga

RA: hmm

AA: mujhe nahi aata k aap ko koi mashwara de sakon unse bhi behtar karein aur yeh k hai na khoon ka joh karte hain bandobast kehte hain k donor laao hamare joh log hai na who toh wahan pe chale jaate hain bahar jaate hain kashtiyon mein bechaare hain na who kabhi time pe hote hain kabhi nahi hote toh us wajha se bhi hota hai pareshan insaan rehta hai hamare liye toh un logon ne hamare sath bohat acha kiya jaise paise bhi un logon ne bhare toh hum ne kaha aap donor le k aao hamare sath us time pe bande thy toh hum le k gaye kabhi kisi k paas nahi bhi hote log toh us wajha se bhi insaan hota hai hum chahte k wohin behtar hojaaye

RA: hmm

AA: k khoon khud hi se wohi de dein

RA: hmm hmm hmm

AA: is wajha se bhi hota hai kabhi hai na

RA: k kabhi aisa hojaaye un k liye bhi donor nahi milte jana pare kuch behtar hojaaye

AA: haan yehi hona chaiye

RA: acha chalein shukriya bohat bohat AA aap ka……………

RG-IDI

RA: acha samina mujhe yeh bataayein k aap ki umar kitni hai

SS: meri umar 29 saal hai

RA: 29 saal hai aur aap yahan kitne arsey se yeh rahe hain

SS: 13 saal

RA: 13 saal se reh rahe hain aap ki shadi ko kitna arsa hua hai

SS: 12 saal

RA: 12 saal hogaye hain

SS: jee

RA: kitne bache hain aap k

SS: 4

RA: 4 bache hain aur sab se chote bache ki umar kia hai

SS: 2 saal

RA: 2 saal hai acha mein na ab aap se parhi likhi hain kuch kitni jamaatein parhi huyi hai

SS: 5

RA: 5 tRA parji huyi hain

SS: jee

RA: acha aur ghar ka kaam karti hain

SS: saara kaam

RA: is k elawa koi hunar aata hai aap ko

SS: jee silaai bhi kar leiti hun

RA: acha ailaai kar k dusron log ki silaai karti hain

SS: nahi ghar mein

RA: ghar k silaai acha acha .. acha ghar mein guzaara karne k liye si leiti ho ya aise hi

SS: bachon ki silaai kar leiti hun shohar ka kaam toh sahi nahi hai guzara hai

RA: acha acha sahi acha mujhe yeh bataayein k hamari health worker aati hain aap k ghar

SS: jee

RA: thek hai kitne arsay mein aap k paas aati hain

SS: tRAreeban bete k time jab meri delivery thi tab toh aati thi haftey mein ajaati thi delivery k baad maheeney k baad do maheeney k baad aajaati thi woh bas baahir se pooch k chali jaati hain bas

RA: acha toh jab woh aati hain toh kia poochti hain

SS: bas yehi number likh leiti hai shanRAati card number likh k chali jaati hain

RA: aur is k elawa

SS: kuch bhi nahi

RA: aap ki sehat k hawale se

SS: nahi

RA: k abhi hamal se hain is tarhan ka kuch

SS: haan yeh poochti hai k hamal se hain k nahi mein bolti hun nahi

RA: nahi hai acha aur bache k hawale se

SS: nahi

RA: nahi poochti hain aur bache ki sehat k hawale se koi sawal karti hai

SS: nahi nahi

RA: acha toh jab yeh aati hain aur apna kaam kar rahi hoti hain aap se pooch k jaati hai toh aap kaisa mehsoos karti hain aap ko kia lagta hai

SS: mein yeh mehsoos karti hun k hamal ka poochne aayi hongi yehi bol k chali jaati hai unko jawab de deity hun aage se chali jaati hai

RA: acha bas aap ko yeh lagta hai woh aati hain toh hamal ka poochne aati hain

SS: jee

RA: is k elawa aur kuch bhi nahi

SS: nahi yeh teekon ka pooch liya hifazati teeke bachon ko lagaaye hain k nahi

RA: hmm

SS: toh kehti hun laga liya bas chali gayin

RA: kaisa lagta hai aap ko in ka kaam

SS: kaam toh mashaallah in ka acha hai ghar k andar a k pooch k chali jaati hain inka bohat bara ehsaan hai aur bas yehi joh hai

RA: kyun aap ko aisa lagta hai k yeh ehsaan hai acha aap ko kaisa lagta hai k joh inka kaam hai yeh joh poochti hain aur aap keh rahi hain ehsaan lagta hai yeh bhi ehsaan hai hamare upar kyun lagta hai k ehsaan hai

SS: acha kaam kar rahe hain na dusre insaan ka joh hai khayal karte hain is wajha se insaan ko acha lagta hai acha mehsoos hota hai

RA: acha aap ko lagta hai k yeh aap ka khayal kar rahi hain

SS: jee

RA: kis tarhan se

SS: ghar k andar a k le k ja k phir le ja k unka ehsaan hai us hisaab se achi baat hai ek musalmaan dusre musalmaan ka khayal rRAheinge toh bohat achi baat hai

RA: acha toh matlab aap ko lagta hai k ghar arahi hain aap se pooch rahi hain yeh achi baat hai yeh a k poochti hain

SS: jee

RA: acha toh mujhe yeh bataayein k jab aap aRAhari dafa hamal se huyin thi toh tab is mein worker ne joh CHW aati hai us ne koi kirdaar ada kiya tha

SS: nahi yeh bas pooch taaj karne aati thi phir chali jaati thi bas

RA: woh kia poocha karti thi

SS: woh yehi kehti thi k aap ka time abhi tRA hai joh mein pooch rahi thi joh masla hota tha joh masla ho hamein bata dou bas pooch taaj kar k chali jaati thi

RA: aur is k elawa

SS: nahi aur kuch bhi nahi

RA: acha aap inse woh karti hain tawwRAul k yeh arahi hain woh hum se sirf pooch k ja rahi hain aur kuch nahi

SS: woh yeh kehti thi hafte k baad aayeinge khud bol k chali jaati thi hum aayeinge toh aajaati thi

RA: aati thi phir kia karti thi

SS: bas yehi pooch leiti thi mere se aap ki tabiyat kaisi hai kaisi nahi hai mein ne bol diya sab thek thaRA

RA: acha koi faida lagta hai aati hain aap k ghar par aap se poochti hain

SS: haan mein ne bol diya yeh faida hai pooch leiti hai

RA: hmm

SS: ache tareeke se pooch leiti hai wohi faida hai

RA: kyun kyun aap ko aisa lagta hai yeh faida hai aap ki sehat se koi is se faida mil raha hai

SS: faida yehi tha k mashaallah aap k pooch leite hain yehi faida hai

RA: acha bas yeh faida hai

SS: dawaai wagera de deite hain

RA: deite hain aap ko dawaai

SS: jee

RA: konsi dawaaiyaan deite hain

SS: wahan jab chali jaati hun rehri goth dawaai wagera joh jaise tabiat hai dawaai wagera goli wagera de deite hain

RA: acha woh center pe deity hai worker deity hai aap ko koi dawaai

SS: nahi woh nahi deite

RA: yeh poochte hain na

SS: jee

RA: acha .. acha joh aRAhari bacha chota wala kitna 4 saal ka hai

SS: 2 saal

RA: 2 saal ka hai jab aap us waqt hamal se huyi thi toh worker wagera aaye thy aap k paas

SS: jee

RA: poocha tha unhon ne aap se

SS: jee

RA: acha

SS: jab yeh mera beta hua tha

RA: hmm

SS: tab nahi aayi thi phir nahi aayi

RA: us k baad nahi aayi

SS: nahi aayin bache ka poochne wagera kuch bhi nahi pehle aaye thy pehli beti pe bete pe nahi aaye

RA: acha yeh kitne arsey ki baat hai

SS: beti ko toh mashaallah 4 saal 6 maheeney hogaye hai na meri beti ko

RA: hmm

SS: beta us ki 2 saal umar hai us ka poochne nahi aayi

RA: kuch bhi nahi kiya is ka

SS: nahi kuch nahi.. sirf yeh poti wagera check karne jab aate keh rahe thy k yeh jaraseem k liye must hai aap logon ko pata bhi hoga

RA: hmm

SS: poti wagera check karne aate thy woh le k gaye 2 3 baar aaye le k gaye wahan pe check kiya bas aur kuch nahi

RA: aur bache ka koi sehat k hawale se

SS: nahi

RA: koi kirdaar ada nahi kiya worker ne

SS: nahi

RA: acha pehli bachi mein aap keh rahi thi woh aayin thi unhon ne kia kiya tha

SS: woh bas yehi poochti thi

RA: haan

SS: dawaai wagera bukhaar wagera check kar k chali jaati thi

RA: acha bache ka wazan wagera kiya

SS: haan wazan bhi karti thi

RA: is ka kia chote ka

SS: is ka nahi kiya tha

RA: wazan wagera bhi nahi kiya

SS: nahi

RA: acha

SS: aayi nahi thi

RA: phir nahi aayin kitna arsa hua nahi aaye

SS: jab meri delivery huyi bas us k baad 1 2 dafa kitne arsey baad aaj aayi

RA: hmm

SS: bohat arsey baad aayin

RA: aaj aayin thi

SS: jee

RA: acha .. acha mujhe yeh bataayein k yeh aap keh rahi hain itne itne arsey baad arahi hain phir aati hain tab aap in ko araam se apni baat joh woh poochti hain aap bata deity hain

SS: jee

RA: bata deity hain aap inse poochti nahi itne arsey baad kyun arahi ho

SS: woh unki duty hai wahan pe time lagta hoga shayad koi na koi masla hoga jaise apne time se aati hain kehte hain wahan pe computer mein pata lag jaata hai k yahan mein jana hai aajaati hain

RA: yeh aap ne unse poocha unhon ne aap ko jawab diya is baat pe k un ko jab pata chalta hai computer se deRAh k idhar jana hai toh ajaate hain toh aap mutmaain hoti hain un k jawab se

SS: jee

RA: aap ka dil karta hai aap k ghar aayein aap se poochein

SS: dil toh karta hai

RA: hmm

SS: isi wajha se bitha deity hun baat kar leiti hun dil nahi karta toh kahan bithaati

RA: kia wajha hai k aap in pe aitebaar kar leiti hain

SS: wajha yeh hai bas mein ne aap ko bata diya hamari hifazat karti hai khayal karti hai

RA: hmm matlab kia cheez in ki achi lagti hai yeh mein poochna chah rahi hun

SS: kia cheez achi lagti hai

RA: hmm

SS: doctor joh insaan ka elaaj karein woh cheez achi lagti hai aur kia

RA: matlab yeh aap ko lagta hai

SS: achi lagti hai beithna uthna unka

RA: hmm

SS: jana aana dusron ki tRAleef uthana yeh inka acha hai aur kia

RA: inka ikhlaaq acha lagta hai

SS: mashallah ache hain bohat ache hain badtameezi wagera koi aise faltu baat wagera kuch bhi nahi apne kaam se kaam rRAhna

RA: acha yeh saari cheezein aap ko achi lagti hain.. acha yeh aap ne kaha k yeh aati hain ache se baat karti hain uthna beithna inka thek hai aap ko sahi lagta hai apne kaam se kaam rRAhti hain

SS: jee

RA: yeh saari cheezein pasand karti hain is ki wajha se aap ko in pe bharosa hai aitebaar hai acha yeh mujhe batao koi cheez hai aap ko lagta hai hamein inki behtar banaaney ki zaroorat hai kuch acha karne ki zaroorat hai

SS: aise toh mein ne socha nahi hai

RA: kyun nahi socha (laugh) kia wajha hai k aap ne kabhi is baare mein socha nahi aap unko sirf data wagera share karti hain unhein bataati hain toh aap ne kabhi socha yeh hamare ghar a hi rahi hain inko yeh cheez bata deite yeh hamein is baare mein maloomaat de dein

SS: aaj toh mein ne poocha tha aaj aayi hain mein ne poocha bhi hai k bay form aap k yahan bante hain k nahi bante toh unhon ne kaha bante hain do bachon k nahi bane toh bana dun school jaate hain aage daRAhila nahi hota

RA: hmm

SS: bay form joh k chaiye hota hai toh mein ne socha is se poochun yahan pe banta hai nazdeek bhi hai aana jaana bhi asaan hoga chale jaayeinge bana dein lekin unhon ne koi english mein baat kahi mujhe samjh nahi aayi

RA: aap ne jawab nahi diya

SS: nahi mujhe pata hi nahi laga

RA: acha yeh aati hain toh aap interest leiti hain k yeh arahi hain matlab aap ki tawajja hoti hai in k kaam ki taraf ya bas arahi hai pooch k ja rahi hain choro

SS: tawajja toh deina parta hai kyun k aaj kal aise nazuk daur hai insaan sochta kuch aur hai aur banta kuch aur hai

RA: hmm

SS: bohat naazuk daur hai un pe aitebaar hai isi wajha se unhein bitha k pooch k bol k

RA: in pe aitebaar koi aisi baat huyi hai joh aap ko achi lagi ho

SS: lekin bohat log aiteraaz karte hain k aise logon ko nahi andar chora karo

RA: hmm

SS: lekin nahi hum logon ko pata hai hum log aate jaate hain hum ko pata hai elaaj hum ne kiya hai hum logon ko pata hai isi wajha se aaj kal toh koi darwaaze k andar bhi kisi ko nahi chorte

RA: elaaj aap ne kahan se kiya hai

SS: yahan se karti thi mein

RA: acha toh aap is elaaj ki wajha se joh center pe hota hai

SS: bohat kehte hain nahi jaaya karo is jagon pe

RA: hmm

SS: toh yeh jagha acha nahi hai bachon k liye acha nahi hai hifazati teeke ache nahi hai lekin nahi mein ne bola is tarhan nahi bola karo yeh joh aati hain koi na koi faida hai hamara bhi hai woh kehte hain hamara nahi inka bhi hoga faida

RA: hmm

SS: isi wajha se aate jaate hain

RA: acha in pe bhi aitebaar isliye karte hain kyun center se aap mutmaain hain

SS: jee

RA: toh aap ko yeh hota hai k who wahan se aa rahi hain is wajha se

SS: jee

RA: center aap ko pata hai k kis ka hai kis ne khola hai

SS: yeh toh mein ne kuch pata nahi kiya kehte hain jab aate hain k aga khan se aaye hain rehri goth center se jaate hain toh rehri goth jaate hain

RA: hmm

SS: case wagera karna hota hai toh kohigoth jaate hain

RA: hmm

SS: yahan pe mashallah bohat sahulat wahan pe bhi hai acha elaaj hojaata hai shukar mere bache normal pe huye ek raat gaye toh dusre din ajaate hain

RA: hmm toh aga khan or kohigoth ki is wajha hai k aap in k upar aitebaar karte ho worker wahan se arahe hain aga khan ki taraf se arahe hain un k naam ki wajha se

SS: bas mein ne bola yehi aitebaar hai woh log kehte hain aga khan se aayi hun toh bas bitha deity hun jaise abhi aap ne card dikhaya mujhe

RA: hmm

SS: ab card aap ne mujhe dikha diya un ko toh card wagera nahi diya bas unki shRAal wagera deRAh leiti hun koi cheez deRAh leiti hun ziada aisi cheez mera naam leti hain pata lag jaata hai thori har kisi ko naam pata hai

RA: hmm acha toh matlab idaare ki wajha se aitebaar rRAhte hain aap aga khan ka koi acha waqiya hua hai aga khan ki tarhan ya kyun aga khan k upar aitebaar hai naam hai bara idaara hai aur aap ka acha woh raha jab aap gayi

SS: aitebaar hai isi wajha se

RA: aap ka aitebaar hai us k upar

SS: jee

RA: thek hai acha mujhe yeh batao k inhon ne aap ko idaare se jora hai yeh joh center hai hamara CHW ne

SS: nahi is baat ka toh mujhe pata nahi

RA: nahi jab yeh aati hain yeh bataati hai k center k upar kia kia services mil rahi hai kia kia horaha hai aap aayein hamare center pe chRAkar lagaayein aisa kuch

SS: nahi nahi aisa kuch bhi nahi

RA: aap ko center se kaise pata chala center ka

SS: yeh rehrigoth ka

RA: hmm

SS: toh yahan pe meri nand le k jaati thi yahan pe elaaj acha hota hai bachon ka bas mein gayi mujhe pata nahi tha bas mein phir gayi bohat kehte thy nahi jao yahan acha nahi hai na har kisi ka apna apna hisaab hota hai

RA: kon kehta hai yeh

SS: yeh bahar k log bolte thy yeh elaaj sahi nahi karte yeh nahi lekin hum ko aitebaar hai

RA: aap gayin aap ko sahi laga

SS: haan

RA: aap mutmaain huyin

SS: jee

RA: thek hai acha mujhe batao aap ko lagta hai k worker aati hai is ko munasib training haasil hai munasib taaleem mili hai joh aap se yeh kaam karti hai poochti hai

SS: jee munasib hai joh taleem jahan pe kaam karte hain wahan taleem k bagair kaam nahi hota jahan pe taleem hoga toh kaam hoga na jaise jaahil honge toh kaise kaam kar sRAeinge

RA: hmm

SS: taleem hogi isi wajha se kyun k yahan idhar udhar jaate hain

RA: in k kaam mein koi kami lagti hai k kami hai kuch acha hona chaiye

SS: nahi kuch nahi

RA: kuch nahi isko kaise behtar banaayein mashwara deina chahogi

SS: behtari abhi bhi mashaallah hai

RA: hmm koi cheez joh aap batana chahti hain khul k bata sRAti hain

SS: mashaallah sahi hai

RA: acha yeh aati hain jaise har ghar jaati hain na inka kaam hai har ghar jaati hain toh in k zariye agar hum aap ko maloomaat pohchana chahein woh konsi maloomaat hogi joh aap leina chaheingi kis cheez k baare mein apni sehat k hawale se koi masla ho

SS: yeh mein kehti hun yahan pe aurat ka elaaj jaise k koi dawaai wagera ho

RA: hmm

SS: aise aurton ka bhi ho toh yeh achi baat hogi

RA: woh toh center pe hogaya na center pe ho is se agar center pe

SS: jaise k delivery hojaaye toh bas aurat ka elaaj khatam hojaati hai aurtein nahi aayeingi bache sirf aayein aurtein bhi aani chaiye jaise woh bhi toh maayein jaise meri tabiat kharaab hai lekin jana nahi hosta kyun k mera card nahi banta bachon ka card banta hai bachon ko le jaate hain aise hamara bhi hota elaaj toh bara acha hota

RA: haan toh us se worker k kaam pe asar parta hai matlab k aap kehte ho k bachon ka elaaj hota hai sirf hamla aurton ka elaaj hota hai toh in k kaam pe koi asar parta hai yeh joh larkiyaan aati hain aap k ghar pe

SS: nahi koi aisa nahi

RA: aap link banaati hogi k yeh sirf haamla aurton se pooch k chali jaati hain yeh aisa sochte hain na aap log

SS: yeh sochte toh hain zaahir hai jab hamal ho phir aate hain

RA: hmm

SS: hamal nahi ho toh bas darwaaze se hi pooch k chale jaate hain aur kuch nahi

RA: aur kuch nahi acha thek hai aap yeh keh rahi hain k dusre logon ka bhi poochna chaiye yehi mein poochna chah rahi hun yeh agar aaye aap k ghar jaise aap k koi masla masaail hai elaaj toh dusri baat hai na pehle yeh aati hain phir aap log center jaate hain

SS: jee

RA: thek hai agar inhi k zariye hum aap ka koi masla hal kar sRAe woh kia hai joh aap inse sunna chahogi matlab yeh aap ko a k bata dein koi aap ko information de dein hamal k doraan ya hamal na ho us k elaawa tab koi aisi cheez joh aap in se sunna chahein

SS: bas hamari madad kar rahi hain yehi achi baat hai garibon ka joh insaan madad kare Allah un ki madad karta hai

RA: madad kis tarhan se

SS: har kisi ka jaise k elaaj bhi karna ho madad yeh

RA: hmm

SS: insaan dusre insaanse achi baat bhi kare pyaar se mohabbat se baat kare woh bhi insaan k liye bohat bari baat hai

RA: aap kehti hain k bas joh yeh kaam kar rahi hain woh kaafi hai

SS: kaafi hai

RA: acha aur kuch batana chaheingi samina

SS: nahi meharbaani

RA: shukriya bohat bohat……
